# Understanding of Waggle Dance in the Honey Bee (*Apis mellifera*) from the Perspective of Long Non-Coding RNA

**DOI:** 10.3390/insects13020111

**Published:** 2022-01-19

**Authors:** Wangjiang Feng, Jingnan Huang, Zhaonan Zhang, Hongyi Nie, Yan Lin, Zhiguo Li, Songkun Su

**Affiliations:** 1 College of Animal Sciences (College of Bee Science), Fujian Agriculture and Forestry University, Fuzhou 350002, China; RoyalJelly1995@outlook.com (W.F.); jingnan.huang@outlook.com (J.H.); hnhynie@fafu.edu.cn (H.N.); ylin19@fafu.edu.cn (Y.L.); 2 Laboratory of Evolution and Diversity Biology (EDB), UMR5174, University Toulouse III Paul Sabatier, CNRS, 31062 Toulouse, France; zhaonan.zhang@univ-tlse3.fr

**Keywords:** honey bee, brain, long non-coding RNA, lncRNA-mRNA association analysis, waggle dance

## Abstract

**Simple Summary:**

Dance behaviour of honey bee (*Apis mellifera*) is highly sophisticated and a unique behavioural pattern that ensures effective and high-quality communication of food information. Honey bee dance behaviour has been discovered and elaborated for decades, but the regulatory mechanism underlying this behaviour is still unclear. In this study, by varying the food quality, such as the concentration of sucrose solution, we successfully manipulated the dance behaviour of honey bees. Then we investigated the effect of long non-coding RNAs (lncRNAs) and their target genes in honey bee brains on waggle dance. The results indicated that lncRNAs in brains of waggle dancers and non-dancing bees exhibited significant differences. Furthermore, lncRNA-mRNA association analysis showed that signal transduction in the brain may be participated in the modulation of waggle dance. Our findings suggested that neurotransmitters presumably served as messengers in the waggle dance. It is the first time that the waggle dance in the honey bee is studied from the perspective of long non-coding RNA. Taken together, this study is expected to provide a new pathway to explore the relationship between behaviour and brain.

**Abstract:**

The ethological study of dance behaviour has yielded some findings since Karl Von Frisch discovered and interpreted the ‘dance language’ in the honey bee. However, the function and role of long non-coding RNAs on dance behaviour are hardly known until now. In this study, the differential expression patterns of lncRNAs in the brains of waggling dancers and non-dancing bees were analysed by RNA sequencing. Furthermore, lncRNA-mRNA association analysis was constructed to decipher the waggle dance. The results of RNA sequencing indicated that a total of 2877 lncRNAs and 9647 mRNAs were detected from honey bee brains. Further comparison analysis displayed that two lncRNAs, MSTRG.6803.3 and XR_003305156.1, may be involved in the waggle dance. The lncRNA-mRNA association analysis showed that target genes of differentially expressed lncRNAs in the brains between waggling dancers and non-dancing bees were mainly annotated in biological processes related to metabolic process, signalling and response to stimulus and in molecular function associated with signal transducer activity, molecular transducer activity and binding. Nitrogen metabolism was likely implicated in the modulation of the waggle dance. Our findings contribute to further understanding the occurrence and development of waggle dance.

## 1. Introduction

The honey bee (*Apis mellifera*), as a model organism, has been widely studied in genetics, ethology and neurobiology [1,2]. More importantly, honey bees are eusocial insects, which offer immeasurable value to the ecosystem, agriculture and human society [3]. A typical colony normally comprises one mated queen, hundreds of drones and thousands of worker bees. Owing to division of labour in a colony, adult worker bees are mainly divided into house bees (e.g., nurse bees) and field bees (e.g., forager bees) [4]. The behavioural transition from hive work to field work is seemingly dependent on age [5]. However, the brain expression of mRNAs and microRNAs in honey bees is primarily associated with behaviour and used in predicting the behavioural plasticity of honey bees [6,7]. The approach that investigates honey bee behaviour on the basis of gene expression profiles or transcriptomics may reveal how gene expression in the brain affects the occurrence of a behaviour.

The close relationship between stimuli and responses has been explored and verified. For instance, a stimulus induces one or more behavioural responses [8]. Profitable food (stimulus) is enough to activate the foraging instinct and dance behaviour (behavioural response) of honey bees [9,10]. In a natural environment, successful foragers returning from excellent food sources convey food information to followers by dancing [9]. It has been experimentally established that sweet sucrose solution as a stimulus attracts honey bees to forage and brings about the probability of dancing [10]. According to the distance of a food source from a hive, dance behaviour is mainly divided into round dance and waggle dance [11]. The round dance is usually used to advertise food located around the hive (<50 m) and cannot indicate the vector information of the food site [12,13]. When a food location is beyond 100 m, dancers interact with followers through waggle dance, which provides not only the distance and direction information of a food source but also the odour and profitability of the food [14,15,16]. Intriguingly, the more profitable the food source is, the more likely the switch of controlling dancing is to be turned on [17]. In this process, foragers need to evaluate food profitability and gauge the location of food sources in the foraging period, as well as transmit information of food sources in the dancing period. Thus, from the broader definition, the food collection process of honey bees mainly includes foraging and dancing activities.

Neurochemicals in the brain probably act as messengers, that is, physical signals are converted into chemical signals, thereby resulting in the presentation of dance behaviour [18]. It has been reported that neurotransmitters participated in the modulation of numerous behaviour patterns in honey bees, such as mating, foraging and dancing behaviour [19,20,21]. The neural mechanism underlying dance behaviour in honey bees has been explored for decades [22]. Current research has shown that brain octopamine modulates the reporting of food value in the dance behaviour of honey bees [23]. The effect of octopamine on dance behaviour is restricted by the octopamine antagonist mianserin [23,24]. Thus, octopamine may function as a messenger for the self-assessment of food value in appetitive learning and memory. Likewise, γ-aminobutyric acid (GABA) and glutamate are involved in sensory information processing and integration in honey bees, particularly in olfactory learning and memory in antennal lobes [25]. Honey bee dance communication is a sophisticated and stereotyped behavioural process, in which multiple sensory inputs, processing and integration are involved. These findings indicated that neurotransmitters are implicated in signalling in dance communication.

Some achievements have been made in the study of honey bee dance behaviour, but the regulatory mechanism underlying dance behaviour still remains unknown. In particular, the effect of long non-coding RNAs on dance behaviour is even more obscure. lncRNAs constitute a class of non-coding RNAs that are over 200 nt in length, contain two or more exons, have no protein-coding ability and show time- and tissue-specific properties [26]. Rather than dark matter or junk genes previously proposed by some researchers, lncRNAs are regulatory factors that play a vital role in the developmental processes of organisms [27]. Apart from regulating the expression of target genes through signals, decoys, guides or scaffolds, lncRNAs can interact with target genes via the antisense-, cis- or trans-regulatory mechanism [28,29]. Some antisense lncRNAs may bind to the mRNAs of sense strands to regulate gene silencing, transcription and mRNA stability [30]. The basic principle of the cis-regulatory mechanism suggests that the function of lncRNAs is related to their adjacent protein-coding genes [29]. In general, lncRNAs within 10 kb upstream or downstream of protein-coding genes regulate gene expression at the transcriptional or post-transcriptional level [31]. By contrast, the basic principle of the trans-regulatory mechanism proposes that the putative target genes can be predicted by correlation analysis or co-expression analysis between lncRNA and protein-coding genes [29].

Long non-coding RNAs have been found in insects, such as fruit fly (*Drosophila melanogaster*), silkworm (*Bombyx mori*) and honey bee (*Apis mellifera*), which are related to neural activities, silk synthesis and caste differentiation, as well as other physiological processes [32,33,34]. In *Apis mellifera*, the potential functions of some non-coding RNAs were identified, such as *Nb-1*, which is related to the division of labour [35], *Ks-1*, which is associated with the regulation of neural function in the honey bee brain [36], *AncR-1*, which is involved in the functional regulation of multiple tissue activities [37] and *kakusei*, which is implicated in foraging activity [38,39]. Furthermore, some differentially expressed genes in the brain involved in the vibration signal communication of honey bees were discovered using microarray analysis, which first explores the neurogenomic expression profile associated with honey bee communication [40]. However, in contrast to vibration signal communication, disclosing the neurogenomics of dance communication is more helpful to shed light on the relationship between neural system and signal communication. The ‘dance language’ of honey bees, especially waggle dance, is unique in insects. At the level of transcriptomics, gene expression profiles in mushroom bodies (MBs), optic lobes, the central brain and the second thoracic ganglion were detected in three honey bee species: *Apis mellifera*, *Apis dorsata* and *Apis florea* [41]. The results showed that gene expression profiles in the MBs displayed the most obvious differences as compared with other parts across three species of honey bees. As shown by the results from our team, differentially expressed microRNAs were found in the heads of foragers and dancers, in which some microRNAs, such as ame-miR-278 and ame-miR-282, may be involved in the regulation of dance behaviour [42]. A recent study revealed the role of lncRNAs in behavioural transition from nurses to foragers in *Apis mellifera* by RNA sequencing [43]. Even though the mRNAs and microRNAs related to dance behaviour have been detected, the function and role of lncRNAs underlying dance behaviour in honey bees have not been elucidated.

In the present work, we intend to explore the effect of lncRNAs on the waggle dance from the biological process, molecular function and cellular component, as well as to detect the related pathway modulating the waggle dance. We expect that the study contributes to further knowledge of the mechanism underlying dance behaviour and can provide a new horizon for investigating the relationship between behaviour and brain.

## 2. Materials and Methods

### 2.1. Bees and Bee Training

Honey bees (*Apis mellifera*) were maintained at Fujian Agriculture and Forestry University (Fuzhou, China). Experimental colonies were housed in an observation hive with two frames, including one food comb containing some honey and pollen, one brood comb using for laying eggs, approximately 4000 worker bees and a mated queen. A feeder with 1.5 M sucrose solution was used in directing foragers to the destination in the open meadow, which was 200 m away from the observation hive. When foraging at the feeder, the honey bees were captured with soft forceps, and then their thoraxes were marked with number tags for identification. Only marked bees that performed the waggle dance on the comb were considered subsequent subjects. During the experiment, 40 honey bees were marked the day before the experiment began. Then we will capture a few of them in each of the four stages. The same sampling process lasted for several days.

### 2.2. Behavioural Observation and Sampling under Different Concentrations of Sucrose Solution

The experiments were conducted from 9 am to 1 pm in November 2020. To regulate the waggle dance of the honey bees, two different concentrations of sucrose solution were used in setting up three variations (1.5 M→0.5 M→1.5 M). The concentrations of 0.5 M and 1.5 M sucrose solution correspond to 17% and 51% sucrose solution (*w*/*v*), respectively. Preliminary experiments showed that honey bees performed waggling dance after collecting 1.5 M sucrose solution; but honey bees only collected and did not dance under the concentration of 0.5 M sucrose solution. If the concentration of sucrose solution in the feeder is higher, such as 2 M, the feeding process is hard to honey bees; and if the concentration is below 0.5 M, the sucrose solution is not attractive to honey bees [11,17]. Moreover, a three-fold difference in sucrose solution concentrations is enough to induce differences in behaviour. Eventually, the concentrations of 1.5 M and 0.5 M sucrose solution were chosen in this study.

**Figure 1:**
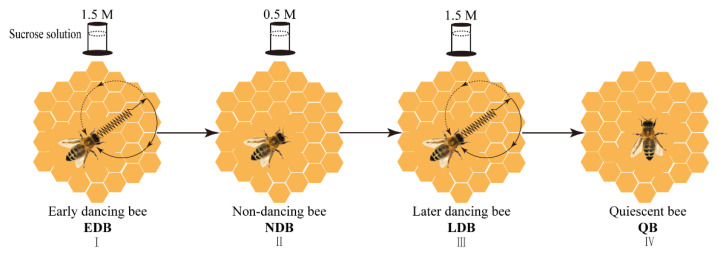
The diagram of the experimental procedure. By varying the concentration of sucrose solution in the feeder (1.5 M→0.5 M→1.5 M) that is located 200 m from the observation hive, the occurrence of the waggle dance could be manipulated, that is, foragers performed waggling dance, non-dancing and waggling dance again. After the feeder was removed for 1 h, the honey bees that kept a quiescent state were sampled. Note: EDB referred to the waggling dancers after foraging on a feeder with 1.5 M sucrose solution (**I**). NDB referred to the non-dancing bees after foraging on another feeder with 0.5 M sucrose solution replacing the feeder with 1.5 M sucrose solution (**II**). LDB referred to the waggling dancers after foraging on the feeder with 1.5 M sucrose solution replacing the feeder with 0.5 M sucrose solution (**Ⅲ**). QB referred to quiescent bees on the comb after removing the feeder for 1 h (**IV**).

By changing the concentration of sucrose solution in the feeder, the behaviour of honey bees was observed in the observation hive. Finally, each group of 30 honey bees was sampled. The detailed procedures were as follows (Figure 1):

In the first phase, a feeder with 1.5 M sucrose solution was placed at the destination. The marked foragers that performed the waggle dance after returning to the observation hive were defined as early dancing bees (EDB) after three continuous observations of foraging and waggling dance. On the third waggling dance, several EDB were sampled.

In the second phase, the feeder with 1.5 M sucrose solution was replaced with another feeder with 0.5 M sucrose solution. The remaining marked foragers that performed the waggle dance in the first phase but did not dance in the second phase were defined as non-dancing bees (NDB) after three continuous observations of foraging but non-dancing. On the return of the third collection, several NDB were sampled.

In the third phase, the feeder with 0.5 M sucrose solution was replaced with a feeder with 1.5 M sucrose solution. The remaining marked bees that did not dance in the second phase but performed the waggle dance again were defined as later dancing bees (LDB) after three continuous observations of foraging and waggling dance. Similarly, on the third waggling dance, several LDB were sampled.

In the fourth phase, the feeder was removed for 1 h. The remaining marked bees that were from the first three phases, which kept a relatively quiescent state in the hive (almost no moving), such as hanging quietly on the comb, were defined as quiescent bees (QB) and then sampled.

The honey bees in the four phases were individually sampled and quickly frozen in the liquid nitrogen. The heads of the honey bees were separated and placed in a refrigerator (Haier, Qingdao, China) at −80 °C for brain dissection.

### 2.3. Brain Dissection of the Honey bees

The brain of each honey bee was fetched on a special dissecting dish covered with dry ice, which kept the entire brain constantly frozen to prevent degradation. The head capsule of the honey bee was exposed using scalpel and forceps, and then the hypopharyngeal glands, the suboesophageal ganglion and ommochrome on the compound eyes were removed and discarded. The whole brain was individually transferred into a 1.5 mL microtube (Corning, Tewksbury, MA, USA) and stored at −80 °C in the refrigerator until further analysis [44].

### 2.4. RNA Sequencing Analysis from Four Groups of Honey bee Brains

The brains from the above four groups of bees (EDB, NDB, LDB and QB) were respectively used for total RNA extraction. To meet the RNA-seq requirement, 10 brains from bees in one group were pooled as a sample. Each group of bees from the same colony has three biological replicas (e.g., EDB-1, EDB-2 and EDB-3). The total RNA of each sample was extracted using Trizol reagent kit (Invitrogen, Carlsbad, CA, USA) according to the manufacturer’s protocol. After the assessment of RNA quality using Agilent 2100 Bioanalyzer (Agilent Technologies, Palo Alto, CA, USA) and RNase free agarose gel electrophoresis, the rRNAs were removed to ensure the quality and efficiency of cDNA transcripted reversely. Then, QiaQuick PCR extraction kit (Qiagen, Venlo, The Netherlands) was used to purify the synthesised cDNA fragments. The purified products were end-repaired, poly(A)-added and ligated to Illumina sequencing adapters. The ligation products were selected through agarose gel electrophoresis according to size difference and then amplified by PCR. Eventually, the final products from the four groups of honey bee brains were respectively sequenced using Illumina HiSeq2500 by Gene Denovo Biotechnology Co., Ltd., (Guangzhou, China) [45]. The RNA sequencing data in this study have been uploaded to the NCBI Sequence Read Archive (SRA) (http://www.ncbi.nlm.nih.gov/sra/, accessed on 5 November 2021) with the BioProject ID PRJNA760337.

### 2.5. Bioinformatic Analysis of RNA Sequencing Data

The raw reads contained adapters or low-quality bases, and thus clean reads filtered by fastp (version 0.18.0) were used for follow-up bioinformatics analysis [46]. The reads mapped with the ribosome RNA (rRNA) database will affect the following assembly and gene abundance calculation. Bowtie2 (version 2.2.8), which functioned as a tool to map reads to the rRNA database, was used to rule out remaining rRNAs in clean reads [47]. Then, the reads unmapped with the rRNA database were mapped to the reference genome of *Apis mellifera* (assembly Amel_HAv3.1) with HISAT2 (version 2.1.0), which is currently the most accurate alignment software with the highest alignment rate [48]. Given that lncRNAs generated by the different transcripts of the same gene greatly vary, lncRNAs were analysed based on transcripts. Therefore, Stringtie (version 1.3.4) and HISAT2 were used in the reconstruction of transcripts [49,50]. By means of Cufflinks (version 2.1.1), novel transcripts were defined by aligning reconstructed transcripts to the reference genome of *Apis mellifera* (assembly Amel_HAv3.1) based on the parameters that the length of the transcript was longer than 200 bp and the exon number was more than two [51]. After transcripts with protein-coding potential were eliminated, novel lncRNAs were obtained with the non-protein-coding potential results from the intersection of CNCI (version 2) and CPC2 (version 0.9-r2) [52,53]. To compare the difference of transcript expression among samples, expression abundances of transcripts were quantified with software StringTie according to FPKM (fragment per kilobase of transcript per million mapped reads) value. Relationship analysis of samples was performed as well.

To determine whether lncRNAs have a potential effect on the behavioural phenotype of honey bees, we compared the differentially expressed profiles of lncRNAs among different groups by DESeq2 [54]. The differentially expressed lncRNAs (DElncRNAs) in two groups were defined according to the parameters that *p*-value < 0.05 and |log_2_FC| > 1. The food collection process of honey bees mainly includes foraging and dancing activities. In this study, compared with quiescent bees (QB) that remained a relatively stable state on the comb, non-dancing bees (NDB) underwent foraging activity, and dancing bees (EDB and LDB) performed foraging activity and the waggle dance. Therefore, DElncRNAs associated with foraging activity were identified in QB-vs-NDB. DElncRNAs associated with the waggle dance were identified in NDB-vs-EDB and NDB-vs-LDB. DElncRNAs associated with foraging activity and the waggle dance were identified in QB-vs-EDB and QB-vs-LDB. By calculating the intersection between NDB-vs-EDB, NDB-vs-LDB, QB-vs-EDB and QB-vs-LDB, DElncRNAs associated with the waggle dance were further identified. Similarly, DElncRNAs associated with foraging activity were further identified by calculating the intersection between QB-vs-EDB, QB-vs-LDB and QB-vs-NDB.

Subsequently, lncRNA−mRNA association analysis was performed on the basis of differentially expressed lncRNAs and mRNAs. In this study, we predicted the putative target genes regulated by DElncRNAs through the following three pathways: (1) The antisense regulatory mechanism: the putative target genes were defined by analysing the binding of antisense DElncRNAs and the mRNAs of sense strands; (2) the cis-regulatory mechanism: the genes located within 10 kb upstream or downstream of DElncRNAs were defined as the putative target genes; (3) the trans-regulatory mechanism: the putative target genes were predicted by correlation analysis between DElncRNA and protein-coding genes. Then the putative target genes regulated by DElncRNAs in the way of antisense, cis- or trans-regulatory mechanism were subjected to enrichment analysis of GO (Gene Ontology) function and KEGG (Kyoto Encyclopedia of Genes and Genomes) pathway [55,56]. Bioinformatic analysis was performed with the Omicsmart online platform (http://www.omicsmart.com, accessed on 5 November 2021) developed by Gene Denovo Biotechnology Co., Ltd., (Guangzhou, China).

### 2.6. Real-Time Quantitative PCR (RT-qPCR) Validation of DElncRNAs

To determine whether RNA sequencing results were reliable, 8 DElncRNAs (MSTRG.3703.1, XR_003305349.1, XR_001702399.2, XR_003304525.1, XR_003304191.1, MSTRG.8314.1, XR_001706524.2 and MSTRG.3555.3) were randomly selected. Due to the large number of DElncRNAs validation through RT-qPCR, total RNAs from each group of the honey bee brains obtained by the same sampling method were isolated again using Trizol reagent kit (Invitrogen, Carlsbad, CA, USA) in accordance with the manufacturer’s protocol and then used for real-time quantitative PCR to examine the reliability of RNA sequencing data. RNAs were transcribed reversely to cDNA using HiScript^®^ II Q RT SuperMix for qPCR (+gDNA wiper) (Vazyme, Nanjing, China) according to the manufacturer’s protocol. The specific primers for qPCR were designed using Primer Premier 6.0 and synthesised by Sunya Biotechnology Co., Ltd., (Hangzhou, China) (Table S1). The housekeeping gene *actin* was used as an internal control [43,45].

Real-time quantitative PCR (RT-qPCR) was performed using ChamQ Universal SYBR qPCR Master Mix (Vazyme, Nanjing, China) on C1000 TouchTM Thermal Cycler (BIO-RAD, Hercules, CA, USA). The 10 µL qPCR reaction mixture consisted of 5 µL of 2 × ChamQ Universal SYBR qPCR Master Mix, 0.2 µL of specific forward primer, 0.2 µL of reverse primer, 1 µL of cDNA and 3.6 µL of ddH_2_O. The reaction conditions of qPCR were as follows: 95 °C for 1 min, followed by 40 cycles of 95 °C for 15 s and 60 °C for 30 s, as well as 95 °C for 5 s; finally, temperature was increased from 65 °C to 95 °C at 0.5 °C increment every 5 s until plate reading in order to melting curve analysis. Each sample in each reaction was performed technically in triplicate. The relative expression of lncRNAs was displayed using the 2^−∆CT^ method [57].

### 2.7. Statistical Analysis

qPCR data analysis was conducted using SigmaPlot 14.0. The results were shown as mean ± standard error. *p* < 0.05 was defined as statistically significant. For the calculation of the relative expression amount of lncRNAs, qPCR data were analysed using *t*-test.

## 3. Results

### 3.1. Quality Control and Evaluation of RNA Sequencing Results

Approximately 79–106 million raw reads from 12 samples were respectively obtained by RNA sequencing, and clean reads accounted for more than 99.82% of the raw reads among 12 samples after low-quality data were eliminated with fastp. In addition, the Q20 values of the 12 samples ranged from 96.96% to 98.61%, whereas, Q30 values ranged from 91.21% to 95.81%. The percentage of clean reads mapped with the ribosome RNA database ranged from 15.45% to 19.95%. Then the unmapped reads were used for subsequent analysis (Table S2).

### 3.2. LncRNA-Seq Results from the Four Groups of Honey bee Brains

According to the lncRNA-seq results, a total of 2877 lncRNAs were detected from four groups of honey bee brains, including 2445 known lncRNAs and 432 novel lncRNAs (Table S3). Among them, 432 novel lncRNAs were defined with the intersection between CPC2 and CNCI based on the transcripts reconstructed by stringTie (Figure S1, Table S4). Based on the expression results of 12 samples, the correlation coefficients among the samples of each group of bees were more than 0.98 (Figure S2a), together with PCA results (Figure S2b), demonstrating the well repeatability of the samples sequenced. Thus, the sequenced results were used for further analysis.

Using software DESeq2 to analyse the sequenced data, DElncRNAs were defined according to the parameters that *p*-value < 0.05 and |log_2_FC| > 1. As shown by the analysis results (Table 1), DElncRNAs were identified between any two groups of honey bee brain samples. Among them, 37 DElncRNAs were detected in NDB-vs-EDB, including 19 up-regulated and 18 down-regulated DElncRNAs in EDB; 33 DElncRNAs were detected in NDB-vs-LDB, including 14 up-regulated and 19 down-regulated DElncRNAs in LDB; 50 DElncRNAs were detected in QB-vs-EDB, including 19 up-regulated and 31 down-regulated DElncRNAs in EDB; 29 DElncRNAs were detected in QB-vs-LDB, including 6 up-regulated and 23 down-regulated DElncRNAs in LDB; and 45 DElncRNAs were detected in QB-vs-NDB, including 16 up-regulated and 29 down-regulated DElncRNAs in NDB.

**Figure 2:**
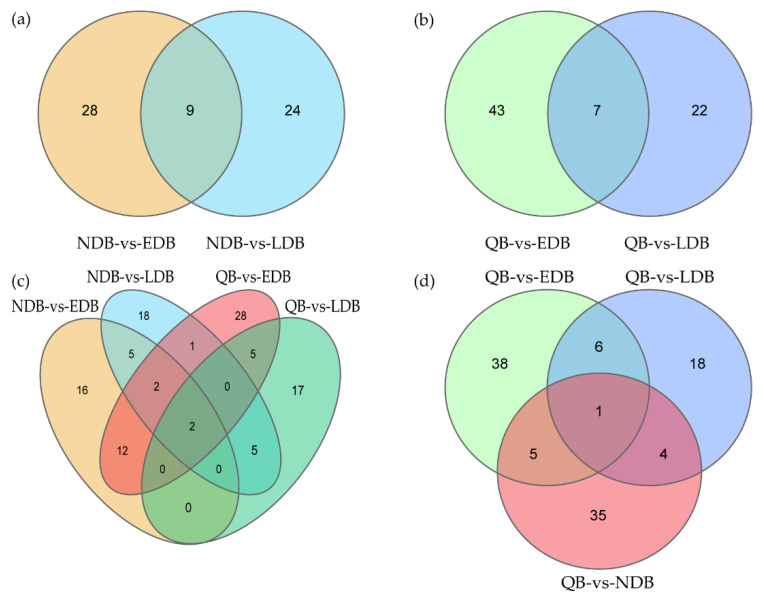
Venn diagram of differentially expressed lncRNAs in honey bees of different comparison groups. Based on the comparison results of different groups of honey bees, some specific and common DElncRNAs were identified using the Venn diagram. (**a**) Venn diagram of DElncRNAs between NDB-vs-EDB and NDB-vs-LDB. (**b**) Venn diagram of DElncRNAs between QB-vs-EDB and QB-vs-LDB. (**c**) Venn diagram of DElncRNAs between NDB-vs-EDB, NDB-vs-LDB, QB-vs-EDB and QB-vs-LDB. (**d**) Venn diagram of DElncRNAs between QB-vs-EDB, QB-vs-LDB and QB-vs-NDB. Note: EDB referred to the waggling dancers after foraging on a feeder with 1.5 M sucrose solution in the first phase. NDB referred to the non-dancing bees after foraging on another feeder with 0.5 M sucrose solution replacing the feeder with 1.5 M sucrose solution in the second phase. LDB referred to the waggling dancers after foraging on the feeder with 1.5 M sucrose solution replacing the feeder with 0.5 M sucrose solution in the third phase. QB referred to quiescent bees on the comb after removing the feeder for 1 h in the fourth phase.

Given that both EDB and LDB are waggling dancers, Venn diagram was used in further identifying the DElncRNAs between waggling dancers and non-dancing bees. Nine DElncRNAs were shared between NDB-vs-EDB and NDB-vs-LDB, namely, MSTRG.3673.3, MSTRG.6803.3, XR_001703122.2, XR_003304535.1, XR_003304660.1, XR_003304701.1, XR_003305156.1, XR_003305397.1 and XR_003305473.1 (Figure 2a). Similarly, 7 DElncRNAs were shared between QB-vs-EDB and QB-vs-LDB, namely, MSTRG.1195.1, MSTRG.6803.3, NR_003565.1, XR_001706449.2, XR_003304336.1, XR_003304726.1 and XR_003305156.1 (Figure 2b). Compared with NDB that only underwent foraging activity, EDB and LDB performed foraging activity and the waggle dance. Therefore, further comparison analysis showed that 2 DElncRNAs (MSTRG.6803.3 and XR_003305156.1), which may be involved in the waggle dance, were shared by the above two intersections (Figure 2c). Compared with QB that kept a relatively stable state on the comb, EDB, LDB and NDB had a common feature that was the foraging activity. The comparison result showed that 1 DElncRNA, NR_003565.1, was shared between QB-vs-EDB, QB-vs-LDB and QB-vs-NDB, which may be involved in the foraging activity (Figure 2d).

### 3.3. GO and KEGG Analysis of the Target Genes of lncRNAs

#### 3.3.1. GO and KEGG Analysis of Target Genes Regulated by DElncRNAs in NDB-vs-EDB and NDB-vs-LDB via the Antisense Regulatory Mechanism

Comparison analysis showed that 9, 2 lncRNAs of 37, 33 DElncRNAs in NDB-vs-EDB and NDB-vs-LDB were respectively deduced to target 8, 2 genes via the antisense regulatory mechanism. The GO enrichment results showed that five terms of biological process, such as signalling and response to stimulus, were shared by NDB-vs-EDB and NDB-vs-LDB. The top 20 terms of GO enrichment between the two comparisons were mostly shared in signalling and response to stimulus as well (Figure S3). The KEGG pathway enrichment result in NDB-vs-EDB was mainly related to metabolic processes, such as other glycan degradation, starch and sucrose metabolism and galactose metabolism (Figure S4). No data about KEGG analysis were obtained in NDB-vs-LDB.

#### 3.3.2. GO and KEGG Analysis of Target Genes Regulated by DElncRNAs in NDB-vs-EDB, NDB-vs-LDB, QB-vs-EDB, QB-vs-LDB via the Cis-Regulatory Mechanism

In this study, 19, 18, 28, 18 lncRNAs of 37, 33, 50, 29 differentially expressed lncRNAs in NDB-vs-EDB, NDB-vs-LDB, QB-vs-EDB, QB-vs-LDB were respectively deduced to target 50, 33, 79, 41 genes via the cis-regulatory mechanism. GO enrichment analysis indicated that the putative target genes were commonly annotated in eight terms of biological process (e.g., signalling, metabolic process and response to stimulus), two terms of molecular function (catalytic activity and binding) and four terms of cellular component (e.g., cell, membrane and organelle) (Table S5). Besides QB-vs-LDB, the other three comparisons were also shared in two terms of molecular function (signal transducer activity and molecular transducer activity). KEGG pathway enrichment analysis suggested that the putative target genes were commonly enriched in three pathways (i.e., nitrogen metabolism, metabolic pathways and ribosome) (Table S6).

#### 3.3.3. GO and KEGG Analysis of Target Genes Regulated by DElncRNAs from the Intersection between NDB-vs-EDB and NDB-vs-LDB via the Cis-Regulatory Mechanism

**Figure 3:**
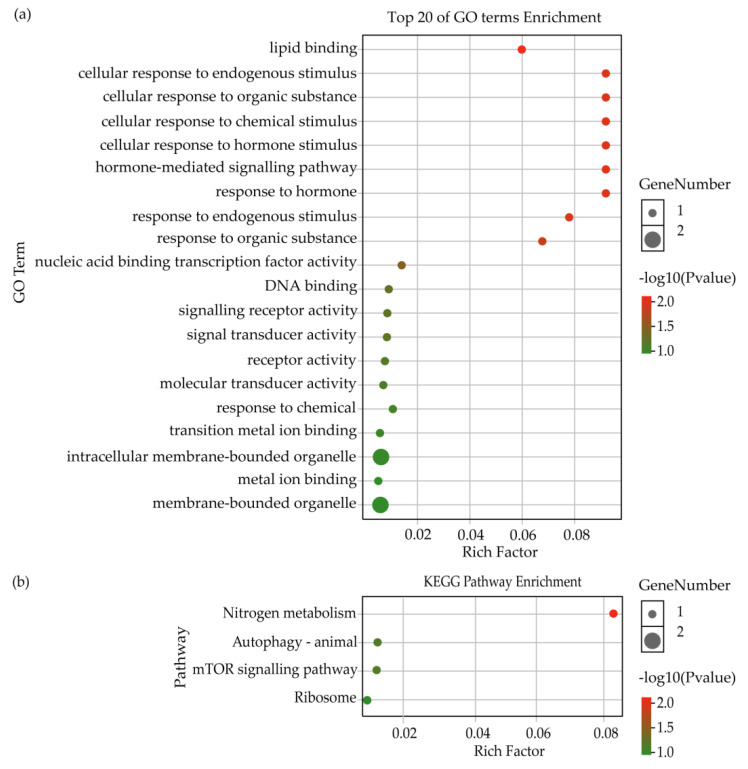
GO and KEGG enrichment analysis of target genes regulated by DElncRNAs from the intersection between NDB-vs-EDB and NDB-vs-LDB via the cis-regulatory mechanism. (**a**) Top 20 of GO terms enrichment analysis. (**b**) KEGG pathway enrichment analysis. Note: EDB referred to the waggling dancers after foraging on a feeder with 1.5 M sucrose solution in the first phase. NDB referred to the non-dancing bees after foraging on another feeder with 0.5 M sucrose solution replacing the feeder with 1.5 M sucrose solution in the second phase. LDB referred to the waggling dancers after foraging on the feeder with 1.5 M sucrose solution replacing the feeder with 0.5 M sucrose solution in the third phase.

Further analysis showed that 5 lncRNAs of 9 DElncRNAs from the intersection between NDB-vs-EDB and NDB-vs-LDB were deduced to target 17 genes via the cis-regulatory mechanism (Table S7). Among them, it was found that DElncRNA (MSTRG.6803.3) was accompanied by three differentially expressed genes (ncbi_411861, ncbi_411862 and ncbi_411863). GO enrichment analysis showed that the putative target genes were annotated in seven terms of biological process (e.g., metabolic process, signalling and response to stimulus), four terms of molecular function (e.g., signal transducer activity, molecular transducer activity and binding) and three terms of cellular component (cell, cell part and organelle) (Figure S5). The top 20 terms of GO enrichment were mostly associated with response to stimulus and signalling (Figure 3a). The KEGG pathway result suggested that the putative target genes were also enriched in nitrogen metabolism (Figure 3b).

#### 3.3.4. GO and KEGG Analysis of Target Genes Regulated by DElncRNAs from the Intersection between QB-vs-EDB and QB-vs-LDB via the Cis-Regulatory Mechanism

Likewise, 6 lncRNAs of 7 DElncRNAs from the intersection between QB-vs-EDB and QB-vs-LDB were deduced to target 19 genes via the cis-regulatory mechanism (Table S8). Among them, it was found that three DElncRNAs (MSTRG.6803.3, NR_003565.1 and XR_001706449.2) were respectively accompanied by seven differentially expressed genes (ncbi_411861 and ncbi_411862 and ncbi_411863, ncbi_410815, ncbi_107966053 and ncbi_412412 and ncbi_726766). GO enrichment analysis indicated that these putative target genes were annotated in six terms of biological process (i.e., metabolic process, biological regulation, localisation, regulation of biological process, single-organism process and cellular process), two terms of molecular function (i.e., binding and catalytic activity) and two terms of cellular component (i.e., cell and cell part) (Figure S6). The top 20 terms of GO enrichment were mostly related to homeostasis (Table S9). The KEGG pathway enrichment result suggested that the putative target genes were also enriched in nitrogen metabolism (Table S10).

#### 3.3.5. GO and KEGG Analysis of Target Genes Regulated by DElncRNAs in QB-vs-NDB via the Cis-Regulatory Mechanism

The result showed that 27 lncRNAs of 45 DElncRNAs in QB-vs-NDB were deduced to target 56 genes via the cis-regulatory mechanism. Among them, it was found that seven differentially expressed lncRNAs (NR_003565.1, MSTRG.1925.2, XR_003305695.1, MSTRG.5114.1, XR_003305121.1, XR_003306302.1 and XR_003306185.1) were respectively accompanied by seven differentially expressed genes (ncbi_410815, ncbi_411564, ncbi_552823, ncbi_411459, ncbi_411272, ncbi_413852 and ncbi_409740). GO enrichment analysis indicated that these putative target genes were annotated in nine terms of biological process (e.g., metabolic process, signalling and response to stimulus), six terms of molecular function (e.g., transporter activity, signal transducer activity and molecular transducer activity) and seven terms of cellular component (e.g., cell, membrane, organelle and macromolecular complex) (Figure S7). The top 20 terms of GO enrichment were mostly associated with biological processes, such as transport and binding (Table S11). KEGG pathway enrichment analysis suggested that the putative target genes were also enriched in nitrogen metabolism (Table S12).

### 3.4. RT-qPCR Validation of Differentially Expressed lncRNAs

**Figure 4:**
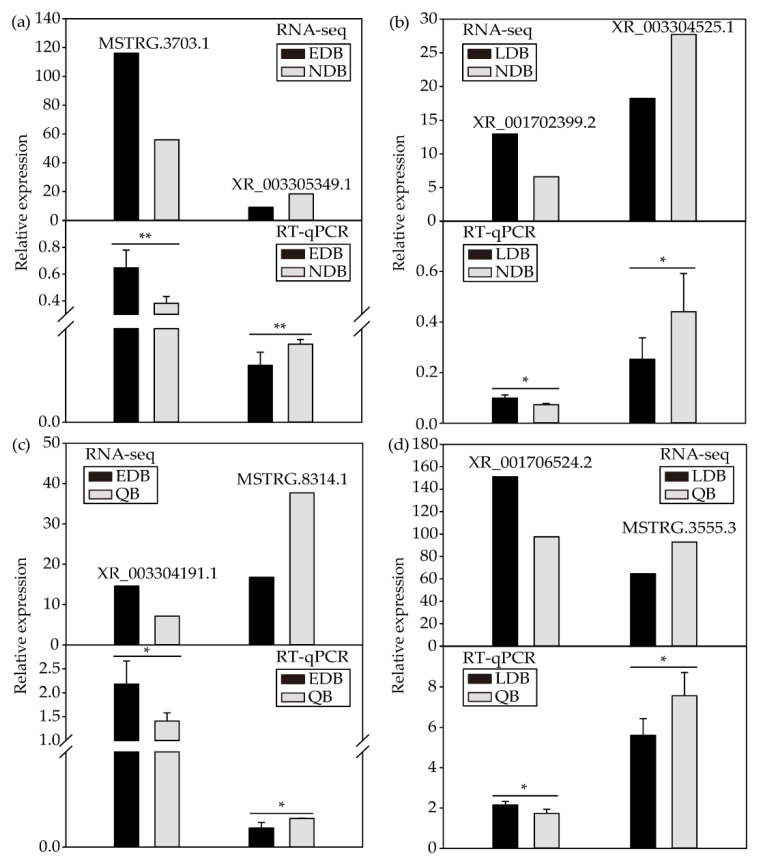
RT-qPCR validation of the expression patterns of DElncRNAs from NDB-vs-EDB (**a**), NDB-vs-LDB (**b**), QB-vs-EDB (**c**) and QB-vs-LDB (**d**). *t*-test: * *p* < 0.05; ** *p* < 0.01. Note: EDB referred to the waggling dancers after foraging on a feeder with 1.5 M sucrose solution in the first phase. NDB referred to the non-dancing bees after foraging on another feeder with 0.5 M sucrose solution replacing the feeder with 1.5 M sucrose solution in the second phase. LDB referred to the waggling dancers after foraging on the feeder with 1.5 M sucrose solution replacing the feeder with 0.5 M sucrose solution in the third phase. QB referred to quiescent bees on the comb after removing the feeder for 1 h in the fourth phase.

As shown by the results, the expression patterns of eight DElncRNAs were consistent with the RNA sequencing results, thereby indicating that the RNA sequencing data were reliable (Figure 4).

## 4. Discussion

Honey bee dance behaviour has been explored and elucidated for decades at the neuroethological, neurochemical and molecular levels [22]. In this study, we successfully modulated the waggle dance in honey bees by changing the concentration of sucrose solution. From the perspective of RNA sequencing based on the four groups of honey bee brains, it was found that 2877 lncRNAs and 9647 mRNAs were detected from the brains of honey bees. Nine differentially expressed lncRNAs were defined from the comparison between waggling dancers and non-dancing bees. Among them, comparison analysis discovered that two lncRNAs (MSTRG.6803.3 and XR_003305156.1) that were probably involved in the waggle dance of the honey bees modulated ten genes via the cis-regulatory mechanism. Meanwhile, GO and KEGG enrichment analysis respectively showed that signal transduction and nitrogen metabolism are critical to the waggle dance. There were no data about the trans-regulatory mechanism due to the less prediction of target genes.

In this study, lncRNA and mRNA profiles in honey bee brains were obtained by RNA sequencing technology, and then lncRNA−mRNA association analysis was constructed to investigate the potential relationship between genes/lncRNAs and the waggle dance of honey bees. By analysing the RNA sequencing data, nine DElncRNAs were calculated from the intersection between NDB-vs-EDB and NDB-vs-LDB and seven DElncRNAs were calculated from the intersection between QB-vs-EDB and QB-vs-LDB. From the definition of four groups of honey bees, two lncRNAs that may be associated with the waggle dance were identified by calculating the intersection of the above two comparisons. First, MSTRG.6803.3 presumably targets seven genes, namely, ncbi_725781 (Vps50), ncbi_409241 (EAG_01737), ncbi_411861 (Nkrf), ncbi_411862 (MRPS2), ncbi_411863 (DHRS7), ncbi_409636 and ncbi_409634, via the cis-regulatory mechanism. As shown in the analysis results, the expression levels of MSTRG.6803.3 in EDB and LDB were lower than in NDB and QB. The DElncRNA MSTRG.6803.3 likely resulted in the differential expression of three genes (ncbi_411861, ncbi_411862 and ncbi_411863), which were involved in the ribosome pathway, via the cis-regulatory mechanism. Second, XR_003305156.1 may target three genes, namely, ncbi_724225 (SPR), ncbi_411290 (SOCS3) and ncbi_412161 (br), via the cis-regulatory mechanism. The expression levels of XR_003305156.1 in EDB and LDB were higher than in NDB and QB. It has been suggested that lncRNAs can interact with different biological molecules, such as RNA, DNA and protein, to exert influence on biological processes [28]. Therefore, the more precise relationship between waggle dance and these two DElncRNAs as well as their target genes needs to be examined in the future.

Additionally, one lncRNA that may be related to foraging behaviour was detected as well. The DElncRNA *kakusei* (NR_003565.1) was shared between QB-vs-EDB, QB-vs-LDB and QB-vs-NDB, which has higher expression levels in EDB, LDB and NDB than in QB. Compared with the quiescent bees (QB), foraging activity was experienced by the other three groups of bees (EDB, LDB and NDB). Previous studies have shown that *kakusei*, an immediate early gene, can function as a marker for the monitoring of neural activity in honey bee brains [38,39]. In this way, the results indicated that the neural activities of the small-type Kenyon cells significantly increased in the brains of dancers and foragers [58]. The Kenyon cells are important components of MBs, which are the centre of sensory integration [59]. During the foraging process of honey bees, visual information is continuously updated and integrated into the MBs via the optic lobes [60]. Using *kakusei* as a marker of neuron activity in the brains of honey bees, the results indicated that GABAergic neuron activity in the optic lobes of the foragers predominantly increased, suggesting that GABAergic neurons may mediate the processing of visual information in the optic lobes [61]. Similarly, another study revealed the relationship between dance type and neural activities of the MBs by using *kakusei*, but the results indicated that the neural activities of the MBs of round dancers and waggle dancers in the field experiments and the tunnel experiments were different, showing the complicated feature of *kakusei* under different conditions [62]. These results were identical to our sequence results, suggesting that *kakusei* may be closely involved in foraging behaviour, which is crucial for dance behaviour. As a lncRNA, *kakusei* is probably involved in the modulation of neural function by regulating the expression of specific genes. In this study, it was found that *kakusei* targeted two genes, SCM-1 (ncbi_408663) and wupA (ncbi_410815), via the cis-regulatory mechanism, as well as two genes, Hspa8 (ncbi_410620) and gpr89 (ncbi_551156), via the trans-regulatory mechanism. Among them, it was described that Hspa8 was related to heat shock protein Hsp70 in *Apis mellifera* that was implicated in foraging activity [63]. Accordingly, further research is required to detect the function of *kakusei* in dance behaviour.

In terms of GO enrichment analysis, it was found that the target genes of DElncRNAs from the comparisons of non-dancing bees (NDB) and waggling dancers (EDB and LDB) were mainly annotated in biological processes related to metabolic process, signalling and response to stimulus and in molecular function associated with signal transducer activity, molecular transducer activity and binding. The top 20 terms of GO enrichment were mainly involved in signalling and response to different stimuli, such as response to the hormone, chemical and endogenous stimulus. Further analysis found that the above results were alike to the analysis results from the comparison of quiescent bees (QB) and non-dancing bees (NDB). However, in the comparison of quiescent bees (QB) and waggling dancers (EDB and LDB), the top 20 terms of GO enrichment were mainly associated with homeostasis. Compared with non-dancing bees (NDB), waggling dancers (EDB and LDB) displayed a symbolic dance communication, which is an intense and energy-consuming process. When dancing on the comb, dancers are confronted with stimuli from the hive, such as feedback from followers; at the same time, dancers also respond to stimuli in vivo, such as the changes of neuronal activities [64]. Thus, signal transduction is extremely important during dance communication. Biogenic aminergic neurons are involved in the dance behaviour of honey bees. For instance, octopaminergic neurons mediate the representation of food value during the dancing process [23]. As compared to quiescent bees (QB) that kept a relatively steady state on the comb, non-dancing bees (NDB) had gone through foraging activity, in which they needed to respond to a variety of internal and external stimuli and consume amounts of energy. Similarly, signal transduction is required for the foraging process. For example, the activities of GABAergic neurons significantly increased in the optic lobes of the foragers that were responsible for the transmission of visual information [61]. While waggling dancers (EDB and LDB) can coordinate changes in their bodies to achieve a relatively stable internal environment, i.e., homeostasis, after flying back to the hive. Therefore, neural activity associated with signal transmission plays an important role in the food collection process, including the foraging and dancing process.

Furthermore, KEGG pathway enrichment analysis showed that nitrogen metabolism appeared to be involved in the food collection process that included the foraging activity and the waggle dance. Two DElncRNAs, XR_003305473.1 and XR_003304726.1, targeted two genes, ncbi-725877 (bca-1) and ncbi-408827 (ca2), respectively, which were predicted to be related to carbonic anhydrase and enriched in nitrogen metabolism, in the manner of cis-regulatory mechanism. It was reported that carbonic anhydrase can function as ion transport and pH regulation [65]. Carbonic anhydrase activity has been observed in the brain glial cells of honey bee drones [66]. These results indicated that carbonic anhydrase presumably serves as a signal regulator. In the nitrogen metabolic pathway, nitrogen can produce ammonia and then synthesise L-glutamine, which is further catalysed to produce L-glutamate. Glutamate is an excitatory neurotransmitter in the nervous system, which is closely associated with olfactory learning and memory in honey bees [67]. It was reported that glutamate participated in the neuron communication in insects [68]. Biogenic amines are a class of neuroactive molecules containing nitrogen, which are the metabolic derivatives of amino acids [69]. For instance, glutamic acid can be catalysed by glutamic acid decarboxylase to synthesise GABA, which is implicated in signal transduction in the antennal lobes and optic lobes [70,71]. In vertebrates and invertebrates, biogenic amines serve as neurotransmitters, neuromodulators and/or neurohormones, which mediate a range of physiological and behavioural responses [72]. The honey bee waggle dance is an extremely sophisticated behaviour, which needs multiple sensory coordination mediated by neurotransmitters. We thus proposed that neurotransmitters requiring the participation of nitrogen metabolism are associated with the waggle dance in honey bees. As a result, the detection of neurotransmitters such as glutamate, GABA and octopamine in the brain using high-performance liquid chromatography (HPLC) is worthy.

A portion of genes and microRNAs associated with the dance behaviour of honey bees were found by genomics and transcriptomics analysis. Some neurotransmitters related to dance behaviour were also detected by HPLC. Competitive endogenous RNA (ceRNA) hypothesis proposed that mRNAs, lncRNAs, pseudogenes and circRNAs will competitively bind microRNAs through microRNA response elements (MREs), disclosing a new mechanism of RNA interaction [73]. In the future, using ceRNA analysis will contribute to the decoding of the molecular mechanism underlying the dance behaviour from the view of non-coding RNAs. In addition, the precise function of lncRNAs in the honey bee waggle dance described above can be examined by means of gene manipulation [74,75,76,77].

## 5. Conclusions

In the present work, the waggling dance in the honey bee (*Apis mellifera*) was successfully regulated by varying the concentration of sucrose solution. The lncRNAs in the brains of waggling dancers and non-dancing bees were detected by RNA sequencing for the first time. Then, the waggle dance was interpreted from the insight of lncRNA−mRNA association analysis. Signal transmission mediated by neurotransmitters is crucial to the waggle dance. Our study provided a new horizon for understanding the waggle dance of honey bees. These findings indicated that food stimuli can induce responses, including visible behavioural performance and invisible changes in gene expression. The occurrence of a specific behaviour was accompanied by nervous activities, such as fluctuations in neurotransmitters, which were synchronised with changes in gene expression in the brain. Therefore, gene expression associated with neurotransmitters in the brain can be characterised by RNA sequencing, which is helpful in obtaining more comprehensive knowledge of the relationship between brain activity and behaviour.

## Figures and Tables

**Table 1:** The number of DElncRNAs identified by intergroup comparisons.

DElncRNA	NDB-vs-EDB	NDB-vs-LDB	QB-vs-EDB	QB-vs-LDB	QB-vs-NDB
Up-regulated	19	14	19	6	16
Down-regulated	18	19	31	23	29
Total	37	33	50	29	45

Note: EDB referred to the waggling dancers after foraging on a feeder with 1.5 M sucrose solution in the first phase. NDB referred to the non-dancing bees after foraging on another feeder with 0.5 M sucrose solution replacing the feeder with 1.5 M sucrose solution in the second phase. LDB referred to the waggling dancers after foraging on the feeder with 1.5 M sucrose solution replacing the feeder with 0.5 M sucrose solution in the third phase. QB referred to quiescent bees on the comb after removing the feeder for 1 h in the fourth phase.

## Data Availability

The data that support the findings of this study are available from the corresponding author upon reasonable request.

## Supplementary Materials

The following are available online at https://www.mdpi.com/article/10.3390/insects13020111/s1. Figure S1: The novel long non-coding RNAs identified by the intersection of CNCI and CPC2. Figure S2: The correlation analysis of 12 samples from four groups of honey bee brains. Figure S3: Top 20 of GO terms enrichment analysis of target genes regulated by DElncRNAs via the antisense regulatory mechanism in NDB-vs-EDB (a) and NDB-vs-LDB (b). Figure S4: Top 20 of KEGG pathway enrichment analysis of target genes regulated by DElncRNAs via the antisense regulatory mechanism in NDB-vs-EDB. Figure S5: GO enrichment analysis of target genes regulated by DElncRNAs from the intersection between NDB-vs-EDB and NDB-vs-LDB via the cis-regulatory mechanism. Figure S6: GO enrichment analysis of target genes regulated by DElncRNAs from the intersection between QB-vs-EDB and QB-vs-LDB via the cis-regulatory mechanism. Figure S7: GO enrichment analysis of target genes regulated by DElncRNAs in QB-vs-NDB via the cis-regulatory mechanism. Table S1: The specific primers for qPCR. Table S2: Quality control and evaluation of RNA sequencing results. Table S3: LncRNA sequencing results from four groups of honey bee brains. Table S4. The sequence information of 432 novel lncRNAs found in the study. Table S5: GO enrichment analysis of target genes regulated by DElncRNAs in NDB-vs-EDB, NDB-vs-LDB, QB-vs-EDB, QB-vs-LDB via the cis-regulatory mechanism. Table S6: KEGG enrichment analysis of target genes regulated by DElncRNAs in NDB-vs-EDB, NDB-vs-LDB, QB-vs-EDB, QB-vs-LDB via the cis-regulatory mechanism. Table S7: Information of target genes regulated by DElncRNAs from the intersection of NDB-vs-EDB and NDB-vs-LDB via the cis-regulatory mechanism. Table S8: Information of target genes regulated by DElncRNAs from the intersection of QB-vs-EDB and QB-vs-LDB via the cis-regulatory mechanism. Table S9: Top 20 of GO terms enrichment analysis of target genes regulated by DElncRNAs from the intersection between QB-vs-EDB and QB-vs-LDB via the cis-regulatory mechanism. Table S10: Top 20 of KEGG pathway enrichment analysis of target genes regulated by DElncRNAs from the intersection between QB-vs-EDB and QB-vs-LDB via the cis-regulatory mechanism. Table S11: Top 20 of GO terms enrichment analysis of target genes regulated by DElncRNAs in QB-vs-NDB via the cis-regulatory mechanism. Table S12: Top 20 of KEGG pathway enrichment analysis of target genes regulated by DElncRNAs in QB-vs-NDB via the cis-regulatory mechanism.
