# Supplementary material for: Understanding of Waggle Dance in the Honey Bee (Apis mellifera) from the Perspective of Long Non-Coding RNA
Source: Insects. 2022 Jan 19;13(2):111. doi: 10.3390/insects13020111 (PMC8878125; doi:10.3390/insects13020111)
Supplement: Supplementary file 1 [file insects-13-00111-s001.zip › insects-1477378-supplementary/Insects_Supplementary Materials/insects-1477378-supplementary files(except table S5 6).pdf]

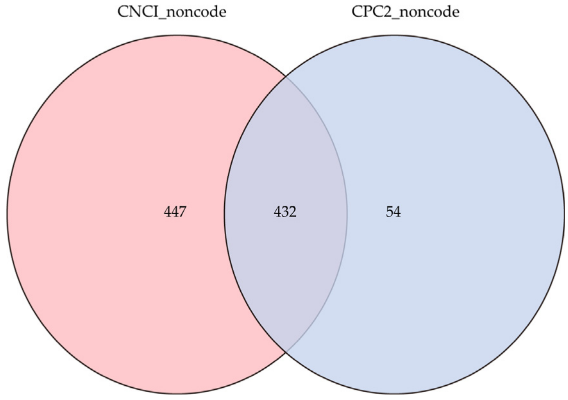

Figure S1. The novel long non-coding RNAs identified by the intersection of CNCI and CPC2. To ensure the reliability of predictive results, 432 novel long non-coding RNAs are defined by the intersection of CNCI (879) and CPC2 (486), which are used to examine the coding potential of transcripts.

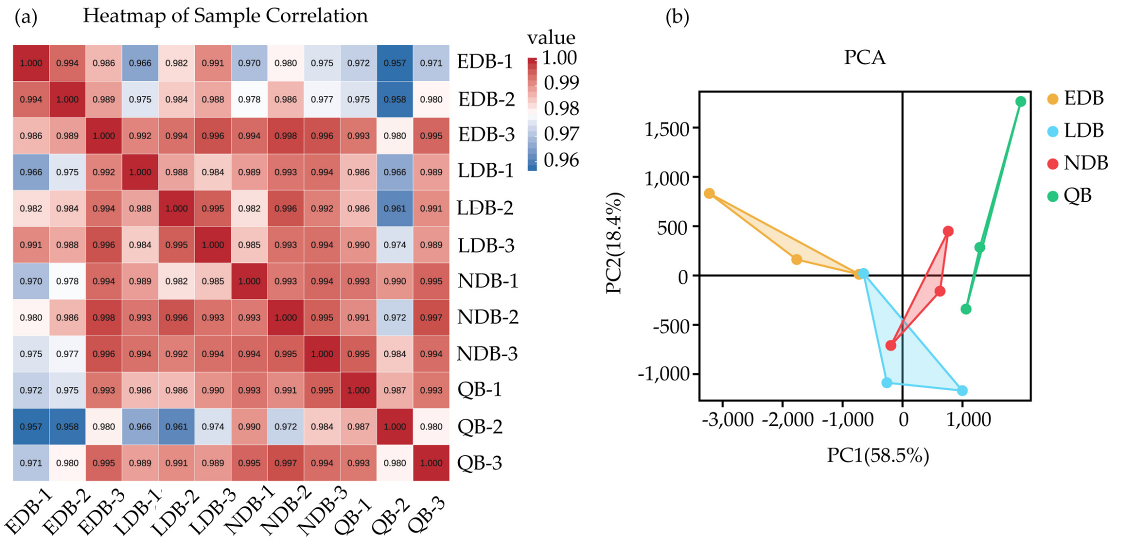

Figure S2. The correlation analysis of 12 samples from four groups of honey bee brains. (**a**) Heatmap of sample correlation. According to the expression amount of the whole lncRNA set in any two samples, the Pearson correlation coefficient between every two samples was calculated, and then the correlation between any two samples was intuitively displayed in the form of the heatmap. (**b**) Principal component analysis (PCA). The more similar the sample composition is, the closer the distance is reflected in the PCA diagram, and the samples from different treatments tend to show their aggregation distribution. Note: EDB referred to the waggling dancers after foraging on a feeder with 1.5 M sucrose solution in the first phase. NDB referred to the non-dancing bees after foraging on another feeder with 0.5 M sucrose solution replacing the feeder with 1.5 M sucrose solution in the second phase. LDB referred to the waggling dancers after foraging on the feeder with 1.5 M sucrose solution replacing the feeder with 0.5 M sucrose solution in the third phase. QB referred to quiescent bees on the comb after removing the feeder for 1 hour in the fourth phase.

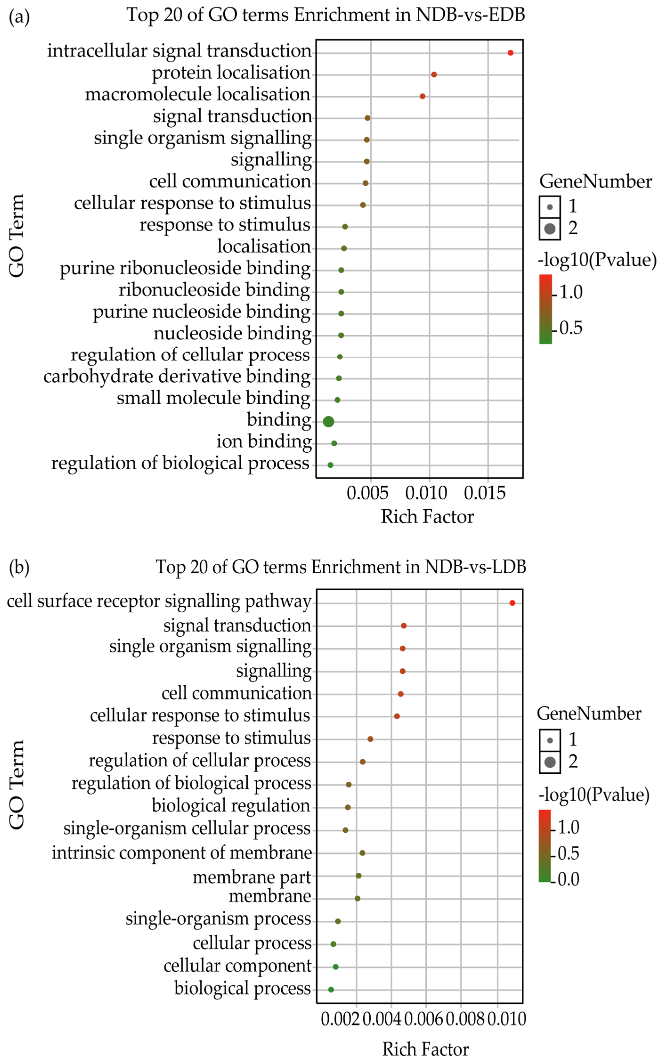

Figure S3. Top 20 of GO terms enrichment analysis of target genes regulated by DElncRNAs via the antisense regulatory mechanism in NDB-vs-EDB (**a**) and NDB-vs-LDB (**b**). Rich Factor refers to the ratio of the number of genes located at this term in the differentially expressed genes to the total number of genes located at this term in all genes. The larger Rich Factor is, the higher the enrichment degree is. The bubble size represents the number of genes, and the larger the bubble is, the more genes are enriched in this term. The depth of bubble color represents the level of significance, and the redder the color, the higher the significance of enrichment into this term. Note: EDB referred to the waggling dancers after foraging on a feeder with 1.5 M sucrose solution in the first phase. NDB referred to the non-dancing bees after foraging on another feeder with 0.5 M sucrose solution replacing the feeder with 1.5 M sucrose solution in the second phase. LDB referred to the waggling dancers after foraging on the feeder with 1.5 M sucrose solution replacing the feeder with 0.5 M sucrose solution in the third phase.

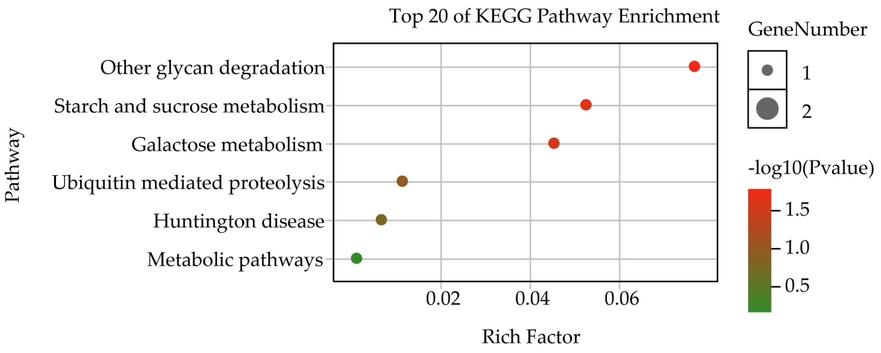

Figure S4. Top 20 of KEGG pathway enrichment analysis of target genes regulated by DElncRNAs via the antisense regulatory mechanism in NDB-vs-EDB. Rich Factor refers to the ratio of the number of genes located at this pathway in differentially expressed genes to the total number of genes located at this pathway in all genes. The larger Rich Factor is, the higher the enrichment degree is. The bubble size indicates the number of genes, and the larger the bubble is, the more genes are enriched in this pathway. The depth of bubble color indicated the level of significance, and the redder color, the higher the significance of enrichment into this pathway. Note: EDB referred to the waggling dancers after foraging on a feeder with 1.5 M sucrose solution in the first phase. NDB referred to the non-dancing bees after foraging on another feeder with 0.5 M sucrose solution replacing the feeder with 1.5 M sucrose solution in the second phase.

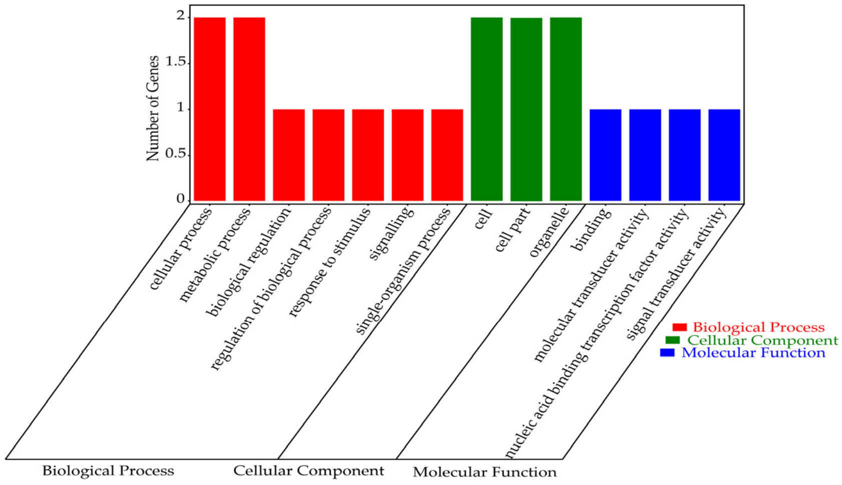

Figure S5. GO enrichment analysis of target genes regulated by DElncRNAs from the intersection between NDB-vs-EDB and NDB-vs-LDB via the cis-regulatory mechanism. Note: EDB referred to the waggling dancers after foraging on a feeder with 1.5 M sucrose solution in the first phase. NDB referred to the non-dancing bees after foraging on another feeder with 0.5 M sucrose solution replacing the feeder with 1.5 M sucrose solution in the second phase. LDB referred to the waggling dancers after foraging on the feeder with 1.5 M sucrose solution replacing the feeder with 0.5 M sucrose solution in the third phase.

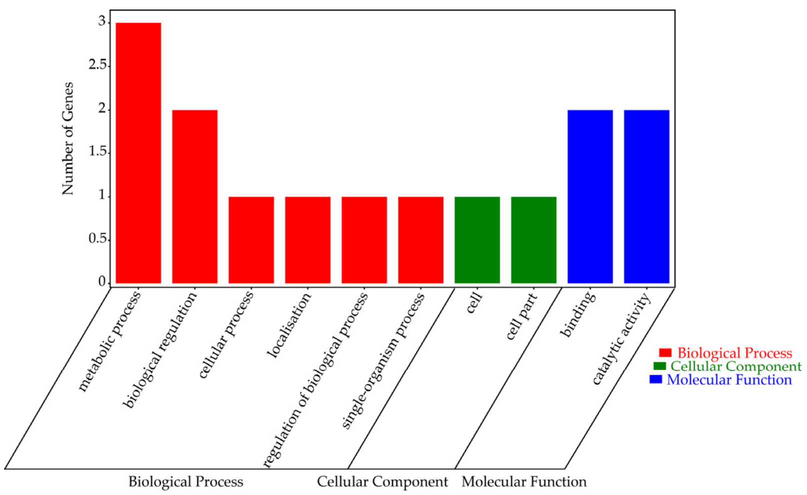

Figure S6. GO enrichment analysis of target genes regulated by DElncRNAs from the intersection between QB-vs-EDB and QB-vs-LDB via the cis-regulatory mechanism. Note: EDB referred to the waggling dancers after foraging on a feeder with 1.5 M sucrose solution in the first phase. LDB referred to the waggling dancers after foraging on the feeder with 1.5 M sucrose solution replacing the feeder with 0.5 M sucrose solution in the third phase. QB referred to quiescent bees on the comb after removing the feeder for 1 hour in the fourth phase.

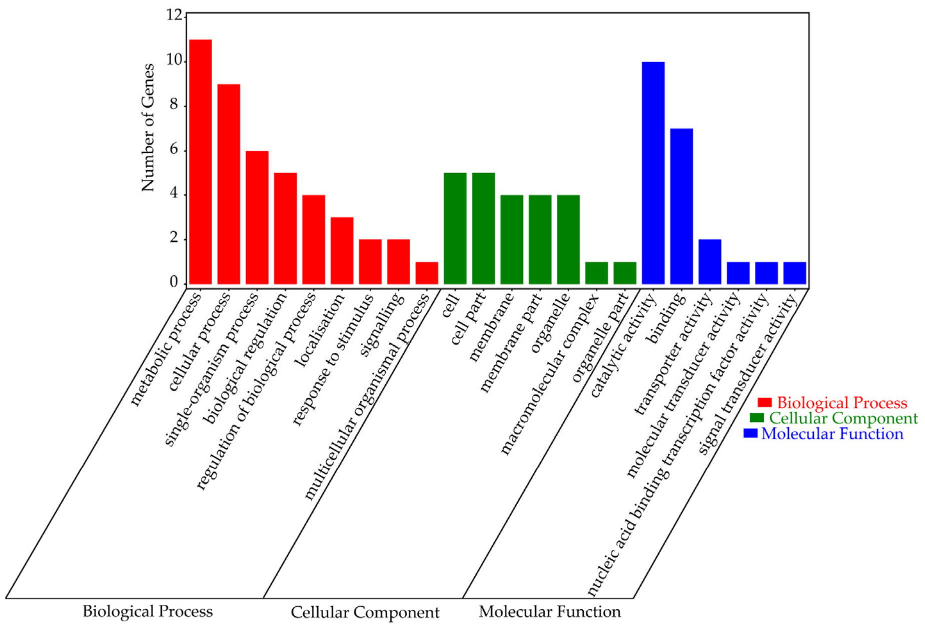

Figure S7. GO enrichment analysis of target genes regulated by DElncRNAs in QB-vs-NDB via the cis-regulatory mechanism. Note: NDB referred to the non-dancing bees after foraging on another feeder with 0.5 M sucrose solution replacing the feeder with 1.5 M sucrose solution in the second phase. QB referred to quiescent bees on the comb after removing the feeder for 1 hour in the fourth phase.

Table S1. The specific primers for qPCR.

Primers	Sequnces (5'→3')
MSTRG.3703.1-F	GCAAGAATAAGAGCATTGGCGGAAG
MSTRG.3703.1-R	TCAACGGCAGGAAGCGAGTCT
XR_003305349.1-F	CCAGAGCATCATTCACCTCCAGTTC
XR_003305349.1-R	AACAGGGCGAAGAGAGCAACAAG
XR_001702399.2-F	ACTGGAGTTGGAGGCGTCTGAAT
XR_001702399.2-R	CAGCAAGCACCGCTAGACCTTC
XR_003304525.1-F	ATATCGGCTCAAGGTGCAAGGTG
XR_003304525.1-R	CGGATTCGCTCGTTCCTCTTCTC
XR_003304191.1-F	TGTTGATGAGGAGCACTCGTTAGTT
XR_003304191.1-R	CGTGGATGAGGCACATTCGTAGAA
MSTRG.8314.1-F	TCTTACCCAGTGTTAGGCTCGCTAA
MSTRG.8314.1-R	ACGTGATGCAAATGAACCAGAACCA
XR_001706524.2-F	AGACGAATGGATTGCGAAGTTGGA
XR_001706524.2-R	CGACCTCTAACTACGAGTCTGCTCT
MSTRG.3555.3-F	CAAGGCTGTATCTTGCTCGGTGATT
MSTRG.3555.3-R	CCGTCCGTTCCAATGCCATCTC
actin-F	GAAATGGCAACTGCTGCATC
actin-R	TCCACATCTGTTGGAAGGTG

Table S2. Quality control and evaluation of RNA sequencing results.

Sample	Raw Reads	Clean Reads(%)	Q20(%)	Q30(%)	Mapped Reads(%)	Unmapped Reads(%)
EDB-1	106618148	106440372 (99.83%)	98.59	95.78	20875902 (19.61%)	85564470 (80.39%)
EDB-2	87365452	87221972 (99.84%)	98.61	95.81	16065688 (18.42%)	71156284 (81.58%)
EDB-3	83544802	83396150 (99.82%)	98.50	95.60	16214902 (19.44%)	67181248 (80.56%)
LDB-1	85998742	85888630 (99.87%)	97.02	91.33	13817784 (16.09%)	72070846 (83.91%)
LDB-2	85111522	85006610 (99.88%)	97.09	91.45	13131116 (15.45%)	71875494 (84.55%)
LDB-3	81773108	81676726 (99.88%)	97.13	91.57	13845216 (16.95%)	67831510 (83.05%)
NDB-1	90789674	90642128 (99.84%)	97.10	91.51	16532822 (18.24%)	74109306 (81.76%)
NDB-2	79308232	79195420 (99.86%)	96.97	91.22	13109598 (16.55%)	66085822 (83.45%)
NDB-3	79929110	79828088 (99.87%)	97.02	91.25	14234548 (17.83%)	65593540 (82.17%)
QB-1	87062400	86956212 (99.88%)	97.05	91.39	14830104 (17.05%)	72126108 (82.95%)
QB-2	83274924	83174990 (99.88%)	97.07	91.44	16591666 (19.95%)	66583324 (80.05%)
QB-3	83322858	83219542 (99.88%)	96.96	91.21	14409218 (17.31%)	68810324 (82.69%)

Note: EDB referred to the waggling dancers after foraging on a feeder with 1.5 M sucrose solution in the first phase. NDB referred to the non-dancing bees after foraging on another feeder with 0.5 M sucrose solution replacing the feeder with 1.5 M sucrose solution in the second phase. LDB referred to the waggling dancers after foraging on the feeder with 1.5 M sucrose solution replacing the feeder with 0.5 M sucrose solution in the third phase. QB referred to quiescent bees on the comb after removing the feeder for 1 hour in the fourth phase.

Table S3. LncRNA sequencing results from four groups of honey bee brains.

sample	Reference lncRNAs	Sequenced Reference lncRNAs(%)	Novel lncRNAs	Sequenced Novel lncRNAs(%)	Total lncRNAs	Sequenced Total lncRNAs(%)
**all**	**3146**	**2445 (77.72%)**	**432**	**432 (100.00%)**	**3578**	**2877 (80.41%)**
EDB-1	3146	1310 (41.64%)	432	392 (90.74%)	3578	1702 (47.57%)
EDB-2	3146	1268 (40.31%)	432	395 (91.44%)	3578	1663 (46.48%)
EDB-3	3146	1333 (42.37%)	432	402 (93.06%)	3578	1735 (48.49%)
LDB-1	3146	1369 (43.52%)	432	402 (93.06%)	3578	1771 (49.50%)
LDB-2	3146	1332 (42.34%)	432	395 (91.44%)	3578	1727 (48.27%)
LDB-3	3146	1344 (42.72%)	432	402 (93.06%)	3578	1746 (48.80%)
NDB-1	3146	1341 (42.63%)	432	399 (92.36%)	3578	1740 (48.63%)
NDB-2	3146	1253 (39.83%)	432	399 (92.36%)	3578	1652 (46.17%)
NDB-3	3146	1359 (43.20%)	432	400 (92.59%)	3578	1759 (49.16%)
QB-1	3146	1436 (45.65%)	432	403 (93.29%)	3578	1839 (51.40%)
QB-2	3146	1413 (44.91%)	432	403 (93.29%)	3578	1816 (50.75%)
QB-3	3146	1446 (45.96%)	432	400 (92.59%)	3578	1846 (51.59%)

Note: EDB referred to the waggling dancers after foraging on a feeder with 1.5 M sucrose solution in the first phase. NDB referred to the non-dancing bees after foraging on another feeder with 0.5 M sucrose solution replacing the feeder with 1.5 M sucrose solution in the second phase. LDB referred to the waggling dancers after foraging on the feeder with 1.5 M sucrose solution replacing the feeder with 0.5 M sucrose solution in the third phase. QB referred to quiescent bees on the comb after removing the feeder for 1 hour in the fourth phase.

Table S4. The sequence information of 432 novel lncRNAs found in the study

1. >MSTRG.3.1 Gene=MSTRG.3

CTACAAAATGTCAATATCAAATTGCTAATTCAAAATTTATATTATGTATATTAGAAAAATGA
ATATTTATTATTCGATATAAAGAAATTAATAAAAAGATTGATCCAATTAATACATGTAAAC
CATGAAATCCTGTTGCTATAAAAAAAATTGAACCATAAATACTATCATTAAAACAAAAAA
ATGAATTTGAGTATTCAATTGTTTGAAGAATTGTAAAATAAATTCCTAATAGAATTGTTAAT
AATAAATATGATTTTCTAAGTTTTAAATTATTAATAATTAAATAATAATGACTTAATGTAAC
TGTAAATCCTGATGATACAAGAATAAATGAATTTAGTAAAGGAATTTCTATTGGATTAAAA
AATTTAATATTTTTTGGTGGTCATGTTATATTAATTTCAATATTTGGAGAAATTGATGAATGA
AAAAATGTTCAAAAAAATGAAATAAAAAATATTAATTCTGATAAAATAAATAAAATTATT
CTAAATTTTAAAAAATTAGTAATAAATATTGAATGTATACCTTGAAATGTTCTTTCTCGAAT
AATATCTCGAAATCATAATATTATAATTAAAATTGAATTAATAAAATTTAAAATTATAAATA
TTGAGATTGATCTATAAATTCAAATAACTGTTCTAATGAGAGTATTTATAAATCTAAATGAT
AAAATAATTGGTCAAGGTCTATTTGTAACTATATGAAATGGGAAATTTTTTTTCATTTAGTTT
TATTTTTAATATTTTAATTTGATTCAGAGAAATATAAAATTAAAAGAATTGAAAATACATAA
CTTTGAATTATTGATATAAAAATTTCTAAAGTTAAAAGTATATTTTGAATTATTAAATTAATT
GGTAAAATTGAAATAAAGTTTCTAATAAAAATTCCTAATAATGTTAAAATTAAATGTCCAG
AAATTAAATTTGCTGATAATCGAATTGATAATGTTCAAGGTCGAATAATTAATCTAATAAGT
TCAATAATTACTATAAAGTTTATTAAAAATACTGGTGAATTTAATGGAACTAAATGACTAA
GGAATATAATATAATTATTATAAATTAAATAAATTAGAAATCTAAATCATAATGTTAATGAT
AAAATTATATTTAAAAGTAGATGTCTTGTTAATGTAAAAACATAAGGAATTAATCTAAAAA
TGTTAGTAATTATAATATAAAGTATTAATCTAATAAAAATAATAATATTAGATTGATATTTA
GATTTGTTTATTTACTCAATTTAAAAAATATTGAAATGAAGTTGATTCAATTATAATTGGTAT
AAATCTATGATTTATACCACAAATTTCTGAACATTGACCAAAAAAAATTCCTGGACGTTTTC
TAATTAAATTTAATTGATTAATTCGTCCTGGAACTGCATCAACTTTAATACCTAAGGATGGA
ACTGTTCATGAATGAATTACATCTGTTGATGTTGTAATTAAACGTAGTGGGATTTTTATTGGA
ATTACTATTCGATTATCAGTTTCTAGTAAACGAAATTGGTTTAAATTATTATAATTTAGTATA
TATGAATCAAATTCAATATTATTAAATTCTGGATATTCATATGATCAATATCATTGATGACC
AATTGATTTAATTGAAAAAAAAGGATTTACAATTTCATCAATTAAATATAAAATTTTTAATG
ATGGAAAACAAATAATTAATAGAATAATAATTGGAATAATTGTTCAAATAATTTCAATATT
ATGATTTTTTAATAAAAATAAATTTGAGAATTTGTTTATAAATAAATCTAAAATAATATATA
CAGTTAATGTTGAAATTATAATAATAATTATTATAACTATATTATGAAATGAAATTAAATTA
TCAGCATAATATGAATTTGATTCTTGAAATATAAATATAAATCATGTGGAAATTTTAATAAA
TTAAATTTTAATAAATATATTCTGTTATATTTTGTTTTATTTTATATTAATTTATTAATTTTAAT
AAAAAGATTAAATCTTTAAGATTGAATTTAAAATTCAATATAAAAATAAAATTAATTATTT
ATTTTATTAAAAATTGTTATTAATTTATTTTTAAATTAATATGAATTAAGTGGGGAAATTTTA
ATAAAAGTTTAAAAATACTTTATATTTGAATCTTAAGTTCAATGCACTTATTCTGCCATATTA
AAATTTAATTAAAATTGATTTTAAATTTAAATTTTTAATTAATAATGGAATTTCTAAATGTGA
ATGATCTAGAGGTGGTAAAAAATTTAATCATTCAAGTGATGATTGGTTGAATTTAAATAATA
ATATTCGTTTAGAAATTAATCTTTCTAAAATAATAAAAATTAAAAAAATTATTCTATTTAAT
GAAATTATTGATCCTATAGATGAAATTGAATTTCAACAGTAATAAGAATCTGGATAGTCTG
AATAACGTCGTGGTATAGATATTAGTCCTAAAAAATGTTGAGGAAAGAAAGTTAGATTTAC

TCCAATAAATATTATAATAAATTGAATTTTTAATCATTTAATATTTAATAATAATCCAGTAAT
TAATGGATATCAATGAATAAATCTTGAAATAATTGCAAATACTGCACCTATTGAAAGAACA
TAATGAAAATGTCCAACAACGTAATATGTATCATGAAGAATAATATCAATAGAAGAATTTG
ATAATATAATTCCTGTTAATCCACCAATAGTAAATAGTATAATAAAACCTAGTGATCATAA
AATTGAAATATTTAATTTTAATTTTGAACCATGATAAGTTGCTAATCATCTAAAAACTTTAA
TTCCTGTTGGTAC
AGCAATGATTATTGTTGCTGAAGTAAAATATGCTCGAGTATCAACATCTAATCCGACTGTA
AATATGTGATGTGCTCAAACAATAAAACCTAGAAATCCAATTCCTAATATTGCATAAATTA
TTCTTAAATTACCAAAAATTTCTTTTTTTCCTCTTTCATTTATTACAATATGAGAGATTAATCC
AAATCCAGGTAAAATTAAAATATAAACTTCTGGATGACCAAAAAATCAAAATAAATGTTG
ATAAAGAATTGGATCTCCACCTCCTATAGGATCGAAAAATGATGTATTAAAATTTCGATCA
AATAATAGTATAGTAATTGCTCCAGCTAATACAGGTAATGATATAATTAATAAAATTGCTGT
AATAAAAACTGATCATGGAAATAATGAAATTTGGTCATAATTTATAGAAAAATTTTTTATTA
TTATAATTGTAACTATTAAGTTTAATGATCCTATAATTGAGGAAATTCCTGATATATGAAGA
GAAAAAATTGCAAAATCTACTGAAGGTGAAGAATGATATAAATATGCTGATAATGGTGGA
TATACTGTTCATCCAGTTCCTGGTCTTGGATAAAATAAATTTCTTAATAAAAGTATAAATAA
TGAGGGAGGAAGTAATCAAAATCTAATATTATTTATTCGGGGGAATGCTATATCAGGTGAT
CCTAGTATTAAAGGAATAAGCCAATTTCCAAATCCTCCAATTAAAAATGGTATAACTATAA
AAAAAATTATTAGGAATGCATGACTAGTAACAATTGTATTATAAATTTGATCATTGCTAATT
CATGATCCTGGGGATCTTAATTCTATTCGAATAATAAGTCTCATTGATGATCCTAGTATTCC
AGATCATAAAGCTAGAATAATATACAAGATCCCAATATTTTTATGATTGGTTGATATGAATC
ACTTTATTATTAAGATTTAAAAAATTAATTTTTATTTTTAATTTTGAAGATTAATAGTTTTTAA
ATTTTAACTTAAAATCTTTGTATATATTTTAAAAATTTAAAAAAATAAAAAAATAAAAAAG
GGTTTTTTAAAT

2. >MSTRG.31.17 Gene=MSTRG.31
TACAACATGGCGGGTGAGTGAGTGCCTGTGGATCGAACGAAGCTGCCCAAAAACACGAGG
GATTTTCAGCTAACCCGTGGTGTTTGTGGGTTTCAGATATATCCTACATCCAGAAAGAAGAA
CTGGACGATTCTGACGAAGTGACAACGGCTGGAACGACACGTGGATACGCGAAGAAATCT
TGGGGTTACTTTGTGCGATTAAAAAGCAATGGAGATTGATGAAGTCGACGACGATGATGTT
GATTATAATCTGAGTCCCGACGCTTCCGGAAACGATATTTGCAATATTGGAGCAAGTGGCG
ACGAAGGTGGAAAGGACGAACTGCTACTGAAATTGCAACAGTACGTAACCTCGGACGCAA
ACTTCACACGGTAAGTCGAATCGAGCTACTATCCATCCACTGTTGCGAGCGATCGTTTCCGG
TAAGTGTGTTTCCCTTACCGATCATCATTCGCCTCGTTCGATCCGAACGCGTTTTTT

3. >MSTRG.52.2 Gene=MSTRG.52
TCTGGATGTTCCGCGCGCAATGTAGTAAAAGGTCGTTCAGCAAGTCGTCAATTTAAATTCAT
TTGTTGTAACACGTGTCAGTGAATAGTTGTATACTTATTGAGTATTGTCATTATTTATGTAAA
TATTTTTAAACTAATATTTTAATAATAAAAAATGTTACGAGAATCAGTTGAAGAAATAGCC
GGAAAGCCTGGTTTGTACTTTTCATTTTTTGGGTTTCTATGCGCACGCGGTTCACTTCGCTGA
ATATAACGGTCGATTCAGCATTGAATATCATATTCTTATCAATTTTATAACGTGTTTTTATAT
AAATTTTTAATTTAGAACTTCTTTGAATATATAATTAACATTTTTTTAAAAATTTGACTGTAT
CATTTTATTTCTTTCTATTTTTTTTTTTAAATTTTTAGATGGCAAAAATCGTATCCACACTGAG

ACAATATCAGTTGATTCACCACTTCGTAGAAGTTCGCGAATTAAGCAAAACCTTAAACAAA
ATGAATTTTCACCTGAATCGTCTCTAAGCGACGCTAATAATATACAAACTTCAAGGAGTCC
AAGACGACGTTTAATTACCATGGACAATACGGTTTCTATTGAAAATCAAAGAAATTTACGT
TCTAGGACAAATTCTGTATCTTCAGATATTAGCGAAGTCCTAGAAACAGGTATTGGTACAC
CAACAAGAAAAACTAAAAATAATACTTCTAATGATAATAAAATGATAGGTAGTCGAAGAA
GTAAACGCCTTGTAAGAGCTGGATCAGAAGCGAATTCACCACCATCAGCAACAAGAAACA
CACGTAATACAAGAGCTAGTTCTATGGGACCTGAAGCTACATCTGATAAGCTAACAGAAA
AACGAAACAATGAATTAATAAGTACATCTATGAGAACAAGAAGACGATCTTCCTTAATATT
TTCAGAAGTAACTACAGAAGAAAAAGGAATATCTATGAAAATTCCTAGGGTAACATTAAA
TCGTACATTACCTGATATTAATGAAAGTACAC

4. >MSTRG.99.1 Gene=MSTRG.99
CTAATACGATCTTCCCGTATATACGTGGCAACGACAAAGGTCTGGAAAAACGCGAGTTCGT
GATAGCCCGGAGGGCAGATCGTGGCCAGTAAGATGCACGTTTTCTCACCGCAAAACACAG
GAAGAAGAACTCGAACTGGAAACGAGGAATGGATTCTGCAACCGTTTCTGCGATTCTTCGA
TGAGATTGCTCGAAAAGGAAAATAAGCAAAGGCGAGAACTTGGCGAATTCTTGCTCGATC
GTTGGATTGGAAAAATTATGAATGTTCCGTGAATTCGGAAAAATAAAATTTTATTGACGAA
TTGAAATTTTGTGAAGAAATGAAATGAGGAATGTATGTTGATTTGGTAAATTTTTAAAAAGT
TTTCAAAGTTTGATGAGTATGTATTACGTTTGAATAATACGAATAATTACATAACCTC

5. >MSTRG.151.2 Gene=MSTRG.151
CTGGTATTGGACAGCATACTGGCCGTGGATAATAACAGGAGGCAGATACATTGTAAAACG
AACAAGGATACTTTCACGAAATATACACGCAGTCGTACATTGATTCAGGAATCGATCAGCG
ATACTATGTACAACACAACCACGGAAGCGTGCACGATGTTGAAGTAATTCGCAAATAATG
CGGTTGCACTTTTCTCAAATTTGGCACGCGGTGCATCTGCTCGTATATTATTTCCATTTTTATC
CCTTTTCCCTTATTCCAGTGTCTTTGTGTGCTCACGTAACAAATTAAACAATCTTAAATGCAC
TATAATCTCTTTCAGTGAGTCAAAACACTGGAATATGTCTCAACTGATGTCTGTTCTTGCTAT
TTTTTTTATAATAAATTTTTCAAAATAAACGTTATTTGATAAACTACCAAGAGGATAAACAG
ATTAGTTTTGCAACAGATCGAAAATGTTTAAGGTTAGAAACAGAAACCAGTCAAGATGATT
TCTATTTTAATTTAGATCCTTTAGATCCTTTAGATCCTAGCCAA

6. >MSTRG.152.3 Gene=MSTRG.152
GCTCAAAACATGTCGTGCATCGCGTTGTCGCGTCATGGAACAAGCTTAAATACGTTTCATTT
GCCTTTCTTGCGAGTGACAGTTAGTTTTTTTAAAAGTACAAATGCTTTTTATCAACTGATATC
ATTCAGTTGTAACAGAGCATTGATAAAATTTCAGAAAATTAAAAACGCCAGGTGACAGAA
TGCGATGCGATGTGGGAACGAAGTGTGGCGGCAGCACTGTACATTAAACGGAGCAGCGAA
GTAGAGGGAGGGAAAGTAAGAAAGATACACAATTGTAGAGTGAAAGAGACAGAGTAATG
AGAGTGAGATCAAACCATATTGCGAACGCCGCGGTGCGAGAAAGACCGAAATTTCGCTTC
AGCAGTACGGTAGTTGGCGTTTGTCCGTGGAAACAGGATAGTAGTCCATTGGAGGAAGAA
GGAGAAAGCATCGGAGAAGGAGGGCAGACTCGCGAGGGAGAGGCGACGAGTTTGTTTATC
GCCTTGGTGAACGGCGAGAGCAGTCAGTGCGGTGCTACACTTGGTCTGTAGTGGTCGGAAC
GATCGGTAGTTCGGTTCGAAAGTTCGGCCTCGTTCTTCTGACAATTTGTTCCGAATAGTGCG
TGGGTGCCGTCGTGTTTTTCTATCGCGCACATGTCAACGTCTATTTAACCTCGTCGTAGACCT

CGTGCCGGAAGAACATGCCGCGAACCACTGCCAAAACATAGTCGTTCTGTGTATGTATCGT
GTGCCAAAGTCGGTGACATTTGTGTCGTAGACGCAAGATCGCTGGAATTTCACTATACAAC
CTGGAAACCTGTAACACCGTGTGCTTACAACTTAAACAGACAAGATTCCCCTTCTCCAGCT
GATCGCAAAAAAAATCGTGAAATCGTTTCCGCGAGTGTCGTGTCGCGTATCGCGAGCGATC
TCGTCACCGTATCAGTCGTTTTATTTTCTTTTTCACTCCTCCCCCTTCGTGTCGTTCGGATGCG
TCGTAACGTTTACGATGGTATACGGAAATTAGATGAAGATTCGCGATTCCCGCCGGTTTTCA
CGGGAACATTAACCTTCGATTTCGTGTTTTCCCCGTCGTACCGTGTCTACGACGTTTGAAAA
TTCGTTTCGGTCGTGAATCTCGGGTTGAACGGGTGGAAAAATTACGCGTAACGATATTTTGG
AAGATAGTATCTCGTCCGTTTGGAAGTCGACGAGATGATCGTTCAAGCGGATTCGTTGTGCG
TGTTACTAGTGATGTTGATCGGTGCTCTGTTGGTAAAGTCGGAACCTGGGCCACGACCGCG
ATCAACGCCGATCTTCACCAATCAGTTCGCAGTCCACGTGCCAGATGGACCGGACGCTGCC
GCGGAGATCGCTGCCAAGTACGGATTCGACAATTATGGACAGGTAACGTGAGTCGTTCCGT
TTCTCGTGATGAAGATATGTAAATAAATATGTACGTAAGTAAATAGTAAATTTGAGTTAAA
TTTATCTCGTTTTTGTTTATTTACAAATCCCCTCCCTTCGATCTTTACCCGGATAAAATTTTTTT
TCCTTTCCACTTGTTCTTTGAACTTGTTGATCGTAATATTTAATTGATATACCGAGTTCCTTTC
CCGATTCTAAAAAATTAAATTCGTTGTCCAAAATGAAAATTTGTCTCGCGTTTCGATTTTATC
CCACGGCTTGATTACTCGAATCATATCACCGTATAAATTTAATGACAGTCTTTGTCGAGTGC
GGATCCCACCGCATTGTCACAATCACGTATGTTAATGAAACTTGGTAAATGATAGAGGTAT
ACGTATAAACAAACGTACCGACAGAGCGGATGGAGTGGGGGGGAGAGGAG

7. >MSTRG.176.3 Gene=MSTRG.176
CAATGCTTAATCCGGTTATAGATTATATAGGTCTTTTGCTTCCGGTAAACCATGGTAAGTGT
AGCGTAGTTTTAAATTTATTGATATTGCTCATAAAATCTATAGTATTATGAATTTATCATTTT
AAAAAAATTGTTTTTACATATTTTATAATTTAATTTATATTTATTTATTCTTGAAAATGCATA
AGAAATATAAAAATTATTCAATATAATCTTGCATTTTTCATAGTTTTCAACCACATGGTATG
TATTTTAAATTATTATAAATATATTAAATTAATAATTAAAAATTATTTATAATTTATGATATA
ATTTTTCATTATTATAAAAATAATTGTTTTTTTTTTGTTAAATAGCCATTTCAAAGGTTTGTAG
AATCTGGTCGTATTGCTTATGTGAGCGATGGGCCGCACAAAGGTAAACTTGTTGCCATTGTA
GACATCATTGATCAAAATCGGGTTTTAGTTGATGGACCTTCTTCAAATGTTCCACGTTGTGA
AATGAGATTAAATGATCTTCATTTAACTAAATTCCGTATACGTTTCCCATATACTGGATCCA
CCCGTGTTGTACGTAAAGCATGGGAAGCTGCAAATATTAATGACCTTTGGAAACAAACTAT
GTGGGCAAGAAAGGTTGAAGCTAAGAAAAAACGGCTGGAACTTAGTGATTTTGATCGTTTT
AAATTAAGAAAAGCAAGACAAATAAGAAACAAATTAAGGACAGATGCATTTTACAGATTA
AAAAAGAAAGCTAAGAAAACAAAGGCTACAGGTACCAGTAAAAAAGTTGAAAAGAAGTG
ATTTTTTATTCTATGAAATAA

8. >MSTRG.231.1 Gene=MSTRG.231
GCACATCCTTCGTTTTTTTCTTTGTTCTTTTCTCTCATAAAAAAAACCTGTTTTTTTTTCTATAA
CGAACTTTTCTCAACGATCTCTTAGTCCTTCTTGTCGCCGTCTTCGGGGTCTTTAAGCGTGTG
CCATTGAGCGATAGGACGTCTCGGGGATGCCAACATATCGGACCAGTGTCTCAACTCTGTT
CCACTCGCGTTGTACCCCAAGACGACCTTCCCAATGGGTTCTGATGTGCCGATACGATCGTA
ATCGACTACCGTGACAACCAATTGCACTTTCTGTATCTGTTCGAAGGGTACCTCAAACGTGA
ATGATTCGTTGTAATACGGATTAAGAGTGCATTTTTTGATAGACGTTTTCTTCTTCTTCAACC

TTTTGCCATTTTGCATCAGGGCGATTTTTACGTAAGGATCTGATAAACCACCGACGTCCATT
TTCTTCAGATTTTTCGCTTCCAAGATGACCACGGTTAATTTACCAGCAGTCGGTACGTATCG
AAGCGAGAAGCAAATGTCACCCAATTTGTTATCCTGACCACCTTCACCCTCGACGCTTTGC
AATTCTCTCCATTCCTCGATCGTCTGAGCCAGATCGACTTGGCATAACGGAACCTTGACTTC
ACCGATCTGATCGTGTTTGGAGAACCTATCGAAGTCGAAGATGGCGAAAACGAGAGTTTTG
TTCATCGCATCCGCATAAGGTACGCTCTTGAATGTGAAAGTCTCGTTGAACACTGGGCTAA
GCGTTTTCCTGTGCACCTTTGTTTCGAATTTTTTCTTCTTGTCTGGTAATAGATAAACCTTCAC
GTACGGATCTGAAGTACCACCCATGTCCAAAGCTGGCAATTCCTCGGCTTGTATTACCGTTA
CTGCTAAACTGTTTGTGTTGAAATCGTATTCGAGCTTGTATTGAAGTTTCCCAAGTTTCACCT
CGCTCTGCTTACTTTCAGCTTCATCCGGTTCTTCGGCATTGTCGGTAAGCTCTTCCATATCCG
GCTGGACCTTGTCCTTGTACGTGCTGCCCAATAGTTGAACAGATTTCAAGTCCACCGCCCCC
TTCAATCCTTTCTTCCCATCTTTGGATCGCCTTTTGCGACAGCATCTTCTGATGCAGCAGAAG
CAGATTCCGAGAACTACTACGCTTACAGCTATTAAAATCGCAACAAGTCCCCATGTTGGTA
TCCCCATTTCCTCCGCGATTTCTTTGCCAAGATTTTCCATTTGAGTTTCAAAATTTTTCCCAGC
ATTTTCAGTCACTACCTCTGCCTCTGTGGCTTCTTTTATTTCCATCTCGGTGCTCAAAAATGTT
TTCAACGTCGATTCAACGACCTCCTCCGGTTGTGGCTCTTCCGGCACCTTCTCAGCTTCTCTC
TTGTTAATGGCAGGCATTTTGATGCTCGTTCTCGGTTGGCTCCTGACTACTTCGAAACGAGG
TGTCTCGAGATTCCTCCTATCCTCTGTCCTCTGTCTTCGTTCGTTTCGAAGATTTCGACGCGG
GCCGGATCCGTGGAAACAATTAGCGAACGTGTTCTGGCGTTGTATGGCAATGAGAAAAGA
GCGAGTGGAAAACACGGGGTCTCGCGTTTTACAAGCGCATCATCGTTTTATACGCCCTGCG
ACATCGACGACGTTGTAGACGGTGGAAAATCTATCGGTGGCACGGTCTCTCGACGTTGGGA
CACGAGTGCGCCTGACC

9. >MSTRG.255.2 Gene=MSTRG.255
TCTAGTTTATTAATGAGTATCACTGATTTCCGCCATTCTCTCTTGCCCCTACCAGGCGTCGCG
AACATATCGTCTTATTATGATTGTAATTCTTGTATTCTTGTTCTTAGTTTGAATTGAGTGTGTT
TGCTATCTTATTGTAGAAATTTGCAACGACTGTCAGTTTCCTAACCTCTTTGCCGACGAATGT
TCCGGTGTTTCATATTGTGTTCAGTTAATAGTAACATATAATCACATAACATGATGTCGCGG
TCTGTGCGTGCAGAGACTCGTAGTCGTGCCAAGGATGATATTAAGCGTGTAATGCAAGTCG
TTGACAAAGTTCGCCATTGGGAAAAGAAATGGGTTACAATCGGAGAAACTACTATGAAGA
TTTATAAATGGGTACCTATATCGACACTTGATCAGAAGAAGAAAGGAAAAACAGTTGCAG
ACAAAGAAAATGGTTTGCCAAGAAAAAGTGGATTAGATTCTTCAAATTCCAACTTTGGTCT
TACAGAGGATTCAAATACATGTTTTTCAACTGTTAGTGATTCTCAAGGATTAACAGACTTTT
CTGCACATCTGGGATTTTCAGAAGATTCCAATTCACAAAATAGTGAACCAACACCTAAAAG
TATCAATATCCATGGGCGCCCCGTTACCGCCCATATCTGTACGCACCCAACCAGGATGAAA
ACATGCGACGAGGA

10. >MSTRG.276.1 Gene=MSTRG.276
AAGCGTAGTAGCAAGTAATGATAGTTTCGACGAAGAATAGTTTTTTTCGTTTGTTTCTAGAA
ATCGACAACAATCGACGAAGAGAATCAACTATGATCGCTTGTTTTACAATAATCCTGAGTG
TTAAAATTTATCTGAAAACAAGTTATTTCTCAGCTTCTGTATACTCATGATTAAAAATCATC
GTTAAAATTCATTTCGATCGTTAAAATATAATTTTAAAAAACACGTATAAACATATAAATTT
TCAATAGCAATAGATTAAAATTGAATCCATCCGATTTTTAAATTTAAATTCTTCCCTGTCAA

ATGTACAATTATACAGACGCTGCTGTGTTCTATGTTTCGAGCATTTTTCGAGCATTTTTCTTA
AGTCTTTCTTTATTTGTTTCCCATCTTCACTGTCGATAGTTATGTTTTTTTTTATATCGCAACG
GTGATCGACGTTGACGATGTTTGATTCTAATTATCTAACCCTTATCAATATTTTAATGATTAT
TTTAGTACGCGTGATTCGCGACGAAAAGCGTAGCATTGCCAGCAGCTCCCACTACACCACC
GCTGTATTTGCCGAGTGCCGATTCGCTGTTAGCCTTAGCCCTCTTGATAAGCTCTTCCTGGCC
AGCGCTAATCTGTTCCTTCTTGCCTCCCCATGCGCGAAGCACGGAAGCTTGAAGAGCACGA
CCATAGCTGAAGGTCAAAGCCCAAGGTTTGTTCGCGGGGAACTTGTTAATAGCATCCAGAT
TCACAGACGCCTCCTCTTCTGATTGACCGCCGCTGAGGAAAGTAATTCCAGGTACTGCAGG
TGGAACAGTACGCAATAAAGCGGTCACAGTAGCTGCTGCGATTTCCTGAGGTGACGCTTTA
TTTGGGCAACTCTGTCCTGGTGTTACCATGTTCGGTTTCAAAAGAGTTCCCTCAAGATACAC
ATGATGGTCACTGAGTGCCTTGTACACGGCGGCCAATACTTCTTCCGTGACCTGTTGGCAAC
GGGCAAGATCATGATCACCATCAGGTAAAATCTCAGGCTCGACGATTGGCACAATTCTAGC
ACTTTGACAAATAGAAGCGTAACGAGCCAAAACATTGGCATTTTCCAAAATGGCCAATTTG
CTGGGACAATCTTTTCTGATTTTCAGCACGCATCTCCATTTGGCGAATTGACATCCATCTTTC
TTGTATTGAATACAACGAGCTTGAAGATTGTCGAGACCCTGCGTAGTGGTTTCTTCCTCGGT
ACCAAATAATGTTACTACACCTGTGTCAACCTTGATACCTGGCAAGATATTACGTTGTTTCA
GCAATTCGACGAATGGTGTTCCATCATCAGCCTTCTGGTACAAGGTCTCGTGGAATAGAAT
AACACCAGATATATGCTGGCTGATGGCATCTTTAGCGCTGGTAAAAAGAAGTTGTCTGTAT
GCTCTGCGATTGTCCTCAGTATTTTCAACATTGATGTCTTTTAGACGCTTGCCGATCGTTGCG
GTCGATTCATCCGCAGCGAGGATTCCTTTTCCGGGCGCAACAATTTGCTGGGCGATGCTCTT
CAATTCTTGTTTCAACTCAGGAGTGAGTTTATCATATTTTTCTGCAGCCATTGTCTTATATCG
ATGAGATGATTTCTAGCTCGAAACGCGTATACGTAAATCGTACGATTTGCTGGTTGGAAAG
TGACTGAGGTAAGACTAGGCTATTCATTGGTTTCAAAGTCTGATACAGAATAAATGGCGCA
AGCGCACTCTCTCACATAATTGCAACGATTCTCAACTTTCATTTGAATGATCATTTTTATAAA
CGAC

11. >MSTRG.280.1 Gene=MSTRG.280
CGGAATAATTGTGAATAAGTGAAATTTGAAAATGAGACAAGTAAAGCAGTAAAGTAGTAT
AGATCAATCATTTATAAGAAAAAAATTATTTAGAATCGCACGAATAATTTTTTAATTGTTGC
AAGTTTCGGAATAATTGTGGATAAGTGAAATTTGAAGATGAGACAACACGTGCCACCGATG
CATATAGAATCTGATGAGAGAACGTAGTCTATATGCTTTCCTCGGACGAGAACGTGTTAAA
CATTCATCTCGAACAATCAGTTTCCGCAAACTGCGCCATGTGTCACCCAAAATCTGCTATAC
ATGCCTCACGTAACACGTCTCTCTCTTACTAGTTTCTCCAAATCTCATCTATGATTAAATTCG
TTTGGAACATTAATTGCATCATCTTGAATAATTTGCAATTAAATTTGCAAAAAATTAAAGAA
AGAAAGG

12. >MSTRG.302.1 Gene=MSTRG.302
AAAAAAAAAAATCATTAAATTTTCAGATAGAAAATTAAGGAAAAAATGGATCTTTCGGAT
TCAATTAATCTGACATTGCCTTTTAAATTATTTCTTCACTGCTTCAAGCAGTTAATACATCTC
CTTGGTTCTTCGGTATCTCGTTGTCGAAATCCTCGACGCTTTCCACACTCCTTGAAGATTGGG
TGCTGGTCTCATTCTTTCCGATAAGTGTAGGCAAAGTGGTCACATCGCTGTCCGCTTCGCTG
CTCACCGTTGTCAAGATTGTTTCATTCTGGGGTCTCACGTCGATCACACGGACGCGGATCAG
TGTACTGTAGTCGTCGCTACCATCGGTATAAGTCGTTTCATTAATGGTTACCACGTGTCCATC

GATGATCTTGGTGGTAGAGGTAGTGTTCGCTCCTTCAGGAATCTTGTTCCAATTCACAACAC
CTTGCTCGGGAATACGTGACAAAATGCCAGCCATCTGCTCCCTCACTTTCTTCATGTGAGCT
GAAATTGCACTTTGTAACGTCTGGTACCAGCCATCCAAAAAGTTAGGTCTGAGCAGCGTGT
TCCAGTTCCAGTTGGAGCCTTCGTCGCTGTCCTCGAAGCTCAAACTCGGGAAATCGAAAGTT
GCAGCCATCATTTGATCTCTTTCAATGGACGGTAGGACGAGAACAGTGTCCACATTCTTCCT
TTCCTCCTTGCTATCCTCATCGTGTGCACCAGGGAAACTGTGCGTGCATGCGATGAACCACG
CTGCAATGAATAGAACGCCAAGGGTCTTCATTTTTCTTTGCACCAGAAAGTCGCTCCGGTAA
CTTTCTACGACGATGATTCGATTAGTACTGACGGGTTCGGCTTCTTGCGAACGGGACTGTCA
CAGAACTCGCGAGAGGATCAAGGTCACTCACAGAACCAGCTCACTGAAACTCCCTGACGC
TTTGCACCACGACTTTTGTCTCGCGCCACTCTATATGTGATCGTTCGCCTACTGCTCAACCAA
TCGTCACGCTTCTTGTCGGGATCACGTGAACAGTCAGTCACGTGATCGTAGGGCGCGCTGCT
TGACTCATAATATATCTTTTCCCGGTATT

13. >MSTRG.315.1 Gene=MSTRG.315
GTCCAGGATACCGGGCTCCTCGGTTAAGGATCTCGTGGTAAGCCTCTTACGCCTTCTCTTGT
GCATTGTCAGGAATCGACCGGTCGGCCTCGGTCTCGAGCGGCCGATATCCAAACTCTCGAG
AAGGATACGATCGACCTGTGACAAACGTCTGTACGCGGGTTCCGTCCTTGGTTCGTCGCGG
ACTTCCGCCAACGTGGCCGACACGCCGGATGTTGGCAGCGCGGCGCTCTCGGAAATGCGG
ATGAACACATTCTCCTCCCCACTGTCCACCGTTATCTCCGCACTTCTTCTCTCCTGCGTCTCG
ACAGCCTCTTTAACGTGTTCCGCTATCTCGGCAGCCTTGAGCCCCGTCTCGATGGGCGCTAG
TTGCGAGTGGCGGCTCGTCCGGGCGTCCGGGGTGATCTCCTGGTAGCAAGACTCGCCCGAG
CCGCGTCTGCTGCCCGCGATGCACATGGCGCGCCTGAACAGCCCCGCGACCACGGCGCAC
AGGCCGCACTGCATGCCGCCGCTGCCTCGGCTCGACAGCGAGCCGCCATTGGTCCGTCGAC
GGGTCCGAGTCCCGCGCGAGCTGCCCTCGCGCGATCCCGACGCCAGCTTTCCTC

14. >MSTRG.345.2 Gene=MSTRG.345
ATTCGGTTTATTTTCGAACACTAACCCGGTATTCCACCGTGTCGAAGTGCTGACGTTGAAAA
AATAAATTTAGGAGAAATTAATTTTGATTAAAATTTTTTAATGCATAATTTAATTAATAAAT
TTATTAAGTTTTGACATTTTATTTATTGATTATAAAATATAATAATGCATACTGCTTTTATGCG
CGGTAATGCTATTTTAGCATATACTTTGAGTGTTTCGGCATGTTTAACATTTTGTTGTTTTCTC
TCTACTGTATTTATTGATTACAGAGCAAACGCAACTTTAAATACTGTAAAAGTAGTTGTAAA
AAATGTAGCAGATTATAGTGCTTCAAGAGAGAAAAATGATTTGGGATATTTAACATTTGAT
TTACAAACTGATCTCACTCCTTTATTTAATTGGAATGTTAAGCAGTTATTTTTATATCTCACA
GCAGAATATCAAACAGAAAATAACAATTTTAATCAGGTAGTATTATGGGATAAAATAGTA
CTTCGTGGAGATAATGCTGTTCTTGACTTTAAAAATATGAACACAAAATATTATTTCTGGGA
TGATGGTAATGGTTTAAGGGGAAATAAAAATGTAACGTTAATATTATCATGGAATATTATA
CCAAATGCTGGACTTTTGCCAAGTGTTAATGCATTTGGTTCTCATACTTTTGCCTTTCCATCT
GAAT

15. >MSTRG.365.1 Gene=MSTRG.365
GTTGCTTCGCCGATTGCTTGTTTACGAGATTTTATCAAGATTTTGCGTGAGTAGAAGTTACTT
TGGTGTCAGTGAGATTCTTCGATACGAAATCAAGGATGTTCCTTTTTCGAAAGGTTGATAGT
TCAGGTTAGAAGTTTATTCTCAACAATCGATCAATCTTAAAACAGAACCTCTTTCGATGTCC

ACCTTTACCTAACCTCAACAACGAAAGAAAAAAGATCAGTTTCATCTCTCGAACGACTCGA
TCAATCGTGAGGACGATCAATTTCAAGTATTCACCAATCGAAATCAATCGAAAGGTACCCA
AACGAAACATAACCTCTTACGTAAGCTCCCTGTTCCCCCTCCACTATCCGTTCCACGGTGAA
GCGCGATACCAGTCCGATAATTCCATCCGATTAAGATGCACCTTGTCGAGACGTGCTCGCG
CAATTCACGTAATTCGCGCAATTAACCCCTTAGAGCCGCATTCCCGCCCGTTCCTCGGCATC
CGGCCCCCTCGTTCGTTCATCGAGGTTTCGCTGTGAAAACGGGTTCATTCATGAACCACGGC
TGTGGTTCGGCAAAAAGCTCCATCTTCCCTCTTTTCCCCGCTCATCCTCCTCACAGTCCCACC
TCTTGTTCGTCGCTTTGCTCGTCTCGTCGGGCCGAGAGAGAGAAAGAGAGAAGGTGCACGT
AGAAAACTGGTATCGGTACAATGCCATCGGAATACGCTATCTCAGCCGCTATTTCAGCTCG
AGAGGAGCGGGTAGAGCAAGAAACAGACGCCACCGGAAATCATCATTCTAACCGGGTAC
GTTTAATCGAAGGGAAAACCGGAATACGCCGCGGGCTGACTGACAACAGGATCAACCTGC
TCTGTGGACGTATCGAAGCCAGGATTCCATTCCGGCTCGATTTTTCAACACCGACACGGCG
ACCGCAAGTATCCCCACTCGTCTCCACGACCGACTCTTTTCTTCTCGATCGCTGATTAATTAC
ACGTATTGTTAGACGAGCGTTCTTCTTCTTCGATCGTGTAAATGTGGCGCCGTTCTAGTCGA
GTGGGAATGGGAAACGTCGGAAACGTTGGTAATACTGGTCTACATTCGCGCTGCGAACGTC
AGAGTGCACCGCAAAGTCCATATCCCTAAGTTTCGAGGATACGACACGAGGATATCGTGGT
CGGCACGTACACCAGCTGGTCCAACTTCTTGTCCAGCGTCGACAGCTGCAACGAAAACGAA
AAATCGGAATTGGAATCAGTGAGAGCAAAAACCCTGACTTCTGGAGGGGATATGTCTGTG
GATCATTCGTGGCTCGAAGAGGAAGAGTGCAAGAGAGAAAGAGAGAGAAA

16. >MSTRG.407.1 Gene=MSTRG.407
CCTCCGTGGTTACCGTTATTACCGACAATGGTTCAGGGGACATGCTGCCCAATCCTTCGTCA
CCGCTCTCGCTAACGTGCATCGGTAGGCTAACAACGACGCCTCCTTGATTATCTGGTTTGCG
TTTCTTGATCGGAACATCGCCACCCTCGTGCTGATTCACCAACCGTTTCAATTGACAATTCT
GTGACAACAGTTGGGTCTTCTCTTGTTCCAGCTGGTATATATATTCGGCAGTCTGCTGAAGT
ATCGCAGCCTTAGAAAGTTTCTCGCCCTCGTGGTGCGGCAGCAACGTCCTCAGCGATTGGA
AACCGGCGTTAATACTCTGCATCCGACGTCGTTCATTGCTGTTGGCGATTTCTCGCCGCATA
CGCTTCTCCTGCTCCATGTGGCTTCGCGGTGTCACACGGCCGACGTTGCTGGTACTATTTCCG
TTGCTCAACGCAACGTTTTGACCAGCAACACGGCGAGATACTGCGCCGTTGCTCTTCGATTG
CTGTTGCTGTTGCTCTTGCGAATCAACGACCTCGGCGATCGTCACGCTCGTGGCCACCGAGC
CCTCCTCCACTGTTCCGATGCTTCCGCACAAATTAGTTTCTTGTTCCTCGGTGGTTTCAACAA
TTTCTTCAAGTAGAATTCGATCCTCACTACCAAACACTGACATATCTCGTTAGTCCGCAGCA
CTGAGAATCCCGCCGCGCGATTCCACGTCCCAGCAACCGTCGTCGCGGCAGCGAAAAGAC
GACGGGCTTTTCTCGCACACTCCACAAAAACGACGCGCACACCTCACGCCTATGACGCGGA
TTCTCCGGAACTCGTTAATTAATTTTTTAAACCACGGTGGCACGCACCGAGATTCGTCCACG
GAACAGCCACTCCGCGCGGCGCACACACAACGCCCGAACGCGTTTTGTAATGGCGATCTTT
GGCGGGAAACAGCTGAGGGCCCCCACAGAGTACCACGAATCACTCCGCCACCGGCTTCAG
GGCCGTCACCATTTATGGTCCATTCAGTGCCCCTTTCTCCCTTTTATAAATATGCG

17. >MSTRG.409.1 Gene=MSTRG.409
CAGATAATTTTCCTTCGGTCGCCGAAATTTCGACGTTTATACTTTTTTTTTAATATCTCGCTTA
TTATTCAGAATATTAAAAAATGTTTCAAATAAAAGTTGATTGGTTTCGAAAGGGCTATATTA
TGACGCTCTCAGTTTTTTAGTAGGTTGACCAATAAGGAGCTTTTTTAGCACAACTTTGTTTTT

TTTACTGATAACATGTATTTTTTAAAGCGCCAATCGATAGAGCATAAAATTTGCTATAAAAA
AATATTAACCCACTTATAGCGAAAGTCCTATGATTTAGAAGATATAGCAAAAATTTAAAAT
TTTTATCACTATTTCAATCTAAAATTGAACATCCTAGCATAATATTAAATTACAATAAACGC
GA

18. >MSTRG.426.1 Gene=MSTRG.426
ACCGCGCCGGCGACACGACGGCTCCCCGCCGACCAATAGCTATTGATCAGCGGCTCTCTTG
TGCCTGTGCGTGGCCGACGGCTTTGGAAAACGTGCAACAGATCCTTGGACCGAGCAATTCG
AAAGGGACATTCGAAGCCACAAGCTTGCCCCTTTAAAGTTGGTCACAATTTATTTCGAGCT
ACGTGGATACTACGCAGCATCCTGTTTCCGTCCACGATCCCTCGCTGTTCGAGATTTTCCAA
TGGATTGAAACTTGACATGAAGATAATTGCACTTCATTTTGAGATGGTCCCGAACATCGGAT
AGATCCAACCTATGATATTACCGTCAAGACATCGAAGACAAGATGATACGTTATACGGTCA
ATCATACATCCTCATGCAAGACATTTTATCCATTTTATTTTTGTTAAAACTTTGTCTAGTATGT
AGAGTACAATGAATCATACGCGCAGTAATTTCTTTTCATCCCACAAATTTTTATCATTACTTG
GATCTTTTGAATACAATTATGTCTAAAATTTTTTTCTGTCTCATATTACAACAAGATATTCGA
AAATTACGATAAAGATCTCTCTTATATTCCAGTGTAGTCGTCTATTTCCCCGAAAAGAAATT
AGAATAGCTACACAAGCGTTCGAGATTTGATCAATTAAATCAAATTACCCGTCACGCAAAA
CACCT

19. >MSTRG.438.1 Gene=MSTRG.438
ATCGCTACCAAAATTCCCATTTCATATTTTTTACTAAATATCTTGTTTATTATTCGGAATATTA
AAATATGTTTCAAATAAAAGTTGATTGATTTCGAAAGGGCTATATTATGACGCTCTCAGTTT
TTTAGTAGGTTGACCAATAAGGAGCATTTTTAGCACAACTTTGTTTTTTTTACCGATAACATG
TATTTTTTAAAGCGCCAATCGATAGAGCATAAAATTTCCTATAAAAAAGTATTAACCCACTT
ATAGCGAAAAACCAATAGTTTAAAAGATATAGCAAAAATTAAAAATTTGTAGGAGGTCGA
CCTCCTGTCATATCATGTTCTCCTAATACAAAATCGAGTTTTTGGCCAAAAATTTTCCTTCGG
TCGCCGAAATTTCGACGTTTATACTTTTTTTTTAATATCTCGCTTATTATTCAGAATATTAAAA
AATGTTTCAAATAAAAGTTGATTGGTTTCGAAAGGGCTATATTGTGACGCTCTCAGTTTTTTA
GTAGGTTGACCAATAAGCAGTTTTTTTAGCACAACTTTGCTTTTTTACCGATAACATGTATTT
TTTAAAGCGCCAATCGATAGAGCATAAAATTTCCTATAAAAAAGTATTAACCCACTTATAG
CGAAAAACCAATAGTTTAAAAGATATAGCAAAAATTAAAAATTTGTAGGAGGTCGACCTC
CTGTCATATCATGTTCTCCTAATACAAAATCGAGTTTTTGGCCAAAAATTTTCCTTCGGTCGC
CGAAATTTCGACGTTTATACTTTTTTTTTAATATCTCGCTTATTATTCAGAATATTAAAAAAT
GTTTCAAATAAAAGTTGATTGGTTTCGAAAGGGCTATATTGTGACGCTCTCAGTTTTTTAGTA
GGTTGACCAATAAGGAGCTTTTTTAGCACAACTTTGTTTTTTTTACGGATAACATGTATTTTT
TAAAGCGCCAATCGATAGAGCATAAAATTTCCTATAAAAAAGTATTAACCCACTTATAGCG
AAAAACCAATAGTTTAAAAGATATAGCAAAAATTAAAAATTTGTAGGAGGTCGACCTCCT
GTCATACCATGTTCTCCTAATACAAAATCGAGTTTTTGGCCAAAAATTTTCCTTCGGTCGCC
GAAATTTCGACGTTTATACTTTTTTTTAATATCTCGCTTATTATTCAGAATATTAAAAAATGT
TTCAAATAAAAGTTGATTGGTTTCGAAAGGGCTATATTATGACGCTCTCAGTTTTTTAGTAG
GTTGACCAATAAGGAGCTTTTTTAGCACAACTTTGTTTTTTTTACCGATAACATGTATTTTTT
AAAGCGCCATTCGATAGAGCATAAAATTCCTATAAAAAAGTATTAACCCACTTATAGCGAA
AAACCAATAGTTTAAAAGATATAGCAAAAATTAAAAATTTGTAGGAGGTCGACCTCCTGTC

ATATCATGTTCTCCTAATACAAAATCGAGTTTTTGGCCAAAAATTTTCCTTCGGTCGCCGAA
ATTTCGACGTTTATACTTTTTTTTTAATATCTCGCTTATTATTCAGAATATTAAAAAATGTTTC
AAATAAAAGTTGATTGGTTTC

20. >MSTRG.463.3 Gene=MSTRG.463
GTCCATGGACATAAATAGAATATGGATTGTGACGCTACTGTGCCACGACGACTATTGCCAC
GATGGCCGAAGAGACTGACACAAACGATAAAACGACGATGGAAAGTATAGCGCAAAATA
TACTCGAGGTTCGTGTCAGCCGTGATCAGGACAGTGCGTGATAACGTAATAGTGCCTTTCG
AAGTGTCGAAAGAAATCCTAGTACTAACAACACGTTATTATTCCGAACTTTTTCGATCATTT
TGACAGGTCAGTACCGGAGGTGGGTCTGCATCGAACGAAGTAGCCGCATTAGAGTTTCAAG
CTTCACAGGTAATATCGAGGCTCGAGGAAGCTGTAGAGAGGGCAGCCGACGGTGGCGGTA
CCACATGGAACAATCCTTCCGGATTTCTTTCACAGCCAAAAACTTCTGATGAGATATTAACT
TTAATTCAGGATTTGGTAATTCACGAGGATGCCCCACAATCTGTTAAAGATGGTTCAACAG
AAACAACAAGTCAAGTAACTACTTTGCCAACATTAACCGAGCCAGCAAAATTAGCTTTAAT
CGCACATACTATATCCGCGTATGCGCTTGCACTTCCAAAATCCCATGCTCAGAGACTAGCTG
GCCGATTGGCTGCAGATACAACTAGATGGCTAAGTCATATTTTCCGTTTCGTCGATTGTGCC
TCTTCATTTCACGAGGATCATTTGGAAGGTTTGGTCCGAGTGACCAGATTGGCTCTACATCG
AAAATATCCTCGATATATGGAAGAAGGTTTTACTGCACTTGCAGCCTCTCCACCTTTAATTT
ACAGTTCTGTGGCTGCACCTTTAGGAGTTGTTCAACATTTGTGCAAACAACTTTCCTTGCCA
CTCCATTGTGTAAGGCCCATTCCCCATAACACAATGTTTGGATCGCGATATAGCATGGATGT
ATCAGCTTTAGAACGCAGACTTGCTGAAGATACACAATCAAATAATGTAACGCCACTTCTG
GTATTAGCAGAAGGAGGCTCAGTTTTAACAGGAC

21. >MSTRG.474.3 Gene=MSTRG.474
GCGAAGTCCTAACGAGTAGCCGGCGAGCGAGCCGTATCGCGGAAAAACTTGTTCGCAATT
AGATCGTGCCGAGATTTCCGTGTCCGTTCTTCGTGTAATCGGTTCCACGATCCGCGTCGCTTT
CCTTTAATCCGTGTTCTCGTCTACATTATTCGTCGTACCTTTTTTTTTTTTTTCAAAAATCGGTC
GATCCTCGATCCAAGTTTCGAAACTTGAGATTAAAAAGAAAGATTGTATGCAGTGAGTATC
TGTAATCGTAGATGTGTCGTGAGCAGTGTTTCCAGGCAGAGGCACGAGATAGGGGGGGAA
GGAAGTCTCGATCTCGGGTGACATGTGACGAGTTGTCGATCGTGCACGAGCGATAAAACGC
CTCGTGAACAAATCTTCCGCGTTCGATTTATCCGCTGGATCCTCGACCGACCCATGAAAACT
CGATGGGATGTATCAGTGAGGAACGAAAGTAGGTGTTTTCTTGTGATTCGGGGAATAATGT
GCCTCGTAACATTATCACGATTCCAGTGTATTTATAGATACCAGTGAACTACTCGAGGATAC
CCGATATACATTCCGTGTGTCGTCATCGTCGCGTCATCGTGGTCGGGGCTTAACGAGGCGCA
GTTAACGTCCGTCGCCCCGTCGTTAGGGTAAACGCGAGTGGGTCGCCCGGGTCCTCGAAAT
TGCCGCCACGATTGCCGGAACGATTGTTGGATGGTTGGATGGTGAGAAACGAAGTGGTGGT
TCGCGGGATCGACGTAGCGGCCGCCGCCTCTCGATCGCTCGCAGCAATTTCGGCATTTTGG
AATTTTGCAAGGGG

22. >MSTRG.510.2 Gene=MSTRG.510
TGATAATAAATATATTTAATAATAATTGGATATAATCGAAAATATATATAGTGAAACGAAG
TAAAACTAAGATAAAACGAACGTAATGTCTCTATCTAAAATATTACAAAAATTAAAAAATT
CTATGCAGAAAAGACCATTACTCTTTAATTCAATAATGTACGGTTCTTTTTATACTGGTGCG

GAATTCACACAACAAACTTATAATAGAATGTTTAAGTTTTCGTTATCATCAACATCAATAAA
CATAGAAGAAACAAATACATTTAAGATTGAAAAGTCGCTGTTTATTTGGATAAAAAATTTT
AATCAAAGATTGGATTTATTAAATAAGAATAACACGATGCAATTGGAAAGTTATAATTGGG
CCCAACTAAAGCGATATGCTATTTATGGTTGTTTCATTGCAGGACCTCTACTACATGGATGG
TACAAATGGTTGGATATGTTCTACAAAGGCAAAACTATGAAAACTATCCTCACAAAACTTT
TAATCGATCAATTTGTTTTTACTCCACCATTAATTACACTATTTTTTATCAGTATGAGCCTACT
GGAGAATAAACCAAATGTATTTGATGAATGTAAAGCAAAATTTTTTCAAACATTTAAGACA
TCATGTATATACTGGTTACCAGTTCAATTTTTTAATTTTCTTTTAATACCACCAGTATTACGC
GTTTCTTTTGTTAGTATTGCAGCTTTTTGTTGGGTAAATATTCTTTGTTATTTAAAAAGTATTC
CTGTATCTGAATGTAATGATAATCAAAT

23. >MSTRG.543.1 Gene=MSTRG.543
AATTCGTGATATTCGAGAAAACACGAAGTAGTAAGTAACTGATTGCGCGGGTAGACGTTAG
TGCGTAGGATTTGTAGAGTATAATACAAAGAAAAATGGCGATGGCAACAACTCAAGTTAA
AGGAAGCGGATGGCCGAGGAGAGCTAGTAACAGTTCGTTTGAAGGGAAAGTTGTACAGAA
CCAGTCTAGTTAATGTTTTGACTGCCTCTCCTGTAGAACAACTATGGCCGGTTAAGAGACGC
TAGCAATTAAAATTATGTTCCTAACACCTTTCAATGCATCTTACATGGTACAACCATGAGAC
GGCTGTTCATCTTTTCGTTAAGTACACATGGAAATTTTAAGCAATATTTCATTGAAATCATTA
TTATTAAAATTATTATTGATTAATGATTTAATAATTTATTTTTGCTTATATTTATCGTGAAACA
TAACCAATTATTTTAAAATGCCATGAAAATTTTCACGTAATAAAATGTTTTCTATAAATTATT
TTCTCACAATCCATAATATATATATCTTCGTTGAATTAAAAATGAATTAAATATATGATTTTT
TGCTTAAAATTTCCAAAC

24. >MSTRG.553.1 Gene=MSTRG.553
TGTCGCGTTTACAATGAATAAGAAAAGCTCGTGTATAAGCGATTTCGATGTTAGTCGTTAAT
TGTAAGACCGCTATGCGCGTCGTTTATTTAAAAACATTTATCCAGAATTATAAATATTATT
GATTATTCAAGTTCAAGAATAGTTTTTAAGAACTATGTTTGGCTGAATAACTATCATTTCTTG
GAAATTTTGAAGAAAAACGAATCGACACTCGCCACTTCTGACACTTCTCCACTTCGTTGTAC
TATCACCACGGTGTAATGATTACAACGACAGACGATGTTCGGAAAAAAGGATGCAAAACT
GTGGCCGGAAGTCGTGACGACTTGATGAAGTACTACGTCGCGTTTATACATATCCGACCCG
CGAAGATTTAAATACACTTATAATGCAAGCAATTCAATAATATTATTTTGTATCTATCTAAA
TCTAAATTATCATGAAACATGAAATAATAAGTAATAAATAAATGAAAATTCACTTTTTTTGT
CAATTTATATTCTTAATTGTTATAAAACCTAACAAAAGTTATGATATATATAAATATAAATA
AAGTAAACATTCAAAATATC

25. >MSTRG.557.1 Gene=MSTRG.557
CCCACCGTGGCGACCCTCAGCGGACAGACGGAGAGCTTTGCCTCCGTTACGTGTAGGCGTA
TGTACTGTGCAGGGCCGTGCCGTTATGAATTTGAACCTTCGAAATTTAAACGAGTCGGAGA
AACTTGATCGCTTAGAGGCTTATGGTGTTACTATTGACGCTAATAATGAATCTACATAGTTT
GCAATACCAGAGACATCTGGTGATCAAAGGAGGAAGCTCTTGATATTTTTTACTAATAGAA
GGCAGACCAAGACCGCGTTTCCGCTGCAAGCAGCTATGTATATCGTATGAACCTTTGCCAA
ACGGCGTTCGTTTAACCCTTTTGTTTCGTCAGAACTTCGTAAAGACAGCACTGTGATGTCCA
AGTGAAACGATG

26. >MSTRG.581.4 Gene=MSTRG.581

CACTGTCCTGGAATATATTACTGACATTACTCAGCTTCTAGATGCGAAAGACAGTGAATAT
GTATTCTTTTCGTATAAGATTCGTATAAGCTTCCATATATCGAGTGAAGCTTAATTATAACTG
TTAATCAAAATCGTGAAATATATATACAGCATTGACATATCGGTCAAATAAATTCCAATTA
AACATGATACCGCCAAAAGATGAACCGTTATGGTCTGATATCGCACTTCGTATGTTAATGA
ATACCGTTTCACATCCCATTGAATACGCTAAAGTTTTAATACAGGTAAAATATGCACAGTG
GATAAAACCTGGATATTAAACCTTCATCGGAAGCTCATAATTTTTATTTATAACGCAACAGA
TCGGATATGAACCGATTCCACCACGACCAACAACTACTTTATTTGGACAACCGGCTTTAGCT
CTACCTAACGTATTTCAATACGTTAGGTACATAAAAAAATGTAGATGGATTTACTGGCTGTTA
TCGTGGTCTTATTCCAAAATTATGTGCCTATACTATGAGTGCAGTAGCTTTTGAAAAAACTT
CTAAATCTATTAAATTTAAAGATGAACCAAGTAAAGAAATTTATGATTGTGATTTAGAAGA
AAGTCAGAAACATAAAAAATATATATATGAATTCATAAGAGACCTGATTAGTAGAATGATT
GGAATCATAGTTAGTCATCCATTAGATGTCATTGCATTAAGAATGATGGCACAATTTGTTGG
TGGAGAAACAAAATACAATGGATTATTTAGATCATTTGTGGAAGTATATAAAGAGAATGGT
ATCATGGGATATTATGCTGGATTATTGCCTCGCCTTATTGGAAATGCTGCTGTACTAATGCT
AGTCAGTTCATCTACTTATGCTATTGATAAATACATTATAAGTGATCGCGAATTAAAACCAT
ATGCTGTTTCCACCATAAGGTTTATAGCAACGACAATTACATACCCATTTTTAGTAGTGTCA
CATTGTATGGCAATAAATAATTGTGGGTTAATTGCTGGTCTTCCTCCACAAATGCCAATTTA
CAATAGCTGGTTAGATTGCTGGTCTCACCTTTCAGCTACCAATCAGTTAAAAAGAGGAAAT
AGCCTTTTATGGCGTTATTATACAGGACCACAAGTGATAATTAATGGAAAACCAATACCCA
TTAATAAATACAATTTTCATTTACAAAG

27. >MSTRG.625.4 Gene=MSTRG.625

CGAACACATTATGATCGCATTTCGAATTATTACTGTATTTTTTGGATCTTGTATTACTATTTTC
TAACGAGAACGAATTATTTATGATATTTTCGATATATTTAAATTCTTCTAATACATTATTAAG
GAAAATCGATTATTAAGGAACAAAAACATGTCACAATTTAGTTGGTATAATACCGTGTAAA
GTACTTCAAGTGTCTGATTGTATATAGCAGTTTAGAGAATTCGAGAACGTACAAATTCTCAA
AATCATATTTAATTACTGCGATTTGCGTTTATTAATAAACGTTGCAATTGTAATAATTTAAAA
TTTAACTATTTAATAAAACATTTTATAATAATGATTATACCTGTACGTTGTTTTACCTGTGGA
AAAGTGATTGGTAACAAATGGGAAGCTTATCTTGGTTTATTACAAGCTGAATATACGGAAG
GAGATGCTCTTGATGCATTGGGATTGAAACGATATTGTTGCCGCAGAATGCTTCTGGGACAT
GTAGATTTAATTGAAAAACTCTTGAATTATGCACCATTAGAAAAATAAAAATGTAAAATTT
TATATAATATTAAGATATTATTATTTATATAAAAATATATTTTTTTAAATTATGTGAGAAAGA
AAAGGAATAAATATACAAATATTATTGTAAAATGATTTGTATTTAAAAATCGTAATTATTTT
TTTTTACCAGAAATATGTAAAAATTTTTAACAATGGTATAAAAAATTGATTGATTTATAACA
AGTTCAATAAAAGTTGCTTCAAAAACATGCTTTTTTTAAATATACATTCATATTAAATGCAA
CACAGAAGAGTGAAACATTTTCTGTATTGTGAATCACATGTATGAAATATATAATGATCTGC
TTAGACGTTAAATATTACAACATATATCATATAATACAAACCATATAATGTATGTTACTAAA
TTTGGAAAAAAATGAAGGTTATATTT

28. >MSTRG.641.1 Gene=MSTRG.641

CTCCTCTCTATTATCCTCCATTTTTCTCTACTGGCACTTTTCTACTTAAAACCTACGGCAAAC
CTTTTATTTCCTTTTTCTCCTCCCTCTCTTCACATCGACGATCGTGTATAGCCCGGCGTTAACC
AGGTGTCGCCGGGAACAACCGCTAGCTCTCCCTCGCGACGGCGTCGAGACGCCTTGTCGTT
GCCTTCCTGTCGTCGAAGCGCTGCTACAAACTACGATTGTTGCTCTGCTGGTGTTGCGTCGTC
GCGTTATTGTTATTACCGTTCGTAGCTTGTTGCTGCTGTTGCTGTTGCTGCTGCTGCGGCTGTT
GTTGCTGCTGCTGCGGTATCTCGTCGTCGCGTAGACAGGCGGACTCGAGGTGCTTGTTCAGA
TAAGACTTGAGGGCGAATGTCTTGTGGCATCTCGAGCACTCGTAGTTCTTGTCCGCGGAATG
GGTCTGCATGTGGGCGCGGAGATTCGAGCGGTCGGCGAACGCTTTGCCGCAGTGCGCGCAC
CCGTAAGGCTTCTCGCCCGTGTGACTTCTCAGGTGGCCTTGGAGCAGCCAAGGCCTGGAGA
ACATCTTGCCGCAGACGCCGCAGCTGTGGGCCAGCTTGTGCGTGAGCACGTGCATGGCCAA
CGCGGGCATGCTTACGTACGCCTTGCCGCAGTGGATGCACTTCTTCGCCGATTGCGAGTCGA
GGCTTCGGTGCGTCTGCTTGTGCCGCGACAGGTTCGACGAGGTCGCGTACTGCTTGCCGCAC
TCCGTGCACGTGAACTTGGGCCTGTCCGGCTGCGCCGCCTCCTTGTCCGGCACCGCGGCCCC
GTTCGTGTTCGCCGATCGACGCTTCGACCGTCCGTCCGACACGAAGAACGCCTCGTACGTG
TACGCCACCGTCTTGCTTCCGCTACTACCGCTCCCATCTCTCCCGCCAGTGCAGTTGGTCGC
GTTGGTCTCGGCGGTGGCCGGTGACTGGGGTTGAGGCGTTTGCTCTTGTTGGCGCAGTTGGT
TGGTCTGCGAGCTGGGCACGGGCACGAGCACGGAGACGGGGATGGGTTGAGCGGTCGGCG
GCGGCGGCGGCGGTTGTAGCTGTGCAGGTTGCAGCTGTCCGTTCGCCGCGACTCCTGGCGC
TGTCTGCTGAGGCGGTGGCGGCGGCGGAGGCGGCGGCGGCGGCGGCGACAAAGTGTG
AATGAAGACCGAGTGGGTGGGCGCAGCTGGTGACGCGCTCAGGTCGAGGATCGCCTGCGC
GGCGGACAGGTCCTCCTCGGCCGACGTGCGGATCTCGGACGTCGGCTGAAGGCTGTAGGGC
CGGTATATGTCCTTCTTCTTGGGGGGCAGCGAGAGCGCCGTCCCCGTGCTCGTGAAACACA
TGGACAGGCTTTTCCTCGGGGGCAGGCTCAACGACGTGGCCGACGACGGGGTCGTCGTCGG
GGCTGGGTGCGGTTGGTGCAGGCTCGACCCGGTCACCACTGCAGCCAGCTCCTGGGGGGCG
GCCGGGGGCGGAGGGGACGGTACCACGGGTCGGGCCAGCTCCGCGGCCGAGCGTAGCGTC
GCGTACTTCGGCGGCTCCTCGTGGTTGATCCACGACTCGTGCGTGTAACCATGATAGTAGAG
TGCACCGGAGGCGTTGAGCGGCGGTGGGCTAGGCGCGGTGGCACCAGCCGTGGGACTGTG
GGGTCCCCAACAAGTCTGCTGATGATCGACCTGCCTGGTGTCCGTGTCCCCGACCACGTCG
ATCTCCTCGTCCTCGACGGGCTCGTGCCTGTGCTGCCTCTTCTCCAACGACGCGGCTGTCTG
CTGCACGCCGCCGTGGCCCTCCTGCAGAACGCTCGACGGATCGTTCTCCTCCTCCTGCTGCG
GATCGTCCACGCGATCCGGCCACGGGTAGGCCTTCCACTTCTTGGCCATGAGGCAGCGCGG
CATGTTGCTGGCGTTTGGCGCCGATCACGCGGTGATTCTCCCACGAGAAATCCTCGACGTTG
TTGTTGTTGTTGTTGTTGTTGTTGTTA

29. >MSTRG.652.1 Gene=MSTRG.652
AAAAGAGCGGATGGACGAGAGGGGTCAACGAATAATTAACAAGATTGACCACGCTTTGGC
AATCTTCGGAAGTGAGCATTGTAATGTTATAGCATTGTAAAGTCAAACAGAATTGACAAGA
GCGACACACGCTGCTACAGAACGCCATGTAACACGCGAAGCGGGTAATGCGAACAAGGAT
GACGCCATGGGCAAAATGTGATTGCGTCGTGATGGGTGAAGGAGCGTGTGGAAATACGTA
GCAGGCTTCCAGGGTTCCGTAACCGTGGACTTTCGTCGAACACCGGAGGCTCCAAAGTTGC
GCATTCTGTCGGAAAGCTTTTAAGACAACGCGGGTGCTTTCGATTTCAGGACACAGGGGGC
ATTAACGACAAAAATTTCAGAAAATTATCACCGTCGGCGCTGTCATCAATATGGAGGACGC
AACGGAAGGGAATACTGTTCGTTCTGCTACGTGCTACAGATAACTTACCAATCTTTTGCGTT

TTGTTCGCGACCGGCGGCCACAAGTCTCTTCAAATGTTGCTTAAAATCGTCTTTCTTCACAC
GGAATCAAGTTTCCACGTTGTGCTAAGTTCGCTTGCTGCACGAAACCTCGAAATATTCGCTT
ACCTCGTAAAGTCGAGAATGTTCGTGTGAGGGCGTTGTGTAAGACCCGGAGGACAATATGG
CCGATCGAACTATGAATGTTCAAATGCTGCGCGTTGGTAGCTGCGCACTGGTACGCGTCAC
TCGTCATGTTTACACCGGACGACTGTCGTTTTAGAGCTGCTAATTCTTGAGCTGATGTATTCG
GATCTTTTAATGGAAGGCACATAACTAAAAATTTATCAACATGTAAAAGAGCACGTACATT
ATGTTCTTGTTGTACAACAACTGAAATTGAGCGTTGTTCTTGAGGTAATAAAATTCCTGAAC
TTGGTCGTACTCTGAACTTTCCAGGCGATGTTGTTTTTACCTAAATGATATAACAATTAAAA
ATTTAGATTATGTCTTATGTATACATATTTCTTAAATTTTCTTATAAAATAAAGATACATCAT
ACTTTATAAGATAAAGGTTTATCACTAGTCACATTTTTAAGTAAAATTGATCCACCAA

30. >MSTRG.670.4 Gene=MSTRG.670
TACCACGTAGGAGGCATAACACTCGCCGTCTCTACAATAGGATCCTAATATTTTCATAAATT
TTTTTGTTCTACGTTTCGGTGTTTTTATTTTTTTAACATTTTCCTCTTTTTATTATTCTTCAAGAA
ATGTGTCATCTATATCTTCATAAATTGTTACACAAGATCAATCGTTAAGAAAATGGTATTTT
GATAGACACATCGTGAAAGTGTTTGACCAACATTGTGATCGAACCGGAAAGACATTTTACA
AAATGTTGCAATATCACGTATGAAATTAATCGCGAACAATATCTATACACTTATGTTCTTTA
CTGTGGTGCCTAAATATTTTTTTAAAAGTAAAGAAAAACTAGTGCCTACATAACGCACGAT
AAATGCGATATATATATACGATACGATATATATATATATATATATGTACATATATATATCGT
AATTCGAAAATACAGATTGACTCGTCGAACTGAATCGCTCAATGTTCCATTCTATTCAGTTC
AGTGATATAATAATGTTTCTCGACGTATCAATAATATCAATAATTCTATCTCTTTAATATTTA
AGTTTTATCAATTGTGTGAGAAAACTTTTCTTTCTGAAAATAAAACTTTTAATCGCCGGAC

31. >MSTRG.678.2 Gene=MSTRG.678
GGTAGGATTCCGGGGGTTGAAAGCAGCTGAGAGCAGTGTACGAGTAGGTCATACCAGAGA
AGAGAGGTAGTTCGCGTTTAACAAACGACTCGTTCGTTGAACAAAGCGTATAATTGTTGGA
CCGCTTTTCTAAATTTTTCATTCGTATCTCTTTTTCAAGAATATCTCGTTTCCTAATATCGATC
TCCATTTTTTTTTATTAGTGTTACTATCATCGTCATCGTCGTTACTGTTTTCATCGTTGTCATCG
TTTTTGTAATATTTTCCATTCCATTTTTCGACGTTGCCGTTGTTGTTGTCGTTATTCTCATCGTT
CTGTGTTGTACTTACTCCTTGACGTGTCCTTGTTATTATTGTCCTTGTCCTTATTTGCGGGGAA
TATAATAGAAAATCGAGGAAAATGGATACTCCCATCGAAGAGCAATGGCTGAATATATTT
ATCATATAAGGAAGAATCGACATTGATTCGATCGATTGAAAACGGAACAATTTTTCTTCAC
AGTATAAATTGTCGCGGAATTGATTTCTCTTCAATCTTTTTTGTAAAAAAGGAAAAAAGGA
ATTAATGGTGGAAGAGAAAAACGAAAGAATATGAAAACATAAGAAGAATATAACGAAAA
ATATAAAAATAAAAGTGCCTCTAAACAGAAGTGGATTTCAAACG

32. >MSTRG.693.1 Gene=MSTRG.693
CTTATATAGAGAATCAGTCGTTTTGATCACTCGTATCCAACTATGCAACGATATCAGCGCAA
TATTTTTCGCGCATCAATCAGATCTAACGAAAAGAAATAAATTTGATCACTAGATGGCATA
ATACGTTATGTTCATCTTTTTTAATTTTAATCACAGATATAAGAGTCCTATAGGAGATACGAT
CTATATATATATATATAAAAAATTCTTATATCTAGTCTGTAATAGGATTTCTATATCTAATCT
GTGACTATAGATTGCTCAAACAAGATTTTTACCTACAATCAATATAGTAATTTATCAGTGTT
AAAATACAGATTCGACTATTACCGGTTGTACATAATATACATTATTGAAATCCTACATGAA

AAAAGGAATTGAACTTCTTCGTATTCAATTTCCGATGTTCTGATTTAAAGATCAACTATAAT
GTTAACAGTGTCATTAATTTCATTTAAACGTGTGTGATCAAGTTAATTAAAAATAAAGTGTA
AATAAACAACAAAATTCTTTGAAATATTTTAAGAGAGTACGAATGTTTTATTCGCTTTGATA
ACGCTACATTGCTTTGTCGTTTTTAACCTAAATCGAGATGGCTGATTCAGAACAAGATTTCG
GAGATCGTGGAGATAATGACAATTTAAAAACTGATAAATTATTTATCTTAAAGAAATGGAA
TGCTGTAGCTATGTGGAGTTGGGATGTGGAATGTGACACTTGTGCAATTTGTCGAGTTCAAG
TAATGGATGCATGTCTTCGATGTCAAGCGGAGAGCAAAAAAGATGATAGCCGACAAGACT
GTGTCGTCGTCTGGGGAGAATGCAATCATTCATTTCATTATTGTTGCATGTCACTTTGGGTGC
AACAGAATAATCGTTGTCCATTATGCCAGCAAGAATGGTCCATTCAACGAATGGGAAAATA
ACTAAATCAATCAAGCGAAACTTCAATACTTATTTTGTTTTCCTTTTTGTTTTCGTTTATTTTC
TGCTTATTTTTCCTTCTTTCATTTCTTTCTCCTTTCTCTCTCACATATACACACATATGCACACG
CACATACACATACACTCTCTCATTCACTCACTAAGTTGATTAATTTACTCATTTTCAAATTTG
CATTTTTCATGTTTTTTTCCTATTTTCCTGCTTTCTTTATTTTTCTGCAGTTTTTAAATTTTATGA
CTTAATATTTTTGGTCATAAAACAATGAATCAATAGTAATTGATCAAAACATCAATACAAT
AATATTATATTATATTCAATTTCAAATAAAACACGAAACTATTTATTTAAGAAAATATTTTC
CGCTTTAAGTTACCAGCAAGCGGGACAAAATGGTAGGCAGTGACAGAAGTAGGAGAAATG
GGCGAATGATGATGAAAAGCTAACTCATTATGATGCGATGTAACGGTTTGTCCGACGGATC
ACATGATATAATGACGTGATTATTGCTCTTCTATTCTTCTACTTTTTTTGCGTCATTGCCAATC
ATTTTGTCTCGTGACTCAAGTCGTTATTATATTATGAATTATTATATTCACGAATTAAATTGC
ATGTTTTTTTTTTGTTAAAAATGTTAAAAAATAAAGTATATTTCATATTATTAAATTATTAAA
TCATGTTACATCATTTTTGATAATCATCTATGCCG

33. >MSTRG.717.8 Gene=MSTRG.717
AGTAAATTAAAAAAAGACAAAAATCGATTTGAACCTCATGGCAACGAAAGGTCATTGACA
CCATTGACGTAACATGTATTCAAGATGAACCACGATTCAACGATTTGTCGTCAATATTTCTA
CTGTAGTCAACGTAGACATCGTATCAGAATATGTGAAAGGCAAAGAGATGTTGATAGAAA
TGAAGATAGAGGAGAGGACGAGGATGAGGATGCTAATCCATTGGGAAAATGGCCACATGC
CCTTAGTCCAGCTTTAGCATGTCTTAGTTGCACCCTTGGTCTTTTTAATATAAGTAGATTTTCT
ATTCTCAGTATACAATTTGGAGCAAATTTCATTGTACAATTTTTAATTTTATCTTTGATATTG
GGTATTCCACTTTTGACATTACATGTATGTCTCGGTCAAAGATTGGCGGCTGGATCTGTAAA
TATGTGGAAAATTTCACCTGTTTTTCAAGGTGTTGGAATAGCTCTTCTAATTGCACAAGCTTT
TATTGGTATTTATAGTGTTGTTGGCATATCTTGGATGTTCGTATACTTTAGAGATTCGTTCATA
ACGAAACAGGATAAGTATCGATGGGCAGAACCATTTTCTCTATACAGGGATGATAAATTTA
TACAAAATAATAGTAATTTGCATAAATTATATGAAACAGTACCAGATTATTTTAGCGGA

34. >MSTRG.723.1 Gene=MSTRG.723
AAAGATTTATGCACTAGTTTTTCTATGATATAATTGTTATTATTATACTTTTTTATATACTTTT
CTTTAAAAAAAAAAATTATTATAAAATTATCTTATTTTATTATTAAATTTTATAAAAAATAAAA
ATATTTTAGATTATTGATATATTTTAACACGAATTGTTGTTTACTCAAATCATTTCAAAGAAA
GGTTAGGTTAAGAAGTGGTTCTCTGAAAATTTTAATGTCGTTATATGAAAGGTTGAAAACAT
TTCCACAATCTTAAAGTTTCATCAGCTCCTGCACTAAGAATTGTAGTACCATCTGGAGACAT
CGCTAAGTGTAAAACACGACTAGAATGGCCTACCAAAGCAAAGAACACACTTGTGATTTG
GTATCTACCATATTTAAACATACACCT

35. >MSTRG.752.3 Gene=MSTRG.752

GGAACAGTAACACTCCTGTCGGATAGTCTGATGCTTCCCAGGTAAAAATCAGCGCATTATG
CGGCCATTTAACAGACCACATCATGTGTCCGGTACTTCACGTACAGCCTTTTAAACCGTGTC
GTCATCGAAAATTGTTTGTTTACTTTGTAAAACTAACTGTGGAAGTGAACTTGGGACTATCT
TTTTTCAACAAAACGAACGAAATTCTTTTACGTTTTCATCGCATAAATAACGATGAATTGAT
TTCTATTATCAACGGTAACTGAAGATCCACGTAAATGCGAAATGTCGCTGTTGGCTATGATT
ACAACGCGTGGACGACACACGATTCTTAAGAGACGAACAGAAACGAAACGTCGAATGAT
ACGTAATCGGTTTGAAGTGAGACGAAGCGGGAGG

36. >MSTRG.771.2 Gene=MSTRG.771

TTCCACGTCCGAAAACGCGCGCTCCAACGCACCCGATGCGAGTGAGCTTGGCGCGGGCTCT
CCCTGCTCTCTCTGCCGCGCTGCGCTGCGCCCAGCGTAAGCTGAACAGATCAAAAGTAAGT
GCGCGCGTGTGGTTCGTGTGTGTATACATGTGCCATCATCCGCGTGAGCGATAAGCGGGAG
AGAAGAGGACTCGGGCCAACAGAAGCCGGAACCAAAAAATGGATGCGACGTCACTGCGA
CTCATTTCCGGACTGCTTTTGTTCGCCTATACCGCCTCACACTTCAGTCACGCCAACAATGG
ACCCAATTATTACGGGAAATTTATCGGAAAACTGAAGAACTACGCGCACGGAATCAAGGG
GGACGTATACGCAGTGGACGACACGACGATATTCATAAAAGGATTCTGCTACGATGGGAC
CGGACCGGACGCGTATTTCTGGGTGGGGAACACCTCAACGCCCACGCCACAGGGATACAT
CGTCCCGTATCCGGAAATGGAGAAGATCAGGTAGGAAGAAAATTCTTTTCACTCCCCGAGA
GCCAATACTCTCGTCTCGTTATACGGTCTCCTCGTTATATCGTACCCTCTTTTCGAATTCCCC
ATTTCGTCGCCTCTCTTCTCACTTTTCGACTTCCCCCGAGAGAAGAGAAACGAAGAAAAAA
AAAAAAGAAAAGAAAAATGGCGGAGTTGCGTATGCGGTCAGGGTGATGGACGCTCGATGG
AAACGGCGAAGGGGACGGGAAAGGAGGAAGAGGGGGGCGGGGGTGGCTGTTGGACGTAA
GCGGCATAACCGTAAGGGATGAAATGAAACTCAAAGACTCTTGTTGTGCGCGTCGATGATG
GGGTGGCCGAAGAGGGTAGAAGAGGGGGATGGAAAGAGAGAACGGGATTGCGGGGGTGC
AGTTAATAAAACGGGAATTATACAGCGATCTCGTTCGGATTTCGTACGCGCGAGCACTTTA
TTACGTTTCGACTCGCCACTCTCTCTCTTTCATCCGTAGTGAAATTCGATGGAAGACGAACG
CGGCGAAAACAAATGGTCTAAGACTCTTCTTCCATCCCCCTCTGTTAAAAAGCGAGTCTTTT
TAAGAGAGACTCGAGCCGCGTACAATAATGATGTCTCGTTTCCGACTGGGGATCCAATTGG
GCGAAGATTATTAGAGGAGGATGACGGTATTAGAAGTATTTGTTCCTGTGTCCCACCAGGA
CAGCGTCATAGCTCCACGTCTCCACTGCTCGAATACGATCGATACATGGTTGTTTGCCAAAA
TCGTCGACGCGTATCACCCACCCCCTCCCTCCCTCCCCCCGACCAAATCGATCTAGCCAGGT
TGAAAAACTGGTCAGCGGGAAATGCGGTATTATTAGAATATAGTTATAGAGCACAATGTTA
TATAGTGCACGCCTATATACGCCTTCGAGCCT

37. >MSTRG.782.3 Gene=MSTRG.782

ATCGTATTTCGGTAGTTAATAATAACATGTCTTGGAAACATCGTATATACTCAACCAATCAG
GAGTTTCCAACCGAGAATCGCATAGCATAAAAAGTGGAAGACAGGAGCGCTGGAGCGCAT
TTTACGTTTTACAATTACGTTGAACGCGTCGTTGGAAGTTCTTAGTTTCGATTAGAAACAAT
GGCTCGTACCAAACAAACTGCTCGTAAATCAACTGGTGGAAAGGCGCCACGTAAACAATT
GGCCACTAAAGCTGCTCGTAAGAGTGCTCCTGCTACTGGTGGAGTGAAGAAACCTCATCGT
TACAGGCCTGGAACTGTAGCGCTCCGTGAAATTCGACGATATCAAAAAAGCACCGAACTTC

TAATTCGAAAATTACCATTCCAACGTTTGGTTCGTGAAATTGCTCAAGATTTCAAGACAGAT
TTAAGATTCCAAAGCTCTGCTGTCATGGCTCTTCAAGAAGCCAGCGAAGCTTATCTCGTTGG
TTTATTTGAAGATACCAACCTGTGTGCAATTCATGCTAAACGTGTTACGATCATGCCGAAAG
ACATTC

38. >MSTRG.795.2 Gene=MSTRG.795
TTCCTTAATTGTATGCAACGTTCTCGATACTCTTGCTACTCGATGCTGACTCGTCTCGTCCCA
TTCAAACGAGAGAGAGAGAGAGAGGGGGAGGGGATCGTTGAACGATGACTAACCGTTTCCTC
CTCCGTGATTGCTCCTCGTGTACGAATAAATTAGTAGCCGGGCTGCTAATGAGAAAATCTTG
GGCGGACAAAGGATTCGCAAAAGGATGCCTCGAAGCACGTACCTCGCACGTATGGTTGTG
TATTTATCGGCTGGATACAGTTTAAATCCCTTCCTTGGAGCCAGGCGTTGGAAAATTGAATT
CGCTTTAACTTAAAGGGACACGATTCTTCCTTCCACAATCCTTTCCTTCTCATCTTTTCCTCTC
GATCTTCCGTCTCATCCCCATACAATTACGAGAATATTATTTTTCCCACTCGAAATACGTAA
AAGATTCGACGAAAATTAACCATGAACGATTGAGAATGAAATATACAACGATTAATAATA
TAACCCTATCTGCTCGTTGGAACGGTCTATCCCGACCGAGGAGGAATACAGGCTGGTAACG
GATCGAACCGAATATACCAGCCAGAGCGCGCAGTGGTGGCGCGTGCGCGTATCATCCCTG
AATCGCGTTGAACCAATGAAACTCAACCTCTTCCACTGGCCTCGGGGGATAGAACATTTCG
ATATATATTTCGGGTAATTTCCATCCACCGCGATGTGATCCCATAAAATCGATCGTCGGAGC
GAAAGAGGAACGGGGCGCGTGACAGGGAACGGTGTTGTCCAGCCAGTGACCGATCGTCTC
CGTTGTCCAGGGTATGTTGACGAGAGGGCACGATCATCAGCTACGAGGGAAAACTGTGCGT
GGATCAGTGTTACCCGGGCGGGGTGGTGGTGGTGGTGGTAGTAAACGGTGGCTCAACACTG
GCCGCCATCTGCTGACGGTCGCTTTAAGCTCGAGCCTTCACTGGTTCCCCGTGAAGTGCATA
GCCGAAACGAGACGTTCGGCTCCGAAACGGCACAAGCACGAGCTTATTTCGAGGGAATC
GAGGGTCGAATCCTCGTCCGTGACACTATCGACGCGCGAACTTGCCGCGACGCGGGTTCTC
GCAAGTGTGTTGTTTCCGTGGTTATCGTGGTGTGTTATTTCTGCCGGAATCGAAGGGGCGAA
CGATCAACGTATCAACGAGCGTTCCAGGATGACGGAGACGACAATGACGTAAAGCCCACG
AGGCCAAGTTGAGCGGAGAGAGATCCTGGTGGCGGCTCGACTCTGTTTCCAACATCATCCG
ATCCCGATCCCCGGCCAAAATCTCTAATCCTGGAGCGATTATCTTCGATTATCTCGACACGA
CTTGAAAAATCATGGGAATTTTCGGACTTGATCGCCAATCTCGATGCTCGGATCCGGACAA
GGAATGATTGGACGAGTGATGGATCGAGGGAATATTACTCGAACGTACGATTATGATAATT
TTAATAACCAGTGATCACTCGTGCAAATATTCGAGGGGAAAGCTAAGCTTGCTCGACGATT
GAACTCTCATTTCGAATTTCCAACAACAGGGATCTTATTTCGTGTCAGCGTTTCTTTCTTTTTT
TTTTTTTCCTCCCCAAAGACACGGTTGAAACTGAAAACGTGATATTGTGGAAAAAGAAACG
TGATTCGAAGCGTTTAGGAAACGTGAGGATAATTCGTTTCGCGAGTGTGTCCGATGAAGGA
AACCGATAGAAGGGTGGACAATCGGGACGAGGCGAAATGTGAAGGAACGGAAACGCGG
GTATCGAATTGTTTCGCTGCGTTGCACGACGTCGTGTTGAACGACGACGAGGCTGGTTGTTA
ACGGAATATCGACCGAAGACCGTAGCTACCGAATTAGTCGTGGCGAAGGTCGCGCATAAA
CGCGTGCAACCTCGTTCCCCGAGATTTGGATCACCCACCTCGAACAGTTCGATCTGTCATCT
CTCCAATCTGTCCAACGATCCGTCATCAAATGTTGTATACATACAAACAATTGCCAACAAA
CGGAACCCTCGGCAAATCGATCGACCAGCCATCGATAACACGGAATAACCAGTTTCCGAT
CAATCGTTCGTGCAAAGTGTTTTCCTTTTCCGTTTTTCGTTTCTTCTTCTATTTCTAAAAAACTT
GTGTGTCAATTGTGTCTTGTATATACATGTATATGTACGTGTATATATAAATATATATACATA
TATATATATATATCCCTTTCTCCAGTGACGCATATATAATACGTTTTAAACGAGACTAAAAA

AAGAAAGAAAAAGAAACTGACCTACAGACTCGTTCGTATGGGTCGATAATTAGTGTATCC
AGATTAACGGGGCAGAGACATTGAAACTGAAAGTGCGAACTGGATGAAACTCCCTCGAAG
AACAGTTCTCTGAACGAGACCTACTGACCTATCGACTGAGTGCCCCACCTCTCCCACCACC
TCGCCACCGCGCATTTTTCTTCCCCATTTTCCTCCTCTGTTTCCTTCTTTTTTTTTTTCTTTTTTTT
TTTTTTCCTCTCATTTCCCTTCCTTACTCTTGTATTTCCTCCGTGTAACCAATTTCGAGTCGAA
ATCGTGGTTGGAGGACACCGACCGAGGTGGAGGAAGCGGTATTAGAAGGCAGGAGGGCG
ACGTGGCTGCGCGTACGAACGAGCGCGGGTGAATCGAAGAGAGTCGTTCTTTGAACGAGA
CCCACTGACCTATCGACTCGTCGCTCCACTCCTCCCCGCCCGTTTTTCTTTCCACGCCACCCT
CGCTTCAGCTTTTCTACCGCCCTGCGTTTGTTGGCGTGCCGCGTCGGCCAATTCGTAATTCCA
TTTCGTGTCTTACCCCCTGGACAGGGGTAACGTTCGAGAGGGTCGATATTATATATACGTAC
ATTCACATATATATATATAATATAAATACTAAACGCGTATATAAAGCGACAAACGAGTGCC
TAAAAGTATAATATTTTCAATTCGGTACTTCTGACTGATCTATATCGTAATTATTTCATCGAG
AGACTCTCTCTCTACCCCCTTTTCAATTCTGTTTTCCTCTGGAAATTTTATTTTTTACATTCCTT
TTTCTTATTATATGTAACAATAGCAAGCTGTATTTTATATATATACACGTGTATCAATCTTTTT
CTTATTTCTCCTATAGTTTCGATTAAGCAAAATTTTATTGATTGAGGGTATCGATGATGATAC
AAGGGTGAAGAGTGCCTAAAAATTTTATGCCTTTCTCGACTGATTATCTAATTGATTATATC
GACACGCGATATTATTTCGTACGTGAAATCGTTACTGCAATCCTCGTTCTTCTCTTCGCAAAT
TTTTATCTCATAACCCAATTATTTCCACTTTCTTTCTCCCTCCCCTCTTATCGCGAAAGAGATC
GTGAAACGGTCTGAACTCTTCCTCATCGGAAAGTAACGAAGGTCGACAACTACGCTACCGC
GATCGATACGATAAACAGTGCCTCGAGATTATTTCTCCACTCTCGACTGATCGACGAAGAA
ACATATACATCGTTACCTCGTAAGCTTCCCTCATTTCTCGCCGTTTTTCGCTTGGGCGTGTCC
TATATTTTCATCCCGTTCCTTTTCGTTTTCAAAAGGGGACAAGACTCGAAAAACAGTGATCG
AAAAAATATATATATATACATTCGACGAAGAAGAAGAAAGAAACTCTTCCATTCGAAATTT
TTATCAAAACATCCGTGTCTCGAGATCCGAGGAAAATCATTAATTAACGATCGAAATTTTTA
TCCAAAGATTATCGTGTCTCGACCTACAACTCGAAGAGATACGAAAAATCGATCGACGATG
GTCTCTCCACGCCTTGCATCCCCGATGCAACGGCAACGGATTCGCCACTGATCCGATCTCA
ACCAACCTAATCGTCCAACTTTTACCGGGTCGACATCACGATCGATCGGATGCACACACGG
AGTTGGCGGCCCTTGACAGTGCATCTTTAATCTCGTTCGCGGAAGAGATGGCATCCGGATC
GCGATTCGAAGCCGTGCCAACGTCCACCGACGTGAAACGGAATTACGTGGCAACCTTCGG
GGAATTTTCTCTTTGTAAGAGCGCGACGTGCTGGTTCAGGCCGTTGACAATAGCCTGATCGT
AAAACGTGTGGGGTGGCGCGATAAGAGAGAGAGAGAGAGAGAGAGAGGGAAAGTCATCA
GCAATAAAGTATCGTAAGAAGGAATCAAAAGCGAAGGAAGGAATAAGAAGCGAGCCAAG
GTCGAGCGTTCTATCCACGAAGAGGATAACGATAATAAAAGACATTTCGCGTGTAAAACG
GGAAATATTACATCTCGAGATATATGAGAAATAGAAAAAGAAAGAAGAAGAAGAGAAAA
AAAAGGAAAGAAGAGAGAACAGTTCTGTGATCGAACGGCGTGAAAAATAATTCGTTGAAA
GAGTGCGCAGGGACAACAAATTTTTTTTCTTCTCTGCGGAACGATATCCGAAAAACGTGTC
CGATCCGATATCTCTACTCGCCTCGTCGCGGAAACCGTGTGACGATCGAATCGCGTTTTTCG
TTTCCCCGAGCGGTGAGAGAAAATGTCGCGGTTCATGGCGATTTGAAAAGAGAAGGAAAA
AAGAGAAGGAGAAAAAAGAGACACAGGGTACCGTGAAATTGGAAAGGGATAGAGAGAG
GGGAAAAAAAAATGGCGGAAAGCGCGGCGAGCCCGGGTACCGTGCAGGTGGCCGCGAAT
AGCCCGCAGCAACAGCAGCAACAGTCGCAACAACAGCAACAAACGCAACAGAATCAACA
ACCGCCGGATATAAGCAGCTCGCAGTTGGCGTCTCCACCGATCACGGGCACAGGTAAGTTT
CCTTGTCCTCCCCGATTTCCAAGAAGGTGTTCTTCACACAAGCGCAAGAACACTTTTCTCCG

GTGAGTACTTTCACGGAATCAACGAAAATAGCACCGATACATCTCGTTAAAGTTTCCCCCC
CGCCCCCCCCCGTTGGCGGTGTCGTCGACGAACTTTGCATCCTATCCGCACGACGGGATAA
AAATGTATAATTCAGGGGAGCGGATAAACAGCGGTGTTACAAGTGATCGAAGCCAGCTGG
CAGTCCGGTCACGAATTTATTTCACAACTATGTAACTTTCCTAACGCTTGGAACTGAGTAGC
AAAACGCGAAGGATTGCGATCCCCGGGATTTATTTGGAATCCGAACATGCGTACAATGATG
GCTGTCGTCGCGTTGTTGTAACAATGTCTTCGTTAACTTGGAACGGAGCACACTCGGTGTAC
ACTTTTCCTTCACTCCTAGGAGAGGCCTAGAAGAGAAATTCAGAACCCGAGGTTTATTCGG
AATTTACGATTTTTTTTACATTTCGAAAATATGTAAACATAGCGAAATAAAAGGG

39. >MSTRG.821.1 Gene=MSTRG.821
CGCGCCGAACGTCGAACCGAATACATGGGGGTGTTATACGTTTTTCACATTAAATTCTTGT
ACGAAATAACGTTATATTTCACGCGCTGATCATCGAAACACAACATCATCGTTGGGAAAAT
TCGTTAGTGATTATCAATAAAAGAGTGACGTTCGATATTATCGACCATTTTAGTGCAGTGTC
GTAACGAAAGTTCTACTTTTTTTTCGCGTACTTTTTTCCGTAAACTCGCTTCGATTCGATTGTC
GTCGATGATCGTGCACAACGAAAAGGAAAACCTTCTTCAATTACGTGATCTATCTTTATTAC
TAAGCACAATTGACAGCATGCATTTGAGTGATGATGGATAGCATTTCTCCGGTGAATGCAT
ACCAGTGAAACGTTCTGTTCGCGAGACCACGTGATCAGTGTTGGGAGGTTATGTAAACGAC
GCATTTACAAAGCAGCGATATTATAATCTGTCAGACATCGATATTTGTGCATTGCGTATAAC
CACGTTGTTGATTGGCTGTAATTTCGATATCTCGAATAATCGATGAGAATAATTGCCTGTAC
ATATAACGCCTGATCTGTTTTCGAGTTCAATCGATACTAATTAATCGATATTAGTACGTTAA
ACGGTCAATGTGCCGAAAACGTTTCAAATTTAGTTTAATATGCGCAACACGGAGAAACGGA
CCGGTTCAATGGCGTCGATTTGACTTCGTGAAATTCGTGTTTGTCCTTCGAAGCATAAAGTC
CTCGTGTTCACATGCCACACCTGTGTTTTTACTTTTTTTCGAAAGTGTTTCACGGTTAATCTAC
ACTCTTCGATGAACAAGGGCGTATGATATGTTAATGTTTGTGTCGCGTTCACTTAAATCTTGT
GTGTTCTGTGTGTGCCAGATGTCAAGACGATAATTGGTGCTGTCTTAAGGACGAGTGATTCA
TGTCACCATGCTGACGATTGTTTCCTGCTCGTTCTGGGCCAACTGGTGGTACAGGCGGACT
ATTTTGAGAATATTTCAAAAGAGGAATAATGGAATATAAACGTCATGGTAACAGAGAATAT
TCGAGACGAGGAATAATGTAAAAGCGTCATCAACTAGTGCACATCGTTCAGTGAATCGTTC
TCACTTGCACAGCCGACGAATCGACGAGGAGGAGGGTGGTGAAGAGGGAGGGGTGTTTGA
CGGGCGTTTTCAGAGGCAAAGGGATAGAAAGGAAAACGAATGGGGGAAATATTAAGCCG
GCAAAGACGAAAGTCTCCTTGGCTGATTTGACATTTCCGTGTGCGTGTGTGCTCGCCGAGGT
CCGTGTTTTCATCGCTTCGAAACGGAAGCTTCGGGAAGAAGAGAAATGGGAATATTGGGA
AAATTGCTTTCGTCGGTGTCCCTGGTGTCCATCTGGTGTCTGCTGATCGTCGAGCCCGGCAA
GGTTCAGAGGGAACAGGATCATCTGGGTAAGTCACGAAGAATCAATTTGGATTGAAAATA
ATTTCTACGCATCTTCTTCTTCTCATAGGTATTTATTATAATTATAATTATAATTGTTTTAAAG
AATATAATCGATATTAGATGACAGAATATTTTTACAAATTTATCATTAATTTTATTATTATTA
CAAATATACAACAAATAATAATTTTGATCATTTCTCTTTTTAAAAAAGTCATACGGAAAAA
ATTTTCTACGCAATATTGTACTTCTTTTTAAAATTTTTAAATTTCACGAGATATATTTTTGGCC
AGTAATGTATCCAATGCATCATCTTATTCCAACCAACGTGCAACGAATCTCCCTCGACCCTG
GTTACGTCCAACGAGCACGAAAACTCGCGCCAGAAAATAATCCTCCTCGTAACGTTGAAA
AGAATTTTTCTCCCTCTTAAACCTTTCAAAACGTTACCAGTAAACAGCGTGTACGAAAAAGT
GGCTCGAGCTTGGCGAGAGATAGCCAAGTATTGTTTTTAAATCCGCACGCGTACACCCGC

AAGTACTCGCGTGTGAATCGAGGGCAAAGTATGCGGTCGGTGCAGAAATAGCTCACGTTTA
TCGATGCTTACGCGTTGGAACTTCTTTCCGCCCCCTTCTACATACGATGCATCTTGCGAGGA
GGACAATAAAACGCGGAGGGATGAAAGACGAGCGCGGATTTAAGGAATGTCGAGCGTCTT
GAAAGTTCCTCGAGCGTCGAGCGGAATATTGAGCAGCTTATGGGCCGTATCGCAATTATCA
ATCGATTCGAGTGGATGAGGATTCTCGAGCGGAGACGAGTGCAGTTTTGAAAAGGATGAAT
CGATTCGAACGGGGGTATTAACGGGAGATGGCATGTTGTCGCGGATTTGTTTTTTAATTGGT
TTGCTTTATTTTCTTTTTTTCTTCTTTGAATATGATGCGAAACTCGAATCTGTTTTTGTAAATA
GACATAATTATTGATGTTAATTTTTTTTTATATATAATTCGTTTTGAATCTCGTTAGAATAAA
T

40. >MSTRG.835.1 Gene=MSTRG.835
CGTTTGAACGAGGCCTTATAGCGTATTCCGGCGTCTCCGGTATTCCGCGTGCACAACGGATC
AGAACACGTTTCTTCGATTGTGACGAAGGGGGGAAAAGTTTAGCGAGGAAATTGGGATTGT
TGGAGACCGTGGAGGACCTTCGTGAAAGGGGATGACTCGAGTGGATTCAAGTTTCTGTGAA
GGAGATTGTAATATTTCTGTTAATAAGTTTTGAATGAAGATTGCGGGAAAAGAACATGATA
AATAAAAGAAGAAAAGTGAGAAACGTTATCATCATCAACGAGATATCTAAATGCATTTCC
AAGAGGAAAATCAAAGAAGACGCCTATGTGACGCTAGTCGTCTCAAGAATTATTGATTAA
AACGAGAA

41. >MSTRG.847.1 Gene=MSTRG.847
ATATATGATACACATTGATATATACATACATTGAACATTAGATTTCATAAGCAATGTTATCA
TAAATTACTTCTTAAATTACTACTGATAGAAATTTTCCAGTACACTAGTAAAGTAAGCGACA
CTTCTTAGCAAAAAACAAATGTCTACATCAACAGATGACAAAACCAGCCTAAAAAATGGC
GAAGAATTAAAAGAAAAATTTGATCCTACTGGTTAGAATTTTCGTTCTTATGAAGAAATTAC
TTCAAATTTAGTTCTATGTTTCATCTATTAGATCAATATTTTATAGAACATCAACATATTCGA
TACAATCCTTTAAAGGGAGAATGGGTATTAGTATCACCACATCGAATGAAACGACCATGGG
GAGGACAAATTGAATCAAATGTAGAAGAAGATCTTCCAGATTATGACCCTAATAATCCACT
TTGTCCAGGCAATATTAGAGCTAGTGGACAGATAACTCCAATATATGAAAATACATATTCA
TTTATTAATGACTTTCCTGCATTATTGGAATCAGTTCCCAGTCCATCAAAATCAGAAGATGA
ATTATTTCAAATGGATGCTGCTAGAGGTATTTGTAAAGTAATGTGTTTTCATCCAAAATCAA
ATGTAACAATAGCACTTATGAAAATTTCTGAAATTAAAGAAGTCATTAAAAGATGGATTTT
TGAAATGCTTGAGTTGGGAGAAAAATGGACTTGGGTACAAATATTTGAAAATCGTGGTGCT
TTAATGGGATGTAGCAATGCTCATCCTCATTGTCAAATTTGGGCTAGCTCTTTTTTACCAAAT
GAAGCTAAAATAAAGGATAGATTTCTCAGTGATTATTATAATCGCAATAAGAAACCACTTC
TTATAGATTATGTGCAAAAAGAGCTTTTTAAAAAGGAACGCATTGTAT
TAGATAATAGAGATTGGGTAG

42. >MSTRG.862.1 Gene=MSTRG.862
AAAACATTAATCCTTGTGCCCACCCACCAAAATGGCTCCTTTATGGGGCAATTCTAGGTGTT
TGACGTGAAGTTATTTACACACCTGGAATTAAGCCTTCATTGAGATAAGTTGTAGATGTCTT
TCTGTGCACCGTGTGCGTGAAACAGATCAAGAAAGAGTGCGTGTTCTATAGCAGTGAAGAA
TCGATCGAGTTCCTGAAGAAAAAATTATACAGGACGGCCGGTTGGTGTAATAGTTCGCTTG
CTGAACAAAAATCTGTACAGATATACAGTGCCATTCCAAAGAATTATTGCTCACACTTGTG

CTAAATTATGAATGTTGTGCTTTATATAAAGAAATAAAGTGCAAAAATTTCTTAAAATCTAT
ATTATTAATTTCTCTAATGATGTAATTTTATCATTTATAAAGTTATAATAATAATTATATTAA
GCTC

43. >MSTRG.887.2 Gene=MSTRG.887
CGGAAATATACCACAGATAAAAAGAGTGTGCGTCATTAGGTATTTCCAGATATAAGATGA
AGTATATGGTAGGAAGTATATTCAAACGTTGAAAGATGGCGTAACAGGAATGAATCAATA
TGTAAAAATTAGAAATATAAAATCGACAGTGGTGAGGAAACAACTTGAGACGAAGGATTG
TGTACCTTCGGTGATCGTCTCTTTCATCGGCAAGTTTTGCTCACGACGAATACGAAGCAAAG
CCTTGGTGTAGAGATGTATCTGACTCCTGCCACTCTTTATGCTCTCAGATTATCATTGCAACA
GGGAACCGTTTCGTCTGTATTTCGTAGGAATATGGTCGAACGTAATATGACCAAATCGGCG
TATATCCTTGCGACTGTTGGCCTGTCTATGAGTATATTTTCTGTGAGAAAAATGTTAACCTCT
AAACACGGACGTCATTTGTAACAAGTTTATTAAAATCAGCAGCAACAAATACAGTTCTAAA
AAGAAAAGAAACGAAAAGAAAATAGATAAAAAAGAAAGAAGAAAAAAATAGGATTCAT
CATATGTGAATCAATTATTATTGGCAAAGGAAATTTTTCATCGAATTTTAGCATCTTATTCAC
GCCGAAATGTATACTTATTTTTTATCGGAAATCAGTTGTCTTGTATTATTGAACTAATCTTTT
ATAATCGATCTATTATCGCCTCCAAAATATTGAACATTTCCTAATGTAGCGTATTAATTATA
GTAAAC

44. >MSTRG.909.2 Gene=MSTRG.909
TGAACATCGAAGCATTCTATTGGTTATTTCTTATAGTAGTGCTATGTTATTAAATCGATAAA
GTTAGAAACAATATCGAAAAAGTTAAAAAACTTTAAAGTCTCTAAAACGTAGAAGAAATG
TATTAAATGATAAATTTATTTTGTGTCTCATTAATTTGAAATTTTAATGAAACAGATTAATTG
ATAATAAAAATACTATTGATTTACTTATTTGCTTACAGTGATTATGAAGATGAAATTTGATC
AGCAGAAAGCTTTAAAACATATACAAGAAGTGGAAGAAAAAGCAGGTGAGATTCTTACTG
ATCGACAAGAAATAATTGCTTTGGATAAAAGAAGAAATAATGATAGAGTTGGAATGAGGG
CATTGCAGAAAGAGAATTGCGAGAAAATTTGGTTAACTATTGGACCATTATTACTTAAAAT
GCCATCCAAAATTGCAGAAGAATTACTTATTAAAGATCAAAAAGAATGTGACATAGAAAT
TAATAAATTAAGAAGTAATTTAAAAGTTAAAGTGAATGAATTAAGAGATTTAGAACTTAAT
CCATCTGTTCCAGGTTTAATGTTGCAACCTATGTCTTATAAAGAAATGTCAGCAATAAAACA
AAATTTGGGTCA

45. >MSTRG.913.1 Gene=MSTRG.913
TTGGACGATGAAAAATTGATGAACGTATTCTTTTATTTCGCTTTCCAATTGGTCGAGTCAGA
ATTGTTGAAAAAAAATTATTTTCCAAAAATAAATCAAGAATATTGGAAAAAATTAAATTGA
GGACGCAATTTCGATCAACCCGCTTGAGTATTAGTTTAAATAGTTTGAATATCGAAAAAAG
AATATTATAATATTCTAGAATACAATATATATTATATTGTTTAAAAAAAATAAATGACATCT
TGTTATCAGGATGACCACGATCGAAGCGTCAGCTCATGAATTGAAAAAGAGAAAAAAGAG
AGAAAGAGAGAGACGAGAGGATGCACGATTCGCGAACAATGGATCGGATATGCGGGTCA
ACGATGCCAGCCAACGTTGTGCCCAATTTTTCCTTATACTTAACTTTGACCGAGAGAGTCCT
CACGGCACGGCGTAGATGGAAAAGTCGGGCGGTGGGTTATCGATCATTAAGAACCAGATA
TATATGCATTATTCCGTACCAAGTACGGCTAGAGCTATCGAGTAATTTAATATAGAATTAAA
ACAGTCAACTGTATCGCGATTAACGCGCGCGCGCGCGCGCGGAGCACGAACGAAGAAAGG
CCTGCATCGACTATCTTTCCTCCACACTTTTAACCTGATTTCCCGTTCCTTCCGAAAAAAAAA

AATCCAATTCCCGTTATTCTAATATTCCTGCCTCCCTCTAACCCTCAACAAAAACAAAACAA
CATATTGTAATCTATTGTAGATTGTTGTAGATAAATTATACGGTTTGACCGTACATTGTTACT
TTTTGTCGACAATATAAAATACACAGATATATAAGGAGAAAAAAAAAAAAAAAGAAACT
GAAAGACCGCAAAACCATTTGACGATCCGTAATTTGTGGGATGGGC

46. >MSTRG.944.1 Gene=MSTRG.944
TTATTATATTATTCTATACGTTCTATATATATATATATATACTATGTGATCTAACTTGTGCATG
TTAAATCGCAGTTTTAATATCGAACAATTAACGGTTATTGCATATTTGAAATACAATTGGAT
TTTATAGTATAAATTTAGTAGTTACATTTATAATTTTTATTAGAAAGTAAAGTTATCTCATAC
TTATATTTTCATTAAAAATAATTAAAATTGTGAATTAAAATTTTATATTTACGTAAAATATAA
TTATAAGAATTATATTATAATTATTATATTTATAATGGCATTTTCTCGTATACCTCGTCGTGA
AGGTTATATAGTTGCAATATTTGGTGCTATCACTGGTTATTATATTTGGAACCAGCCATTGA
AACAATTTGCTGAAAAGAATTTTTCCAATACAGACTTAAATAAAGATATGTACCATGATCA
AAAATAAATTTTTTATTTTGCATCCTATAATCAAAATAAGTTGTATAACAGTTGGTATTCTAG
GTTGTTTTGCTACTTATAAATTTTATTTCAAACAATATGTTGATTTTGTTGCAAAAAAGCAAG
CTGAAGAAGAAGCAAATATATTATATGAAAAA

47. >MSTRG.950.3 Gene=MSTRG.950
CATCTACTGACTTATGTGTTGTTACCGGTGTGATATATACTATTAACAGGTTAGTCGGTATTT
AACATCAATGAGATTATCTTTATATTTACATTCTCTAAGTTTTTATTACATTAAAATATCATT
TAATTTTTTATTTAAAGAATATTTTATTTTATTCGTTATTCTGTGCTGAAAAATATTTAAGATG
GCTTTAAAAATTTCTGTTAATACTGGAAATATTGATTTGGATAATAAAATCAATGAATGGTT
GAAATGGAACAAGGATGTAATTCATGAACATAAAGTTCAAGAACTCATCCTTAATAATAAT
AAGAAAGTATTATCTTCCTTATTTTTAAAAAGACTTGAATTTGGTACAGCTGGTTTAAGAGG
TCGTATGGGTCCAGGATATAATCAAATAAATGATTTAGTTATAATTCAAACTGGTCAAGGTT
TATCAATGTATTTACTTGATACCATAGTTGATGCAACTGAAAAAGGCATCGTAATAGGATAT
GATGGACGTCATTGTAGTAAAAGATTTGCAGAATTAACTGCTGCAATTTTCATGGCAAAAA
ATATAAAAGTCTATCTTTTTTCAAAAGTTGTACCAACTCCATTTATACCTTATAGTATATTGA
AATATAAATGTGCTGCTGGAGTAATGATTACAGCATCTCATAATCCTAAAAATGACAATGG
TTATAAAGTTTATTGGGAGAATGGTGCACAAATAATATCACCACATGATAAAGAAATTCAA
AAATATATATTTAATCATTTGGAACCAATGGAATCTTCATGGAATATAACAAAAATTTATG
AAAATTCTTTATATCTAAATCCATGGG

48. >MSTRG.955.9 Gene=MSTRG.955
GGCGAAGGTCGTGGTGGTGGTGGTGGGTGGGCCGTGGAGAGAGGGTGGCCGGGATGGTGA
TATAAAACCGGCGCACATGATGGGGATCTGGATCCGATAGGGAACTGGTTGCGACAACGG
TCCGGTCCTACCGACACGCCAAGAAGCGGAGGCTCGGTGAACGAGGCCGGTGTTTTCACCC
TTCGTAGCAGGCAGGAGGGTGGACGGCGTGCTAGAAGAACCACGACGAGCACCACCAGC
ACAACGGCGTCCAGCTCGACGTTGATTTCTCTGTCGGACGAGGTGGCGCAGCCCGCGGCCC
TTTTAGCCCCCGAAGAGGACTTATGGTCTACCAGTTCTTCCGCTACAACCAATGTTCCAGTC
ACCTTAGATACAGCTGCCGCTTCGTCAAAGCCACCCTCGGAAATGGTGGTGAAACCTCACA
GCAAGGTCATACTACCGTGCGACCTGGAGGAAAATTACTCGAGGCTGCTGTTGCCCGGGGT
CAGGAGCTACAGAATACGGCCAGCGACATGGTTGCACGAGGGAAGTCCTGTAGATATGAT

TACGATCAACACGAAGACGGAGATGAGCGGGACCGGACATAGATATATCGGCGACTCCAT
GACTGACGCGTTGCACATAGACAACGTGAGATTAGAGGACGACGGTATATGGGGTTGCAG
GCTCGAGGACGATCAAGGGAAGATACTTTCCGGCAGGCCTGTGAAACTCGTCGTCCTCGG

49. >MSTRG.997.15 Gene=MSTRG.997
CGAGGTTAGGATAATAGATGTTTCTCAGCCTCAAGACTTATTTAAAAGTGGTTTTCTCGATG
ACTTTCCATGATGATAACATTGATGGCAGTTTTTTCGCATCTAATGGTAGTGGAATGTACAA
CTCGGATGCAGAAGCGTGGACATTTTTGTATAGATTATCTTTCGTGTAACGTTATCAGTTCG
CAATGGAATGGAATAGAATAGTAAACGCTCTCGAAAACAGAAACAATTTTTCAACATGAA
CATCGGAGCTACGAGTTATCCAGCCGGTCCGCGCTTCCAGACAGACGTATAGATCGCGGCC
CATAAATAACAAGGAAGGGAAGGGCCCTCGTCTTTAATACGATCCCCGTTTCCCGAACTCA
TAACCGTTGATCTTCCGCTGGAAATTCACGAGGAAAATGCGACAAAAGTTCACCGCGCAA
GAAGATGCACACGATGTAACATTCAACAAACACCGGTAGACAATGTTCCAAACTATACAA
CATACATTTTGACAAGTTCCAGTTTCGCTTCGCAAGCAGCCGGAAATTGAACACCGGCTGT
GTTTCCTGTATCCACGGCGGCGAGCCAGATTCCGCGATGGTAGTTTTACCGCGCTTTCAAGA
CAAACCCTGAGACGATGAAACGGATTTACACGATAAAATTATCAAGAAAGAAAATAAGA
AGATCGAAATTGAAAAATTCATCACATATCGAATATATACACGTTCATGGATCATCTTTGA
AAGGATTCTAGAAGTTGATTGAAGAGGAGTTATACAGTATCGACGTCAGCGACAATGCTAG
GCCGAAAGTTGTAGCGGGCGATGTCGGCAAATTGGTGAAAGGGTTGGCTTCAAACTGAAAT
TGAGAGGGGTTGGACGTCGCCGGTACCCAACATCAGAAGGGCAGAGGCGGGATACGACGC
TTCTGGGCTGTCGTGAACCCTGGGCTGGAGCCACCGGTGTACTTGGCAGAGAGTGCTTTTCA
TCTCCGTAAATGGACCGGCGGAGAAGAAATCCAGCCGAAACGAAGTGCCTGGGGAGAGTT
GACGAATTTATCGATTCGTGTACGGGGTAAAAATTGCACGCGTGCACACGAGAGATTTGAA
ACAGAACAGGCAATCCATACTTCCAACTATCTCAGAAAGTGTCGCAAAAATCAATCGAAT
AGATGCGTAGCGAATTCATTTATCACAGAGTGAAATTTAGTTACGGATTCTCATCGAGGCA
AAGAAAAAGACGGCTAGACACGACTAGAGACAGAGAGAGAGAGAGTAACTTTCCTCCGC
GCGCTTTGCGGCCGTGAAGAAGGGGGTTGTGCCGCTTTTGTCGTGCTGCACAGACGCGTGG
AAAAGAAGAGGCCGATGGGCAGCCTCTTTAAAGAGTAATTACGCCTACACTCGATGTATCC
TGCGCCCCGTGATATTCTTCATCGTTTTTTTTTCAGGTGAATACGTGTTTGTTTGCCATTGTTC
CATCCTCGAGGAGCAGCCACTTGAAGTCCCATACGCGTCGAATCCTTTAGAAAACTGCGAC
GAGAGGCGCGGGAGGATCGAAGAGACGAGACTTCATACGAGACTTCGTTTGACAGCCCAC
TGGTCACCGAAGCGATTTCGCTGCAGAAAAATGGAAGCTCGTTAACGGGCGAGGAAAATG
GTTGCTTCGACTTGGTGTTTCGTGGGTCGAAGGTCGTTTTGTGAATCGGGCGGAGGAAGAGG
AGCGAGGACGGAGGGAACGGCGGATGCGGACTTTGCGGCCGGTGCACAATACAGT

50. >MSTRG.1001.1 Gene=MSTRG.1001
ATTGACTAAGATAATGATTAAGTTCCGCTAATTCACCAGACATTTCTAAATATCTATGATAT
TTATTTAATATTTTTGTAGGCAGTGTTAAAACGAATATTTGACCAATATCTGTAAAATTTTTA
AATTTATCATCGAAGAAAGTGGTTAGAAACAGCAACGATGAGTAACGATCAAGCAAACGA
ATTGTCTGAAACAGTTGAGTTATCTTCCAATGACATTGAGAAAATAGAAGAAGCAAAATTA
AGAGCAAAATTTCCAAATACTGGACGTCCAATTAGTGGACATTCAGCATTCCTACAAAAGA
GGCTAGCTAAAGGGCAAAAATATTTGATTCTGGAGATTATCAAATGGCAAAACAAAAAT
ATACAGCTAAGCCTAAGCCAGCTGGTGTTTTGCCAACAGGTGATGCTATACCTACACCTGA

AACAGTACCACAACGAAAAACATCTATAATTCAACAAAAATTTAATACAACAACTACATC
ATAAATTTTTGTACTGTTTTAAAAGTATTGCAACTCAAATGATATAATGTGGTTATATGCAA
TCTGAATTTTAATTTTAATTCAATATCTTTATATTTTATAGACGGTATTATTTTGAAACTTCAA
CTTTTATGTTTTTTGAATGCTTATACATTATCAATATATTGAAAAAGATTTATGTTCATAATA
ATATGTATATCTTTTCAGTACATCATGTACCATAAAAACATTCCAAAAATGAAAGTTTTTAA
GGTACTTCAAAAAACAGAAATTATATACTTTATGATAATGTATCATGTATCAAATATTATTA
TTCTGATCTTTCTTTTATTTAAAGTGAAAGTGTTAATAATTTTGTATTGTTTGAAATGATATTT
TAGAAACATAAATATAGAATTTCAAAATTTATTTTTAAAATAAGTAGTGAAGTTAAATATTT
AAAAATACAAGATTGGTGATAAAAAAGACAATGTTGTAACCATTTGTATATGCAATAGAAT
GAGATATTATACAGATCTGCATTTCCAAAGTTGCTGTGATGTGCTTAGACTATATTCTTATA
AGATGTCAGATTGTCTTAAAATCTGTATTTAACTTAAAATAATCTTAATATAGCAGTTGTTTT
GTTTATTGTAAACTATATTATTAATATATTGCAATTTAAATTTA

51. >MSTRG.1075.24 Gene=MSTRG.1075
CAAACATTGCACTTGATTATATAGTATCTACTGTTCGTACCGGTAGAGAGGTTAGGAACATT
TTAATGTTCTTAACATACCACTTTTTGTAATATTATTTGCACGAATTTGACATATACATTTTTT
TAAGCATGCGATGACGACTCTCAATCATTTTCGAAGATCTGTCAGAGCTGTCCAATTTTTAC
AACCACATAAGAACAATGTTTATAATAATTTCAGATTATCCTTGCGAAATGAATCGAGTTTT
GTGCCAAAACGAATACTTATTGTAGTAAAGTTATCGCGATATTATTTTGAAAAAATACGAG
AACCAAAATTGAACGAAGAACAATTCAAACAGAAA

52. >MSTRG.1090.1 Gene=MSTRG.1090
AATCGCGACTTTTTTTAAACATATCACTAAACTAATTATAAAAATTGAATATCGTTTAAATA
TGATCTTTCTTTCGAAAATTTTTTTAGACAATTGTATAATTATGAATATATATATATATATAT
ACACATTTTTTATATTTAAATTATCCATCTACTTTATTGAAGAAAACAACTATACGAGTGTAT
GCAATTGTTTGCAATTATGAAATAACAGTATAAACTGATTAATCAAGAATCTCTTAACGGA
GGAGAAACTGCAATGACAAGATAGGCTTACTTACTTGAAACTACTAGTCAATGAATCTACT
GGTATGAGATATTTATAGCAACTGCTGGCTGAGGAAACGAATAAAACACATTGAAAAATA
AAAAGATCATTTATTGCATTTATTTCAGCATTTCATATGTACTCAAGATACAAATAGAGAAA
AGGAATGTGTACAAAGGAATTTTAAAATTATAAGGTGGAAAATCTATAATTGAAAATTAAG
AATTAATAAAACAGAACATATTATCATAATCTTTTTAAAGATTATAATTATTTTTGAAAAAT
ATTTTTATAGAAAAAGTGATGCATATGTGAACTATCTTTATAATTATAAACTATATAATTTA
AGATAAAATAATTTTTACATTGAAGCCTTAATAACTTATTAAATTTTGAATATGTTTTTAATA
ATAGTTATATCACAATATTATAATTAGTATGTGATATTTAATAATATATAAAACAAAAAATT
TGATATCTAATTCTATCATTCATATATACAAGATCTTATTTTGGATTTTCCACATAAAAGAAC
GCTAAAAAATGCTATGAGATGGAATTATTCAAGTTAAGGTGATTACTTATTTAATATGAATA
ATAAATAATTATAATT

53. >MSTRG.1129.1 Gene=MSTRG.1129
TTTATAATGTATTTATAATATATCTATGTAAATACTTTATTTAAGATAGTGCATTATATGATT
GTATATTAAGTTTTATGAAATTTTAAAAAATTTATAAAGTTAAAAAGAGTTCTGTGTTGTTTA
ACATGAGTGATATGTTTAATATATATAAAAGAAATAGAGAAATTCACAGTCTAGTAGGGTA
TTCATATATGAAATAGGTGCAGTTGCGTCATGACAGTCGTAAGACATAGAACGGTGAATAA

TGGATGAAAATATTTGGAAACAAACGAAATCACTGGAAATTCCAGAAGAAGCTCTTAGAC
AAACACTAGAAAATGAAAAATCCATAACAGAAAAATTATATGCACTAGTAAAACGATCTC
CATTTGCTGTAGCTGGTTTAACAGGTTTATCTATTGTATGTGGAATAGGTGTATATAAATGG
AAAACAAAAACAGTAAATCCTTCTATATATTTAGTACAGCTAAGAGTTGCAGCTCAGGCTA
CAGCACTTAGTATTATTGGTCTAGGAATGATGTATAGTATGTATAATGATTTTATACTGAAG
AAAAAAAAGGAATGATATTCATGATATTAAAAACAAATAGACACTTAAATTAGAAATTTA
ATTAATTTTAAAGAACAAATTACAATATTTATTATTTAATAAAATATCCAAAAAACATATAT
ATGTAATAAATATCAAAC

54. >MSTRG.1137.4 Gene=MSTRG.1137
GGAAAAAGAATTCGACTGGGATTCAAAAACACAAGGACTAGTTTTAAGTTCTTTTTCTATG
GATATATAAGTACTCAATTACTTGGAGGATTGCTTAGCACGCGTATTGGCGGGAAAAAAGT
TTTTGGTTTTGGAATTGCAATTACTGCTTTTCTTACAATTATCACACCACCTTTAGTTAAAGT
GAGCGTTTACATTTTGCTCGCTTTGCGAATTATAGAAGGAATTTGTGAGGTACATATATCAT
AGAAGGTAATAATTTGAATTCAGGGTGTAACATATCCTTGTATACATGCAATATGGGCACA
ATGGGCACCTCCATTGGAAAGATCAAAATTAGCAACCATTACATTCTCCGGAAGTTTCTTTG
GAACTGTATTTGCCATGCCAGTTGCCGGATTGATGACTGAATATCTTGGTTGGCCTTCTGTTT
TTTATGGTTTTGGAGGAGCTGGTCTTATTTGGTTTTTTTATTGGTGGATAGTCATCAAAGATA
GACCAGAGGACGATAAATCTATTTCAAAAGCTGAACTTGAATATATTAAAAATAGTCTTGG
AAATTTGAAAGAAGAAAAAATTAAACATCCATGGAAAGCTATGCTTACATCTCCACCAGTT
TGGGCAATTATCGTAGCTCATTTTAGCGAAAATTGGGGTTTCTATACTATGCTTACTCAGTTA
CCAACATTTATGAATGATGTACTTGATTTTAAATTGGACAAAACTGGATTTTTATCTGCATTA
CCATATTTAGCTATGACTTTCGTCGTTCAATTTTCTGGACATTTAGCAGATTATTTAAGAGTG
AAAAAAATTTTAACGACTACACAGGTGCGTAAATTATTCAATTGCGGGGCTTTTTTATTTCA
AACTATATTTATGACCTGTACAGGTTTCATTTCAACAACAGCAGGGGTTGTTATTTCTATTAC
TATAGCTGTTGGTCTTGGTGGCTTTGCATGGTCCGGTTTTGGG

55. >MSTRG.1158.1 Gene=MSTRG.1158
AATTGCCGAGCTTCCGCGTGGTTCCGGCGAGAAATTCCGGACAGGAACATACTACCAATAC
TGGCGCTCCCGGGGGTGGCGGGGGCGGGGGTGTCATGAAGCCTCGCGGCTCGTGACCAGG
CCTCGAAGACCGAACCGTACCGTACAGCCATGGATCCCCGTAGATCATTTGACGCGAAATC
CCGCTTCCGCCGCTTATCGTTCCCCACCCCTTCCCCTGGGGGTCATCGGTGGCCTGGCTCTGC
CGCGACAAGCCCAGTCTCACGCTCCACGACTTCTTCCCTTTGCTCAGTGTATCGTAATTCTTT
CCGGAAGTGGGCGAGGGCGATGCCGAGTTTAACCCCTGCTGTAGATGATACATCTTCGGGG
CGAGCATAGATGGAGGTGCACCGAGGCTACCCGGGGCGACAGCCACGCTCGTCATGGTCG
TTTCCTACGATGGGCGCAGTCCTCGGCTTCTTCCAACCACCTGTCCGCGGCCTTCTGCGATC
CCTGTAATTGCTCATCCTCGTACCGTCACCTCGGGGCTGCCTCCTAATCCCTCGTTCACGATC
GTATCCAGATTGTCATGGCACAACAATACAATCGGCCGCGCACACAATCGGCGAGAACCG
AGCGTCGAATGAAAGGCAAGAAGAAAAAGAGAGAAAGAAGAGAAAAAGAAGAAGAAG
AAAAAAAGAAAACCACCTTCGAAAAACAAAAAAACTTCACTCGAATCAGTCTCCTTTCGC
ACTCGTTTCTTTTTCTTTTTTTTTTTTTTTTTACTTCCCTACAACAGGAGGAGACAAGGGAGAG
ACGACTCGGTTTTACATCGTTTCACGCGTTTTACACTGGCGCGCGCGTGTAAATTTGTCGAG
CGGCGGACATCCCCCCTGGCTGGACGGATCGATGAGGGACGACGAGATCGAACGGCGGAG

ATCGGACGGAGATCGAAGGGGACGGAGCAAAGGTGAACCGAAGCGAGCATGCCGAACGT
CGCCGGCCAAGTGAGAGGCTAGCGGCACTACCGACCAGCCA

56. >MSTRG.1166.3 Gene=MSTRG.1166
CGTGGCGCTATAAAGTATAGGTTAAATGTTTATAGTTTATGGTGAATTGACAGTGTTTTGCA
ATTTGTAGTTTAGTTATCTTTGGATGGCAGTATTTTTAAGTTGCGTAGTGCCAAAGTCACTAC
AAATATGAGTGGTGCATCACGTAAAGTGAAAAATCCATTATTATCAGCAGCTGGTGAAGGC
TTTCCGTTTGAAAATTATACTGAAAATGCATTTTTGGTGCCTGTTACGCCTGCGACACCTGCG
GTACTCCGCGATGTACCCGAGATAGATAAGGTGACTGTATTAGCTGTATCTGGTGCAACATT
ATCGGGTACAATCAAATCTATTGCTACATTGCTCATCTGTCCAGGATAAATCCATACGCTAT
AATATAAATAATTAGTAAATAATAAATAGAATATTATCACAAAAAAATTGAGGATGGTTG
AATATTGTATAATCTAAAAGGACTGCTCTTTACTCTGAATGCATATCTAACAGGTAAAACGC
ATTTGTTCATCACATCGAATACTATTCTATGTATAGTTCCGGGAATCACAAGCAAATTGCTA
TTTCTGTTAACTTCAATCAGCATCTTTTTCTTCTCGATGATGTTTTTACCGACATGGTTTACCA
ATTCTTCGGACATTTCTTTATTATTCAGAGTTTCGATCATTCTCATCGATATGTTAATATTTTT
CTGTATTGGAGTTCTTTTAACGTCGAGGTTTTCAATTACTCCAGTCGAGGGTCTTGTATGAAG
ATTTTCCTGTTCCTGAATCAACGATATGTTTTATTGTGTGAACATTGTTTTATGATATTTCTAA
TGTTTGCGATAGGTTAATATTAACTTTTTTAGCGTGAAG
57. >MSTRG.1192.2 Gene=MSTRG.1192
AGCGTCCGTTATCAATCAAATGCTTCGAAAACTGTTTTGCTGATAGTATTTCGTTGGCTATA
GTATAAGTTCATTTAGTCATGTTTTATAATATTACTTGATTTTTAATAATTTTTGCTATATTTA
TTTAACATTAAAAGCATATAAATAATTAATACTTTCCTTTGATATATAAATGCTGCTTTAAAT
ATTTAAAAAAAAATTTTTGCAGGCTATCAATAAAGTGATACAAACTGTTTCACATACTGCA
AGTCAACCACTTATTGTACAAACTATTCCAACTAGCACTGTTCAAAATTTTCAACCTGATGT
ATCCAGCACAGTACAAGACACAGTCTTTCGAGAAAGGAATGCAACATATTTTAATACAGTG
CCAGCAACTCTGCAAAATTTTTTACCACCTAATTTATCATATAACTTTTATAATATTTCTAAT
GTTACATATAAAGAA

58. >MSTRG.1195.1 Gene=MSTRG.1195
TGAAGAGTAAGAAAAAATAATAATGGCATTTACATCGATATTCGTCAACCGAGACGACGC
AAGATTTATTTTGGTTTAGAAATTAGTCAACGATCGTAAAGAGGGAAGGTGTGTGGACGTG
CTTTTTCGCGTCATTCAATCAGGGCCAAACTTTTAAGTACTTGCTTATCGGAGGTCAAGAAC
TGAAAGAATGACCCGTGGAAATCAGCGAGAGCTAGCACGTGCCAAAAATATGAAAAAAA
CTGTTAGAAAATCAGCAGCTGAACAAGAAAGTAATAAGGGTCTTTCATTAGAACAGCGGA
AAGCACGGGATGCAGAACGAATGAGAGAAAAACAACTGAAGAAACAACAAGAACAACA
AGAGAAAGTTAAGCAAGGTGCAAGATAATTCTTTCTATTATAAAACATGTATATTGTTTGAC
TGAATTCAAAAATAAAATTTTAAGTATGTTTTTACAAATCTTGATTATAAAGCATCAAAGCT
ATGTTTAATGTGAGTGCATAAAGAATTCATAGTGTTTTTTAATTATAGCAAATGTCTTCAAA
CTGTGTAATGCAGTACACATTACAAAAGTAGGTTCAAATAATCATGTTATAATGTTATATAA
CAATAATCATGTTTACAAAATATTAATAGCGTGTATAAAAATGTTATCTCTACTCTAATGGT
TGGAAAAAAATACGCATTTGACTGGTTCATCTAGATATAAGAGTATTAATTAATTAAATATA
AGGGTATTTATAGTACTAATTTTTAATTTGTAAATTCAAGAATTGAGTTATTTAATATGATAT

ATAAGCAGCTATTACAAACTATATTATGTAAAAAAGATATGTAAGTACCTTTGTCATTCTCT
TTGTCTTATAATAAATTGTTCAATTTTA

59. >MSTRG.1195.2 Gene=MSTRG.1195
TATGGCTACCAAGAATTGTCATTTTGAAATGGCATTTACATCGATATTCGTCAACCGAGACG
ACGCAAGATTTATTTTGGTTTAGAAATTAGTCAACGATCGTAAAGAGGGAAGGTGTGTGGA
CGTGCTTTTTCGCGTCATTCAATCAGGGCCAAACTTTTAAGTACTTGCTTATCGGAGGTCAA
GAACTGAAAGAATGACCCGTGGAAATCAGCGAGAGCTAGCACGTGCCAAAAATATGAAA
AAAACTGTTAGAAAATCAGCAGCTGAACAAGAAAGTAATAAGGGTCTTTCATTAGAACAG
CGGAAAGCACGGGATGCAGAACGAATGAGAGAAAAACAACTGAAGAAACAACAAGAAC
AACAAGAGAAAGTTAAGCAAGGTGCAAGATAATTCTTTCTATTATAAAACATGTATATTGT
TTGACTG

60. >MSTRG.1216.1 Gene=MSTRG.1216
ATTCCTGGCGTGCATCCACGCTACGTTGGCTGCCGCCGTCAGCACCGCCGTCATCATCGTTC
TCGTTGTGTTGTTGTCGGTGTGATCGCAACAATTTGAAAAAAGGATTATTACAAGACAATCA
CAAGAAAAAATTCTGTTGTTAATGCGCGCTCGAATAACAGTTTTTCCTTTTTAAGGAATCGC
GTGACCGATGTTACAATATTTTGCGCAAGTTCCAAAAAGAGATATTTTTTTAAATTGATAAG
TTATTGTTCAATAGTTCAGTGTTTCTTGTTCCCCTTATTATTGTTATTTTTATAGTGAGCCCATT
GTTTTCTTGTGATAATTGTTCCTGAATTTGACTTGATCGTGGGATCGGTGTTTCAATTCTTCAA
ATTACTGTTAAATCTCGGATTTCTCTAAAGACGATTTCCTTGGACTATTGTCACGTGACAAA
TGTGAAGAAGAAACGTCATCGCATGGCCAGCCCAAGGTATGTATCACAATTCTCTTTAATT
CTAAAATTTTTCTGGATACTACTGGAAGAAAATAAGGAAGAAATGGTCAAAAACAGCAGT
ATTGGTGAGTAAAATTTCCATTAATGAATTCTGAAAGCATCGTGCAGCGATGTCGTCAGAC
GCTTCTTCGTTTTCTCGTTTTTATTTTCTTTTTGTTGGGATCCCACGACGGTCTGCTGTAGATG
GTGACTAGTCGTTCGAGGAAATGGACAAAAAATTTTAATTTCTTCAGCATGTGCGTTGGAG
GTGACGGATCAGAGCGATAAATGTTCTTCTCGTTATTCTCGTCAGCTTTTAGATTCATGTACA
TGCCCAAAAGCAAAAAGCATTTGATTCTCTGAAACTCTATCTCCA

61. >MSTRG.1243.1 Gene=MSTRG.1243
TGAAAAAAGAACAGTTTGACAACTGAGAGTTTAGGGTTAGTAAAAATGGAGCATTATACG
ATACAACAACGTGAAATGGATTATTAATCATCAAAAAGTGGATGCTGGTTTTTCGAGCAAA
ATAATCCTAAGCATATTTTTACCTCGATGGTTTTGTTAATCGCCAAAATTGCCGTGTTTGAGG
TTCGGAGAACCCACGTGTGATTAGCGAAAAACAAATACACAACGTATCACTGTTTGGTGAG
GATTTTGGGCAAAAGGCATTATTGGACCATACTTTTTTGAGAATGAGGTTGATCAAGCAGC
AACTGTTAATCGCGTATTCGATATCGCGACATGATAACACAGTTCTTTCTGCCGAAATTGGA
TGATATTGGTGTGGCCAATATGTGATTTCAACAAGATAATGCCATATGCCATACAGCCAAT
AAAACAATTCAATTACTGCATGAGACATTTCCTGGTCGTGTATTCTCTCATTTCGGTGATCA
GAATTGGCTTCCTAGATCATGTGATTTAACATCATTAGATTTCTTCTTATGGGTTCTTCTTATT
ATAAGTCAAAGGTCTATGTTAACAATCCCACAACCACATGTGCATTATAAGAGGAAATTAA
ACGCTGCATCAACGAAATTCAGCCACAATTATGCAGAAAAATGTCATGAAAAATTTCGAC
GAAAGGCTGCAAACGATCAGCATATTAATATTAGTAACGTTCTGCAATTCAACGCATAAGC
ATTCAAATCTTGGTGCTGTAAACCAAAGTAAAAGTGATCCGCACATGCATATGCACATTGA

TAGCTAAGGAAAGATTGATATCTGATGGCAAATACGAGTGTGTAATTAAGTTGAAATGGAT
ATTCTGCATCGCAGGAAAGAGAAGTTGATATGAATACAGATCCAATCGTAAATGCCAAAG
CGGTCAAATACATACACGTTATATTACATCCCCAAACAGTTTTAAATTTCGCAATATATTTT
TGGAAAATTTCTATTTCATATTCTTGCGTTTCTTTTATGCAGATTTTCATCTCTTTGATTATAT
ACTGCACAATCTCCAATTCAAATATTAATTTTTTACACCATATTTTTTTATAAACGCACATCG
ATCAAGTTTCTATTTGAGAGAGAATTATTTATAGAAGATAATACTTTACAAAGCATATGACA
GTTTGAATAATATTTTGAGCCACATCTATAAAACGATGATAGTTCTTAAAAATACGACATTG
TCCGTGTGAAATAAATTGAATATGACGAAAGAAGAAGCAGCATGCAAGCGCTAACAATAC
TAATTATCTGTAACATTTTGAAAATGAACACTTGAGTTTTGCCACTATTGACAGAAAGCGGC
CAACACAAAGCTACGCTCAACCAAGTAATATGGATAGTTTTCTCAGGTATCATTTCTTTTAC
CATATTTTCGAGGAAATACTACAACAACAAAATTGATTGATGAATCAATCGAAATAAAATT
TAACCGATACAATAAAACGAAATATGTATTCTTCGATTGTATAGTTACATCACTTCGAAAA
CGAGT

62. >MSTRG.1271.1 Gene=MSTRG.1271
TTCAAATAAAAGTTGAATGGTTTCGAAAGGGCTATAATCTGACGTTCTCATTTTTTTAGTAG
GTTGACCAATAAACAGCTTTTCAAGCACAACTCTATTTTTTTGACTGATAACATGTATTTTTT
AGAGCACCAATCGATGGAGCATAAAATTTCCTATAAAAAAGTATTAACACACTTATATCGA
AAAACCAATAGTTTAAGAGATATAGCAAAAATTAAAAATTTTTAGGAGGTCGACCTCCTGT
CATATCATGTTCTCCTAATACAAAATCGAGTTTTCGGACAGAAATTTTTCTTCGATCGCCGG
AATTTGGACATTAATACTTTTTGTTGAATATCTCGCTTATTATTCAGAATATTAAAAAATGTT
TCAAATAAAAGTTGAATGGTTTCGAAAGGGCTATAATCTGACGTTCTCATTTTTTTAGTAGG
TTGACCAATAAAGAGCTTTTCAAGCACAACTCTATTTTTTTGACTGATAACATGTATTTTTTA
GAGCACCAATCGATGGAGCATAAA

63. >MSTRG.1313.1 Gene=MSTRG.1313
GGCGACCGAAGAAAAATTTTTGTCCAAAAAGTCGATTTTGTATTAGGAGAACATGATATGA
CAGGAGGTCGACCTCCTACAAATTTTTAATTTTTGCTATATCTTTTAAACTATTGGTTTTTCG
GTATAAGTGTGTTAATACTTTTTTATGGGAAATTTTATGCTCTATCGACTGGTGCTATAAAAA
ACACATGTTATCAGTAAAAAAACAAATTTGTGCTAAAAAAGCTCCTTATTGGTGAACCTAC
TAAAAAACTGAGAGCGCCATAATATAGCCCTTTGGAAACCAATCAACTTTTATTTGAAACA
TTTTTTAATATTCTGAATAATAAGCGAGATATTAAAGAAAAAGTATAAACGTCGAAATTTC
GGCGACCGAAGAAAAATTTTTGTCCAAAAAGTCGATTTTGTATTAGGAGAACATGATATGA
CAGG

64. >MSTRG.1325.2 Gene=MSTRG.1325
TTTTGTTATTCGCCATTAGTTTTGTGGTTTATAGTTCTAAGTGATCTATTCTCAATATTTCTCA
ATATTTGTTACATTTTTTAATATTTTTTACATTATTCTTAATATTCTCACGTAATTAATATTAA
AGAATATAGTAAAAGTATAATATTTAATAACAATCGTGCTAATATATACTATAATGGAGAA
GAAAATTATTGACCAAAAAGAATATTTGAAAAAGTATTTGTCTTCAGGAGATGGCGATGAT
AAGAAAAAAAAGAAGAAGAAAAAGATGAAATTGGGAGCAAAAACGTTATAGTAGTTGTT
TTACACATTTTCCTGGAATATTTAAATTGAAACATTTTGAATATTTATATAATATTTAAAAAA
TTTCTTATATGATAAATAGTAAAAAGAAAATGTATTTTATTCATTACCATTTCCTTAATATAT

TATTATATTAATATGGACGATATATTTAATGCAAAAAGGAATTTCAATTTTGGTTTTCAAAT
CGATATAAAATATTTTTAAAAATTATATTTATTTCTTTGCACAAATTTTGAGATGTGTTTTGC
AGAGTAAAAATCATTGATGATGACATAGACTTGAAGAACATAAGACCAATCGAAGAGAAT
GAATTTGATATTTTCATCAATGCCGAAGATGCACCTCAAATAGCCGGTGTAGTGGATGAAC
ATGGACCAGTTGATTTTAGTGATAAAAGAAGATGGAAAATTATAGCTAATGATGAAAGTGG
TGATTTAACAATCACTAGTGTAGCAAAAGAAAATAAACAATCTAAACAAGTTAAGAAAAT
TTCTGATGATGATTTAAGCCCATCAAAGATACAACAGAAATATTCAAATGATGATTTATCTC
CTCCAAGAATATCAAAAAGATCTAAAGATGAGAGAAATAAGAAAGTACAAAAGGATGAA
GATAGTGATCTGAGTCCACCTAGAATAAAATCTAGAAGTCATATTAATTCTAAATGTGAAT
CTCCGCAAAGTAATAGTGATGATGATAGCAGTCAAGAAATAGATGTTCCATCTTATAAAAT
TAAAAAAAAGACAAAAAGAAAAGAGAAAATATCAGATGATTCTGATCTTAGTCCACCTAG
AAAAATTAAAAATTATAATTCAGATATAAATTCACCAAGAAAGAATAAAAAATATAAACT
TGAGAAAAATATAATACATAAAAATGATTCAGAAATGAGTTCTCTTAGGAAAAAAAGAGA
TTCTAATTCAGATGTGAGTCCTAGAAAAAATAAGAATGATTCTGATTCAGATATGAGTCTTC
CTAGGAAGAATAGAAAAGATTCTGATTCAGATTTAAGTCCACCAAGAAAAAGTAAAAAGC
ATAAGCATAGAACAAAAGATAATTTTGAATTTAATACATCTAAAAGAAAATATGAAAGTA
ATGAAAACATTTACATGAATGATAAAAATAAAAATAGAAATTATGACTCAAAATGGCATA
GATACAGAGATGAAAAAATAGAAAGAAATAGAATGTATAAACATTCTTATAAAAATTTTA
AATCAGATAAGGGATATTATGATTCAAATATGAGTCCACCTCAAAAAAGAAACAGAGACA
GTACAAGTCCGAATAGATTTAGAAGAAATGATAGAG

65. >MSTRG.1333.1 Gene=MSTRG.1333
GAAGATGGCGCTGATTACAGTATTTTTACTCTATCTCTATCCTTTACTTATTATTTGCTCTCCT
TGTTTCTTTCAAAGTAGTGCAGTCATTTCTAAACTCGTTCGTTTTAAATAGTGAAGTTTTTATT
ATTTGACAATTCCCCAACCGGATAGGATGCAGTCTGATCCTTCATAAGTTTTACTACCCGAA
GCCAATTTTATCGGTTGGATCAAGTTAGTGAACGAGATATCTTGATTCACACGGATCAAAG
CAATGTCGTTAACGAGACTGAGCGCGTTGAAACTGCGATGGGCAACAACTTTCTCCGGTAC
ATAAGACACTCCACCTGAGTTCAGTTGATTTGTTCCAGCTTGAACAGTGATGCCATTCAAAT
TACTCAATCTGTAGAACAAATGTGTTTCATTGATTTATCAGCATTTATCAGCGAGAGAGAGA
CTCTAATTTCATTTCAGTTAAGTAAAATTCTATTTAAGGATAATAATAAATT

66. >MSTRG.1338.4 Gene=MSTRG.1338
CGCCGCGCGTCGTGTCTAGCTGGCGACGTTGGGCGTCCTGCCCCCAAAGCGTCGGGTGGGG
AGCCGTGGTGAACGCAGTTCTCGACGGGTCGCCGAGAAGGTGTAACGGTGTTGTCCTTGCC
AGGCTGCGAGCTGCGGCCACCCGTGCGAAAAGCCACGTGATCCATCCTGTTGGACCGGCTT
CCTCGGCTATCTTTCTTTCGTCCCGTTATTTCGTTTTCTTTTCGGATCGCGTCGCGTGTGCGTC
GATGCTCGATGTGATCGCGGCGAGCCGATAATTGAAAGAAAAATTCGAAAATCGTGAATC
GCGAGGAAAGCATGGTTGATCGTTGACCATTGTTGTTGTCCTCTTACCATTGATTTCGTGGA
ACAGCTATGATTCACGAGTTCTCACGGTTTAGTTTGTCCCGTTTGTTTTCGTTTCTTTTTGTCC
TTTCGTGAATCATACGAGTGGTGGTTTTGTTGCATGAGAAGGATCGCGCGAGTATCAAGGC
CGCGCGTGTTTGAGAGTTGGAGAGTTGTCGGTTGTTCGCGCGGTTGCCCTTCTGGGATACGT
GTTTCCGTTCCGCCATCGTGTTCGCCACGCGGACTTGGACATGTAACGCATTCGAACGGACA
CGTGATTTCTTGTGTGATACAACTCGTGTGGACCGCGTCGTGCAGTGTTTTAAGGGTGTTTTT

TTTAAGGTGCCGCCTAATGAAGGGATAAGAATGCGAGCGGTGGTCGAGGGGTAGAGGAGG
AGGGTGCCAGGGATTTTTCGTGGCCGAGGAGCGGTTTGGATAAGGCATGCTATGCGTCAGA
AGACTGGAAACAGATGTTCTCGCGAAAGATGACAGGGTTAAGTAGATAAAAGGAGCAGCT
GCTGGTTCCAACGACGATTCATCGCGTTTTCCATGCAGTGCACAATGCTCTGCCATCGCGCC
CTTGTCGTTCTCTTTGCTGTCAACGTGCCAATCGGATGATCAGAATTACCCCATCCTCGACT
AAAAAACTCCATCCCAGAAAGACGAAGAAAAAAATACAACCCCTCTCTTCTTCTTCGAGA
ATAAAGAAAAAAAGGGGAACGGATGCGAACACGCCGCGGATGGAAGCACACTCCCTCCC
TCTCCCCCTCCCAAATTCCGCTCGAAGTCAATCATCAAACAGTTCTCTTCGGGGTAAACTCG
GGAATTGGGGCGCAATCGGTCGGCGCAAAAGCAGTTAGAGCCCGCTAATTTCGCCGAGGC
CTCCCCGCCGAATTGGATATCAATTCGTATATCGCGCCGATTCGCGGATTGCGATCGACAC
AGTTCGGGTTTGTACAGCGAAATCAAACCCTCCCCTCCTCCTCCTCCTCCACCGTCACCGCG
ACGTCCGATGCAGATGCAAGGGAGGGGGGCCGGGAGGAAGAGAGAAGGGCGCGGAGAA
ACGAGATTTGGAAACCTGTGCCAAACACGCGGGGAAGGTTTGGATGAATTCCGGATATGC
GCCGGGATCCGGAAACCGACATCCACGAGCGCCAACGAGAGGAGGACGAACCCGGTCAA
CTTCTGCCGTTGATGTTTATTCGCCGAGAGCAACGGGGGTACGGCGACTGGGCTTGTCGTTC
GTCGCGAATTAATTCACCGTTTCCGGGTCCCGAACCTTTACGGCTCTTCGACGAGATTCGAG
GCTCGTCGTACGCCCTTCGATTCCACCCTTCGGAATTCTAGCAGGAATCCGGCTGGATCGGA
ATTCTCCTCCTAAAAAATAAATTGGTAAATCCTTGTTCTTCTGTATCCATGGTTTTCTAGATT
TAGGAATCGTACGATTTTAATATTTAATATTTAAGGACGAGGGATAATTGGTTTATTTGATTT
AAAATGTTCGAGAGTG

67. >MSTRG.1340.1 Gene=MSTRG.1340
AGATCCAAAAGAACGGAATTAATCGAAGATGGAAAAAATTCGAACTTTTAACGTTCTCCTC
GATAAAAAATCAATGGAAAAAAGGAACACCGCTAAAAACTAAAGACCCGGAGAGGAATA
CCAAAATTAAAAAAGGCTCGTTTCGAGGCGAGTCACGAACGGGCCGGATGGAGGGGCAGT
TCGAAGTGGACGATGGAAAAAGGGTCGGCTCGTTAATCCAGATCGGCGAGGAAACGCCGG
AAGGGACGGAGAAAGAGGCGTTGGAAGGGTGAGTGAAGAAAAATTGGACGGAT

68. >MSTRG.1343.2 Gene=MSTRG.1343
AGAAGCTCGATTCCAGCTCAGTATTAATCGAGTCGTCCGATAGTACGACAGGTTTAAAGTG
TTGTAATCATAACATTGACACGTTGAACAGTACGGATTGTTTGTTTAATCAAATTGATTGGA
TTCGCACGTGGAAAAATCGTTAATAATCGATTTTTTATTCTACTATTATCTAAACTACCGATC
AATGATGTAATCGAGTAAAGCGATAGCCGAGAAGGATAGAGACGAAATCATTCGCAGACC
TTGAATACGCCGAATACAACGAAAAAGAAAGGTTATATTTCCATTAATTTTTACGAATATG
TGTGACAAAGTTGATATTGAAGCAAATGTTACTGGTGGGGGGTCTTCTGGACAAGCTGGTG
TTATACGATTAGGTATTTCATTAGGGCTTCGAAGCTTTGTCGATAATGACATGATAGAAAGA
ATGAAACTTGCTGGCCTTCTAACGAATGACTGGAGGAGGCGTGAAAGAAAGAAGTGGGGT
CAGGAAGGAGCTCGTGCAAAATTTACATGGAAGAAACGTTAATTTTTTTTTTTAATTTCCTG
CATTTAAATTTCTATATTAAATTTGAATAAATTATATAAAATTTATTAAACATTTTGAAATAA
AATGCATGTTATTATAAAATTCTGTAGAAAAAATGCAACA

69. >MSTRG.1385.1 Gene=MSTRG.1385

ATATATAAAATTCATTATACATATTTATTATAACACAGTTCATACGTTGATGAAAAAACACG
TGGTAAAGAGTGTAATTATTAAATTTAAAGCAAAATAAGAACATAATCAGAATGCCTCTTG
CTAAGGATTTTCTTCATCCAAGTCCAGCTGAAGAAAAAAGAAAACACAAGCTAAAAAGGC
TTGTACAGAAGCCAAATAGCTATTTTATGGATGTGAAATGCCCTGGGTGTTATGCTATCAAA
ACTATTTTTTCACATGCACAGAGATCAGTAGAATGTGATGGATGTCGCACAATATTGTGTAC
ATCTACAGGTGGTAAAGCTCGATTGACAGAAGGTTGTTCATTTAGAAGAAAAGTTCAATGC
TAATATAATGATGGTTAAGACAACATAATGTATACATTTCTCTTTATATTCAATAAATAAAC
AGAATTATTACTTAA

70. >MSTRG.1408.1 Gene=MSTRG.1408
CCAACGGACGAGCGAGCAGCGACGGCACGGGACTGCGATTGAAAGTATCCACGCGATCTT
CGCGAAAATAAAGAGTACAGATACAGGCATACACCTGAGATTAAAACAGGTGAAGAATC
ACATCGTGACAAAACATCTATTTAAACAGTAGTTACCAACCGTTTTGCGATAGCAAATCAA
TCGCCGGTGTAACACGTTGGTTTGTACGGTCCACTCGAACGAAGCGCTCGTTTCTCGTTCGG
TTCGCTTCGGTTACGATCGTGTCGGCCGCAGTGCTTACGGTGTTCGTCTCGCGTGTTTTCATT
TCAGAGAGGGAGAGTGACAGCGAAAAGAGTGGTGGAAAGAAAACAAAACTGTTGACAGT
GATTAAACGTGACTACTGCAAAACATCCTTAAAGGATATCTGTGCTGTGTGACATTCTTCTC
GTTCGAAATATCAGGAAAACGAGAATTCGATTGCTCACAAGGACGAGTTGCAGGGAAGGA
ACAGTGTAGTTTATTCATTAAACCACTAGGGTGCGTGTTTCGTATGTGTGATTGGAATCAGT
ACCGGTACAACGGCATTACGTGAGACATTGAAAATCGAATTTGTGCTTCGAAAAGTGTTCG
TTGTTGGTTAAAAAGTTCATCGCGACTATTTTTATTCGTGGTCGGATTGTGTTATAGTGTCGT
TTCGTGGTCGATTTAAATCCGTTACATTTTTTCGAAGTACATCGATGAATCATCGATATTTCG
AACTGAAGCTGGTTCAAATCACTTGCAAATCGTTCATTCTATGAGGTTAAGTGTAGAAGGA
ATATCAAAGTTACAAACAATAACAGTACAATTTGCCTAAAGTAAGTTATAGAATCAAATTC
TAAGAATATACGAGAAGAAATATACAAAAAGATATATACAAAACATTCCAAGGAACGAA
GAGACGCCATTAGTCTAACAGACCTGAGGCTATTGTAGCCATTATCTTGTAACGTACGACA
TATTTGAAAAATTGAAATCTTGTTTCGCGCGCAAACGGAATATCATATTGGCTGCTTTCGAA
AAAAGTGGATTTGTTCAAATATCTTTTTGATCAACTACGAGAAATAGACCAACGTTTATTCC
AATATATCGATAAAAGAAATAATAAAATTTCTGGTATATGAAAGAAAATACGTTTGAAAG
AAAATTCAATTGTATATTATTCCTGTGGAAAACGATCGAAAGAAACAACTAAAGACTGATT
TGTGATTGAACTGTGATTGAAGCTTTTCTTCACTGTTTTTACGAAAATTCAAAAGAATTATCA
TCTTAAGATATATTCCGAGTGTCAATATTTCGCCATAACGTTACAATCTCGACACTGAATTC
AATGGTGGCGCAGTATTGAGGAAACTAAAGTTGCTTTTAGCAACTGAAATTCAACTTCAAT
GTTCTTCGTGTGCTGTCTACTAATTCGACATCGTGTTTCGTGACATTATATTCGCGTGTGTGCT
ACAAAGGTGCTCGTGGACGAATGATTTATCGTACTATCACGTGAAAAAATATATTCGTGTG
TGCAAAGAAAGCTTTTTTTCCAGTTGGATTACAACGAAGCAACGACATTGTGTTCACTTCGT
AGGAGTGGAAGGATTTGTCGCGACCTCGTGAATTTCGAATTCGTCGTGTCGATCGAGACAA
CAGACAGCGCGTGGCCAATTACTCGCATCGTTACAAGCATCAATTGATTAATTAACACCTT
AATTAATTCGTTCGATCGGTTTTAACTCTTCGACACCGTTGCTGCTCTCGTGTCTACACTTTA
ACGTTTTCGTCCGTTTTCACGAATTTATCAAGATCGTATCGCGATTAATAGTAATCGGAATTT
TTCTCATTTTACACGCCAACGTCGGAAAATTGATTACACGCGGTTCAACTTCGTGGCGATTA
ATTCCAGTTTTGATTGTCAACGTTTTCTCGAGTTCGATTTTCATCGTATAGGCAGGATCTATT
CTACATAGATCCTTTGTCCGATTAAACCGGGATTACGTGCTAGTTCGTGGAGGCAATCGGTC

TGTAATTGTGCCACGCGTACGGTTAATCGCTTGTCTACGCGAAGAACAATAAACGGTGGTG
TTTACGTGACGAAAGTGCAGACGCAGCCCACTCGGTCACTTGCAAAAAGTGACCCATAGTT
TTCTCCTGGAAGTGGTACACAAACGGCCTCAAGATTCAGCTGGACATCATGCCTTGGTTCAT
CCTATTTCTTCTTCTGTTAGCTGTGATGGAGGCCTTCCCTCGGGAGGCCACCTGTTCCCGCCT
GCCGGACATCTACTGGAACTCCACCAATCCAATGTAAGTGTCCACGCAGCACCATTCCGCC
GATAAAATTTCCTGGCGTCCTGCGGCGCGAACTCGGCGTGACAGCCGGCCAGACGAAGAT
GAAGGACGCGGTTGCCGAAAGAGAGTGGCACGACTCGCGGCCAAATTGGACGCTGGAATG
ACGAATCGCCGCTCTGCCCGTACGCGAAATTCAATTAGGCCGGGTTCAACGGTTAGCCCCG
CGGTCTGCCTTCGAACGAGCCCCGCGAGACTGCGAGACGAGCGGCCTGGCCGGTCAATTTC
GATCTCGGATCGATGTCGCGGCCGATCGACCCTGCAACATCGTCCACTCGTTTTTCCACCCG
AGGTA

71. >MSTRG.1412.1 Gene=MSTRG.1412
GTGGTACTGATACTAATCTATATACCGGTCCTGGTGGATCTTTTGTGGTCTCACGTGGTTCAT
CGGTGTCAATTTCTGCAGCCAAGTATTGTCCTGTCGCCAAGTGTTTAAATCTGAACAAAGAG
TTCCAGTGACCAGCTCCTCCTCTGCAAGGATCGTGTTGAACTACCTCAACCTCCCACAAAGC
TTTACTACTCGTAGCAGCAGTGGCACTCGTTCTACCAGTGGTCCTAAGAAATACATGCTGCT
TCTTCTTGTACTCGTCCATCGTGAGAAATTTTTCTTGCTCTGCGTGAAACAGTCGAACGACAT
CTCCACCCTTTAAAATCTCCTCCTGATTCTCCCTGTGCTCCATAAATAACGTTACTTTCCATG
ATGTGGCCGAATTCACAACGTTCACCTCCTTGCAACCAGGATTGTCGCTCAATTCATAATTA
GCGGCCACATGCAACCCTTGTCTCCCAGCATTCACTGGTTCCAAAATCACTTTGTCGCCAAC
AACGACACTGTCTCCGTCGCTTCGAAGCTTGTAGAAGGGCATTATATAGAGCCAAGAGCCC
TCGTTCCCATTCGCGTCTAAATACACCCTCATCGCGTTCTTTTCGAGGAGCGCCGGAAGTCT
CTTGTTCACCGTCAAAAATTTATTGGATTTCAGGTGTAATAGCTGGACGACGTTACCATAGG
AGACTACACTGCCCAACAATTTCTTGTTCTCAGTCTCATTTTGCTTCTTTTCTATTTCAGCGGC
GTGGTGCAGTCTTTTTAAAAGCACCGCGTCGGTGCCGCCAGCGCTTTGTTTCGCCGCCTTCC
AAAATTGCTTTTGGGCGGAATAACGATTCATAGGACATATTTTGAAGAGACAATCTCTGAA
CTTCTTTGGTGGATTTGACAAATCTCCAGCTTCGGGACATACTACGCATCTATCGTCCACCA
ACCCGAGGGTGGAAAGAAATCCAGAGACACTGCCCTCGGCGTATAGGCTGACGATGTCGC
CTAGGTGGAGGAAGCTCGCCGAGCCGAGGATCTCGCCCATCTTGTGATTCTCTGTTGTGTTC
GAGCCTCTCCTCTCTCTCTTCCTTCCTCTCTCTCCCCTTTCTTTCTCTCTTCTTCTCGAGTATCT
CAAAGTCGGAGAAATTCAACCTCTCAGCCGAGTGAATTATTCATGGCTACACGTTCTCGCC
GCTTCGTAGCAGCTCCAATACTTTCAGTGCACTTCACGTTGTCGATCGAAATCGTTCCCGAG
GCGGTAACGCACCGGTTTTAGAGAATGCCGGAACACTTCCTCGAATGGCCAAAAATATTTC
AAATACGTTCACGATGATCACACCTTACGAAAAAGGCGAAAACGTTCGTGATCGCCTCGTT
AGTTCACGTTTACGTGTCACAGATATTGTGATTCTCTTGTGCACAGATTTTTGAACATCTATC
ACGTTTGATTCATAGACGAAATTAGTTAAAATATTCACGAAGTTGTATTATATCGGAAATGT
GCGACTTGATATGGTCGTAAAATCACCGATACGCGCGTATATACACGATTATTAGAGGACG
ACGCACGAGGCACAAACGTTCGCACCGCTCGGTGGTTCTG

72. >MSTRG.1427.2 Gene=MSTRG.1427
CTTATACTACAGAGTTCCTAGGTATCTTAACTGTATATAATAGAGAAATGTCATTGTCGTGT
ATTACTTTTAATTTTGATTGAATCATGTCTATATAACCACTTATATCTAACATAAATATTATT

TCTAATATTGTGTAGAGTGCTACGTGACAATTTTTTTCGTTTATATTATTAATATTTTATTTC
ATAAATTTCAATGATCATCTATTACATTGAGCATGGTAGAAGATAAAAGATATGAAATTGA
CAGTCGAATTGAGTGTGATGGACATCAAGGTACATTAAAGTACATTGGTCCTGTTGGTGAA
ACTAAAGGTTTGTGGCTTGGTATAGATTGGGATGATCCAACTCGTGGAAAACATAATGGTA
CATATGAAGGAATAAAATATTTTAAAGCTAGATATCCTACATCAGGTTCATTTATACGTCCT
GGTAAAGCAAGATTTGGAATTTCTTGTCCTGAAGCTATTAAAATTCGTTATGGTTTCATCAA
TGATGAATTAGCTGGTATTGATAGAGATACATTAATAAGTTTACAAAAAGAAATTAATGCT
CCTTTTTTGGAAGTTGTTGGTTTTTCTAAAGTAAATAAAAAACAAAGCAAATTTGATCAATT
AAAAATAATATGGTTAAGAGAACAATGTGTTAGCACTGCGGGTGATTCTGGTGAATTAAAA
GAATTAT

73. >MSTRG.1435.2 Gene=MSTRG.1435
ATGTTGTAAATAAATATTGACAATTACAATTCTGATTGTTCTGCTTCAATTATAATTGATGAA
ATTGTAGAAGAAATGTCTTCCTTTAACTCTAACTTTTACTAATATTATTAGTTGATAAATTAT
TGTGCAAGAGGGCCCAGGGGTGAAAAGTAGGATAAAATGCAGTATCAATTTTTTCCCCTTT
TTTTATTTTTTATCACTAAATGAGTTAATCCCATTTTCCAGTCGAACTTGGCCTGTGTAATCTC
ATCGAGATTATTGTGAGGGATCATTGTGTTAAGTGATTCGAACTGATATCACGTAGGTTAAT
CGTACTAATTTACTAGAATTGATTGTTATCGAGAAAAGTATAAAAGTGGAGTATTAGTGTG
ATCGATTTGCGTTTTTGTAAGACTGAGAGAGGAAGAAATATTTCGAATTTCGAGTTTTTGAG
AGAAGAGTGACAGATGCAAGAAATAGATTATATTCGAAAAATGGAAAAATATTGCAACTA
TTGATCCCTGCAAATTTAGATTACAATTATCATATTGAATAAATGTAAGAATTAGAAATAGT
ATATAATATATTCAAAAACTAAAGATAGATAGTCATTTATAAGAAAAGAACGAATTTATTG
AACAGATAAGAACAAAAAAAAAATGAAATTTTGCTTAAAAGTCTAAAGTATCGATAATTT
AAAATAGAAAGTCATTCTAGAAGAAATAACTAAAATAACTAAATAACTCATCTAAGAAAT
AACTAAACATGCTCAAAAAAATAAGTGGCCTATTTATAAACACGAATCGAAACTTTCCAAT
ATGAAACAGTACAAATTAAAATTTCATAATATTATAAAAGAAATTACCAAATAGGAATCCT
TGCTCTATCACATCCGATTCTTTTCTCTTTTCCTCCCCTGTACCCTTTACACATTCCAAAACGC
TACTCTTGCAACCCATTCTCGAACCAGCTTCTCACTTCGATGCGCAATCTTTTCCCCTTGAAC
ATATCGTCCAAACTTCATCAACTGGATTCTAGAAATAAAAGATGAAAGAGAGAAATGAAA
AATAATTCCTGTTATCGTTGAAGTTTCTCAATTTTTTTTAAAACAAAAATATAGAAAAAAA
TAAATAATTGTTCAAATTAAATGGTCCAAGGGAATAATAATTTATGAAAAATGCCACGAGA
TTATTTTTTCAAATGCCAGATAATAGATTTGTGACAATGGAAAATGTGATGCATAATCGATT
CGATGATGAGAATTTCGGCTTGTTCGAGATGTGTAAAGAGATTGAAAGGGTGTCTGTCGTG
GAACGATGCATAAACTCGATATCAGGAAGTATTGAAAGAGAGAGAGAGAGAGAGAGGGAGT
GGAGAATGGTTAAGGCAAGAGATAATGAATATTCGTGTGACGTTGTCATTCGACATTGAAA
ATCGCATTTTTTAAACGAGGCTTACCATCGAGACAAGATGAATGTTTTGCATACATGTATCT
TGAAGAGTGCATGATCATTGGAAAGATTCTTGCATGGATCGAGCACTTGGAAAATTGTTCA
ACACTGTTCGATTACACTGATTACACTCAACATTTTATTGGATACACAAGTTCTTTCTTGATC
TTAAAATTCTCTTCAAAAACTGTCGCTAAAGTTTCTTCTCAATTGTTGAAAATTTTGAACGTA
TCATCAGTTTTTCCAAGAAAACTGCGTTCACTTCCTTTTTATGATGGCTTTCCTCTCTCTCTAT
GATAAAGAGAAATAAGATCCAGAGAAAAAGAGTCAGTAGATATAGAGCTTAATAGATTTT
TAGAAGATATTGCTATAGATTCTTTTTAAATATAATTATAATATTTAATTCAAAATATATAA
TTTAAAAAGGAGAAAAAAAATTAAGAAGTTAGTAAATTCTTTATTAAAATTACGTGTGTGA

TATGTTAGAGTATGTTTCATAAAAATTAATAATATAGACAATATAGATTAAAAAGTATGAA
AAGTATTAAGTAGTAACGAGCTAATAAAATTAACACAATAATATAAATATTTTAAGAAAAT
TGCTTATAGATTATTAGATACAAAAATATATTTGGAGAAATGAAAATTTTCTATCTAAAAA
ATCATCTTAATAGAAAGAACAATAACGTCAGAATCAATAGAATAAGATAAAACAAATTAA
TTCAAGTCTAAATAATAAACAAATAAAAAAATAATCTAGCATCTCCTTGTATATCTCTGTAA
TCAATTCTAACTAGATTCAAAATCAAATCCGTGTTCTGTAACATGCACAGCCACTACAGTTC
CCCGAACAAAATCATAAACCAGTTATCGTCACAGATACGCAACAATTTATGATATTTTTCA
AGATAATGTTCCTCTCCTAGTTTCATTCAAATTTTAAACTATAATATATCATCAACCTCTTGT
TTAAATTCTTTCCAACCAAAACATACGTGATTAAACAAAGATTAATCGACAACAAGATTAT
TAGAAAATTGAGCCAAGAAAAATTATTTTGCCAATTATTGACCAATCAACGTAAGAAACAT
ATACATTATATCTTGTACGTACGCACAAATACTAGTAGCACGCACCGATCGTTGTTATTTGC
TTAAATTAAATCAAAGTTAACCCCTCCCCAATTATTCACGGAGCACAGAAGAAACGGAAG
AAAATTTATTTTTTCCACCAGATAAACGAAATCAATCTTGACACTGAATCATGCGACACTG
AAGGCCAAGCTTTCAAATCACACTGGTACACGCTTTCTGCTCCCGATTAATTCTCCGCTTTA
TCTTATCACACCAGCCTCACCAACAACATCGATTCAATTCCAAAGATTCCTACATCCGAAA
AATCACAATCGAACGATTCGCAATTTTACAAAACTCCGCCCGATTCCCGATCTCTTCCTCGA
TCAACGTGACGTGATAAAAAACGTGTGTTACATTTGAGAGAGAGAGAGAGAGAGAGAGAG
AGAGATAACGATAGAGTCGAGAAGAACTTCGAGAAGAAAACGTGTTTGAATGATCGATCG
GTTTGGACGTGGACCAAAGAAATCGTCGGATGATGTCGATCGCTGGCTCGATTCTGATCGA
GATCCCGTTTCAACGTCGGCTCTCGATGCCTCCCAGAAAGATTTACGGCCAGAGGTCGCC
CGCTTCCTTCTCCGCTATTTGACTTTTATCATTGTCTGCTAGTTTTCACTTGGGAATAATTAAA
GTCGCAACGGTTTGGATTTCGAGATGGAATTCAGGATCGATAAATCGGTGTAATGATCACT
GGTGCGTAAATCCATCGAAATTGATAGGAAATGTTGCTGAGTTTTTGGCAAAATTGTGTAA
AACGTGATTTTATGTAACGGAGTAATTTTATTACGATGAATGATTTAAAGAATAAATATGGA
TATGTAGAAGTTGTAGAGGAAAATATTTGTTTAAAAGTCGAAGAAAATTTGCCAGATCGAA
AGTAAAATAATCTATAAAAATTCATTCGATTCATCTCTGATTGAAATTTGAGAAAGATTTTG
TTCTGATTGAAATTAAAAATTAAGATGACAAATCTTTGAAAATTCGATATAAAGCTTATTCA
AATTTGAATCATTGTTGAGATTTTAAAAACTAGATTCTTAAATCACAAATTACTCCCATAAT
AATTTAAAATTCGAAGCACAGTTTTTAAAAAATTAAAATTATAAACAATAATTCTTCCAAA
ATTATACATGAAACTTATCTGAATATACTTGAATCATGGATGCAGAATTTTAAATATTGTTC
AATTCAGATGTTTAAATTGAAAAACAACAAATATGAATGCATACAAAGACTATCATACTAA
TCAAAGTAAATTTTCAAACCTGATATCAACAGTTTATAAACAAAAACAATTCTTTAAAAAT
TCCATAAAATAATCACTAAATCGTTAAAAAAAATTCACATCTCGAAACAATCAAAATTAGA
GAAACAATTTTTCCAAAAGTCCTCGTACAATTTACACTCGATTACTTCATTCCGTGCACACG
ATAAAATAATTCGACGTAACGATTGACCGACCACGGGATCACAATTTCATCGACGATCCTC
GAGTCGATCGATCGAGGATCCATTGTTAGGCAACGCGATATCCCGAGATGTGCGCCACGTT
TTAATAAAGCTAGACCCAGTTGCAGAAGACGTTGTGGCCTCGAATCCACTGGTTAAAAGTT
TTCGATACGAGAAGGGGGATCGGGTGATGCTTGAACCCCTCTTTTTCTCGATCGTCTAGCCA
AATATTTTGTTTGCTGTTTGCAATTGCGTCGATGGGGGGATTCGTTATTCCGGCATTACGTGA
TGCGTGATTTATTAGAGAGTTTTGATAAGAGAAGAAAAATTTTTTGGCAAATATATCCTCCG
GATAAAAAGAATCTGTTAATGATAATTCAGAAAATATTGCACCTAAGATTGTATCATTGTAT
ATTGGATTACATTGTACGTTGAGTTTAGATGGTAGATTATCATAGTACATGTTGAAAAGTCA
CACATAGAAATATCATATAGCAATATCACACCAGTATGGAATATATGCAAATGCAAGAGA

AATGAAGGCCTCCTAAAACTTCTGACCGATTGAAGTAAGAATACTTTTTATTGATGTTGATC
GAACAAATTTTACTTTACCTTTATTTAAAAGAAAATCTAAAGCACGTTGTTTATCTTTTGTAT
CCCCCCTAAAAAGAAATAAATCACGTAATCTTAGCCAGGGATCCTAGCCAGCATTGTGAGC
AGGTGAGATAGATGATTTGGCTAGTGCAGACCTATCTACTTTTATAAATTATTAGTATACAG
TAATAAAGAAGGAAAATTATTAGTATTTGATCCCTTCAAATCGAATAAAATCCTATCCAGC
TGGCCAGTATTATGAGTATACATATAAAAAGTTTCTTTTGGTAGCTTGAAAATTAATATAAG
TATATGTTTTTTTAAAAAATATATATGTAGGCTAGTAGAATTAATTAAATGAAATAGATCTT
AAAAATGAGCCTTTTAGTGAAATTATTTGAAAGCCAAATATCTATTTAACTAATAATGGGA
ATATTGAATAAGTAAATAGTGAAAATTCACAGGTTTATTAATAAGATTCATTGCGATGATGT
TTCAGTAACTAATGTAAGTTCTTTAACTGAGCAATGTTTTTTAAATTTTTTTACAAACTGTTT
ATAAAAAAGTGTTTTAAAATATAAATGGAAATATCTGAAATAAGATGATGAAGAAAGAAA
AAACTTTTGTATGATAAATATTAAAAGAAAAGGGGAAAAAAAAGAATAATAAAATTAAAA
TTATACTTCATAGTATTCAATAATTCAATAATTTGATCGTGTTTGTCTAATTGAGAAAATAGA
ATGGAATGTTTATGATAGCAAAACAATAATATAGATAGAATAATGATAGAAATAGTAGCA
TAAGACAATGTGAATACGTTCGCAATTCGATACTGAACAACACGTTATGAAACATTCAATG
GAATCTCCTTGAAGGTATTATTCGATTTGAAAGGCTAATACTGCACACTGTTCGAATGGCGA
ATAGCAATTGCACATCACGGTCTGGTTAGTTTAGCTTGCAGAAGCTACAAAAAAAATCCTAA
ATGCAAAACGTATTCGATTATAAATATTTAAACACGAAGAGAAAAAGGAAAGTTGATACG
ATCTTTTTAAACTTAAAATTTCTTGAAACTTAAAATCTTTTAATCTACTTTAATTTTCGAATCT
TTAATAACGCAAAATTTCATATTTTCATTTTTTCTCTTTCAAAAAGATTACAGAAAAAAAAA
TAATATTCGCACAAAATAAAAACTATATAATCAAATATTATTAATTAAGCCAAAAATCGAT
GCATAACAATTTTCATAGAATTCAAAAAAAAAAAAAAAAATAATAAAATTCGCTCAATTTA
ATTTAAAATATAACATTTTAAAACGTCCAACAAAATTCATATCAAAGCAAAATATACAAAT
AATCAATTTTTTATCATTAATAATAAAAATGAAAATTTTAAAAAACAAAAGTCAATTATTCC
GAGAACTTCTTAAATCTTTATTATTAAATTTGAAAGAAAATTAATCCAAAGGAAGGAAATT
TTACGCTCGATAAAACAAAGTTAACCGAGTATTTAAAATTTGCGCGTAGCAAATATCAAGA
TTATTCGACCAATTGTTACATGCGAGTTTACTCAGCGGTCGCACAAAGCGATTATTTATACA
GGGGCGCATTAATTTTCTCGACGGAAATTATCTTTGCCTGTGAGCTCATCGTAAATGCATTT
CTAATTGTCTCCCTCGTTGCAACTTCATCGTGGAATCTATATCTTGCTATATGACGTTTAAAT
TATACATACCACACCTCTCCAGATGCGTACGTAGAGGCTGAAGTAACTGTTATTCACGGGT
GAGTGACGACCATTAATTTTTGACAGTAATTGTTCCATTTTTCTTACCCTCTTTCTTTTAACTA
GGTTCAAAATAAACTCCCTATATCTTAAAGCTTTTGCAAATTTTGAAGAAAATTTGATAAAA
AAGAGAATGGCCCATGGAATTAGAATTGAAATTTAATTTAAGTATTTGGAATAATAAATAT
ATGAAAAATTTGATTTTATTAAAATCATTTTAAAAAAATAATAAAGGATAACGAAGAAACT
AATAAATCTCTCTACCCTCTATGATAATATATTATCATATCATGTCATATGACACCATGGTA
CTAAAATTTAATAAAAATAGATATAAATAATTCTTAAAAATTTTAAGAAATATATTATAAA
TTAAGAATAAATTATATAAATATTTAAATTAAGACTTTTTTGCGTTTAATCGAAGTTAGATA
ACTTAACTTATAAATATAAATTAATAATTGTAAAATAGATTGTAAAAGAAAACTCTCGTCTC
GAATCTAATCGATTTATTAACTAAAATCTTTTATTTGTAAGTGATACATCACATAATTAATTG
ATAATTATTATAATTTAAATAAATTTATTTGTAAAAACGATAAAACAAAAAAAAAAATAAAA
AAAAATAAATGATATTGTTAAAATACTATAAAATACTATACTATACTTAAAATAATATTTG
AAGAATTGTAATTAAAAATGTAATTAAAAATAAGTTATACATTAAAAAATAGTTTTATAAT
CTTAATAATAATTGCTAT

74. >MSTRG.1438.1 Gene=MSTRG.1438

CGACGCCACATGCCTATATAAGAGGATCGCTCGGCGTGGCTCAACGCATTTGTAAATCAGT
TTCCAGAGCAGTCAGAAGTGGCGTTCAAAGTGTGCAGTAGTGTGAAAATTTCTTCCTCCACT
ATGTTTATAGTTAATTCATCATCATTTCTTGGCAATAATACCATTATTGCAATATCATCCACG
AAATTGAAAATTCTCCATTTCACCAAAGAAGACACATGAATTTTCAAGAATGAAATTATTG
AAGAAAGAAAAAAAGAATGGCTCGACGCATTCATAAAGAAATTTTCGGTTCGGATGCAGC
GAATATTCAATTCCACCGACTGATCATATTTCTTCTATTATTGCGATCTACGACAACATGAC
ATGAAAACAGACGAATGGATTGCGAAGTTGGACTATTTACGTGCACCCTACGGGGTCGGTG
CTTGAACGCAACTATAATATGGTCTGATTAGAGTTTGAAATTTCTTCATACATTTTGAACTAT
TTGAGAAGAGCATCTCGAGATATATTAGGTAAGCGGTTATTGAACCGATGTCTTTCTTATTG
TTCTACAATCTTAACGTTAATATAAGATCAGAAAATTAACATTTAGGGCTCACAGTGTCGCT
ACTTAATTATTTAATGCAAAATAATATAAATATAAATCATATTATATGTTAAAAATAATATT
TTAAATTGAATGTATAAATTTTGAAATATATCACATTTCAAAGAATTCTTTTTAAATCAAAA
TGATCGTAGCGTCACTTTAGCCTTTATCCTAGTTTAGTTACAAATCAGTTATCTAAAATATCG
TTTTTTATTTCCTCTATGTTAAATAGAATTTAATGTATTTTCACGATGTCTGCTCACTTCTGAG
AGAATCGTAAAGTACCGACTGTTTAAAATATGTTGATGAGGAGCACTCGTTAGTTTTAAATT
TTATCGATTTTTAACTTTATTTTTTAAATTTCGTCCGTTTATTACTTTACTGTAACTTCTTTAAT
AAATATTCGATCAAATAATATATTAACTTATCTCGAATTATCGAAGTTGAAATCGATAAATG
CAAATTGATATTTCTACGAATGTGCCTCATCCACGTGAAAATCTTTAATTCAACGTGACGTT
CTTTTTATAGTTTAGAGCAGACTCGTAGTTAGAGGTCGAGAATAAATTATGGGCACCATCTT
GTCTAAACCAATACTCTACTACAAAGGCATACGGAATTTGGGAATTGAGAAAAAAAAGTA
CAGAATCAAATAGAACAGGAAATGAAAAAAAAAAGACGATTTTGCGGTTAAAAATATCC
AATATAAATAATTAAATATACAATTTCGAAATACGATTGAAGATCTTCCGGTTTTATGATTG
GAAACGTACGGTAGCCTGTTTTTCTTCATTTAGAAAAAAAGTTAAATAAAAAGACAAAAT
TCTATAACGACGAATCATTTTGTTCCGTGGTGTCACGAATCTAAAAATAAATGAATTTTTAT
GAAACTGGGTAGATATAATAAAATAAAATGAAGATAGTTGAATTACGTGTATTTTCTAAAA
AAAAAAAAAGCTTCAACTGTACAACGTATTTATTTATCTCTATTTTTTAAATATTATGAATAT
TTTACAAATCACTTGCACAAAACTATTGCGTTTTGGGACGGACAAATGAAAAAGGCGATTT
TTTTCCATGGGTCATGTGTTCATAGAATCTAGTTTAAGTTCAGGAATTGCTTCTTGTATCTTTT
CGATGTCCTGTAGAGTTGTTATGCCTTTCTCCTATTATTAACAAAAAAAGACATTCTTAGTAT
ATTATAATAATTTTTATATATCTTTGTGATTATGAGAAATTTCTAATTTCTTCTAACTGTTCG
TAAAAAAATATAAAGTTTGTTTATTATTATTCTTGCAACATAATCAAATTTGTTAGATTTCGA
TGATTAGTGAATGAAGTAAGAAGAATATAAAACAATTCAAGAACCAAGAATAGAATTCAA
GAGATGAGTGCACAATTGAATAATTAAATATGAAGAAAAATCTTTTATAATTACAAATATG
TGATTGCATAGTTTTTAACTTACATACGTTAACAAAAAAAAAATTGTTATTCTTAGAATACA
TTTGAATTCAATAGTTCACAAAGATTAGCAAAAGAAGCAAGAAGAATCAAGATAAAAATT
CAAGAAAAATATTATTAAATTTAGTAAATTGAAGAAAATACCAAGAATATAAAATGTTTGT
CAAGAAATTAAAATAAAATTCAATCACATTCAAAGAAGAAATTTTTATAATAAAATATTCA
TAGTTACAACTTCGTGTAACTTTTTAAATTGTTAATATAAAAATAATCCGCTTTTTTTTAAA
TATAATTGAATTTAGAAGTTTTTGAAGATTAGTGAAAGAAGAAAGAAGAAGACTACAAGA
TAATTTATAATGCGTGAGTAAAATACGTTTGTACAGAAAGAAAAATTCTTTTATAGTGAATG
TTTATGGTGATATTATGGTAATATTTTTAATTATTTTAATTATTGATAAAAAAAAAGTAGTG

TTTTATTCTCAGAATATAACCAAATTTAATAAATTTTAGAAGTAAAAAAATGGAGAGAATA
ATTAAATTAAAAAAATAGAAATAAAATTCAAGATTTAATCATTACTTACGTTATATTCTTTC
CATTTATCATAATTTTGTCTCATAAATCCCACTATCTCCGGCATATTGATGAATACTGTAAAT
AAAATTGTGTCCATGATAACAATACATACGTTTATAAGCATTCAAGATATAAGAGTTACTG
ACCATCCCATGCCTCGACCATGTCGTTGATAATGAAATCGATAAAACCAATTTGCGCTCTTG
GTATCGAGCATGTTATTCTGTCGAACATTGGCATCATTACTGGAAGATGCAATTGTTTTTCTT
CGTCAGTCTGAAATAAATTCAATCAAATGCATCAAAATAATCAAAAACTCTTTCTCATTTTG
AAGCATACGTTCAGTACAATGTGATATATGTGAACAATTTTTTAAACGTGTAAACTCACAGT
AATCTCTAGTCGAGAAAGAATAATTGTCAAATTAAAAAGAAATCTAAAAAGAAAGGAAGG
AAACGTATGTGTTATTATACGTACTTGACTAAAATACTCTTCTGCAATTCGTCTGGCCCATTC
GACGCAATATTTCAACGGTCGAGTAGGATTCGACACGTCCGCACACTTGATTATCATTCTCT
TCACTAGTATTACATTCTCTGGCAGTATCGCAGCTGATAGATCTAAATCCTGAGAACCCTGT
ACTCAGATTTTTAGAGAGAACGTTTATAAGGATTCAAGTAGAGATGACTTTGTAATAGATTT
ATTTAAAGAAAAAAAAAAAGAAAGCCGAGATAATTTGGTGAATTTGAAATCTATTTAATAA
CAAATATAGGAGAAAATGTAAAAGTAATCGTTACTTCGATATACATCTCTGGAATCCTTGT
GCTGAAGGCATTCATAAACTTGGCGAGATGTTCAAAATGTTTCGTCATCTCGGTGGCTAGG
ATCATGTCTATCACACTTTGGCGAACCTGTTTATAAGTATCTCTTTCCAGATTCTATAAAATT
TTATTTAAATTTTGTTTTACTTTATCCTTTTCGCGATTCACGAAGATTAATTCTTGATTTATGA
TGAAAATTTTTGATACTCACTTTAAATATGTTCACGTTATCGTCCGATAACGTTAATTTAAAT
GTTAATGCTGCGTGATGCGATTCAAGAACGGTCAAATCGTTGTAAAGGATCGCTAGTTTGTT
ATCAGAATTGCAGAGAAATTGACTGAAATCGATTAAATGATTATTTGATATTCTAGAAAGA
GTATAAATACGATATAAAATAATTAAATTTTGGATAATTGTGAATAATTATTATTCTAATAT
CGATCAGTCGATCGAATTAATCGGATTTACCTCGATCTACCGGGATGATCAATATCGTGAG
CAGCAGCTGCGATCAAAGTTGCGACTTCGTCTAAAGGTTCCAAAACCTGTTTCAGCCTTTCC
GTTTGCATGAACATAGCAGCAGCTTGCAATACGTCTGCAGCGTGTGTTGAATTATGATAAC
GATTCGTAATGTCGTAATTAGCCTCTATCACAATCAACCAATTTTGTACAATTTTCTCCTCGC
AACCGAGTCTAGCTGGGACGCGAAATAGATTCATGATGGTCATTCCCAAAAATAATAGCGC
TCTAAAAATCGGAGAGAGATCGATCAATTATCAGTTCAATCATTTATCGTTAAATTTCACTA
AGAGAAAAAGAAAGAGAATAACGAGTTACGTATAAATTTTTCTTTCATAATCGAATACCTT
TTCTCCGTCAATATCTCTAATTTGAATATATCGAAATCCCATTCCAAAGACTTCTCTAGAATT
TCTGCAATTTCTTGAGGACCCCTGTAACCTTTGATCTGCATACGCGTGGAGGCAGCTAACGT
TTTTGCAGGGGTTTCTCGAACTGATTCTATCGATGGTTTCCTAATATCCCATGATTTTTTTGGC
TATATCGAATAACGTTAAAAATAAAATCGAAATCACAGATGATGATGATGATGGATGATG
AATTATGGTATAAAAAGAGAGAATTTTTCTTTTCAACATTACCGTGATCAAGGCCTCGATGA
AATCGGTCGCTGGATCACCTGGCTCTTTAAAAACTGGTGAATAAAGTTCGGTAGTTTTGAGA
ATATCAATCGCCTGCATTGTAATTAATTGATCCATATTAAAAAAAAACAAAATATACGTGG
GGAATAGAATTGGTGAAAGGAAATGGACGACATATGATAATTGATATTGTGATACCTGCTC
GATTTGTAACCTCGTCTCGGACGATGTATCTTCTTTAATCGAGGCCGTAAGGAGGGCGATCA
CTTTTGTGATGGGTGCTTCTAATTGCAAAGTTTGTAAATCACGTTTACTGAGCCGTCTGCGA
ACTGAAAATTAATTTTAAAAACAATTGTTAGAACACGATGATAATTTTAACTTATCGTTTCG
ATATGGCAAATAAAAGGGAATGAGAAAAATTTAATAGCTGATGTTCTATTTTCTTTATCGA
GTATAAACACTATTAAAGTGACGAGTTAATAAAAAAAATTGCAAGTGTTTGTAGTTTTCAG
ATAACCTAATTTTTAGCCTCGAGTAGTTTATCGAAACCAAAAATAAAATAAAATAGTTGGA

TCGAGTTGAAATATATTACCTTCTTGAATTCCTTTGAAATTGATTTCGGATACTCTTTTTTGA
GCTATAACGACAGTTTTTGGATGAGTGATGGGCTGAAGCAGGGGCTGAATAGTTTCTGACA
AATATGTTGTATCGTATATGAAAATATAATGAGTCGGTTTTCTGAAAGAAAATGTTCAAAA
GCACATGGGAGGACGATAAGTAAAAGAAGGACGTATTTTTCTTTTTTTTTTTTTCTCCTTTTT
TTCTTTTTAAATGATAAATCTAATTTGACTCACTTGAAATAGGCGAAAGGTATCACCCTGCA
ATTAATTGTTAACATTTGATTGTTTCTTCGAATACAGTTTATATTCCCATCATATTCTCGGCCT
TTGACTATATGTTGCTCCATTTGAATAAAATTATCATACAAAATTAAATCTGTAATGTTTTTG
CCCCAAAGTTCATTAATCCTGTAGCCTAATAATTTTTCACTAATTTTATTGGCAAACTGTAA
AAAGAAACAAGAGATGATAATAAAAATTAGAGGCAAAATTATTTTTAAAAATAATTGGTT
GAAATTTTACTTAAATTACAATAAAAGAGTTTTGACAGAAATATCATGATAAATATTTGAA
ATATTACAGATGTTTAATTATTTATCCTCAATAATTGCAATAGTAACATTTACCTGTATAATA
TATTTATCGTCCGTCACATGAATCATGTCACGACATCTGTCAAGAGCCATATACAGCATGTT
GACAGCAACCAGTTGTGTCCTCGGTAATATTCCGCTAGTATAAATTCCTACTAATTCGTTTA
TTAAGATTCTTTCGTGAGCGCATTCCATTAAAGTCTGAAAATGAACTCAAGATAAATACAC
GTGAAAAATCAAGTTTCGTCTTTCAATCTTTCAAAGATATTTCAGAAGAACTTACTTTTGTGT
ATCCTATTTCCATTAAATCCAATGTTACGATTTTTTCACTTTCGCTATGCATGAGATACCTTTT
ACATATACATATATTGATTTGTAAAATAAACTCGAAGATCGATTGAAAGGAAAGAAGAGT
AGTTATAAAAGATATTCGATTCAACTTACGATTTTTTTACAAGAGCTATAATTATGGAATTA
TAATAAAAAGGACTAGCCCTGATTGCTCTGAAACGAATATATTTATTAGTAGTATTTTTATT
TGTACTTTCGATTAATGTGATTAACATTTTTTTTTTTTTTTTTCTAATAATTTTCCATACCTACA
AATAATGTCAGCTTCCGCAGCGCGTTGCCCCCGATGATCGATGATCACTAACTCGTGGCCTC
GAGCCTGGAAGAATTCGAGTGCTTTTTCCGCGTCTTTCGCAACTGACACGCTCCATCCCAAA
CGCCTCGAAGTAGTGGCAAGGATATCCATTTGTTGATCATCTTTGGGGAAGACCAGTAATA
TCTGAATATGAATAATTGCAATAATCGATAAGATATATATATATCAATTCATGAATGAATTC
TAGCATCGATAGATCGTAACTTACTTTTATGAGACTAGGAATACTGTTCGTTAACGGATGGG
GTTCAGTATCGTGGGATCCTATCTGAAATCAAATCAACACGCAAACTTTGAAATATCGAGA
AAAATCTTGATCCTCTCGAATTGTTAATTATTATTTCCTTCGTTAAAAATTTTCTTCTTTTCCA
TTATTTTTCCTTCCCTCCCTCCCTCCCCCCTCCTTCCCCTCCTCCCTCCGTATTTTTTAACAATA
TTGCTAATCAATTTTGCAACGTACTACTGTCTCGCTATCACGTTCAAAACGTGACAATGGCT
TGTGAAATTCAGCGTATTCCCGCTGAACTTTGGCCCGCCGACGTGCACGGGCCGATTCTTTT
AAATTTTTTTGTATATTCCCATCTTGATATTTCGAGTTTTGCATTCGTACTCGACTAAGTATCG
CTTGGTTTTATACGGTGTGGAATTGTTGTATAACCTATACATGTTAGGAACACGGCAAAGAG
ACAAACGAATCTGAAGTTGAGGCGTAACAGCGAAATAAGATGAATTTATTTCTCTTCTTTG
ATAACGTTATTCCGAATCTCGATAACATTTTCGATCAAGACGATAATTTTCTAAATTCAGCT
TGAAAGAATATCGTCGTAGATGACTTTTTGTTTCTTTGTTTGTATCTTTCTTGTTATTTTTTTTT
TTTCTTCGAGATGAAGATTGTCCAATCAAACGTTGGATACGCATCGTGCCTTTTATTCTGAG
CGCATGAAACGTCCCCAAGTACATAGGTACAGTTTGATCAAGAGATGTTGAAATGAATCTC
GAATCATCCTCAATCATCTCTTTCGAACGCGTATCTTGAACAAAGGGGCATGCTTGCGCAAT
GATGAAACACGAAGAAACTTTGTTGAGAGGGAAACTTTTTCGTAATCGTCATTAAAAAAAA
AAAAAAAAACGGGAAGAAAATTATAAATTCAAAATTTTAAATGGAAAGATCAATTGCAAA
AATATTTCAATTTGTAAATATTGTATACACATCTATTGTAATCGCAATAAAAATTGTTGTTGT
ACGATTGTATCATTTACTGCCACGAAAAAAAAAAAAAGAACGAAGACGATAAACCGATAAA
AACAATCTATATAACTAATCTATATATAACCAGTTTACCGTTTCGTAATGCAATTTATTCATT

ATTCCACGATACGAAAATACGAATATAGAAAAAACAAAGACCTAGTACGAGGCGAGCAG
CACTTCATCATCTACAATGATCCTCTTTCGAATTGACGAATAAATCGACCACAGCAGTAAC
GAAATTCTAAGTAAGTGCAACCTATAATAATATTTCTTTTAATTTCTTTTAGTTTCGTTTTTAT
CTCGACGAGAAATGGGAGAGATTAAATTAAATCGATAAACGAGTGTTTTTATCGTGTAAAA
TTTTTCCAGGACGAATAAAAAAAAAGAAAAAAATTATTATTGCTCGTGATTAACAAAAAA
AAAAAACAAAAATATGTTGCAATATATATATAAATATACATCGAAATTTACGAAATGGATG
AACAAATTTACAAACGAATAATAAATTTCACAGATCGATATTGCGATCGATTCTTTCATATA
AATGGATCTTTAATCGATTTGGAAAGATAGCGGATCGTACTAAGAAACTTTCAATTACGTTT
CACACAACTTTTCTGGTACCTGTGTCACCGACACAAGCGACGGAGGTATCTCGTTTACGTGT
ATCTTGTCAGTCTTCCATTTCCTTCGAAACGGTTTACACAATATGTTGGAGATCCAATTGTAC
CACGATATAGATTTCTCCTCCTCTTCGATCGAGATATAGATCGGTTTCTGCGTCGACCTCCTC
TTAATCGTTTCCTCCTCTTGGTTATACATTATTATTATTATTATTATGAAGATAACAGAAAAT
TAAAATTAATTGACACAAAAAACGTGGATGATGGACGAGGCACTAATATAGATCGATTCG
ATCAATAAAAACGATGATCAAACATAATTAATAGTGAATCGGTTCGTTCGATATTTTTCAA
AGGAGAAAAAATGATTAAAAATCGTGTCCCTCATTGTTCATCGATTTGGGTTTTGAACTTGG
AACGATGAAAGAAGTGACATGTTCGACGATGCAATTTGACGAGATTGATAAGTTTACGGA
AAAACGTCCATCGCGATACTGAATCTATATTATCATTGTTGGGAAGATTATTCCTCTTTATTA
TCTATCTACTCTAGCAGCGGAACATGTCCACGATAAGAAATTTCTTTCAACAAGAGATCAT
CTCCGTCAGATTATCAATAGTAAGAATTAGATAATAACTCGGTCGTCACAAGCAGATGATT
AATTATAAATAAAGCGATATCGTTTTTTTCTTTTTGTTTATCGAATTGTTGAAAAATATATCC
AACCAGATATCATTGTAATTTTTCACGATAACAATTGTCGATCTATAAAAATTTTATGCCAT
TTTAGAGAAAGAAGCTTGCCATTTTTAAATATTATCGATTAATTCGAAAATAATTTCGAAGA
AGAAAAACTCGTATACAACGAACTCTCTCCCCTCGTTGAACTGAATTTCCAATGATGAATA
GTGTATGCTTCCAAAGTACAAGAGAAAAAAAAGTCTCTGAATTGAAAGATTACCCTTCATG
ATAAAAAGAAGGAAAAAAACAGATTCGATAGAGAATATATAACTAACATTTACTAGTACT
ACGACTAACTATAAAATAACAAACTTCTCAGACAACAGAAGCATTAAACGAGAAACTTTC
CCTCGCTGAATCCTACCTTTCGTTAATTCGCACGATAATATACAAACTGAGCTGATATATCG
CGGGATAAAAACTTGACTAGAAATCTATTTTAAAAAAGCGGTCAGACTTTTAACAGACTTC
AACTGTGCATCGCGTTTAAATCCTGACAAATCGATTTACGGCAAAAGAAAGGTAAAAGTAC
ATGAAATATATGAAAGACTGAATTTTCATTTTAATAATTTGAATAAAATACACGGGTAAAA
ATTTCACGAGAAATATTACGTTGATTTCACCAGGGAAAAACAAGCATGTACGAAATCCCAA
ATTTTTCCACAAATTGTGCAAGATACGAGTTCTTCCTCCTACTCCTCCTAGGTCGCTCGTTCC
AAGCTCTTCCGGCTATAGACCGTCTATAGATCGTCTATAGATAACAGCTTATACCCTGTCCA
TCTGTTTGATCGAGCAAGAGAAGCTTTCAACGTGAGAGAAGGATAAGAAGAAAGACGAGA
GAAAGAGAGAGAGAGTGAAATAGCGGATGGCGTTTAATAATTCGTCACGAAGTGGTGAAT
CGGTCAGCCTGTTCAAATATTTAGGACAGGTTTAGACGGCAGTGTCGTGATGATACTTTAGT
CAAGCCCCTTCTTGAAAATTCCATGAAAACATTTGATGCAAGTTTAATGAGATCACTCGAC
ACACTGCGAACGAGCCACGTGTTCGGGGAGGGGAAAAAAACAGTATGCAATTACTTCAAC
ACAATTACTTGAGGAAAGGGTTAATTATCGGGTAATCGAATGCTCGTGAGAAAGAAAAAG
AATGACGAATCGATTCTCCCAATTTCGCTTCTTTCCCTTCCCCTCTTTTTTTGTTCCACAGACC
TTTGAACTAGTTTTTCCCTTCCTATTTCCTCCTGCTCATTCGATGATCGATAAGCTAAACTTTT
TGGAGAATATTGGAGAAATATATGGAGCACTGAGATAAGATTGATTAATAATGAATGAAG
AATTGGAAAAATTGAATAGAGAAGAACATAGTACAAAAAGTATTGAATTTGATTATAAAA

ATTGTTCAGTAATTGAATTGATTAATAGTTTCTTTTTTTTTTTTCTTAATTAGATTAGATAAAT
CAGAGAACAAGAAAGATCGTAGCATTACAAAAAAAAATAGAGGTCAGAGTATATTTATCCG
AAGTAATTAAATGGTTCAGAGTGATATATATATATATATATTAATAAAAGCGAGCAGAAAA
AGAGAAGGAGGACACTTCACTGATGGAAGAGTTTAGAGTCCAAGAATAGCAATCGATCAG
AGACGTTAAGATATATTTGAAGTAGCTTGAAATAGTATCAAAACGAATGAATAAAAAGAA
ATGAACGTAAATGGATTGATTAAAAGAAAAAAAAAAAAATTAATAACATATATAAAAATAA
AATACATATAATCTAAATAATAGAATGGCAAGCATATCCGAACGTAATATTAATTAATCGT
TTACCAATATTGAATAATATTGAATTTGGATTTGGATTATTAAATTTAATCTCATTGGATATA
TATACATTTTGAAAATTCATAGAAGTAGCGATGAATTGCATCTATTCAATTGCCTCTTATAT
ATCCTTATGAAAAATTCAAAAGTGGTACATTGTTTGTCAATTTTTATCTTCGATTGAAACCA
CGAGAAATCAAATATAACGCGATACGGCGAGATACTTTTAAATGTCATTTGTCGTTTTACA
ACGTTCTACCGATTACCGATAGTAGTCCATCCAATAATTATAGTTTGCTTTCACATCTATCG
AGAACTTTGCTATTTTTAACGTTAACGAGGTTGCAAACATAGTGGAAAATATATTGAACGA
CCTCTTTTTTTTTCTTTTTTTTTTTTAAATTAAGAGATTATCCATTCTCTCGATTATATATATAT
ATATATAAAAGGGACAAAACAGAAATTTACTTACCAACGTCGAATAATCTGCATTTTTTTC
ATCGCCAACATCCTCGAACGACAATTTTCTTCCCAAAATATCGTACTCTGTCTTTCCTGAAA
AAAAAAAAAAAATAAATAAATAAATACACACAATTGAACGATGATGCGTTATTTTAAAAGA
AAAAATTTCGAAGATAAAAAAAAAATTAATATCGAACAGTATTTATATAATAATATTTATAT
AGTGTGTCATTCTATTGCAATAAAACGAGACTTTTTTTTGAATCGATTGCAGCAATTAATCG
ATATCTTTTCAATAGTGAAAATCGTTTAACAATCGGATTAATCGAATGGATCGTCACGGTTT
TGGTTCAATAAATCAACGTTGAGGCTGACAATTGGAAAAAATAGTAATTTATCATTCATCTT
TGATATCGAATGAATTCATTGGACTGATTTCTTTAAATAAGTCGTCTGTAAAAAAAAGAAA
AAAAAAACATTTTTAATTTCTCAATTAACTCTTCGTGTAATACCTTTAAACGTGTTATCGATA
ATTGAATTAAAAGTTTTCGATGATGACTTTTCTTTTTTTTTTTTTTTTTTTTGATAATGCTTAA
TTGAAATGGATAATTAAGAAAGAATTCGAACGAAAAACTTTATAAAAAGAAATGGAAAGA
GGATGTGTGAACGAGGTCGATCCTTATCTTTCACCTTTTACGGCGGGATCATTCAGTGTTTAT
CCGATCGATTCGAATGGAAGTTCAGAATTTAAAGTTAATTGATCGAAAGTAGGACGCCTTA
ATTGGACAACCTGAGAGTTAATAGGTACGAAACAAGCGTTAAATATTTTAAATCGAAGCCG
AGAGAATTGTAAATTTTTCGAGCAACAATACAAATTTTATTAATCCTTAAATCGATCTTCAA
TTTTCTCTCCTCTTGTTGATTTTTAAAGAAAAATGAAACGAATTATTAAAAAATATTTGCTTA
ATTTTTATTCCGAAAAATAAAAAAGATGGATAAAACAATTTTTTCTTTCAATCTTCTTGGTTT
GTCTTCAAAATTTTATGACAATATTTCAACTAGATTGCT
ATTAAATTTTATTATAATTATAAAAGGATCATAAAATTATTACAGAATTTTTTTTCAAGAGA
ATTACATACCATTGTCAAATATTGTGTCATCCAAAATATTTTGTTCCTCGTTTGGAAAATAAT
CGTTCGAAGGAGATGCCATGTGAATTTATAAGCAGATGAGATTATGGAATCGTAGTATCGT
GATACGATTATTAAAACGTATACAAAAGTTTAAGTCGAATCAATAATTGAAAATTACAGAT
CAAAGATCGATACATATTTAAAAATCAATCTAATATACGAATATTAACAATATTATAGTGC
AAAATTATTCCATCGAAAATATGAATTTTTTTTTTTTCAAACTAAGAATATGAGCTTTGATTA
CATTGCAATCTCAATCTTACATCATATCGATTAGAGTATCAGCTGTTTATTGATTGAATCATA
TTTACTAGATAAGTTTAAAAAGAATTTAATCCAAAAATAGGAAAAAAAAAAAAAAGAAAA
ATAAAGGAAAACAAGTCTGAAAAATTTAAAAATCTATACACTCCACTAAATTTTCCTTTTG
ATCTCCAATATCTTCCATTCCTACACTGATCATGCTTATCTATTACTCACGATGCACTCGAAA
AATAAATAAATAAATAAATAAATAAACTAGAAAAATATTCAGAAAAATCAATAATAAGA

AATTCCAATGTTTCAAATATTTTTATAAACGATTAATAAATGTCGAGAGAAATCAAAAATT
GACAGATTTAGACAGTTAATTTGTATTGTAACGATGTGTTGTAACCGACTCAGAGACAACG
ACTGGTCGACAAAACCAAATTGTGCAGGTTAAAATTCTACGCGTGAGTGAGTGGCCTCAGG
AACGAGATAGGATCGTTGGAGAAACTCGTGATACGATGATCATGTTCGTCAAACGGGAGC
ACGTTTCAAGGGAACACGAATAATGAATGAAAAAAAAAAAAAAAAAAATATCAAAAGAAAA
GTAAAATTTTGTCGCAAAATTTAGAGACGCCTCGTTTCTCGCAAAGCAAAAGTGCTTCGAG
TTTATGGCTGGTATTGAATTTGTATTGAATTTCTTTTCTCACTCCATAGACAGCTTTGAAAGT
AACAGTTTGATGCGAGGCAAACAAATTATATATATTGGTGCTAATAATATTTTTTCCTTTGG
CTGTTTAAAATATTTGCTTGATATATATACATCATACATATATATATATATACATGTGCCTGT
TGTTTCATTTTCTCAAATTTGAATGTCGATTGGCTGAGGAATTTAGTATTACCAAATGGATCG
AAATGAAGATTATCATGGAGATTAGTTTCCTTCTGATAATTGTACGTTAAAAAGCGTGTAGT
TTTTTTCTTTCTTTTTTTTTTTTTTTTGTAAGATTTTAGCGCTTGTAGAATAATTAAATGGAAGC
CTACTAAGTTTGAGTAAATGTGATCGAAGATGTATTCGCTGATTTTTGGTATGTATAGAAAC
GAAGATGATGTTTACAATTCTTCACAATTTCTAGAAGAAAGTCTACTATGGTTTGAGAAAA
ATTACCGAGAGATTTTTTTTTATTAATTATTAAAAATTATGCTTATGAAGAAAAGAATAGAC
GAAATTAGAAATTTTTCTTTGAACAGAATACGCACAAATTTAATAAAACACTCGATGAAAC
TATGCTTCCAAACCAAAAAATAATGTTCAAAGCGATAATTCAATAATTGAAAAGAATGTAT
TCAAACATAGTCGAGACCGAATCTAGCACACTAGATTTAAAATTGCAATAGCTGTTCAAAT
TATCCGAACAAAATCATCATTTCATTGCTTTCTAAAAATGAGCAAAGGCATTATTGCCATTA
ATCCTATCTGAATTCTGTTCAGTTCTATTAAACTGCACTTTGGATAAAAAAGATATCTGTCTG
TATAAAAAGATAAAAATGTTGTATGAAGTTTTGAACTTATTCCTATGGATCAACTTTCAGAT
CGAAAAAGTAAAAGTAAATATCTCCCGCAAGATTACGTTATCAGTTTTCGAGCAATTTACG
TATCGTGCACAATATTTTTCCTTATTCTATTCGCATTTAACATGTATATAACTGGCCATTGTGT
TAAATGTAATGTGTCTCTAAATATGATGTCTCTTACACTTCATATTACCTTTCGACTGATTTTT
CTTTTCTCAAAACTGCTAGAACCAAGATCTCGCCAATCTATGATATATAAATAGTGAGCTTT
GATATCTTTGATTTTTTTTGTTAATTGAGTTTCAATAACACGAGTACAAATGATGATTAACAT
ACGTAGCTAATTTTGCTTGCACGCTCGCAAAAAGAAACTCCGTATGGAATTGCCATCCGGC
ATTATCTGTGTCTGATTTGCATTCGTAACCAACACGTATCCAAATATGTGTGACTCTTTTATC
AAAGAATCAATATAGCGTTTTATTCGATATTATATCTCTGTGCCATCGAGCTTCTTGCTTACA
AAAAAAAAAAAACGAATTTCAATGGGAGACATGAATGTGATTAAAGATTAATTGAAATTTG
AATATTGATAAGAAGAATAAAATTCTGATCCAATCAATAGAAATGAATTCAAATGTTATCA
GGAATTTTGTGAATGAGTTTTATTATTTATATTAATTCCATTTAAAAAGCAC

75. >MSTRG.1443.1 Gene=MSTRG.1443
CGATAACAATTGGCCAAGCACCGATTATTTTCAAGAACGGTTGCGCGTAGAACGCTTGCTT
CAAATCTTTTTCCTTCGTTTCCATTATTCCTTCCCGATGATTGAGAGTTGATGAGATTGAAAT
TGAACATCAACATACAAATCCAGGTTGTGCCATTTGCAAAAAACGTTTCCATTCTTCTATCG
GTAATCTAGGCTACACCAATGCATCATTAAATTGAGAAAGGTGATCAAATTATAGACATTC
CAATTTCTGGTTTACCTTTTGATCTTCGTTTGATATTCCACTATGTCCACAATTCTCTTCTGCA
TGGTATTCTCGCTGGTAATCGATCCATCCGCGAGATCGATCATTTTTCTTCTTAAAATCTTCA
AAAGACTGCACATATGGAGAGAAAGCATCAAGGAAATTCCGATAGTGCCACTGAGAACTG
TATAAACGAAAACTCCAGCCAGAATTTGCAGCATAAAAATCATCTCGTAGTATGGGCTAAC
TAATTCGTTAAAAAAGGAACATGACCAAAATGCACGGAACAACGATACACATTTGCAAGG

AAATGGAGAAAGATATTATAAACCATTTTTGTATCTTCGAGCTCAAAGAAGATTCGATAAG
AATTGGCCAAGCACCGATTATTTTCAAGAACGGTTGCGCGTAGAACGCTTGCTTCAAATCTT
TTTCCTTCGTTTCCATTATTTCTTCCCGATGATTGAGAGTTGATGAGATTGAAATTGAACATC
AACATACAAATCCAGGTTGTGCCATTTGCAAAACGTTTCCATTCTTCTATCGGTAATCTTAA
ATCGGTAATCTAGGCCACAGCATCATCAAATTGAGAAAGATGAGATTGAAATAAAATGAT
CATGCAAATCCGGATTATCCCACTTGCAAAGCGATCTTTCTATTGGTATAACTTCGAACAGA
CCGCAGCAGTGCATCGTTATAGAGGCAATCGAGCTGAACACGTCCAAAATGCATGGAGCC
ACGATACATATTTGCAAGAAAAGATAGAAAGAAATTATGAACCATTTTTGTATTTTTGAGC
CCAAAGAAGATTCGATAGCGATTGGCCAAGCACCGATTATCTTCAGGAACGTTTATGAGAC
TGAATAATGTCAGCGATGCGGCCACGCAAATCTCGGTTTTTTAAAATCGTCTCGATCCTTCT
GTTGGTGACCTCGAACAGACCGCAGCAGTGCATCGTTATAGAGGCGATCAAGCTGTAGATC
CCATAATTCAAAGACATTATTAGGAAACTGGTGAACATTTGCACGACGAAAGTTATCTCGT
AAGTTGGCGAGTCTTCGATGACAAAGAAAACGTAATCGCTCAAGTATGGCAAAGGTATCTG
AGTACTGTTGTCCGCTTTGACGATCCTCTCTGATATGAGGGGCAAAATAACGTGATAGGGC

76. >MSTRG.1443.2 Gene=MSTRG.1443
CGATAACAATTGGCCAAGCACCGATTATTTTCAAGAACGGTTGCGCGTAGAACGCTTGCTT
CAAATCTTTTTCCTTCGTTTCCATTATTCCTTCCCGATGATTGAGAGTTGATGAGATTGAAAT
TGAACATCAACATACAAATCCAGGTTGTGCCATTTGCAAAAAACGTTTCCATTCTTCTATCG
GTAATCTAGGCTACACCAATGCATCATTAAATTGAGAAAGGTGATCAAATTATAGACATTC
CAATTTCTGGTTTACCTTTTGATCTTCGTTTGATATTCCACTATGTCCACAATTCTCTTCTGCA
TGGTATTCTCGCTGGTAATCGATCCATCCGCGAGATCGATCATTTTTCTTCTTAAAATCTTCA
AAAGACTGCACATATGGAGAGAAAGCATCAAGGAAATTCCGATAGTGCCACTGAGAACTG
TATAAACGAAAACTCCAGCCAGAATTTGCAGCATAAAAATCATCTCGTAGTATGGGCTAAC
TAATTCGTTAAAAAAGGAACATGACCAAAATGCACGGAACAACGATACACATTTGCAAGG
AAATGGAGAAAGATATTATAAACCATTTTTGTATCTTCGAGCTCAAAGAAGATTCGATAAG
AATTGGCCAAGCACCGATTATTTTCAAGAACGGTTGCGCGTAGAACGCTTGCTTCAAATCTT
TTTCCTTCGTTTCCATTATTTCTTCCCGATGATTGAGAGTTGATGAGATTGAAATTGAACATC
AACATACAAATCCAGGTTGTGCCATTTGCAAAACGTTTCCATTCTTCTATCGGTAATCTTAA
ATCGGTAATCTAGGCCACAGCATCATCAAATTGAGAAAGGTGATCAAATTATGAGATTGAA
ATAAAATGATCATGCAAATCCGGATTATCCCACTTGCAAAGCGATCTTTCTATTGGTATAAC
TTCGAACAGACCGCAGCAGTGCATCGTTATAGAGGCAATCGAGCTGAACACGTCCAAAAT
GCATGGAGCCACGATACATATTTGCAAGAAAAGATAGAAAGAAATTATGAACCATTTTTGT
ATTTTTGAGCCCAAAGAAGATTCGATAGCGATTGGCCAAGCACCGATTATCTTCAGGAACG
TTTATGAGACTGAATAATGTCAGCGATGCGGCCACGCAAATCTCGGTTTTTTAAAATCGTCT
CGATCCTTCTGTTGGTGACCTCGAACAGACCGCAGCAGTGCATCGTTATAGAGGCGATCAA
GCTGTAGATCCCATAATTCAAAGACATTATTAGGAAACTGGTGAACATTTGCACGACGAAA
GTTATCTCGTAAGTTGGCGAGTCTTCGATGACAAAGAAAACGTAATCGCTCAAGTATGGCA
AAGGTATCTGAGTACTGTTGTCCG

77. >MSTRG.1457.1 Gene=MSTRG.1457
AAGTGAGATGTAGTGGATTGATGTACAATGAAGTGAAATTTATGGTTTCAACTTATATTTTT
TTTGTTTGTTGTTTTTTTATCGTTTCTTTTATTTTTAAATGAAAACAAATTGACAAGTTAAATT

AATATTTGATAAATGTGTCTTTTTAAAATTGTTTTTAATACGAGGAATAGTGAGATACTTCC
ATAAGAATATCCTACCTGTTCGGCAACCAGATCGCCTATATAACAAAATATGAAGATATTG
AAAGTGATAGATATGATTAAAGCTACCCAGCTCGACAACACCTGCATCTGGCCGCAAGCAT
GCACCGCGAATATAGCAGCCAAACTACAAGCTGCAGTAGTTATGGTATGCAATAAAACAC
CGGCGACCAATTGAATGCAAAATACAATCTCATTCGTAAAACTATAGCGAGTGTCGATGAT
GAATCTCGAGAAGGGAAACACCAAGGGAATGAAGATATCTTCCACTTCGAGGATCACGTA
TAAACTGCATGGTATCGAGAGGAAACTTATTAGACTGTAGCAGATTACATTTATTAATCGAT
AAAGATATTTTCCTGTCGTTGAAATGGTGGAGGAATTGGGCCAGAGACCGATCGGTTTTAA
AATCCAGCGACTCCATTGAATGCTCAGCCAGTTCGACAACACTTGCATCTGGCCGCAAGCA
TGCACCGCGAATATAGCAGCCAAGCTACAGGCTGCAGTCGTTATCGTATGCATCAAAGCAC
CGGCGATTAATTGAATAGAAAATACGATCTCATTTATGAAACTGTAGCGAGTAT

78. >MSTRG.1495.2 Gene=MSTRG.1495
GAAAATCAAAACCGAACGTGGTATCATATCCCGTGTTCCATGCATCCACGATACATCGATT
CGATACATCAATAAGAAGGGAAGCGATGCGGAAGAAGTTTTAGCGCGCGTCATTTTTCCAG
AGGATGAATTACAACTGCGACGACGATAGGCGAGGAGAGTGTTTGCTCTTCATCGAAGATG
TGCCGTCGTATGTATGTTGACACTGTTGGAGAGAAGGTTTGTTGGATTATTGGTGACTAACT
GACACTTGTTGATTCGAGAATTATTGTTAAATTTCCTCGTTTATCTTTGGCCATTTCGACGGG
ATAACGCGAAAAGACAATCTATTGGATATACAGACAATTCTTACGTAACGCAAGAGGATT
ACGTTTATTTTTTTAATAAAGTGTTTAAATGGAAATGATTAACGATTCGACTCGTTGCTCGGG
GAAGAATTATATGAAATCTTTATCGAAGTTTCCTCCTTTATCTCTCTTATTCATTTCGATAAA
CGAACGCGTGAAAAAAAAAAAAGACAATCACGAAGATATTCTTACGTAACGCAGCTCCGG
AGATTACGCTTATTCCCTCTTTTTTTAATCCCCTCGTTTATCGTTCCCATCCGATATCTCGAAT
CACAAACACAGGTGACAACGCGTCGTTCAAATAACACGAGGCTCGACGGGTGTATATCGG
TCGCGAGATGCAGGTAGTCGCGCATTGTTTGTATGTACACACAGTGAGGGTCCACGCCTTTC
CGCGACCATTGACTCATCAAGGCGAAAGGCATCGAAGGGATTCAGCGAAATATCAGGCAG
GCCCCAGATACACCGCGGGTCCTAACCCAAGGGACCTAACCCGAGGGCCGACCGACCCTC
CCCCGACCGATCAACTCCCTCGCCGAGTTGACTCGAACGCGGTGTGTGACGGACCTACAGC
ACCAACAACAGGAAACGAACCCGGATGACTCACCGTCTCGCTCCGAGGAAACGAAGCTAC
GAAGCATCGTGCACAGAAACTGGAACGCAACCAACTCTTACCGCGGTTACGCGCATCGCA
TAATCTCGTTACCGTCTTCTCCTCAGCCGGTCACGCAATCGTCCACGAAATTCACGTGCCTTT
CGTGGCATCACGTGTCGCAATCCGTCCGTGCAATCCTTGACAAATGGTATCAAAAGTGGAC
AGGAAGTTTCGATTCTTGATTGAGAGTTGATTAACCCCCTTTCGGTTAAAAATTCGATTGGC
TATCCGTCGTATTTGAAAATTTTTTCGGGTATAAGTAATGAATTATTATATCGAATTATCAGT
AAAGTTTCTCGTTTTCTCGGGTACAACGATCGAGCAAAAATGCATCCTCCAAGTTCCTTAAC
CTCGCGTTTCGATTATGGGGCGATTATGTGTCTGTTGTTCTTCGCGGAAACAGGCGTCAGTTT
CTGGTCGAGAAAATTCTCTCCGAAGAGGAGGAGTGCTTCGACCCCCACGGGAAGGAGTTTC
GTTTTTGTTAACAAGCCCGCGTGCGTTTCGGCCGGAAGGGGCCGGAAGCGAGCGAATAATT
TCGCAATAAGGTGTTTCTAATCGCCTCGCAATCTCGCGTATCCTCCTCGGCATTTTCGTGCAC
GTGGAATCGTAATTAAGAGGAGAGGAGAGGAAGTGGCCGGTCAGGAAACGAGAGGACGA
TTTTTCACGGCCCTCTCTCATTGTTTGTTGGCGATAAAACCGATTATTCTCGCGATCTTTTTTC
GCGCGAGGAGGGAACGAAGGGAAGACGAAGCGGCACAGGCCGACAATTCGCTTAAATGG
AACCAAGAACACCATTTACTCGTTTATCGAGCGTTCGCCGTGCACAACTCGTGCGATCTATT

GTATTACCCGACCGATGAGAAAATTTCATCGTGTTATGGTGCCGTTGCGTGCCACGCCGGCC
TCAATCCTTTTAATGGCCATCCCCTATCATTTAAATCCCTCACCGGGAATGGTTTATGGTTTA
TAAATGAAGATTGTCTTCGCCTCCAACTTCGTTTCTAATATTCCTCGCAGAAGTGAGAAGAC
CGATCGATTCAATTTCAATCTCTAATAATTCAGGGAATTTTTAATTTTTACAATTCCATAAGA
AACAAAGCAATCAATATTTAACGTTACGAGGAATCGTTTTCATTTTGCAAAAATGATTACG
ACGCGAAAGAAAACAAAAAAAAAATAAATAAATTTTCCAGAATCGAAGGGGCTAGATCTT
CGACGATCTTCGTCGATTACGTCGAAAGAAAGTCGCGAGAGTAGAAAGATCCATAAATCG
CTGCGACAGCGGCGCGACTCGCAGGCCGGACAGGCATAGAGAGCCAGACAGTCGCCAGG
CAGACGGACACCAGACGGAGACGGGAGAAAGCGAAGAGGAGGAGGATTTCTGTCTGGAA
ACAACAGAATCGACGACCAAAAGCAAACTCGGCGTCCATGTTCCGCCACCTTGAGACTTG
AGGCGACTTCCGTGCCCTTCGGGTTGCGCGAAATGGGATCCATTTCGCGATTTCCTGCGAAC
ACGATCCGCGGAGTAGAAAAGCTTGGAGGCTGGAGGAAGAGGCTGGAGGAGGAGGAGGA
TGGAATACAATGGCGCGGATCGTGGCTGCCGCTTATGCCCTTCGGTTATGGGGAACGCACG
AATTGTTAGAAACCGTCGTCGCCCTGCTAGGTGCAGCCGAAGGTTCAGCTGGAACCGGGCC
AGCGGCAATTCCGGATGGAAATCTCATTCCGAGGCGGATATCTTCGTTGCGTCGAGATTGT
ATCGACCACGGTAGACAAGGACTAAGGTCGCGTCCGACTCCTACGG

79. >MSTRG.1495.4 Gene=MSTRG.1495
GAAAATCAAAACCGAACGTGGTATCATATCCCGTGTTCCATGCATCCACGATACATCGATT
CGATACATCAATAAGAAGGGAAGCGATGCGGAAGAAGTTTTAGCGCGCGTCATTTTTCCAG
AGGATGAATTACAACTGCGACGACGATAGGCGAGGAGAGTGTTTGCTCTTCATCGAAGATG
TGCCGTCGTATGTATGTTGACACTGTTGGAGAGAAGGTGACAACGCGTCGTTCAAATAACA
CGAGGCTCGACGGGTGTATATCGGTCGCGAGATGCAGGTAGTCGCGCATTGTTTGTATGTA
CACACAGTGAGGGTCCACGCCTTTCCGCGACCATTGACTCATCAAGGCGAAAGGCATCGA
AGGGATTCAGCGAAATATCAGGCAGGCCCCAGATACACCGCGGGTCCTAACCCAAGGGAC
CTAACCCGAGGGCCGACCGACCCTCCCCCGACCGATCAACTCCCTCGCCGAGTTGACTCGA
ACGCGGTGTGTGACGGACCTACAGCACCAACAACAGGAAACGAACCCGGATGACTCACCG
TCTCGCTCCGAGGAAACGAAGCTACGAAGCATCGTGCACAGAAACTGGAACGCAACCAAC
TCTTACCGCGGTTACGCGCATCGCATAATCTCGTTACCGTCTTCTCCTCAGCCGGTCACGCA
ATCGTCCACGAAATTCACGTGCCTTTCGTGGCATCACGTGTCGCAATCCGTCCGTGCAATCC
TTGACAAATGGTATCAAAAGTGGACAGGAAGTTTCGATTCTTGATTGAGAGTTGATTAACC
CCCTTTCGGTTAAAAATTCGATTGGCTATCCGTCGTATTTGAAAATTTTTTCGGGTATAAGTA
ATGAATTATTATATCGAATTATCAGTAAAGTTTCTCGTTTTCTCGGGTACAACGATCGAGCA
AAAATGCATCCTCCAAGTTCCTTAACCTCGCGTTTCGATTATGGGGCGATTATGTGTCTGTTG
TTCTTCGCGGAAACAGGCGTCAGTTTCTGGTCGAGAAAATTCTCTCCGAAGAGGAGGAGTG
CTTCGACCCCCACGGGAAGGAGTTTCGTTTTTGTTAACAAGCCCGCGTGCGTTTCGGCCGGA
AGGGGCCGGAAGCGAGCGAATAATTTCGCAATAAGGTGTTTCTAATCGCCTCGCAATCTCG
CGTATCCTCCTCGGCATTTTCGTGCACGTGGAATCGTAATTAAGAGGAGAGGAGAGGAAGT
GGCCGGTCAGGAAACGAGAGGACGATTTTTCACGGCCCTCTCTCATTGTTTGTTGGCGATAA
AACCGATTATTCTCGCGATCTTTTTTCGCGCGAGGAGGGAACGAAGGGAAGACGAAGCGG
CACAGGCCGACAATTCGCTTAAATGGAACCAAGAACACCATTTACTCGTTTATCGAGCGTT
CGCCGTGCACAACTCGTGCGATCTATTGTATTACCCGACCGATGAGAAAATTTCATCGTGTT
ATGGTGCCGTTGCGTGCCACGCCGGCCTCAATCCTTTTAATGGCCATCCCCTATCATTTAAA

TCCCTCACCGGGAATGGTTTATGGTTTATAAATGAAGATTGTCTTCGCCTCCAACTTCGTTTC
TAATATTCCTCGCAGAAGTGAGAAGACCGATCGATTCAATTTCAATCTCTAATAATTCAGG
GAATTTTTAATTTTTACAATTCCATAAGAAACAAAGCAATCAATATTTAACGTTACGAGGA
ATCGTTTTCATTTTGCAAAAATGATTACGACGCGAAAGAAAACAAAAAAAAAATAAATAA
ATTTTCCAGAATCGAAGGGGCTAGATCTTCGACGATCTTCGTCGATTACGTCGAAAGAAAG
TCGCGAGAGTAGAAAGATCCATAAATCGCTGCGACAGCGGCGCGACTCGCAGGCCGGACA
GGCATAGAGAGCCAGACAGTCGCCAGGCAGACGGACACCAGACGGAGACGGTGA

80. >MSTRG.1517.4 Gene=MSTRG.1517
ATAAGATTCACCCTGGCGCACTTTCAGCACGCAGTTCCGCGACGGCAGAGATTGCGGCCGG
GGGTTGCAAATGGGGTGTCAACGGCATCAGGATACTGGAATAATAATCGAGAAGTGATAA
TCGCAAAGCCACAATGGATGGGACGAAAAGCGAGCCGAGCAAGAGCGGGGCTGTGCAAC
GGGAGTGCGGTGGTTGCGGAGAGGTAATTACGGAAAGGTATCTTCTCAAAGTGTTGGACAT
GTTCTGGCACGAGGATTGCTTAAAGTGCAACAGTTGCAATTGCAGACTCGTGGAAGCTGGT
CCCTCGCTTTACATTAAATCTAATCTGATATTGTGCAAGAAGGATTATTTAAAATTGTTTGG
ACTCACTGGCCATTGCGCGGCCTGTAACAAAACCATCCCAGCCTTCGAGATGGTGATGAGG
GCGAGGACGAACGTCTACCATTTGGAGTGTTTTGCGTGTCAACAATGTAACTACAGGTAAG
AGG

81. >MSTRG.1537.1 Gene=MSTRG.1537
CTCGTTGCTTTGTCGATCAATACTCGGTTTTTGCTTATTATTTTCGAAAAAATATGGTAAATC
GTAATGATCGTATCATTGAATAATAATTCATGACACAAGGAACACAATAATATTAATAATT
GTCATATATATGATGGAAAAAGTATTGAAATTACAAGTTGAAAGATAATGCGCATTAGATG
CCGGGTGCCGGTACAAGTTCTCGAACTTTCAGCACTGCGACGCCGCATGCCTATATAAGAG
AGCCGGCCGGCTTGCCTCGGCGCATTCGTAAACAAACTTTCGTTCGGTTTGGACGCAGTGA
ACACCCGAAGACCCACTAACCAGTGAATATACCTCTACTTCTTTTACGTGGTCCACGAGGC
TGCTGACAATACGACGTGAAAATGTAGACGAGACGGATTCGGTGCGGAGCGCGACAGTTT
ACCTGCACCCTGCGGGGTCGGTGCTTGAACAATCCGTACGCAACCATAATCTGATTAGAGA
GTTCGAAATTTCTTCACACATTTGAAGTATTTAAGAAAAGCACTTCGAGTTATATGCAGTAG
TTTAGAGCAGATTCGTAGTTTGAAGTCGAATAAATCGTCGGCTTCGTCTTGTCGGAACGAAT
ACTCTACCACAAGAACATATGGAACTTTGGAGTCGAGAACGAAAAATAAAATCAAAACGG
AACGGGAAATAAAAGAAAAAAGGAACGTAAAAAGGCTCGGAAAAAAAAAAACGGATCA
TTTTCCTAGCGTCGAGCAGCATCGAGCGAGTCCTTTGCTCGGGACTCACCACAACCACCAC
CACCACCACCACCGAGCTCTTCTCTCCATCTTTTTACCTTTTTGGGTGCCGTGATAGTTAAAA
CACCATCTGAAGATAGACTCGAGGTAATGTTGATCACGTCGTGAGATGGAGGCAAGACGT
ATCTGCGTATGAATTGTCGGCTGACAAATCCGTGCTCGTCTTGTCTCTCCTCGTGTTTTGCTT
CGACAATAACGTTATTACCGACCGTCTTCACCGTAATCTCTTCCGGTGAGAATTGTTGAACA
TCCAGTATTACCTGTGGAGATCAGAGCAGAACAATTGAGACAGGCCCGAAGTTTAAAAGG
TCGAGCAGGGAGATATCTTTTATTGGAGCACGATGTTACGTGTAACGAATATGATATTTCAA
ACAGGCAATAAGAGAGATCTGAACGACCGTTGAGAAAACGCATCCATTCCACATCCGATT
CTGGCCTCGCATCTTATAATATACCATTGATTCTATTAAATCATCATTTATTGCGAAAATTTC
AAATATCAAGCTTTTTCATCACCGATTCAGGGAAAGGCTAATTCTTTTGAATTAACGCCGCT
TTAAGCAAATGAAGTTCATCTTCGCCTGCTATTGAAAACCTACGTTTAGAATTTAACACGTT

AGGCAGTCTGTGTCTATATTTCGAAATAACAGATTTTTCCTTCTCTGAAAGATCCCTAAATG
TCCAGTTATTGGATATAAATTTGCAGTCAGCTATTAGCTACAATACGCACAACCTTTCTAAA
AATCTTCTGTAATAATCACTTTAATAAGTATTTGACAGAAAAAAGTAAAACAGTGAAAAGT
ATAAAAAAAAAAAATCCGTTCTTCCGGTAAATTCGAGTTATCCATATGCAAAATAGACGAGC
CACGAGGCTCGTTGGCGTTTAAAAAATATGAATACATATTAGAAAAAAATGTAGAAAAGT
AGTTTGTTTTTCTATTTTCTACTGGTTGCAAAGTTTCGCTTATTTCCTTTTTTTTTTATACATAC
ATACATATAAATATATATATATATATATCTTTTCATATGACATCTGCGTTTTCTTATGTTGATT
CTAATATAGAAGTAACCGTTGTGACGAGCAAACCGGAAAGATGTAGTTAAAAAACAAGCC
TACTGTAACATCTTTTCTCTTTTTTTTTCTATTCGCAGAAAAGACAAGCAAAATATGCAAGTA
GGTGGAATGATTTTTTATTATTGAGCAAAAACAAACAGAATTGCTGTGTTGAGACTTTATTT
TAAAAACAAATTATCTTGTGATTAGCAGTTCCAATTTAAAGAAGGTTGTAAAAAGGAGAGA
AAAAAGAAGGAAGATATAAAATATATAAAAGAAAAGCCGTGATTTTTTCGGGTGATTCTTA
TATGATCCCAAAATCCGATGAGACTTGAAAGTTAAAAATAGAAAAAGAATCTGTATAGTG
ATGTACGAAACACCTTCGCTTTCTTTCTTTTGAGCAAAGTAGCAAAAGACCAATAAAAACA
GATTTTAAATCTCCAGTGCTGCTGCATATTGTATTACAGAGAGGGAGAGAGGGAGAGAAAG
AGAGAGAGAGAGATTGCGCACTTGCATTTTTGTACATATAGAATATTTCGTATTACAAGAT
ATTTGAATCGTTTTCTATCGTTGATGATTCGAGAAGCATTTATTTTTATATAGAGATTATTAT
ATATAAAAAAAGAGAGATCCGAAAATTTTAAAGGAAAGCCGAATTTTTTAATCCGAGAGC
GGCGCCCCCATTTAAGGGGGGTGCTCTGACACACACCTCACACTTTCTCGGTTCTTTCTGTA
AACAAAGAGAAAAAAAAAAAAAAAGAAGACTGATGATACTTATGTGACACGATCCATTTTT
CGGATTTAAATGAGAAGAAAAGTCGTCCTTTGAATATTACAAGATATTTGGCATCGTTTGTT
AAACGGAACAGAAAATCCTTTCGTGAATTAAAAAGAAAAAAAGTAAAAAAAAAAAAGAA
AAAAAAAGAAAAAAAGAAAATGGCGAAAGCCTGTTAAACTGTGATTTTTAGGAAGGAATT
TCCGGTAAAAAAAAAAAAAGCGTGTGTCTATCGGAAAATAATCGCATGTACGTTCGGTGTGT
GTAAAACATTTCTTTTGTTACAAAATTTTAATAATTTTTCGCTACTCGAGTCATTTTGAAGAA
AAGTAATTTTCGAGACCTGACTGCATCCTACCAAAATACCCTTTAAAGCCCCCTCTTTGAGG
GGGGGGGGTCTCATTTACTCAAAACAAAGAACCAATAACTAACATATATAATAATCCCGA
AAAACGTTCTGAATCTTTACACCACTACGCAAAGCTGTGAACCATTTGAAAAGAGAAAGA
GGGGGTTTTACGGTGAAGAGAGATCGATTTTTAAAATTTTTTAAAATTCTTGTACGACAAAC
AAGATAAAAAGAAAAAAAATTAATTATGCAGCCGGCAAAGAACATGCTAAGGATAAACA
GCAGCAGCAGCAGAAGCAGTAGTAGTAATAGTAGTAGTAGTAATGGCAGAAACAGAAATC
GGTTTTCGGGCCTTCTGGTGTTCCAGTTCTTTCATTCATTTCGTTAATTATATTTCCTGAAAAT
TGTATTTTTTTTTATTATTATTATTATTTCGTTTCTTTAGTTATAATTGCTTTTAATTCAATGGTT
AAATCGCTTCGTTATAATAATCTTGCGTTAGAACGTTGCATTGAAATTATTACTTTTCTATCA
TAAAGTATTTTAAGATATTTTCTATTATAATATTTTTTAGTTAATTTTTTTTTTTTTTTTTCATT
TGGTTCCAGTTTCCCTTTTCTTATCCTTGATAAGCACTTAGACACACACCCTCTTCATTTGTTT
CTCTTTGTATTCCTTTTACGAATGCGCTTTTTTCTTTCCAACTTCCTTTACACCCTTTCCTTACA
TTCCTAACTAAATTTCTAGGTCTTGAAAATAATATCGATACAAATTGAAATTTCGAAACACG
AAATTTTTTTTTTTTTTTTTTTCCATTCGGTTTTTTGTCCTAGACACGCGTATATATATACATACA
TGTAATACGTTCCGTACTTATTGGACGACACCTATGACAAAAATCTATAAACAACAATAAA
AAAAAAAAAAAGGTTATCACTTTAGATTGTCTCAGCCTAAGTTAATCGATCATTAATCCGC
GAAATAGTGGAACAAATATTTCGCTCACTGTATTAAAAAAACTCCAATTGCACGATATTAT
GAAAAATAGAAAAAATGGGCCAGTAAATGTTTAGCCTGCTCTATTCGAATGTATTAAAATT

ATCGAACGTATTTAGCTTGTGGCGATATAAAAAAAAAAAAAAATATATTGTTACTTAAAA
AGCTTTCTAAATAATTCCTCGAACTAAGAGTTACTAAGTTACAACGGTTACTTATAAAGCGC
AAAGAAATGAAATAACTTGTATTTGCCGTAGAAGAAAGTAATTGGAGTAAGAAAGAAAAA
TATAAGAGAAAAAGATAATCGAGTATATGCGGTGTAGTTTAAAAGTTCAGGCGTTTTATCC
AGTTCGAATTGTCATAAATACCGATACGTAGTAACGACCAAAAAAAAAAAAAAAAAATAA
AAAAAAAAAACTTGATCTTCGAATAAGCTTTGTAATAAAAGTGTGTGTATATATTTATACAT
AGTAATTTTTAAAATTAATTTCTAGAAGTTTGCCTTTTTTTTTTCTTTGCTATTTATTTTCTC
AATTCTTGAGACTGTTCTTGATCGAATTTTTCCTTATATCTTTCTTTTCTCGAGTTTGTTTTTTC
AACTCGTTTCAAGCAACTTTCCAAATTTAATTTAACGCTCGACAGTATTATTATTATTCGTTA
TAATAATTCGTTTTCACTCGATTCCAAAAATCAATCTCGTTAATAATTAACTTTTTACAGTTA
ACTTTTACAGTTTAAGACCAACAACGACTATCCCATCGCTTTCTTGCTTCCTTGCACGAAAG
TCGTGGGATAGTCGTTGTGAATATATTTGTGGTGCTATAATAATGATACAAGAATATTTAAG
GTTCGGTGAATTTCCAAAATCCAATCAAATAATAGCAATAAAAAAATTATAATAGAGCGTT
AAATTCCAACAAATTTCAAGCACAGTGGGTGTTATAGCAAAGTTGCTCCAAGTCGTGTCGT
TTATATATCATTCGCATATATCGAGCTTCGAAAAGCGCTCCTATATCTTCGAAATCTTTTACA
AGAAGCTTCATCCAGGTTTAAGGAACCAAAATTGTCTTTTAGGCAACTTATAATATTAAGTA
TCTTCCTTTGACGATAATAAGCGAAATCGAGGAATAACGATCAAAGTTTTCATTTTGTAAAT
CATTTGAACAAAGCACTTCACTCTCTCTCTCTTTTTTTTTTGTATTATTTTTTTTTTTTGGCCTA
ATTCAATCAACACACCACATTAATTTTCAATTTTTTTTTTTTTTTTAAATTCTCCTCTTCTATA
GGATTCCATACGTGGAATTCTATGGAAGACGAGAAATAATCTATCTTCCTTGCAACATACG
CTTTTCAGCCTATGATGTAAAAGTAATATTTTCCAATACGAAATTCTTGAAGTGAGAAAAAC
CTTAATCGAAGGCAAACTCGGTGATTGATATCGAAAAGTCGTGATCATGATCGAAAGGTTC
CCTATTATAATAATACAAAGTACAGTTTGAGATCATATTTTGTACATCGCATTCAACTATAT
AAAGGTTCCCTAAAGTTGTGTAGAGTGAAATCGTACGAGTAAAACGTAAAATTGAAAATTG
CGCGTTAATTATGCGCCTCGAACAACGATTTTGTGTATAACGACGTATATCTGAGATTGACA
ATTTCCAGAGATCATCGAACGAGTCTCGAATGAATTGGAATGGAAAAATAATCGAGTAAC
GATGAAACGTATCCTCCATATTCGACACACGCATAGGTTACTTGCTTAGCCCTAAGATTGAC
GTTTCCGTCAATGGAAGGAAATTAATACTAACGCACAAGTTCGTTTGTTGCAAGCAAAATT
TATATGAAAATAGTGATAGTATAATATACTCGTCAAGTTATCTGAGAAAGTGTGCCAATTA
ATAAATTGTTCCGTTTTGAAGTATAAAGTATTAAAATTTAAATAAATCGCAAAACGATTATC
CTGTAATTATCAGTTTTTTGGTGGTGACATAATATTAGGATATAATATCGATTATTAATATTT
TTCGATTCGACAATACCGAGTAATACTTAATTAACCGTAGTCTTCCAGGAGATCACATTTGA
TCCGTATCCATAGTTATCACTCGAACCAAACGGTACTCGTAACCATACCTATTGTCCACCGA
CAATTGGTGTGGTTCGATACCATCGAGTGATAACTATGGATATAGTTCGCGATTCTCTATAA
GCAATAGTATGTCAATACTACAAGCGAATTAATCGACTCTTGAAAGATGAAGAAACGACA
ATACTAGAAACAGCAATATGTCTCCACTCCACTGATTCGCGAATTGATAGCGAAAAATTAA
AAAAAATCAGAACCGAATCTATATCCACTTTCATCATTGGAATCATTCTTCTAATCGTCGCA
CGAAGAAATAAATTATATGACAAGTCGAATAAAATCCAATAAGATCCAACAAGAATCTTG
CAACTCATTCGATACACATGCATGCATCATCATCCCTTAGGGCTTTGCAAGTCTCCGAAATT
CAATTTGTGATAAGAAAAAAGGGCTAGTGATCAAGTTGCACGATTTTGTTTTTTCGCATGTT
CGTTGAAACATCCGAGAAGATGATTGCCGGGGTAATTTTAACGTGTCTTTTACCAACCTGGA
AGTTGTCCTTATTGTCGAGTTGAATCGTGCTGGATCCGCTCGAGGGTTGACCTGTCACGTTA
CTCCATGGACGGTAATACCTATCGCGAAATATCGAGCGTAGAGGTCTATCGAAGTTGATAT

CGGAAAACCGTGATAACAAGTCGTCTCGATTCAGTCCTCTGCCGAAGTGTTGGTCCATCAA
TCGGGACACTGGACGTTCGAAATCGTCCCACCAATCGCGAAATATCAGAGGCACGACCGA
CATCTTAAAATATTTCTTTTGTTTTCCTCTCGTTAAAGAAGAAGGTAGATCGGAGGGAAAAA
AGGGTATACACGGTTCAGGAATACGAATTTCGACTGATTCAACTGCATAAACGCGAGAAG
CTTAAATAGGCGCACCCTCGAAATGCGTCTTCAACGCCTACGGATGAGAGAGGAAAGAAG
GAGAAATGAGATCCGAATTTTCGGATACTCCCACAATGTTTACGTCACGTTTACTGACTACG
TCCAGAAATATCATCCGATGAACGTATGTAAATGCGAGGAGGGGAAGATTTTCTAAGAATG
CGTAATTCAAAGCTGTGATTCAGTGTTTTGGATAATTTTGGCATGTTGCACATGTGTAGGGA
ACTTTTCCGTTTGCTTTCCAAACCTCGAGGAGACATTGCATCGCTGTAAGTCTCTTTATCTAG
AGAAATATTTTTCTTTTAATAAAGCAAACTTTCATTAAAATTTCGAGTTTTCCTTCGGGGAAG
AATATATATTTACTAAAAAAAAAAAAACCCATACACATACACACATACACATACAATATTAT
TACATCCTGATAATTATTCAATGTCAGCCTTCAATGTTATACGTCATGATCATTGCTGCAATC
GTTCACTTATCCTCCAAATGTCATTAGAAATGCGATTCGTTCCGATCGTACCTGCTGGTGAG
GAGTTCCATGCCTCTAACAAACGAACGTGTACATGACGATTAGAGGATAATTTCATCCGAT
TGTGCGTTAAACGTAGAGAGGGAGGAGATTGCCCTTTTTGATTTTTCACGTATACAAAGTAT
ATCAACGTTTGAAGAATATTCAAATGTATTTTTGGCAACATTCGAGAATTGTTTTCACGAAC
TTCTCGTTGTTAACTTGCTTTTTCGTTCGAGACGAACTGAACAACTTGATGAAAGAGATAAT
CTTCCAATGTATTTATAACTCCCCGTAATTCCTCCCCTCGATATCGAGAAAGAATCTCCATG
CCGAATGTAATGTATATTAACGTGCAGATTTTAAAAT

82. >MSTRG.1537.4 Gene=MSTRG.1537
CTCGTTGCTTTGTCGATCAATACTCGGTTTTTGCTTATTATTTTCGAAAAAATATGGTAAATC
GTAATGATCGTATCATTGAATAATAATTCATGACACAAGGAACACAATAATATTAATAATT
GTCATATATATGATGGAAAAAGTATTGAAATTACAAGTTGAAAGATAATGCGCATTAGATG
CCGGGTGCCGGTACAAGTTCTCGAACTTTCAGCACTGCGACGCCGCATGCCTATATAAGAG
AGCCGGCCGGCTTGCCTCGGCGCATTCGTAAACAAACTTTCGTTCGGTTTGGACGCAGTGA
ACACCCGAAGACCCACTAACCAGTGAATATACCTCTACTTCTTTTACGTGGTCCACGAGGC
TGCTGACAATACGACGTGAAAATGTAGACGAGACGGATTCGGTGCGGAGCGCGACAGTTT
ACCTGCACCCTGCGGGGTCGGTGCTTGAACAATCCGTACGCAACCATAATCTGATTAGAGA
GTTCGAAATTTCTTCACACATTTGAAGTATTTAAGAAAAGCACTTCGAGTTATATGCAGTAG
TTTAGAGCAGATTCGTAGTTTGAAGTCGAATAAATCGTCGGCTTCGTCTTGTCGGAACGAAT
ACTCTACCACAAGAACATATGGAACTTTGGAGTCGAGAACGAAAAATAAAATCAAAACGG
AACGGGAAATAAAAGAAAAAAGGAACGTAAAAAGGCTCGGAAAAAAAAAAAACGGATCA
TTTTCCTAGCGTCGAGCAGCATCGAGCGAGTCCTTTGCTCGGGACTCACCACAACCACCAC
CACCACCACCACCGAGCTCTTCTCTCCATCTTTTTACCTTTTTGGGTGCCGTGATAGTTAAAA
CACCATCTGAAGATAGACTCGAGGTAATGTTGATCACGTCGTGAGATGGAGGCAAGACGT
ATCTGCGTATGAATTGTCGGCTGACAAATCCGTGCTCGTCTTGTCTCTCCTCGTGTTTTGCTT
CGACAATAACGTTATTACCGACCGTCTTCACCGTAATCTCTTCCGGTGAGAATTGTTGAACA
TCCAGTATTACCTGGAAGTTGTCCTTATTGTCGAGTTGAATCGTGCTGGATCCGCTCGAGGG
TTGACCTGTCACGTTACTCCATGGACGGTAATACCTATCGCGAAATATCGAGCGTAGAGGT
CTATCGAAGTTGATATCGGAAAACCGTGATAACAAGTCGTCTCGATTCAGTCCTCTGCCGA
AGTGTTGGTCCATCAATCGGGACACTGGACGTTCGAAATCGTCCCACCAATCGCGAAATAT
CAGAGGCACGACCGACATCTGAAACAGTTTAAATATAGATATAAAAAAAAAAAAAAAAA

AAATCGGTGAATATCGGTCAGTTCTTACTTTTTCGATTCTTTTTCGATAAGACGGTAGTATAA
ATGGATAAAAGTAAACGGAAAAGAATTAAGGAAAAGGAAGATGGAGACGTTATGTTAAG
TATTATTACCTAGTTGAACGTGTACTCACCACGATTTATAACACGCGAATGGAAGTTTGACA
AATTAAAAGTTAAGCTGTAAGCAATTTTTACTTAGGAACGTGCTAAGGAGATTCTTTTGAGC
TTAAAATCTTAATCCAACTAAAATCAATTTGCACGCCTGTCGTCACAGCTATTAAAGAACA
CGATTTAGATTTACTTTGGACAACGGTTGCAATTAGTAATAATCTTGATAATGAAACGCA

83. >MSTRG.1556.2 Gene=MSTRG.1556
CTCGAAAAATATGTCACGGTATGTGTTCGAAACAACGAAAGAAAGTTTCTTCTTCTTAGATT
GTTCGTGCGATGCAGATCGTGAAGAGAAGAAAAGCGCCAGTCGGCAAAAACTTAAACGAT
TACTTAGTATACCTTAGCGAACGTTTCTTTTCCCTTTGGGTGAACGTCTCGTCATTCCCGTGA
TCGTGGAAACGACTTGTTACCACGGTAACAACGATCTCTAGTGGCGTCTTCTCGTAAAGATC
TCCGGACTCGCGGGTACAAACTTATCATTATTCCGAGCGATACGTTTAAGCGAAACTGTAG
TCGGCATTCCCGTTTCTTTTTATGGGGTGGGATGCAATGGCACGGCCTAAAGGGAGTCTATC
CTTTTTAATTCACGGCTGTAAATATATTAAAACGTTTGTGATAGAATCGTGTGTCTTACGTTA
GGTTTTACGAGAATGAAATTTCTTTTATCGTTGATTGTGATATGGATATATATATATATATGT
ATAGTATATATATGTACATTTTATCATCGTTACGATAAATTTTATATAAGATGGCACTATCGT
TTATTACATTGGCGATAATATAATTGACTAAAAAGAGTAAAGAAAACTTGAAAATTGAACT
TTGGGAATTTTAAGCTTATATTTATATTTCGAGTTTCGTTTTTTTTCTTATTTAATTCGCTTTTA
AACCGTTCATTAACCAACCGTCCGTTCGTCCGTCCAAATGGACCAGGAAATTCCAAGATTA
ATACGATCCGAAAATTGTAAGGAGATAAGACGTTGTTCGATTAACGATGGAAATCGATCAC
AAAGCTGTGAGTCCACATTCTTTTACACAACGACAGTGCAATAACATGCTAGAAAAATTTT
TTCACCCGCGTTTACGTCGGTCAACCGCAACGATGTTCCTCGACATTCTTTCTCTTCATAACC
AGTCTGAACGCTTAGCTTTAATTAACGATCGGTGTTAAGTAACAACGAAGCGGTTGCTCGC
GGATCACTTAACGCGCTTG

84. >MSTRG.1599.1 Gene=MSTRG.1599
CATTTTTGAAAAATGTTTCATCTTTTGATGCGAAAAATTGAACCAGTGCTTGTTCGACATCTT
CTTCATTTTTGAATTTTTTGACGAGTCCCTAAAGCAGCGTGTGGTCTGGCGTTATCGTGGTGC
AGTATCATGTCCTTCCTATTGAACATTGCCGGCCTCTTTTTTTCGACCGCTGTCTTTAAATTAT
CCAGTTGCTGACAATACTTTTCCGAATTGAGCTTTTCGTTCGGTTTTAAAAGCTCATAATGTA
TTGGTCCTCGAATGTCCCACCATATACACAACATTCTCTTATTCAGAGTCAAATTTGGTTTAG
AGGTGGAAGGGCTAGGTTTTCCGGGTTCACAATATGCCCTTTTCCTTACAATGTTGTTGTATG
TAATCCACTTTTCATCCCCAGTTATCATCCGGTTCAAAAAGGGCTCGATTTTGTCCCGAGCC
AGCAGAGATGTGCATATGACAACTCCATCGTCCAAATTTTTCCGACTTAATTCATGTGGCAC
CCATCTTGAATACTTCCACACCAATCCTATCTTCCGAATATGGTCAGAAATGGTTCGTTGAG
CTGAATTAAGCCTTTCTGCGATCTCCGATGTTGTCGGAAAAGGATCTTGCTTCAACATTGCC
TTAACAGCATCGTCGTCGATCAAAGATGGTCGGCCAGAACGTGGCTTATCAGAAAG

85. >MSTRG.1603.3 Gene=MSTRG.1603
ACACGTTGCCGTTGTAAAGTATTGAGAATAGAATACAATCACGATCTAACAAAGAGTGTCA
CAAAGTATTTGCTTGAGGGACATCGAAAAGAAGTAAAACCACGAGAGTCATGGAACTGGA
GCCTATGTGTATCGCTGAAAATGAGACGTATTACCGATTATATCGTTTGGGCACCGGTATCG

AGCACGACGTCCCTGAACTCAGCATTTTAACTCGTCAACTGCGCAGTTGACACAGCTCACA
CGTCGCGTAGCACCGGAGGGATCTCGCGGTGATCGGAAAGAGGGGGGTAAGAGGGGACG
CAAGCCTGGAAGGAAGGCGTCCGACAAGACGGACATGAAAGCCAAGCTTGAACGAAGTC
GACAGAGTGCGAGGGAGTGTCGTGCTCGCAAGAAGCTAAGGTATCAATATCTCGAGGAGT
TGGTAGCCGACCGCGAAAAGGCTGTGCTCGCACTTCGAAGGGAGTTGGAGATGTATCGGC
AGTGGGGACAAGAATTGGACGCCGGACGAGTTCCCGAAGGGCTACAAGCGATGCTGGAGG
AGCTCGGCTCTCTGAAGCGGGAGAAAGTGACCAACTAACCGAGCGAGGAAGAGAGAAAG
AGAAACACGAGTTTCACGAACATCGAGCTTTTTTCGCCACATTTTCTTTTTCTTTTTTTTTTTT
TTTCTTTTTCCGCGATATCCGATTTGATAATCGAACGTCATTTCCCTCGTATTTCCCGATTATT
ATATTATTATATTTCCTTCGAAGGGCGTGTCGAGGATTCAACTCGAAGAATTCGGATGCCTC
TTCTTCTCGTTCACGGTGGAAAAAACCGTTGAAAAAACAAAGGAAAATGGATGGAAAAGA
AATATCATCCCACCTTACGGATTGCTGTCCCGCTTTTTAGCTCTAGTCCTCTTCTCTCGTCTCG
TCCAATCCTCGAAGAAGAAAAATTCGTTCGGTGTTGTTGTTTCTCTAGTTATTATTATTACTA
TTATAACGTTTAACGTTTTATCCTTGACGCCTCACGAAAGAAAAT

86. >MSTRG.1638.1 Gene=MSTRG.1638
TTCTCATCTCTTCTGCATCAGTCTTCAACTCTTTCCTCCGCGATAACTTGGTGTAAAACGATA
AAATTTAACGAGCAACAATCCATCAAAATCGTTCGTAATTTACGTGTGACGGGATGGCGCC
GTTACGTCTCATTTTATTCGTCTTGTTTTTGTGCATTTTCGTCACCATGCACGAATCCAGACCT
CTGACATTTCCACAAAGGAAGAACACAACCGCGAAGAAATGCCCGCCAGGATACAGATAC
ATTCGCAACGTGGGATGCAGGAGGCTCATATTCGCTCAAACGTAAAACGTAAAATCCGGAT
AATAAGAAACTTTTGCAAAGTTTTCTGAAAGTGACTGAATTGCACTAAACAGAAACTAATC
AAAACATCTATTCTTTATATCGTATATATTCGGATATCTAATTTATAATTTAACAGTGACAAT
GAAATAAATAACAATTTATCGAT

87. >MSTRG.1740.3 Gene=MSTRG.1740
AATACGTCAGTAGAATTTAATCGCCCGTAGTAACAAAAAGGTTAAGTCGCGATGACTGCTG
TAATAGTTTCCAGTCCCGTGCAACGATTTCGAGCGACACGCCGCGCGAGAAATTATTCGTT
GGGAGAGAACGCGAAAAGATTCGAAGCCGGAAATTTTAATTTTCGAGAGACATCAGTGCA
CGGAATAAGTTGGACGAGTTCGACGAGTCAGAAAAATCGATATTCATCCGACCGAAATAA
ATCCGATTTACGTAATAAAATTTGAAAAAAAGAAGCACACCTAATAGCAATAATAATATAT
AATAAGACTCGTATCGAGGGGAAAGAAACAGATGCGCGAAAGTTGAAGCGACGATTTCGA
CGTGGATTCAAACGCTGGTGACTAGACGACTAATTCGATCGAGAGGAGGGGGCTTCTCGTT
ATTATCTCGGAGAGGGGATCGATCGAAGGTGGGATGGATTCGGGTGGAGGACAAGACGAT
ACTGTCGTTGGGCCAGCGAACGGTGACTCACTCGCCCCGATTCTTGGTCACGTTGGAGAGC
GCCAAGTCGAAGAATCAAAGCGAGTGGAGGGACGAGGAGGAGGCCACCTCGCGGCTTCA
CATCCGCCAACTTCGGGAAGCCGACCGGGGATGCTACATGTGCCAGCTGAACACGAAGCC
CATGTTGAGCCAGCTGGGATGCGTGGATGTCCTAGGTATGCCTAATTGCGCGTTATACCGCA
TTCCTACGGCCAGATACAGTCGGGTGTTTGCGGTCGCTCGTGTGCACACACGTCACGTACGC
ACGCACCGGTTTCGATTAGCCTATCTCAGAGAAGGGATGGAAAATATGCGCGCCTCGCCGC
TAGAATACGTTTCAATGGGACCCCTGTACCACCATGCCTCACCCGTGTTGCTCGCCCGCCTC
GCCTCTATTCGTTTCCACCATCCTCCGATTCCTACTTCCCTCCGACTTCCCCCCCCCCC

88. >MSTRG.1740.4 Gene=MSTRG.1740
AATACGTCAGTAGAATTTAATCGCCCGTAGTAACAAAAAGGTGGGATGGATTCGGGTGGA
GGACAAGACGATACTGTCGTTGGGCCAGCGAACGGTGACTCACTCGCCCCGATTCTTGGTC
ACGTTGGAGAGCGCCAAGTCGAAGAATCAAAGCGAGTGGAGGGACGAGGAGGAGGCCAC
CTCGCGGCTTCACATCCGCCAACTTCGGGAAGCCGACCGGGGATGCTACATGTGCCAGCTG
AACACGAAGCCCATGTTGAGCCAGCTGGGATGCGTGGATGTCCTAGGT

89. >MSTRG.1753.1 Gene=MSTRG.1753
TATCGCTGAGACGAACGTGTCGTGTGCCGTCGCTATCGCCACCGTGGTCGTTGTCATTATCG
TAACCGTATTCCTTCAAGTAAATTTTGAGAATTCAACACGCGAATCTTGGTCAGTTTCTCTTT
ATCGGTTAAAGAGTGCAAACTTTGATGAAATTCTCTTTAAGATATTCGCGTTGAGTTTAATT
CGATCGAAATAACGCCCGAGCTCCCGGTCGTGGAACACAATCGTGGTGAGAAGGAAGTAT
ACGCGTAACAGGGGCTACGGCTACGCGTAACAGCGAACTGGAACGGTGGAGTTTGACAAA
AGTTTGTGTTACGGCCATGCTTGAAACGGCAAAACCTTCGCGAGTGTGAGCAAGAGGGAA
AGAGGAAAGACAAATTAAGAAAAGTATAAATAAAGAAGAAAGAAGAAAGGATAGGAAA
AGGGAAAGAAAGAGTGAAAGGGTGTATGTGAGTGTGTGCTAAGAGAAAGAGAGAGGGGA
GGGCGAAAGAGAAAGAAAGAGTGCGAGAGAGAGAAGGAGGAAGAGAGAGAGAGAGAG
AGGGAGAGAGAGGGAGAGAGAAAGAGAGAAAGGAAAAAGAAAAGGGAATAAAGAGAA
ACCCGGAAGAGAGAAAAGTGATCCGAAATATCATTGACGGGACAAGACGAAGCAATCAG
AATCGTCGATCCGTTTAAAAGAGAAATCGTCGAGAAGATTCGCGAGGGTGCGAGCTGACG
ATAACCGTAGTATGAGAGAAGAGCGGCACGCAGCGAGAAGGTCACGGAGGGAGGTTGGA
AAGAGCTCAGTGCCGTAGCATGCCGGCGAGCTGCAGCGGGTGGTGGGGCGTCTCTGAGAG
AGAGCGTCGATCGGCGTCGTCAGGCGTCTACTCGGGCGTCTGCGTGGTCGAGCAGCGGAAG
GGTGCCTACGCGAGTATAACAGGAGGCGGAGGAGGAGGAG

90. >MSTRG.1762.1 Gene=MSTRG.1762
TCTATCTTATATTTTGTTTATGACTATAATATTTCTGAAATCGTTGAATATTGAATCACAGTG
CAGACAACCTTTTTGTGTTCAACGTAGGACATGAACGACGCTTCACATCATTACAATGTAA
AGATTACGGAATATTAAATTTAAAATCAATTCTTAAACGACATGAAATGTCATGTAATTAA
TATTAATTTATAAATTATGTTAATATTTATATATTTTATTTTCCACATCTTTGTAATTTAGAAA
CATAATGGATCAAGAACTTATTGAAAAATTTATAGAAGTGACGGGGGAAAGTGAGGCAAC
AGCTCGTCAATATTTATCATTAGCTGATGGAAATGTAGAAACTGCAATAAGTTTAATGTTTG
ATGGTAGCCAAGCACCAGAACCATTATGTACACCTGATCCAGAACCAGAAGTGAGACCTC
CTATTTTACCTATGCAGGAAGTATTAGTGCCATCTGGTCCAATATGTTCTCTTCCAAGATTGT
CCACTAATGTATTTGACAGATTCAGAGATTTTGCCGTAGAGACTCAACGGCAAGAAGAAGA
AATGGCCCGAAAAGTAGCTGGAGTTAAACAAATATCTTATTGCAAATCAAAAAGATTGGA
AGATTTATTTCGTCCACCATGTGATATTCTCTTTTTGGGTTCTTTTATGGAAGCCCGTGAACA
TGCCAAATCATTAAATCGTTGGTTACTTGTAAATATTCAAAATTCTCAAGAATTTTCTTGTCA
AATCCTTAATAGAGATGTATGGTCCAATCAACAAATTCAAGAAATTGTAAAGGATCATTTT
GTTTTGTGGCAAGTTTTGTCAAATACAAGTGATGGAAATCGCTACGTACATTTATATGATGT
ATATGAATATCCTTATTTAGCAGTAATAGATCCTAGAACAGGAGAATGTATGAAAACTTAC
AATCATATTACTGTAGATATTTTAATATCTGCTTTAAATGATATGTTAAGTTCTCATCCATCC

CCGGAATGTGTATCAAGTGATTCAGTTCATTCTAAAGAATGGAATACTTGCACTGCCACAA
CAAC

91. >MSTRG.1765.1 Gene=MSTRG.1765
ACAAGTTTACGATTGAAACATTGATCGATATATAATAATCGTCAAGCGATAATAACGCGCT
CTATGATCGGAAGGAAAAATCACGAAAGTCGATCGATACATCGAAAGGTGAGAGGGATAT
GATATGCGTTCGACGATATTGCGAAAAATTTGGAAATATTTTCAGTTCGTGGTGTAAACAA
ATGCGATGTCGTTTTTCGACCGCTGCATCGTATACACATAAGTATATGCGTGTGTTATACAG
TGTATGATACGTTACGTCAAAACAATAACACGTGTACGCGTGGGCACATGATTAATTGAAA
ACTTTATATGTTTAATGCGAATCTAGTCAATTCGATTCGTTGAATCGTGGCGGTTTATCGCAA
TCGACCGAGTAATTTTGCACGCGGTTTAAATTGAAAAAGAAAACTAATAAATGGAATGGAT
CGTGATTAAAATTAGTCGATCGTGGATAAAGCATTTTAAATTAAATTCCAAAATATTAATTG
TAATCGTGCGAAACGCTTCTAAATTCTATGCGTTACTTTTTATTTTTTTTTTTTTCTTCTTCTT
TCGTTGTACGAATTGTTTAGAAAAACAATTTGCTCGATTCGCTTCCGTCGGAAGAAGATGTT
CCATCTGACGGACGAAGAAGCGACGACGAATCATAACCTATACGGTACAGTACAGTGTAG
TGTAGCGACTTATATAGAGATATATGCATCGAACAATAAACGCTCACGATCGGCGATCATC
TTTTTGAAATTTTAGTCGTCTTAAGCTCCTACATCTACGTATATGTAACTGAGTAGACGTATT
CCGTTGCGTGTACACGTGATTGTGCCACAGGATGGAAACGACTCGACGCGACTAAAATGAT
TTTTACCGGAATACCGTATTCGATCGCATCGTATTTTTATCGACGTATATACGGAACGAATG
ATTCAAGATCGATCGACCAGGCGCTTCCATTAGATTGTTTTCCTAATTTTTCTTCTAATTCGA
AGAACTTGTATATTGGAAAAATGAAAATGGGTTGGAAAAATCGATCTTGATTTGTATGATT
CGAAATGGAAAGAGGGATCGAGAGAAATGGCGGGAGGGAGGAGGGAGGGGGAAGAGAG
AAGTGGGCCGCGGATAAAATCTCGATAGTCGATTCGATAGACATCCTCGCTTTCGTACCTC
GCACTCGCTACAGCCATAGCCATAACCGTTCTCGTTGCAATTTCTTTATCGGTTAATGCGTAT
CGGTTAATCGTTGACTATTGACTATACGTCGTTGTGAAATATCATATGCGTTACCGTGATTTT
TATAGTTATCGCGTTATGTTGTTTCTCGTTTAATTGTACATTTCATTGCTGCATTTCGAGCAAT
CGAGAATTACAGTGTTTATCGATACACGATCACGAACAGTCGATATAGATGTATGCTATAA
CCGACATCTTCTTTTTGTCGCTATTCATCAACGCTGTAAAATTACGCATCTAGCATGATTACA
TGCAAAGAAATAAATATATGCACGCGTACGTATACTCGAACGTTACCATACATAACAAATA
CATGTATATATATATATATGTACATACACACACATATATATATATATACATATATGTATAAG
TATATACACACGTGTACGCGTCGCGTGCCTATACTTATAGAGGATACACATACAAAGTATA
AATATTATATATTGACTTATCTAACGATAAGCGAGAAACATTTATCGAGTAATCACATAGG
ATGTGTTGAAACATTTAAAATGATGCACAGGGTATAGATGAGAAAAAATCGTTGATATAAT
AACTGATGTCTATATAATGAATGTTTCTTTTGTTCTATCGGCCTCAAGGACACACAAAGCGC
GTACGATCGACAATTAATCTATGGTAGATGTTTTTCAGAAATTCATTCCGATTCGAAAAAAA
AAAGGACGGTTCTAGTATAAGTGTCGTCGGTGTTAATCGTTCGTCGATAATGGAGAAAAAA
AAAAAAAAAAAAGGAAAAAAAAAAAAAGAAAAAAAAAA

92. >MSTRG.1766.3 Gene=MSTRG.1766
CCGTGCTGTTGGCGAGAAGGTCGTTGTTGTTGTTGTTGTTATTGTTATTGTTATTGCTGACCG
AGCGCGACCGAGTCGTTCGACACGAGCCGTGGGACAAAGTCGGACGACTGAACGAGGAG
GACAGCAGATCGGCGGAACGGTTTTGCTTCTTCGGCCGGCCGAGTTTTCTCTTGCGAGATCT
CCAAAATTGATCCACAGTGATCGTTGAATCGTTCGCGTACACCTTCCTCCCCACTTTCTTCCC

AGATTTCGACCCTCGTTTCCCTCTTCTTCTTCCTCTTCGTCCTACGTTGGCACGTTTCTTTCCA
AACCGCGGCGTTACGGTTCTCAACCGTGGCGAGATCCGTAAGGGGGGCGATGACGATGCG
TTTCGCGGCGATTTGGCCGCGACCTCGGAGCGAGACGAGACCTCGACGTTGCCGCGAGAG
GCGGTCGTATCCGATATCAGGCCCAATTCCATCGCGCACACCAACGACCAAACACTTTTTC
CCGCCCACTGGTCCAACGTATCCTTCGTGCTCGCTGCTTTCATTTCCTCCTGGGATCAACGTC
ATTCTCGTCATCCTCGTCGCCGTCGTCGTCGTCGTCGGTGTCGTCGTGGTCGTTACCGTCGCT
GCCGTTGCCGTCGTCGTCGTCGTCGTCGTCGTCGTCGTCGTCGTCGTCGTCGTCGTGATCGGC
GGGAGGCTATGAAACCGAGCGAGACCGAACATAGTCGATGGAGGTCGGAAACGCACGAA
CCTCACGGCTCGAGCGACCGAACTAGCGACACGATCAAGCAGTGCGGTGCCGACTCTTGCT
GCCGCGGTTTCGACTTCTGTGTTATTCGTGGACGAGTGTTTACGCGACAGAGGACTTCTCGT
CGAAACGAATACGGAATCGTATCCGTCGCCTGTGCAAAACGGAGGGCCACCAAATGGGAC
GCCGATGAATTGCGCCGGTTCTCAAGGAGTGATGGTAAAGCAGGAAGCGAGGGATGACGG
TTACGAGACAAGTCCGGACCTCGAGATGAGGAGCACCGGTGGTGACAGCAGTCAGCAGTA
CCACACGCCGCTGACACCGCACACACCGCACACACCGCACACGCCGCACACACCCCTCAC
GCCGATATCGGCGACAGCGCATCAACCCCAGCAACCTGCCTCCGCCGCTTCCACCCTCGGA
CAGGTGAGTCTTTTTTCTCCATTTTCTCCCTCCGAATCCTCCTTTTCCAAGCGCGCGTATCTTC
TACACGCGTACTCTATCGATATCTCGAGATCGAGATTTAAGGATCGATCGAGAGATCGATG
AAAATTTGGTAAAAATCGAAGGAGGAATTACGAAAATGTGTATTTGGTTCCTTGCGAGCGC
GACGATGCGCGCGCCCTCTCGAAAGATATCTCTTCTCATCCTTCGATTTTCCTTTTCTCGCCT
GTTGATGCGACAAGGTAAGGTATATATATATATACCGTGTCGTATATACCGGGCGAGACCG
AATGTATTTCGAGTCGAAATTGCCGTTCGTTTCTCTCTTAACTTGGAACACGAGTCGTGTAC
GTACGGCGAACTGTTCGCTATCGTAACCGAGATAACCCAAGATCGAGGCTATCGAGCCGA
AAGATAATCGGTCGCGACGACCACCAGACACCGATAAAAGTGTATCTCTTCCTTTTGTGCT
ACGCACTGTGTTTCGCGTATATATACGCGCGTCCGGAGGTTGCGAGCAACAACCGGAAATA
ATGGTGTCGCTATCGGCCGGTTATCTGGTCGCGGGATCTAGATCGAGAGTTCGAGACGATTC
CGCGAGAATCGAGGAGCCAACGCGTAAGAATAACGTAAGGGATTCGGAGGGAGGAAGGA
GGAGGAAGGAGGAGGAAGGA

93. >MSTRG.1793.3 Gene=MSTRG.1793
GTTTCGATACGCTGCTCTTTTATCGCGCGATTTATTCGTAAAAATCGCAAGATGATATTCGGT
CCAACTATACGATGAATTTTCTAATCGATCACTATGCCCGTATATACGTGTACGTGCATAGT
TATACGCGTATCTGCACAGGTATTTATACGCATAATAAAAGTGGCCATTGTAAGTAGATTA
CTAGCTATTCTTCGTCCGTTCGCAATTTAAGGTTTCTTTGATTCGCGCGTTTGCCGATAATTG
GATAATGACGGGTTGGAAAGGAAATATAAAAAGCGAACGTTTGAAGCGGGAACGTTTATT
CTCGGATTACGCGTGTTGGCGCGTGTCGAACAGGTTGATAGAAATGGAAGGGATATGTACG
AGCGAGCGACAAACGAGCGATGAAAAGGGGAAACGTTAATATTCGGGTCAGTCGGCGGG
ACGAGTTGCGATACTCTATCCTGAGTAGAGTCGGTGGCAAGGCTATTATAGTATAGGGTGC
ACGATAGTGATATAATCGTTCGTAATACCCGAGTAATACGCGAGTATAGTATAGAATCGCA
TCGACGAAGGATGCGAAGCCGTCGATCGTAAAACCGTACACGAGCAGATCCTCACGACGT
GGTGTATCGAGTCATCCTTGGCTCGGTTGACGCGGGACCAGAAGAGGAGTATCGATCGGTT
TTTCCTTTTTCCGATTGCTCGAATGGAACACGTATTAGCCAGTGTTCCACGTGAAACACATG
TAGGATGAATGGAGGAAATTCAAATTGGAGATTAGCAGATAAATTACCTCAGAATTGCTGG
TCGCGACGCTGGAAAGACCTGGTGACATGGCACCAGTCGTAGGATCTGGACTGGCCGCAA

CCTGATTACCGAGCGGCAATACCGTGGGACCGTGTGGATACGAGGCCAGATGAGGATGCT
GCGGCTTTGGATGATGCACCGTCGGGCCGGGTGGTGCGCTCTGCACTATCATCGGCGCGGA
TGTCGGCGCACCTTCCACCGAATTTCTACCGAGAACGTTTCCGTCTCCAGCGCTGATCGTTC
CGTTACCGCGAAACGATTCGTGAAGGTATTGCCGCACTTGATTCTTCTGCTTTTGAACGACA
TGGTAGCGCGTCGGATTTTCCAGTAATGTCCGTACCTGTATCGAACAAAAATTATATGGCTT
TCATCCATCTCTTTCTTTATCTTATTTATAACGTGGTCTTTAGATCGATATTTTTGCGCAAAAA
GAAAAACCACACCACAGTTTTGTGTTACTTTTAAAAGTTTATGTATAAACCGTTCTCGTATA
TATATATTTGGTAAGAAAGTAGTATCTAAAAAGAAGAGATAAATACGCATAAAGATAATT
GGATGTATCGTTACATTTGTATGTACATGAAAAATATTTGTGTGAGGACAAAAAAGATGGA
GAACGAGAAACGGCAATAAAAGAAAAAGGATGCGCGATAACGAGATATGAAATGAACGT
ACTTGCAAGACTTGTGGTGGTACGTCTACACCGATAGTCGAGAGTGAGGTAGAAGGTACTG
GCCTGGGAGGAGCAGCCGTTGGTCTCTGTTGCTGTAAATTTTGACGAAATTCAGCTTCTCGC
CTTTCCTGCTCTTGCAGCTGTTCTCGCATCAGCTGAAGTTTCAGTTGCGTACGCGAGGTGGGT
GTTGCAGTCTTAAACGTAGGTGGACTGTCAATATTCGGGGCTCGGCTCTTTAATTCATAATA
CATGATGTCTTGTAGCGGTGTCGACAAAAAGGATAAAGCCTGTTCCCAATCATCATTGTGGT
TATTCTTATCTTCGTTTTCTAGTTTATTGCCACAACACGATTCATCGATACCGGTTATCCCATT
TTGTTTGTTCTGACATCGACGATTGCACAAATCCGCTTCGCGATGTTCCAAATCAGCGGTAA
TAGCTGCCAGATCGAAA

94. >MSTRG.1801.6 Gene=MSTRG.1801
GCTTTGCTTCACACGCATTACGTACCGAATCTTAATCACGATCACGATGAAATTCTCGAGAA
TTTGACGCGATACTGCGGAAAATCAGTTTATAATTATACCGAAGGCGATTATCCCCTTAAAT
GGGATTTTTATAATAGTTTTTATTTCGCTTATACCGTCGTCAGCACAATAGGTACGTACGTAC
TAATAAATTCTCAAAATATCGTATTAATCATATCTAATAATTACGATTTAATTACGATTTATA
TCACGTATGAATTGTAACGAAAAATTTATAATTTTCATTGTTTAATCGTGTATCGAATACAC
ACGATATAATACACGTAGGTTACGGGAATTTGGCTCCAACGAACAGGCTCAGTCGCATCCT
GATGATATTTTATGGTCTGATAGGCATACCTATGAATGGAATTTTGTTGACACAATTGGGAG
AATTTTTTGGCCGTGTATTCGTTAAAGCTCATCAAAAATATAAATCTTATAAGCATGGCCGT
GACAATTATTATCCTAGAAAATTAACAACATTCGAAACGGGAAAAGCTGGATTGGCTGCAC
AAATATTTGCACACTTGTTGCCAGGTTTCGTCATGTTCATCTTTTTCCCAGCATTTCTTTTCTC
TCATTACGAGGGTTGGAGTTACGAAGAAGCTGTTTATTACGCTTTCGTCACATTGACCACCA
TTGGTTTTGGAGATTACGTGGCAGGACAGGACAATAGCAAGGGTAGCGGGTTTTTCTTTATA
TTATACAAGATCTTTCTGATTTGCTGGATTTCCTTTGGATTGGGATACACTGTCATGATCATG
ACATTCATCACGCGAGG

95. >MSTRG.1805.50 Gene=MSTRG.1805
ATATATATAATATAAATTTAATGCATAATTTCAAAAGATTGGTGGAAATGGACAATGTGTA
AATATTCGACAATGTGGAAATTATAATGGTCTAATCTCGTGGTTTATGTTCTAAAAACTGTA
AATACTGTGATGGAATATTTAAAGAACTTGGTGAGAAAAGAAAATTGTTCAATTGCCATTA
ATAAATTTTTAATAATTTGTAATGAAATGACAACACTCGTTTCATTAGAATCCATTGTGCAT
TGTGTTTTCATGTGAAAATGGTAAAGGAAGTTTTGACTCTAGTCTTCTTAAATATGAAAAAA
TTCATCCAATATACCTGTCATTGCCAACGTACACAGGCAGCCATAAGATGTTATGCAGATG
CACGACCATTAACTTTAAAGCTATTCTATTTTATCATCTTTAAACGTATGAAACGAAGCAAG

AAATTTGGTTACCAGATATAGGAACTTTTGATCCTTCGAAGAATTTAAAGTTGTGAATATTA
ATTTAGATAAAAATGATTTGAGCATATCAAAAGAACATTAACATTAACGTATTACGTAACA
GGATGAACAAGATAAAATAGTAAGCATTGATTTCGATAATCTAATTACGGATGATGGAGAT
ACACATTTCGAGATGGTACTTTAACAATTGAAAGTTTAGATGGAAATAAATTATATTTAGAT
TCAACGACTCAATTTAGAATGTGTGGAAATGGACAATGTGTAAATATTCGACAATGTGGAA
ATTATAATGGTCTAATCTCGTGGTTTATGTTCTAAAAACTGTAAATACTGTGATGGAATATTT
AAAGAACTTGGTGAGAAAAGAAAATTGTTCAATTGCCATTAATAAATTTTTAATAATTTGTA
ATGAAATGACAACACTCGTTTCATTAGAATCCATTGTGCATTGTGTTTTCATGTGAAAATGG
TAAAGGAAGTTTTGACTCTAGTCTTCTTAAATATGAAAAAATTCATCCAATATACCTGTCAT
TGCCAACGTACACAGGCAGCCATAAGATGTTATGCAGATGCACGACCATTAACTTTAAAGC
TATTCTATTTTATCATCTTTAAACGTATGAAACGAAGCAAGAAATTTGGTTACCAGATATAG
GAACTTTTGATCCTTCGAAGAATTTAAAGTTGTGAATATTAATTTAGATAAAAATGATTTGA
GCATATCAAAAGAACATTAACATTAACGTATTACGTAACAGGATGAACAAGATAAAATAG
TAAGCATTGATTTCGATAATCTAATTACGGATGATGGAGATACACATTTCGAGATGGTACTT
TAACAATTGAAAGTTTAGATGGAAATAAATTATATTTAGATTCAACGACTCAATTTAGAAT
GTGTGGAAATGGACAATGTGTAAATATTCGACAATGTGGAAATTATAATGGTCTAATCTCG
TGGTTTATGTTCTAAAAACTGTAAATACTGTGATGGAATATTTAAAGAACTTGAATCCATTG
TGCATTGTGTTTTCATGTGAAAATGGTAAAGGAAGTTTTGACTCTAGTCTTCTTAAATATGA
AAAAATTCATCCAATATACCTGTCATTGCCAACGTACACAGGCAGCCATAAGATGTTATGC
AGATGCACGACCATTAACTTTAAAGCTATTCTATTTTATCATCTTTAAACGTATGAAACGAA
GCAGAAAATTTGGTTACCAGATATAGGAACTTTTGATCCTTCGAAGAATTTAAAGTTGTGA
ATATTAATTTAGATAAAAATGATTTGAGCATATCAAAAGAACATTATCATTAACGTATTAC
GTAACAGGATGAACAAGATAAAGTAGTAAGCATTGATTTCGATAATCTAATTACGGATGAT
GGAGATACACATTTCGAGATGGTACTTTAACAATTGAAAGTTTAGATGGAAATAAATTATA
TTTAGATTCAACGACTCAATTTAGAATGTGTGGAAATGGACAATGTGTAAATATTCGACAA
TGTGGAAATTATAATGGTCTAATCTCGTGGTTTATGTTCTAAAAACTGTAAATACTGTGATG
GAATATTTAAAGAACTTGAATCCATTGTGCATTGTGTTTTCATGTGAAAATGGTAAAGGAAG
TTTTGACTCTAGTCTTCTTAAATATGAAAAAATTCATCCAATATACCTGTCATTGCCAACGT
ACACAGGCAGCCATAAGATGTTATGCAGATGCACGACCATTAACTTTAAAGCTATTCTATTT
TATCATCTTTAAACGTATGAAACGAAGCAGAAAATTTGGTTACCAGATATAGGAACTTTTG
ATCCTTCGAAGAATTTAAAGTTGTGAATATTAATTTAGATAAAAATGATTTGAGCATATCAA
AAGAACATTAACATTAACGTATTACGTAACAGGATGAACAAGATAAAATAGTAAGCATTG
ATTTCTATAATCTAATTACGGATGATGGAGATACACATTTCGAGATGGTACTTTAACAATTG
AAAGTTTAGATGGAAATAAATTATATTTAGATTCAACGACTCAATTTAGAATGTGTATGTAT
ACAATTTTACCACATAGTATCGTTGAAAATTTTGAATCGGATGTGATCGTTCCTTATTAGAG
GAGAATATGATATCTCTCTAATATATTTTTATATATTTAAAATATATTCAATGATAGGCAAT
GATAGAGATTGGCATGGTGAAATTAGATAGAAGTTTGAAAATGATAAGCGATATGTAATTG
AACAAACGAATCATAGAAAAAAATTGAAGATAAATTTATGATTAAATTTATGAAAATAAA
TATAAATCTGATGATATCTTCGTTAATAGTATT

96. >MSTRG.1806.30 Gene=MSTRG.1806
TGTTATAGAAGAGCTAAAATAGGTTATTTAATTTAAATATCGTTTTCTGCCGTCACGTTTAAT
CACGTTATTTTCTAGTAGATATTCTGTGAATTTTTATTTTGTAAATCATATTCGCATATATATA

TATATTTAAAGTATAATTTATAAAAATATAATATATATAATATAAATTTAATGCATAATTTC
AAAAGATTGGTGGAAATGGACAATGTGTAAATATTCGACAATGTGGAAATTATAATGGTCT
AATCTCGTGGTTTATGTTCTAAAAACTGTAAATACTGTGATGGAATATTTAAAGAACTTGAA
TCCATTGTGCATTGTGTTTTCATGTGAAAATGGTAAAGGAAGTTTTGACTCTAGTCTTCTTAA
ATATGAAAAAATTCATCCAATATACCTGTCATTGCCAACGTACACAGGCAGCCATAAGATG
TTATGCAGATGCACGACCATTAACTTTAAAGCTATTCTATTTTATCATCTTTAAACGTATGAA
ACGAAGCAAGAAATTTGGTTACCAGATATAGGAACTTTTGATCCTTCGAAGAATTTAAAGT
TGTGAATATTAATTTAGATAAAAATGATTTGAGCATATCAAAAGAACATTAACATTAACGT
ATTACGTAACAGGATGAACAAGATAAAATAGTAAGCATTGATTTCGATAATCTAATTACGG
ATGATGGAGATACACATTTCGAGATGGTACTTTAACAATTGAAAGTTTAGATGGAAATAAA
TTATATTTAGATTCAACGACTCAAT

97. >MSTRG.1842.2 Gene=MSTRG.1842
ATATTCTATTATTCATTTAAATATATAAAATATTGAAATAATTATACAAAAGATATTCAAAT
CAGAATATTTTGATTTATTTATTATTATTAATAATTATTATTATTATCATTATCATTTTTATCTT
TCTTTATATTTTATCCCCTCTCTAAACATTTCTGATTCATTTTATAGATGACTTCATAGTTAAT
TTATTTTAGCCTTTTGAAAAATTTAAAATGAATATTGAAAGTTCTAATAGTATTCATTCTGAT
ATAAATGCAGAAACTTATGATATCCCAATGAATATACTTATTCGACCTATTCCTCCAATTAT
TAATGAAAAAAAGGTTCAAAGTTTAATAAGTACATTAAGCAATCCTGAAACAGAAACATTT
GTACCACCAATCGATGTTTTATGGATTAAAGGAAGCGAAGGCGGTGATTATTATTATTCTTT
TGGCGGATGTCACAGATACACAGCTCATCAAAGACTTGGAAAATCATTTATAAAAGCTAAA
TTAATACAATCTACAATAACAGATTTAAAAACTTATCTAGGAGGTTCTATGCCAA
98. >MSTRG.1877.2 Gene=MSTRG.1877
GTTCTTGCCCGTTTTCGCTCGGGTGACGATCGATCGGCGATTCAACGCTCCGTGACCTTGTTT
CCGCGCATCCCTTTCCGCGACACGATTTCCGGAGAGATTAAACGATCGCCTCTCGGAAGAG
AGAAAACCACCGGCTCTGTGCGGTGGTTAAACAGAGACGGACGGTTCGTGATTCCTTCTCT
TTTTTTTTTTTTCTTTCTGTGAAGTGCAGTTGTGTTTCGCGCCAATCCAATTCCAATTGTTGAA
AAGTGTCACTCGGATTCTGGCTTCTTCTTTCTTCTTTTTTCACGAGGATGAACGATTCAACGA
TCTCCGTTGGAATTTTATATCGCTTCTTAGACGAACGGTGAACCAGATTCTTCGCGGACATC
GCTTATCTCTTGGAGGGTTTCGCTATCTGTCAAAACGTGCCCCTCTCCATCCACAAGGACGA
TCAGCCTCAGTTATCCCGTCCACGAGTCGTGAACCACGATACGAGAGAGAATCGACAGTTT
CAGCGAGGATGTCCCTGCACGAGGCGCGTTTACAAGCCCTCCTCTCTTAACATCTCGTTCCT
GCTGCCATCGACACGATCCTGCGAGGTGTTGGCTCGAGGCAGGGATCGGCTTACGGGGGTG
GAGGCGCTATAAAAATAAAGGAAATGGGGGTGCCGGATGTAGAGCAGGCCAAGAGGCGA
CTCCAGGACATTCTGAGGAAAGCGCAGAAGCTCGAGATCCCCCCGCAAGAAGTGATCTCG
ACGCGGACCGCCCAGAAGCTTCTTGCGAGGACGAAGAACGTCCTGGCATGGACGATCTGG
ATCACTGTTTTTATCCTCCTGCTAAGCGGAGTGGTCTACGCCCGTTGGCCAACGAGGCAAGA
AGTCGAGACCATTAGAACTGTCCTCAGGCGAACGTGCTCGGGAGCAGCGTCTGGGGACTTT
TCGGAGCGCGTGGCCGCCCTTTTACACTCCTCCTCCACCCAGGACGACTGCTAGTCTTCGTC
GAGGTTCCTCCAACTTGTTCCACCTTCCTCCAACGAGCGAACAGACCGAAAATGGAATGC
CAAACAGAGGATATCGATCCTGTGATCCACGCTCGTTCCCCAAAGCCTTATACTTTGGCTTC
GAAATTAAGCGTTAATTGTGTTAATCGCTTGGAAACGATTTTCGTCGATCTGAGAAAGAATT
GTATAATATAATCGAGTGGGTG

99. >MSTRG.1883.1 Gene=MSTRG.1883

GGATCAACTCTTGTTCTCTCGGATCATTCTCTTCGGCGGAGCGCTCAGTGCGCTGCAAGCGC
TTCGAGGTTGCGTGTCGGCTTTGTCGCTTTTCGCGGGAAGGGAAGAATTTAAATCGTCGATC
GGACGAGTGATCAGTGCGAAGAGATTATTTCCTTACGCGAGGATTACGTGAAAAAAAGGA
GAATTTCGCGAAAAAGTTTATTTGGATTTGTTCGGCTAAAAAAAGGAAACGAACGCTCGGG
TGAAATTCTGAAAACGGAGCCAAGTGATATCGACGGGAGTGTCGTCGACGTAATTTTAGAA
CGTTTCAAATAAGGTGCCAATAAGATAAAAAAGAGAAGATCACGGCGGATTTCGAAAAGT
GTAAACGAAGAATTGGAGGAGAAACAAGAGGGATAAAAGAAGATACGTGAAAGATACGT
TTTGTTGATGAAAAAATTATCGTCGTCGTTCCTCTGAATGGTCGAAAATTTATTTCGCATAAT
TTAACGATTAATAATTCCTGTCTCCTTTCCTTGTCAGTCTTCTTCATTCTCTTTTGGGATATGTT
TGAATAGACAAAAAAAGAACTTGTGTCAGGATCAAGTATAAAAGGCTCGGGCCTCTTAAC
AAACTGTTGACCCGAAAAAGCGTTTTTCCTCGTTAACTTAGCCCATTCTTCTTCGAATGATAT
ATATATTTAAATTCCTCTTCATCCAGATCAGATTGATGCGTCGACATTTTTTAATATCTATCC
CCCCTCCCCCCTTCCACGAATCGTTTATTCAAAATTGGTTTTCCCATTTATCCACTCAAGTCT
CATCCTCGGTGAATATTAATCCGGTTAATTCTAACGAAAAAATATCTGTGCAAGGAAAAGT
TTAATATCACGTTTCCGACATTTAGAGGATCGCGTCAAATTTTATCAATTTCTTGGAAAAAG
GAAAAGGATGCGTTAATTAAAATTTGATAAACTGTATCGGCAGCAAAGAGGCAAGAAAGA
TAATTAATGGAAGGATTGATTTTCTTGCTAGGCTTCAAGTAACAAAGTTTCTCCTCGAAGGG
GAGAAACAAATAAATAAATTGCGAGGAATTATGCGAAATTAGCTTCATCCGAATGAAATT
ATGCATTTGAATCGGAGATAAGATGAAGAAGAACTTTCAAATTATTCTATTATTTCCTTCAT
CTTCGAATGAAATCACGGGAAAGACTACTTCCTCGAGATCGTCAAAATTATTAATTTCGAT
AAATTATTGAAGAATAATTTTAATTCAAATTTAAAAATAAATGATAAATAAAGAAGAGATA
CTTTTGAATAAGAGAATATTAATTACACGTTTAATTGAATCTACTGATTATCGTATATCGAT
AAGCAAAAAAAGAAAAAAAAAAAGAAACACGAAACAATAGTTATCGAATCTCTCCGTTAA
AAGAAAGAGAAATGGTATTGGTCGAAGCAGAGGGACGATAAAAAAGATACGATAAAGGC
ACGAGCTTTACAGCGTAAAGAAGAAATTCGATTATCCCGAAAGGGACCGTTTACCTGATTT
CGAATCGAAAGGGGAGACGGTGGCGCCCTCGCGGCGATTCGATCGCGCTTGTCAACGATC
GTCGAACTGAGGGCTGTTTCGATATCCCCTGCGAAGATAAGGGACAAAGGGAAACGAGGA
CAAGAGCTATTCGTGTTCCAACTTCCTGCTATTTCTCTCGAGAGACGGAGGAGGAGGAGGA
GAAGAATGCGGGAAAACGGGGAAAGAATCGTAATCGACTTTTCTCGTAGAAAACAGTTTC
TTCATATCTCTCGTATAGAGTCGTGTAAATGGAACGTCTCTACGAGGATAGATTAAAGTTGC
AAAATTCTATTTATCCGCAAGAACGAGAATCGTGTAATTAAAAAAAACCAAGGATTTCTAT
CTGTATAAATGAACAAGTGATGAAAGTGAAAAAAAAATACGAGTGTCGATAAAGAAGAT
AAAAGATTCGAAACTTTTGAGGTTATGAAGAGAATAGCGCGAGATATGAAAGATGTTTGA
AAGACGCGTCACATTTAAACGTCGGTTTTAACTCGAGACGAGTGTAAAGGTAGATCTCCTT
TAAGCTGAGAGAAGATATATGAGACTGCATAGGGAAATTATATAGAACAGTGCCGGATCT
TTATGTGCGATAATACCGACGGAGGAGATTTAACGCGCAATGTCTCGAATTGTTTCTTTGCG
ATCGAAAAAGAAACTGAACTCTGTCATATTGTTTGAGATTTTTTTTTTTCTTTTCTCCTTGTAT
TAAGAACTTTAACTGAAAGATAAATCTTCGAAAAATTAAATATGCAATATTCTCGTGATATT
TGACATCGGATATGAACACGCTATACCAAAAATCGATAAAAGGAGGGGCACGCTGCAGCA
TCTGACGAAAATAAATCTTTGACAGATTTACGATTCTGATTATTTTTCACGCACATTCCTATT
GATAAGTGATTCGTTCTATATTCTAGTGATGATGATTGTCACAGAGAGGCGAGTAATTATGT

CATGATTTCGGATACGAATATTTGGAAATGCGAGTTAACACGTTCAATCAGATTTATGAGA
ATAAAAGCCGAAAGGAAATCGAATTATCGATTGCTGCAAAATTACGATACGTTACAGATG
CAAATACAAGAAGTTTACAAAACTTTCATCAAGACCAAGCAAATTGAATTAAATTCAATAA
TATTTAAGAAAATAGAAGAACAACAAGAAATAAGATAGC

100. >MSTRG.1898.3 Gene=MSTRG.1898
AAGCACGCGAAACGCGAGTTGTTCAGTCCTCGGCAAAAGGACACGAGGTTTCGTGTTATTC
CTAGATATATCGACGTTATTCCTAGACAAGTGTTCTTGAAATCCTGATAATTAAACTAAAAG
ATAAATCAAAATAAACTATAACTTTTGTTTATCAAATAATTATTTCGAAACTTTAACACGTG
TTTAAAAACGATCGAAACGTTCGCGAAATAAATTAAACTTGTAATCGTATATACGTAATAA
TGAATCTCTTTAATAATTTTAGCTCGTGAAATATAGATTCAAAGACGAAAGGTGATACGATC
AATCGTCTGTGGATAAGACAAACTACGCGATACGGAAAATTCGATCGTGAAGAAGCATCT
CGAGAAGAACTCGAACGTGACTCGACTCGAAAGAACAAGATCGACAAACTTCAGGATCGA
CAAACTTTCTTAGATACACGGACATGGCACGTCGTCAAGCCAGTGACAAGAAATCAATATA
TCCTTTTCGATCTTCCCGCGATATCGCGTTCACTTGATTAATCGAAGACATGTGCACATTGTC
GTGAATTTGAACGATCTAAAATCCAAAATACGTATAAGATCGATTTTAAGTGCAAATCAGT
GAAACCGTTCTCTGCCATTGTTGTAATATATTAGAGAAATATAATATTAAAAGAAATTTATC
CATCATCGATAACGTTAATATTTGAATGAATAAATCGAAGAAATTTGGTTCATAAAAAACT
TTTAAGACATTAATCGTGATGTGTCTACAAACTATCCATTGATTTATTCGAAAAACTTTCGA
AATATTCAACGGTCAGTTCCTATTAATAAATCGATTTTTCGCATTCGAGTGAAGTTGCAGAG
AGTTCGATCGATGGAACGACCAGAGGATTTAGTGTCGAAAGAAATGTGCAGAATGTGGTC
AATTTACAACGTATAAATTGTCTATCAGTTAAAACACGAAGAGCAAAATGTCGGTTGAAGT
GAAGGGGGGTCGACCAACAATGCCAACAATTCCACAATCCAAGAGACCAACCATTTTCGT
TTATCCCACAGTAACTCCAGAGAGCATTATCATCCCGATAGTATCATGCATACTCGGATTTC
CATTACTGGCCCTTATGGTCATCTGTTGCTTAAGAAGAAGAGCAAAGTTAGCGAGAGAACG
TGCACGAAGAAGAAATTGTGATCTAGATCATGGAGCCCTTAGTCTCGTTCGTTTTAGCCCTG
TTCACCGTTTAGATTGAGGTCACAAAACATTTGTGTTCAACCCCAAACCGCTACTTCCGCTC
GCTTTTTCACCTAGAAACGCTGCTGTACTTACACATGTATATTCAAATATACACTTGCCT

101. >MSTRG.1907.3 Gene=MSTRG.1907
TTCCGGTTCTTTACGTTGGTCGTGAAGATTATACATGTTATTCCACTCGACAAAATTCCTCTG
CTCTTCAAAACTATTGCTAGCTTTCGGACTCGAACTTGACATGCTGCTTTCGATGCTACTGTC
CTTTTTATCAGACGATTCAGAGTGAGTACTACTACTTTGCTTTTCTGGTGAAACGGTATCGA
ACCTTGTCTTTTCCTGAGAATTATTCTCCTCGAAACTCTTCTCTTGCATTTTATCATTGTTGTTT
ACGTAGTGTCTATATGTTTTTACATGCTTGCGCAAAGATGAGGGATCAGTGTACCTCTTCGG
GCAACCGGGAACCTTACAACAGTAAGGCTTGTCCACGGCGTGAGTTCTGGTATGCTTAAAT
CTGTCCGAGGAATTCGAGTAGGCCTTATTACAACCTTCGACAGGACAGACATAAGGGCGTT
CACCTGTATGGGAACGAGCGTGAATTTTCAGATTCTCCGCTCTGCTGAAACTTTTATCACAT
TGAAAGCAATGATGCGGCTTCTCATTCGTGTGCGTTCGGACATGGACCAGCATCTTATACCT
AGAAAAGGAAAAATGCGATGAAGTAAGGATTGAAATTCAGTCGATTTGAAATCATGCAAA
ATGAAAAACTCTTTACCTAGCATTAAACCCACGTTCACCTCTGGCACATCCTTCCCAGCCAC
AATAGAAGAGTCCACGTGGTCCTGGCGCAGCATGCAATCGTGCAACATGTCCTGCCAGCTG
TTCCAAGGACGTGAACCAACGACCACAATTCTGCCATCTGCAAATTGGAACGGAATGTGTT

AGAAGAAACAAGTAGCATGCCATCACGTGTACCAGAGTCGACGTATAGAAACGTAATCGG
AGTGGAATGGCATTCAGTTGAAATATCGACGCATGATTTACGTCAAGAATGTCCGCCAACC
GACCAATTGATACCAATTCCGTCCGTTGTGCAATTGGCGTCGCTTGACATCACTTTATTTAG
ACGCATGCGCTATTTATCACGCTAAACGTCGTATTCTGTGAACGGCCATGTTGTGTAAAAGG
TTGCGGATCAAGGTAATGATGTTCACCATCTAATTCCCCCGGAACTGTATACAATTATTCTT
TTTTTATTTCATTGCTGTATATTCATTGCTGTTATATTCATTGCTGTTTAATAATCTCAGCAAC
TATATGAAAGGAAGAATTCGTCACTTATCAAATTTCAGAAAAAAATGGAGGATTTGTCACAT
GACGAATATCGTGATTCAACGATGACAAAAATAACGTGTGTGATTACGATAATGAAACAG
ACATTCCCGAATTCACGAAGTTACCACATGCAAAATGAATGTAATGAATTATTGGACCATG
ATAAACGATCACATCTAAACCCAAAATCTCGTCGAAATATATCGTGAACGAAATTTACAAA
TACGATATTGCTGAGTAATCAGAAATCTTTATTTCGGGGTTATCGAAATCTGACATTTTCTTT
GAAACAGTAAACACTTAGATTTGTCAAAGTCTCGATTCTAGACCTTTAAAAATGACCTA

102. >MSTRG.1924.1 Gene=MSTRG.1924

AGGTTGGTCAATTTCGAGCGACGAAGCTGAACACTTGAAGCCGGCTGGGCGGATCTTGTTC
GAAGCTTCAGCGACATCCCTGCTCACTCTGTCTCGCTTTCTCACACGGGCGTCACCTCCTTC
GATCCTTTATTCGATGCTCAGAAACGGCAATTATTCGTGCACGCCATCCTCGTCGTCGAGAT
TGTCTTCGACTTATTCCTGTTTTTATTGATTTTAGCCGACTTCGCACATTCTTACACTTTTCAT
GATTACAAATTCACTCCTCGAAAAACCACGAAATTACGAGTCTCAATACGAGAACCCGAT
AATTTTACAGACAAAAAATTTTCCTCACATCCGACGGAACGGAGGTCGTCT

103. >MSTRG.1925.1 Gene=MSTRG.1925

CGTGTTGAATAATATGCGTACACCAACGCTTCTTTTTTTCTACCTGTGTTCACGGTTCCTTTTT
TATCTACCGTAAAACCGTGCAGTTACTATAAAGTGGACTGAATCGTTCGTACCAATCGTCGT
TCGAGCGATGTTTAATTAAATAGCAAGGTGTGAAAGGAAGCGGGAGTCTCGAATAGACCC
GTTAGGCATGCTAACGTACAAAAGAAAAATTTTTAAAGGAATAAGTTTCATATATATATAT
ATATATATATATTGCCTTTTTCGTCATGTTTTGCTATCGTTTAAGGTACAAGCGTTTAATATAT
TAATATATGTATATGTATGTGCGTATGTATATATATATAATATGCATATATGTATATATATAT
ATACATATGCATATGCATATTACATAATCATTATATCGTTCATTTAGTCATTCAGTTGGTTCT
ATTTGGAACCGAGTGGATTTGTGTCAAATATCTTCGTCAGTGTTTCGATAATATATTTATTTT
TATTCTGGAATTTAACTCCACTTTATAGTATTTCTTAATATAATCTCGCGATTCTTTTATATTT
TTAGTCTTTATGCTGGCATCTTATTTTTTTTTTTTTTTGTTCTTGTAAAATTATGATTGTACTGG
GAAGTACATGATCGATGTCTGTGCGTGTGTGTATGCGTGAATGGACGCGCGTATGAGTGTAT
GTGTGTGTGTACATATATATATATTTATATTTATATATATTATATATATATATACACACATATAT
ATATATGAAAGAAAGAAAGAGAAAGAGGAGCGTCGTTTTATTGGATGCAGAATTCGAATT
CGCCGTTTCCTCCCACAAAAGATATTCCATTTTATTAAATATTTTGAATCGGTGAAACGAGA
TTCATCAAGTGCCACATTATTTAATTTTTTTCGAGGCTGAATATTTTTTCGTAATATTCCTATA
ATTATTATGATATTCTCGTCCCTTGTACCTTTTTCCGATATAATAATCTCGTCGATCCAGAGA
TAAACGAAATAAAAATAAAAAAGGAAGAAAAGAATAAGAAGAAGAAAAAAAAAAAGG
AATGAAAAAACTCACCCACTGATTTTTCAGTTTTTCTGGTACAATCGGCGACAGCATTAGTG
ATTCGTTGTATATATAGAGCGGTAGTAATTGTTATGTAGGTAAATGGCTGGTGTTGTATACA
CATTCGTTCTTTTCCCTAATTATTCGAATTATCTTGTAACCTGTAACCATAACTCGGAGCGTT
GAGCGGACGGCGCTCAATAAAAATTTGTCGTACGCTGTCGTTGTATGCGAATGAAAATGGC
AAAAGCGAGATGACGATAACGTTCTGTAAATATATAGGAGTGCCTGAAACCACGTTGAGA

GTGAATGAGATACAGAGAGAGAAAGAGGGAGAAAAAGCGAGAGAAAGAGAAATGGAGA
GTGGCAAATACTTTTCCAGCGGTGCAAGAACAAATGAATGAAGCCGTTGAAAAAGCAATC
ACAGAATAAGAGGGGAAAAAAAAAAAAGGAGAAAAAAAGAACAGAATAAGATTATTCTT
TGCTCCTTGGCGCGTTGCGATCAAACCTGATCGATTTTGCAAATTAGATCAAAGGGAAAAA
AAGAAGATTAAACGATGTGTGAAGCTAGTGATCGTATGCACAAAATCAGCTGTTGCTTTTT
AAGGAAATCTAATTTTTTTATTTACTTATTAATTACAATGAGAGCTTTTTTAGGAGTTATTTTT
AACAATTTGACGATTGGACCTCAAATGGACTCTCAAATGTATGCGACAAATCATCATATTT
GGATTAACGCGAAAATGCAAATCCTGTATCCTTGAACAAGTATTCCTGCTTTTTGTTCTTGA
CTAATGTAGATATCGTAGCAATCATCATTCTCGTGGGATTGCTTACTTTATCGTCGCTATATA
GCGAGAATTACGGGCTCACGTGTAACAAGTAAATTGTTCGGGCAGCCATTTTGAAAATTGA
TGTTTGCCAACACAGTCGTTCGTCGTATAGAAAATTAATTCTTGTCTAGTTAACCGTGTGGA
TCGATTAAATGTTTGAATAGCAATATTTTACGAGTATTACAAAATCAAATGCGCTAATGTTT
TCTTCTACCTAATAATATCTAACAGTAAATCCTCGTCAATAATTCAAAAACGTCAATGCGTT
ATCAGCTTCCACACGATGTTATTCAACAAATAAAAGGATTTAACGTTGAACAGTATTAAGT
ACAAGAAATTAAATATGAAGAAATGTCTAGGAATCGTTACCATCTAATCAATAACAAATG
AACGAGGGAGCCCGTAATTTATCCTTTCTTTCATCATCCGCTCTTCCCTTCGATTGTTCATTTC
CTATTAATTTTTTCATTACGTTATTTCAGTATTTTTCCACACAGCGCTTAGTTCCTGAGAGCCT
TCGCGCCAAGGTAGTCATTTCTCATTTTCATAAGGGAGTGGAGCACGGACGAGGCGTCGAT
CGATTTACCGCATTTTTGCACTATTTTTTCAATCTGTAAAAAGATATTTATTTGAAAATAAAT
AACTCGAAAAAAAAAAACGTTCGGCGCCTCGCGATCTTCTTATGTCTGCGTATTGCGATCGTA
TCCTTTCTTTATTCTCTGTCAACATTTCCTTAATTTATTCTTCTGCTCAGGTTCAGTCATGCGA
AGCGAAATGACTAGAAAAAAAAAAAAATGACATTCACCTCGAAACAGGATTCCAGTAAAG
CTGCTTGTTTCGTTTCACGTAATATCCGAATGGAAAAATATAGGTCGAAAAGTAACCTTGA
AATGGACTGACATACCGTGTCATTGCAATCGTCGAGTTTCGAATTATTATGCATTAACCGGT
CCATGGAAATTGTTGACACTTATCTCAGACGGTTAATTGATTGAAAAATTTGCATAGATCCC
TTGCAGTATTAGAAGTTTCACATATCTTTTCAAGACGAGTACAAAAGCCCGAATACAAATTT
TATAAAGGAGAAAATTCTGCAAATAATTATCTGATACTTGTTTAATAAAATGATTATTTATA
ATAAGATCGAATGTAATTGCATCCAGTTGAAAAATTTATATCGATGAAATATATGTCGATG
AAAAATATTTCGTCACATCGGCAAATGCATAAAACAAGTGAAACTCGTTGATGAAGAAAG
TATTGTCGGTAATCTCAAGGCAACCCGGTCAAAGAATATTGTTTTTCGGTTGGTCAAGATAT
ACCAAGATTTGAAATGCACTTTTATATATATATATACATATACATATATATATATATATATA
TATATATATATATATAATGATTTCATCGGTAACTTACATCATTATGTCTGTATAAGAAAAAG
AAAGATTCGAAGGACTAATTTCTATGAAACACGTGTGTCTGATGTTTCATTAGACTTATTTT
GTAACGTGATAGTCAAGCCGGTCTTCATGCTTCCAAAAATGTCAGAACGTGAAATGAACGT
TATTATACAAGTCATTTTCTGAGAAGTTAAAATTTCGAAAGTTCAAGGTAAAGCGTTTCTCC
CGTATTAATTTTTTTTTTAATAATTCCAACAATCGATACTTAGAAATTTATTAGAAATTATGT
AGAAATTGCATTCCTTTCTCTATTTCTTTTCTTCCTTCCTTTGTTTCTTTAATCCTCTAGATTTT
CAAAATAATGTCTTATTTGTATGGATGTTTTTTGAGGCTTCTTGTGCACGTCTTTGATTCCATT
ATCCGGCAGATATTAACGTTGTGATATTTTATAATATTGTAACGGAACTCGTTATCACTTCTG
TATTATATTACATGAATGATATTATTTTATAATTATTTCGGAACTGGCTTCTGGAACACTGCG
GGAAAATGCGGCTCTCCGGCGACCTGAATGGTCCCTTTCTTTTTTTTTTTTTTTTTTTTTTGT
TTGTTTTTTATTTATAATTGTTGACGTTTTGTCTGATTCTTATTTTGTGAAATGGATGCGAAAC
TATCATGTGCTGATAATACTTTGAAAATCAAAAAAAAAAAAAAAAAAAAAAAAAA

104. >MSTRG.1925.2 Gene=MSTRG.1925

ATATGCGTACACCAACGCTTCTTTTTTTCTACCTGTGTTCACGGTTCCTTTTTTATCTACCGTA
AAACCGTGCAGTTACTATAAAGTGGACTGAATCGTTCGTACCAATCGTCGTTCGAGCGATG
TTTAATTAAATAGCAAGGTGTGAAAGGAAGCGGGAGTCTCGAATAGACCCGTTAGGCATG
CTAACGTACAAAAGAAAAATTTTTAAAGGAATAAGTTTCATATATATATATATATATATAT
ATATTGCCTTTTTCGTCATGTTTTGCTATCGTTTAAGGTACAAGCGTTTAATATATTAATATAT
GTATATGTATGTGCGTATGTATATATATATAATATGCATATATGTATATATATATATACATAT
GCATATGCATATTACATAATCATTATATCGTTCATTTAGTCATTCAGTTGGTTCTATTTGGAA
CCGAGTGGATTTGTGTCAAATATCTTCGTCAGTGTTTCGATAATATATTTATTTTTATTCTGG
AATTTAACTCCACTTTATAGTATTTCTTAATATAATCTCGCGATTCTTTTATATTTTTAGTCTTT
ATGCTGGCATCTTATTTTTTTTTTTTTTTGTTCTTGTAAAATTATGATTGTACTGGGAAGTACA
TGATCGATGTCTGTGCGTGTGTGTATGCGTGAATGGACGCGCGTATGAGTGTATGTGTGTGT
GTACATATATATATTTATATTTATATATATTATATATATATATACACACATATATATATATGA
AAGAAAGAAAGAGAAAGAGGAGCGTCGTTTTATTGGATGCAGAATTCGAATTCGCCGTTTC
CTCCCACAAAAGATATTCCATTTTATTAAATATTTTGAATCGGTGAAACGAGATTCATCAAG
TGCCACATTATTTAATTTTTTTCGAGGCTGAATATTTTTTCGTAATATTCCTATAATTATTATG
ATATTCTCGTCCCTTGTACCTTTTTCCGATATAATAATCTCGTCGATCCAGAGATAAACGAA
ATAAAAATAAAAAAGGAAGAAAAGAATAAGAAGAAAAAAAAAAAAGGAATGAAAA
AACTCACCCACTGATTTTTCAGTTTTTCTGGTACAATCGGCGACAGCATTAGTGATTCGTTGT
ATATATAGAGCGGTAGTAATTGTTATGTAGGTAAATGGCTGGTGTTGTATACACATTCGTTC
TTTTCCCTAATTATTCGAATTATCTTGTAACCTGTAACCATAACTCGGAGCGTTGAGCGGAC
GGCGCTCAATAAAAATTTGTCGTACGCTGTCGTTGTATGCGAATGAAAATGGCAAAAGCGA
GATGACGATAACGTTCTGTAAATATATAGGAGTGCCTGAAACCACGTTGAGAGTGAATGAG
ATACAGAGAGAGAAAGAGGGAGAAAAAGCGAGAGAAAGAGAAATGGAGAGTGGCAAAT
ACTTTTCCAGCGGTGCAAGAACAAATGAATGAAGCCGTTGAAAAAGCAATCACAGAATAA
GAGGGGGAAAAAAAAAAAAGGAGAAAAAAAGAACAGAATAAGATTATTCTTTGCTCCTTG
GCGCGTTGCGATCAAACCTGATCGATTTTGCAAATTAGATCAAAGGGAAAAAAAGAAGAT
TAAACGATGTGTGAAGCTAGTGATCGTATGCACAAAATCAGCTGTTGCTTTTTAAGGAAAT
CTAATTTTTTTATTTACTTATTAATTACAATGAGAGCTTTTTTAGGAGTTATTTTTAACAATTT
GACGATTGGACCTCAAATGGACTCTCAAATGTATGCGACAAATCATCATATTTGGATTAAC
GCGAAAATGCAAATCCTGTATCCTTGAACAAGTATTCCTGCTTTTTGTTCTTGACTAATGTA
GATATCGTAGCAATCATCATTCTCGTGGGATTGCTTACTTTATCGTCGCTATATAGCGAGAA
TTACGGGCTCACGTGTAACAAGTAAATTGTTCGGGCAGCCATTTTGAAAATTGATGTTTGCC
AACACAGTCGTTCGTCGTATAGAAAATTAATTCTTGTCTAGTTAACCGTGTGGATCGATTAA
ATGTTTGAATAGCAATATTTTACGAGTATTACAAAATCAAATGCGCTAATGTTTTCTTCTAC
CTAATAATATCTAACAGTAAATCCTCGTCAATAATTCAAAAACGTCAATGCGTTATCAGCTT
CCACACGATGTTATTCAACAAATAAAAGGATTTAACGTTGAACAGTATTAAGTACAAGAA
ATTAAATATGAAGAAATGTCTAGGAATCGTTACCATCTAATCAATAACAAATGAACGAGG
GAGCCCGTAATTTATCCTTTCTTTCATCATCCGCTCTTCCCTTCGATTGTTCATTTCCTATTAA
TTTTTTCATTACGTTATTTCAGTATTTTTCCACACAGCGCTTAGTTCCTGAGAGCCTTCGCGCC
AAGGTAGTCATTTCTCATTTTCATAAGGGAGTGGAGCACGGACGAGGCGTCGATCGATTTA
CCGCATTTTTGCACTATTTTTTCAATCTGTAAAAAGATATTTATTTGAAAATAAATAACTCGA

AAAAAAAAACGTTCGGCGCCTCGCGATCTTCTTATGTCTGCGTATTGCGATCGTATCCTTTC
TTTATTCTCTGTCAACATTTCCTTAATTTATTCTTCTGCTCAGGTTCAGTCATGCGAAGCGAA
ATGACTAGAAAAAAAAAAAAATGACATTCACCTCGAAACAGGATTCCAGTAAAGCTGCTTG
TTTCGTTTCACGTAATATCCGAATGGAAAAATATAGGTCGAAAAGTAACCTTGAAATGGAC
TGACATACCGTGTCATTGCAATCGTCGAGTTTCGAATTATTATGCATTAACCGGTCCATGGA
AATTGTTGACACTTATCTCAGACGGTTAATTGATTGAAAAATTTGCATAGGTAATAAATTTT
TCTTTTTTATTATTATTTAATAAATAATACTTTATAGAGGTTGGCCATTTGGAATTTTCAATTT
TTTTGCAATTCCAAAAATCTCGAAGTCTAAAAGATGACTTGTAAAAAAAATTTAAAGATGT
TGCAATTTAAACTTTAGACTCATTTTAATTCGTGTGATCTCATCATCATTTCTTAACAATTGTT
CGGGAAAGGAAAAAATGAAATTCTTCGTGAAAAAGAATAACTACGAACAACTTCATTTTA
TTAAAATTTCCAGAATATTGAAATTAAATAAATTTATTTAATATAAATAAATATACAATTTC
TTGGCAAAATAAATAGATATTTCTTGAAAAAAAAAAAGAAAGAAAGAAAAAATAAATAG
ATTCTGAATCTCTGAAGAGTTTCGAGAATTTTATGACTTTAGTTTCAAAGTTTATTATTCAGT
CTTCATAGTTAAAATATTTTATTTAATATTTTTTTTCTTTTTTTTTAAGATCCCTTGCAGTATT
AGAAGTTTCACATATCTTTTCAAGACGAGTACAAAAGCCCGAATACAAATTTTATAAAGGA
GAAAATTCTGCAAATAATTATCTGATACTTGTTTAATAAAATGATTATTTATAATAAGATCG
AATGTAATTGCATCCAGTTGAAAAATTTATATCGATGAAATATATGTCGATGAAAAATATTT
CGTCACATCGGCAAATGCATAAAACAAGTGAAACTCGTTGATGAAGAAAGTATTGTCGGT
AATCTCAAGGCAACCCGGTCAAAGAATATTGTTTTTCGGTTGGTCAAGATATACCAAGATTT
GAAATGCACTTTTATATATATATATACATATACATATATATATATATATATATATATATATAT
ATATAATGATTTCATCGGTAACTTACATCATTATGTCTGTATAAGAAAAAGAAAGATTCGA
AGGACTAATTTCTATGAAACACGTGTGTCTGATGTTTCATTAGACTTATTTTGTAACGTGATA
GTCAAGCCGGTCTTCATGCTTCCAAAAATGTCAGAACGTGAAATGAACGTTATTATACAAG
TCATTTTCTGAGAAGTTAAAATTTCGAAAGTTCAAGGTAAAGCGTTTCTCCCGTATTAATTTT
TTTTTTAATAATTCCAACAATCGATACTTAGAAATTTATTAGAAATTATGTAGAAATTGCATT
CCTTTCTCTATTTCTTTTCTTCCTTCCTTTGTTTCTTTAATCCTCTAGATTTTCAAAATAATGTC
TTATTTGTATGGATGTTTTTTGAGGCTTCTTGTGCACGTCTTTGATTCCATTATCCGGCAGATA
TTAACGTTGTGATATTTTATAATATTGTAACGGAACTCGTTATCACTTCTGTATTATATTACA
TGAATGATATTATTTTATAATTATTTCGGAACTGGCTTCTGGAACACTGCGGGAAAATGCGG
CTCTCCGGCGACCTGAATGGTCCCTTTCTTTTTTTTTTTTTTTTTTTTTTTTGTTTGTTTTTTATTT
ATAATTGTTGACGTTTTGTCTGATTCTTATTTGTGAAATGGATGCGAAACTATCATGTGCTG
ATAATACTTTGAAAATCAA

105. >MSTRG.1937.4 Gene=MSTRG.1937
TTAGGTAAAGCTAGGTGCTATTTAGAATGTAAAAGACAATTTTATCTAGAATTCTTATCATT
TCGAATAATTATCTTAAAATTCATTATATAAAAAAATAGAATAAAATTAATTATGTATACA
ATGGTGTATATGGAGTTTCCAAATATTTCATTGGTTTGTACATACATTTCTTGGTTATCTTTA
ATGTTAGCTGTTATGACATATAATGTATATATCACTGTTACTATTGTGCTGGGAGCATGTCTT
GGATATTGGATATTTGGTCCACAACTTATAGAACTTAACATGAAACGATTTTATAAAAGAC
AAATATTATTAGACTGTGATAAAGAATGTGCAGATAACATAATACATAGTCAGAGACATG
AATCAACAGTTTCTGTTGTCGCAGACCAATTAATAACAGAAGCTACTGTTGAAGTACATAT
ACCAAGAGATGCTTGAATCAAACATTTATATTACTAAAAA

106. >MSTRG.1945.3 Gene=MSTRG.1945
GCAAGATGGCGAACGCCGTCACGAGAGTAGTCTTGCGCTGCGAAGATGCACAAGAAACTA
ACAATCTTGGTGCATCGCGGGTTTTATAAAAAATTGTGTACTTTTATTAAAATTTCCCGTTTT
TTGAGTGCAGTTTTTACTTAATGAATTGAATTGAAGTTGATATACAATTTTAAGATTACCGA
ACAATGGTAATATTTGAAAAGTGAACAAAATTCTATCATGAAATATTGATACACTGACCCA
GTTCGACAGCTGATCTGTCAAGATCACAATACGGATGAATACAATGTGCT

107. >MSTRG.1960.1 Gene=MSTRG.1960
AATGTATCCAGTCAGAAAATCGCATTTTGATAGACGCGTAGTATGAATCGTTTTAATCGATA
AACTGCATCGCGTGTCACATTGAATAAAAAATCTCCAATAAATGACAGTGTCCGATTATTAA
TTGACATTTCGACCGTGAAGTTTATTCAGTGTAATCCGATACAACGTAGACGTGAAATATCG
TTAGAATTCAGGTGCCCGACGCGCAATGAATATTGGACTTCATAGTGATTTCTTCTTTTCCG
GTTCTATTGGAACGTATCCCATATTTCTGCAAAATGGCAAAACGCACGAAGAAGGTTGGAA
TCACTGGTAAATATGGTACTCGATATGGTGCTTCCCTTAGAAAAATGGTCAAAAAAATGGA
AATTACTCAGCATAGTAAATATACTTGCACGTTTTGTGGCAAGGATGCTATGAAACGAAGT
GTTGTGGGTATTTGGTCTTGCAAACGATGCAAAAGAACTGTTGCTGGAGGTGCATGGGTAT
ATTCAACAACAGCTGCTGCTTCTGTTAGATCTGCAGTAAGAAGATTACGTGAAGTTAAAGA
ACAGTAAATAAATTTAGTGAATTTCATATAAATA

108. >MSTRG.2107.1 Gene=MSTRG.2107
TTTTTTCTTTTTTTTTTCTTTTTTTAGTTCTATTGTTTGTACATGTTGAACGATACCTTCTCTCGG
GTTGAACACATTTCTGCTTTCGATTTTGATAATAATGTTATATTTGTTTTATATTCGGAACAC
ATTTGTCGATGATTGTTTTATCCACACGAGGATCGATCGATCCGATGACACAATCGAGTCAA
CTGTTTTATATTTTTTAATCGTATTTTAAAAACTTTATCCGTTTGATTCACGTCTACTACTTCC
GTTGTTAGTTATTTTATCGAATTTAAAAATATGCGAATCAGGAACGGACAAGAAGATTAGT
TTAAGTAATTTTCATTATATATATATATATATATAAAGAGAGAGAGAGAGAAGGAGGGAGAG
AAAAAAAAGGAAGAAAAAAGCCGATACCTCGATCGATCGATCGCCAGATCGATCGTCCAA
CGGAAACAAAAAAGTCGCAGGGGAATATCTTGGCTTGCGATCACCGAAATAGAGCGGCCA
TGTTTTTTTTTTTCGAATTCGTCAACTTTTGCCGCCGAGTAAAGGGTGTTCGAAAACCAAGA
AGAGGAAACAGAGTTGCGAGAAACAAAAATGAAAAAAAATATGGGTGTTTCGAGATATTT
TTTGTGAACTGTCTGCCGCCTGCCTGTGCAAAACTACGTTGCCTCGACAAATGTGTCTAATT
TATTGTCGATACGATAATTAGCTGGACTTTTAAGCTGCGTGCGTGCAAAGTGATAATCGAG
AGAAAGAGAGAAAGAGAGAAAGAGATATTGTGTGTGTGTATGCGTGCGAGTGTGCGTGTG
TGTGTGTATGTGTATACGTGCGCGTTCGATGTGAAAGAAGCGGGCAAACAAAAGGGTTGTG
CGAGAATGGATGAAAGAAGGAAAAGCCGGAACAAAGAATGCGAGCGAGCATTGCATCTG
GCACCGGATGGTCTTCGTACCGAGTATATTCGTTCTTTCCCAATTCTTTTTGTTTGTTTGCGCG
GCGAAAAAAAGAAAAAGAGAAAAACGAGAATTCAACGCGATCAAGTTGGGCGATTTAAA
ATTTAATTGAACTTCTTCGTAGTCGTCGAGAAACTAAACTAGTAATAATAGAATTATTCGAA
GAGAAGAGAGAGAGAGAGAGAAAGAGAGAAAAAAAAAAAGATACAGAGAAGAAATACG
AAATACGAAAATATTCCGAGGAAGTGGATTATTTTTTTAAGTTTGGAAAGGACAGCGTGTA
TCGAGACCACTTCCGGCCCGGTTTTTGCGAGCCTGAATTCACACACAATGTTCTTTTTCTCTT
GTGTTTTTTTTTTTTTTCCTTTGAAGCCTGGACTATTGCAACGAGCGATCTCACGTCTATTTAAG
CATAAGTTTACGAGTTTAACGTGGTATTGCTGTATAGTGGCGAGTGAACACGAAGAATTTAT

AAATATGTAATGCCTTAATGTTATATAAACGATAATAATGGTATAGCGTTACTTGTATAATT
AGTTGTGACGCGAGGCTGGAGGATCAAGGAGGATCGTGGATAAGATAGATAGATCGAAGG
GAGGTGGTAAACAGAAAGGGAGAGAAAGAGAGAAATAACGAACGAACGAAGAAAAACG
AGATATATATCGATGTGAAAACAAGAGGACAAAAGTAGAAAACGTAAAAGGGTGATGCG
GTAGGTGCGGCGGTTTGTCTCCATTCTGTTCGTGATTTGATATTTTTTTATAATTCTGACGAC
GAGAGAGAACGAGATTGGAGAGAAAGAGAGAGAGAGAGGGAGTGAGTATAAATGGAGC
GTGTATAGAGAGCGGACTGCTATCTAAAATAGTTCAGGCTAGATGTCGATGTTTGTAGCAG
ATAAAGAACAAAAAAAAAAAAACACAACACAGCATATTGTAATTAAAATTAAAAGTCATTA
ATACCGCCCGCTTTTTTATTCGTCCGATCGATCGATCCAATCTTTTCTCCTTGTCCCTCTTTTT
TTAAATAAAAAATCAGCCGATACATCTATTATTAGATTCGTCCACTCGAAAAGAGTAATTT
AAATACCGCGTTCGTTGTCAATTTTTTTTTTCTTTCTTTTACGAAAACAATCTTCATTAAGAG
GAAAGGAAATATACGAAACATTTTGAAGAGAAAAAACTAGAACTATTCGAATCATTCGTC
GAATCCTAGATTTTTCTATTTCTTCCGCCGATAATTCCTAATTTTTCTTTTTCAAAATCAATTC
TAATGAATAAAACGCACG

109. >MSTRG.2144.3 Gene=MSTRG.2144
AAGAAGAAATGATCTAAAGAAACAATAATCTGGTGTTGAATGAGCAATTGTATTTCTTTTTT
CTTGTTTTGAAACACGTGATACATTTTACAAAGACTGAAGAATTTTAACTCTAGAATGTATT
AAAATTGAGAAGATAAAACAACCAAGTGTCACGCCCAGGAACATTTATGGGCCGTAAACG
GTGGTGGCGTGGAGGTCCAGAGCTGTTGGTGCTCTTCATTCTCCATCATATAATTCTGGGTG
ATGGTGATGTTGGATAGGATTTGAAACGTCGAAAAAAAAAAAGAAAATGAAAAAAGAAAT
GGAAAAAAGATGATGTTCGATTTTCGCCCACGAACTCTTCACTATTATTGAGCTATACATCC
TACGCAACCACCACCTTTGCAAACTCGGTTTCTCTCGTTTGCCCTGAATTCCTTTTCACGCAC
AAATGCTTTTTACTTCTATTTTTTCTTTTTTGTTCTCTCGTTTCCCAT

110. >MSTRG.2145.2 Gene=MSTRG.2145
TATATATTTGAAATAAATTTTTGACTTCTGGTTGATAGTTACTATTACAAAAAGGTATTAAG
CATCAAAGGATAAGTTTAAATAAAAATAAATTCATATTTTTATTGAAATTTAACTTTTATCA
TTAAAGAATTAGGATATGGGTGAACGATATAATATTCATAGTCAGTTAGAACATTTGCAAT
CGAAGTATATTGGCACAGGCCATGCAGACACAACTAAATTTGAATGGCTTGTAAATCAACA
TCGTGATTCTTGCAGTTCTTATATGGGTCATTATGATCTGCTAAATTTTTTCGCTATCGCAGA
AAATGAAGCAAAAGCTCGTGTCCGGTTTAATCTCATGGAAAAAATGTTACAACCTTGCGGT
CCACCACCTGAAAAACCAGAAGATTAATTAATTCAATGAACAACGTGCTTATTCGAAAATA
AATTATTTAGTAAAATTAGAATAACCTTTATAAGAATAAATCATAACACGATATATGGACT

111. >MSTRG.2160.1 Gene=MSTRG.2160
CTTCATCAGGTGCAACGGGATTTAGACGAGGAGCAGAAGTGCTACCAGTAGTTGATTCGTC
TTGACTATCTTCTCCGACTCTCTCGTCTCCACCTGGTTCTTCTTCAACAACCATGTTGGCCAA
AACATGTTCACTATATCTAGTATCCCTCCGTCTAGGTCTTCTTGTAGTTACATCTCCACCTTTT
TGACTTTCTTCGTATAATTCGATTAATCTGACGTCTAAAATGTTCTCTTCAGGTTCCCATGTG
TTATATTTTTTGCTCCATCCCTTCCACTTTACGAAATATTCTACTTTACCCCGCTTTTCCCTCTT
CTTCGTAATCCGTTCCGCAGCGTAAACTCTGTCACCTAGATCCATGATGTTTTAATCCTTGTT
TTTTAGATGCGTATGAAAGTTGTTAATATATCCAATAAGAAAGTGATGCGTGTCACTGAAAT

ACACCACTGAGCAATGTAACACCATATGTAAACACAAAAAACGCGCGTCCCATCGGAAAG
GGAATTTCAAGCGGCACAGTTTGCGCGCCTGAGTTTCTTCTTCGTGTGTTCACGCACAATAA
TCTTGTGTACGATATTCGTCGAGCACTCGAAATACTCGACTGCGTTTCGTTTTGTTTCGAAGT
TGTGTTAGATTTGGTGTCTGTGTTGGCCGGCAACACGTTTTCTGCTTCTGCTGGTGTTGCTGCT
CCCTTCGTGCAAGATCGGGGTTCGTGACGTCAGCAATTCCCCCTCACCTCGTAAACACCAA
ACATGGCCGTCGTACGGATATACGCTGATCGAGTGCGTCCCAAACTGATGGTGAAAATTAA
CGTCGATATAGGCACTAGCAACTTGTGGATCCGCGTACGAAGTGCGGAAAACCGTTTTCAA
GCCCCGTGAACGCTCTACAGTCTCTTTATATGCAAATATTTAAAAACTGGAGCGATCGACTC
ACAGCGTCTAGCACATTCTGCGGATCGAGGCCGACTAGAGCCGAGGTCTGCCGAACTCCCC
TCGCAAGCTTAGTGGACTCGCCCGACCCGCTCGAGGTTAGAAGGGGAGGGGGTAACGGTA
GAGAAAGAGTATCGAAGGGGAAGGGAGAAACTCTCAACGGTACGTGCGCCGCACACGAG
AGAGAGAAAGAGAGAAAGCAAAAGGAGGAGAGTGTGATTGAGTGACCGAGGACGAGAG
GGGTAGGGAGAAGAGGGAGAAAAGAGCAAGCGCAATGGAGGTAGAAAAGAGAGACGGC
ACGACCAGGAGCGCCGTCCTCGGAACAAGAAAGCTGCCTGCCTGCTTGCCTGCCTGCCGCC
TGCTAGCCAGCCACCCCAAGTTCCCTTCTAGGCAGTTTTTCGTATGGACGACTAGCTACCTT
CATCGGTGATTTGTCAAGCATGCACATAAAGATCCGGTAGATGTGGAGTAGTGGACCAGCG
ATATTCGATAAATCAAGGTCGATCGAAGGGAAGGAAAGAAAGGAAGGAAAGAGAAGGAG
CCATCGGGCCGGGATTCTGCCGTGACGCAGAAAAATCAAATTAAACAGCCGCTTTTAAGTC
GAATTAAAACGAATGCGCTTAATTAAAGCGGCCTCCGCCGCTCAACTAAAGGAGTTTTATT
GACAGCGACCGACTCTTCCTTTGTCGGCCCCCACTGTGTACCCTTTCATTATTACGTTAAAA
GATGCCCTCCAATTTTTTTTTCTCTCTCTTTTTAGTTTTTGCCGCAAATAACGCTGCATCTTTTT
TTTCTCTCTCCCCCCTCTCCAAGAGACCGCACGCGGTTTATCTCTTCTCTCTTTCTTTTTTCCC
CTAGTTTTCCTCTCCACGAATACGAAACACGTTTCGTTTCTTTGTTATTCTTTTTTCTTTTTTCA
GGGGTATTTCGATGCGAGATTTTTGTTCTACTCGTTTTCCCGGAAACGTGTGGATTCACGACT
CTTTTGACGAAAAGAAAGAAAAAAAAATGTTAGAAAAGAATTAATTTTCTCTCTTTTTTTTT
TTCTTTACTTACTTCGTTTCTTTTTCCTTGGATGGAATTTATTCAAATTATCGCGAGGTTTCTCT
CGCAGGAGTAAAGTTTAATTGGATCGGA

112. >MSTRG.2169.11 Gene=MSTRG.2169
AGAAAAAAAAAAAAAAATAAAAATACGATTCCTAAGGAGAAATGTTTTAGTGAACCTTTTA
GCGCTCGTGTGTGATAATATCTTGCGGATAGTTTGCAAAATACTCAAATTCGACGTGAGAA
ACGAGATATATGTGTGCATCACGATAAATGGTTAAAGAAAAAGATGAAAAAGAAAAAAA
AAGAGAAGAAGAACGTCGATAAGGAAGATTGATTTAGCTTTCGATTTAGTTGTTTAAAAAA
TGCGTTAGTGTTTGAGATATTTTTATATGTTGACGTTTAAAGTAAAAAAAAAAAAAAAAA
AACGAAAAAAAAGAAAGAAAGAAAGAAAGAAGGGAAAAAAAATAGAGAATTATTATTA
TCATGAAGGGAAGAGAAAGAATTTTTTCAACGAAATATAAGCGAACACACTTCGTTGTCTT
CTTTTTTTATCGACGAAATATATTACGAACAAAAATTAAGGGAAAATATTTAAGAGAAAAT
TAATACGACATTAGAGAGTTATAACGGAAGCTTATGTGCGGTATAATGGCGGCTATTATGT
GATTTATCGATATAATTCCACGGACCGTGATTTTAATCAACTTTTTAAATCGCTAATCCGTTA
CGTAGTTTAATTATAATTAAGCATTGTTCCATTTTTATGCGCGGACAGAGGCGTGTTAATAT
CGAGTCGAAACAGTGTGCGAATAATATCTATGACAATCTTGTTCGAATTGACAAGACAAAC
TATTTTTCCTTTTTACTGCCAGGTATCACTATTTCTATTTGCGGAGCTGCCTTGAGGAGCAAA
CGCTTATTGACATATACTATAATACATACACACACATACACACAGACACACAGAGATAGA

AACAGTGAAGAAAGAAAAAGAATGAAAAAGAAAGAAAAAAAAGAAGTAACGTGTGAGT
GTAAGTGGAGAGATAATAAAATATAAATGTAAAACCTCATTTTCTCTCTCTCCCTCTCTCTC
TCCCTCTCTCTATCTCTGTTCTCGGCGAACAACACGAGAGAGAGAAAGAGAGAGAGAGAA
AGAAAGAAAAAATATCGGAGAAAGGAAGAAAAATGCAATTGTGCAATTTTACACATACAC
GTACACGTACTTTGTACAGAAATAATATACATAGCGCGTATTTTAGTGGTTATTATTATTGTT
ATTATCATCACTGCGATCCTCGTTGATTTTCGATCACGCGTTTCGATCGTGGAAGCATCGAT
CTTGAAACAAAAAAGAAAAAAAATATATATATAAAAATATATATATATATAAATATTTGTC
TACGAATATCGGATATACTCATCGATAGTTCTTCTAAAAATTGATCGGGTAGAGTAGAGAG
AGAGAGAGAGAGAGAATGAATCGGTGATTTGATTAGTAAACACGTAGACATTGTAATTAA
TTGGATTAGATCAAAAGAAAGCGTTTTCTTACGAAAGAGGAGAAAAAGAAAAAAAATGAG
AGAATCGAAACGTGTTTCGATGTCTTGCACGCTTAATGCCCCTCCCCTCGAAGCTTCCTGTC
TCTGCAGCATGTGATCGTCATAAAGCTCGCGTAAATTCGCGTCGAAACCAAATCGAAAATG
GGGAACTCGTATCGATATCTTCTGTTTCGATGAACTTCGTCGTGATCGAGGAACAATAAAA
AAAAAGAATCGAAATCTGAGCTGAAATATATCGAAAACGAAGTTATTCGTTAAAATATATT
GAAACTCGTACCATACTATTTCTATTTTATACATTTTTCTTTTATATTAATTTAAAAATAAGA
GGATATTAAATTCGCAAAGAAGATTCAACAAAAATTATGAAAAATTAATAAAAGAAGATA
AGCTAAGTAAAGTAGCTTATACTCCTCGTTATATATTCAATCTTTTTCCTCAACTTTCGAAAA
CTTTCTAAATATTTGAAACTCGATCCTCGAACGAATCTCCATCGATATCGGAAAATTTAATT
CGAGTTTCCCGCTGCGGACGTAAGTCGTTACAGCGTGTCCATCTTCTATCCTTCTAAAATAG
TAAACTTAGTTTTAACGATCGTCCAATGGGGTGAGAGGATCGGATGATCGATCTCTCTCGTA
AACCCGTAGGTGAACTCAGTTGTTCGATATTATAAGCAAAGAAGCAAAGAAAATTGAAAA
AAAAAACAGACAAACAAACAAAAAAAAAAAGAAAAATGAAGTAAAAAAAAAAAAAAAA
TAAAATAAAAATAAAAAAGAATTTTTCAACTTCGCGATCGAGAAGGTGTCAGGCAGAAAG
GAGAGAACGATTGAGAAAGAGGGGAAAAGTAAGAGTGAAATACGAAGACCTTCTCTTAA
GCTAGAACCACGGAGACGCAGGTGATTAAAGGAACAATGTAAGATAGGGAGATGAAGCG
TCTCTCTCGTTCCATTGGTCGACCGCTGTGGCTACCGTGTAGTCGAACAAATATATCCATATT
AATTTCCGAATTGCCTGATGCGTGGTTGTATGTGTGCACCGTGATGCGTGAGAGTGTTTTAA
TTGTATTCGAGAATAGTTGAGAAAATGCACGAGAGTGAAGAACGAACGAGAGAGCGACGT
TGAAGGTCGTTATAGTATTTTAAAGGATTGAAAACGAAATTTTTCTTTTTTTTTTTTTGAGGA
GGAGGAGGAGGAGGAGGAGGAGGATGAGGACGAGAGTGAAAATGGGGAAAAAAGTCGA
GGAGAGAGCATGGAACAAATGTGAAATTCTACTCGACGACGTTTTCTCATTTTCCTTTCTTT
ACTTTTTTCCTCTATTTTTTTCTTTTTTTTTGTTTTTTGTTTATATATATATATATATTTTTTTTTCT
TTCTTTCCCCGAATAATTTTTCCACCATTTTCGTCGTAGAGGAGCCACTCTCGAGGACGGAT
GATTTAGGAGCGACGTGTACAAAACACGAAAAGCGAAACATATAACGATTTAAATAAATA
TAAACAATTGTAAATAATTAAACCGAGAGAGGAGACGAGGCGATTTTTTAGGCGTGACGA
ATGATTTAAAGAAAAAAAAAAAAAAAAAAAAAAAAAAGGAAAAAAAAGATTCAAAGATA
AAAAAGAGAGCGCATTGTAAAACACATCGCAGTAGTGAACCACGCTACACGCTAAAACGA
TATTACGCATGGCAGAAACGAGTGAATATACTTATAAATATTTGATATTAGATAAACGAGG
ATAAAGAAAAAAAAAAAAGATAATGAAGAAAAATGACGCCCTAACGCGAGCGATACACAC
CATATACATATATAAATATTATACATATATACATAGATATATATATATATATTATTATATCG
AGGCGTACAAGCAGTATGTGCGTTTTCCAGCACAGATAGTTGGTATTTTGTGTACAAAAAA
AAAGAAAAAAAGAAAGAAAGAAACAAGAAGAAGAAAAAGAAGAAAAAGAGGAAGGGA
GAAAAAATTTTTTGCGAATAAAAGCGAACCGGATGAACAATTGTTGGATATTTTCGCCGCG

CCCTGTCCAGTCTGCGTGATTATTATTATTTTTATTATTATATTATTATTATATTCGAATTATT
ATGATTCTCTAATTATTATTCTATTATTATTATAATTATAATTATTATTATTATTATTATGTAG
CATGATTTGCGTAGGAGAGGCGCGATGAAAAGATAATTTGTCAGCGCTGATAAGCGTCACT
TTTCGTGTCGATCGTTCGCGATGGTCGAACGAGAACGTTCCCACGTTTTCCCAGAGCAAACC
GAAAATGCAGATTACTCCCTTCTCGCAACGAAGATTATCCTCTCATATTAACGAATGTCTCT
GCCACGATCGATCCATCGATCGATCGAATCCATCGAAATTCGCCATTCACTTTTTTGCTCTC
AGCCATCTTTGCCATTTGTGCAATTTCGATTTGCTCTTCTACAGGTATCCTCGATTTCTTCACG
CTCCGCGGATATTTGCGATAATCTAATTATAGTACAACGTGTAACAACGTGTGTGCGTGTGT
ATAAATTAACTAATTGACGTTACGAACATTTAACCTTTGCTATTTGATTTGTTTGTTGAAAGT
ATTGAAAAGAAGAGGATTCTATATTTTAGGATTCTATCTCTTCATTTTTAAAAGTACGATAA
AAAATATTTGGAAAAGAGAAAGAATTATAAAGGGATAGAGATATAAGGGATATTGGTGTC
TGTTTTAAAATTTATAAATTTATAAATTATTCGTAAATTTTTTCAAAGAATTCTAAAGATAAA
ATCCTTGACCATGTCGCTTTAAATGTTTGACAACCATATCTCGATTGAAAAGGGATAAAAA
AAAAAAATAAGTTAAACTATCCAAAAGCGTCGTTTGCCAAATGAATCCAAACTTGAGAGA
ATTTGCGCGGAGCGTGTTAACTCGAGGGCGCCATCTACACAGCGAGAAGGTGGAACTAAA
AGAGGGGGAAATGAAGTGAGGCTGTAAAAAGAAATTGTCGCGGTGGCCGTGGCAGACAC
GGCCGATTCTACTTGTATCATAATCTCTTACGATAGTGCGCTATAATTAAACAATAACGAAG
AAAACAGTAACAACAATAAGATAACTCGTAATTTAAGGGAGCGTGAAGCGGAATGAAAG
AGCGAGAGAATGCGAGCGGAAGAGCGTATGTATGCCGATTAATCACGTGCAAACCGATTT
AGTGATCGATTGTGATCGCTAAGAGAAGCACCCTCGTAGCTACGAACGGGCTTTTCACGGC
TTTCAGTCGCTGGTCTCGCGATTTTCCTCACTTCTGCTCAACCTTGATCTCTCCAATTACGAA
ACAATTTATTCACCGGGCGGATTTTCGAACAATTTAAAAAAAAAAAAAAAAGAAAAAGAAA
AAGTAGCAAAAAACGAGAAGAATCTGAAACGGTCTCTTCCCTCTTCTGTGGTGGAAAATAG
AGTTAGAGGGAGGGAAGAAATATAGACGAAGAGACCGAAACAAAAGAGAACAAAATGG
CGCGAGACCAGTGTCTGCGGCTAGCCCAAAGGATTTTTACCAAAACGACTCCGCGATTCGC
GGAGTGGAAGCAGTTATATAAAAAAAAAAAAAGAAAAAAAAAATAGACGTAATTATTGAAT
TTCGTTTCGATTTTGTAAACGAAGTCGAGTGCGCGAAATTCAGCCGGCGATTAACCGTGCCT
CCTCGAAGGGCTCGGTTTTATGCCGAGGAAAATAAATAATACGGGGACAAACGTGTGTTTT
CGCCGGCTTTTCGCGGATGGCGAATTTTGTAAAACGCGGACGCGTCCCGCGAACGATCCCT
GGCACGTCGAAAATTGCACGATCTCATTGATTTTATAAGCATGAAACATTTTAATTATATAT
TTTATTCCCTTTTAATCGAACATTGAGTTTTTTTAATCGACTTGTCTATATATTTTATATACGC
GTATTGGCTGTTGATAAAATTATCGTATATCTATATTTATTATTATTGTTTAAAACAAATTTG
TCGCGTGAATCGAATGAATGATAAAAAACGTGATTTTCTTCAAATTTTATATTTTAAAAGAC
GAGTCTTATTTTTTTTTAAAATTGTTTCTTTTAAAAATGTTATTTGAACAGAGATTTGTTATGA
TCCTTCAAATTACAATTAATTGTATCATTTTTGAGGGGTGTTTTTGTCAAAGATTTATTGTTTT
TTTTTTTTTTTTAATAATGTTGTACCTTTTTTTTCTTTTTTTTTTTATACATATATATGTATTTGTG
TTTTATTCAAGCGTGAGAAAGAAACATTCGACCAGTGATCGCTTGCCGACGCGTCATTCGG
GACGTAATATGAAGGAATGCGTTGTATTATATATCGAGAAACCCTCTAAGTGTAAGAATAA
TAATGTATATATAATTATTATTATACCATAAAAACTTCGATTGATTGCACTCCGGGGAAAAG
GAAACTGTCTCCCGCTCCCAACGTCTATGAAGAAGGAACAAAAAGCGAAAAAAATTGAAA
AATTGAAACGCACGAAAAATGAACGAAGACGAAGCAATCTTCAAATCTTCAAAAAGAAA
GATTTAAAAAAAAGAAAAAAAAAAAAGATAAAACGTACATTCATGGATACAAAATGGGG
AAGAAAAATGAAACGATAAAAAAAAGAAAAAGAAACTGTTCTCATGATTGATTTCAAAAT

GAAGAAACGAATAAGATGAAGAAGATGAGGAAAAAAAAAAAAGAAAAGACGCTGCGTTTT
TCGATCCAGATTCGTTTTGTTTTTTGTTGTTAGTTTGTACGCACTACGATTTATCGCAATTTAT
TGTATTATCGATGAAATACTGTAACAAAAATACTGTTAACTATTCTTAAAAAAATTAAAACT
TTTGTAAACTGAGACCAACCGCGAATTTCTCACGACATACTCTCCTGAAACATAATTCCAAT
AGGATTCATTAAGTTGTCGAGCAGCATTCTGGTGTAAAAGGTAGGGATAAAAGATAAAAA
CTCGAGAAATTGCACATTCATAAAGATATTTTATTTTTATAATTTTATAATTGGATATACATT
TTACTTTCATGTGTTTTATTTTTTATTTTCACACGATGCATTATCACCACGATATGGGTATAA
AGCAGTACAACACTGCACACAGAAGCTACAACCGCACATTGACTCATTATACATTAAACAT
TTTCACGGTACAAAAGCTTCGCAATTTATTCTTAAAAATATACGATAAAAAAATCTCTGAAT
TCTTCGCAACAATTTTCTTCAAAAAAATAGCACCAGAGGAGATAAAGAGATTCATATTTTAC
AACGAGTTATATAGTTAAAAAAATATATCGATCTTCGAATTATAACGTAATATATAGATAG
TTCAAAAATCCAGTTGTTAAGTTGATTCGCTTAAAAAGTAATGGATGGCATCGACGACGTT
GCTCGATATAAATCTACGACGAGCAACAAGCTAACATTAAAAAAGCTACATTAAAAAAAA
AAATAAAATAAAATGAAATAAAAAACTAGGG

113. >MSTRG.2199.1 Gene=MSTRG.2199
AGCGATCCTTTGTAAAACGTGAACGATCAAAATTTTGTTTCCTTTTTCTCCGTACATATTTTC
CTTTTCCATCTTCTTATTTTGCTATCGTTTTTTTCGATTATACAAGAAAAAAAAAAAAACAATT
GGTGTTTAATTTACGCGAATTGTGCGGACGATCGAAAGCGAGATGGAGAGCAACATTCAGT
TCGACAACATCAGTTTAACCTCTAAAGTGCAAAGTCGGCGAACGCGGTCTTAGGTCGTCGA
GAGGAGGCTAGCAAGCAGCCCGAGCTCGACACACACGAAGATAGCTTTTATGACTTGCCT
GCGTCCTTCCAGGAAGAACAATCGGTCAAACCGAATATTCGATTCGTCTTCTCGGTCTTCCG
TTCGTCCATCGACGCGGATCATGCGCGTATCCCACGCTAAGGTCCGTGTTTCTCTCATCGTG
AACCTCGTTTTCCGGGAGAGAGAAAATCGAGGGGAACACCGAGGGGAAAAAACACGACT
ATATTTTACAAGAGGAATAAAAGACGAAGATAGAGAAAAATAGAGAAGAAGTGTTTCGGT
GACATGCGACACACACTGAACTCTCCCCACACCGAACAGATTTATCACGTTATCGATCCTTT
TGTCGCGATGCTTCCTTACGGAGATCTGTTTGTGGATCAGGTCGAACGGTTTCGCAAAATGG
TGGACGATATTGTAGAGTACGTATATTCGCTGATCCGTGACCGATGATACGCAACGCGAAC
CATACGATTCGCGCGCGTGTATATCGCGCGAGTATATTGTCGCCATCGCCGCGGCAACAAC
AACAACAACAATTTCGTCACGATTGCGTCGATGATAATGACGATGACGACGACGACGACT
CTGTTTGACTCTGTTACTGACTGAACAAAGTTTTGACTGGCTATGTGTTTGACTGTGTGTGTG
TTGGAAATTGGCAATATTTCGTATTTGCTGGCAGTTGGCAGAGAAGTATTAATGTAGCATTG
GCAAACTCTGGGGTCGGCTCGGCGACGGTCTCCGCCCGCGGACGTACGAACGGAGCGGCC
GCTTGGCCTAGTCGCCGAAACGACTGCAGGCCTTCTTCCATTTGCACTGTGTGCACCATAGG
TCGCGATGCTCCATACCGTAGACCTTGCGGCACTTCTTCGTCTCGCCGCGAATCCGTCGGGG
CGACGATGGCATCTTTCCCGTGGACACCGTCAGCAGGCCCATGCTCTGTTGGTACGCCTGG
AATGGCCTGGGTGACAACTTTATATGCTTGATCGGTGTACCCGGTGATTGCGAAGTGGCGA
ATGCGATTCGTTCTCTGATGCTCACCATCATGCTTTCTGCGTTATTGGTGGCAGAAGTGGTTT
CCAAACGAAGCTGTTTCTGGTACTCTGGATCCTCGTGAGGAGGTCTGGCCATGTCCCTGTGC
GACATAGTCGGCGGAGAGCTCATATGATCCACCGCCACCTCCTGATAGTAGAATTCTTCCT
CGTGATCGGAAATGTCATCGTCCTCTTCGTTATCGTAACTGTTAGTCTTTTTAGGTCCTAAGT
GTGATTGGCGCACGTGCTCCTCGATAAGCGGCACGGTCGCCTTTATTTCTAAACAACCTGGC
CAAGTACATTTAAATACTACCGCTGCATTAGCCTGTTCGCTCTCGTAGACGGCGCTGTTAGC

TGGTCCATTCTCGATCGGGTCGCGGCTCACTTTCGCCCGGATCTTCTTCTTTCGGGGGTGTTG
CTCCTCGAGGTAGTCGGGCACTATGCCCTCGTCCAGAGTGGTGGTCGAGCCTGGGGTGCTG
CTCGACGACGATCCGGACACGTTGTAAGGGTAGTGCTGTTGATTGTGCGAGGAGTGGGCCG
AGGTCGGCCACAGGGAACTGGGCGCCCCCTCGTCGCTGAGGGGGGGGCTCGGGGTGCCGC
TACGCCACGAAGCCCCGCTGCTGCTGCTGCTGTTGGACGTGCCGCCGACTCCCGGCGAGCC
GACCACCATGCCGCCACCTCCGCCTCCTCGACCTCCCCAGGAACCGTAATTATGTTGGCAG
TGTAACGGTAAAGGATTGTGAGGGCTGTGTGGCGAGCACGAGAGCGACATGAGAACTAGC
GCGGCTGTGCATTCGTCCATGCAGTCGCCGCGCTCGTTTGTGCGACACCCCTCGCGACCACA
TCCGTGGGGTATTCGCTCGCTCCAACCGCTGAACGATCGTTCAGAATCGTTGCTCGAGCTGG
GTCGCCGCTTTCTGGATCCAATCACGTGTGGCACGTCGATGGAGTGGGAAGATGCCCGTTTT
CGATCACCCGCCATATCGGCGAGCCTAGCGAAATCCGTGTCCTGATCGGCCAGCCGTGCGC
TCTTACGCGACTCCAGCAACCTGACCTCGTCTATACGCTTGGTCAGCCTCATCGTCTCTTGA
CCGTTTGGTGCAATTATAACATCCACTTCGTCTTGATGTTCACGATGTTGCGTGACCTCCGCG
TGAATTTCCCGTCCGTTATACGTCAAATAGACCTTCTGGCCGGGCACGAGGCGTGCGCTGGT
TACGGAACCGAAGCCTGGTCCGATCAGGTCGCGATCAGAGTATTCGGTGCTGGGACTTGGC
GGACGACCGCCAGGCACGGAATCGAAGCGTACGGAATAACGAGTCTTCGGTGTAATACTG
AGGCCTGACTCGGACGCGGCTGACGCCGGCGTTTTTACGGCGTGAATGACGCCACTATAGT
ATTTCCCGTCCTGTCCGGGAGCGCACACTCTGGTGCCGATTATGCTACGTTTCGCCAGCCTC
TTACCCGTCGACATTATTCCGTGGTTATTTATGGATAAACGATCGTTTCGATCACTTTATTAA
ATCGTTTGACGAGGATGCACTGATATTATTTCTTACGATAAAACACCTTATAACAAATCGTT
CTCAAAAGTATCTCGCGCAAATTGGTATTTCATAAAGATCATTTGCACTACAATTTAACTCA
CCCTTCTATTATCGTCTTTGAAATTTTCGTTGATAAATATACGTATACGTATATCGTGAACAA
ACCGGAAGCAAATATATATTAATATCACACCAAAGAGAAATAACAATTTCACGCGCGATTT
AACAGTTAAACACTTGTGAAGAGACGATACGAGTGTATCTATGATTTGATTGGAATATTAG
CAAAAAAACACTCCCGGAAATAAAACACTTGTTATACTCTTTTTTCTTCCTTCCTATCGAAG
TTCCGTACTGCAACACGGTTACTTTAAGTCATTGTTCCACGATCACTCGTATACACACTGAG
GACAGATCGGGCAGTTTTGCGTTAAATTGCAATTGCGTTAGCAAATGCGATCCTAGACCGA
AGAAGGACGCGGTGGCCTTCGCGGCCTGAACACAGAACTCAATGTATCAACAAAACGGCG
TGCACCGTTGCCTGTGTTCGTCACGCCGTCTGCATGTTCGAGGCACTTCGTGATTGATTTGTG
ATAATACGTTGGAGTATACGCGGTAGATAGCATAATATATGACTAATGCTTGCCAGTGAAT
AGAAACTCAGCGGAATTCTGTAAGCTGAACGTACAAGCGGTACACGATTCGATGGTATAC
GGACAGGAGTAGGGTCCGC

114. >MSTRG.2242.11 Gene=MSTRG.2242
CGAACTCAAACTCGGAGGAGGGATCCATCGAAACATCGGTCGTCGAGCAGTGTCGCCCCC
TCGAATCCTCGAAGGGGATTTCGGAGGTTGAGAAATTCGCGCGAGATAATACGAAACGAA
GTCGGGCGGCGGTCGAGCGGAACAAGTGGTTGGTTAAACTGTGATGACCGGGCACCGGTC
GGGCAAAGTACTGGAGTGCAAAGCCTCGATCCCATCGTGCACCGTCCGGATACCGGTGCA
CAGGCCAACGATCGAGAGAAGAGGAAAGAAACGAGCCCCGACGACGATCAACGGACAA
ATATTATTCTTATTATCGTTGTTATTACCATCGTCGAGCGTCGGGCCGAAGCGTTGAGGAGA
CGGGGTTATCACGACGAGGCAGGAGGCTGGCAGGACGGTGGAACGATGCCCACCACCGGC
GTGGATTCGCAGCAGCGAACGCGCAGTTTTTCGTAGGAAACGGGAGCACGGCCGATCACG
AAAGCGGCGACACGAGGGCCGACGACGAAAGGAAGACGAGGCGAGGAGGGACGCGGCA

AGGTTGGAACGCGGCGGCGGAGTCGGCCGGCTCGAAGCGGAGGGAAAGCGAGCAGACGG
AGGAAAGGACAGGAAGAGGCGAGCAGCCGGAGAGTGAGCGGTCTATGACGTTACCGGTG
GCCGTCTACGTCACGTTAACGGTAACGGTAATCCTCGTATACACGACACGCACGCGACCTA
GTCGCGGCGCTGAGCGGAGCAAGTGGAGAGTAGTATAGCGGGTAGCGAGCTAGCGAGCGA
CCGCCTCGAGGAATTTAAAAGTGAAACTGTGACGATCCACCGCCACTCCACGCGTTTCCCC
CTTCTCTCCTATTTGCTATCGTTCTCCCTCGACTTCTTCTTTTTCTTCTACTTCTACTTCTTTTTT
CTCTCTCGCTTTCTTTCCCTCCCTTCCAACGTCTTCCCTCGTCCTCCTCCTCTCCCTTCTCCTCG
TTCTTCGCTCCTTTCTCTTTCTCTGGCCGCCGCTTTGTTTCCTCTACTCCCTCATCACGGTTTCG
CCCGTCGTCTGTCTGCGTTCCTCTTCTCTTCGTCTCTCGGCCCCCTTCCCGCACGCCCCTCGGT
CGCCTCGTGATCGTGACACGGAAGGGAACGCGCGGCGCGCAGTGAAAGTGATCGACATTA
CGATTTTTCCGGCTAGCGAACCCACCCCCCCTCTCTGGGCTCGCCTCGAGAAAAAGGCGGA
TCGTCCGCGCACGAGAATGATGACACGCAGGTGCGAGGAGACGGGGAGCAGCTGAAGGC
GGCCGCCCGATCAACGTGTCGTCCACTTCCGGAACACAGGTGGGGGGAAAGAGGCTACGC
TAACAGCTGCCGCTCACGTTGATGGCCGATCATCGACGAACCAAAAATGTAAGCTCCCATC
ATATATATTTTCGTTCAACCAGCTTATCCAATTAATCGAAATAACTAAGTAAGTATTTATCA
CCTCATTCTCTCTCTCTCTCTCTCTTTCTCTCTCTCAAAACTTAAGATTACAAAATGCAAAAT
TTTTGAAATTCACTCCATCCGCTCCTTTCTCCCTCCCCTTCTTTCATCCTCTTTCTTAATACGC
GAGGTCAAGTACAGAGCGTGGATGTTGCACGGAAGGCAAGAGAAAAAGAGAGCGCATCA
TAGAAAGAAGGAAGAAAGGAAGGAAGGAAGGACAGGACAGGAGAAGATAGAACGTGGA
TAGAATCGGAATCGAGTGTGTGTGAACGAAGGGTGAACGACCGAAAGGTA

115. >MSTRG.2265.2 Gene=MSTRG.2265
GAACAAGAACTTTAAGGACGAGCGTGGTTATCGTTCCGTTCTGAGGAGGCGATCGGGAGG
GGACGAGGCGAATTACAGGAACCAGGACGAGGAGGAGGACTTGGAGGACGAGGACGAGG
ACGGGGACGACGAGAAGAGGGTTTTCTTCGCCGTTGGGAGGCCCGATGATGACGAGGACG
ATGACGACGACGACGAGGACACGGTGAACGAAACGGAGGAACCACTTGGCGATTACGAA
TCTACCGGTGGAACTGTACAGATTGGCCTCGCCCTGGAACTTGTTGCACTCAGCATCTTATT
CTGGTTATTTTAAACGGACAAGTAGGATGAAGATAGAAGATTGAATTTTGGAATTAATGTA
TAATTAATTTTATTTTACTCTAATAAATTTGTATTTAAAAGATGTAAGTTGGGTGTAGAATGA
TAGAATTATTAGAGACCGAGTTGAAGTAGGATCAAAATATATGATGGCATTAAGATGGGA
G

116. >MSTRG.2278.2 Gene=MSTRG.2278
GAGAGGAATGATACGGGAGGATGGGAAGAGTAATTAACGGCTTAATTATACGGCGAAGAG
TGTTCATTGCTAAAGCTTAAATAGAGACAATACGTAGAGCGTATTTTCCACGGCCGGCCGT
GAATGTTGATAACCTGAATAGTGTTTTCGAGAATCGGCGAATGATGACGTTTTCGATTGCCA
ATTTGGAAAGAAAGAGAGGCCACTTTTATATTTCGTTTCGTTGACGCGACGATCCTGCGTAT
AGAAGCGTCAGGTTTTTTCGTGTTCGCTCGAAAACTGGTCGAACACGATCCGATCCGTGTGT
CTGCGCGACTAAACAAATATAACTAAGAGAGGTGGTTCGATGCTGGTTGTATGTGCACCTT
TCGAGGAACGCAATTGTGTACACTGTCACGGTCTTCATTTGCGTTCAATCCCCTCTCGTCTCG
TTTCCTTCTTCGATCGACTTTCATGCACGGTTCATTGAATAGCTAGAAGTTTGCTAATCCCTC
GTGTTTTAACGGCGATATCTATAGTATTTCGTCTCGATCGAAACTATCGATCGAAAATATTC
CTCGAGTTATTACCAAACAGATTATTAATATATATATCTACGGAAGAATTAACTTACTACAG

CTGATTCAATAATTTTTGACGTAATCACGTTAGATAATTCCTCGAGTAGAGTAAACACAGA
GAGTATTACTTCGGTACAACTTCTCGACAAATTTCACGATTTCTCTTTCCGTGTAAACTCTAA
ATCTCTAACGCGGTTAAAACCATTCCCGTCATTACGGGTCAAAATTTGTTTTGGAACAGATT
CGTGTTCACCGATTACAACGTCCTTTTACCGCACTCTATCCTCGTTTTTTTTTTTTTTTCTTTTT
TTCTCTTTTCCTCGCGTTTACCGCGTATGACGCAATCGTTATACGAAACCGACTGTCCCCCCC
CTCTCTCTCTCTGTTTTCGATTTTTAACGAGAGAAGGTGAGAGCGTGTATGGAAAAAGGGT
CGAAGGACGCGGGGAGGGGGAGGGGGGATCGATAAATATCCCATCGTGATTTTCATGGGC
TGCGTTGATCGCGCAGCGAGACTTCCACTTTTCTACGGCCGAGAGTTTGCACGATTTCGTTA
CGTAATCCATTTATCGGGAAGTCGAGGTTGAGTCGGAGCCGATGTCGAAATACGACATCGA
AGGGAGGCGACTAACTCGCGCGCCACGACTCGCGTTTCACAAATCTCTTCGATCGCGATGT
GTTCTCGTTGTATCGCATTGGCGTGTGACCATTGGCTGCTGCACCATGATGACGTGCCATTC
ACCGTGAAAACCATTCATTCAAGATCACGAGAAGGAAGGATATGATAAATTGTGGCCGAT
GAAGAAAAGAGCACGAGCACGAGTACGAGCAGGAGCACGAGCAAGAAGAGCAAGCGGC
TTCGTCGAGGAAGGCGGGAAGAGGCGACGACGAGGCGGCACAAGCAATACCGATAAAAA
GATCAACACTAGATCTGATGTTAGCGGACATTAACGGTAACTTGGCGGATCTGAGAAGCGA
AGCGATGGCGTCGCAGAGGTTTTGTCTGCGTTGGAATAATCATCAAAGTAATCTACTCTCCG

117. >MSTRG.2279.1 Gene=MSTRG.2279
GCCATCGCTTCGCTTCTCAGATCCGCCAAGTTACCGTTAATGTCCGCTAACATCAGATCTAG
TGTTGATCTTTTTATCGGTATTGCTTGTGCCGCCTCGTCGTCGCCTCTTCCCGCCTTCCTCGAC
GAAGCCGCTTGCTCTTCTTGCTCGTGCTCCTGCTCGTACTCGTGCTCGTGCTCTTTTCTTCATC
GGCCACAATTTATCATATCCTTCCTTCTCGTGATTTGTATCGCGTTGCCTCGGCTGGTCACAC
GAATGTGCGCGCACACATTTACCAACAGAG

118. >MSTRG.2304.1 Gene=MSTRG.2304
CCCATTACTTCCATAGGTACACCAGCACGTCCATGTTTAAGCATTTTTAAAAGAGCTAATGA
CGATATGTATACCTGCTCTGCAGTATCAACAACTGGAGCATCTGAAGGTGGAGGTCCTTGA
CTAAGACCTGGCATAGCACCTCCTAATCTCAATAATCGATCCATCACTATTTATTTTTTATTT
CTTTTAATAATTCTATTAAATTATATCACTGATTGAAAATACTACGATTTATCAGTCATCCGT
AGCATTAAACAAACATATCTTTTGTAAGCTAATGCATTGACAACTCTCAAATCTGCACACTC
ATGAGCGCAGCCTATTGTAGCGACTAGCAGTTACAGAATTTTTAGTATCGTTATCATAGTTT
AATAATAATGAAAGAAGTTAAAAATTCGAATCGTCCAAGATATGGAGGTGGACCGCATAA
TTTGGGAGATCCAGAAGATAAACATTTAAGAAAAGTAGAAAAAGATGTTCTCATACCACA
AAAAATGCGAGATAAAGCAAAACAAGAATGTGTGCAAGAGGTTCATGAATTCAATGTGTG
TTGTAAAAATGCTAGTTATCTTATGGCATTTAAATGTAAGAAAGAAACTAATGCATTAATA
AACTGTTTATCAAAATGGTATAATGATCCAAAATTCAAAGAAGAATGTACAGAAGAATATT
TAGCAGAAAGAAGCGAGTATAGAAGAACTGGTATTCCTAAAAAATCAAAATTAGGGAGAT
TTGGATCTTCAAATTAAATTATATAATGAATAATATATTAAATATATATTAGTATTGTTATTT
TTTATTTAGCATATGTAATTGTTTATTGTAAATGAAATGAAGCTATTAAAATGCTATTAAAA
TATTATAATTATATAAAAAAAAAAAGAATTTTAAACTTATGTACTTTATTTACAATTATGAA
AAATATGTTAAATACAAAATTAACATGAATCATCA

119. >MSTRG.2304.3 Gene=MSTRG.2304

AGCCTATTGTAGCGACTAGCAGTTACAGAATTTTTAGTATCGTTATCATAGTTTAATAATAA
TGAAAGAAGTTAAAAATTCGAATCGTCCAAGATATGGAGGTGGACCGCATAATTTGGAATT
CAATGTGTGTTGTAAAAATGCTAGTTATCTTATGGCATTTAAATGTAAGAAAGAAACTAAT
GCATTAATAAACTGTTTATCAAAATGGTATAATGATCCAAAATTCAAAGAAGAATGTACAG
AAGAATATTTAGCAGAAAGAAGCGAGTATAGAAGAACTGGTATTC

120. >MSTRG.2322.1 Gene=MSTRG.2322
ATTAGGATTTGAACTTTTTTTCTATTCCTTTCAACGATGAAAATGAAAAACTCGAAACACCG
ATAATAATAAATCATCGAATAAAAATTAAAATTTTTCGATGATTTATCGATGAAAAGTTCG
AAAGCTAATTTAATATTTCGATACAGGAACGATATTCCAAATTCGTTCACTCGTGGAGAAA
TAATTAAACTAATGACGAAAAAGAGGTTATTACGCAAAAGTAAAGTAGGTTAGGTTAAGT
AGAGTTTACGAGTCGCGGTTGATCGTCATTTCTAAAATTGGCAATTTGATTACACAATCTAA
TATAATACTCTTTGTGTAACCTTGGCCGTTGCAGTTAATTACTTGTTGAATCGAGATTTCAAC
TCCTCTTCTATCTTTCGATATCCTTCAATGAAGAACTCGAGATAATAATGAATCATCGAACA
ATAAATTATTCAACTTTACTCTTCGTTTGGGACGAAAAATATGTATTTCGAACGGGTTAAAT
AAATTTAAAAATTCATTTCGACAATTAAAATAATCTCTCGTTTCGTTCGATTGCTATTGCGCA
ATTGCGATAAATTTCCAATTTAAATGGAAAAAGAAAATTTTTACCGCTCGTTAAATTCTTTC
TCGCGGTGTACAACGGCGAAAATTACATCCGAGAACTCGTCTCCATGGTCGAGGCGAAGA
AAAAGTTAAACCGAGAAGGGAAAATCCCCTCCCATTCGTCTCGGTTGCCGCCGTAAAATTC
CTATTAGACGAAGGAAGAAAGCCGCGATGCTAAAAATTCCCCCTCCACTAACGAAAATAT
ATCGTGTTGGGGAATCGACCCAACCTATATATTCCGATACATGTATAAAATATCACGCTTGC
GTAAATATTAATATTGATAATTAATCCCGTCGAATCAATTAATCCTCTCCTTTTGATCGGGTT
CTCTTTGAAAGGGAGCACGAAGGATCCTCGAGGGTTTTAATCGAACGAACCGTTTGCTCGG
GCGGCGTGGAATATGAGAGAATTCGCGGGTAAACCGGGTATCCTTTGATCGAAATATTCCC
GAAAACGTGAAGGCAATATGACCGAAAACTTGTATCTTCCATCCATGTTTCGCTGATAACC
GCGAAAAGTACAGCCAAGTTTTCAACCGAGTTTCGAAAAAAAAAAAAAAGAAAAAAAAA
ATCAGCTTTTCATTTGTTTCATTTTGTTGTTAAAACGCGACAGCGACAGCGAAAAGTGATTTT
CGTTAATCCTTGGTATTACGGATTATATTTGATGTTAAATTAAAAGTAGGTGTTTGTTTGACA
AAAATAAATTAATGAATAAACAAAATGCGCGAATAAAAAGGGCGTTTATTTATAAATCTAT
AACGTCAAAAGTGGGAGCTGTCAGCGGAGAAAATAAAATAAGTGAAATATTACAGCTCAC
CTCTCGGTTTTCAGTTTTATATATATACAAGCGCGCGTACGTGGCGGTGCGTGTGAATTTTCA
CTTGAAAAATTAGCATCAATTTGTAACTCGGTCGGGAGGGGGGAGGGAAAAACAAAATAT
TCTGTCCGAACGGTAATTTAAAATTTTCTGCGAAATTCTCGACATTATTCTCGAATGGGCGC
GAACAAACGATGAACTACCACGTTCGCGCACGAGAACGTTTCTCTTGAATTTAAAATCGGT
AATAAAACGTCGTATCGAGATATATAAATATGCTCGTTTGTTTCGAAGAAGAACGAGATTT
AGAAATGACAATTGTTTATCTTCTCTTCGAGAGGAATAATCAATTGAAATATGACGTGAAA
CGGGATGCGTAATTTATCTTGATAAATCTATCCGTGCATTTCGATCGAGATTGAATAACAAG
GTTTTCATTAAATTGTTTTCGTTAAATGCGCATGCGCGCGTTAAAACGATTTATCTATGTCGA
AACGTATTCGCCGATTTCGGTGGGTCGCGCGTATATAAATACGAACATGGATCGTAGTATC
GTGTTGTGTAACTATATGTGTGTGTGTTGTATATGCCAAGTATATCCGAAAGGTGGAAGGGG
AAAGTGACTTTGGGCATCTTATCTGGATATTTAATCTTTACGATGCTACCTTTACCACGATAT
TTCAAATATCGAAGAGAGAAGAAACGGACGGTTTTATTAACCGTAGAGAATACCTTCTAGA
AGATTTCTTCTTTATCTTCGAAAATTGCGCAGGAAAACAAAGCAAGCCAAGAACATAATTC

GACGATGGTACGATTTTCTCGATTTCGATCGAGAAAATGTATCCTAAAAATTGCTTCTGGAT
TAGAAATTTATTAAAATTTTTCCTTTTTTTTTTTTTTTTTAAATTAATCTACAAGAGTAGAGCT
ACTAGTAGAGATCGTAAAATCTTACGATCGTGAGGGAAGGGAAGGGAAAGGAAGACCTAC
CCACGATGTTTTGTCGTTATTTTAAAATTCATAGTTCCGGTAGTTTACGCCAACTGGTTGGTT
AGACGACGCGAAAAAACCAGCCACGCGAGCGAAATTCAACGCAAGCAAATCCCCCCCTCT
CCCCTTTCTAATACATCGAATCTGTCCATCCGCGTGGATTAGACGGATATAGGCGTTATTCT
CGGCGCGTGTTAATTTCGAGGATGGAGGAAAGAAAAGGAGGGGGGGGGGGGACAAAAA
GGTACAAAACAAGCGCGTTTCCCTCTCGTAAGGCAAAGAGACAGGCCATAAATAATCCGC
TTTACAGCGGCGGTGAGTCGTGCATCCGCGGAGGTTTGCCGTTGAAAAGTGCAGCTTGCAT
CGATGCGCCCTGTTATTCCGTTGCAATAAATACACTCTATCTCTCCCCGCGCCCTGCGGGGA
GAAATTAAATTTCGATCACAGAGCGCTCGATCGTGATCTAGGAGGGAAAGGGAGGAGAGG
GGAGGGATAAAAATTTCAACTCCGGTGAATTTTTTACGCCGGTGATTTCGCCGCCTCGAAG
CGAAAATCGCGTCGATTTCCCTCGCCCCTGCGTCAAGGGAGGAGAGAATATAATCATCCCC
CCCATTGTCGTTCGATCCGTCATCCATCGTTCGAATCTCGCGGCCGCGTATCCGCGTCGGCA
ACGAAGGGACGACGTTTATCGATCGCGCGATGCCGAGAGATCGCGTAACCCATACATATC
AAAAAGTGTGATACGAGCACAAGTTTTTTGTACAGTATATATGTATACACGCGTGTTTTTTA
CCATTAAGGTGATTATGGTGTTAATTATCGTTGTTGCGAAATTCGAAACACTTCTACGCAGT
TTTTAACGAGGTTATTTTGCACGAGGTAATGCAACTTTTTTAAAAAAAAAAAAAAAAAAGGA
ACGTGAAAAATATTTAAAATAGACTACCGTGTAATACGTTGCGCGTTATAAAGGATCCGTA
AAGTAATACGTTTGGAACATGATTGTCGCGTCAATTTGTATTTTCTACATATGTTTTTATCTTT
AATAAGACGGTTTTTCCAAAATAAATATAGCGTATTGATTTTAGAAAAATGCACGCACGCG
CACATACACACAAACACACATATATATATAGCGCGTTAAAAATTCGTATTACACGGAAATA
CATCGTACAAATTAATTAATATTTAAGAAATTTTTGAATGAAAGTTATTATGAAAGTACATA
TATATATATATACATATATAAATAAACATAGCAAATGTGGTGAAATAATTTATGCAATGAA
TCGCATGACGAAACAAAGGTTTTCACGAAGCTTGTTATCGCTTGTAATACAACCATTGATA
AATTCCCACTTGTTAATCGCCGTTGTCAAAGCAAATGAATTTACAACAGGTATACGCGACG
TTTGACGACTCGACTCTTACGCGTCGCTCAACCGATCGAACATTCGCTTAATCTTCGACCGA
CCGAGTAAACACTCTCCCACTCCTTCCCCTTTGAAAAATTAACAAGATCCGTGCCTTACGCG
AGTCGAGATAACGATCGATCGAATCGAAGTACGAAAAGAAAATATATATATATTTATCGA
GAGAGGAATAATTGCATTTACGATTTCCAAACTCCTTCTGTATTTTTATAACCTCGAAACGT
GCGCGTACGATAGAGATGGAGCCACCGTCGCCGCGACAAGAAGAGAAAGAGAGAGATAT
ATAAAGAAGAAGGAGAAAAAATTACCACAGTTGTTTCCTCGCTCGCTCCTCTCGTTTCAAA
GCTAAAATTCCCGCGGGGAAACGCAGTGGTTGCTTCGGCCTGTATCCTTGCGTCGAGGCGA
GCAGTGAATTATACCTTATTACACGGCGGAACGGCCCGACTTCTGTAGATGTCGCTCGCGC
GAATAAATTGTATCTCTGGCGACGACGGACGCAAGGAAACCCAGGCGAGCGGAAAGATTA
GAAAGGACGCTCTGGAAAAAAAGAAGAAGAAAGAAAATAAGGAGAACTTTACTTTGTA
ACGTAGGACCGGTACAGGACGGCGAATTAATTCGGTGCATTAAAGCACCTCGTCCCTCCCT
CGAGCGGCGTGCTCGTTTCAACGTCGTTCAACGTCGTTCCCCCCCTCTTTTCCATCGCCGTGT
GTTTCTCGCTCGTTCGTCGCTCGTCTTTCGTTCTAAGGCAAACCGCCGCGACGCTATCGCGTT
AGATTTGGTTATGTGAAGCGTTCCTTTACGGGGGAAAGGATGGGCCGAGCGCGGGATGCTC
GTTTGCGGATCAAATTTATCTCTTAATCATCCAAACGGTGTGTTTTATCCCTGCGATTAAAGT
TGTTTAAGCTTTTTTTTTTTTTCTGAATTTTTTCATCTACCACGGAATAGTTCGTTTTTCATTCGG
CGATCGTTTTTGCGTACGATAAAGTTTAATTCAACGCTCCAAGGTAAATAGAATCGTTAATT

CCCGCTGTTCATGATTCATCATTTTCTCGATTTATATCAACCGATCGTGATTAGAAAACGTTT
TCACTATCCTTCCCCGATTAAATTTTTTTCTAATAATTTTGTTTCCCAAGCTCTACGAATCCAC
GATTTCTCGTTCGAAACGAGATTATTATTAATCAGGAAATAATATACATTCTTCGATGCAAT
TATTAAATTTACATTAGAGAATTAATTATTATAAAAATAAGAAAAAGAAACTCGAACTCGC
TGACGATGACACGATTATACATTTTCCTTGGGCGAGAGAGGGAGAAAAAGTTTTTTCAGGA
TTTCGGTTGGGTGTCGTAGCCGGCCAAGCGAAAAGGCGAACATTCGGTATCGGGTTGACAC
GCGACCACCGTTCGTTCGTTCGCTCGCTCGCTCGTTCGCGAGGTTCATTTAAATACGGAAAT
AAAGTAATTTCGCGTGACAGTGGTAATAACTTGCGCGCTCTGATGGCGCGCACGAGAACTA
CTCCACGTCCACGCTTCTCATAGGAATCATCGTTATCGCGACTTGATACACAGATGGAAGG
CCGATTATTACGCCATATTGCGATCGCTGGCGATCTTCGTTTCTTTGACCGCGTTAACGAAG
GAAGGAGAATTCTTCGTATAAGCTAACCGTGTTTTTTCGTTCTTGGATCTTTCGACGACTCGG
TTTCTCTACTTGGTTTTTTCTTTTTTTTTTTTTTTTTTTAATTTAAAAGGAAAACGCTAGGTCTTT
TTTAATTACGTTGAATAAACCGCACCTTTGTTCGTCGATTCGTTGTTCCAGTTCTCTTCCTCCT
TGCTACATCTTTGCATTCAGTTTTCCATTTACTATAATTTTAATTTGAAAGAAAAATACCAGT
ATGTTTTAGCTGCGTTGGATATGGACGCCGCTCGCCCGATGCTTCTTTGTAGATTTTCGTTCT
TCTTTGCTACATCTTTGTAGGTTTTTTTTTTTTTTTTTTTTAATCTTATCTCGATTCGAGGAAAA
AATTTAAAGGGGCAAAAAATCTTTTAATTACAGTTGAATAAACTTCGTCGATGTATTTTTTTT
TCTTTTTTACTTGATTTGTTTCTCAATTTAAACGAAGAAAAAAAATTTTAAACTCAAAATCCT
CCGGTCGAGGCTACTTTTGTCGTTCTTTGTATTCCATTTACTAGCTTTTCCAATTTGATTTGTC
ACGTTTAAAGGAAGAAATGTAAGATCTTCTAATTACGTTGAATAACTTTTTCCTCGATAATT
ATTCTTCTTGTTTAGATCCTCCAGTTGCTCCATAATTAATTCTTTACACTCCTACATTGTATTC
CCTCGAATCTCGTTTTCTCACATTTTAATTTAAAGAAACAATTTCAAACTCTAGATCTTCTTG
CTAGATCCTCTCTGTATTATTAATAATAATTCAAGGGCAAAAATTCTTTTACATTAAATTTTC
TTACATTTTAAATAAAAAAAATTTCAAAAATCTAGATCTTCTAGATCCTCGATTTTTGTATTA
AAAATTCAAAAAATTGAAAATTAGTCTAACTAAATTGTTTTTTGCAACTGGATTCTCCTTTT
ATTTTATTTCATATTTTAATTTAAGAATTAAAGAAAAAGAAGCTTCAAACTCTAGATCTTCTT
GGATCAATTTTATCAGTTTCCCACGTTAATAATAGTTCGAGGGAAAACATGGGAGAAGGTA
GTTGCAATTACGTGGAAATAGGGAAAGCGAACATCAAACGTGTCGGATTCAGAATTTTCAC
AGGGTTTGAACGGGAGTCCCTTTTCTGCCCTCACGCGGCCGCCATGTTTAATCACGATATAA
CGGTGGATGACGTAACCACCGATACGTGCGGATTAAAAGAACCTTCCAAGTTTTTCCATTTT
GAACGGTGTCGGGTTTTAATGTGATTGAATCACCTTATCGCCTAATCCTCTGATTAGGATAT
TTTATCATATTTTATCAGTGTTTCGATGAAATTAATTCTCCCCTCGATTTCGATCGAATTTTTG
TTAAAAGTTTTTTAAAGGAGAAAAAAAATTCAAAAATTTACAATCTTCATTATCAAATTGTAA
ATATTGAATTAATTAATATTAAATTTTATCAATATTAATTTTAAGAAATTGAATATTGATTAA
AGGAAGGAAAGGATGTTTATAAATATTTATCGTTTGAATTTTATTCTGAAAACTGTTTTTCG
ATTCTCGAAATTGAAATATTATTGTTTCGTTCGAAACGAAATGAAATCGTTTCGTTTTCCAA
GTCGAGAAGCGTCGAGGAACATTATTCGTGTAAATTAATTTTCATGATATTTTAATGCTCGT
GTTCATCGATGATATTAACAGGGGAAAGTGGAGAGTTATAAATTGACCAAGTTGAAAAAG
TAATCGGTTTAAAAATCTCGAATAAGATCACTTCGAGTAATTAACTTGTTAAAACGCTCGA
ATAAAGCGTTTATTTCGTAAAGCGAAAAGTTGTTTTGCTATTATTATTATTGTTATTACGTTT
CGAACGATAAATCATCGAAGTAACTTTGTTCTTGTAACTTTAACCTATTAAATTTTCCGTTGT
CACAATTTTGAACGAAGAATGAAGATACTTTGAAGGAGAATTAATTCCTTTTTTCCTTTTCC
GGTTGAAAATACGAGCGAAATCGTATTATTTAAAGCCCTTTCACGATCTCTAATAAATTCTA

GCAATATTGTTGCGCTCGAATATCTCTCTCTCTCTCTCTCTCTCGTTGCGCGTGGAGAAAACG
TTACGAGACGCGCCAATCAGAGACGTCCAATCACGCTTTAATTGTTCTTCCTTCGTCCTCGA
GCCAATCAACTCTTAAAACCAGCTCGCGACTAAAAGTTCTCGATGCTCTGCGAGATACCCC
CCACTACTACGTGTATAATATCGTGAATACACGTGGCTGGTTGGTCCTCTCCAAGGCGGTGG
AGGGGGGATCGTTAGACCGGCGTTTGTTCCAAAACCCATCCGAAAACCTCGTCCAATCGTA
TCCTCACAGTTGCCGTGAGAAAATCTTGAAGTCTTTTCGTCATCGCGTAACGCGATACGCTT
TCGTAAAATAATTCACCGAGGATGTTTCGAGAAGCGTTGCGAAAAGTCAGTCGAATCTGGA
ACCCGCTCGCGCGTTGTTACAATTTTATCGCTAGTGAATTATCGTAAAGTGAAAATTTCTCC
AATTATTCGAAAATTATAATTACTTATTTTTCGTTCCAATCGTCGAAGGATGAGGATAAATT
TCGTTCCTTGGATTTTCGTCTCGGTCGTTCCATCTTTCACTTGCTTCGCGCTTTCTCCTTTTCCT
TTCCTTTTTTGTCTTTCAATCCAAAGCGCAATCGGCCACCTTTCTCTCTCTCTCTCTCTCTCTTT
CTCCTTCAATCTAGCCCACGATAAAAGCCCCACTTTTGATCTCTCGAGGGTTGCTTGCCCCC
CTTTTCTCCATGCTAGTTACCCCTTTCTCCATACTCCCTCCTCCTTCCTCCACCACCCCCTTTC
TCCACCGTTTCTCGACCGTTCCACTTTCGCCTCTTTCAACTCTACGTGTCTTTTCTTTTTTTTTT
TTCTTTTTCTTTTTTCTTTTTCCCTTCCGCCACGGTGCCCCCTTTCCCCCTTTCCGACATCGCGA
TGTCCCGATTCCGCCTTTTACACCTTTCTTCGACGAATATTCTCCTCTCCCCCTCCTTCCATAT
CGAAAAATTGGCCAGTTAAGAAGACTTTCTCAAACAAAAAATCGAATCGATTACGAACGT
GACGAAGATAGATATATTAGATTTGGAAGAGGATCGTTCGAAAGTTATTTTACGAGAAGAA
ACAAAGAGAAAAGAAAGGAGGGATGTTTAAAATTAGGAGTGTAATTCGGTTTCGACCTTG
GAATATAAACTGGATGGACGTGTCTCTCTCTCTCAGTTTATCCCTTTCTATTTCTTTTTTTTTTC
TTTTAATCGAAACCCTGTTACAACATCTGAAGCTGAAATTGAACTTAAATTGGAACATTAAT
AGATGGAATTGTAGAACGGAGACGCGAAACTCGGTCAATCCGCTCCTCGATCAATTATCGT
TTTTCACCGAGCTACGAGGATTATGCGGACCGATGCAGAACTTTTCTTTTTTTTTTTTTAATT
ATGAAAAATCAATAAAAAATCCCTAATTACGATAATTAAAAAGTTGAGAGAGATCCTCTAT
TCCCTTTTTCTCGTAAAAAAAAAAAAAAAAAAAAAAAAAAGGTATATCATCGTCAAAATTTA
TATGTACAATTGTAATAAAATTAATCGTGAGCAACCGAATCGATGATCGAGATAATATTCG
TATTGGAAATTTCGCGGAGAGTTTAATTTATTTATTTATTTATTTATCAACAAGAGGAGGGG
TCCGAGACGGAGATAAAAAAGCTCGTTGGATAAAGGGCGTAAGAGGAAAATAAAGTTTCG
CGGCCATTATCGTCGCCGTCTATATTTAATCGGGTCGCTGCGGGTACAATAAAATTGGCCCG
ATCGATTTTCCATTAATCGGCCATTAGTATGCAATAGCTAGTTTTCCCGTAGTTGTACGAGA
AATGACTTTGGTCTCGTGAGATAATAATAAGGCGGCAATAGTCCCGCGAGGACGTTTCACT
CTATAAACCGTGTCTGTGTATCCACTGACCTTTGAGAGAGAGTGTTCTTTATCCGTCGAATA
ATGGGCCGTATAATCCGACGTATTTGCCTTATTACCTCGAAATTGCGAAAACGCCTTTGCAC
GCTCGTTCCTCAATTACCCCCTTCCTTCTTTCGCCTCGCTGCCTACCTTCCCACCATTCTAAA
AAAAAAACAAACAAACGAACTAGAATTAAATTCTTGAAAATAATTCGTATTCGAGGGACG
CTGTAATAAATTTTATTCATGTGATTTGTTAATAAATAGATTTTAGATTTTTTCGATGTTTTGT
GATTGTTTTGCTTTTTTAATTCTTGCAAAGTATTGAATAATCGTTTGGGAAATAATTTCATTTC
TTCACACAAGTGTCGCTAGTCGAACGTTCATCTAGCCAACTTCGTTCTAAACCATGGAAAGT
AATTTTGTTTCTTCACGAGTATTGCTAGTTTATCTAACCAACTTCGTTCTAAACGAAAAATAA
TTTCGATTCTTCACACGAGTGTCGCTAGTCGAACGTTTATCCGGCCAACTTCGTTCTAAATCA
TGATATTCGGTAAGTAATTTTGTTTCTTCACACGAGTGTCGCTAGTCGAACGTTCATCCAGCC
AACTTCGTTCTAAACCATGGAAAATAATTTCGTTTCTTCACGAATGTTGCTACTTTATTCAAC
CAACTTCGTTCTAAATTGTGGTATTCGAAAAGTGATTTTGTTTCTTCACACGAGTGTCGCTAG

TCGAACATTCATCCAGCCAACTTCGTTCTAAACCGTTGTGTTGTTATCGTATATTTTGAGCGT
TTCATGGTTTATTTATAAGAAAAATTCGTTCGATTCTGTGCGGTTGCGTTTTTCGCGCGTGAT
AAATATGCAAGAGGAGTATTTTCGGCATATTTTACTTTTTTATTACCAAAAAAATAAGAATA
TTGTTCAGATAGGAAAAAAGTTACGTGAATTCTATGGAGAAGATATATTAACGGTACGCCA
GTGTCAAAAACTCGTGTTCAAAATTTCGTTCCATCGTTAAAAATGCGCCACGAACAACGTC
TCGAAAAATTGCTAATTTGACTGTTCATAATCGCGTGAAACGAGACTTGGTGTTTCATTGTC
GTGAAAAAAAAAATTATATTTCAAACAAGTTAATCGTTGCACGAGGTGCATCGTACGTGAG
ATAAAGTGAATAATTCGCATGGAAAGATTGAAATTAATCAATTTCACACACGAGGAAGAA
AATATTTAATACGTCGAGGATAGCGTGCCAGGAAACTCGAGAAAATTGAAACATCCAAAA
CTCGCCTAAATGCCCGACCTATACCCGTGTTGACATTTACTACTACCCGCTCCTCATTGTCA
GGGATAACTGTTCTCACGTTTTTGGCAATTTTCCAGAGCTCGTTCGAGCTCGTTCCAACTCGC
TCTTCGCAAATCATCCCGTGGCCAACGTGTTAAATAGTTATGCACAGACAAATTGCTCGGC
ACAATACAGCTCCTCGTTGTCCATGGAGAAATGATAACCGGATTGAATCGATCCGATTATT
CCTTTCTCTCTCTCTCTCTCTTTTCCTGTTGCTTTCTTTTACGATTCGGAATATAAAATATTGTT
TCTTTCTCAAAGAAATGTTTCGACTCTTGCGAAAAAAGAAAAACATAACAAAATAACGGG
AAGAAGCGAGTTTTATATATAAAATATTTACGAGAACGATGACGCAAGTTTTTAATTCGTC
GTCGAATTCCTCGTGTGTTTGTGTCTGTGTGTGTGTGTAATATTATGAAAAACAGCGGAAGC
CTCGGTATAACGCGTCACGTGGAGGGAGGGAGAGAAGGGAATTTACATTTTCTACGCGTGA
AGCTGCGCCCAACTTTGTACATGAATAATTGAAACGTGTCATGAATCAACTGATTTCAATTT
CAAAAACATCCAACCCTTACGCGCACGCGCAACTCCACGTTGAACGTTTAACATGCAACGC
GTATGAAGCGACGGGGCAAAAAGATGGATTAAAAAAGGCAAGGAATAAAAAGAAGAAG
GAAGAAAAATGGAAAGTCTGATTTAGTTGAGAAGGATGGAGGAAAATTTAAAAAAAATTA
AGACGAAAGATTGGAATTACATCTTATGAATTACAAAATTCCTTTTGATCCGAAATATATTT
CAACGGTATATAAATTTTTACTTTGAACGAGATCTGATCTTCGCCGCGTCTACACTTAATTA
GAAACCGCGATTAATTTCAAGTTACCGACCGAAGAAAATCTATTTATCCGACCGGGTAAAA
CGAAAAACGAAAAACGTGAATACGTGAATGTGCGAATAGAAAGAATCTTGGCGAGTTTAT
TCGAGTGAGAATTTATTCCTCGTGATAGTTTAACGATTGCGTAATAATATTTCGACCGTAGT
AATAATACTTGGCAAGAAAAATCGTGAGGCGAGAGAATTGATAAAATCTTAGTCATGCGA
CTCGACATTTGTTCGATCGGTCAAATTTTGCATTCGTCGGCAAAAAAGTCACGAGCTCTCCA
ACTTCAATTTCCACTGGTCGAGAGCGTTTCAAACAGCCAACTAGTTTCGCAACATTTGTTCC
TCAACTTCGATTCGATTCGATTCGAAAGTATCGTTTCACAAGGGTGAGGTTTCGACGAGGA
GAGAAAACAATCGCGAGCAATAACGCGGAAAATATTTTTTTTCCCCCCCCCTCGTAAACT
TCGGACGATTTCACTCGTGAAATCGACTCTCGATCTTCTCTCTTTTTCGCTTTCCTTTTATTTTT
TTTACTTTGAAAAGAGTTTACGCAAGTCGAGTTGTCATCTCGCTCAACGAGAGAGAGAGAG
AAAGAGAATCCGTTTCACTTCTTCTCGTATTGGAAAAGCGCGGTGGCGCACCCGATTCGCG
AAGCTGGGAAACAGAGGAGTAGGAAGAGGATCCTTTTTGAAACGAATCAAGGACACCACT
TTACGCGAGCACAATCTTCAACCGAATCTTCAAGTCTCTTGCCTTTTTCCTCGAGATTTAAAT
GATAGTCGAGGAAGAAAGAATTGTTTCGTGAAAACGTCGTTTGCTTCCCCATCGAGAGACG
TTTCGTACCTTCAACGGAAGTTAAAAACTTCGAATAGACGTATCTTGATTCCGAATGTTGCC
GCTTCAAGCTTGTTCTTGGCATCGAAGCACGAGGATGTTTATGGAAATGTCGTATCTTTGCA
TTTAACATAAGATACGAAAGTTGAAAAATGGAGGATGGAAATACGTTTCTTTTTACGAGTT
CTCTTGACGAACAAGCTTCCATTCCACATATATATATATATATATTAATCCCGATATTTCGCC
GTTATGATCGATTATTTTATTCGATAAATGGATCATTTTATTATTAATTCGTTCTGAGAAAGT

TAGCATCTAATAAATCATTCTTTATCCAAAATCGATCGTTTTAAGCCTCGACGGTGAATTGA
ATGCGAATAATAGAGTCTCGAAGAAAATTATCCTAACGCAAAGAGAGAGAGATAGAGTTA
TCGAGTGTCGAGTTAAATCGATCGGTTTGCGCGTCATGACGTTGACCAATAATTTTTGAACG
GGATAACAAAATTCCATCCGTCGGTGAAATAATTTCATCGCGGTACGGAACGGTGGTACAT
TTACGGGAATTTTAATGGTCAATTGATCCTCAAAATTGTCAAAGGAGGGCGTGCAAACTCG
TGTAATCACGTCGATAAATTTC

121. >MSTRG.2343.1 Gene=MSTRG.2343
AGTTGGAAAGCAGCGGCCGCCGGGGATCGCTGTCGTAGCTGGGCCCGCAAAGATTACGCA
TCACGGCCGGGTAGGACGCCATATTTTCTCCTCCCTTTTTGCCCGGTTAACCGTGTTGCGCAC
GTTAATAGGGGAAAAAGTTAAAATTCCCTATCGATGGACGGGGATGAAATTTCCATCCCGC
GCAAAGGAGAAAGGAATTTTTGAAGAAGGAACGATAAAAAGAATTCTTTCGACGTGATTT
GCGCGCTTCCGGGAGAGGGATGGATGTTTCCTCGCGGAAATGACGGAAAACGTGGCGGAA
AGGAAAGAACAAGGATAAGAATCGAGG

122. >MSTRG.2366.1 Gene=MSTRG.2366
CTCTTCGTGAAATCATTCTTTTTCATCTGATAAAATAAGATGTAAACGTGAAACGGTAAAAC
AAAAGTTTCAAATTTTATATTTTCCCTCGATACGTAGAAATTTAATAGATATCGGATTTTCA
ATGGTACAAACATCAAAAATCCATTTAACTTTGTACTTGACGAAAGTTTCTTCCACATTTGG
CAGCGGTCATCGATTTCTAACGACTTGGTAAATCGTTTTAAAAGTTATTTAGCTATGTGGAA
GAGTCAGAAAAACGTGAGATTCGCTTTGAATTTCTGTACGCTAACAAAGGAGAGACCAGA
GAAACGTTAACGTAGACGATGTTAATCTATCATCAATTCGAAATGGACTACGCCCATTTTGT
CATGGCGGCTATGCTTTATGTTTCTCGCCCACGCCTTGCACTCTACGTTTATTAAAATTCCAG
TGCGAGGACGGACGAAATGGATAGCTACCAAAGGACTGAGATAACCCTCTGAATTTTCAA
AAGGATAAAAATAACCAGGAAATCCTCTTCGTGGAATGTATTCAATTGGTCCTATATTTTCT
TGATCTGCTGGATTCTCTCCCTCGCAAGATACCCAAATTGTGTCAAGCTGGTAAGTATTTTTT
AATCCAGTGATGTGCTCTTTGAGATCGATCGGCATGTTTTGAGGTAATGAATTCGTATCGTT
ATAAAATTCTGGTTTCCAACCATATATCTTGTTAAGTTTAAGAAATATACATGGAGCGGATT
TATGGTAATTATATTTATTCTCTTTAGTACAAGGATACCAATTTTTAACATCAACGTCGCACA
CTTTACCTGGAGGTGGTGGTTGATTGTAATCACATTTATTAATATTTGCACCGAGACCAGGA
ATCGAACCTGGCGTTATATAATCTTTTAAGAACGATTCCAAAGAATCGATCCAATATTTAAA
ATTCTCGCTATCGGTTCCTCTGTACCAAATTAACGTACTCTCCACATTTTCTAATGGCGGTTG
TGGTCTAAATCCTAATCCAGGATTTGTCCCAATTATCGAACGTTCTAGTTGCCATCTTGGTAT
CCTTGGATCTAACGTTTGAAAAAAACCCCAAAAACAAATTGCAACCAGTGCGGCTAGCAC
CCCGTAAAATATCAAATAGAACAATCCTATTTTACCCCAACTACTAGCTGTTCTGCCTAAAA
AGGCACCAGTTTCACGATTGTAAATAAAAGTCTTTAAAGACTGGCTGGTTGACATACTCGG
CGGTTTAAGATAAGGATTTGCCATCTCCGGAGTGACCATTTTCGAATCCTTGCTGACCATTG
TGTTCCCAAGTGAAAGACTTTATATAAGATTTACGCACTAGTCACGTTTTCAGATTTTCGAA
ATTATAATTATGGCAAATGCACGTAATATAATCCAAAATGAAACAACACGATCGACAACA
CCTGCACCGAACTGTGAGTTCTTCG

123. >MSTRG.2369.1 Gene=MSTRG.2369

CACATAAATAATTTTATTTCTGATCACAACTACAATTTCTAAAGAAAAACAAACTCGCGAG
GCACGACTCGATTTATACCGTTCATAAATTTCCGGTAGATGCTATTAATAATATACGTATAT
TAACGTTGTGTACCTTTGTTGCCCACGCACATTCACATGAAAAAACCTATACAATTAAGATA
TACAAAATACAATAAAAAAATTAATTTCAAATAATTTAGTATCTAGTGACATTACACGCAA
TCGCTCCTCTTAAATATTCACAAAATTTGTTCCGTTAGCACTGATTATCAATACTGTGAATCT
TACTTCTGTGAAATAATTTAATTCCGTCCTAAGTATTCGTTTTACGAGGTTAACAACTTAACA
AATTTTGAACTTAATCTTTCACTTGACTATATCTTACGTATAAAAGTCTCCTTTTTTTCCCCTC
CTTCAAGGTATCGTAGTATATATATATATATATAAAGAAGATTTTAGCTTCTACGATGATTG
TTCATTTTAGATTTATTTGGCAACCGTAGAACTTCCGCATAAGACATTTCTAAACGTTTTAGC
CGGGCTACTTCGACCGACGCTGTGCTCGGTTTATAGTCTCTGTACTCGAAACTGTTGTTGTGT
TGACTACTGTTGTTGCCTGCGTTATTCGAAGATTTTGCGGCCGAATTAGGAGGCGAGTCTTT
CGTTTTGTTCGGATAAGCGTACCCGTTGCACGTGCCGCCATTTTGATCGGCGATCGTAAAAG
GTCTGAATTTCTTCTTTCCTCCGCTGTTCGATTTCGATGGAACGAACTTGGGATTTGTCGTTG
GAATCACATCGGGCGTCGTTGGTCTAATATCGTTATACGTTAACTTTTTACTAGCGGAAGAA
TGTTTTTTCGATTTCTTTTTACTGTTGAGCATTAAATAATCCGACCGATTCACGTTTCGAATTA
TATTCTTCCCGTTCTTAGCAAGTGTCACTTCACCGTTTTGCTGTTCGTTTACCCCGTTCGATGA
GATTTGTACTATATACGGTGGTTCGGATACGGCGGATGAAAAAATAGAAGAGGAAACGCT
TTGATTATCGTCGGTGGTTTCGCTAGGAACATCTTCTTGTAGCACGGATTGTTGCTGAGAATT
GTTCGTACTCGTGGAATCGTCCAATAAAGGTAATTTCTCGCCTTCGTCCTTAGCTTCCGCCGC
TCGTTTCAAGACTCGGAATATTTCACTGAACAATTGTCTGTACTCCGGCGTTTCCCGGAATC
TACTCTCGAACGGGCTGTTGTCGTCGTAAATGCTGAACTTGCAATCCTCGCCGTCCGCGAAG
GAGAAAAGAAACGAGCCGGGCCTTCCTTCGGTTTGCGTCGCTTTGTTACTCGTCTCGTCCAA
AAAGCCGGAAGACGACGTCTCGGCCTCGGAAAAATCCGTGGGCGTGGCGGAGAACACTTT
TTTACCGCACATCTCTGTTTTGGCACGTGTCGCGTTTCCAACGGCCGATTTCGCCGCGTTCGG
CTCTTGTTGCGTCTCCGTCTCCAACGTCGGACTCTGAAAGCTGTTAAACTCGCCCGACATCT
CTAGTTCTTCTTGCAACGACATAGGCGTAGGCACGCTATCTTTCCGCCGGAGCGCGGATCGT
CTTTGAACTTCTAACAAGGCTTGATATTTCTCCACTAGCACTCTATAAGGATTCTTCATGTCG
TTCGACACTTCGTTTTGATCGTTCGATTCCTGAGTGAGAGACTCAGTGTCACGTTGACTATCG
TTCATTAGCGATTCAGCCATGCTAATATCATCGTATTCCTCCTGATTCAACATTACAGAGAG
TTCGTCGTGCGTCTTAGAGTCCAATCCGCGTAAACATCGCCCGCATAATTGACCCCGCCTTA
CTTCTTCCAGAGTATTTATTTCCTCTTGAAGATTTTTCACGTCTTGTGCTTTATTACGCCAAAC
GTTCAACTGTTCTTCCAACGCGTTATTTTCCTGCAACAATGTTGCCAATTGTTGCCGAAGCTC
GGTAACTTTTTGCTGCTCCCTCGCCCTCTGACTTCGCGCCTCTTGCAACTGTCTCAACAATTC
GGTAACTTCTTCGTCGTTCGTCGGAATCGTCGGTAATTGTCGCACGCTCGAGTCCGCATTTG
CCTCTTGGAAAGATTTCTGACCAGACGGAGCAGCGTTCTGTTGTGTGCTGCTACCCTGCGTG
ACCGATACTTTCCAATGAATGGTGTGTGTTTGTGTGATATTGTTAGCCGATTGACTCGTAGAT
TGTTGCCGGAGCAGACTTTCATGTTGCTCGTTTATCTCGTCGATTTTCTTCTGAAGTTCCTCGC
ACCTCGCCTCTAGGCTCTCGATCGTCAAACATTGACTCTTGATCAATTTTTTGTCGCTGGTGT
TCTCGATTACTAGACGATGATTCGCGCGTTCCAAATCTTGAATACTGACTTCCAGTTGCTCG
TAAATTTTCAATCGCGAGTCGTTCACTTCTCTAAGGGCAGCGGTTTGTTTTTTCATATACTCT
ATCTCTTGTGCTTGTTCCTCGACGGTCGATTGGTGCGCTTTGATAATATTTTCTAATTCCTTGT
TCCGTTCCAAAAGCGTTTTTCCAAGTTCTGCCGCTAATTGTAAATCCTGAAGCATATTGTGA
TCGGCCTCCTCGTCCGCGAGGCAGCGAACGTTGCCGATGGAAGAAGACAGCAGACTCATG

TTTCCTTGTTAGAGACGTCGTCGTCAAGTTTCACGAGCGGCTCACGGCTCACGCCGTAACGA
AATCGATCGGCGCCGGAATCGCGGAAAAGCCGCCTAGTCCGTACGCGTACGCGATTGGTC
GAAGCTGCGGTTGAAGCTTGCGTTCGAACCAATCGTTACAGAGTAGTCGGTTGCTCGGTAC
ACCGAGCGAGCCTCTACGAACGCACCTGTCGGCCAAGCATCCGGCCAACTCCTCGAAAAC
GCTTCACCGCCGATCACTTTCACTCGACGTAGAAAGGACGATCGCGATATCGTTCGAGCAC
CACCTGCCAACGGTGGAGACTTTCCGATTCTCGATTCGCCGTTTTCGCCGACAGGGAATCCC
GTTTTCCAGCGGCTGGACCAGGCGGCAGTTACAATTCACGTTAAAATCGCCGCGTTTCTCGA
TCGCATCTTGTTAATCTTGTTGCGATCTATAAACCCATACGAGCCCCAACTACACGCACTGC
CGAAATAAATCGAAAATACACACGTCTGCTGTCACTGGCACCGTTTTTTTCGGCTACCTCGA
ACTGGACGAGACGGAACACGAGTAAAACTTACTGCCCGGCACCGTCGCTACGTCGCGAAA
GACGACGACGCATGACGCGTGCAGGGACACCGAGCACCACAGAGCCACGATCAATAAGC
GTTGCCACGGCGTCTGCGCCGACAATGCGATGCTTCGACGGACGAGAGGAAAGAGACAGG
GATTACGCGCATGTGTTAATGTGTCTGTGTGCTCGCGCTATCGACGCGACCAGTATCGACAA
TTTATAGTGCTCTTCGTTTACACCGACACACGCTTCTACCTCCATTGCTCCGTTTGCATTAAT
TGCTTCTTCGATTTTAACATTTATTTCCTCTCTCAATCGAA

124. >MSTRG.2415.1 Gene=MSTRG.2415
TTTTAATAAACTTTATAGTAATTATTGAACTTATTAGATTAATTATTCGACCTTGAACATTAT
CAATTCGATTATCAGCAAATTTAATTTCTGGACATTTAATTTTAACATTATTAGGAATTAATA
AATTTACATATCGAATTAATAAATAAATTCCAGCAGTAACTAAAGTTGATGAATGAACTAA
AGATGAAACAGGAGTTGGAGCTATTATTGCTATTGGTAATCAAGTTGAAAAAGGAATTTGT
GCTCTTTTAGTAAAAGCTATTAATAAAACATAAATCATTATAAATTCATTTATTTTATAAAA
ACTTAAATTTCATCTTCCATAATATGTTATTAATCCTATAATTAATAATAAACCAATATCTCC
TAATCGATTTAATAAAATAGTAACTATACCTGAAGTAAATGATTTATTTTTATATAATAAA
TTACAAGACAATAAGAAATTAATCCTAAACCATCTCAACCTAAAATAATTGATAATATATT
CGGTCTTAAAATTAACATATATATAGAAATTAAAAATAAAATTATTAAATATAAAAAACGA
T

125. >MSTRG.2429.1 Gene=MSTRG.2429
CGCTTCGATGACAAGGCCGCCAGTTACTGTTCGTGCACGAATGCGACGAAATACCCGAACG
TCTTGGCGGGTTTTCGCTCGGAGCTGTGTTCACGAGACCAGCTCAGCAACGAGGAAACGAA
GGTGACGTTTCTACGAGAAGGAAACAGTGAACGTTCAACAGTATACGAGAAAAATAAGGA
TCAGCATCGAAGTGATACTTGCACATAGGAGACACCAACGCACGGAGAACCTGTCGAGCA
CCTGAAGTTGCATTTTAGTCCAACTTTGGCCGCGCTGATAGGCGGACGATTCGCAGAAGTTC
GACGTACCACATTCTGTATTAAGGTTTCCAGAAAGTCTTACAATGGAGCCTGGCAAAGAGT
GTATTCAGTATCGTTCTCTTTATGAATCGAACAAAAGGCGAACATGGCAGTCGCAATAGTC
TGTAATATACTCAAAGCAATGGAAATACTGTAATTAATACAGACCAAGTATATTTAGCCAA
ATTGCAGAAATGAAATTCTATCGGATAATTATCTTCGATTCTTCGAAAAATTTAATCAGCAA
AATTGAAAG

126. >MSTRG.2459.2 Gene=MSTRG.2459
CGTTATAGTACGCAAATAGAGGCAAGTGGAATGGTGTGAAAGAATTTAGATCGTGTGAACT
TTGTATGAATAATAGTGTATGAACAATATTTTATGAATCATTTATAGTGAGATAAAAGTCAA

TTTATTATTATGCATACACAATTGCATTCGATTTAATTCGTCCTATATTTTTTATAATAAACT
TTAACCCCTATAATTGAAGTTTTGGTTTCTTTTAAAATAGAAGTGTACTAAACGAGAAGGAC
ATCTGTTTGTGTGCGTGTCTTCACTTATTCTCCCTACTTGTCGGATTCATTCTAAAGTGCAGTT
GATCTTTGTTCGTTGCACGTATTAAATCTTATCGGAAACAAAATCGAAACGTGTACGTCGAG
CGAAACGACGCTCGGCTAGGAAAGTGCAAAGGGATGCTATCAAAATGGGTTTGCATAATTT
TTAATCTAATAGTTTCTGTGATCGCGGCGATGAAAGATGAAAATATTAGAGCTTTCATAAA
AATAACTGAATTAGAATACGAGGATATTTGTTTAAGTACATCTGAGGCGGAATGGGCATTC
ATTAATGCACCTTCAAATGAAACATTATCATTATGGGAAGAAAATTTAATTTCTTATGCTAT
TTTCAAGAATTTTCAAAAAGATCAAATAGCTAATATTTCAAAAACTGATATTAAGGATAAA
TCTTTACAATATAAATATGATATTACTGAAAAAATAGGTGATGCTTTATTAGATAGTGAAGA
TTTTAAAATACTTGTTCACTTTTC

127. >MSTRG.2473.5 Gene=MSTRG.2473
CTAATATATCTTTGTTGCATAAAACTTGTATAAAAAAATTCACGACTAGAAGTTAAACCGTT
ATTTCAAAATTGTGCGCAAGATGACGTAATATATCTTGATTGGACCAACGTTATAATGTGAA
CGGTTTCTATTCAGCACAGTTTACATTTGTTTGAGAAAGTGAGTGGATATAGTTATATCAGTT
AGTTTTAAATAGATATATAAGGTAATAGGTATATAATGTAATTGTATATTTCTTCAATTGGA
CATCTTTTACTTGACTACAATCATTTATTGAAATTAACGATATCTATTATAGTGTGGAACACT
TTATCTTGCTTAAGAAACCTATAAAAGAACTCAAGAGTGTTATCTGAGTAAGTAAAGCAAA
AACCTATAAAGTAGTAAATGTATTTTTATGGAAGGCTAAAGTATGGATCAATTTTGTGGTAC
TGAATTTTGGAACTATAATTTAATATGGAATACAGATGACCCAGAGATTACAGAATGTTTTC
AGAAAACTGTGTTAGTATGGGTACCATGTGCATTTTTATGGTTATTCTCTGGAATAGAAATT
TATTATTTTTAAACAGCAAAAATAAAAATATTCCATATACATGGTTATTTATTTCTAAACA
AATACTCATAATAACTCTGATTTTACTTAATATTGTTGATTTAGGAATAGCTATACATAAAA
GTACTTATGAAAAAGTTTATAATGTTGATTATTGTACACCA

128. >MSTRG.2510.1 Gene=MSTRG.2510
AAGACGCTTATTCCTCTCCGTGTCTATGCGCGTATCATAAAGCATGATTCGCGAAAGAACA
CTTGGAACGAAACGCTCGGTTTCTCCTCATCTTCGTTTCTTCATCGCCAACGGAACATCTTCA
AATATATCCATATGATATAAACGATACACACACGCATACACACGGAGATCTCCGCTTTTCT
CCTCCCTTACAAACAAGCTCGCTCGCTCGCTCCAGCGCGGTTGTACGCGGCTCCTAAGTCGT
GATCCAACGCGACCACGGATCGTGGAGCGGTGAAAGAAGATCGTACGAGTCGTCGTCCGC
AGGGAGGAAGAGGACAAAAGAGACGAGGGCGCGTCGGGGTGCGTGCAGCCCCTCCCGGC
GGGTGAACGAGGCTTCGCCCCTTCGTTTTGGACGCGAGTTGAACGAGGCCAGGCGACCGAT
TGTCGAGCATCCCCATCGTCGTCCTGGCCACCTGTCGCAGCATCGGTCGCAGCCGGGCCGC
GTGCAGCATACGCGACGACGACGACGATGACGACGACACTCGTTGTTCCCATGACGTGTTC
CTCCGCATCTTCCGTCTTCTCGTGGCGATCGATTCCCTCGTGGCGAACGAGGCTCCTCCTCG
GGATGGCACTCCCTTCTCGACTCGACGAACGGCACGCTGGCGACAATAACCGGGGGTGAG
AGACGAGCGATGGTTTGGATCTTTCGGCGGTGGCGGCGACAGCACTGGGACCTTAACACCC
ATCCTTGCCACCTTGATTGGACGAGTGTGAGGCTCACGATTGCGGTTGCGGTGTGCATGGCT
CTCGCTTCTCTGCCAAAGCCGGCGGCTTTATCACAGGCTTCTCTTCGGTCACTGGCGTGGGT
GGGGCGTCCTTTCTTGGCGGCATCCTTTCGTTTATAGCGTCCAGACCCTTCGCCTCGGCGAA
CGAAGTGATTTTGTGGTTCGGCGAGAAACCTTGGAGAGCGTTTTTAGCGACGTTTTGCCAC

CCGATAACGCACCAAGAGGCAAAGCTCCAGTGGGCGTGAGGCCCTTCAATTGTAACACAC
TCTCGCTTTCCTCTCTCAGGACGGAGAAGACGCGCGCCATTTTGCCAATGGCTCTGATCTTG
TTTCTGATAACCTCCTTGCGTAGATTGGCTGCTGCACCTTCTTCGAGCCCGTCGTCTCCGTCG
CTCATCAGCTCGTCGTCCGAGCATATGTTGAGCACGTTCACCAGCATCTCTGTCACTTTTTCA
CCTACAAACGGCAGGGACCACGTGAACACGTCCATGAAGTTGGGCAACCAATAAGGATGA
GGTGAACAGTTGAATTGCCTGATGTTCATCACGTTGTTCTCGTACTTCAGCACTGCCGCCTTG
TTGTTATAAACGTCGAGGTAGTTGGGCGCACTGAAGATGGTGATTAGCGATGGGAAGCCCG
TCGTCTGAGATTTCCGGTACATGCGATAACCCGCGTCCTGTGCCTCGTGCGCCCTAATGATG
CTCAGCAAGTTGTTGTTCTGCAGGAAATCGCAGCACGCCGCGTAACTGTAAAAGTACGAAC
AACCCCTGACGCTGTTGTGGGAGAAATGTTCGGCGTTCTTCTCGTTCCCAAAGTCCTCCAAC
GGATCCGACCAGAGTAGATCGCACATTGGCCCGTACGCCGGGGGCTCTTTGAACCTGTCCA
ATTTGCGTATGTCTTCTAGATTGTGAATTTCCGGCGAGAGTCCACCGTGCACGCAGAGGAAC
TGTTGGTTCATGAGAGCCGCCAGCGGCAGACAGTCGAACGCGTCCATGCAAGCGTCGTACA
CCCTCTCCGAGTATTTTATTTTACATTCTTGCTTAAATGTGAAGTATTCCGTGAGATGACGGC
ACTCGTGATTGCCTCTGAGGAGGAACAGTGTGGTTGGATGGCACAGCTTAAGTGCCCACAG
GTACAATACGCACTCGATACTGAAATAACCCCTGTCTACATAATCGCCGAGGAAGAGATAC
TTTGTCGTGGAGGGTGATCCGCCCACTTCGAATAACTTCATCAGGTCGTAGAACTGTCCGTG
AATGTCGCCGCACACGGTGACTGGAGCTTCGATATCGATCATAGTCTTCTCGGAGCGGAGG
AGGGCGGCTCCATCGGTGACTATGCGAAGAGCCGCCGCCTCGTCGATCCTACCTTCGAGGA
TAAAGTGTTGCTTGAGGACTTCCGGCCGCGGCCGGTCCGTCCGCGGGTCGAACACCTCGGC
CACTGTAAGTTTGTGCGACGGCGGAAAGAGCACATTTTTGATCACTCGTTCCGTCGTGGAG
AGCTTCTGCGAATTGTTCGAGGTCATGCTGTATCAGGATAGTTGACGGGTGGGCGCCGGCCT
CTCGCTCTCTTTCTCTCCTTGCTCTTCTCTATTTCACTCTTCCTCTTCCCGGTCACACAGGATC
GTCTCTCTCCCACTCTTTCACCGTGTCACTCTGTCACTGTTTTTTTCTTGTTTCTCTAGCCGTAG
TTACTCGCGCGAGGTAGGTTCCTCTCCAACGGGGGGTTGGGTGGAAGGAGAGGGCGGTATT
TTCACCTCGAGAGCCCGGCGCGGCACACACACATACACGCACGGAGTAGGGGAGGGAGG
AGACGGGGGTCGGAGAGGTGACGGAAGTTATCGCACGGTGCGGCACATCGGTGGCTCTGA
AGCGTTGTTCATTGGGCGTTTGCAGAGTGAGAAATTATTCTGCTGTGTTCCTCTCACTTCCCG
TATATTTACACCGAACGACCTACAGTATGCCTCTTCTCGTTTTTCTCACTCCCTCTCTCGCTTG
CTCTCTCGCTCTCTCTCTCTGCCCGACAAAAGATACCACCAACGCCTTTTGCGGGGCGAGTA
ACTGGATCGCTGGACCACGCGAGCTGCACGGCCCGCACCGAGGGGGTCTCGCAACCGCGG
TACCACCACACGAGCAGCACGACAAAACCTCCGGTAAACGACAATAAGACGATGATTATT
AACGACCGCAACGACGTCGACGAAGTAGAGGACAACGACAACGACACAATTGCGGCGTG
AACACAGGGAGAACGATCACGGCGACGACGGCGGCGACGACGACGACGGTGAAGACGAC
GATGATGACCACGACGACGGCGATGGCGACGACACCGTAATGACGAGCACCGCGACACG
AAACCGTGACACCGACACCCACGGTCGAAAACACCACGCCGCTAAATTCTACTACAAGAT
GTCTGTCTGTCTGGCTCTCCG

129. >MSTRG.2529.1 Gene=MSTRG.2529

GGAAGGGTTTACGCCAATCGAAAGGAAAATGCCGATTAGCGGTTGTCCAAGAGGCCACAG
ATTTTTACCACAGTCCGGATGAATATCCACCAGGTGGCACGCGTGTGCGTGCCGGAACTTTT
GCTGCTGGCGATGTATCGTTCGATTTCTCGCTGGAATTCGAGCTAGTCGTTCCATTTTGCACA
GCTGGTCCGTTGTTTTGTGATTCTCCATAACCAATTCCGGTTACCGGGTTGCCATCGGGCTTG

TTGCGCGGCGTTTCGTTGCTGTTGCTGTTGTTCGGTGCTGTATCGCCGAACAAAGCGGAGCC
CAGCTGCGATTGGTTGTGGTTCTTCACGCGGCGGGGGCTAGTCTCTTCGGGCGCCGCGCCGA
AAATGTCGCTGCTTCCGCCACCAGGGGGTTTCAACACCCTTTTCTTCCCGTATCCAACATTG
TCGAGCTCGACGTGTCTGTAGGTCGCGTAAGTGGCCATCGTTCCTCCTTCCTTTCGAAAAAC
TCCCCCGCGTCCACCCTCAGCTGCGTCTTCCTTACGTCGAAGGTGGGTGTAACTCCCGTCTG
CGGTGTCACACAGAGCCAGAAGAAGAGAACACGGTTGTCGTCCTCGCCCTCTCGGTGAGTT
TCGCGAGAGTTTCTCTCGATGTTGCGCAAAAGAGGGACGGAGGGAGGAAAGGAAAGGAA
AAGAAAAGAAAAGGAAAAAAAAAAAAGGAACGAAAGATCAATATATCCAGAGACGACG
AGCCGACACGGTGAGCTTCCAACCAATTCAGAAGCTGCACTCGCAGAATCTGTGCGTCCTT
GACTCGAGAGACGGAAAAAAAGAGACCTCTGCGAGCTCTCCTCTTCCTTTTGCCGTTGTTGT
TATTGTTTTTGTTTTTGTTGTTATTATTATCTCTCTCTCTCTCTTAAAATTGATCTTGAAACCGA
TTCCAAGCCGGATAAGGTCACGAATAATAATAATAATAATAATAATAACAATAATAATAA
CAATAATAATGATAATATCGTTTATCGAGGCAGAGGTAAACACTCGTGCAGCCG

130. >MSTRG.2554.1 Gene=MSTRG.2554
GATGGCGGTTAATACTAAGAAAGTAACATCTATATATCTTCAATGTAGATAGAAACATATG
AATAACTAATAGACGATACAATCCAAGTGATACTTTATTGATATCAAATTCATTATAAATTA
TTACGGATTTTATAATTATTATTATATTATATATGATATAAATTAATAAGTTTAAAAAATGGC
ACAAGAAGTAGAAGAAACTATGAAACGCATTCAATCACATAAAGGAGTAGTTGGAACAAT
TGTAGTTAATGGAGAAGGTATCCCAATCAAATCTACATTGGATAATACTACCACAGTTCAA
TATGCAGGATTAATAAGTCAACTGTCAGATAAAGCACGCTCTGTAGTACGAGATTTGGATC
CAACAAATGATCTTACTTTCTTACGTATTCGTAGTAAGAAACATGAAATTATGGTTGCTCCA
GATAAAGAATTTATACTCATAGTAATACAGAATCCAGTTGATTGATAACCAAAAATTAAAA
TATAAAATATTATATGCTTTGGATGGATGAAAAACACCATAATAAATAATCATAGCTGTTA
TACCTAATAAAAATGGTACAATACAAAAAATAATGAGTATATTAAAATACCAAGAATTTTT
AACTTCATTTGATGAAACATATATCCAAATAACAGTTGCTATTATATTTTCAAGAAAACAA
ATTATATAAAAGAACACATGTTTAAGTAATGTACTACCATCTATTGTATTTAGATATATGAA
CACATGTATTACACCAACTACCATGGAAAATAATATAGAATAAATACGTTCTTTAATTTTTG
GGGAACAATGTGGAGCATGATTAGAATCTTTACAAAATTCTAATATACCATGAGAATCAAC
AATAATCCAAATAGTCATACTTATCCAATGAAAAATACAACCAATTATAGTATATATTGGC
CAAATACTTGCTAGAA

131. >MSTRG.2556.3 Gene=MSTRG.2556
TGCTTGAACTAATATTCCTTATAAAATATAGATGTAAAATATACTTATCGGAAAAGTGTTAG
ACTTAACTTGTTTCAACTTATCCTATTATATTTAAATTATAAAAAATCTTTTTTAAAAAAAGT
ATCATTAAAAATGTCTGCGTGGAGACAAGCGGGATTAAATTACATCAATTATTCTCAAATA
GCTGCTAAACTTGTTAGACAAGCATTAAAATCAGAGTTTCGAGCAGAAGCTTTGAAACGTG
ATGAAACAAATATAAAATTTACTCAATGGAAAGATGGTAAACCTACTAAAACTCATAAATT
ATAATATCACAGATTGATGGAGAAATCATCCTACACTTCTAATATATATGCATATTCTTGCA
TATTGTATATGAAACAAATAAGAGATTTAATAAAATAGTAATTTATGTATAGTAATTCAAC
ATTCTTGTAAGAAGTAAAAATATCCTATAATATTATCTGTTTTTCTTTAATTTAATCCATTTTT
CTCTTAGAAGATCATCTTATAGTAGGACAATGGGCTTTACAATGTACTAAAAAAATATATA
TGTTATAATTGAATATAATTTCTATATAATTTTATAAACTCACCTATAAATTCTGAAACAAG

ATCATTATCACTATCACCACGCATTGTACCTGTTATTTCTTCATCTTCAACCATAGGAAGAA
ATAAATGAATAAATTCGACCGTATATGGTGGAGCTATTGCTTCTAAAACCTAAAATATAAT
CATTATATTATTAAACATAACATACATTCCTTTTAAAAATAATAAATAATTTTATAATACCT
CTGTAACAAAATATCTAATTAAAGATACATCTGTATCTCCACGTTGCCAACATTGTTTAATG
TAACACACGACGGGTA

132. >MSTRG.2580.1 Gene=MSTRG.2580
AATGCAGCCGAGTGAGGAATTCCGGCCCCTCCCGCATATTTATCTGAACGCTGCTCACCGG
TCTGCAGGGCCCCGCTCCGAGCGAAGGGCCTCTTTTATAGTACGGCAACTACTCTTCGTCGC
TCATACCAACGGAGAAACGAAAAAGGGATAGATAGCAGCAACCGCACGAAGTATCGGGC
CACTATGCGTACAGATCTATTCAGGAATGCCCACGGTGTTTTGTAATTGGGCGCATGCGTGG
CATTTTCTCCTGAATATGCAACCGCGATAAGGTAGCCAGTAGAGAGGAAAAAGAATCAGC
AATAAGCAGACTGATGACGGAGAAAAGCATTTCTTCGACCGGACAGCTGTTGCCGAACAA
AGGCCGAAGGATACAAAAGAGGAATTTCTTGGGCCGCACGTGGCTCCCGAGAGAAAGAG
AGAAGGGAAGGGAGAAGAAAAAGAAGGATTCACGGCTAATGCAAGGGGCCGACTATATA
CACGTGACCCGCTGCACCCTTTTAGGCATTCCTCTCCTTCCGTCAATTTCCTCTTTTCTTAATT
TCAATCATCTTTCATTTCTCTTCTGTG

133. >MSTRG.2606.1 Gene=MSTRG.2606
AGGATGTTCGTACCCGCACGATGGTTGCATTACCGGTAATCCTGAAAAGCTGAGGAACTTT
TTTACCGGATCTTTCGAAGGAAATCCTACGTGAAAGAGGAAAATGTCCGAGATATACGAGG
TGAGTCTTTGTGACCGAAGTTCAAAGCTGTTCGTGCGCCACGATTGCCGGATTGTAAATCGA
TTAAAAAATTGCGATGAACGATCGTTATAATCGATTGAAGTTAAAAGAAAAGAAAAAAAA
AAGAAAGAAAGAAAAAAGAAAAAAAGACGAAGCAGAGGGTCTTGTTACATATAATTGCG
AGGATGTAAAGG

134. >MSTRG.2634.3 Gene=MSTRG.2634
AAAGCCAGTAAGTGAGCGTTGTTACGTGAGCATGAAAAAATAAATTATACTTCAAAGTAA
CAAATGTCGATTTGTGATTTATTGTATAATTATAGTGACTGTTTTCGCGTGAAAAAAATATAT
AAAATCGAAAGCAAATAGTAAAAGAAAGAAAAGATAAAAAAATTAACCTTAATTAACAA
GATTGTCATTTGTGTTTCGTGTCGACAGTACTTTCATATGATTCACCGATGAACTCGCAAGTA
TTCGTTCCTGTGGACTGAATCTAAAATCGCTATGCTGCGGTCGGTGTATCGGCGCGCCGAGA
CGAGACGGACGGGACGAGACGAGCGACGGCGGCGACGAGGAGGTGGAGGAAGAGGCGG
TGGAAATCGGTGTGTGCGCTCATGACGGAGACCTCGTGCGTACCAGTCGCGTCGTCGAGCG
GAGCCGAGTCGAGTCGTGTTACGGCCGTAATCGTGGTTATTCCTCGCAACAGTTTGCTCCGT
GACGACGTCAGTATCTGTGCGCGAAGACGAGAAACGATTACCAGTAGAAGAATTTAAAAT
AGTTAAAAATCAAGTGTAACGTCGCGGTGAAACTGAATGCAATTCTTTTCCCTACTACATCC
TAACATTTTTTATCCGAGTACGTATCGAAGTTTTAAACGTGCAACCGAAGCGCTACTCTCGT
TCGACCTCTCTCGCCCTCCGCGAGTTATATATCCGATCTACGTGCGCTAGTCCTCCCTACTCT
CTTTTTCTCGGTGTATATTTTGCTCTCTCTTCTAACTGTCTGTCTTACTCACGCGTTATCCCTCC
TCATTAGTATCTCACTCGTTCTCGCTGGCTCTTCGAGCAGCGATTTCAAGAATGTGACAGTG
CGCGTCCTCGCGGTCGCCTCGCGCGAATAAGCCCGCAGGCCGGCGACGGCGATGGGTGAC
GAGCGACAAGTGTTACGGTGAATAGTGAGAACAGTGAAGAAGTAGGGAGAAAAAGACAG

ACGATGCAAGCGGAAAATGTCACTCGGTGATACGCGTATACGGATGACGGGGAGTGATCG
ACGCGACAATCTCGAATTTGAGGTTAAAGTGTCGTGGTCGTCGTCGTCGTTGTTCTTGTTGTT
GTTGTTATGTGGTGCCGGTGCGCCGGCATTGGCGACGGCACGGGCAAAGGAGTATCAAGA
ACGAAAACGGCACTATCGTATTCTACAGGGCTTGGCAGCCCGAGATTCAAGGGACATTGAT
CAAGCGCCGTGGTACATTGTTTGAGTACAGACTCATCTCCTAAACGAAGGAAGCGAAAAA
AAGAAAGATTCGCAACGATACAAAACAATTTTTCGTAAGGCGATTGTGCCAATGTACGTAT
TATATACGCGCGCGCGTGTGTGTTTGTGCTTATGTGCCGGGTCGTGAGGTTAAGGCACCACA
TCGCCTGTCGATATACAACGTTGAGGAAAGTCAAGAACCGGTGTCGTGTGTGCGCGCGCGA
CCGATTCGTGCTGCTACATCTAACGGATAAGAAGTGTATCGTGATACTAATAGTGCGCGTC
CGTAACCCCGCTGGCGACCGTTGCACCGTGCACATCTACGGACAACCAAAGCTGACGTTCG
CGAACTGTCGAGACAAGTAGACAATCCTCGTGCCTTTTCACTTGTATATTATGCGCTACGTT
TTTTCGTTCTCGAAAAAATCCTCGAGTACTTTTTGTCTCCGACGACAACAACTCCCTTTGGAC
ACCAACTTATCCAACGCGACACATTTTCTTCAATGTTGTCATTTTGTTATTCAGACGTGAAG
AACGTAAGATAACGTTTCAACGTTTGGTCGACAAAGCAAAAACAGTCGTCTCAAGGAAAT
AAAGTGGCGCTTTAGCTGATATCGTGGCACACAGGTAAATGAAGGTTCAAGATTAATAGAC
AGTATTTGTCTCAACCTCGTTTACAACGTATGAAAAAAATTATCGTCACAAAGGTAATATCG
TTTATTATACGTTGATCCGTCCCATCGCTTGTCACGCGTCATCTTTAGTTGGAAATATTTTCGT
TTTCCGGAACGAAAAAGAAGAACGAATGACATTCACAAAAACTGGAGTGTCGCGCGTATC
GGATCTGAGCATTTGAACCAGTTTTAGCGCGAAACGAACGCCATCGTTCCGCTACAAGGGG
ACTGTCATACTATTTCGGGACCGTGATCCAAAGTGCACGAGATACGTAGAGAGCATAAATA
AATATACGCGCGTGTTCTGGCCCCGTCAGAAGTCTCGAGCTGGGGACCGTCATTCGTCTGTG
ATTCCCTTCGGTCGTATGTGTGTTGGCATAAGGCGGCGGTGTTTGTTTTTTTTTCTTGACAGTG
TATAACCGCCCGGTGTATATACTTCTCGTGTTCCGTTTTTTCGTCGTCGTCGTCACGTTGGCG
AAAGAGGAGCGGCAACGGGGAAACTCAGCACTGCGAAAGGGAGAGAAACGAGGCGCGG
GTAGAGGAAACTCGCGGTGGAAGAAGAGCGAGAGGGGACGGTGGAGGTGGTGGAGGGGG
TGGTGGAAAGTGGCGTGTCCAGTGCGCGGTGGTGTGACGGTGTCGCGTCAGGCACCGATTC
CAAATATTTCACATTTTCCGCGCGGCTACGTTGAAAAGTACTGGCCCCCAAATGCACGTAC
CGCAAATCCTGTTTCATGTTCATGTTCTGGAGCAAACGGACGGCGCTCACTAGACGTCTCCT
AAAGGCCAAGGTCCGTGGAGAGCATGAAACCGAGTGTGAAGCTCAAGAGGATTCGTCACG
TATTCATCGCGCAAACAACACGAGTGGCACCGACAACGACACGACGAGCAACGTGACGGC
AACGACGGGCCAAACAACCAGAGGAGCGCGTGGAGCGTCGTCAGGAG

135. >MSTRG.2688.1 Gene=MSTRG.2688
AAAAAAAAAAAAAAGAAAAAAAAAAAAAAAATTACAAGAGTGGAAGAGAGCAGATAAGA
AATAACGAAGAGGCTAGACATTATCTTTCTTAAAATAACCAGTGTCAAAGTACCGCTATGA
TAATAAACCGATGTACAAAGATGCAATTTTTTGGGTTATTTTGAATCGCCTTCTCATCGATCT
TTTCATTTTCGTTTTAGACATAGCCTTGTATCTGTCTAATATATCTTATATTTTATATTTACGTT
ATTTTATAAATTAAAACGGTGTACTTTTAGATTTGTCGATTTTTTTTGTTTTTTGTTTTATTTCT
TTCTTTCTTTCTTTAGAGTCGAGAGAAATAAATGAGAAATAAATTGACGTCTCTTTTAAGAA
AGATAATTTTCTTGGAGAATAATAATGAATGTGACGCGCAGGTAAGTTAACATTTTCTCGTT
CGTTTGTTCTTTTTCTTCTCTTTTCTTTTCCTTTTTCTTTTTCTTTTTTTTTTTCTTTTCTTATATTC
GTTAATGCGTTTGTTTCCACCGAATAAATCACGGAACACGGATTCATTTTTATTCTGATGTC
AGAAATTTTACTACAATGCCTGACACGAGGAAATATTTTTGTTTCGCTTTACGGAGCACGTA

AATTTACATCATGCTTCACATCTGACTGTTATAATATTTTACGATTTTATCTTTTGTAGAAGG
ATAATTGCATGCCAGCCACAGCTATCGATTTTTCGTTTCGGAACGGATTTTAATGTTTAATGC
GCTACTGTCAAACTCTGTCTCTCCAACTATATATTTTTCATTAATTGCGAATCTTTTATTAAC
GAGTGTTTTGCGAATTTTGTTAAACACGATTATACTTGATAACATTATACAATGCATGATTA
CAAATATTTATTCACATTCCAAATGTATATTTATTCGTCAACAAAAATATGTATTATATATAT
ATATACAGTAATATATTTTAATATATACGGTATTAGTATTTAAACAACATTTACATTGATTA
CAAAACGATTCATATTACGTGTGTATTACGTTTGCTTATAATAACGCGCTTATAATGATTTCT
ACGAAAATACAAATCTTGAAAATCTTCAAATCTTTCTGTCAACAAATAAAACGTGTCCAAA
AACGTTTTAATTATTCGAATATATCAGTCCCTGAGACATCGATGGAAAAAAAGGAAATACG
ATTAATTTCTCCACCCTTCACCCCCCGCCCCCAGGAAACGGAATAATTAGATGCACCGTAA
AAAATGAGAGTCGCGTAATTTATTCCATTCCATCATGAAAATGAATTCCAATACAGAGTCC
AGGCCAAAATTATCACGCTTGTTAGCGCGTCAATCGATTCGACTGATCAATTCTGTAATACG
CAGCAAAAAAAAAAATTGATCGGGCAAAAAATGCTATATCCTGGCGGTAAACAGTATTTCC
GGCGGGGGTGGTGGCGGTGGTGGTGGTGGATAATGCGTTATTGGTTCGAAGGCGCGAAATA
AGGAAGACGAGGTGGGAAGAAAGAAGAGAAGAGGAGAGGAGAGGAAAGAGGAGAGAA
GTCAGGAACTGTCGGAAAAAAAGATCGACAGCTGTCGGAAAAATCTGTGCCTTTCGAGAT
AAGGCGGTATCCGCTTCGATTCGAGATACAAGGATTTCACGCTTTCAGCCATTTATCGAGCA
AACAGATTTCCTGCATCTGCCGCGTTCCGCGTGTTTCGTCTCGTCTTCTTCTTCTTCTTCTT
CCTCTTCTTCTTTTTTTCCCCCTCTTTCTCTCCTCGGAAAGCTTTGGAATGAAGCTTTTCGGAA
ATTCGTGCGTTTAAAATAAAATGTCGATGGTTTTACAGAAGAGACCGTCCTACGGTCGGTTT
CTATATCTCGTGTGTGAACGTTTTCGATTTTTATTCTTTATAAATGTTTCAAACGCGCATTGG
ATTGTAATATTAATATTATAAAATTTATACCGTAGTTTTTATATCTCGGTGTAATATTTTTGAT
ACGACGACGATTGTAATTGAGGTTGATTGTAAAATAACGAATTAACGAGAGAGAAAAAAA
AAAGAATTTAATACTACATATACAATTCAGAGAAGCGTAAATTAATGTTGGAGCATTCGAA
GATTATATAAATATTAAAAAAACAATCTCTGCGTATCGACAGATATTTCCGGTGAATGACA
TCGTTAAAACTCTCTTATCGGGCTGAAATTCCGTATTATTTTTCGTGATTACGTTGCACCATT
TGCAACAACAATTTCCACACTTTTTGCTTTAACAACGTCCCCCCTCGCTGCAGAAAATCCAT
TTTACAAAACCGTATTATCATACAATTAAAATTAAAACCGTTTCGACGTTATACCCCGAAA
GCGAAATTTAATTTACAAATTTTATTTTCACCTTTGCATTAAAGCAATTTCCGCCGATTCAAT
TATTGACCTCTCGCGAGTTCATTCTCGCCCTCCCCCCCCCCTCTCCTCGCATGATGCGCCACG
TTTATTAAGTTAAACGACATGTAAAACTCTAATATCGCGCAGACTACTATTATAACAACAA
AGATGACGCTGCGAAACTGTAATCGAAATATATCGAAAAATTATTTCATCCCACAACAAGA
ATCCTTCCAATTATCATTTGCAACGTGACCCTTTCCCCTTCACCCCGAGATAGTACAACTTTT
CACGCGTAAACTTTCGTTCGACCATCGACGAATAACCAACATCGGCTCCATCCACAACAAA
CATTTCCATAAGCAATTCTCTTCTCTGTTTTTACAACACACAGTAAGCGTGTAAAAAGTTCC
CGTCCGTCTTTCTTCTCACCCTAATTCACTCCCTCGATAAAATCTTCCACGATAAAAGGATA
AAACGACGCTTCTTTTTCAAAAGAAAGAAAGAACAACACATACATTTTTAACTTTCAACCA
CGCGGAAGATTTCCCTCCCCAAGATTCTCCCCCATAAATTTCGATTTACAAGAAATTAGGAC
GAGAGAGAGAGAGAGGAAAGCGAAGGCGAAGGAAAAGTTTAATTCTCCCCCTCCCTCTGC
CTCTTCCCCCTCCTCCGTCTTAATCTCAGTAATTTCATCCACCGCGGAACTCGCTTGTTCCGC
AACGAATGTTAATCGGGTTGTTCGCGGTTCCACCAGACAAGAGCGATAAGCAGGAAAACA
CGCTTGCCATCCTCCTTCCTCCCCCCTCCCACCTTGCCTCCCACCCAGCAGGAATCGTGATA
ACGTCACCGGCGAGCAAGGAAAAGGAAAGTCGTAGGAACAACAAATAGAGGGAGGGTG

GGTGGAGTGGAAGGGTGGGTGGTTGCAAAATCACGAGACTTTGAGGCAACGTGAAATGTT
GCTACATGAATAATTCGCGGCGAGCGTGTCGTGGCCCCGGGGGCTGAATTTTGCCTGGAAC
ACGGTAACGAGACCGCAACCGGGGGAAAAACGATATTTAGGAGGGGGGCAGGGAGGGGG
CGAACGACGAGAGACCGTTCCTTAAAGCCCTTGTTTAAATTTCCTATGGATATAACCCTTCG
TCATGGCAATGCAACTAGGAAATTTAATCACTGTATATATATATATATATATATATGTATAT
ATATATCCCGCGGCCACGGAACCGGCGAATTCGGTAACATCTTGCAGAAGTTCGTTATATC
CCTACCGTCGCTCACCCCTTCCCTCCCCTTTCTTTCGACTCGATTCCAGAGGAGGTTTAACCA
GAGGAGGAGGGTTATACCCCCTTGTTTCGTGATATTGACTTCCATCCATCCATCCATTCGGA
ATGAAAAACGGAATGCAGGGTTTCTTGGGAGAGAAATCGAATCGATCTCTTTTCGAGTTTC
GTTCCGAAGGTGGAGAGGGATCGATTTAGTTGTTGTCCCGAATACCGTCCGGGGGGGGGAG
GGTAATCGAGTTACCGGAATTCCTGTAGGTGCGATGGTTAAATAGCAATTTAAGTTGAGGA
GGGACGTTGAAGAGGCGTGGCAGACTGTTTGGTCGGCAAGTAGCGTGAAATTTCGTTTCAC
CCTTCCGACGTACGAGTCGGATGAAATTATGAGCTCTGTTACATCTATTCCGATTATTATCC
ATCGTGGATAACACTTTCGATATTTGCACAATCGTGAAATTCAATTCGGTGTTATTTTTCAGG
CGATACGTGACAAAGGATTAATGATTTTAATCTTTCTTTATATGTCGTCGAATCAACAGGAA
ATCTGTCGGACGAACGTATCGAAAAAGATTAAACGCACTCTTTAGACCGTATATAATTTCG
TAGAAAGCCGTATAACGAACTTAAACGGCACCCGTTTCATTCGCTTCCCCTCGCAACTTCCC
CTCCTCGCATCCCCTCTTCTTTAATGAGCTACCTATTTCCTCGTTGGCGAGTCAAACGTTTCC
TATATCAGAGAAGTTAAATACGGAACCCGAGCCAAACTAATTATCCGCGATACCTCGATTA
ATCCTTCTCGTAACTGCAAGAAGACGGCTATTTGATTTGCCAGTTATCCGCGCGGAGAAAA
AAAAAATCGGTAAAACGGATTAACAACGGGATTTTATAAATGGCGGAATTCCAACGGAAT
TTGGATGATTACCGTTGTAATTTATGATTCGATATCAGGATTAACAGGATTAATTCGAATTT
GTAAATAATAAAATATAATTTATTCCTGCAAAACGTTCGTTAGTTTAATTGCGCGATATTGA
TTCGATTTAAGTCACAGAATCAGAGTGTAATTTAAAGAAAAAAAAGGAAAAATACTCGAA
GCGTTTCGCTGTTTCATTTGATATTTTAATACGGATACCTGTATGCAATTTTATTAGCCAGCT
TTACGACCAGTATTCCGTATAAGAGGCTTTCATGTTATCCCGCAATAAATATCCTTCGAATA
ACTTGCGCAGCTTTCAATATCTTGCTATCAAAGAAAGTTATGTAAAAACGGAAACAAATGA
GAGTCGAACGATGAATGGGACGGATGGGGTTGAATATTCCAGGCCAGGGTCCAATATGTTT
TTTTTTTGCTTCGCTTCCATCGATACACCAATGCCTAACATTGTCTCGTTGAAAAACGAGATG
CTTCTTCTATTCATCAACCTCCAATATTACTTTTCATTTATTCCATTAACATTTTTTTATTAAAT
TTATCATATCTGTGTCTCAACTTAAACTTAATAACAATCTTTGGAAAAAGAATTTAAGCAAT
TATTGATAAAAAAAGAAACACATCAAAGTATATTAAAAAGTAAAAGTAAAAGAAAGATTC
ATCGTGTTCTATGCTCATTCATTAAAAAGTAACGTAACATTTATCATACTTAATCTGGATCA
GCTTTGCAGAATATATAGCCTGTTATCAAAGCATAATTAATACAACGAGTTATCGAAAGTTT
GAGTTGACGAAAGATTGAAGAATCGAGGAAAAACGGCGTCCATGACATCCTCGACCAGAA
TCTAGGTGAACTAGAATTATATTATACAGGATCGAGGAAGCGAGAGATCATTTTATCTCGA
AACTCCACGGAATGTCTCCCAGTGAGCAGAGTCGAGAGTTTCCCGACTCGGCCTCGGGATC
TTCACCTTGTTTTACTCGAGCAGAATGCGATCCGACGACGCGCGAAAAATCTCCTCGCGTCC
GATTAAACTTATAATCGAAACGTCCAACACGCCGACTTATCGAATCGTTTCGCGTCGATTTC
GCGCGAAAATTCGATTTCACGAGGAGAATGTGTGTTTCGCGAGAAAACCGATTTATCTGAA
TGCTATCGAAACGAGGGATCGAAGAAAAAAAAAAAGAATTTTATTGCGATATTTATGAATTC
GATTATTTTCTTCATTTAATTTTTAAAAAGGCAATTTTCTAATCGATTAAAATTCGATTTTCGT
AGCGATAATGAAGCATCGATATTTGAACAAAAAATTTTCGCTCCTAGATGTTAAAGCTCGA

ATATTTTTCTTGACATTCTGCACCGTTCCATTGAAATTCTATCGTTTGTCTTTTCCATTTGAAT
CCGCTGCGTATAAAAAAGTTAAAATAAAAATGTCAGCTCTGCTCGATATGGTTTTATCTTTA
TTGGAAACACTACTCTCGTGTTCGGTATCATCGTCTTTTAAAAATACACGCCGTGGAAATAT
ACGCCTTGTTGTTCTTTTTTCTTTTTTTTTTTGCGAACAAAATTTTTAAAAATTGAATATGTAA
CAGTGTTTCACAAGTGTCTTCACCGAATTAATAATAATAAAAATTACTTTGAAATGATTTTG
TATTATTAAGACTGGTGCGAAACAATTAAAAAAAAAAAGGAGGAGGAAGAGACAGGAGG
AAGTGGAAAAAGAGCAACAGATCGCGAACATGTCACGCAAGGGGAACAAAGTGCTGTTG
CAATTAAATGTTCATTAAACCTGTATACATATGTGCGGCTCTCTGTAAGGGGAGGGGAAAA
AAAATTACCGAAATGTGGACTGTACGTCATAATTCTTCCATTTTCGCATCGCGTCTAAAAAA
AAAAAAATACATGGCGAAGTTTTTAATCACGAAATCACATAATAAAATCGTTGCAATCGTA
ATGAAAAATCATGTACGCGCAGGCAGAGCGAAATAGGGAAAATGGCATATTGTTAAAACG
GAGAGAAATGGCGTACATTTATCCGCCATATCCAATCAATTTTTGTATTATTAACAATCGAG
AAAATTGGGCGAAATTTTAATAAACGCAGAGAGAATTTATAACTTTTTATTTCTATCTTATA
AAATTTTTGCATCATTTTTGTAAAACGCGTGGTGAAAAATGCAAAATTTTTTTCAAAGTTGG
ATTTAATCTTGGAAAAATGTAAAAATATTATCTATCGTACAAAATAAATAATTTTTCACATG
GCAATTGTCTCGCAGTGTTTCGTATAACTAATATTAATTCTTGACATAAATAGCTTGATATTT
TCTCTTTTTTTTTTTTTTTCTTACAAAAAATAACTATGAAAGAAGCAATTACACTTTTGAGTATT
ATACTTCTCTTCAACACGAACACGGAATATTTCAACCAAAAGGTTCAAAGGGGATGACCCT
GGATATATACGCAAGGAAG

136. >MSTRG.2748.1 Gene=MSTRG.2748
CGTTGACTCCTCAATTTTTCGAGGGCTCGTCCGGGTTCCCCCTCCCCGCCGCTCGTTAGTCTC
TACCCCTCTCCACGAGCTTCTTGTCGACGACCGCGGCACAACCACCACTCGGGGAACCCAA
CCGTGTGCCCTCTCTCGCACACACCCTCGAGTGGTCTTATTTTCTTTCCACCTTACTTTCACC
GCGTTCGCTCACTCTCCTCACTCAAGAATCCCCGAGGAAATTCGCGACCAAAACATCCGAG
ACGAAGTACTCCCTGCAGCCAACATGTCGTCTGCTAGGCCCACCAGCGCGGCGGCTCACCG
CGCTTGAGATGGATCAGATTCACTCCGGACTCGGGGGCCAACAATCTGTCTTGTGAAGTGTT
AATGCATTGACTCTAGCGCTATCTGTAACATCTATTGGAACTAAATCGTTATACTGCGATAT
ATTCATATGTAACACACATGTACATTTCGATGTTTTCATGTACGTTTAGTTTTATAATATGAC
AAGTGCGCTGTGATCAATCCATTTATATGTGTGTATATATATTATATATAAATATTCATATGT
ATTAAATGGATATAATTA

137. >MSTRG.2784.4 Gene=MSTRG.2784
CAGAGGCTTACGTGAATCTGGGCGCGGCGCTGGCCTCGGTCGGACGTGGAACGGAGGCGG
CTGCGGTTTTGCGGGCCGGCGCATCCTTGGATGGTTCCGGGCTCAAGGACAAGAGGGCTCA
CGAGGCGGCGAGGGTGCAGGCTCTCCTTCAATTAGGCGCCCTCTACGCCGATCAGGGCAGA
TTACAAAGGGCCCTGTCCGCGTACAGAGAGGCACTTCACGCCCTCCCGGATCACTATCCAC
CTCAGAGCGTTTACAATCTTCTGGGGGAAACTCTGTCGAGGCTGCAACAGTACGCGGAGGC
GGAAAGGTGGTTCCAGGCGAGTTTGGCCAGCCAGCCTGACCACGTGCCGGCCCACATCACT
TACGGGAAACTTTTGGCCCGAAACGGCTCTCGTTCGCCGTAGTCTGACCACGTAGCGACCC
ACATCACTTAGCAAACCTTCGACTCGGCACTCGAGCCGCGTGCTGGAGGCCGAGAGATGGT
TCC

TGAGAGCCCGTAGATTGGCGCCGGACGACTCGAGCGTGCACCATCATTACGGACTGTTCCT
GACGTCCCAGGGCCGACTGTCGGAGGCGGCCGAGGAGCAGCTGCGGGCGGCGGAGCTTTC
GAGATCCGATTACGAGCTGTCGATGGCCGCGGCGTCCGCGCTTCGGCAGGCGGGCCGGCTG
GAGGACGCGGAGGTGTGGTACAGGCACGCGGCGAGGCTGAGGCCGCACGAGGCGCGCAG
CCACACGAACCTGGGCGCGATCCTCCACCTGAACGGGAAATACAAGCAGGCGGCGGCCGC
GTACAGCGAGGCGTTGAGGCTGCAACCGGGCGACGCGACCACCATCATGAACCTGCACAA
ATTGGCGGCGATCCTCGCGTGACCAGCCACCGGAATGGGACACGACGCGACGACGACGAA
CCCACGAGACGAAGAACACCATCACCACAACCACCACAATCACCACTCCCCACGATCACC
GACTCGCCGTATCCTCCCACCCACCCACGTCACCCCGATCTTCCTCCCGATATTCGTTTGTAT
CCGTCGACGGGCGCGCCGACGTCCCCGCGGGGCACAGCTAGCCGTCATCCCCCGGGGGA
CCTCGACGCGCCCCCGACTCGTATCGCCTGCTATGGTATGTAAAGAATAA

138. >MSTRG.2787.1 Gene=MSTRG.2787
GAAAAGAGAGAGGAAAAACTCCTCGACAAATTTCTTTTAGATCGATCGACTTCGTATCCGT
AGGAAGGAAAAAACGCGGCACCCTAGATCACAGAACACCAACGAGATTTCAACGAGAAC
GTCGAGGTTGAGCACATCGCAACATTTTACAACACATATGTTTCATATACATGGTTATATAG
AAGGGAGAGAAGATTTAAGGCGCAAGCAAAAAACACACCACGTTGCTCGCTGTCGATTTT
TTAATCGTCCTATACGATATATTTAGTCCGCTGTACACACACATATATCCAAGCAAGCTAAT
CTCGATCAATTACTTGAACGTAATTCACGTGAATTTAATGATATGAAATGCGACGATGATAT
TATACGTGTAAGCAATTCGAGCAACAAATACCGATTTAAAGTATAAGTAGGGCATAAGTCG
GACGGATGGTCGCGAGATCCGCCGTTTCTCTTTCGTTGAATGGCGACGCGTCGGAAATGAG
AATGAAAATATGGATAAATGCAACAAAACAAAAAAAAAAAAGAAGAAAAAGAAAAAAG
AACGCGGATGAAAATCGAGGAAGAAAAAAAAATTTTAAAATATTCGCGTTAACATTTGAT
TCTTGTTACATGGATGCGATGCCAGGATACGGTTACGGCGCGTGTCTCATAGTTCGTTTTTAT
CACAATACTATTTAGATATAAAAAAAAAAAATATATAAATATATAAATATATATATATATA
CATACATTGACGTTCATATACATACGTTGTAAGTAGGTATGTCTAGATGTCGCATTTATTTGC
CGAGGCGATGCACGAGTGGTACACCAGTCGAGCATGATCACGGTCAAAGGGATGTCTCCT
CGTTGACGACGAGATATTGCCGGGGTACTTTGACCGTGAATTCGTAATCCGTTCTATTCAAA
AAGGGATCCGCATATATATCGCTGGCTACGATTCCTGGCTCGCCGACGCACTTTTCATCGAT
GTGATTTTTAAAAAAGGCTGCGCAAAGTCTCGTGTCTCGTGTATGTGTTACGATCGCGCTCT
ACCACATTAATGTAGCCATATCCAACTCACCTTTTACGATGTATATCACGTTTTACTCACGT
AACGCTCGCGGAGCTTTTCGCTTTCAAGCTTTGGAAACTTAACGCGAATAAAAGGGAAATC
GAAAGATATTTAACTTATTCGATATTATTAGCCGTACGATCGATCTCGCCGCACACGCGTGT
GACGCGTCGATATTGAGGAAAAGAAGAAGGAGAAGGAAAGAAAAAAAAAAAGGAACAT
GAAAAACATGAAAGAAAAAAACGTGATGTCCGCGGATTGTCGTATAGCTTTCTTCTATTAA
TCGATTGGGAAATATTATCTGTTTATTATCTGTTATATTTTGTGTGATCGATCTGGTATCGCTT
CTATTTGCAAGGTTTAACGTTACTTTTTTTTTTTTTTTTTTTATAATGCTGTGAAACGGTCGG
TGCCGCCGTCCGCGTTATATACGTATATATATATGTTATGCTTTGTTCATTGAAGATATTGGA
TGTCCATTTTTGTGCAACGACGAGGCGGTTCTTCGAGCTTCTCTTTCGGCGGGGCGCGTATC
GTTATCTCTTTCGGTTGCAAGCAGACTCGGGACATTTCGTAATTAGTACCCTGTGTTGCGCTC
ACAATGTCAGTGCTCGCCTTTAAATTGGCTGTAAGTTCATATTCTCGTACAACGATACTCGT
ATTTCTCCGCGTTCGTAGGGACTCGAGGATCCGCTCGCAATACACCGTTTCCTCAGTACCAC
TATTTTATGTTTTTGTACCAAAGTCGTAGTAGCGTCTAACTTTACGTTAAATAAATAAAACC

AAAAAAAACAAAAAAAAAACAAAAAAAACAATTATTTGTTTATGATTTTTAGATTCCCAA
ACATCTATATTATAATCTAATCGTATCAAATTAGGAAAGATTATTTTTTCTTTTTTTTTTAT
TAAATGTAACATTAGAACAATTGTTATATCGTACGTTTATTATTTTAAAAAAATCGCTAAAG
TATATTTTATCGTAATACGAAAAAAAAAAAAGAAATACGTTATTGATTTGCAATATCAAAT
GTTTTTACACGATTTTTAGCCTGTAAAATTTTGAATGTTTACGATATGGTAACATTTGAACGA
TGTGCAGTGATAACTCGTAGTGGTTGGTAACATCATTTGTTGGCACGATGGCGTCTGGCGGT
GGTACGTACCGTTTATTTTGCTTTTTATCATTTAATGGGTACCGTTAAGCGATATCTTTGTTGG
GAATTTTTGGTATTAACAATGTCGATCGACGTATTCTTTTTATAATAGCGGAAAATACACCA
GAGGAAAGGACCGAGCCAGCGGCGCCGGCACAACCCCTCACCAATGTTGTGAGCGAAAC
AGCGCACAGGATATTAGCAATGAATAAGGATTCTGATCTCACTGGTGTGCCACCAAAGAA
GTCACGTGTCGAAGTACAATCTCTTCCAACGAGGCAATATTTGGATCAAACTGTAGTACCA
ATTTTATTACAAGCTCTATCCACATTGGCCAAGGAAAGACCCGCAGATCCCATCAGCTTTCT
AGCTGGATATTTGCTGAGGAATAAAAATCAATACGATAATGGTGAATCTCCACCAACTAGT
CAATGAACTGCTTACGCATTTATAAATATGTGGTGAAAATTAAAGAATTACAAACATTTATT
GACACTTCTTAGGAACATACTCAAGTCGGGCAAGGTGTATTTGTGTAATTTTGAATTAAATA
AAAATGTATTAGCTTTTTATCGTATAAAA

139. >MSTRG.2789.1 Gene=MSTRG.2789
CGAGCGAACGACAACAACGAAACTGTACAAAGACACGATTGATTAACGTGCATGCATATA
TATATATATATACACACATATATATTTCCTATATTCTCTGTATATAAGAAGCGAGTAGAGTA
CGAGAGAAGAGGGAACATAGGAAGGAGAATACACGTACGTAGCTACCCGCCACGTTTTTT
TCTTAGTTAGCATTGCACGAGGCCAACGCACCCACAAGAAAGAAAGAAAGAAACCACCCA
CGCCACGGATTATCCGA

140. >MSTRG.2900.2 Gene=MSTRG.2900
AAAATACATATCATGTTACTCTTACCGAGAACTAATCGAGCTATTTCCGACGATCAAAAGT
GGAAAAAGTTCTTTAAATTTAGATACATTATAGATTCATAACAAATGGGAAATTGGCAAAT
GGAGGTTGGAAGAATGATGTCATATATATTATTTCCCATTGGAGTTTATTATTATTTTAATCA
AACAGAAAATATTGAAGAATGGATAAAAACTGAAAAGAAAAAGTTTCAAAATTCAGATA
ACACACAACATACAGAATTTATGAATTTTATTGAGGAATTTAATAAAAAACAACAGTTAAA
AAGAATTATAGAAATGGAAGTGCA

141. >MSTRG.2921.2 Gene=MSTRG.2921
AGAGAAAGAAAGAGAGTGAGTGAGTGAGTGAGAATGTGAGAGACTGCAAGGGGAAAAG
AAAGAGTGCGGAATAAACGAAGAAGAGAGGATAGAAAGAAAGAGAGAAAGATGATGAA
ACGGAGATGACGCGAAAGAGTACCAATATTCTAAAGTAATTCTAATGATTGAATTAATAAT
TTGATAATTAATCGATATTGTACATCGTAAACGACGTAGCGTGTAGGATATTAATCAACGA
GAACTACGAACGCGGCGAACGATAATTAATTCGTTAAATTAATTATTATTACTCGGAGGAG
CGAGGAGAAGAAGAAGAAGAAGAAGAGGAGGAGGAAGCGTGATCGGGCCACGCTGCGA
TTTTCGATTTATTCGTTATTCGTTGTATCATAATAAAGTCGAATGTTAACAGGCGCGTCTCGA
TCAGGCTTGCTTTACGATCATCTATAAATAATCTACGCCGCAACTGTCCACGCGATGCCGTC
TATCTAACGAGAAGAACGCGAATTCTCGCCGAAAAATATTGTCAAGCTCGAACATTCTCGT
TCTCGAACGAGGAAAAAAAGAAAAGAAAAATAAAAAAAAAAAAAATACTAAAAGGAA

TCGATCGGTAATCTTTTTATACGTCTAACGGCCATGATCGATTCGAATCATCCATCGTTTGTT
CGTCGGAGCGGGAAATCGAGCTGGACGGAAGCACACGGCACCATGTGGAAAGTCGATGTG
GAGTAACGTGTCTAACCGAAGGAAGAAGAAGAAAAAAAAGAAAAGAAAAAGGACAAGA
AAAAGAAGAAGAAGAAGAAGAAGAATCGGAACGTCGATCGATCGCCTCCGTGGATCGA
CGTTCTCGTCCATTTTTCTTCCTTCGTTTCGGGCTTTCGAGCCTGTTTAGAAGAAGAAGAAGA
AGAAAAAAAAAAAAAGAAAAATAGAAAAAAGGGGGAAAAAAAAAGTGGAAAAATCGAA
AAAGCGAAAGAGAAGAAAGATCAACCAATCGATATAGGCGTATAAGTAAAAAGTGTAGA
TATATCATAGTTAGATACAACGATCTCAAGAGGACACCCAGTGTCGACGATCGCAATGAAT
CGAAAGGAAGTGAGAGGGAAACGGGGCATCCTCGACGATCGTAGAAGATGCCTCGGTTTA
CAAATTTTCAGCGTAATCTCGTGGCACGTAATTACCACGAATAATCGTTAAAGAGTCTGAG
ATTTAGCAAGGAATTATATATATAAATATATATATATATATATACATTACGTACGTGTACAT
GTATACAGAGATTGAGAAGAAGAATACGAGATTATTATAAAGGGAGAAAGAATTAACAGT
TTCGCGCGATCGCGTGTTAAATGAACGATATGGTTGAATCGTGAATCCGTTCGTGTAACGTT
TGTCATCGTTGCATTCATTCTCGATATAACTTGGTTTCCCATTCCGAAATGGATTTATCGTTCT
CTTATCCTTTTTTCTTTTCTTTCTCGTTCATTTTCATACTTTCATCGCTCCATCGCCCATCCACA
CACACGCACACGTATATATATATATATACCCTCCCTCCTCCACTTTTTCTAACTTACCACCAC
GCGTTCCCCCCACTCCCATTACTCCCATCTCTTCTTCCCACCCACCCCCCCATCTCCACTCGG
CGGCTACCTACCGAGTGGTTCGACACTTCACCTTCCTTCCTTTTTCTTTTCCTTCGTTACTCGT
GACCTTAGTATCGTGTTATTTATTTAGAGTTCGAGCGAGTATAAACTTACCGCCGGATGTGG
AAGAAATTAATTTAGGTATTCGCTCTTTTCACGTACCTGAAAGCCATCTGGCATAGCATATT
TTTCCGAATCGCGAGTGAAATTCTTTATCGCTCGAAAAAGCAGAACGTTTAGTAGCGATAA
CACCACCAATGCGGGCCACGTTACTGGAACAACATTACCGATTCGCGGTCGAGTTCGAGAT
GGCGCGAGAGTCGGATCAATCGGTTAACCATCCAAACGATTTACGATTTATATTTTACTCGC
ACACCGCGTTTTTACCTATTATTATATTTACGATAGACTCGAAAGAACTCGCGAAAACATTG
CACAAGATATATATATATATATATGTATATGTATGTGTATATGTATGTATATGTATATATATATC
GTCGAAGCGCGCGCGATTAAAACGCCTAGAGAACATCGGTTTTCGACTATCTTTTTTTACAC
AGCGTCACTCGTGGCTGTAAATCTAAAATGGCTCTCGGGTACTGTACCGTAACGCTTCACTT
CACGGTGGTTGCGATCTTTTCTCATCACCATCACGGTATTCCGCATTCTTTCGACGCGGGTTT
TCCTTCGAAAGTCGACGAAAACCGCGAAATCGATCGTTTGATGATGGAGAAAGAAAGAAA
GAAAGGGAGAGAAAGAGAGCGAGCGAGCGAGCGAACGAACGAGCGGGAGAGAGAGAG
AGAGCGAACGAGCGAGAAAGAGAGAGAGAGAGAGAGTGTGTGTGAGGTAAAGGAAAGG
AATGGGATTCTAGCGCCGCCAAATGAACGATCGTCGCAGTTCCAGATACGAGTTTTCGAGT
GTTCAAAGGTCCATCGGCATCGAGTACACCGACGACAATTTCTAGCACAACGTCCAATCGT
TCGTTGAACCGTGTTCATTGTTCGTTTTCGTTTGATGAAAAAAAAAAAAAAAAAAAAAAAGA
AAAAAGAAAAAAGAAATACGAGAGAAAAGCGAAAAAAAGAAAGAGAAGAAAAAAAAG
AAAAGGAAAAGGGAAACGAACGAGAGGCGCGTTTTCGTTAGTCACATTCGCGAGTGCTCG
CTACTTCACCTCCATTTTTCACCGTTTTATTGATTGACCTATGCTTTCTCGCGAATCTTCCCCC
CTTCCCCCCTCCCTCCACCCACGAGAACTTCAAGACGTACTAACCTATAGGACAAATTGTA
GACAAATTTATTATCAAATAAAAATGAAAAAAATAAAGCGACGTTTGAAAGGAAAAAAA
AA

142. >MSTRG.2925.2 Gene=MSTRG.2925

AGACAACCTACATTATACGTATAGGTTTACATACTTTTCTTTATTTTCAGTATTTAAAGTACA
AAATCGTTACGAGTACGGGTAAGCGCCATTACAAGAACAGTCTCCAAAAGCATTAGAAAA
AATAGAAACACCAAGAATGATGTTTGATTCTATTAAAACTAAGATAGCATCTACATCTAGT
TCTCTCTCTAGACATCATTCTACAATACCTCAAAAAATGAAAAAATTACAACAAGAATGGC
AGGCTGATTTATTAAAACCAACATTTCTTTTACGAGGCGCATCAGATATGGCAATTTATCGT
TTAACCATTGCAACAACCTTGATTGGTTTTGTTATTAATTTATATTATATAAATGAACTTCGA
ATGAAATTT

143. >MSTRG.2942.1 Gene=MSTRG.2942
TGTGTTGTATGTATATATCTACCTACATAAATATGAGTAAAACACAACAGTACCTTGCACTT
GCCGCTCAGTTTGAGCGTAAGCGACCTGGTGGCTGGTGTTCCATCGTTGTATGGCGACAGA
GAGAGAGACTCACGCAATGATTCTCTGAGTCAGAAGACCAGGAACCAGGAGGAAGTTCCT
GATTTCTCCAAGGAACAGCAGATTCTGGAGGAATAATGGATGGTGGACCCATAGGTCCAA
AAGGTGGTCTGACATGGGGACGAATATCATCAAAGGGAGGTCTAAAAAAGGCATCTGATT
CTCGGGGATCGAAAGGTGGTCCACGAGGTCCAAAAGGCCTCATTCCAGGACGAAGAGAAA
ATGAAAATATTTTTACCTGGTCCGCGAACTCCTAAAGGAGTTGGACGTGGTCCAAATCCAT
CAGGATGTCCAGGTCTTGGACCTCTAGGTCCCATGATTGGTGGCATTCCAAATATAGGATCG
ATTCCAAAGAATTCTCGTCTCCTAGGTTCAAAATCACCTCGATCAAGATGCCCTCTTGGATC
GAAATTATCTTGAAGTCGCGGGTCAAAACTATCTCGTCTTATTTCATAATCAAATCGATCAT
CAAGTGGTCTTCTTCCTCGTACTTCAGATCTATCGAATTCCTCCCTTTGTTCTCTGAGTGGTCC
AAGTCTTCCAAAATCTTCTACTCTTTGCTGTCCATGTATTCTATTATCAAATTCTTCTTGATAT
ATTCCTCCTTGCAACCTTGGA

144. >MSTRG.2981.1 Gene=MSTRG.2981
TCTAGATTCTAAACGTTAGTCAAGAAAACGCGGCTGATAGCATCGCGCGTTGTTAATCAAT
CTAATCGTGCGCGGACGAATTTGGAAATCATTGAGATTTTAAAAAAAAATTCATTATTTTTC
AAAATGCGTTTCTGTGGTATCTACTTGATAGTTATTTTTACTCTCTGCACACCGTCCTCGTCG
TTCAAGCTAAAACCGAATTATATCGAAGTGTACGACATTGACGACAATACCTTAAAGCAAT
TACCTCCTCAAGATCCTGCATTTCATTCTTTTCCAACGATACCTACTGTTCCACTTATCCCTA
CATGTATGGAAAAAGGATATTTCAGGGATCCTTTCAACTGCAAAAAATTTTACTATTGTCGA
TACGTGAACGCTGTTCCAAAAGGTTTTTACTGTCAAAATGATTTGATATTTAACACGATTAC
CGATCAGTGTGATAAACCTAGCTTTGTAGATTGTTGATTAATATTGTATTCGTTAGAAAAAT
TTGTAGTACAAATATTTTTGACTGTATGATTTTATTTATTAATTCTCATGTATGTATTATTTAA
GAATTATGAATAATCTATGTCTTCAATGTTATAATATTAATATTTATTTATTATATCTTA

145. >MSTRG.3014.1 Gene=MSTRG.3014
AAGTAATAGATTGTATCGTGATCTGTGACCAGTGTGATATTTGTAATTAATTTTATATTGATA
TTAAATTTTAAAAGAACACATTTATCGCATACAAAGGATTAGTGGATATTTTTTTACGTAAA
TAAAATACATAAAATTTTACGTAAAATTACTACAAAAATGAATTATAATATTCATATATTA
ATATGTCTTACTGGTATTACTGCTATTCATGTTGGTTGGAATATGGTACAGGAATATAGACC
AGAAAAAGATCGACTAGGCAGGTGATCAAAATTCTGTTGATAATTCATGGAGGAGCTTTTC
GGCCGATTTTCATTTTGACTGTGTACCGAAGCACCAGCTTTAGGAAGTTGTGGTAATACCAT

146. >MSTRG.3022.2 Gene=MSTRG.3022
GTTTTACTCTTCGAGCTTCGTCTACCGGTTAGCCGATAAAAAAGCTCCGCCGCGAGACTCGT
CGTTGGACGGTGTTCCAGGCGCGTTGCCGTGTTGGTTTCTGTTCGTGCTGAATTTCATCGTGA
CCGCGTTCTCTTGGTGCGAGAAAATCCAGTTCAGTGGATGTACGAGAAGAGACGGACGAG
GGGTCGAAGGTTGGAAAGGTTGGAGAAAGAAAGGAAGGATGAAGGGGAAAGAAGAAGA
ATTTCGACCGCCCGAAGAAGAGGCAACAAGACGAAGGGGAGAAAGGAAATTTTAAGGAT
ATTGCCACGGTTATCTTCCACGATCTCGGCATCTGTTCTCGTGATCGTTCTAGTTGACGTCCA
TGGCCTCCAACGGTCGGCTATAAGGGTTGCTTCTCCGTGGAAACCCGGGGGTATCTAGAAA
CGGTGCATCGTGCATGCGCTAATCAGCCGCCCCGAAGAGTTTTCACGGTAACTGGAGCACG
GTCGGTCGATCCGGCGCGAAGAGGGGCAAAAGAGGTGAAGCTCGCAGAGACAGGAAGAT
GGATCGTTGCTCAGGTCCCGCAGCAATGTCCTTGGACGCTGTCTGTGCCTTAGGACAGCAC
GTAATCATACCAGTCCGCTATTACTTCCTATAACGCTATCTACGCCAGGAAACGCCATTGCT
CGCTCGTTCCTACGTAGAAGGATGAAACTTGAGGAAGTGACGGACTCTCTGTTCTATCCGTT
GCGGACGAATGGTCTGTGTCACCCTCTCGAAGCGGTTGGAAATTAACTTTGAGTATTACAG
ATGTCGAGGCGCGCCGCGGCGAGAAGCTAGCACCTTGTTAGTCTGACAAATTCGGTCCGCT
TCCCCTTCCTTCTGTCCTCCACTTTGCCGTATAAGCAGTTAGTTCCATTCCGCGGAGAAACAC
CGAGCCTGTTACTTACGGGACTATCTATTTGTAAATTATATTTTCCTTCTTTGATACAAGCAC
AGGGATCTTTGCTTGATCCGTTTAAACGAAGTCCTTATCTTGTCCTGTACAAATTCTGCTCCA
CGCTGAGTAAATTAGTTGGCAGCTTGATTAAATGTAAACTCGACTTAATATACCCTTTTTCC
TCCTCTTTTTTTTCCCTCGTTTCGCGTTAAAGGGGGAAGTAATTTTAAAAAGAGTTTAATCTA
AAAGAATGTATTAAATGTTTTTTCATAATTTAATATATTTTTTAACGTATATACTCGGTTTTTA
ATCCTCGGGATTAATATAATTTTATTGAATTAAAAAAGGATAAAGCGAAGAG

147. >MSTRG.3025.1 Gene=MSTRG.3025
CGACGTGGTGTGACACGTAACACGAGTGGGAAACGCACTCGAAACACGCGCCCTCCGAAA
CGATAAAATGTCTGACAACACGAGAGAAGAAAAAACCGGGCAGTGACTGTGAGGAAACG
AACAGGATATCACGGACCGCTATCCAGTGCGCTGTTTTCGTTTGTGAACACCGTTTACGCAC
GTCCTCGAACACGTCTTCTTGGGATCGCCGCGATCGCACAGATTGACGAATGACGACGTAA
CAGAGAGCTGGCTGGTGCGCGGATACTGGGACACAAGAGGGGACGGCATCTTTCAAATCG
CCACGGCAACGGTTCATCACGAGATAAGAAGAATCGCGGGAAGGAAAATCGAGACGAGG
GAGCGAGGGGCACCTAGCCCTTTGGCGAGCTCGAAGGATGCAGCTCCCAGGCGGGAGCAC
TAGAATTCCGCCCGCGAGAGCACCGGCCTCAGTTCTCGTGATTATAAACGGAAGCAACATC
GTGTCGAAGACTGTGACTGTGAAGAAACGTGACGGAGAGTGTAGCGAAAACGGCGGACCC
GGAACGAGCAGCGATAGATACATAAAGAGTAAAAACGATAGGAATAACGATCCAGAGGA
CGTGCCGCTGCTCCTGATAAGAAATCGCCATCCCGAGACGAGGACGACAAGCCATCGACA
AGTCACCCGAGTCACCGAGAAGCCCCAGCTGTTGGCGTCAGGATCCCCAAGGCGGAAGGA
TCGGGGCGAGGAATAGTAAAAAGAAAATAGTAGAGCGAGATCGAGGAACGACGGAAGAA
GAGGAAGAGGAGGCAGGAGAGAAGGAGAAAAAGGTGAAGAGGAGGAGAAGGCTAGAGT
TCTTTGGGAAGGAG

148. >MSTRG.3031.1 Gene=MSTRG.3031
AAATATCTCGTTTATCATTCGGAATATTAAAAAATGTTTCAAATAAAAGTTGAATGGTTTCG
AAAGGGCTACATCCTGACGCTCTCGTTTTTTTAGTAGGTTGACCAATAAAGAACTTTCAAGC

ACAACTTTATTTTTTTTACCGATAACCTGTGTTTTTTAATGCACCAATCGATAGAGCGTAAAA
TTTGCTACAAAAAAGTATTAACCCACTTATAGCGAAAAACCAATAGTTTAAAAGATATAGC
AAAAATTAAAAATTTTTAGGAGGTCGACCTCCTT

149. >MSTRG.3031.2 Gene=MSTRG.3031
GAAGGGCTATATCCTGACGCTCTCGTTTTTTTAGTAGGTTGACCAATAAAGAACTTTCAAGC
ACAACTTTATTTTTTTTACCGATAACCTGTGTTTTTTAATGCACCAATCGATAGAGCGTAAAA
TTTGCTACAAAAAAGTATTAACCCACTTATAGCGAAAAACCAATAGTTTAAAAGATATAGC
AAAAATTAAAAATTTTTAGGAGGTCGACCTCCTT

150. >MSTRG.3031.3 Gene=MSTRG.3031
AGCACAACTTTATTTTTTTTACCGATAACCTGTGTTTTTTAATGCACCAATCGATAGAGCGTA
AAATTTGCTACAAAAAAGTATTAACCCACTTATAGCGAAAAACCAATAGTTTAAAAGATAT
AGCAAAAATTAAAAATTTTTAGGAGGTCGACCTCCTTCGTATCATGTTCTCCTAATACAAAA
TCGAGTTTTTGGGCAAAACTTTTTCTTCGGTCGCCGGAATTTCGACGTTTACACTTTTTTTTAA
ATATCTCCCTTATTATTCGGAATATTAAAAAATGTTTCAAATAAAAGTTGAATGGTTTCGAA
AGGGCTATATCCTGACGCTCTCGTTTTTTTAGTAGGTTGACCAATAAAGAACTTTCAAGCAC
AACTTTATTTTTTTTACCGATAACCTGTGTTTTTTAATGCACCAATCGATAGAGCGTAAAATT
TGCTACAAAAAAGTATTAACCCACTTATAGCGAAAAACCAATAGTTTAAAAGATATAGCA
AAAATTAAAAATTTTTAGGAGG

151. >MSTRG.3080.1 Gene=MSTRG.3080
AATGTGATAAGTTGTCATCGCATAAAATTATTAATGTTAGTTTGACAATGTAGTTTAGTCGA
CACTACAGCGTCTTTTCAAAAAAAATATTCTAATTATTTGATGTTTTAATATAAAAAGTGCA
CTGGACTTACCGTACGGCAACGCGTACACTCGTATCATCAGACATTTCGATGTTAAGTATTG
TGCAATTCTTTAAGACGTAAAAGTAACAGAACTGAAGAAATCAACGAGAAATTACGATTA
GGCTGAACTATTAGGCGACAGTTATAATATTTCAATACACGAATATACTGAATGCGCACGC
GTTATTAGATGCGGTGGTTGTGAAAATTCCGCATTTTTCGTCGTGGACGCGCGCATAGATAA
CCATAAAATCCACTCAAACATGTCTCATAACGAAAGGAAATCTCTATCCTAGCCGCGTCAA
CTCAAGGATTGTATGGATCCTGAACTGAAATCATACGTGACAGACATCTTTAATTCTTTATT
ACTTAAAGTGACTATGTGCATCATCTGTTGATGGTTTTAAAAACTACTTACTACTTTAAAAA
TAACTTTATCAGTTGGAAATTATCGACTGTTTTCTATTCTTTTTTAATTGTTGATAACATTGGT
CACTTTTCCGGTTACGATCATTCTTTCAATATAACAAGATGGCTGATCAGAGTGAACGTGCA
TTTCAAAAGCAACCCACGATCTTTCTTAATCGTAAGAAAGGTCTGGGTCCCAAAAGACGGA
AGCCTATGAGGTATAGTCGTAATGTAGGTCTTGGTTTTAAAACACCTCGTGAAGCGCTTGAA
GGAACCTACATTGATAAAAAATGTCCATTTACTGGTAACATTTCTATTAGAGGACGCATTCT
TACTGGAGTAGTACAAAAGATGAAAATGCAAAGGACCATTGTTATACGTAGGGATTATCTT
CATTATATCAGAAAATACAATCGTTTTGAAAAACGTCATCGTAATATGAGTGTTCATCTTAG
TCCTTGCTTTAGAGATGTGGAAATTGGTGATGTAGTAACAATTGGTGAATGCAGACCACTTA
GCAAGACAGTTAGATTTAATGTTCTGAAGGTTTCAAAAGGAACAGGATCTAAGAAAAGTTT
CAAAAAATTTTAAGGAGATACTAATAATATTGAAGAAAAGAAGGAAGTTATATAAATTAT
CATCTTGACTTGTATCTTTTCATGTTACAATTAAAAATAAAAACATTTCTAAATAAAAACA

152. >MSTRG.3161.6 Gene=MSTRG.3161
AGAAACGGTATCAATAAAAACGATCAGCGATTGCTAATATCTAGACATGAATCAAACATTT
GATAAATAAATAGGATAATATATTCGATTGTTACTTCGCATAGAATTATATCGTGCGACATT
CTTCTCCGTTACATTTGTCTATTTTAATTTCGGAGATCGATATATATGTAGGAAATCGCGAAT
TTCGAAACGTAGAATAAGATATACGTCATATATGAGAATTGTTCAAAGAGTGGGCAAAAG
AATGGTAAATTTGGCAACTGTCACGATATTTAGGAATTTCTATTTCTTTCTTGAGCAAATTT
CGAATACCATTCGCTTTATAATCGTAAGAAATATAAGATAATCGTTGAAATTTTTCCATATT
TCATCCGTTATTATTATTATGTTTTTTGAAACAAATTAATTATAAAAATTAGAAAATAAATA
GAAAGTAAATATTTTAGAATGAGAAACTAATATCGAAAGAATCAATATACGCACACACAT
ATAACACATCATCTTTGATCCTTTGGTAAATGCGACACGTGTTCCTCACTAATAGAGACACG
TGCTGTATCATGTTTGTTTTTTTATTCTCATGGTTCTTCTACTTCTTGGTTAGACGTACGAAGG
ACAACCTTGGCTAATGGACACTGGTGAAACGTGTTCTGCACAGCTCAAAGCAACTTACTGT
TGGTTACTATGTACTTATATATGTCTAACTTTCAAGGTGACTTCAATCGCCTTTTTTTATGCCA
ATGAACTGTTCTCTGACGAACTTAGACACGTGAGTCATTCGTTTTCGAAGATACAATATTTT
TATCTCTTTTTGAAAACACCAATATTTTCTATCATATTTAATTTGGATCTCTAACATTTTTGTA
AGTTTCATATCTTTGAGAAAACTCGTGTCAACGTGACTTTCTTCACAAATTGATGCTAAAGA
TTATCTTGGTTAATGTTTATACTATTATTATTCATCTGGGTAACTATCATTAATGAAAGCAGG
AATATAAAGAAAAAATATTTGTTGATTACTGAAGTATAGAAGAATAAATGTATATAACAAT
TAGATTATTAGATAATTTTTGAATTATTCTTAAATTAGAATAAATTAGAATTATTTTCAGTTA
AGAAAAGTCAGTTAAGATAGATGAAAAAAATAGCATTCGTGATATATTAGATTCATAAATG
CGCCCAAGGCCACGTCACGCTGCAGCAAACTCCCAACCCCCCACCCTTCAAATATCGTCAT
TTTGACTTGTCTCACGAACAGTTCTGGATGTATCTCGTTGATTCGGGCGCTGCAGAGGGAGT
GGGTGCGTCACACCCACCGATAGCAGGGGTGGGAGAAAGAGGTGGGGTACACCGCATCGT
CGATCCACCCCTGCTTGCTTATTATGTTTACAGATTCATATCCTGTTTTTGCTACTTTGAAGAT
TCTCTTCTGTCTTTATCCCCCCCTCTCACAAGTTTTAATATCGCGTAGTTTGATTTTATCTCTTC
CTCTTATCGTTTCGTAATATTATTCTACTTCTTATTATTTACCAAAAAACAAACATTGTAGTA
AAAACTACAAATATATTCAAAATCATTCCAAATATTTCATGGAAAATAAAAATTTAATGGA
CATAATACAATCTATAATCTAATCTAAATCTATTATAAAAAAGAATCGAGATCACCGGTTT
ACCGTTGGCGAGGAGCAAAGAGGCGGGATGGATTTTGCAGGAAGGAGCTTGTCGATTGTG
CAGAACAGAGCGAGATAGAATTGCGTTTGGGAAGGGGAACGAAGGTTAGTCCATAGGGCC
AGGCAGTCCCGCTGTATTGACGCGCGGCGCACAGGCTTGCGCGAGATGACGCAATCTCCCT
CCCATGCGCCCGGCTTTGCTCGTACCACGTTCTACTCCCCTCTCTTCGTCCCGTTCGCTCGTG
TTTCACTCTTCTCTCCTACCTCACCCGGCAGCTCCCATAAGTTCAATGCCAAGGTCAAAACG
TCCACGACTCGTAGCCGTGTCGGTCGCGATACTCGTTCGCTGGGAGAAGAAGTAGCTGTGT
GTGTGTGTGCGTGTGTGTGTTCGTGCTCTCTCTCTGATCGTATGCTTATTTGCTGGTATCGCTC
ATCCATCGTCGTGGTCATCATCGTTGTGTCGTTTCATCGTGTATTCACATTTACCTTCCTGTCG
CGTATCGCGCGCGCTTTTTCTACCGGTTTCTTCGCCGATGCGCGTTACGTGCCATGATTTTGT
ACATGTACACGCAGTTTCGTTTCGCGTCGCGCGACTAACCTACATTACACGACGTACGGAC
ATGTTGAATTACGCCAAGTTAAAAATGTGTGTTATGATTGTGTCGAATACAAGGCATCGAA
AAAGAAATGAAATATTGCAAATAAATCGAAGTTAATGTAAAATGTCATATGCAGTTATTCT
TCAAGAAATATCTCGATGTTGTGACTGTAACTGTGTTCTGAAATCATTTCGAGACAAATCAT
AAGTGTAAATGTGCGAATATTGGAATATAGAACAAAAGTAGAATATCCTACGCGACGAAA
TACGGATGCGCATCGTGTGAAGAATGGATATAAACTATCGTGACAAATTTCGTCTGGTTCTG

GCTACGACTTTGAGAGAGGACGGTTATCCTGATGGAGGGGAGTGGAACGCGACTGAACAG
GAAGGCGGTTCCAGAGCAGACAGTTTTGAATATGTGATGTCCGGTATGGTTTATCGAATAG
AAGGTGATGAAGCAAGCAATGAACCCAGCAGTAGACTATCTGCCTACGTATCCTTTGGAGG
ATTGCTGATGAGGTTACAGGGTGATGCAAACAACCTACACGGTTTCGAGATAGATCAACAT
ATGTACTTACTCATGAAAAAGTTAGCGTTCTAACTATGTTATATTTGTTATAACTATGTTACA
ATTATAACTTAGAAAATATGTATTTTAAATATATTCTTTTTTCATGAGGAATTATTTTAAATA
TAAGATAAAGATAATTGAACTGAAAAAATATTAATGATTGATGCGTACACAAAAATGTATT
GTATGTGTATTTGATCTATAACAGTATATTTATGTAAGAAGAATGTTTGCTAATTGGTTGATC
ATTTCATGATCACATCATATTTTTCAGTTCATGAATATTTTATTAAATATTGTCACTGCCATTG
G

153. >MSTRG.3180.1 Gene=MSTRG.3180
AGACAGCTGATTGTCGCCATATTTTATTGAATGCTTGTAGCGCCAGTTATTCAGAACTATGA
TAAACAATCTCGAAAGAAGCATAACATCTTGTGCGATTTATAGAATAGTAATTTTTGAGTG
AAGTTATTGACTTGTGTCGGGTTATAGGGTGTCGATAAGTATCATACATAAAGAAATAAATT
TTAGTAAATAATTGAAATGGGTAGCACAGATAAAGCTGTGATCACGGGTTTTATATGTAGA
CTTTGCAGCAAAATGAACCGTTTCGTGATCCACATATACGGTGAAGAGGGTGAAAGAATGA
AACTTGCTGAAAAAATAAATGCTTATCTTCCAATTACGGTAAACATGAATGACCCATTACC
TAAAACAGCATGTCTACATTGTATCGAACGACTAGAAGCTCATCATGAATTAATGGGACAA
TTTTTGTTAGCTAAACGAAGATTAACAAAATCATCAACAGTTGCATCAACTTCAACACAAA
CTGTTGATACTGCGCCAACATCTAGTTCACCTCCATGTTGACATATAGAATTAAAAGCATAT
CTTTACGCTCAATATTATTAAACATTTTCTGTATCTTA

154. >MSTRG.3183.4 Gene=MSTRG.3183
TAATATATTTATTCGTTTGTAGAGAATTTTTAGTATTATATTTATTTGTAATATTAAAAAATTG
TAAAATATATTGAAAAGAAGAATAAAAATTTAAAAAATTATAAAAAAATAAAAAGAAAG
AAAGTGATCTTTAAAGATAAATGCGCAGACGTGGTTTTGCACTGAATTGCTCGTGTCCCGTC
AAATAGAGGGAGTGAAACAGCACCTGCTGCCATTCTGGCATTACGTCAATGGTAGACATTT
CATTTGCTGGTTGGGGAGGAGTTACCGTGACCGCCATTGTTACAGATCTTTCATATTAAAGA
ACGTGATCTGTGAATTTATATTGTCATATTGTTGATTTCATCGAATTGTGTTTATTTTTTAGAT
TTCCGCAACAACAATACAGATATCAGTAAGAGTGTTCTTAAGCATAGCATGTCCACTATGA
ATCCCGAGTATGATTACTTGTTTAAGTTACTTTTAATTGGAGATTCAGGAGTTGGAAAATCA
TGTCTTTTACTCCGTTTTGCTGATGACACATATACAGAAAGTTATATCAGTACTATTGGTGTA
GATTTCAAAATACGAACAATTGATCTAGATGGAAAAACAATAAAATTACAAATTTGGGAT
ACAGCAGGTCAAGAACGGTTTAGAACAATTACAAGTTCTTATTACAGAGGTGCACATGGTA
TTATTGTTGTTTATGACTGCACAGATCAGGAAACATTTAATAATGTTAAACAATGGCTCGAA
GAAATTGATCGTTATGCTTGTGATAATGTGAATAAGTTGTTAGTCGGCAATAAATGTGATCT
TCATACTAAAAAAGTTGTTGATTACACAACAGCAAAAGAATATGCAGATCAACTTGGTATA
CCATTTTTGGAAACATCTGCAAAAAATGCAATGAATGTAGAACAAGCTTTTATGACAATGG
CTGCAGAAATAAAACTTAGAGTGGGTCCTCCATCAAGTGGTAACAGTGATCCAGCAAATA
AAGTGAAAATAGAACATGGACGTCCAATTGAATCCTCTAAATCTGGATGCTGCTGAGAAAT
GTAATTTTGCAATTAAAACCAAAAAGGACATCTTATGGTAGCAGTTTTATACTGCTTAAAG
ATTACAAGAAGACATAAGAAAGATTGTGGAAGTTTCAGTTTATATAAAAAAAAAAAAATG

AGGTGTGAAACATTATTAATTATGGACCACCAACTCTATTATAGAGTTGAATAAAACAACT
TTTGTTCTATCTTCTTTTTTTTTTTTTAAGAAAGAAGAACATAATTATTCATTTGCTAATTATTT
AATACAAAAAATTGACCATAGATAATTTCTCGAAGCATTGATAATCATTGATTCTATGCAAT
GATGTGTTTAACAACAATTCGAATTTATAAAATACGTAATACTATGTATCGTGGTTATAAAG
ATAAGCACTTTTATTTTTTAGCGTTATTGTAACATGTATAATTGAATATTTTTTGTCTTAGAAC
AGTCATTCTTACTCTTCATATTACCCAAACATTTTTCCACATCTATTCCAACTCTTGAGATAA
ACAAGAATTAGAGTAATGATATAATTGAAACATGCCGTTGTTGCTGCCTCCCTCTTTTTTTTC
AGTGCTCATTAGTATAATTTAACAATGATATATTAATAAATATTTCAACATTATTACAAAAA
GTTGTAATACTTATACATATAAAAATGTGTATATGTGTGTGTGTGTATATATATATATATATA
CACACACGCATATTAAAAACACTTCCAATTGGTAACTTCTGTAAAGTTACATCAATTTTTTA
AACTTGCTACTCATAAAATTTATGAAACTATTCGCCTATTATAAATACATCATACCGTTTGT
AAAATATTCTAGCGTTTGAAATTCAATATATTTTGTTCTGTAACTATATTCATAAAGTGTGTT
TCATATGAATTTGTAAGATAAAATTTTTTAACTACAATATTACTTTACTTTTGCATCAATTAT
TATTCAAATCTAGTTATATTATTTTGTATTACATACTCGTTATCAATTGTTTTTGCTTTCTTATA
AAAATACTTAAAAAATATTTAATAAATCAAATTACTCTAGTAAAATTTTATTCTGCAATATT
AATACCAAAAAAGACTTTATATATCGTATAAAAAAAGAAGAAAGAAAAGAGAGAAAAGA
CATATTTTGTACAAATACAAAAAATATTACGATTGCAATTAATCTTGTTTTTGTATTGTTATT
ACAGAATTTTTATGAAACAAAATAAGAATATTTCGTAATTATGGGATTTATTTCAATATAAT
AATAATATGAATTAATTAATAATAATGTATGAAACTTCATAAAAAGAACGTATGTAATAAG
TAGAATGTATGATTATAAAAAATAAATATCATGTATAAGTTGAAAATTAATTATTTTTTATA
AATACATTAATTACAATCTAATTTTAATTTGTAATGGTATATGAACTATACGATATATTTAC
AGATATTCAAGTAAGTATAAATGTATATGTTCTTCTTTGTATAGTTAATATGAAATAGAATA
GGATACATTAAAAGTTAAATAATTGAAAGATAAAATGTTTTTTAGTCAAATTGTAGTTTTTA
ATAAATGATTGATAACATTTTATTAAAATTTATGTATCTTTTTATTATTTTTATTTAATCGAAA
TAATATTTATATTTCACAATATATTGTAACCTTACTTTCATAATTGTCATTTGAATTTTATCAA
AATGATGCAACAGTATATGAAAGAATTAGAACAGGATCCATTTGATCCTGAAGAATTTGTT
GAAAGACTTGCTTGGAGAACACTTAATGATACGATAAAAGACGATGGAAAGACATTTTTCG
ATCCTACAATAGTGCATGAGACATTTTTACAAGCAATTAAAGATTTACAAATTCTACAAGA
ACGTCAACAAAAAAAAATGTGATAAACTTGAAACAGCATTAAAAGATGAAGAAGCAAAAC
ATATACTTGAAATTCTTGAATTACAAGAAAGAAATAAACATTCTATTGATCTCTTTCATCAA
TTAGATGAAAGAATAAATCTTGTGGCTACTAAAGTTTTACATTTAGGAGATCAATTGGAAA
GTGTTAATACACCAAGAGCTAGAGCTGTTGAGGCTCAAAAATTAATGAGACATTTTTCAGA
TTTTTTAAGCCCTGGACCTTTGACAGATCCAATTTTTACAGATAAGTCATCGCTATATGAAG
CAGCAGATGTGATACAAAAACTTCACCTTATTTCACAAGAATTACCATCTGAAAAATTTGA
ACATGCCAAGAAAAAAATTACTGCTAAATATGATGAAATTGAAAGAAATCTTATAGAAGA
ATTTGTTAGAGCACATAATAGGGAAGATGCTAATCGTATGAGAGAATTAGCATCAGTCTTA
ACTCATTTTAAAGGATATTCTCAATGCATAGATGCATTTATAGAACAAAGCCAGATGGGTT
CATTTGGTGGAAAGGATGTTTTTCAGGATGTTATACCTATGTGTACGAAATATCACAAATTA
ATGCAACAAGTATTTACAAATCCTGAACAAGTAAT

155. >MSTRG.3216.1 Gene=MSTRG.3216
GAAGATGGCACTGATTTTTATTCTATTTTTTTGTTCTTCTCTTATTATTTGCTTTCATCTCTATA
TATTCAATTGTAATAATAGAAATGCTTGTTATCACTTACAAATCAAAAAATGAAAAAGATA

TAAAATTTTTTCTTTATAATAAAAAAAAAAAATAATGATTGTTATGTATATATATAAATTTCTA
TATTTATTTACGTCATATAATATAAACATATTAATATTAGGTATGGATTAGCTGGAACTAAA
ACACTGTGGGAGTGAAAATGTGTATGGCGCGGTGGTGATACAACACGTACTGCTTGTACTT
CATGAGGATAACATGGATGCTCATATTGTGCTTGCCGTCCTGTAGGTAAGCCTTCTTTTTCA
AGAGGATGAGACCAATGAGTAGTTTTTGTATTGTGATCTATATAATATTTTCTACCTCTAAG
AGTGAAGTCTACAGACCATCCAGGAGGTGGAGTTTCTTTTTAACATATTTACCAACAACTC
CTTCTTTAATTGTTCTTAAATCCTTATTTTTTCGAGACAACATAATGGAATAAATATTTGATC
GAACGACAAACCAATAAATCTTCGATATTTATAACCTACAGTCACGATATTATATTCGTAA
AACTAATGAAAATATATTCCAAATGTATAATGATGTGGTCAAAAATAACAAATTATTCATT
TTATATATTTGTATAAATTTGTTTTAATAAAGATTCAAAGATTATTAAAAATTTCTTAACTAA
ATTATGTACTTGTCACATGTATGTCTTTTCATAATATGAACGTTCGAAAAAA

156. >MSTRG.3227.1 Gene=MSTRG.3227
TGCGTTCGTAGTCGCTAACAGACTAAAAATCCCCGGGAGTTGTTTATTGATCGTTAAAAATT
CGTGCTATTATTCATCGGTTAAATGCACTAATAATGGAGAGATTTAGTGTAAATGAAAACTC
AATGTATTAAATAAATTGATATCTATAACTGTTTGAACTAAATAAACTTAAAAGTTAAAAA
AAAATTCACAATGATTCGAAATTTCTTTTTGATCGGGCGGTTAATAGACAATTGAGACTGTG
TCGCGTTGTCGAGGCGCAACTCCAACACGTGGGTCGATCAGCTGAGCAACTACGCTCACTC
GAAACTTTTTAATGTGATCAGTATCGATCTAATGAAGAAGGACCAATCATACCATAAGCTA
ATGGAG

157. >MSTRG.3279.6 Gene=MSTRG.3279
CAGAGATAATGAGACGCGGGGATTTGGGGATGGGAATGTATAATAAGTAAAGTTGGGGGG
TGGCAGAGAGGTGAAATGGGGGTTGTATCGATCTGCTGGGGGTGAGTTGGGGGTGGTGCGC
CTCCCAACCCACCTCTCCCTCTTTCTACCTCTCACCCGAGTCTCTCATACCATCCTGCCTCCC
GCCATCCTGAACTAAATGAATGACTGACGCAGGGGTTGGTTGGTGGCAAGTGCAGAGCAG
CAAAGGTCAGAATCTCCTGCAAACACGCAGAATCAGAAACTCGAGAATCCAGATACTACT
AAGGAAATGACTAATAAAGATGGGATCGGGCCCAAAGTGATGAACAAAGGGGGCGGGAA
GGGGGGCTGGCTGAAGGCCCCACTGTCAGAGGCTCAAGAGCCTCATGGTGAGTGAGCAGT
CAATTAAAAGGTGCTCCTTACAGATGAATTTCATTTTATTTTTTCATTATTTTTCTATAAAATT
ATAAAAAAATATATTAATTTGAAAATTTAATGAATTTATAATTAAAAGAAAATAGCAAATA
AATGATTTTCATCAAATATGTGAATGCTGAAACAGTAATAAAAATTAAATATTAATTAATTC
ATCAGTAAGTATTATATAATATGCATCTTTTAGTTGTCTTGTTAGTCTCATATAGAAGCCAGT
GATGATATAAGGCTAATGATATAGCGGCGGGATTTATAAAATTCTTTGTAATCTTGGTGAAT
GTTATAAGTTTTAGCTATTTCACTGTAAGGCCAAAGAGATAATAAATTGGGTTTAATTATGA
CCTCTTCCTTTAAGTTATGTAGAAAGGGGTTTAATCATTAAATATAAAATTTAATAAACCCA
TACATTATACATTAATATATAATTATAATTATTCAATTATTTATCATCTTCTATAATTTTCCTT
TTTTTTTTTTTTTTTTTTTAGAAATCATTGTAATGAATAATTTAATTCAATATGTAAATGCTAG
ATTTGTTCTCTGGAAAAATTGTTATAATTATTATTCTAATAAATAAATCTTCTTCTAAATATTT
AGAAGAAAATCCCGCCGTACAAAATGATAATTCTTTAGTAATCTGAGGCTTCTTTAAACAA
TTTATATTTCTTTGAAAATTAATTTCATTTCTATTATAATAAATAATGTATCACTTAATGTTGC
TTTAATTTTCATTTTTTTGTTACCTGC

158. >MSTRG.3292.1 Gene=MSTRG.3292
TCTATATTCATTGTAGTTTCTATAATATATATAATTTTTTTAAGAAGTTTAATTATATAGTAAT
TAAGCGATCTGTCTTTAAAATTATTTAATAGTACATTAGAAATTTATTAAGAATTATTTTTTC
TATAATATATTGGGAAATCAATTTTTTTTTTATTATTTGAAAGATGGCACTAACCAATATTTT
ACTTCTTAGTCAAATATCTGGATATGTCGTAGCTCTTGTTTTGTCACTTTGTATTATCATACCA
ATGAGTTTACATGAAGATGAATTTAGAGGACATTGTCTGTTATTTTCAACAGGAACTTGGCA
AGAATCAGATGGTCAATTTATAGTAAATTGGGCTTCACAAGCTTATTGAAGCACAACATAA
TGTTGTGGTGCACATGGTCCTTGTCTATATGCTTCATGACAACTATTATTCAATGGAAAATAT
AGAAATCCAGGCTTACAATCACATATCCATGTACTTATATTTCCAACTCCAGAATAAAGAA
GCATATTTTTAGGACATGAGTCTGGTATAAATATTGGATTACGTTCTGTTATCTAATTAAAG
AAAATTTTTTTTTTTCAACAAATTTTTTAAAATTATTTTTACGATTAAATTAATTTTTTTACCG
TCTTAATATTGCC

159. >MSTRG.3297.1 Gene=MSTRG.3297
CAGTAGACTTTGCCGAGTTTTCTTCGGCCGATCGCCGGCTAAACGATTTCTTATGCCGCGAC
ATTGAACGCTTCGTTGTTGAGAAACCGCGTTACATTTTTTTCTTTCTTATATTCTCTGTCTTTT
CTTTTAACAGAGTGCGCTTCCTTATTTGACGAACTTGTACGAAGAACAAATCTTGTGGCGCG
ATTGATTGCACATATAGATATATCTTACGTACGGTGAACGAGGGAATATCGCAAATTCCGT
GACGGTACGGATGGCAAAGGCATTCTGGCGTAGCAATTACCTGCAGGGCGAGGGAGTCAA
GTAGCAAT

160. >MSTRG.3306.3 Gene=MSTRG.3306
CGCCGAGCCGATCAGCAGTACGGTGCTCGCTGGATGCGACTGTTTCGCGTTAGTGATGATC
AAAACGAGTTGTCGTCGTCGTTGTTTGTGGCATTTCGCGAAGTCGAAGAAGCCGTGATCCGT
GATCAAAAGCTCGAGACGGAGAAGAGGCCGAGGACACGCATCGTCTCTCGTTGCCGCGGC
CACGGATGCATATGGAGGTGTCCCCGAACCGGCGCTGTCTGAACCAGCTCAACAGCTGATC
GGAGTACGAAGCGGCGGTGAACGGGGCCACTATTGCACCACCACCCCGCGATTTCGAGAA
ATTCGTTGTATCAATCGGGTTGTTAACGGCCAGACGTTGTTCCCACGAACGAGAGGAGGAG
AAGAAGAGGACGACGACGACGACGACGAGCAGCAGGGGGGAAAAGTGGCGTTTTGACAC
TTGATAAAACGAGGGAAGAAAATCAACGACGACGACGACGACGAAGACGAAGACGACG
AAGACGAAGACGAAGACGACGACGACGACGACGACGACGACGACGACGACGACGAAGA
CGAAGAAGAAGAAAACAAGGGCGAAGTTTCCTTTGCTTGCTTGGACACGCCATCATGGTCA
AAACTTTTCAAGCATACCTGCCCTCGTGTCACCGCACTTACTCGTGCATTCACTGCCGTGCT
CACCTCGCCAACCACGACGAACTCATCTCTAAGGTGAGCCGTGCTAGATCAGGTAACGGAC
AGACTTCTCGCATTCACTTCCGAGCTCGGTACACACAGCATGTATACCGTGATATGCGTATT
ACGTGTATGTATATATATATTGTGCACATATGTATACAGCTAACGTACAGTCTACTGTAATA
AGCTTTTTCTTCCTTTTGATTGCAGTCCTTCCAGGGCAGTCAAGGTCGTGCCTATCTCTTTAAT
TCCGTGGTGAACGTGGGCTGTGGTCCTGCGGAAGAGCGAGTTCTCTTAACTGGCCTTCATGC
TGTGGCTGATATTTATTGCGAATGTTGCAAGACCACCCTTGGGTGGAAATACGAGCATGCG
TTCGAGTCGAGTCAAAAATATAAAGAAGGAAAGTTTATAATCGAGCTTGCTCATATGATCA
AGGAGAATGGATGGGAATAGATTGAGTTGGGGGTAATCTGATAAGCAAAAAAGAAAGAG
AATAACCTCTATAAAAATATATATACGAGGCCCGTTCAAAACTTTTCCTGATATAAGAATA

ATGCCTACAATGTTGTTCTAATTGCAATGTGCGGCCCCCCTCTCCTCTGTCAAACCACCAAC
GAAAGTCTGTGATTTCATTTGTGATTTGTAATCTTAGTAACTTTTTATGTTTCGGACAA

161. >MSTRG.3336.1 Gene=MSTRG.3336
CTCGACTCGAACTGGTGGCGAATGGAGTTAAGAAAGGAATGATACTGTCCTTAGCGATAGC
TTGGAGTAATCGTGGAGCGCCTGTTAAAGATTGAAGTCCAGCACCAAGAGTGGACAGGAA
AGATCCAATCAAGATGACCCATTGATTAGGCCAAGCCATATTCGCTACTACCAGTTTGCCA
CCAATACTTTGGCCAAACTTATCTCGAAGCAGAAGATTGTCCACTGTACCAGCAAAGAGTA
AAACACTGGAGAGATATACTGTTGAGGTTGTCAAAATTGCACAAATAGTACCAATAGGAA
TAGATTTTTGGGCATCTGCTAAGTCACCTGATCTATTGGAGCCAGCCATGATTCCTGTGACT
GAAGGGAAGAAAATACCAATAAGAATGGTGAAGGTAGTGGTAAGATCGACTTGAATTTGA
TTGAAGGAAGAATTGGACATCATGTCAATATCCTTGGGATCTCTTCCATAGGCAATCAATTG
GCCTTCTTCTTGAAAGCTGTCCCATATATTGTCTAAAAATACACCACTAGCCAATCCACGAA
TTCCGTTTATAATAGTCACATCGTTTTCCATATAATAAGGATCACATTTGCTAGTGGTTGTGT
TTCCACAGTAAAGTTGATGAAGAATTCCACTAGTATTCTTATTACATTCGGTAAGTACGTTG
ATATCTTTTAACAATCTCCTTCCAAGAACACACATCTTGAGAGATTCGTTGCCATTAAAGTT
ATAGAATAACCCTACGTAGACAGCCACGATAGAAAGGATGACACAAGCTAGAGCGACCGT
CGCAAATTTGTTGACGAATTTCACGCCAATGAATACGATGGTGCCCATTACCATAAGCAAT
CCAGTACCATACACGCGAAAGTTATTATACATGATGTTAGGGTCTTTTGTAAAGTCTCCAAA
TATACTAAGCGATGGCGCCATGTAAGTCAGAACAATTTCAACAGCACCGATGATATACATA
GCAGCAGCTAGTGTAGTACCCGTATAGAAGAGCATTCCTACGGCACCACCGAATTCTGGAC
CCAAACTTCTGGATATCATGAAGTAAGACCCTCCAGCTGGTACAACGCCATTAGTCGCGAT
GGCACTCATACTGATAGCTGTCAGCATCGTCACGCAACAACAACATAACACGATGAAGAA
TCCCTGAAAAGCTCCAGCGGTGCCAACGACCCAGGTCAAACGAATGAATAGAATCACGCC
AAAGATGTTTTGGATGCAAGGTAGGAAGACACCGATCAAAGTGCCCATTCGTGCACCACCT
TGCACGGGTGGAGGTTTCGCATCCGGGTCGGTCGCAGCTGGAATCGTGTTGCTGTAGTCCG
ACAGGCTGTTGAGCAGCGTGGAAATTCGAGGACGAACTTCCATCTCTTCGCTATAGAGGAA
GAGATTCGTCTCGTATTCCGTATTCTTCTTCTCGGAATTACCGCCGCCCACCATGGAGTCGC
CATCTCCGCCAGCGCCGCCGCCATCGCCGGCTTCCATTCGGGCTGGCGAGCCATCGGGCAT
GGTTTTGCCCTGGGACTTTGGCGTGCCCTCGCTCATGCTTTCGCCACGACTTCGATCCTTCGA
GGCTGGGTCAGTCTCTCCA
CTCCTATTCCACTCCTCTCCTTCTCCTCCTCTTGTTTACCAACTTTATCCTTTTGAAAATCCTTC
GAAGCAAACCCTTTTCCAACACCAACCTTTCTCTCTCTCTTCGTCTATCTTCTTCTTCCCCTCG
TCAAAATTCTATCGAGATCGTTGCAGTTGACACTTTTTGAACTCGTCGTTTCTCGCGAAACA
AATCCCCACTTTTTCTTTCTCACTTTAATTCGATTTTAATCTTTGGCAGAAACGATGACGATC
CTGTCTTTATTAATTCTTCGACTCTCGACGAATATATTTGGAATATATTTTAGACAGTTCGAA
CGAGACACTATTAGACGGGGTGGTTAGAGAAGAACACGGTCGCGCACGGAACCCGGACG
AGTCTCGCGGAGGGTGTGCGTTGGGCTCAACGCTTCAACCTCATCTTCCGCATCCCTCCTTC
CCACACGGCACACGGAGAGAGGCGGCTTGGGTTACGCTCGTCTTGTCGCGGTGTCACAAGG
TCACGGGATCACCGACAGGATCGGGTGGTCCAGATCGAAAGAGAGGAGCCGCACGCACGC
CAGCCGGCTGCTCGCTCGCTCGCTAGGTCGCGCACGCTTCTCTAATTAAACCACGGCGGAA
CGACGATCCCCGGAAGTGCGCGTCCGTGTTCGGGAGGATCGATCGTTCCGATTGCCACGGA
GGCGCATCGACAGGTGAACACCGACCGGGTAAATTTCTCTCCCTTTCCTCCTTCCTTCCTTCC

TTCCTTTAGTTCTCCCGTAAGACTTTCCTCCTCGATTTTCGCTCGAGTTCGATCGAAACGATT
TTCCAAGAAGAGAAGAGAAGAGAAGAGAAGAGAAGAGGAAAGTGTATGTGTCTCGAGCA
ACGAACAATTCGAGCTCGATCCCGCGCAGCGCGTAATATCTCGCGGACGAGCAGTTGCAA
GCTCGTTGGAGCGAAGGATTTACACCGGGGTTGGGATTCTGGGGGTGCAGGTCACCACCCC
TGGACGCTCGTCTTCCTCGTGGACGCTGCGGAGTGCTGGACCACCTTCCTGCCTCCCTCCGC
CTACCCCCTCCCGCCGCCGCTCCCTCTATGATACGTCTCTCCTTCTCCCGTCTCCACCACCCG
TTTGACGGCGCTCCGGTCGGTCGGTGTGTCTGCTGCTCCGCTTCCACGAGACCTGGATTCGT
GGACTCGGCCTCGAAGGAAGGCGGTGCAAGTGTGGAGGAGCACGAGTCGATGGATGGATA
CGCGTCGAACACGGGAGAGCGAGAGCAGCGATCACCTCGTACCGCACCACAAAGACGAC
CACCGCACCGCACTCGGGGAGAGAAACAACCACGACCGGAGGTGCTACCTTCTTCTTCCTC
CTCCTCTTCTTTTTCTTCCTCTTCCTCTTTTTCTTCTTCCTTCTTCTCTTTTCGGAGGAGAAGAG
AAAAGGAAAACGTGTCGACCGAGACGCGGAGTCGAGGAGAGAGGACGTGGCCCGAGAGG
GCTTACTCGAACGAACACTCCCGGCTTTTCCAGGCCTCCTCTCCTTTCCCTCCTCGCTGCGCA
CGCTCTATCCGACTCCCTGTTCCTGCTTCCCTCTCTCTCTCCCTCTCTCTCTCCCCTCCACCCC
GTTCTGTTCTACCCCTCGTTCTTCTCCCTCTCTTCTTCCACCCTTTTCATATCCTCCTTTTATCT
CGCCACTCTCCTCTCCTCTCCTCTTCCCTTTTCTGCCTCCTCCTCCTCCACCCTTCAAACCGGC
ATCTTCTCGCCCTGCGTAGCGTCTACGCTTCTTCCACGTATTTTGCTTCCCTCGTTTTCTCGCG
AGCCAGCGTCCCGGGCGTTCTCAGCTTTTCCACCCCCGGACGGCGCACGCGCCGCTGCATC
CACCCCCGGGATTGTCCCCCTTTCTCTCGCTTCGCTCCTCTCTGTCCTCCTCCTCCTCCTC
CGTTCACTCCGCGCAGCTTCCAAACGTGGGACGAGGATGTGTGTACGTGGATGAG

162. >MSTRG.3344.1 Gene=MSTRG.3344
AATCCTTAACTACACTTCCTATTAGATTAACTAACGGCGCGTGTTAGCACGTATCTCTCTTCT
AATGATTCTGTCCGATCATTTTTCGCATTCTACCGCGCCATTTCTACCGAGTATCGCTATCCA
GAATCGATATCATCGATACTTTTCTACCGCTTACTATTTTTTTTTCTTTTTTTTTTTTTTGTGAG
AAGCGATTTCATCAATGAATCGTTAGTTTCATCGATCGATGGACCATTTTCAATTTGCAATC
TCGTTCATTCTTCGTTTCGAACATAAACTTGCTGCCCCTTTTGTTATCTCGTGATGAATATTTC
TTCCAGTTTTTCGTGACCCGTTAAGAAAAATGTTTCGAGCATTTTGAAATTTAAAAATCATT
CGTGCAAAATTTCTCATCGTTTTTACTTTTAATCAAGCTCGGATCTTTCTCAATGGATCGCGA
ATTGTGAGCGTTGTCTTCCGACTTCTTCGCATTCGGTTAACCTTCGAGTAACCTCATCCAGTA
AGCGTGGTCTTCAAGTTGGATCCAAAGTTTCGATCGAGGATCGTGGAAAGCAGTATCCTGT
AGCAGCTCCTCTTCGTTTTTTAACGATCTCTCGCGAAAATTGGATATCTCGGAAGGTCAGAT
TTTTTTTTCGATCGGCGCTGCGATGGATTAAAAAGAAGAAGAAGAAGAAAAAAAAATA
AAAGGAGGACAAATGGTGGGATCTTCGACGGATCCTGGATCCGTCCCTCCGTCCTTGCTTG
AGCTACGAGTTTTTTGATTAATCGAAGACATCTCGGAGATCCTCGACGCCCCATCCGGACC
GCTCTCTGCCTTCTTTAGTCCTTCGTCGGTTCGAATGAGTTAGAGGTAGAGCGTGCTAACGC
GTCGGGGTGCCACCACGGTGACGGCCAGACGTGGTCCGCATTTGGTGGGGGGCAGCGGTG
CGGCGGTGGCCGGCGGCGGGGTTTCCGGCCTCGCGCAGCCGAGGCCGCTTCCGCCGCGCA
GCTCCCACACTAAATTCGTCAACGAGCGCGGTCTGCTCAGACGCCTCGATCTCTTTTCGGGC
AGCTTAGCACCAGCACCAGAGTCGCCCGAGGTTCTGCCCCCCGAGTGGCTCGAGTCGAGG
GTCCCGTCGAGCTCCATGGCCTGTTGCAGGTTCGTCAAGCTCTTGTATCTGATGTTGAAAAG
GGGGGAGCCCCCCTTGTACCCGGGCGGGGTCTCCTTCAGAGGGGACGCGCTCTTCTGCCTC
ATGAGGGGGCGCCCCCTCTGGGTGCCGCTGTTCTTCACGTAAAGCTTGGCCAGGGGTGGCT

TCTCGGCCCTCGGCTGCGGCGCCTCCTTCACGGCGCGCCTCGTCCTCTTCTCGGCCTGGATCT
TCTTGGTCGGTATCGTCTCGATGGGCGGCGGTTCCGGTCTGTCGTCGAGCAGTGGCGCGACG
GTGACGGTGACGGGGGTGAGGGGCGGGCTGGACGACCCCGCCTGCGGCAGCGGGCTCTCG
TAAGGGGACCTCACGCTCTTCGGCAGCGGGGCGAAACCCCGCACGTAGTCGTAAATCTGAT
CGATCTCGTCGTACTCCTCGGTGCACGCCGAGGGCGGCACGCGATTCTCGTCCCTCGCGTAG
CCGGACGAGGGCGGCTGCTGCTGCTGCTGTTGCTGTTGCTGCTGCTGTTGCTGCTGCTGCTG
CTGGCCGTTGCTGTGATAGTGGTGGTGGTGGTGGTGGTGGGTGGCCGCGTTGCTCCTGT
GGTGAGGAAGGGGCCCGTTGTTGTCCGAGGTGGCGGCCGAGGACCGCCTCAGGAAACCCG
ACCCGCCTCTCGTCTGCCTGGCCGCCTGCTTGCTCTCGCCCAGCCGCCCGTCCAGCACGTGG
GCCCGGTCCGCCACGTCGGCCACCAGCCGCGACGCCCGCTCCACCAGCCTGCCGAACTCCT
GCATCGACCTCAGCGCCTCCTCGTTCCTCGCCTTGACCAGCTTGAGCGGCGCGGCCAGCGG
CAGCGCCACCGCCGCCCCCTCTCGCTGCAACGGCAACGCGAACACGTGCTCCTCCTCGAAC
GTGGACAGCAGCCTGAGCTCGGGCACGAACTGCGACGACGACTTCAACCCCCTCGGCGGC
TGCCCGTGCACCAGCCGCACCGTCAGCGGCAGACGCTTGCTGAGCAACGCGCGGGCCGTG
TGCACGCCGCTTATGTTGTCCTCGCGCGCCACCGCGCTGAAACGGCCCCGCTGATCCATCCC
GAGCAGCACGCTCTCCCCCCGACAGTCCACGCAACGGAGGAATCTGCCGCGGCCGGGCAG
AGTCACCTCGCCCGCGGTGACGATCGTCTCGCCCGCGGCCAGGGTCCTGGTCCCCTCCGGG
ACCACCAGCCCCGACTCGTCGTCCACCCTGCACGCTATGCCGCGCACCGTCTCCCGCACCA
GGGCGCCCTCCTCGGGGCAACGGCGGGACAGCTCGTTCACCGACTCTATCCCGCGCACCGC
CCTCCCCTCCTCGCTCAGTATCTCGAAGTAGCCGGCGTACGAGTCGGGGATGGCGAGCCTC
GGCCCCACCCCGACCACCCTCCTCCCCTCCTTGATCTTCACCGCCTGGGCGACGATCTTGCG
CCGCCTGCCGGCCGACACGAGGAGGGCAGTGCTCTGCAGGCTGGGCGCCGGCAGGGTGGG
CACCCCCAGCCCCCCGTACTGGCCCTTCGTGATCTTGATCACCGCGGGCAGACGATACTTG
GAGACGAACTCGCGGAGGTAGGTCGCCTCCTCGGCCCACCGCGCCGCCGCAACCACGATC
TGACCGTCGGTCGCGGCCATCGCCGCGCTACAACGCCGCCTCTCTCGCGTCCGATCCCTCCG
CCTCCGGCCGATAGGGTGCCGCTACTGCCGATCCGACGCCGCGCCTCTATGCTCCATTTGTT
GCCTCTCCTCCCCCTGTGTGCCTCGATCCGACAATATTGGCGCGCTCTCCTCCTTTCGTGGCC
GTGATCCTTTTCGATTTCCTATCGCCTCCTGCTCCCACTGCCTCCGATCCAGAACGTGGTGAA
CGACGGTCGCCGTGAGGCACGTCCGCTTGCAAACTAATTTTTGTTTTCCCCACTGGTAACTC
CACTATGGCACTGTATTACTGTATGTGTACTATACGTATCTAACTTGTACAAGTGTACGTAG
ATGTACCGGGCGTATGTTCCCGAGACCACGAAAAGGGGATGCGTACGCGAGCCGAGGCGA
CAAATACGAGAAAATCGCGTGAAACCACCGTTTCGTATTTCAACGTGCGCCGGACGGAAA
AATGTCGAGAAAAGACGAAATAGCCGGCGGAGAGAAAAGGAGAGTGACGACTGGCCCTG
CTCTGACACAGATCACTGACCAATTCCGCTGGAGGACTGACGCGATACTGACTCGACATCG
CATCTGGCGAGGAGCGCGACAACCGGC

163. >MSTRG.3344.2 Gene=MSTRG.3344

ATCGGCGCTGCGATGGATTAAAAAGAAGAAGAAGAAGAAAAAAAATAAAAGGAGG
ACAAATGGTGGGATCTTCGACGGATCCTGGATCCGTCCCTCCGTCCTTGCTTGAGCTACGAG
TTTTTTGATTAATCGAAGACATCTCGGAGATCCTCGACGCCCCATCCGGACCGCTCTCTGCC
TTCTTTAGTCCTTCGTCGGTTCGAATGAGTTAGAGGTAGAGCGTGCTAACGCGTCGGGGTGC
CACCACGGTGACGGCCAGACGTGGTCCGCATTTGGTGGGGGGCAGCGGTGCGGCGGTGGC
CGGCGGCGGGGTTTCCGGCCTCGCGCAGCCGAGGCCGCTTCCGCCGCGCAGCTCCCACACT

AAATTCGTCAACGAGCGCGGTCTGCTCAGACGCCTCGATCTCTTTTCGGGCAGCTTAGCACC
AGCACCAGAGTCGCCCGAGGTTCTGCCCCCCGAGTGGCTCGAGTCGAGGGTCCCGTCGAGC
TCCATGGCCTGTTGCAGGTTCGTCAAGCTCTTGTATCTGATGTTGAAAAGGGGGGAGCCCCC
CTTGTACCCGGGCGGGGTCTCCTTCAGAGGGGACGCGCTCTTCTGCCTCATGAGGGGGCGC
CCCCTCTGGGTGCCGCTGTTCTTCACGTAAAGCTTGGCCAGGGGTGGCTTCTCGGCCCTCGG
CTGCGGCGCCTCCTTCACGGCGCGCCTCGTCCTCTTCTCGGCCTGGATCTTCTTGGTCGGTAT
CGTCTCGATGGGCGGCGGTTCCGGTCTGTCGTCGAGCAGTGGCGCGACGGTGACGGTGACG
GGGGTGAGGGGCGGGCTGGACGACCCCGCCTGCGGCAGCGGGCTCTCGTAAGGGGACCTC
ACGCTCTTCGGCAGCGGGGCGAAACCCCGCACGTAGTCGTAAATCTGATCGATCTCGTCGT
ACTCCTCGGTGCACGCCGAGGGCGGCACGCGATTCTCGTCCCTCGCGTAGCCGGACGAGGG
CGGCTGCTGCTGCTGCTGTTGCTGTTGCTGCTGCTGTTGCTGCTGCTGCTGCTGGCCGTTGCT
GTGATAGTGGTGGTGGTGGTGGTGGTGGTGGGTGGCCGCGTTGCTCCTGTGGTGAGGAAGG
GGCCCGTTGTTGTCCGAGGTGGCGGCCGAGGACCGCCTCAGGAAACCCGACCCGCCTCTCG
TCTGCCTGGCCGCCTGCTTGCTCTCGCCCAGCCGCCCGTCCAGCACGTGGGCCCGGTCCGCC
ACGTCGGCCACCAGCCGCGACGCCCGCTCCACCAGCCTGCCGAACTCCTGCATCGACCTCA
GCGCCTCCTCGTTCCTCGCCTTGACCAGCTTGAGCGGCGCGGCCAGCGGCAGCGCCACCGC
CGCCCCCTCTCGCTGCAACGGCAACGCGAACACGTGCTCCTCCTCGAACGTGGACAGCAGC
CTGAGCTCGGGCACGAACTGCGACGACGACTTCAACCCCCTCGGCGGCTGCCCGTGCACCA
GCCGCACCGTCAGCGGCAGACGCTTGCTGAGCAACGCGCGGGCCGTGTGCACGCCGCTTAT
GTTGTCCTCGCGCGCCACCGCGCTGAAACGGCCCCGCTGATCCATCCCGAGCAGCACGCTC
TCCCCCCGACAGTCCACGCAACGGAGGAATCTGCCGCGGCCGGGCAGAGTCACCTCGCCC
GCGGTGACGATCGTCTCGCCCGCGGCCAGGGTCCTGGTCCCCTCCGGGACCACCAGCCCCG
ACTCGTCGTCCACCCTGCACGCTATGCCGCGCACCGTCTCCCGCACCAGGGCGCCCTCCTC
GGGGCAACGGCGGGACAGCTCGTTCACCGACTCTATCCCGCGCACCGCCCTCCCCTCCTCG
CTCAGTATCTCGAAGTAGCCGGCGTACGAGTCGGGGATGGCGAGCCTCGGCCCCACCCCG
ACCACCCTCCTCCCCTCCTTGATCTTCACCGCCTGGGCGACGATCTTGCGCCGCCTGCCGGC
CGACACGAGGAGGGCAGTGCTCTGCAGGCTGGGCGCCGGCAGGGTGGGCACCCCCAGCCC
CCCGTACTGGCCCTTCGTGATCTTGATCACCGCGGGCAGACGATACTTGGAGACGAACTCG
CGGAGGTAGGTCGCCTCCTCGGCCCACCGCGCCGCCGCAACCACGATCTGACCGTCGGTCG
CGGCCATCGCCGCGCTACAACGCCGCCTCTCTCGCGTCCGATCCCTCCGCCTCCGGCCGAT
AGGGTGCCGCTACTGCCGATCCGACGCCGCGCCTCTATGCTCCATTTGTTGCCTCTCCTCCC
CCTGTGTGCCTCGATCCGACAATATTGGCGCGCTCTCCTCCTTTCGTGGCCGTGATCCTTTTC
GATTTCCTATCGCCTCCTAATCGCACCGGTGTTCCATCGTCCACGGCATTGTCTCTTTTCCTTT
CCCGTCCCCTTCACCCTGTGAACCGTTCTCTCCTCGGTGGAAGCCCGGTCCTACGGTCGAGT
TCAACGACGCGTGAACGCAAACTTTACTCGTTCCATTACATTTTCTACTTGGATAATCCGGT
ACAGTTGGAGAGCCTGCGCGCCGACGCGCGGTGTCACGTTCCAGGCAGACCGCTCGCGCG
GACGAATTTCGAACAATGCGAGTTTATAGCAATTGGCAGATGGACGATTGGAATTTCGTTA
TGCTTTCCAGAAAAAAAAACGAAAATTTTAATAAGATTCTCTCCGTCGAAGATAGAAAGTA
ACTAGGAACTTGAATATAAAATTTCCAATTTGGCAATTCTTCCTCGAAAATTCGTAAAAAG
AAAAAAGAAAAATAACTAACCTTGATTAGATCCTCGTAAAAATTCTCATCTTATAAGAATT
CATTCTTAATACTTGGACTTGGAGGGGCGCTCGGGATCTAAGGGAAACGAGAGACATTCCT

164. >MSTRG.3382.1 Gene=MSTRG.3382

CGGCAAAAATTAGTGGAGCTTGGTTGGGATGTTTCGCCACATCCACCGTATAGTCCTGACCT
TGCACCATCCGATTACTTTTCGTTTCGATCTTTACAAAACTCCTCGAATGGTAAAAATTTCA
ATAACGATGATGATATCAAATCGTACTTGATTCAGTTTTTTGCTAATAAAAACTACAAGTTT
TATGAATTATGCTGCCTGAAAGATGGCAAAAGGTCATTGATTTAGTTCCATGAAAAAATTG
TCTTTGATTTTCTAAAAAAAATCCGCAATTACTTAATTCCCAACCCAATATATATAATCGGG
AATAAGCGAGAGATAGAAAGAATCCTTGACAATATTTGATAAGTGACGTTGAAATGGAGG
AGGAGAAAAAGAGATTATAACCACGCTCGAGGAACGAAATTATCGATGAACAAGCTCGTT
GTAAGATAATATGAATCTCTAATATGAATCTGGATTATATAGTTTAACTTTTCACGTTTTCAC
GATACTCGTTCCTCGAGCGTGGTTCTTCTCTCTGGATTCGATGATTTTCTCATCGAATCGAAA
TAATAGAATAGGAAAAGAGAAAAAATTTGTTAAAAATCTCAAGTAATTGGAATACGATCAG
GGAAGAAAATTAAGCGCTGTTTGGGGATAAATTTTCTACGAAAAGAAAAAAGAAAAAAA
AAAGACGATAGAATAGTGATTGTCAAAGTGTCGCCTTCGATTCACATATCATTTGTATTGGA
GTTTTATTTATCGTTGGTTCGAAGTTGATCGGCGAGGGTTAAAAGTAGCTGGTAAGCGTAAA
ACCGGCGTTTCAATTTAGCGAGAATGTATCGATTAGAAGGGCGAGGGAACGGACGGCGGA
GAGGAAAATTAGCGGAAAATTGAGCCCGTGGCATATCACGTGGGGTGATAATTCTCGCCTA
GGACCAGACACTGTGCGTCGTTGTGTACATATAAAATGCGTTCAATTAGTTTTATCGGTCCT
CGTTCGAAAGAAAGGGAGAAATTGATTAAGCGCTACTCCTAGTATATCAGAAATTTAAAA
AACGTATCCGAGATTATGATGATTCGAGATCATTGAAATTTAAATCGAATCGAAAGTTGGA
CTTTCTCTCGGTCGAATCGTTTATCGATTCGAAAAGGAGGCAGCCCGGCGCGAAATACAGT
TGTACGCACACGACAGTGAGGAATAGAAGGGATGTCGTAAGGGTACGCTGATATATCTCCT
CGAATTTTCAGAATTCATGGAAATTCATGTACCTTTATAGAATCCTCTGTATCTACCCGTAC
GTAGGATATTCGATTTAAGGAAAATGAGAGAATAAATGTCGTACGTTGTATATTTGTCCC

165. >MSTRG.3453.2 Gene=MSTRG.3453
TACGGTAAATTTATACAAGAGAAGAATACGTTACGTCACGTTATTTTCAATGCCGTTGCTAG
ATTAGACAGAGATACTACGCCATGTAACAAGGCTTCCATCGTTAAGTCAACGTTATATCGT
AATTGTGTATGTAATATATATACTTCGAATTGTGTAGCAAATATTGGATAAATAACGAAATT
TAATATATAATATATAAATATATATAAAATAAATAATAAAATTTTGCAAAATATGGCAAAA
CAAATAAAAATGGTACTAATAGATCTTAGTGGTACTCTTCATATTGATAATACAGTGATTCC
AGGTGCAGTGGAAGCTTTAAACAGCTTCGAAATGCTAATATACCATATAAATTTGTCACAA
ATACAAGTAAAGAATCTAGCAACTTACTATATAATCGATTAACAAAATTAGGTTTTACTGTT
AAAAAGGAAGAAATTTTTAGTAGTTTAATTGCAACAAGAAAACTGATCATTTCGAAAAAAT
TAAATCCAATGTTATTGATTGATCCTCATGCTAATGAAGATTTTGAAGATTTGGTAAAAACA
AATGAAACAATGAATGCTGTAGTAATTGGATTGGCACCGGATAAATTTCATTACGAAGAAT
TGACAAAAGCATTCAGGTTATTATTAGATGGTGCATCTCTCATAGCAATTCAAAAAGCTCG
ATATTATAAAAGATCAGATGGTTTAGCTTTAGGACCAGGGGCATTTGTTGCTGGTTTAGAAT
ATTCTGCTAATGTGAAAGCAGAAGTCATTGGAAAACCTACTGCAGAGTTTTTTAAAGCTGCT
TTAGGAAACATATCTCCCGAAGAAGCAATTATGATCGGCGATGATGTTAAAGATGATGTAG
CTGGTGCCCAAGCTATCGGTATAAGAGGAATTCTAGTACAAACTGGAAAATATCGAGATGG
AGATGAAAATACAATTACACCACTGCCTACAAAA

166. >MSTRG.3457.9 Gene=MSTRG.3457

GTCGGATCTCGAGCCGTGCAGGTCGTTTAAGAGTTTTTGTAGTTTGCGTTCGATTACCGAGA
CGCGTGCTACAAACCGCGTGGTTATCACGTTATCAATCAAAGTTTCTGGTGTGTTTCGTTCA
GTGCTGTTTCATGTTATTTTTCATCGTCGTTGTGTGAACGTTCCATTGAATTTTTCGACAGGCA
ACAAACAGTGGTTGCAATTTTTGGGGTGTACAACGTGACAGACAGGAAGATCGTTGGATAA
CCAGACTACCGACGCCACCAGCACGGTGATCCTCGTAATCTTATCAGCCATCGGTCGAGCA
ACGCGCCAGATCCTCGAGCTGCATCGTGACTCTTCTCCCCTCCCCCCCTTCTTGGACCGAAG
GCCAAGTTACACGCCGGATACATCGCGAGGCAATTAAGGAGATTCACGGGAAACGCAGAT
GGAAATTGGAAGGGTATCGAGGTTGGCCGGCGTCGTTCTGGCCCTTATCTCGATCACGACC
ACGTGGTCCCGGCATGGTTGCTGCGTCGCGGAACTTTACACCGCGTTAGCGGACATGGAGG
AGCTGCTGGAGACGGAGGCGGTGCTCATCGACACTCTGAACGGGTACATCAAGGCGCAGG
AGGAACGTCTCGCCACTCTGAGAAAAATCTATCGATCAACGAATCCGTTACGATTCTTCAC
GCGTCGCTCTTGTCGAATAATTAATGAATAAGGATGCGATATTCGTCCAAAACGTTTATTTT
CTACGAGAAGTGATAGCGAGAAAAATGTCGAGG

167. >MSTRG.3461.1 Gene=MSTRG.3461
GTGATAGTAATCACGATTGTGATAAGATTGTCTCCCAAGTTCGAAACAGTCGCTGGCGGTT
AATCCTGTGCTGTATTGGACCCCGTTCAACACGCCCCTGGCGACCTGTGCAGTCTCGAGTTT
GTACGTGTCCTGTAACCTCATCAAAGCGACGGCCGCCCCATTAAGATCCTCGTCAGTTGGG
AATTTAAGGTCGCTCCTCGAGCTGGTTATATTCGCCACGAAAGACTTGCCCACGTCCTGGGT
GATCAGCTCCTCGACCCGTTTCCAATCCGTGGTC
AGCCGTTTCACCAATAGATAAGCGTTGATCGGATTCGACAGGTACTGCTGAATGTTTCTCGA
CGCCTCCTCGTGCTCTCTCGCGTAATCCTCCACGTTTTTTCTCAGAGTGGCGAGACGTTCCTC
CTGCGCCTTGATGTACCCGTTCAGAGTGTCGATGAGCACCGCCTCCGTCTCCAGCAGCTCCT
CCATGTCCGCTAACGCGGTGTAAAGTTCCGCGACGCAGCAACCATGCCGGGACCACGTGGT
CGTGATCGAGATAAGGGCCAGAACGACGCCGGCCAACCTCGATACCCTTCCAATTTCCATC
TGCGTTTCCCGTGAATCTCCTTAATTGCCTCGCGATGTATCCGGCGTGTAACTTGGCCTTCGG
TCCAAGAAGGGGGGGAGGGGAGAAGAGTCACGATGCAGCTCGAGGATCTGGCGCGTTGCT
CGACCGATGGCTGATAAGATTACGAGGATCACCGTGCTGGTGGCGTCGGTAGTCTGGTTAT
CCAACGATCTTCCTGTCTGTCACGTTGTACACCCCAAAAATTGCAACCACTGTTTGTTGCCT
GTCGAAAAATTCAATGGAACGTTCACACAACGACGATGAAAAATAACATGAAACAGCACT
GAACGAAACACACCAGAAACTTTGATTGATAACGTGATAACCACGCGGTTTGTAGCACGC
GTCTCGGTAATCGAACGCAAACTACAAAAACTCTTAAACGACCTGCACGGCTCGAGATCCG

168. >MSTRG.3471.1 Gene=MSTRG.3471
AACTTATACAAGTACGTGGCTTTATGACTATTTTCCTTTTGCTGACCGCAAAGTAGAGGTGG
TAAACGATAAAGGTAATAACATAGAGGTTGACAAGTAGGTAAAGAAGGAAAGAAAATAT
TAAAGATTTAAAAGGCTAAACTCTTATCTTACATCAGTTAGACGTGTTTTATCTTCGAATTG
AATCTTATTCAGAGGAAGAACATATTGTGAAACAGGATCTTGAATAAACAAAAATGTATA
AGAAAGGAACAATTCTCGTGTTAGCGTATTTATTAATTGCCACAGCAGTCTGTCAACTATCT
TATAAAGAAGGTCATTGTCCATTAAGAAATAGTGTTTCAAAATGCATTCCTAGATGTGTTTC
CGATTACCAATGTTCTTTTAATGAAAAATGTTGTCCAAATAAATGTGGAAGTGAATCTTGTG
TTCAAGCAAGTCCTATTAATACTGGCAATGGATATAAAGGATCGAACGATGATGTTTATTG
CGCTGGAGTAAAATGTGGGCCATATGAAAAATGTCAATTTGATCGTAAAACAAAACGAGA

GAAATGTGTTCGTACATAGATGATTTATTTAAATGTAATAATAATATTATATTAATAATATTT
TTTTTATAAAATAAATAATTTCTTTTTTGTTTAATCAATTTATTTGTATATAAATTTATTTCT
CAATAATAGAGAAATTTCAAATTCAAATTGCGATTAAGAGCGATTAAATCGAGTTACGACC
AGTAGCGATAGAAAATCTGTTGAAATAAGTCGACAATCGACATACAGATTTTAATCGATAT
TTTTCGATAACCTTTTTATTGTTTCATAAGGAGTGCATGCCGAACGAAGTACTACTTGTTTGT
TATTAATATTTATAAAATAAGTACAATATAAGATAATTACATCGTTCTAAAAATTTTTCAAT
AAGATGGCATTTGATCAATCTTAAATTATCACAATATATAAAATCATTTCATTACACATTTT
CTTAATATTATATAAAAGAGGTTAGGCTTTATGCCGATATAATACAATGGCAATAGAATTTT
TAGTAAATCGATTCAAAGCACTCGTTGTATTTCTTTTGGGAGTATTTCGGCGAGCAATGTGTT
GTTTTCGACGTCGAAGAAGATCATCCTGTGATTCTATTCCCTTATCTGCTATTGGCGTAGTAC
CGAACACCTTGAACATAACACAACCATAATTACTGAAGATGCTTGCTCAAGGAATTAGAGC
ACTGGGATCAATGGGAGGAAAATCCTGTCGTGATCGTGCCAGATAGGCCAGTTGACACAAT
ACAAGCAAAGATTGAACAATATAGACAGCAAGCATCTAAACCACCAGAATCTACGGAAG
AGCAACAACCAGATTTTTTTGAGAATATGACACCAAAGATCACTACACAGACAAAAATTTT
AATTAAAGATAAAAGCTTAGAGAACACATCCTGGAATTCAACAAAATTTGCTGTGATCAAT
GATCCTATACCATCTAATGAATTAGGAGAATGGGAAGACAATGCTGTTGGCTGGGAGGAA
GAAACAACAAAAGAATTTGGAG

169. >MSTRG.3480.1 Gene=MSTRG.3480
CCCTTCCTGTTCCGCTCCACTCCGCCACTGGTCCGTCCCAAAGCCAACGAATACCCGTCGAC
GCCGAAAGTGCTTCACTGGCATCGACTATGAGCCTGGTCATCGTCCTCCGTTTGTCTATTAT
GGACGCACGCGTGTTGTTTCGAGCATTTTCGTCAACTCGATTCGCGTCAGAGGAAGTTGTTG
ATTGAACTATTTTCTCACGATCATCGTCGAGCGAAGGCAAAAGTGTGGCCGGAGGATTGTA
AATCGCTAATCGACCTTCTGCTTATTAATTGCACCGATTATTCCGTACAGAACTTTTTTTTCT
CCCTTAACTTCTTACTCTTTGATCGTAAAC

170. >MSTRG.3501.1 Gene=MSTRG.3501
CTCGTCGAGAATGGCCATCGTAAAACAGGAATATGGATATAATAATCTCCGCGTATATTTTT
CGCATCGGCATTCCCGACCGAATATTTTTCAATTCCCTCCGGACACAGAATTTTCCGCTCTA
ACACTAACTAAATTACAATTCTTCTCTTCTCTTCGTCCTCCTACTTTGCAAGTAGACGTCCTC
GTTCCTCGTTCCTTCGACCTCTTCCCATCAATTCTCTTCATTCTCGATCGTTCGAACACAGGA
CGCGATCCCATCGTGCACTTTCGCTTCCGGCTATTCAACACCGAGCCACTGAGAGCTAGCTA
CTGAGCAACACTCTGGTTTCTTTCTCTCTATGCGTGGAACGATCATCATCATCATCATCCTCC
TCTGGGAAAAAAATATCTTCGTCGTATGCTCGATCGTCCAAAAATGGATCACTCTCTCTCTC
TCTCACTCTCTCTCTCTTTCTCTCAAACTTGGAATAAAAAAAACACAGTCTTGATTCGTTCGT
TCGTTCGCTCGCTCGATCGATCGAACAGATCGACCGATCGATCGGCACGCATTCGATATTTC
TTCCGAGTCACGAACAACGAAACAGATTAATCCCCCGTTATATATATATATATATATATATA
TAGTTTCCCGGACAGTTTTGCTCGCTTTGTCCCGGTCAGGCGGCACAGATATACAAACTATA
AAGCAGATTTCGTAAGATTCGGAATCGGATCGATCCATCCCTCGTGTCGAGTAGGATCATC
CTATTCCCCGACGCGGGCACTAAAAATCGCTCTCTCTCTCTCTTGCTCTCTCTCTCGCTCTCT
CGCTCTCTCTCTCTCTACGTGTTATCCTAGTAGATCGCGGCTACGAATCGTTCCCTTCTTTCC
ACGTCCACGCTCCGCAACTTAAGCGATCAACAACGGTGCACAACAGCCAATCGCCATCGA
AACGATGGACGAAAGCAGGATGATGCTCGATCCAGCGCCGGCCGTCACCGGATTCTCGTG

ATTCGTCTTCAGCATTGGATCAGGTGTGGTGGGTCCCATTCCGGATCCAGACGCCTCCTCGT
CGTCGTGGTCGTAACCGTGCACGTCCTCGTCGTCGTTATCTATATCCTCGTCTTTGTTTTTCTT
ATTCCATATAGGCCGGTCGCCGCTACCAGAACCGTCCAATGCCTCGTCACCGAGCAACGTT
CCGCTGTCGGGCGCAGACGCCAACTGAGACTGCAATATCTGATTAATGTGCCTCAATTGGT
CAATGAGCACGCTGGTGTTCGCGTTGCCGTAGGAAGCCGTCGTCGGCGACATCGTCGTCAC
GGTGAACTCCGGGTTGTACCTCTGCGCCGTCAAACTCGAGTCCGCCACTGTCTTCGTGTACT
CCCCGACTCGTTCGCCGTTCCAGCACCGTTTGTCCGGATACTCCTCGCAGATCGTGTCGGCC
AGGGTGCCGTAGAAGGTGCGAGACCTGACCACGCTTGCCAGGAAATTGCCGAGCTGCACG
TGCAACTGGGACGGCAAGGTCCGGCTGGGCGGCGCAGCGGCAGCTGCGTGCATCTCGATC
CCGCCCGACGACGAGGACGAGGACGACGATTTCCCCGTTCCACCGGCTGCGTCCGCGACG
CTCCTCTCGCTCGACGGCGATGGCTCCAGCTCCGAGCGGCCGCACACCTTCCTCACCTTTTC
CTCGAGCGCCGGTCCGTCCGTCATTGCGTGCATAATGGCGTCCGAAATGCGGGTGTCCAAT
TGTCTGACAGCCCTCTCGGCATTCAGGCCCAACGGATCGTTACGACCGTCGACCGCCACCA
CGAGCCTCTCCACTGTTTCCACGTAACCGGACCAAGCCAGGTCCAGCTCGGATGCTGGCTG
CGTCAGGCAACCTCTCATGACATTGGTGCAGAATCCACTGCAGGGCCTGACGGAGGGACC
GATGCCCTTGCACCTTGGACAATAGGTCATCCTGAGGAGGGCGGCGTAGCAGGCCTCCTGC
TGGGGGCTTGTGCCGGAGAACAGCACGCTGTCGGTCCTGTCCAGCACCGTTTTGCCGAGTG
TCAACGCCTGGACCAGGACCCGGGTCGCCTCCAGGCTTTTCGAGACCGATTTCGCGACCTG
GACCGGTATGTCGCCGAACGGTTGGATCTGTTCCATCGTCTCGTACAGGCACGTCTTGAACT
TCTCCGTGAAATCTTGCTCGTATTGGCGGGGATTCACGGCGCCGTGATACGCGATCGGGAA
CAATTTGGCGAAGAACTCGATGAACCTTTCTTGAAGCATGTCCCGCGTCAACGGTTGCTGC
AAACTGTCCGGCGTGTTGCTCGGTGACACGTAATCGACCATCGCCTGGTACAGGGCTTTTAT
CGAGGGCCTGCTCGATACCGCCATCGTCCGATACACTTGATCGAACAGTGTTAAAGTTTTGT
TCTCCGACTGTCTCGCGAGCATCGTCACCGTTTCCCTTAACGCGTCCGCCGTTGTAGCCAAA
AGGCCTTGCAAACTTCTGCTGTGATGATGGATCAAGTCGTGGAAATCTGCCCTGGCTTGATG
TCTTAACTGTTCTTCGGTGTCCCGGTCGCAGCACATGCCGTTACAAGTTGTTTTTGCGATGTC
TTCTGCTGTAGCTTGCTTCCTATCAACCAAGATCGTGATATTCTTGCTCTCGAAAAACGGCTT
AACACGTTCACAATCAACGGACACGAAATCGCTATCATTTTTCGGATGTTCGGCAAAACGT
GGCGATCGAGATGACACGAGATCGGTACAACCGCATAATAATATCACGATCGATACAGGT
GCCAGAATCGCACGACGATACCTCTGAATTATTTTCATTTTGGCAAAATGGCCAAATCGGC
GAGATGAATCGGTAAAGAAAAGCAAAAGCGAAAGAAAGAGAAACCGGGAGCGGAAACT
GTGCGAAAAAGATTGGTGGTAAGTTCCGTAAATCGTGCTCTGAAGCGCAAGAAGAACAGA
GAGAAAGAGACGCAGCACGAGCAACGAGACGATTCAATTCCTCTTTCTCCTCCTTCGTTGT
CGCGTATTCGGTACGATCGTTCGAAGAGTCATCAACCGCGGGACTAGACGCGGCTTCCTTC
CCCTTTCCTCTTCGTTCTTCGAATCGGCCGCGCTGTCAAACGGCACAGCAACAACCACGGGT
GTTACTGCAAGATCTCGAGAAGCGCGAGACGTGCATTTGGCGTGATCGCGATGGACGGAC
AGACGAGACGATGGCTATAGTAATCACGACATGCACTGTCGAGACCGCGCAACACCGTGT
TATGACCCGCACACATGCACACGCGACCACTCCGGGTACACCACAATAATAAGCACACAC
ATGCACGCACGTTTAACCGCACGCAAGCATGCATGCATGCACAACACGACACGCGTTCCA
CGAGTAGCTCGATGCGACTGAAGTGCCGAGAGCGGCACCGCGTATTCACAATT

171. >MSTRG.3503.1 Gene=MSTRG.3503

CCACGAGGAGGTTGCTGCAAATGGTGACTCACCCTAACTCCACGGGGCCCATCCACCCTTC
CTTTGCAAATTTTAAACTGCGGTAGACATCATCGCAGTACATCAACCGTTCATTCCAATTGG
AACAGACGGAAGAGAAAAAAATGGACCTACCCGAAAAAGAGCTAGACGTGCTAATCATC
GCCTATGGTCTTTTGAAGCTAAAACACCCTTCACCAATTCCTAGCTAAACTTATAAACTAAG
CTAAGCCCTAGCTAAATTAGATTTCTGATAGGCAGGATTTATAAACCGTACAATCGTTTGAT
TTATATGTTATTAAATGGCATACTTCTTAATAAAGAAGGGCCTATGAAAAACGGAACGGT
ATTAACTAAAAAAAAACAATGCAATTCTACTATTCTAAAACGTGCTCATTTGGTAACATTG
CATGTCTCGTCCAATACGTCGTCACGAGGATAAATGGAATTCCTCTACTCCCGTCATCACGA
TCATAGAGGAAGGTATAACGAAACAGAATATAAACATTTAAGTTACTATTCCATTTACGCA
TTGCGAATATTGCAGACGAAACAAAGCTTCGAAACTGCGTTTCTTTGCGATGCAAAGAAAC
TATGTTTCTTTTGCTTCCGTTGACATCTACAATCGCAAAAGAATCCTAATGGGTTACAAATTT
TACGCGTGTGATCTCCTTTAACCAGAAAAAAGGTACTTAAGGAAACGACAGAATCACCGA
ATCTATCAAATCACCGTGTAAAATGGGTCTTGTAAGAGACATGCACGTAACGGGCATCCTC
TAATTCAAAAAAATCAGATTATTCCCAAACGCAGCATAAATTATATCTTCTTGATCGTAAA
GCTTTAGAAAAAGATACAAAAATCTTGTTCGAATCGACTATGTCAATAGAACATGAAATTG
GTAGCTATTTCCATTTAACTTTACTTTCATTAAGTATATAATTTTCATCTTACAGACGTAGAC
AGAAGGAAAGAAAAAAGGGAAAGGGGACTTACGATTGTCCGAGATTACAAAGAAACCAA
ACTGCACCACGCGGAAGACGCGGAAGATCTGAAAAACACTAACATATGAAACCAAACGA
TTACACAACAGTCTAGCTTGAACGTTCGCGGAGTGAAGTTCTCTTCTCGTTTTGTTCTTAATG
AACTTTCAATTTTATTCTAAATTTCGATCTTGTAATCCATGTTTTCATCATTCTTTCCATTTCTC
AAAATTTTACTTTAATTCGTAATATCATGTTCAAGCTAGACTGTTGTGTAATCGTTTGGTTTC
ATATGTTAGTGTTTTTCAGATCTTCCGCGTCTTCCGCGTGGTGCAGTTTGGTTTCTTTGTAATC
TCGGACAATCGTAAGTCCCCTTTCCCTTTTTTCTTTCCTTCTGTCTACGTCTGTAAGATGAAA
ATTATATACTTAATGAAAGTAAAGTTAAATGGAAATAGCTACCAATTTCATGTTCTATTGAC
ATAGTCGATTCGAACAAGATTTTTGTATCTTTTTCTAAAGCTTTACGATCAAGAAGATATAA
TTTATGCTGCGTTTGGGAATAATCTGATTTTTTTGAATTAGAGGATGCCCGTTACGTGCATGT
CTCTTACAAGACCCATTTTACACGGTGATTTGATAGATTCGGTGATTCTGTCGTTTCCTTAAG
TACCTTTTTTCTGGTTAAAGGAGATCACACGCGTAAAATTTGTAACCCATCAGGATTCTTTT
GCGACTGTAGATGTGAACGGAAGCAAAAGAAACATAGTTTCTCTGCATCGCAAAGAAACG
CAGTTTCGAAGCTTTGTTTATCTGCAATATTCGCAATACATAAATAGAATAGTAACTTAAA
TGTTTATATTCTATTTCATTATACCTTCCTCTATGATCGTGATGACGGGAGTAGAGGAATTCC
ATTTATCCTCGTGACGACGTATTGGACGAGACATGCAATGTTACCAAATGAGCATGTTTTAG
GATTATAGGATTGTGTTGTTTTCTTTTAGCTAATACCGTTCCGTTTTTCACAGGCCCTTCTTTT
ATTAAGAAGTATGCCATCTAATAACATATAAATCAAACGATTGTACGGTTTATAAATCCTG
CCTATCAGAAATCTAATTTAGCTAGGGCTTAGCTTAGTTTATAAGTTTAGCTAGGGATTGGT
GAAGGGTGTTTTAGCTTCAAAAGACCATAGGCGATGATTAGCACGTCTAGCTCTTTTTCGGG
TAGGTCCATTTTTTTCTCTTCCGTCTGTTCCAATTGGAATGAACGGTTGATGTACTGCGATGA
TGTCTACCGCAGTTTAAAATTTGCAAAGGAAGGGTGGATGGGCCCCGTGGAGTTAGGGCGA
GTCACCATTTGCAGCAACCTCCTCGTGGTGTGACTTGGGAAATTTGTAT

172. >MSTRG.3504.1 Gene=MSTRG.3504

GCCCTAACTCCACGGGGCCCATCCACCCTTCCTTTGCAAATTTTAAACTGCGGTAGACATCA
TCGCAGTACATCAACCGTTCATTCCAATTGGAACAGACGGAAGAGAAAAAAATGGACCTA

CCCGAAAAAGAGCTAGACGTGCTAATCATCGCCTATGGTCTTTTGAAGCTAAAACACCCTT
CACCAATCCCTAGCTAAACTTATAAACTAAGCTAAGCCCTAGCTAAATTAGATTTCTGATA
GGCAGGATTTATAAACCGTACAATCGTTTGATTTATATGTTATTAGATGGCATACTTCTTAAT
AAAAGAAGGGCCTGTGAAAAACGGAACGGTATTAGCTAAAAGAAAACAACACAATCCTA
TAATCCTAAAACATGCTCATTTGGTAACATTGCATGTCTCGTCCAATACGTCGTCACGAGGA
TAAATGGAATTCCTCTACTCCCGTCATCACGATCATAGAGGAAGGTATAATGAAATAGAAT
ATAAACATTTAAGTTACTATTCTATTTATGTATTGCGAATATTGCAGATAAAACAAAGCTTC
GAAACTGCGTTTCTTTGCGATGCAGAGAAACTATGTTTCTTTTGCTTCCGTTCACATCTACAG
TCGCAAAAGAATCCTGATGGGTTACAAATTTTACGCGTGTGATCTCCTTTAACCAGAAAAA
AGGTACTTAAGGAAACGACAGAATCACCGAATCTATCAAATCACCGTGTAAAATGGGTCTT
GTAAGAGACATGCACGTAACGGGCATCCTCTAATTCAAAAAAATCAGATTATTCCCAAACG
CAGCATAAATTATATCTTCTTGATCGTAAAGCTTTAGAAAAAGATACAAAAATCTTGTTCGA
ATCGACTATGTCAATAGAACATGAAATTGGTAGCTATTTCCATTTAACTTTACTTTCATTAA
GTATATAATTTTCATCTTACAGACGTAGACAGAAGGAAAGAAAAAAGGGAAAGGGGACTT
ACGATTGTCCGAGATTACAAAGAAACCAAACTGCACCACGCGGAAGACGCGGAAGATCTG
AAAAACACTAACATATGAAACCAAACGATTACACAACAGTCTAGCTTGAACGTTCGCGGA
GTGAAGTTCTCTTCTCGTTTTGTTCTTAATGAACTTTCAATTTTATTCTAAATTTCGATCTTGT
AATCCATGTTTTCATCATTCTTTCCATTTCTCAAAATTTTACTTTAATTCGTAATATCATGTTC
AAGCTAGACTGTTGTGTAATCGTTTGGTTTCATATGTTAGTGTTTTTCAGATCTTCCGCGTCTT
CCGCGTGGTGCAGTTTGGTTTCTTTGTAATCTCGGACAATCGTAAGTCCCCTTTCCCTTTTTTC
TTTCCTTCTGTCTACGTCTGTAAGATGAAAATTATATACTTAATGAAAGTAAAGTTAAATGG
AAATAGCTACCAATTTCATGTTCTATTGACATAGTCGATTCGAACAAGATTTTTGTATCTTTT
TCTAAAGCTTTACGATCAAGAAGATATAATTTATGCTGCGTTTGGGAATAATCTGATTTTTTT
GAATTAGAGGATGCCCGTTACGTGCATGTCTCTTACAAGACCCATTTTACACGGTGATTTGA
TAGATTCGGTGATTCTGTCGTTTCCTTAAGTACCTTTTTTCTGGTTAAAGGAGATCACACGCG
TAAAATTTGTAACCCATTAGGATTCTTTTGCGATTGTAGATGTCAACGGAAGCAAAAGAAA
CATAGTTTCTTTGCATCGCAAAGAAACGCAGTTTCGAAGCTTTGTTTCGTCTGCAATATTCG
CAATGCGTAAATGGAATAGTAACTTAAATGTTTATATTCTGTTTCGTTATACCTTCCTCTATG
ATCGTGATGACGGGAGTAGAGGAATTCCATTTATCCTCGTGACGACGTATTGGACGAGACA
TGCAATGTTACCAAATGAGCACGTTTTAGAATAGTAGAATTGCATTGTTTTTTTTTAGTTAAT
ACCGTTCCGTTTTTCATAGGCCCTTCTTTTATTAAGAAGTATGCCATTTAATAACATATAAAT
CAAACGATTGTACGGTTTATAAATCCTGCCTATCAGAAATCTAATTTAGCTAGGGCTTAGCT
TAGTTTATAAGTTTAGCTAGGAATTGGTGAAGGGTGTTTTAGCTTCAAAAGACCATAGGCG
ATGATTAGCACGTCTAGCTCTTTTTCGGGTAGGTCCATTTTTTTCTCTTCCGTCTGTTCCAATT
GGAATGAACGGTTGATGTACTGCGATGATGTCTACCGCAGTTTAAAATTTGCAAAGGAAGG
GTGGATGGGCCCCGTGGA

173. >MSTRG.3525.1 Gene=MSTRG.3525

ATCCGTGGCGCCGCCATACGCAATGAACAACATTTGTGAAGCACCAAACCCTCCTCTCTTTT
CCCTTCTTTCCTCTTAGCCTAGCAAACACTTTAACGTGTGTGTTTCCTACGCTTCGTCGAAAT
GTATGTGTGTCATCGCTATATAGTTTATGAAACGTCTTCTTTATCGAGTGTGCGAATCGTTTT
TGGGTTCGAATGGCAGTGACTATTGGCCCAGGATTCTCTTTATTTCACACTCAAAAAGTGGT
GTACTCATAAATCCGTCATCAATGACGTTAAAAGAAATTAAGCTATAAAATAAATGCTTGC

AAAGCAAGCTGAAAAAGAAACAATTATAAATACTTGACCAAAACGGTCAGGTTAAAAAA
AAAAAAAAAAAAAAAAAAAAAAAAAAAAAAAA
174. >MSTRG.3536.1 Gene=MSTRG.3536
TCACGGTTTTACATTCTTTTTAATATATATGTATTTTTATTATATACGTATATTAGAATTAATT
AGCGCCGTTAATTCAACTCGATTCTTTTTCTTTTGAATCGCTATCGGATTTGTCTTCGGAAGT
TCCATTTGTCAATTCTGGTTCTTTCTTCTTCTCTCCTTCCTCAGATTCGACGCTCGAAGCTTTTT
TCTCTTGACTTTCTTGGCTAGCTGCCTTCTTGTCCTTCGTCTTTCCTTTAGCAGGAGTTGCTGG
CTTCGGTTTTGGCTCAAGACTCGGATCCAATACAATTTTACCGATGTTTTTACGGTCGTGCAT
CTTCTGCATAGCCTCCGGGACGTCTTCCAAAGCCCATGTCGAGTCCACCACAGGCTTTATTT
TACCCTCGTTCCATAAAGCGAATACCCGTTCCACTGCTCGTCTCACAAATGCATGACTTCCA
TGCTGATACATCAAATGACGCAAATTCAACCCAGACAAGGTTTTATTCTCGTCGAACAGTTT
AATGGGAGATACTTTGTCCACTTGCCACCATGATCGTGCTGCTGAGAAGAAGCTCTTAGTTT
CGCCAGTGACAACATTACTAGATCCATAAAGAATATATTTTCCCATAGGTTTTAGCAAAGC
ATATCCTTTGTTGCATTCTTCACCACAAAGGCAATCCAAGACGATATCCACACCTTCTGGTG
AGATCTTTCTGACTTCGTTGCTATAGTCCGCGCCTCGTTCCAAGATGTGATCAATGGTGCCA
GTTGCTTTCAATGCTTCGTGCTTCGATTTGCTGCACACTCCAAAAATGGTTACATCCTTCACA
GTTTTGGCCAATTGGACGACTGCCTGACCCACACCCCCGCCGGCGCTGTGGAGTAACAGAT
TCTTTCCGGGCGTTAAATTAGCCAGTTCGAACAACAGAATGTAAGCAACAGTATAGTTCAT
CGTGATCGCCGCTGCATCGAGGTAGCTCATTCCGGCTGGTAGCGCGAAAACGGACGTGGCT
GGTACAGCGACAAGCTCTGCCCACGCCTTGTGATCAGGAAGCGCGACCACTCGATCGCCCA
CTTTAAAGTTCTCCACGCCCTCGCCGACTTGCTCGATATCACCTGCGCACTCGGATCCCATA
ATGAAAGGAGTTTTTGGTGGGGAATCAATAGCACCCTGCCTCGCCATCAAATCTTGGAAAT
TTAGTCCACATGCTTTGACTCGGATAAGAACTTCTCCTTCGGAGAGGGTGGGCTCAGGCTTC
CTCAAAGCCTTGACACTTTTCAGTCCGCCGAAACCGTTCAGTACTATAGCCCGCATATCCTT
TGGTTCTGGCGTGGGTTTCTCTTCGCCATTCTCCTTGGGTTTATCACCGTCGCCATTCTCTTCG
ACTTTCTTTTCTTCTTCTTTATTTTCTTTTGGCTCTTCTTTTTCTTGCTTCGACGGCGCCGCTTCC
TGTGGTGGCTTTTCACCCTCGGCACTTGGGCTGGTTTCATTTTTGTCTTCTGCCATTTTTCTTTT
TTATT

175. >MSTRG.3551.2 Gene=MSTRG.3551
ATTATAAATAAAATAAATTATTTAAATATTTTTTAATTTATATATTTATATTCCTAATTTTATT
TTTTAATTATTATAAAAAATTTATATTAATACTTTATTTTATTCACCATGGTGACGAATATAG
CGAGGTTATATTGATCTTCTCTAAAAGATGTTTATTTGTAGTTATTAGGATAGTGTAAAATA
GAGAATCGAATTTAAATTTCAAAAAATGTATATATGATAAATTTCTTATGATAAAAATAAA
TTAATAATTTTTTTTATAAATTATTATTTTTTATTTCTGATTTTTATATCTATCTTCTAATATATT
GTAAATTTCATCGTAAAATTTATAGGCAATGACAGAACATAAAGGTACATCTTTTTACAATT
CTTGTGTGAAAGTTCCTGTAAATCCCTGTAATAGGCAACCAACTCCTTCTTATGAACGTCTT
GAATTTAAAAAGAATAAAGAAG

176. >MSTRG.3555.6 Gene=MSTRG.3555
GACATTTCCTGAGTCGTCTTCGAACGGACAAGGCTGTATCTTGCTCGGTGATTTATAGTCTT
GTGTGTAGTTGTGCAACTTTCTTCCATTGTTCTACAAGAAACACGAGCCAGATACAGTCTCA
CCGTTTGAGATGGCATTGGAACGGACGGAGGAACTTGCTGTTTGCAGCGATGCTGGGCGTA

CCGCAGAATTGGGGACCCCATGTTCTAGTCTCTACACCTTTCATTGGTTTGAAGTATGGCGG
TTTACGTAAAAAAATGTGTACTTATATAGCTTAAAGAAAATTTGTATAGTCAATTTAACCTT
TATAGATTTAAATAGCGTTTTAAATGTTTACTTAATGATCGAAGAATAATTTTATATGTAGAT
TTTGTACTTACTTATAAATGTTAATTAACAGTTTTGTAAATATTGTGATACAGCAAAACAGC
AGCGGAAAAAATAAAAAAGAATGATAAAAATTAAAAATGTTAATAATTTTCCTATGATTTT
TAAATCGCCATATTAATGCCAAAAATCCTTTTCAGTCCAGCATACTACATAAAATTCTGTCG
AACGCAAGAGTGTCCCGCACGTGTGTCCAGGCTGTTGGTCGTCATCGGCGACACTATTTGG
CCTGTCTATATATGAGATAAGGAATGGTGAGAGGATTGGTATGGGATGAACAAAGGGGCA
AAATCTTTGTTTTTAGAAATTTGCACACCACGATCAAGTCGTACATAAACGTCTTCTCATGTT
CGCGCTTCCGTATCTTGAACATCTGAGCATCGACTAATTCAAAAATCAATGTGATGCGTGAT
ATACATAGGCTTACTGTGACATACTGCAGCAACCTCGAGCAAGTTCTATTTTGGTGAGCTAA
TCCCTTCTGTTCGTTACCTGTGCAAATATATTTTCTCAGGTCCAGAAAAATTTTTCCAATGTT
TATGCAACCGAGTTCTATATATGGGTTTCCCTGGCTTATTAATAAATCCTGTTCAAAGTTGAT
CAAGCTGGATACTAGACATCATATATATTGTCCCATTTGAATTAAAATGTATAAAAGCTTCA
AGGTATGGAGTAAATTAAATGCATCTTTGTTGAATTTAGATGATCCAGTAAGGCGAGTTACT
GCAAGTAGCACAACTAATTTTAATAATAGGCATGCATATAAAATATAAACAGCTCAATTTG
AATAGGAAATTGCTACAAATTTTTTACTATATTAAATATATTTTTTTGGGAATCATTACTAGA
TACTTACTTTTCACAGATTTTGATATTACTTCGCTTACTCATTGGGTCGTAGAGCAAGAAGA
ACTTAATGTATCATTAGATTAACAAAAAAAAATTCCTGATAAGTGATGTGCAACAGGGTTAT
GGAAGTAAAGAGACCGATTTAATATTACAGTTTAATTGATTTGAGAAATACATTGAGAATT
AACGAATACTCTGAAAAAATTTTTTAATAATAGTAGGAAAGCAGTGATCAAGGATGAACA
AGCAGG

177. >MSTRG.3555.3 Gene=MSTRG.3555
GACATTTCCTGAGTCGTCTTCGAACGGACAAGGCTGTATCTTGCTCGGTGATTTATAGTCTT
GTGTGTAGTTGTGCAACTTTCTTCCATTGTTCTACAAGAAACACGAGCCAGATACAGTCTCA
CCGTTTGAGATGGCATTGGAACGGACGGAGGAACTTGCTGTTTGCAGCGATGCTGGGCGTA
CCGCAGAATTGGGGACCCCATGTTCTAGTCTCTACACCTTTCATTGGTTTGGTTTGTAAACTG
TATCGGAACCAATGCATTTCTATTTGCAATATTTTTGTAATAATATACCAATCGAGTTCGTCA
AACAAGTTAAAAGAGCGTTTGATTCGATATTTTTTTTTTTCAGAAGTATGGCGGTTTACGTA
AAAAAATGTGTACTTATATAGCTTAAAGAAAATTTGTATAGTCAATTTAACCTTTATAGATT
TAAATAGCGTTTTAAATGTTTACTTAATGATCGAAGAATAATTTTATATGTAGATTTTGTACT
TACTTATAAATGTTAATTAACAGTTTTGTAAATATTGTGATACAGCAAAACAGCAGCGGAA
AAAATAAAAAAGAATGATAAAAATTAAAAATGTTAATAATTTTCCTATGATTTTTAAATCG
CCATATTAATGCCAAAAATCCTTTTCAGTCCAGCATACTACATAAAATTCTGTCGAACGCA
AGAGTGTCCCGCACGTGTGTCCAGGCTGTTGGTCGTCATCGGCGACACTATTTGGCCTGTCT
ATATATGAGATAAGGAATGGTGAGAGGATTGGTATGGGATGAACAAAGGGGCAAAATCTT
TGTTTTTAGAAATTTGCACACCACGATCAAGTCGTACATAAACGTCTTCTCATGTTCGCGCTT
CCGTATCTTGAACATCTGAGCATCGACTAATTCAAAAATCAATGTGATGCGTGATATACAT
AGGCTTACTGTGACATACTGCAGCAACCTCGAGCAAGTTCTATTTTGGAATACCCAATTTCG
TAATCATGGTGTACAACTAAACAACTACAAGCAGTTATTTCTATTCCTGGAGCATGTTGGAT
TCGTATTGCAAAGTGGTATGTTGATTAGAATGATCTAGTTCCGAGTTGTAGGGGTGGGAGG
GAGTTACTATGTTTCAGCTAAGCTTGGATAGAGAAAAAATCTATTTAATCGACGAGGTATA

TAAAGATGTACTGAAAATATATTTTGGTAACTCCTAATTGGATAAATATGAATAATTTTTTA
ATCTTAATAAATATCACAATCTATATATCTATTTATATAACTGCATTTTTGACAAGAAATATT
GATAAAAAAAAAAAAAGGTAAAAAAATCCGATTTTTAAAATAGAAAAGGTATGTTTTGGTT
GAAGGTAACAATTTAATATGATCAATGATTTGTAATAAATAGTTGAAAAAACGAGAAATA
GGTGAAATTATTATACTGTAAGCTGCAGGTTTAGTATTATATTTATCTTTCATCATAATGGAT
GCTATATTATCGATGACATTTTATAATAATGGAGATATATCCTATATTATATCTGCAATACC
AGGAAATAATTCTACAATTTCAACTATTTGAACTATTCACAATTATAATAGATAAACATGAT
GCAATATATTTCATTCGGCTGAAGAAGAAATTCAATTGACAATGGAAACAGATTTCTGCTA
AAAGAAGAGTAAAACTGATTGTAATATATATCATTGTTTACTATTTTACGTTTAACTATTTTA
GATCTACAAAAATTGACATTTCATTTTAATAAATGTATTTTAAAATTACATTTGACCTTGATA
TTTTTATAATTTATCAAGACATAGGTATAGGACATAGGTCTAGGAAAAAGGAGACAAAGG
AATAAAGCTGCCGATGGTTTTATTTTTTCACAATGAGA

178. >MSTRG.3555.2 Gene=MSTRG.3555
GACATTTCCTGAGTCGTCTTCGAACGGACAAGGCTGTATCTTGCTCGGTGATTTATAGTCTT
GTGTGTAGTTGTGCAACTTTCTTCCATTGTTCTACAAGAAACACGAGCCAGATACAGTCTCA
CCGTTTGAGATGGCATTGGAACGGACGGAGGAACTTGCTGTTTGCAGCGATGCTGGGCGTA
CCGCAGAATTGGGGACCCCATGTTCTAGTCTCTACACCTTTCATTGGTTTGGTTTGTAAACTG
TATCGGAACCAATGCATTTCTATTTGCAATATTTTTGTAATAATATACCAATCGAGTTCGTCA
AACAAGTTAAAAGAGCGTTTGATTCGATATTTTTTTTTTTTCAGAAGTATGGCGGTTTACGTA
AAAAAATGTGTACTTATATAGCTTAAAGAAAATTTGTATAGTCAATTTAACCTTTATAGATT
TAAATAGCGTTTTAAATGTTTACTTAATGATCGAAGAATAATTTTATATGTAGATTTTGTACT
TACTTATAAATGTTAATTAACAGTTTTGTAAATATTGTGATACAGCAAAACAGCAGCGGAA
AAAATAAAAAAGAATGATAAAAATTAAAAATGTTAATAATTTTCCTATGATTTTTAAATCG
CCATATTAATGCCAAAAATCCTTTTCAGTCCAGCATACTACATAAAATTCTGTCGAACGCA
AGAGTGTCCCGCACGTGTGTCCAGGCTGTTGGTCGTCATCGGCGACACTATTTGGCCTGTCT
ATATATGAGATAAGGAATGGTGAGAGGATTGGTATGGGATGAACAAAGGGGCAAAATCTT
TGTTTTTAGAAATTTGCACACCACGATCAAGTCGTACATAAACGTCTTCTCATGTTCGCGCTT
CCGTATCTTGAACATCTGAGCATCGACTAATTCAAAAATCAATGTGATGCGTGATATACAT
AGGCTTACTGTGACATACTGCAGCAACCTCGAGCAAGTTCTATTTTGGTGAGCTAATCCCTT
CTGTTCGTTACCTGTGCAAATATATTTTCTCAGGTCCAGAAAAATTTTTCCAATGTTTATGCA
ACCGAGTTCTATATATGGGTTTCCCTGGCTTATTAATAAATCCTGTTCAAAGTTGATCAAGC
TGGATACTAGACATCATATATATTGTCCCATTTGAATTAAAATGTATAAAAGCTTCAAGGTA
TGGAGTAAATTAAATGCATCTTTGTTGAATTTAGATGATCCAGTAAGGCGAGTTACTGCAA
GTAGCACAACTAATTTTAATAATAGGCATGCATATAAAATATAAACAGCTCAATTTGAATA
GGAAATTGCTACAAATTTTTTACTATATTAAATATATTTTTTTGGGAATCATTACTAGATACT
TACTTTTCACAGATTTTGATATTACTTCGCTTACTCATTGGGTCGTAGAGCAAGAAGAACTT
AATGTATCATTAGATTAACAAAAAAAATTCCTGATAAGTGATGTGCAACAGGGTTATGGAA
GTAAAGAGACCGATTTAATATTACAGTTTAATTGATTTGAGAAATACATTGAGAATTAACG
AATACTCTGAAAAAATTTTTTAATAATAGTAGGAAAGCAGTGATCAAGGATGAACAAGCA
GGATATTTCTAAATTAAATTTTTAAGTATAACATGTAGATAATATTTTTATATTTTGATTTTA
AAAAACAAAATTATGTTTAATGTTTTTAAATTCCAAGGAAGTACATCATCAATGATTCAATT
CTCGGATGTTGGAAATTGTTTACAATTAATTGTACAATCATAATTAAGCAAGTTAAAGGTTT

GATTTGAAGTTTGTTCAAAGGAATTTAATTTCTTCTACACATGGTTGCAAATGCAATGTGAA
GTATCATGCTTTTATAATTGTTTTAAATATTTTTTCTTGAAATATTGTGGTTCGGGTGTTTGTTT
TGTACTGCAGGAATACCCAATTTCGTAATCATGGTGTACAACTAAACAACTACAAGCAGTT
ATTTCTATTCCTGGAGCATGTTGGATTCGTATTGCAAAGTGGTATGTTGATTAGAATGATCTA
GTTCCGAGTTGTAGGGGTGGGAGGGAGTTACTATGTTTCAGCTAAGCTTGGATAGAGAAAA
AATCTATTTAATCGACGAGGTATATAAAGATGTACTGAAAATATATTTTGGTAACTCCTAAT
TGGATAAATATGAATAATTTTTTAATCTTAATAAATATCACAATCTATATATCTATTTATATA
ACTGCATTTTTGACAAGAAATATTGATAAAAAAAAAAAAAGGTAAAAAAATCCGATTTTTAA
AATAGAAAAGGTATGTTTTGGTTGAAGGTAACAATTTAATATGATCAATGATTTGTAATAA
ATAGTTGAAAAAACGAGAAATAGGTGAAATTATTATACTGTAAGCTGCAGGTTTAGTATTA
TATTTATCTTTCATCATAATGGATGCTATATTATCGATGACATTTTATAATAATGGAGATATA
TCCTATATTATATCTGCAATACCAGGAAATAATTCTACAATTTCAACTATTTGAACTATTCA
CAATTATAATAGATAAACATGATGCAATATATTTCATTCGGCTGAAGAAGAAATTCAATTG
ACAATGGAAACAGATTTCTGCTAAAAGAAGAGTAAAACTGATTGTAATATATATCATTGTT
TACTATTTTACGTTTAACTATTTTAGATCTACAAAAATTGACATTTCATTTTAATAAATGTAT
TTTAAAATTACATTTGACCTTGATATTTTTATAATTTATCAAGACATAGGTATAGGACATAG
GTCTAGGAAAAAGGAGACAAAGGAATAAAGCTGCCGATGGTTTTATTTTTTCACAATGAGA
AAAAGGCGGATTAGTATATATTTCAAATAATTTAAATATAAAATTAGTTATAGCATGTTAAT
AAGCATGGTAGTGTTATATTCATAGAAATGCTCAAATTATCGTTATTTAAATATACCTGTTTT
GGCTCTTTTGGATGTTTGTGATTTTTGCTTCAGGAAATCGAAGATAATTTTATTCTTATATGA
ATTTTATGATAAATTTTCTATTCTATGGAGAAAAATGCTATGAAATACACCTGAATATGTAT
GTATTATATAAATATTATTTTTATTATATTTAGAAAACGATGCCGGATATATGAACAGTATA
AATATAAAGCTTATATTCAAGATAACAACGGGGTGAATAACTATCGATATTTGATATATTTA
TGAAGATAAGTTTTCAATTATATTTAAATATGATAATATATAAGTTGAAATAAATTTTTATTG
AAATTTTTTATTTAAAAGAAAAAGATGAAGGATTAAATTACTAACTTTATAGTATCTAGATA
CATTTCAGAAAATTTGTGATATAGAAAAAAATTTTCTAAATGGAAGAGAGGTCATTGTTAT
ATCATCTTAGCCGAGATTTGATTAAGACTTGGCTGAATCTAAATTTATTTTAGATAATAAAA
AAAGTATGTGTCAGGTAAATTAAATGTTCAAAAAACTTACATATTTTAAAAATGTATTTAAA
ATATGTTGTTAAAAATAACTTCGGTTATATCAAGATTATAGTTTATCTAAATTGATGAACAT
GAGTTTTCATTTTCGATTTTACTTTTTGAAGAAAATATCGAATATTAAATCGAATATATCTAT
TCAAGAAAAATTTAAATCAAGATAAATTGTTACAACTTGAAATGCTGATTACGTTCAATTA
AATGGATCATTGGATAATTGAAATATATTCGACAAGATTTTTAAATTAATAACTTTATAAAT
CTCTCCTAATTAGAAAAGAAATATAAATGTTCTTTTTTTTTTATTGAGAAATGATTTTAAATC
TAATAAATAATGATCTGAAATTAATTCGAGTAAAATCTGTCGTACATACAACAATTGAAGA
TTTTGAATGTAAAAAAAATTGACGCAAAATTAAAATAGGATTTAATTTTTCATTAAAAAATT
TAATGATGAGTGTACATTTTCAGAACATATTTGAAAATATTTAAAAAAATATATATATATAA
AATTGAAGTAATTTTCTTTGTAATAAAAAATAATATAAAGTGAATAGAATAATAGAAATAC
AAATAGTAATCTATAACATCTGGCATAGTTCGAATTATTAAAAATGATTATATTCTTCAAAA
AACATAACCTTAAAAGAGAATCAACGTGGGATTTTATGGTATATCTACTACATTGATTCAA
AGGAGACTCGATACGAGTCTTTCTAGGATTCAAAGATTATCAACGTAGGATTCAGAAGAAA
TTATCACAGGATTTCGAAGGATTTAAAGAAGACTATCAACGCGGGACTTCATGAAATTCAG
AAGATCGACTCAGGATTTCGTGAGATTCGGAAAAGGTTACTGATGCGGGACTTCGTGGGAT
TCAAAAAACTATCAACGCGGGACTTTGTGGGATTCGAAGAAAGACTATCAACGTGGGACTT

CGTGGGATTCAAAGAAGACTATCAACGCCGGACTGTGGGATTCCAAGAAGACTATCAACG
TGGGACTTCGTGGGATTCAAAGAAGAGTATCGACGTGGGACTTCGTGGGATTCAAAGAGG
ACTATCGACGTGGGACTTCGTGGGATTCAAAGAAGACTATCGACGCGGGACTTCGTGGGAT
TCAAAGAAAACTATCAACGCCGGACTGTGGGATTCAAAGAAGACTATCAACGCTGGATCG
AGGGATTCAAAGAAGACTATCAACGTGGGACTTCGTGGGATTCAAAGAAGACTATCAACA
TCGGACTGTGGGATTCAAAGAAGATTATCAACGTGGGACTTCGTGGGATTCAAAGAAGATT
ATGAACGCAGAACTTTGTGGGATTCAAAGAAGATCATCAATACGGTTTAATGAAATTTCAT
GATAAATTATTAATGCGGGATTTTATAGGATTGTAGGAAAGCGCCTTCGTATTCAAAAAAA
GACTTCGATGAAATTTGTAATCCAAAAAACCGTCATTGCCAGATATCATGAAATTCAAAAG
GAATATTAAAAAAAGATTTAATGAAATTTAAATATTAGCGAGATAAATTTTTAAAGATATA
AAAAATTATTGAAATTGGAATTAGAAACAAATCTCAAATTCTGATATT

179. >MSTRG.3555.7 Gene=MSTRG.3555
GACATTTCCTGAGTCGTCTTCGAACGGACAAGGCTGTATCTTGCTCGGTGATTTATAGTCTT
GTGTGTAGTTGTGCAACTTTCTTCCATTGTTCTACAAGAAACACGAGCCAGATACAGTCTCA
CCGTTTGAGATGGCATTGGAACGGACGGAGGAACTTGCTGTTTGCAGCGATGCTGGGCGTA
CCGCAGAATTGGGGACCCCATGTTCTAGTCTCTACACCTTTCATTGGTTTGGTTTGTAAACTG
TATCGGAACCAATGCATTTCTATTTGCAATATTTTTGTAATAATATACCAATCGAGTTCGTCA
AACAAGTTAAAAGAGCGTTTGATTCGATATTTTTTTTTTTTCAGAAGTATGGCGGTTTACGTA
AAAAAATGTGTACTTATATAGCTTAAAGAAAATTTGTATAGTCAATTTAACCTTTATAGATT
TAAATAGCGTTTTAAATGTTTACTTAATGATCGAAGAATAATTTTATATGTAGATTTTGTACT
TACTTATAAATGTTAATTAACAGTTTTGTAAATATTGTGATACAGCAAAACAGCAGCGGAA
AAAATAAAAAAGAATGATAAAAATTAAAAATGTTAATAATTTTCCTATGATTTTTAAATCG
CCATATTAATGCCAAAAATCCTTTTCAGTCCAGCATACTACATAAAATTCTGTCGAACGCA
AGAGTGTCCCGCACGTGTGTCCAGGCTGTTGGTCGTCATCGGCGACACTATTTGGCCTGTCT
ATATATGAGATAAGGAATGGTGAGAGGATTGGTATGGGATGAACAAAGGGGCAAAATCTT
TGTTTTTAGAAATTTGCACACCACGATCAAGTCGTACATAAACGTCTTCTCATGTTCGCGCTT
CCGTATCTTGAACATCTGAGCATCGACTAATTCAAAAATCAATGTGATGCGTGATATACAT
AGGCTTACTGTGACATACTGCAGCAACCTCGAGCAAGTTCTATTTTGGTGAGCTAATCCCTT
CTGTTCGTTACCTGTGCAAATATATTTTCTCAGGTCCAGAAAAATTTTTCCAATGTTTATGCA
ACCGAGTTCTATATATGGGTTTCCCTGGCTTATTAATAAATCCTGTTCAAAGTTGATCAAGC
TGGATACTAGACATCATATATATTGTCCCATTTGAATTAAAATGTATAAAAGCTTCAAGGTA
TGGAGTAAATTAAATGCATCTTTGTTGAATTTAGATGATCCAGTAAGGCGAGTTACTGCAA
GTAGCACAACTAATTTTAATAATAGGCATGCATATAAAATATAAACAGCTCAATTTGAATA
GGAAATTGCTACAAATTTTTTACTATATTAAATATATTTTTTTGGGAATCATTACTAGATACT
TACTTTTCACAGATTTTGATATTACTTCGCTTACTCATTGGGTCGTAGAGCAAGAAGAACTT
AATGTATCATTAGATTAACAAAAAAAAATTCCTGATAAGTGATGTGCAACAGGGTTATGGAA
GTAAAGAGACCGATTTAATATTACAGTTTAATTGATTTGAGAAATACATTGAGAATTAACG
AATACTCTGAAAAAATTTTTTAATAATAGTAGGAAAGCAGTGATCAAGGATGAACAAGCA
GGATATTTCTAAATTAAATTTTTAAGTATAACATGTAGATAATATTTTTATATTTTGATTTTA
AAAAACAAAATTATGTTTAATGTTTTTAAATTCCAAGGAAGTACATCATCAATGATTCAATT
CTCGGATGTTGGAAATTGTTTACAATTAATTGTACAATCATAATTAAGCAAGTTAAAGGTTT
GATTTGAAGTTTGTTCAAAGGAATTTAATTTCTTCTACACATGGTTGCAAATGCAATGTGAA

GTATCATGCTTTTATAATTGTTTTAAATATTTTTTCTTGAAATATTGTGGTTCGGGTGTTTGTTT
TGTACTGCAGGAATACCCAATTTCGTAATCATGGTGTACAACTAAACAACTACAAGCAGTT
ATTTCTATTCCTGGAGCATGTTGGATTCGTATTGCAAAGTGAAGAGAATCAACGTGGGATTT
TATGGTATATCTACTACATTGATTCAAAGGAGACTCGATACGAGTCTTTCTAGGATTCAAAG
ATTATCAACGTAGGATTCAGAAGAAATTATCACAGGATTTCGAAGGATTTAAAGAAGACTA
TCAACGCGGGACTTCATGAAATTCAGAAGATCGACTCAGGATTTCGTGAGATTCGGAAAAG
GTTACTGATGCGGGACTTCGTGGGATTCAAAAAACTATCAACGCGGGACTTTGTGGGATTC
GAAGAAAGACTATCAACGTGGGACTTCGTGGGATTCAAAGAAGACTATCAACGCCGGACT
GTGGGATTCCAAGAAGACTATCAACGTGGGACTTCGTGGGATTCAAAGAAGAGTATCGAC
GTGGGACTTCGTGGGATTCAAAGAGGACTATCGACGTGGGACTTCGTGGGATTCAAAGAAG
ACTATCGACGCGGGACTTCGTGGGATTCAAAGAAAACTATCAACGCCGGACTGTGGGATTC
AAAGAAGACTATCAACGCTGGATCGAGGGATTCAAAGAAGACTATCAACGTGGGACTTCG
TGGGATTCAAAGAAGACTATCAACATCGGACTGTGGGATTCAAAGAAGATTATCAACGTG
GGACTTCGTGGGATTCAAAGAAGATTATGAACGCAGAACTTTGTGGGATTCAAAGAAGATC
ATCAATACGGTTTAATGAAATTTCATGATAAATTATTAATGCGGGATTTTATAGGATTGTAG
GAAAGCGCCTTCGTATTCAAAAAAAGACTTCGATGAAATTTGTAATCCAAAAAACCGTCAT
TGCCAGATATCATGAAATTCAAAAGGAATATTAAAAAAAGATTTAATGAAATTTAAATATT
AGCGAGATAAATTTTTAAAGATATAAAAAATTATTGAAATTGGAATTAGAAACAAATCTCA
AATTCTGATATT

180. >MSTRG.3555.1 Gene=MSTRG.3555
GACATTTCCTGAGTCGTCTTCGAACGGACAAGGCTGTATCTTGCTCGGTGATTTATAGTCTT
GTGTGTAGTTGTGCAACTTTCTTCCATTGTTCTACAAGAAACACGAGCCAGATACAGTCTCA
CCGTTTGAGATGGCATTGGAACGGACGGAGGAACTTGCTGTTTGCAGCGATGCTGGGCGTA
CCGCAGAATTGGGGACCCCATGTTCTAGTCTCTACACCTTTCATTGGTTTGGTTTGTAAACTG
TATCGGAACCAATGCATTTCTATTTGCAATATTTTTGTAATAATATACCAATCGAGTTCGTCA
AACAAGTTAAAAGAGCGTTTGATTCGATATTTTTTTTTTTTCAGAAGTATGGCGGTTTACGTA
AAAAAATGTGTACTTATATAGCTTAAAGAAAATTTGTATAGTCAATTTAACCTTTATAGATT
TAAATAGCGTTTTAAATGTTTACTTAATGATCGAAGAATAATTTTATATGTAGATTTTGTACT
TACTTATAAATGTTAATTAACAGTTTTGTAAATATTGTGATACAGCAAAACAGCAGCGGAA
AAAATAAAAAAGAATGATAAAAATTAAAAATGTTAATAATTTTCCTATGATTTTTAAATCG
CCATATTAATGCCAAAAATCCTTTTCAGTCCAGCATACTACATAAAATTCTGTCGAACGCA
AGAGTGTCCCGCACGTGTGTCCAGGCTGTTGGTCGTCATCGGCGACACTATTTGGCCTGTCT
ATATATGAGATAAGGAATGGTGAGAGGATTGGTATGGGATGAACAAAGGGGCAAAATCTT
TGTTTTTAGAAATTTGCACACCACGATCAAGTCGTACATAAACGTCTTCTCATGTTCGCGCTT
CCGTATCTTGAACATCTGAGCATCGACTAATTCAAAAATCAATGTGATGCGTGATATACAT
AGGCTTACTGTGACATACTGCAGCAACCTCGAGCAAGTTCTATTTTGGTGAGCTAATCCCTT
CTGTTCGTTACCTGTGCAAATATATTTCTCAGGTCCAGAAAAATTTTTCCAATGTTTATGCA
ACCGAGTTCTATATATGGGTTTCCCTGGCTTATTAATAAATCCTGTTCAAAGTTGATCAAGC
TGGATACTAGACATCATATATATTGTCCCATTTGAATTAAAATGTATAAAAGCTTCAAGGTA
TGGAGTAAATTAAATGCATCTTTGTTGAATTTAGATGATCCAGTAAGGCGAGTTACTGCAA
GTAGCACAACTAATTTTAATAATAGGCATGCATATAAAATATAAACAGCTCAATTTGAATA
GGAAATTGCTACAAATTTTTTACTATATTAAATATATTTTTTTGGGAATCATTACTAGATACT

TACTTTTCACAGATTTTGATATTACTTCGCTTACTCATTGGGTCGTAGAGCAAGAAGAACTT
AATGTATCATTAGATTAACAAAAAAAAATTCCTGATAAGTGATGTGCAACAGGGTTATGGAA
GTAAAGAGACCGATTTAATATTACAGTTTAATTGATTTGAGAAATACATTGAGAATTAACG
AATACTCTGAAAAAATTTTTTAATAATAGTAGGAAAGCAGTGATCAAGGATGAACAAGCA
GGATATTTCTAAATTAAATTTTTAAGTATAACATGTAGATAATATTTTTATATTTTGATTTTA
AAAAACAAAATTATGTTTAATGTTTTTAAATTCCAAGGAAGTACATCATCAATGATTCAATT
CTCGGATGTTGGAAATTGTTTACAATTAATTGTACAATCATAATTAAGCAAGTTAAAGGTTT
GATTTGAAGTTTGTTCAAAGGAATTTAATTTCTTCTACACATGGTTGCAAATGCAATGTGAA
GTATCATGCTTTTATAATTGTTTTAAATATTTTTTCTTGAAATATTGTGGTTCGGGTGTTTGTTT
TGTACTGCAGGAATACCCAATTTCGTAATCATGGTGTACAACTAAACAACTACAAGCAGTT
ATTTCTATTCCTGGAGCATGTTGGATTCGTATTGCAAAGTGGTATGTTGATTAGAATGATCTA
GTTCCGAGTTGTAGGGGTGGGAGGGAGTTACTATGTTTCAGCTAAGCTTGGATAGAGAAAA
AATCTATTTAATCGACGAGGTATATAAAGATGTACTGAAAATATATTTTGGTAACTCCTAAT
TGGATAAATATGAATAATTTTTTAATCTTAATAAATATCACAATCTATATATCTATTTATATA
ACTGCATTTTTGACAAGAAATATTGATAAAAAAAAAAAAAGGTAAAAAAATCCGATTTTTAA
AATAGAAAAGGTATGTTTTGGTTGAAGGTAACAATTTAATATGATCAATGATTTGTAATAA
ATAGTTGAAAAAACGAGAAATAGGTGAAATTATTATACTGTAAGCTGCAGGTTTAGTATTA
TATTTATCTTTCATCATAATGGATGCTATATTATCGATGACATTTTATAATAATGGAGATATA
TCCTATATTATATCTGCAATACCAGGAAATAATTCTACAATTTCAACTATTTGAACTATTCA
CAATTATAATAGATAAACATGATGCAATATATTTCATTCGGCTGAAGAAGAAATTCAATTG
ACAATGGAAACAGATTTCTGCTAAAAGAAGAGTAAAACTGATTGTAATATATATCATTGTT
TACTATTTTACGTTTAACTATTTTAGATCTACAAAAATTGACATTTCATTTTAATAAATGTAT
TTTAAAATTACATTTGACCTTGATATTTTTATAATTTATCAAGACATAGGTATAGGACATAG
GTCTAGGAAAAAGGAGACAAAGGAATAAAGCTGCCGATGGTTTTATTTTTTCACAATGAGA
AAAAGGCGGATTAGTATATATTTCAAATAATTTAAATATAAAATTAGTTATAGCATGTTAAT
AAGCATGGTAGTGTTATATTCATAGAAATGCTCAAATTATCGTTATTTAAATATACCTGTTTT
GGCTCTTTTGGATGTTTGTGATTTTTGCTTCAGGAAATCGAAGATAATTTTATTCTTATATGA
ATTTTATGATAAATTTTCTATTCTATGGAGAAAAATGCTATGAAATACACCTGAATATGTAT
GTATTATATAAATATTATTTTTATTATATTTAGAAAACGATGCCGGATATATGAACAGTATA
AATATAAAGCTTATATTCAAGATAACAACGGGGTGAATAACTATCGATATTTGATATATTTA
TGAAGATAAGTTTTCAATTATATTTAAATATGATAATATATAAGTTGAAATAAATTTTTATTG
AAATTTTTTATTTAAAAGAAAAAGATGAAGGATTAAATTACTAACTTTATAGTATCTAGATA
CATTTCAGAAAATTTGTGATATAGAAAAAAATTTTCTAAATGGAAGAGAGGTCATTGTTAT
ATCATCTTAGCCGAGATTTGATTAAGACTTGGCTGAATCTAAATTTATTTTAGATAATAAAA
AAAGTATGTGTCAGGTAAATTAAATGTTCAAAAAACTTACATATTTTAAAAATGTATTTAAA
ATATGTTGTTAAAAATAACTTCGGTTATATCAAGATTATAGTTTATCTAAATTGATGAACAT
GAGTTTTCATTTTCGATTTTACTTTTTGAAGAAAATATCGAATATTAAATCGAATATATCTAT
TCAAGAAAAATTTAAATCAAGATAAATTGTTACAACTTGAAATGCTGATTACGTTCAATTA
AATGGATCATTGGATAATTGAAATATATTCGACAAGATTTTTAAATTAATAACTTTATAAAT
CTCTCCTAATTAGAAAAGAAATATAAATGTTCTTTTTTTTTTATTGAGAAATGATTTTAAATC
TAATAAATAATGATCTGAAATTAATTCGAGTAAAATCTGTCGTACATACAACAATTGAAGA
TTTTGAATGTAAAAAAAAATTGACGCAAAATTAAAATAGGATTTAATTTTTCATTAAAAAGT
AAGAATTGTTTAATAAAATATATTTTTAATTAAGTACATAATATTCAATCGGTAATAAATTT

TTTCATTTCAGATTTAATGATGAGTGTACATTTTCAGAACATATTTGAAAATATTTAAAAAA
ATATATATATATAAAATTGAAGTAATTTTCTTTGTAATAAAAAATAATATAAAGTGAATAG
AATAATAGAAATACAAATAGTAATCTATAACATCTGGCATAGTTCGAATTATTAAAAATGA
TTATATTCTTCAAAAAACATAACCTTAAAAGAGAATCAACGTGGGATTTTATGGTATATCTA
CTACATTGATTCAAAGGAGACTCGATACGAGTCTTTCTAGGATTCAAAGATTATCAACGTA
GGATTCAGAAGAAATTATCACAGGATTTCGAAGGATTTAAAGAAGACTATCAACGCGGGA
CTTCATGAAATTCAGAAGATCGACTCAGGATTTCGTGAGATTCGGAAAAGGTTACTGATGC
GGGACTTCGTGGGATTCAAAAAACTATCAACGCGGGACTTTGTGGGATTCGAAGAAAGACT
ATCAACGTGGGACTTCGTGGGATTCAAAGAAGACTATCAACGCCGGACTGTGGGATTCCAA
GAAGACTATCAACGTGGGACTTCGTGGGATTCAAAGAAGAGTATCGACGTGGGACTTCGTG
GGATTCAAAGAGGACTATCGACGTGGGACTTCGTGGGATTCAAAGAAGACTATCGACGCG
GGACTTCGTGGGATTCAAAGAAAACTATCAACGCCGGACTGTGGGATTCAAAGAAGACTA
TCAACGCTGGATCGAGGGATTCAAAGAAGACTATCAACGTGGGACTTCGTGGGATTCAAA
GAAGACTATCAACATCGGACTGTGGGATTCAAAGAAGATTATCAACGTGGGACTTCGTGGG
ATTCAAAGAAGATTATGAACGCAGAACTTTGTGGGATTCAAAGAAGATCATCAATACGGTT
TAATGAAATTTCATGATAAATTATTAATGCGGGATTTTATAGGATTGTAGGAAAGCGCCTTC
GTATTCAAAAAAAGACTTCGATGAAATTTGTAATCCAAAAAACCGTCATTGCCAGATATCA
TGAAATTCAAAAGGAATATTAAAAAAAGATTTAATGAAATTTAAATATTAGCGAGATAAA
TTTTTAAAGATATAAAAAATTATTGAAATTGGAATTAGAAACAAATCTCAAATTCTGATATT
TGAAACTTATTACCAAATACAAGTGTGTTACTTAATGAATTTTCTAAAGAAAGGA

181. >MSTRG.3555.4 Gene=MSTRG.3555
GACATTTCCTGAGTCGTCTTCGAACGGACAAGGCTGTATCTTGCTCGGTGATTTATAGTCTT
GTGTGTAGTTGTGCAACTTTCTTCCATTGTTCTACAAGAAACACGAGCCAGATACAGTCTCA
CCGTTTGAGATGGCATTGGAACGGACGGAGGAACTTGCTGTTTGCAGCGATGCTGGGCGTA
CCGCAGAATTGGGGACCCCATGTTCTAGTCTCTACACCTTTCATTGGTTTGGTTTGTAAACTG
TATCGGAACCAATGCATTTCTATTTGCAATATTTTTGTAATAATATACCAATCGAGTTCGTCA
AACAAGTTAAAAGAGCGTTTGATTCGATATTTTTTTTTTTCAGAAGTATGGCGGTTTACGTA
AAAAAATGTGTACTTATATAGCTTAAAGAAAATTTGTATAGTCAATTTAACCTTTATAGATT
TAAATAGCGTTTTAAATGTTTACTTAATGATCGAAGAATAATTTTATATGTAGATTTTGTACT
TACTTATAAATGTTAATTAACAGTTTTGTAAATATTGTGATACAGCAAAACAGCAGCGGAA
AAAATAAAAAAGAATGATAAAAATTAAAAATGTTAATAATTTCCTATGATTTTTAAATCG
CCATATTAATGCCAAAAATCCTTTTCAGTCCAGCATACTACATAAAATTCTGTCGAACGCA
AGAGTGTCCCGCACGTGTGTCCAGGCTGTTGGTCGTCATCGGCGACACTATTTGGCCTGTCT
ATATATGAGATAAGGAATGGTGAGAGGATTGGTATGGGATGAACAAAGGGGCAAAATCTT
TGTTTTTAGAAATTTGCACACCACGATCAAGTCGTACATAAACGTCTTCTCATGTTCGCGCTT
CCGTATCTTGAACATCTGAGCATCGACTAATTCAAAAATCAATGTGATGCGTGATATACAT
AGGCTTACTGTGACATACTGCAGCAACCTCGAGCAAGTTCTATTTTGGTGAGCTAATCCCTT
CTGTTCGTTACCTGTGCAAATATATTTTCTCAGGTCCAGAAAAATTTTTCCAATGTTTATGCA
ACCGAGTTCTATATATGGGTTTCCCTGGCTTATTAATAAATCCTGTTCAAAGTTGATCAAGC
TGGATACTAGACATCATATATATTGTCCCATTTGAATTAAAATGTATAAAAGCTTCAAGGTA
TGGAGTAAATTAAATGCATCTTTGTTGAATTTAGATGATCCAGTAAGGCGAGTTACTGCAA
GTAGCACAACTAATTTTAATAATAGGCATGCATATAAAATATAAACAGCTCAATTTGAATA

GGAAATTGCTACAAATTTTTTACTATATTAAATATATTTTTTTGGGAATCATTACTAGATACT
TACTTTTCACAGATTTTGATATTACTTCGCTTACTCATTGGGTCGTAGAGCAAGAAGAACTT
AATGTATCATTAGATTAACAAAAAAAATTCCTGATAAGTGATGTGCAACAGGGTTATGGAA
GTAAAGAGACCGATTTAATATTACAGTTTAATTGATTTGAGAAATACATTGAGAATTAACG
AATACTCTGAAAAAATTTTTTAATAATAGTAGGAAAGCAGTGATCAAGGATGAACAAGCA
GGATATTTCTAAATTAAATTTTTAAGTATAACATGTAGATAATATTTTTATATTTTGATTTTA
AAAAACAAAATTATGTTTAATGTTTTTAAATTCCAAGGAAGTACATCATCAATGATTCAATT
CTCGGATGTTGGAAATTGTTTACAATTAATTGTACAATCATAATTAAGCAAGTTAAAGGTTT
GATTTGAAGTTTGTTCAAAGGAATTTAATTTCTTCTACACATGGTTGCAAATGCAATGTGAA
GTATCATGCTTTTATAATTGTTTTAAATATTTTTTCTTGAAATATTGTGGTTCGGGTGTTTGTTT
TGTACTGCAGGAATACCCAATTTCGTAATCATGGTGTACAACTAAACAACTACAAGCAGTT
ATTTCTATTCCTGGAGCATGTTGGATTCGTATTGCAAAGTGGTATGTTGATTAGAATGATCTA
GTTCCGAGTTGTAGGGGTGGGAGGGAGTTACTATGTTTCAGCTAAGCTTGGATAGAGAAAA
AATCTATTTAATCGACGAGGTATATAAAGATGTACTGAAAATATATTTTGGTAACTCCTAAT
TGGATAAATATGAATAATTTTTTAATCTTAATAAATATCACAATCTATATATCTATTTATATA
ACTGCATTTTTGACAAGAAATATTGATAAAAAAAAAAAAAGGTAAAAAAATCCGATTTTTAA
AATAGAAAAGGTATGTTTTGGTTGAAGGTAACAATTTAATATGATCAATGATTTGTAATAA
ATAGTTGAAAAAACGAGAAATAGGTGAAATTATTATACTGTAAGCTGCAGGTTTAGTATTA
TATTTATCTTTCATCATAATGGATGCTATATTATCGATGACATTTTATAATAATGGAGATATA
TCCTATATTATATCTGCAATACCAGGAAATAATTCTACAATTTCAACTATTTGAACTATTCA
CAATTATAATAGATAAACATGATGCAATATATTTCATTCGGCTGAAGAAGAAATTCAATTG
ACAATGGAAACAGATTTCTGCTAAAAGAAGAGTAAAACTGATTGTAATATATATCATTGTT
TACTATTTTACGTTTAACTATTTTAGATCTACAAAAATTGACATTTCATTTTAATAAATGTAT
TTTAAAATTACATTTGACCTTGATATTTTTATAATTTATCAAGACATAGGTATAGGACATAG
GTCTAGGAAAAAGGAGACAAAGGAATAAAGCTGCCGATGGTTTTATTTTTTCACAATGAGA
AAAAGGCGGATTAGTATATATTTCAAATAATTTAAATATAAAATTAGTTATAGCATGTTAAT
AAGCATGGTAGTGTTATATTCATAGAAATGCTCAAATTATCGTTATTTAAATATACCTGTTTT
GGCTCTTTTGGATGTTTGTGATTTTTGCTTCAGGAAATCGAAGATAATTTTATTCTTATATGA
ATTTTATGATAAATTTTCTATTCTATGGAGAAAAATGCTATGAAATACACCTGAATATGTAT
GTATTATATAAATATTATTTTTATTATATTTAGAAAACGATGCCGGATATATGAACAGTATA
AATATAAAGCTTATATTCAAGATAACAACGGGGTGAATAACTATCGATATTTGATATATTTA
TGAAGATAAGTTTTCAATTATATTTAAATATGATAATATATAAGTTGAAATAAATTTTTATTG
AAATTTTTTATTTAAAAGAAAAAGATGAAGGATTAAATTACTAACTTTATAGTATCTAGATA
CATTTCAGAAAATTTGTGATATAGAAAAAAATTTTCTAAATGGAAGAGAGGTCATTGTTAT
ATCATCTTAGCCGAGATTTGATTAAGACTTGGCTGAATCTAAATTTATTTTAGATAATAAAA
AAAGTATGTGTCAGGTAAATTAAATGTTCAAAAAACTTACATATTTTAAAAATGTATTTAAA
ATATGTTGTTAAAAATAACTTCGGTTATATCAAGATTATAGTTTATCTAAATTGATGAACAT
GAGTTTTCATTTTCGATTTTACTTTTTGAAGAAAATATCGAATATTAAATCGAATATATCTAT
TCAAGAAAAATTTAAATCAAGATAAATTGTTACAACTTGAAATGCTGATTACGTTCAATTA
AATGGATCATTGGATAATTGAAATATATTCGACAAGATTTTTAAATTAATAACTTTATAAAT
CTCTCCTAATTAGAAAAGAAATATAAATGTTCTTTTTTTTTTATTGAGAAATGATTTTAAATC
TAATAAATAATGATCTGAAATTAATTCGAGTAAAATCTGTCGTACATACAACAATTGAAGA
TTTTGAATGTAAAAAAAATTGACGCAAAATTAAAATAGGATTTAATTTTTCATTAAAAAATT

TAATGATGAGTGTACATTTTCAGAACATATTTGAAAATATTTAAAAAAATATATATATATAA
AATTGAAGTAATTTTCTTTGTAATAAAAAATAATATAAAGTGAATAGAATAATAGAAATAC
AAATAGTAATCTATAACATCTGGCATAGTTCGAATTATTAAAAATGATTATATTCTTCAAAA
AACATAACCTTAAGTTAGTATTAAATCAATATTAAAATACAAGTTTCAATTTGAATATAATT
AAAAAAACTATACTATCATAAAAACATAGAAGAAAAACAAAGTTGATTATAATGTATTGT
GAAAAAGAGAATCAACGTGGGATTTTATGGTATATCTACTACATTGATTCAAAGGAGACTC
GATACGAGTCTTTCTAGGATTCAAAGATTATCAACGTAGGATTCAGAAGAAATTATCACAG
GATTTCGAAGGATTTAAAGAAGACTATCAACGCGGGACTTCATGAAATTCAGAAGATCGA
CTCAGGATTTCGTGAGATTCGGAAAAGGTTACTGATGCGGGACTTCGTGGGATTCAAAAAA
CTATCAACGCGGGACTTTGTGGGATTCGAAGAAAGACTATCAACGTGGGACTTCGTGGGAT
TCAAAGAAGACTATCAACGCCGGACTGTGGGATTCCAAGAAGACTATCAACGTGGGACTT
CGTGGGATTCAAAGAAGAGTATCGACGTGGGACTTCGTGGGATTCAAAGAGGACTATCGA
CGTGGGACTTCGTGGGATTCAAAGAAGACTATCGACGCGGGACTTCGTGGGATTCAAAGA
AAACTATCAACGCCGGACTGTGGGATTCAAAGAAGACTATCAACGCTGGATCGAGGGATT
CAAAGAAGACTATCAACGTGGGACTTCGTGGGATTCAAAGAAGACTATCAACATCGGACT
GTGGGATTCAAAGAAGATTATCAACGTGGGACTTCGTGGGATTCAAAGAAGATTATGAAC
GCAGAACTTTGTGGGATTCAAAGAAGATCATCAATACGGTTTAATGAAATTTCATGATAAA
TTATTAATGCGGGATTTTATAGGATTGTAGGAAAGCGCCTTCGTATTCAAAAAAAGACTTCG
ATGAAATTTGTAATCCAAAAAACCGTCATTGCCAGATATCATGAAATTCAAAAGGAATATT
AAAAAAAGATTTAATGAAATTTAAATATTAGCGAGATAAATTTTTAAAGATATAAAAAATT
ATTGAAATTGGAATTAGAAACAAATCTCAAATTCTGATATTTGAAACTTATTACCAAATAC
AAGTGTGTTACTTAATGAATTTTCTAAAGAAAGGAT

182. >MSTRG.3571.1 Gene=MSTRG.3571
TAATTCTCATATTTTTACTTTCTGTTTTTTTTTCACTATTAATTCTCAATTTAATTACTTTTTAG
AATATAAATTCATATTATACGGTAAATTAATTCCTAGATAATTGATTTATCTTATAATTTAAT
TTTAATATTTTATAAATTAAAAAAATCGTATCTAAATATAATTAAAAAAAAAAAAAACAATA
ATTAGAAACGCGGTATAAAATAAAAAAAGACAATTAATAAATAAAGCAATCATTTTGCTT
CATAACATGAAAACAAGCCTTGTTTATTATAATTTATACTAGTATAAGAATTACTTATTGTG
AGCATTAAATCATAATTATCGTTTAACATATATATTGTTATAATATTTATTATATCATTTTAT
AATATTAAATTTGTTTTGTAATATTAAAATGCCTGCAACAACTCTACACAAATTTACGACAT
TCATAGGATTGATATCAATATTACATACAGCATATTCAGCTGCTCAACATCGTTCGTATTTA
CGAATAACAGAACAAGAATTCATTACTTTACCAATTGATATTTTAATACAAGGTATTATCAG
TTTATTTTTGGTTATGTATGGAGTTATGTATATTGCTGGTGATTTCAAAGAAATTCGAGCAGT
TGTAGATTTAGAAAATAAATCTTGGGAAACATTGAGAAATCTTCCTTCATTTCAAATATTTA
ATCATCGTGGAAGATCTTTATCACCTCAATACACCCAATAAAATATTTTTATTATTTATTCGT
GGAAAATGGACATATAATTAATCTTCTGCTTCTTTTTCCCTTTCAATTTCAG

183. >MSTRG.3617.2 Gene=MSTRG.3617
CATTTTATCGTACAAAACTTTGCATCACATTTGCTGCCTGTCCAAGTTTGGTAACTAAGGGTT
GAAAAATTCTGATCATTATATAGATAGGTTAATATGAATCCCGAAAAATTGAAGAAGCTTC
AAGCTCAAGTACGAATCGGCGGTAAGGGAACACCCCGACGCAAGAAGAAGGTTGTACATG
CTACTGCTGCTACGGATGATAAAAAATTACAAAGTTGTTTAAAAAAATTATCTGTGAATAC

AATTCCTGGTATAGAAGAGGTGTTACACAATTGAAACGACTAGCAAGTACAGTTTCTGGAA
GTACAGTTGGTAAATCAACATTGGAAGAGGATGATGAAGTGCCAGATTTAGTGGAGAATTT
TGATGAAGCTAGTAAAGAGGAGGTAATGAATCAGCCTAAATAACAATAATTGTTATATGA
ATGATAATTTAAATTTTATATATTATGTATAGAAAAACTACAGGAAAAAGAGTTAATATAA
TGCATGTGTGTTATCATTAGGAATATAATTTTTTCTTTTTGTGACTATAATTGTTTTCAATGGG
TTAAGAAATATGAAAACACACGCGCGCGCGCACGCGCACACACACACGTAAAAATTATAA
ATATATATATATATATATATATATATATATATATATA

184. >MSTRG.3621.3 Gene=MSTRG.3621
GATTATTCTCCAACCGTGATTCTCATTTCTAACCAATTATTACAAGATCTCTTTCATTTTGTTC
TGGAAAAGTCGAGCGTGTATTAAATATCGTTTTCTAACACAATATTTAATAAAAGTATTAAA
AAAATTGAAGAAAAATGTTTGGTCCAGGAGCTGCTCCGATCGTAGTTTTAAGTATGTAGAA
ACAATAACAATTTTGATTTTCATTTATTTGAGTAGTGAAGTGAGAAAATTCATGATGTCGTG
CGCTTTGATTATTGCAGGTCAAAATACAAAACGGGATGTTGGCCGAAAAGTTCAGAGAGA
AAATATACAAGCTGGAAAAGCAATTGCAGATGTCATTAGAACATGTCTAGGACCACAAGC
AATGTTAAAAATGTTAATGGATCCAATGGGAGGTATAGTTATGACTAATGATGGAAATGCT
ATATTACGTGAAATAACAGTACAACATCCTGCTGGGAAATCTATGATAGAAATAGCTAGAA
CTCAAGATGAAGAAGTTGGAGATGGTACTACTTCAGTTATTGTATTGGCAGGAGAAATTCT
AGCTACTGCTGAACCTTTCTTGGAAC

185. >MSTRG.3671.1 Gene=MSTRG.3671
GCATCATGTTCTTCTAATACAAAATCGAATTTTTGGGCAATACTTTTTCTTCGGTCGCCGGAA
TTTCGACGTTTACACTTTTTTTTAAATATCTCCCTTATTGTTCAGAATATTAAAAAATGTTTCA
AATAAAAGTTGAATCGTTTCGAAAGGGCTATATCCCGACGCTCTCGTTTTTTTAGTAGGTTG
ACCAATAAAGAACTTTCAAGCACAACTTTATTTTTTTTACCGATAACCTGTGTTTTTTAATGC
ACCAATCGATAGAGCGTAAAATTTGCTACAAAAAAGTATTAACCCACTTATAGCGAAAAA
CCAATAGTTTGAAAGATATAGCAAAAATTAGAAATTTTTAGGAGGTCGACCTCCTATCGCA
TCATGTTCTCCTAATACAAAATCGAGTTTTCGGACAAAAATTTTTCTTCGATCGCCGGAATTT
CGACATTAATACTTTTTTTTAAATATCTCCTTTATTATTCGGAATATTAAAAAATGTTTCAAA
TAAAAGTTGAATGGTTTCGAAAGGGCTATGTTCTGACGCTCTCATTTTTTTAGTCAGTTCACC
AATAAAGAGCTTTTTAAGCACAACTTTATTTTTTTCACCGATAACATGTATTTTTTAAAGCAC
CAATCGATGGAGCATAAAATTTCCTATAAAAAAGTATTAACCCACTTATAGCGAAAAACCA
GTAGTTTAAAAGATATAGCAAAAATTAGAAATTTTTAGGAGGTCGACCTCCTGTCGCATCA
TGTTCTCCTAATACAAAATCGAGTTTTCGGACAAAAATTTTTCTTCGATCGCCGGAATTTCG
ACATTAATACTTTTTTTTAAATATCTCCTTTATTATTCGGAATATTAAAAAATGTTTCAAATA
AAAGTTGAATGGTTTCGAAAGGGCTATGTTCTGACGCTCTCATTTTTTTAGTAGGTTCACCA
ATAAAGAGCTTTTTAAGCACAACTTTATTTTTTTCACCGATAACATGTATTTTTTAAAGCACC
AATCGATGGAGCATAAAATTTCCTATAAAAAAGTATTAACCCACTTATAGCGAAAAACCA
ATAGTTTAAAAGATATAGCAAAAATTAGAAATTTTTAGGAGGTCGACCTCCTGTCGCATCA
TGTTCTCCTAATACAAAATCGAGTTTTCGGACAAAAATTTTTCTTCGATCGCCGGAATTTCG
ACATTAATACTTTTTTTTAAATATCTCCTTTATTATTCGGAATATTAAAAAATGTTTCAAATA
AAAGTTGAATGGTTTCGAAAGGGCTATATTCTATTTATTTGTTTTTT

186. >MSTRG.3673.2 Gene=MSTRG.3673

TCGATCGTGGAAAGGATAGAAGAGAAAACAGTATCGATGTGTTTTCTGTGGTTGAATTCGC
GTGTGCGACGACTCAACAAGAGAGTTCGATCAGATTTTCGTTAAACGTTCGTTTTTCGCGA
GTGAGAAATTGTGTTGCGTGCGGAAAGAGGACATAATCGTAGAAGGATAAGTGAGAAAGA
AAATGGGAAAAAAATGAGCAGCCTTTTCAATCTGTGACGTACCTACGACGGAGCGATGAA
AACGGCATAGCAACGGCAATAGCTCGTCCCAACGTCCAACGGGCACGTATTATGTGAGGT
GAAACTTGCAACGAGGGAAACGAAGATGTCCGCCATCAGCGTGGCTGCATGGCTGCTCAG
CGCCGTCGCTTTCGCCGCGATTAATAACGGTAAGTTTCAAAGGATGGTTACACGCCTTGCT
ACGTAAGTAAATATATCGAATATAATATTGGAAGAATAAAATTCATAAAAGTTTTTGAAAG
AGAGTTTTTTTTTAATTCTTTTGCGATGATCCAAATATTTGCAATATAAAAATTAAAAAAAA
TCAAAGTTAATCAAATATGATAAGTTTACAGCTCAGGATACTGCATTTTAAACAATTTATAG
GATAATTGAACGACTCGAAAATTTATCTTCACGATCTAATAAAAACTTAATGCGATAGGAA
TACGATAAAA

187. >MSTRG.3673.3 Gene=MSTRG.3673

GATGGTTCACCGGACGACCGTCGTTGGGGCGCTGCAACCCGTCAGGACGTCAGTTTGCTGT
CGTACCGCGACACGGTCGGATCTTATTTCGTAATCATCACGTACACGTACGTACACGTATAC
ACGCGCGTGAGAATTATTAGTAAGAATTATTTGTTTCGGAATCGTAACGGTTTTCTCGTGAT
AAATAATCGATAATAAACGGGTATTTAACGTGAAATGACACTAATCACGTGATCGGTGATT
CGCGATACGAAGTGAGAAAGGATCGTTTAATCTCTAGTTTCGAACGTTTTTTTGTTCGCGAT
TGAACACGTTCGTTGAATTTTATGTGCAAGTGTTCGTGTTAACTCGCTTAAATTCTGTGTATA
TGAGGTCCAGTTAAGTTTCGTGTCTTCGTAAAAATAAAATATATACGATTCAAGGACGTATG
CTCTCGAGTGTTACAAGAGAGAGCACAGTGTTAAAAATAATATATATTGAAACTTGGAAGG
TTAAGTGATACGTTTGTTGACATCTATAACATTGTTGGAGAATGTTATTACGTGGAAATTCG
TGGAATAATAATCATTAGAACGCATATTCACAAGCAATATTTGATTGAGGAGAAAGATTTG
AATTTTTGTTTCGCGTTTTATCCACCGTAGCGTTGTGGGCGATCGAGAATCGAAAGTGCGCT
GCAATCGATCTATCGATAGAATCGAAAGTTGAAATGTTCGCGAAAGAACGTCGAATATGC
GAAACACGAGTGTACAGTTAATCGAGGCATGTACGCGTAACCGTCATGGGTCGTTATCGAA
AGAGTTTCAATTAAATTTCTCGTGAGGAAGGTCAAGTGCGCTGTTCAGTTTTCAGTTTTAATT
AAAATACCCCTGAGATTCTCTATTGTCGTGCTTGTGTCTAAAATTAATTAACGACGGGTAAA
GACAACGGGCAGGAGAAGGCGAATAAGCAAGATTATGACAAAGTGCGAAGAAGGTAATA
ACATAACCTATAAAAAATCAATGACCATCGTGCGACAATAGAATTGACGGGAGTGTTTTCC
GTTCGAACGATTCGATATATCGATCGTGGAAAGGATAGAAGAGAAAACAGTATCGATGTG
TTTTCTGTGGTTGAATTCGCGTGTGCGACGACTCAACAAGAGAGTTCGATCAGATTTTCGTT
AAACGTTCGTTTTTCGCGAGTGAGAAATTGTGTTGCGTGCGGAAAGAGGACATAATCGTA
GAAGGATAAGTGAGAAAGAAAATGGGAAAAAAATGAGCAGCCTTTTCAATCTGTGACGTA
CCTACGACGGAGCGATGAAAACGGCATAGCAACGGCAATAGCTCGTCCCAACGTCCAACG
GGCACGTATTATGTGAGGTGAAACTTGCAACGAGGGAAACGAAGATGTCCGCCATCAGCG
TGGCTGCATGGCTGCTCAGCGCCGTCGCTTTCGCCGCGATTAATAACGGTAAGTCGGTGAA
CAAGCAGTCGGGACTTTTGACAAAGTTTCCTCGACGGGTTCTAACCAAAGGGTCCAAGCAA
GTGAGTAACGATCGGATTAATATTAAAGCGATATTACCTCGTTTATTCGAGATTTTGGTTCG
AATAAAGTCCAAGTTTGGAAACTTGTTCAACCGAAGACGGATTTAACTTTTCAATTACATTA
ATTCGCGTTAGATACATAATTCCTT

188. >MSTRG.3687.1 Gene=MSTRG.3687
CATTAAGTTGTTGGCAACCCTGAAGTTTCCCAGGGGGAAGGGTTAGTAAAAATGGAGTGTT
ATACGGTACAACGTGTCTTCATTATTGAACAATATTTTAAAAATAATTAAAGTTTGGTGGCC
GCAAAATAAGCGATGAAGCACATTTTCACTTCGATGATTTTGTTAATCGCCAAAATTGCCGT
GTTTGGGGTTCGGGGAATTCACATGTGATTAGCGAAAAACAAATGCACCTACAACGTGTCA
CTGTTTGGTATGCATTTTGGACAACAGGCATCATTGGATTATACTTTTTTGAGAATAAGGCT
GAGCAAGCAGCAACTATTAATGGTGCTCGATATGGCGACATGATAATACAGTTCTTTCTAC
CGAAATTGAGTGATATTGATATGGCCAATATATGATTTCAACAAAACGATGCCACATGCCA
TACAGCCAATAAAACAATTCAATTACTGCATGAGATATTTCCTAGTCATGCACTTTCGTTTC
GGTGATCAAAATTATCCCCCTAGATCATGTGATTTAACACCATTAAATTTCTTCTTATGAAG
TTATTTGAAGTCAAAGATCTGTCAACAATCTCACAACCACACGTGCATTACAAGAGGAAAT
TAAATGTTGCATCAACGAAATTCAGCCACAATTATGCAGAAAGGTCATGAAAAATTTCAAC
GAAAGGGACTTAGTATTTAGCAAATTATTACACATTATATATAATTCTTATTTTTATATAAT
TAATTACATATAACTATTTATATTTCATATTTATTCTGATATATGTATAATTATACATTATGCA
TATAATTAAAAAGAGCTTTGCAAAGAGTTTTTGTGGCAGTTTCTAAAATTTCTTTTTTATGAA
AACTTATACTAAGTCGTAGAAAATTATTAGATTTTTCTAAATATGAAAAACGTATCCCTGGT
ATGGCAGATACTTTAAATGTTTCTTGACACC

189. >MSTRG.3697.1 Gene=MSTRG.3697
CTTTTTTATAGGAAATTTTATGCTCCATCGACTGGTGCATTAAAAAATACACGTTATCAGTA
AAAAAAATAAATTTAAGCTTGAAAGTTTTTTATTGGTCAACCTACTAAAAAAATGAGAGCG
CCAGACTATAGCTCTTTCGAAACCATTCAACTTTTATTTGAAACATTTTTTAATATTCTGAAT
AATAAACGAGATATTTAAAAAAAGGTATTACTGCCGAAAATCCCGGGACCGAAGAAAAAT
TTTTGGCCAAAAACTCGATTTTGTATTAGGAGAACATGATACGAGAGGAGGTCTACCTCCT
GATAATTTTTAATTTTTGCTATATCTCTTAAACTAATGGTTTTTTGCTATAAGTGGGTTAATAC
TTTTTTGTAGCAAATTTAACGCTCCATCGATTGGTGCATTAAAAAATACACGTTATCAGTAA
AAAAAATAAATTTGAGCTTGAAAGTTTTTTATTGGTCAACCTACTAAAAAAATGAGAGCGC
CAGACTATAGCCCTTTCGAAACCATTCAACTTTTATTTGAAACATTTTTTAATATTCTGAATA
ATAAACGAGATATTTAAAAAAAGGTATTACTGCCGAAAATCCCGGGACCGAAGAAAAATT
TTTGGCCAAAAACTCGATTTTGTATTAGGAGAACATGATACGAGAGGAGGTCTACCTCCTG
ATAATTTTTAATTTTTGCTATATCT

190. >MSTRG.3703.1 Gene=MSTRG.3703
ATTAGCTAATACATATATATATATATAATAATATAGAGAGATTTTAACGATATATGTTTCCT
TTCGCCGCTTCAAAGAATAGACATATTCAGGAAGAAATTAAAAGTTTCCAAATTTAGCATC
TTGAGAAATTAAGATGAAAAATCCTAAAGCAGAAACTTTTACTTGTTCATTGTTGGAGATG
GACTTTATATCAGAGTTAAAATTGTTGTAAAACATATTCAATTTTTTCGGCACGTTATTGAAC
GCTTTCAAATCCACGATTTGTTCCTCGTTCGAGAAGTTCAATATTGCGTAAAGAGATCCATT
ATCTTCGGTTTCCCTAGAGAATGCGAAAACATTGTCGTTCAGCATCCTCGTATTTAAATTGG
CCTCTTTAAAGTATGGCGATTTTTTCAGCGACGCAAATTTCTTGAACATATTGAAGAACGAG
TTCTTGTCCTTCTTTTCAGCAGCTAGATTGACAGTCTTGTAATTTTCGTTGACACGAAGCCAG
GTATTAGAGCTTGAGGAAAATCCAGCAGAAACTGAGTCGTCCCATTGGAATGGCGTTCTCG

CGGGATCTCTCGACATCGTTTGATAGTTTTCTTTACCGGCGCCGCATCCCTGCGGATCCTGC
GTATCCTCCCACGAGATATAAGTATCCGACATACCAATTTCATCACCGTAGTAATTCACGGC
AACACCTGGCAGCAAAAGCGACATCGTGGTGATCATACGGGCCTTCTCCTCTCCAAATCTC
GACACCAATCTCAATTGATCGTGATTTCCGGGCACCCAGTTAGGAATACCACTTGGTGGCA
TGTACGTCATCCAATTATCGACCAATTTTTTGAAGTCTGATGAATTTGAATCCCTAGAAACA
TTCTTGATGAATGCAAAATTGAAGGGAAAATCTGCTCCGTAATCGTAATATTTCATCGTCAT
CGATAAATTCGTGTATGCCTCGATAAGCATGTGTTTTGGTTGCGGGAATTCGTCTAACACAT
CTCTAAATTTGCGAACTACATTGTAGGTTTCTGGGATATCGTGAGTGTAGATCTTGAGAGTG
TACTCGGTTTTATTGGGATCATTTGTTTCACCTGATAGAGGTTCGTCTAAGAATCGCATGTCT
TCGCAAATGTAAGGCAGAGCATCTACTCTGAAACCATCAAATCCCCTTCTCAGCCAGAATC
TGAGAACATTTTGCATATCATCCAGTACAACTGGATTATAGTAATTTAGATCTGGTTGTTCT
GGTGCAAATTGATGCAGATAATATGCCTGTCGTTCTTCCCGCCACGACCAAGCTGATCCAC
CAAACACGCCTACCCAATTAGTTGGTGGAACACGTTTGCCATTTACAATTTTTCCTGGATGC
CAAATGTAATAGTTGTTATAAGGTTCAATGTTTTTCAAACTCAACTGGAACCATTCGTGTTG
ATCAGATGTATGATTCGGGACGAAATCCAAGATTATCTTCAATCCTTTCTCATGTGCAGCAC
TGACTAGATTATCTAAGTCTGATATGGTGCCAAATATGGGATGAACGTCGGTGTAATTCGA
AATGTCGTAACCAAAATCGACCATAGGGCTTGGATAAATAGGGGATAACCAAAACATGTC
GACCCCCATTTCGAGAAAATGATCCAATTTTTCTTTAATACCTTCGATATCACCAATACCAT
CTCCATTGCTATCCTTGAAGCTTCTCGGGTAGACCTGATACACGATCAAGTCCTCCTTCAAG
TTTTCAGGGAGCGGCTTCCATGCTGCGTCCACAATGGACAATGCCATAAGGCAAAATACGA
TTACTGCCTTCATGCCTGTTTAACACACAAAACGGATCGTTGCATCTCCGCCGGATCGAAGA
GCCACAAGAGACGATATTGCAGTGTCCTCACCTGGTGCATCTCCGAACGTTAATGCAACTA
CGAGCTGCACTGGTACAACATCGTCTCGATTGCACAATCCTTCCTCG
CCAGGTCGAAATGGCCAGCTATCGAAACAAGGTACATTAACGATAGTACGCCGGAGTTCT
GGCAAGAATAAGAGCATTGGCGGAAGTTTCGAAAGTTCTCCGCCCCCGCACAGTCCTGAC
ACTCTTTCGTCAAGTCAATCCGTGGATCTCGACGAGATCCTTGATAATGTCGATCTAACGAC
AGACTCGCTTCCTGCCGTTGACACCCCCGACGCTTGCGACAAAGCTGCTCTCAGATTGAGA
TGTTTACTGAGGCAGCTTCAACATGGCGAAATATCAGCAGAATTACTTCAACGAAATTTAC
ACTACGCGGCACGTGTACTCGAAGCTGTCTTCCTCGATGAAACCAAGGTGAATTCAGGAGG
GGTTGAGTGCTCTCGGTCATCGCGTAGGGGTACGGGCCTGTCATCTGCATCCCTTGGGAGCG
CCTTGGACTCGGATGGACAACAGGGCCACGCGGTGGTAACCCAGAAACGGAAGCT

191. >MSTRG.3716.2 Gene=MSTRG.3716
ACTCAGAATAAAATATTTTATGAAAGACGCAAGCGCTATGATATTAAATTCTTTAATTTATT
TGATAAGAATATTTCTTATAGTTATAAATCTTACTTTATGTAAAAATAGCAGATCGCGAATG
GAGATAAATAAGAATAAATCAAGAGTAGATTTTTCCGGGAATGTAATTGAAAGCGCCGCG
TGTTTAGTTGCAGCACTATTGAAATTGGGCTTTGATCTTGATTGGATATAAACCAGCAGAGA
TCTGTAAGCGAGGATCCACTAAGAATGGCTGCCGTTTTGAGAAGATTCAGTGCCGCGCAGA
TCACGGTGATCAGCAAAACTAAACTACCGTCATTTATTATCAGCCGGTAGGCGATCAAGAT
TCAAAAACCTTGAGGAGCCCTTCTTTTTTCATATGTTTCGTACATAGACTACGAGCTAGTCTC
TAAACAGCGGCTTTTTAGGGCAATAGCCACATCGTATGTGTTGTAGATCTTTTGCAAGGTCA
TCGATCCGACAGGATATCAGCGAAGGATGGATAAACAAGCTTTCCGTAAGAAAAATCGAG
CCAACAAAAGAGTCTCATTCAAGGATGCTATCAGATAAAGGAGTTATTTACGCTTTGCATA

CGCACAATATTCGACCTGATTCTATTGATAACTATTTAACTAATTATGAAGAGATTGTTAAC
ATTATAAACTCCAAGAAGTCTGAATTGAAATTGGAACTAGTTGGTTCATGGACTGTCGTAG
CTGGTGATATCGATCAAGCTTTACATCTATGGCAATATTCTGGTGGTTATGATAGCGTTGAT
CATACACAAATTGAATTGTCTAAAGACAAAGCATATCAACAATTATTCAAAGAAAATGGC
AATTATTTACGATCACGTTATTTACAGTATTTGTTAGCATTTAGTTATTGGCCTCCTCTTACA
AAACGAAATGATTCAAATATATATGAAATACGCAGTTATAGGCTACAACCTGGTACTATGA
TAGAATGGGGCAACAATTGGGCAAAGGCTATAAATTATAGACGTAACAATAATGAACCTT
TTGCTGGTTTTTTTTCACAAATTGGTAGATTATATAATGTTCATCATATTTGGTGTTATAAAA
ATCTTCAAGCGCGAATGGAAACTCGTGAGAGTGCATGGAGACTTCCAGGATGGGACGAAT
GTGTTGCATATACTGTACCATTAATTAGAGAAACACATTCCCGTATATTGAGTCCAACTAGC
TTTTCACCAACA

192. >MSTRG.3749.1 Gene=MSTRG.3749
AGTTGCTCCAATCCCACGAAGATTTTTCGGTTAATCTTTGTATGACGCTTCTTGTCATGTTTG
AATTATTCGAAAGTTTTTTCATGTATTATATTATTTTTGTTGAGTTAAAACTTTATCATTAAAA
CGAATTATTCAGTGTGAATTTGTATTGTGTTTGTTAACACAAATAATTATAAAGGATTGGTT
GAAATAAAAATAATATCGTTTAACATACCATGGACGCATTCACTCAACGAATGTTGGAGCG
AGCAAAGTCAAGAAGAGAAAAATTAGATATTAAATTATCAAATGCTGGACATGATGTAAA
AAAAAGACGTAGTCCTTTGAAGGATGCAAATGCTATTTTAGCTCAAGCT

193. >MSTRG.3803.1 Gene=MSTRG.3803
ATTCGACTAATCGCATTGGGTGTTCGACCACGCTTTCCGCTCTTCGTCTCCTTGTGTTACTTG
TGGACATATTTACGTTCTTTTTCGAATTCTTATTATTTTGTGATTACGAAAATGATGTGCTAG
CTTTAGAATTTACGTATTTATTGTAATTATATTAATAACATTTTGTTGGATATATAAAATCTC
TGCGCATCGATACTTTCGTGAAACGTCAACCTAACCCTCAATAAGTGCAGGAACGAATCTG
AAATTCCATTATGATATATAAGGTTTTCCTCCAGGACGGCCGAAAGAGGCAGTGGAGACAG
GGATGGACGATAAAACTAAAATAATCTATTGCTACTCTTTGTAATAATTTGAAGATTTGATT
AATTGGAGATTTGATTATCGAAATTACCA

194. >MSTRG.3801.2 Gene=MSTRG.3801
GAGAGGCACACACAGAACCGAAACGCTATTGCGCGAAACAATTTCCTCCGTTTCTTGCTAC
GATTCTGTTTCACGCATAGTTCCGGCCGTTTCCCCGCGTGCTTTTGTTTTATTCGAAAGCAAA
AGAAAAAAAAAGAAAACATAAGAATCGTGCGTGTTAAATATGTACGTGTGAAAATATTG
TACGAAGAAAAAAAATTATAAAGATGCTACTCAGAACATGAAAAAAAAAACAAGAAGAG
GTTAGAATGGAAACTTGTGAGTGTGATGAAAATACAATTAATATGTAAAAAGAATGATGAT
GAGTTCTCTTTGTCGAACGAAGAGAAAAAAAGCACAAATGAAATAAAAGATAAAAAGGC
ACGTAAGGAAAAATAGAAAAGGATCGCACAGGTGAATAGGCGTTAGAAAATAAAACAGG
AGTGCCAAAATCATCCGTGACTTCCGTCCCACTTGGGCAATAACAAGTACAGCAACTTGGC
GAGAATACAAATGTTTGCCATTTGTCCTCCAATAATCGTAAGGATTCTTAATCTGTCCGTTA
TATCGATCCTTTTTTTGTATTGTACTCGAAAAATGATCAATATATCCATTTTCACCAAGATTT
TTTGATCGAAAATATCGTGATCATATATTAGTAATAAAAACAAAAACAAATAAATACTAAT
CATTGAAAAATAGAAAGAAAAAAGAAAAGATTATTTGAAACATAATTTTACAACAAAGAT
CGTTACTTTCGAGCAATTGATACAAATAATCGATCTGAATAAAATAATATTGACACTCGCTA

TTAGTGCCTGTCGTTTCGTCGATTCAGGACAGAGTATTTCGTCGAGAAACCTCTAATTCCATT
CGTCGAACTGACTAAAATGAGGTTGTATCCGATTCTCCATTTATATATTATGGATGTTGTTTA
TTAAAAATAATAGATTGGCCGTAATTAATTTTGACGGCTAATTAAACTATAAAAAAAAAAT
ATTTATTCAGACGACTTGCCAGTTTCGTTGCACAGTATACACGATGTAGGTTCGAGCGAAAT
AAAAAAAAAACATATAAAAAAATAAATCATAATAATAATGGAAAGAAATTTAGAGATTAT
GGATTAAATGGATGAGTTGCATTAGTTATTTGAGAAGTTTCATTGAGAAGAAATATCGATGT
ATATATTTTAAGTTTGTATTATAATGCCCCCACTTAATTTGTATGAAATGGAGAATTTCAAAT
ATTAAAATGATAAAAAAAAATCTTTGTTCTTTTTTTTATTTTCCTATTTTTATAATTTTTTGTCG
TCAAATGTTATATATCCCC

195. >MSTRG.3801.3 Gene=MSTRG.3801
GAGAGGCACACACAGAACCGAAACGCTATTGCGCGAAACAATTTCCTCCGTTTCTTGCTAC
GATTCTGTTTCACGCATAGTTCCGGCCGTTTCCCCGCGTGCTTTTGTTTTATTCGAAAGCAAA
AGAAAAAAAAAAGAAAACATAAGAATCGTGCGTGTTAAATATGTACGTGTGAAAATATTG
TACGAAGAAAAAAAATTATAAAGATGCTACTCAGAACATGAAAAAAAAAACAAGAAGAG
GTTAGAATGGAAACTTGTGAGTGTGATGAAAATACAATTAATATGTAAAAAGAATGATGAT
GAGTTCTCTTTGTCGAACGAAGAGAAAAAAAGCAATATAAACGGAGTATGTATAAAATGT
ATTTTAAGTCTGGACAAAAAAATCAATTTACACTTACAATTTACAGTAACGAGTAACAATA
ACTGATGTGGGTTGTGCATAAGAAAACAAAAACAAAAAGAAAAAAAAATGAAAAGCGTA
TATATACGGTAAACATGACGATTCGTGATAATGGAGAATCGGATCGACTCAACGATCGAAT
TGCGCACGTGCTGCGTGTAGAAAACAAGTTTTCGTCCCAGTGCGGATTCACTTCTTTCCTGG
CTGTTCTTTTTTATTTTGTTTTGTCATATTCTGCTTATATAATTAAATTATGTGGTTCTCAGTGT
AGTTAATTGTATTCCATTCGTATTCCGTTCGGTAAACTTGCAT

196. >MSTRG.3813.1 Gene=MSTRG.3813
AAATAAAATTGAGATAGTTAAGCTTTTTATAAGTCCACAAAAAATTAAGGGCGCCAGGATG
TACCTCTTTTAAAACCATTCAACTTTTATTTGAAACATTTTTTAATATTCTGAATAATAAGCG
AGATATTTAACAAAAAGTATTTCTGAAATTCCCGCGACCGAAGAAAAATTTTTGTCCAAAA
ATTCGATTTTGTATTAGGAGAACATGACACGATAGGAGGTCGACCTCCTAAAAATTTTTAAT
TTTTGCTATATCTTTTAAACTATTGGTTTTTCGATATAAGTGTGTTAATACTTTTTTATAGGAA
ATTTTACGCTCCATCGATTGGTGCTTTATAAAATACACGTTATCAGTAAAAAAAATAAAGTT
TTGCTTGAAAAGTTGTTTATCCGTCAACCTAGTAAAAAAATGAGAACGCCAGAATATAGCC
CTTTCGAAACCATTCAACTTTTATTTGAAACATTTTTTAATATTCTGAATAATAAGCGAGATA
TTTAACAAAAAGTATTTCTGAAATTCCCGCGACCGGAGAAAAATTTTTGTCCAAAAATTCG
ATTTTGTATTAGGAGAACATGACACGATAGGAGGTCGACCTCCTAAAAATTTTTAATTTTTG
CTATATCTTTTAAACTATTGGTTTTTCGATATAAGTGTGTTAATACTTTTTTATAGGAAATTTT
ACGCTCCATCGATTGGTGCTTTAAAAAATACACGTTATCAGTAAAAAAAATAAAGTTTTGC
TTGAAAAGTTGTTTATCCGTCAACCTAGTAAAAAAATGAGAACGCCAGAATATAGCCCTTT
CGAAACCATTCAACTTTTATTTGAAACATTTTTTAATATTCTGAATAATAAGCGAGATATTT
AAGAAAAAGTATTTCTGAAATTCCCGCGACCGAAGAAAAATTTTTGTCCAAAAATTCGATT
TTGTATTAGGAGAACATGACACGATAGGAGGTCGACCTCCTAAAAATTTTAATTTTTGCTAT
ATCTTTTAAACTATTGGTTTTTCGATATAAGTGTGTTAATACTTTTTTATAGGAAATTTTACGC
TCCATCGATTGGTGCTTTAAAAAATACACGTTATCAGTAAAAAAAATAAAGTTTTGCTTGAC

AAGTTGCTTATCCGTCAACGTAGTAAAAAAATGAGAACGCCAGAATATAGCCCTTTCGAAA
CCATTCAACTTTTATTTGAAATATTTTTTAATATTCTGAATAATAAGCGAGATATTTAACAAA
AAGTATTTCTGGAATTCCCGCGACCGAAGAAAAATTTTTGTCCAAAAAATTCGATTT
197. >MSTRG.3825.1 Gene=MSTRG.3825
AGACAAAGGACACGACGAGGCGGGAAAACGCCTCTCTCTTCTTGAGAAAAATTCGTCCAG
AATTCGTGCAGAAGCAAGGACGCTTTTTTAAAGGCGATGGATTGTATTACGACGACGATGA
GCGAAGGAAGGAATGCGTGAGATTTGTTCAACAAATGGGAAGCTTGTTGTGCAACAGCTTT
TTGCCCGCGCAAGAAAAAGCCAGGATATGTACGGATCGAACCGGAACTCCATTGTTTTTG
AAAACGGATTGGAACCGGTGCTGCTCCAAGTAATTCGTGACGAATCCCTCTTTGTGTACCG
GATAGGTATACAGATTCAATTGTGCCCGGTTGACAGACGAAATTGGCCAACCTGAGATCTC
CTCCGGATCCGGTGCGCTCTCTCTTTTCAG

198. >MSTRG.3891.1 Gene=MSTRG.3891
ATTCGGAGAACATGATATGACAGGAGGTCGACCTCCTAAAAATTTTTAATTTTTGCTATATC
TTTTAAACTAATGGTTTTTCGGTATAAGTGGGTTAATACTTTTTTATAGGAAATTTTATGCTC
CATCGATTGGTGCTTTAAAAAATACATGTTATCAGTAAAAAAAATAAAGTTGTGCTTGAAA
AGCTCTTTATTGGTCAACCTACTAAAAAAATGAGAGCGTCAGAATATAGCCCTTTCGAAAC
CATTCAACTTTTATTTGAAACATTTTTTAATATTCTGAATAATAAACGAGATATTTAACAAA
AAGTATTAATGTCGAAATTCCCGCGACCGAAGGAAAATTTTTGACCAAAAACTCGATTTTG
TATTAGGAGAACATGATATGACAG

199. >MSTRG.3902.1 Gene=MSTRG.3902
ACACCGGTCCGTACAGGATATTTCGTCGGCGCGTCAGTGCTGCAGTCGACAGTTTTTCCTTT
TTTCTGTGCAACATAATGTGACAATCGGAACGCACGCGTCAGTTCGTGATCAATATCGATTC
TCGCGGGATTTAACGTAGATAAATAAGTCGCAAGTGTCGATGTTTTCGGTTATTGTACTCGT
CGAACGCGTTACCACGTGATTTTTCTCTGTCAAGTTTGAACACGGAAAGAACCGTGTTACTT
GCGTGCGAATATCGTCGAAGTGAGTGTTTGATAATAATTTTCGGAGTGTGATCGTGAAATAT
ATACGATGCATCCGTTGCGGTGAGAACGGAGGGAAAAAAGAATTTGAGCGGGAGTGCCTC
GTAACCATTTTGTAAAGTTTCGTGTATCAGCCGCCGTCTCTGTGTGTGTCGCAGCTGCAGCG
TCCCTCGCGGCCGGTCAAGAGAGACAAGGAAGAAAAAGGAGCAAGGTTGCTGGTGGCGG
CGATTGACGGATAGTGGCGATCGAGCGGGGAGAACGGCGACCACCTTGTATGGCAGGTAA
GATCTTTTCTCAGACTGATGAGAGACAGTTCTCTTTGAGTTTGGATTTGGATTGTGCGTGCTT
CTTGTCTCATGTAAGTGTAATATATATAAATTGTTTTATCGCAATTATACTATTTGCATGGAA
TTGTAGGAGAATGTAGAAAATTGTTTCCTGTAATTTATAGTTTTTTACATTTTGAAAATGATG
ATGATCTTTTAAATAACAAAAGTAAAGATACGATTATTGTTTCATATTGTACGTG

200. >MSTRG.3951.3 Gene=MSTRG.3951
ATTTCTTCTATTAATTATTACGTATCGAAATATTATTAGCAAATACGCATGCGCAACTGTTAT
ATAATGATTATAGATATATAAGTAATTCTGCACTAAAGCATGATAATTTAATTTTTAGCATG
GTTTAAGGAGTTCCTTACATAATGTAAAAAAATGTCAAAGGGTCTTGGTTATTATTTTGGCC
CTGTGGTTGGAGTATAGCTATGGCAGCAGCACCAGGTGCCCTACCAGATTTTCAACTTTTAA
GTCTTTTTGGAATAGGAGCATTCATTATGCGTGGAGCAGGATGTATTATAAATGATATGTGG
GATCAAGATATTGATGGAATGGTAGCCAGAACAAAAGATAGACCATTGGTTACTAGAGAA

ATTTCACCTCTACAATCATTGATTTTCCTTGGAAGCCAATTAACTTTGGGATTGCTTATATTG
TTGCAATTAAATCTTTATAGTATTATATTGGGAGCAAGTTCACTAGTCTTAGTAATAATATAT
CCTGTTATGAAAAGAATAACATATTGGCCTCAATTAATTCTTGGGATGACTTTTAATTGGGG
TGCACTTTTGGGTTGGTCTGCTGTGGAAGGATCGTGTAATTGGTCTGTATGCTTGCCACTTTA
TACTGCTGGAATTTGTTGGACACTTTTATATGATACTATATATGCACATCAGGATAAAATGG
ATGATGTTATAATAGGTATAAAATCAACAGCTTTAAAGTTTGGAGACAAAAGCAAAATTTA
TTTATCTGGATTTAGTACAGTCATGATAACAGGACTAATTGCATCAGGTATATTAGCTGCAC
AGACTTGGCCATATTACACAGCAGTAGGATTAGTTGGTACTCATCTTGTTAATCAGATATAT
AATTTGAACATCAACAATCCTGCAGATTGTGCCAAAAAATTCATCTCTAATCACAGGATAG
GAATGATTCTTTTTACAGGAATTATTTTAGGAAATCTTATTAAAGTTTCTAAAAAAGATAGC
GATATTGTACAAGAATCAGAAAAAAAATCTATTCTCATTAA

201. >MSTRG.3974.1 Gene=MSTRG.3974

CTCGAGGTATAATCAACAGGTACAAACGCGATGTTTCATCATCACATTATATCGTACCTTCT
TTAATCCTTTATTGGTCCATATTTGTCGTTGACATTTCATAATGGCCGAATTCCATCGACGAC
GCCATCTGGATCTTCATTAAAAAACTAGAACGCTTTTAACATCGTTATTAAGCCGACCAGCC
AGAATATCCCTTGTTCTTGAATCCTGGTGGAACCATTTGCGATAATTAAATCAGGTCCTGTC
CAATTCGTATCATTATCATCGAGACATTCGAATTCACAGCATCGTTCGCCGACCCGAAACTT
CTTGCATCTGTAAGGTGGTGTGCAGAAGATGGATTGACACCACATTGGTTCCGAATGATAA
CAGACGCAGAGACTGCAAACCGCAGGTCCTGGTACATAAAGGAGACCGTGTCGAACTATC
GCTCCTTGGAAATCCGTGCAATTCTCCTCCAAGGCAGCGGTCCTCGAGGGAAGGAGAAGA
AGAAGAATAGCGGGCAGATAGAATACCCACACGAGTATTTTCGAGTAGAGGCTCGAACTG
CTCTTCATGGTTCTCGATTGAGTTAGCCACAGAAGATCAAAGAATGATGTCCACCAGGGAG
GCTAGATAGATGAAAAGTAGTTATTCCAACAACCACTGTGCATGCACTGCAGCGGCCAGG
AGTATGTTATCTCGAAAGTGCATCACGTCGATGTCTCGATCGTTCCTCATGTATCGCAGTCC
ATGTGTCCGAACAATATTCTCTTCAAATAACAAAGATCGAAGTAAACAATTCTCTTCTTTTA
CGGACGAAATCGGATATGAGAAAAATGATAAAAGAGAACAATAATCATACGATAAAAGT
GGCGATACTAAGTAGGCCGGTTTTCGGAAGAAGATTCACGAATAAGAAGCAAAAGATCAA
AGACCGACGCGACCATATGCCGTTGACGGGCAAACAGCGACTAACCGAAATGAACTCTGT
CGCAGTGTGAGCACGGGTTGCGTTCGCGGGTCG

202. >MSTRG.3990.1 Gene=MSTRG.3990

GGAATTTCGAAGGGGATTTTAAAAATTCGTTTTTTTCGTGCGTTCGACTGGTCGATTGGGCG
ATCTATTGATGCGCAATTGGGATGAGCTTCTCGCCTGTGCCAATCGCCGGAAGCGGAGCCT
CGACGGTGAGCACGCCATCTTTGCTCAACGAAGACTTGATACTTTCGGGATTGGTTCCCTTG
GGCAGCAGGAATTCGCGATTGTACTCTCTGTACACCGATTTACTCTCTGTCTTCTCTTCGTGT
TTTGCATGGACCAATAGTTTGTTGTCCACGGTCTTGACAACGATCTCCTCAGGCGTGTACTG
GGAGACGTCGAAACGAAGTTTCAGGCATTTGCTGTCCCCTTCGTCCTGAATGAGCGGGCTG
TTCAACCCGTCGAGCCACGCCGAGTCGTGTGGGGGGCTGAGAGAACTGTTTTGAGTGCTCT
GCGTTGTGGTGCTGGTAGTACTCTTAAAGAAGTTGTTGCTTTCGCGATTCATCAATTCGCTTC
TGAATCGTGACATCTCGTCCTCCATCTTTCTCATCTCGGCGTCGAAGCGTTCCCGTATGTTGG
AAAATTCGGTGTCTATTACGCTGAAGTCACCGAGCTTGATAGGAATATTACGCTTGATACC
ACTGTCAGCCATGATTTTTCTATAAGTCACACTTGTGTTTCCTT

203. >MSTRG.3990.2 Gene=MSTRG.3990
GGAATTTCGAAGGGGATTTTAAAAATTCGTTTTTTCGTGCGTTCGACTGGTCGATTGGGCG
ATCTATTGATGCGCAATTGGGATGAGCTTCTCGCCTGTGCCAATCGCCGGAAGCGGAGCCT
CGACGGTGAGCACGCCATCTTTGCTCAACGAAGACTTGATACTTTCGGGATTGGTTCCCTTG
GGCAGCAGGAATTCGCGATTGTACTCTCTGTACACCGATTTACTCTCTGTCTTCTCTTCGTGT
TTTGCATGGACCAATAGTTTGTTGTCCACGGTCTTGACAACGATCTCCTCAGGCGTGTACTG
GGAGACGTCGAAACGAAGTTTCAGGCATTTGCTGTCCCCTTCGTCCTGAATGAGCGGGCTG
TTCAACCCGTCGAGCCACGCCGAGTCGTGTGGGGGGCTGAGAGAACTGTTTTGAGTGCTCT
GCGTTGTGGTGCTTTTGAAGCTGTCGAACGCGGACCGCGTTGGCGGTGCTGCAGGATCGAC
CTTGTCCCAACCTTCGCTCTTCGAGGTCGTCGAGGTACGGTGTTCGCTGGTGCTCGTGTGAT
GTCGACTGGTAGTACTCTTAAAGAAGTTGTTGCTTTCGCGATTCATCAATTCGCTTCTGAATC
GTGACATCTCGTCCTCCATCTTTCTCATCTCGGCGTCGAAGCGTTCCCGTATGTTGGAAAA

204. >MSTRG.4038.1 Gene=MSTRG.4038
AGGGCTAGGCTGTGGTATGGTCGGGGTAACGCGGTGTCGCTTGTAAAATGACGTAGAGTGT
GCGAGGGTGTGCCGCGTGCGGTACGAGGTGTGTTAGCGGAAACGGTGTCGCGGTGTGTAGG
GTCGGCGCTGGCCGTAGCTATCGTAGCGTACGGGAAATTGGCTGTGGCTTCGCCAATGGAT
GCCAGGCAGGAGGAGACGCCATTTTGATATACGGGCGCACGTCAAGCGTTCGCTCGTAGG
GAAACGCGGTCTCGAGTCGGTCTGCCGCTTCGCGTAGGACCGCGCGGGTGAATTCGTCGGT
TCGCGAGGTTCACGAGTATCGTTAATACGAGTGCGTGGCGCAACGGATTCGTTGCGTTTCGT
TGACAAGTGACAGGTGAATCGATCGGGCCGCGAGGAAAGTCGCGTTGCGGCCCCGTGCCC
GTAACACGGTTCGCTCGGTTGATCGGTAGTGACTACTGTGTGTGGAACTACGTGTACATTCG
GATTTTTCCCCACGCGGGCGAGTTCAGTGCGAGGGACACGGTAGCTCGTGTATATTGGTGC
GCTGATCCGGGGTGGCGGTGTACGGCAGGGAAAAGCCGCTGTCTCGTTTCGCGCGCGCGAG
AGAGGCCACCGGCCGACACTTCGGGCCAGGACAAGACGACCCGATGTCGGGAGCGGGTCG
CGACCCCGTGTCGACCCGTGGTCGTCCTCGTCTTCGCATATCCATCTCTTTCATAGCACCAG
GACACTCCTGGCGTAAATGTGCTCTCATGAGTGCCCATGTGCACCTTTCTTTCTGTCTGCTGC
CCCTACGTGCCCTGTAAACCCAGAGCACACCCCGATTAGCACGCCCACGCACTCTATCACT
CGCGAATATGGCCAAGATCCTCAACAAGGACCCCGTCACCTACGAAAGGGAGAGGGAAA
ACTTCCTGAAGGATCTCCGTCACTTCCATGAGACTAGAGGGACTCCCTTCAAAAAAAATCC
GAAAATCAATGGAAAAGATATAGATCTGTACTTGCTGTATGTTGTCGTTACTGCTCATGGCG
GATGGATAAAGATACCATCAAGAGGGTTACCTGTTCGAATGAGGTGACATTTTGAGAACGA
CCGAAACTGTAAA、

205. >MSTRG.4046.1 Gene=MSTRG.4046
CCACGAGGCAACATCGCCAGTGCCATTCGTCGCCGCAGTGCGATACGGTCGATAGAAGAA
AGACGACAGTTAACAGTAGTGATTCCAGCTGCCGCAGCATCGCCATTCCATAATTTTCCCTT
GAATCGGCACTCGACACGCGTATCCCACCCCTTGTCGAGGGTGTCCACGCTTCATCTCGCTT
CGGGATGCCGTACGTGCGACCGTCATGACGCGGCTACGGTTGGATAAGAGTTTTACGAATG
TGGCGAGTTGCAAACATCCGATCGTTCCGTCGGATAACGTCCAGAAGTTATCACGATCACG
AGGGCGCCGGAAGTAGAGAGGAGGGAAGAGGAAGCGTGCCCGATTCCGCGTTTCAGGAA
CGTATTCGTGTGCCACGAGTGAACTAAAACTGTTTTCGCGCTCGGCCTCGTGGTCGAGCCGT

GACATTGGACTTTCTTGGAATCTGTTTACACGCGTGTCACCGCAAAAATCGTGGAATCCTCG
ACCTTTGTGCTCCTGTGCTCTATCGATCACTATCGCTGGATCGCAACTGTGAATTATACAACT
GACCGAGGATTTAATTCGATTCGTAATAATCGATGGCGTTGCATTGCATTGCATTGATTGTG
ATCAGTGGATTGGTTTTAAATTAAGATCGATTTTTTTTTTTAATTATA

206. >MSTRG.4077.1 Gene=MSTRG.4077
GGTAGCACGTGCTTGAAAGTTGTTCACTTCGTACGTTCAGCTTGCCAGCTTAATGATCAATG
CCCGCGATTCTTTGAAATCACGAGTTATTATTGAAATTGTGCACGAATTATGGCCCGTTCTT
AACCTAGAATCACGTTCGATTCCGAAACCGTTTCGTTGCGCGCGCGAATCGTCGCAGAAAA
GTCGTACGTGCGAAATGTAACGTAACACATCGAAGGAGAGTTCGCACGGGTCTAGTTTAAC
GGTTGTAATACAAAATCTTTCGATGTTATTTCCTTTGGTTTTTGCGAGTTTTTCTTGTCGATAA
CGTTAACAAGCGAATGGCTGTTCAAACGGATTATAACAAGAACGTATCAATAACGGCACG
ATCCAAGATTATAAACGTAAACACTATTACGCGTTTCTTATTATCTTGCGACAAATGTGTAT
TAGAACCGGTGCCTCCTTATTCGTCAGTCGAACTTCGGTAGTTTTTATTTATTGATGATCGAC
TTAATCGATGATAATTTGTTCGTTGCGCAAAATCTGTTTAATGGTGCTTCCAATCAATGTTCC
CGATAATATTCATTTATGCGTCGAATTAAGAAACGTTTGATTTTTCGTGAAGTGCGACAATC
GGTAAAATCAATTACAAATTTAAATTTATTGTGCAAGTGATTATGTGTTTATTTTCGTTGATG
TAAGAGGAATTTTGTTTTCTGTTTACTCGTCTGATGAGTGTTAAAAAAAAAATTGAACTTGT
CAAGCTCATAAGGTGTGAAGTGCATTGTGAAATGTAGAAGTGAGAAACAGAAATTGAAGA
ATCGTTCAACATATTTTAAGTGATATAATCTGCAACTGTTGGAACATACTCGAAACGAACTT
CGTCTTCCCAACGATAAAACAAGGATCAT

207. >MSTRG.4086.6 Gene=MSTRG.4086
GGAATGTTTATCGAGATAAGAAATAATGGGATAATCAGAATTAGTATTATTAATTAATTTA
ATATATTAATAGAATATTTTGTTGATTGTATTTTCAATTTCGAATTCAAATTTAAATCATATTT
TCGTTACATTTTTTTCTAAGATGTAGGTCACATAGTCCCCTTCTTGTACCTATCCTAGACAAG
CGAATCTAGGCTGTGAGCAGAGTACCGGGTGCATATAGGTATCGATCGTAAAACGGCGTTG
CTGCAGCCTGGCCTGGTGTACCGGCGAAACTGGGTTACCTACCTAACCTATACACATCATTC
GCGGATCTTGTTCCTCATTTGAACAAAGTCGAGTACGCCTCTCGTGTTTCTTCTCTGTATTTA
TGCGTTCGTGCAGTTTCGCCCACGCATGGCATACGTTCGTGTATCTTCGTGACTCGTCTCTCT
TTTCTTTCTCTGTTTGCTTTATCGTTCTGAATGTCGTTAAGAAATATACTATCTTCATCATCTC
TTTTCTTCATCTATAATAATAATAGAAGCATATTCGCTGTAATATATGCATTCTATGATCACG
GAGATGTTTTGATTCATTCAAATCAATGTTTATTATGGTGAAAAAACGATTTAAATTAAAAG
ATTTAATTTCACCTGCTTTATCGTGTTATTATTTATGTAGAAAAAGTGAATACAAATATACG
GGTGTGACAAAGTTAAAGCGAATAAACTATACGTGCGCATGCACAAGGCTGCACGTCACC
TTACCCTCAAGTGTTGTGGGGCTGCGCCTACTGCATTCATGCGAGTTGAAGGGTCTGCCGTG
TCGACGTCCATTACGCATGCGCAGGGTTTTGCACTCTGTCCACTGGTCTGCGCACGCGGCCG
TAGTTGCAGAGCGAAACGGGTAGGGGGCCGCGGGTCGCGATTCGTGAGTCGTGTGAGAAG
GACTAGCCTGCATCAGTCTTCGTCGCGTCCCTTGCAAAAGGGTACGCTCGTCTTCACATCAT
CGTTAATTTATTTCTCCTATTTCTGAGTGCACACGTTATATTGCTATAAACGCGTACGTTAAA
TATATATTAAGCTTCGATTGCAATATCGAATAAGTAAAATTCTTGAAAAAGGGTGATATTTT
GGAACTTATGGTTTCCGAGAAAGAATCTCACGCAACCTAGGAACAACTTGACGCCGTTTCA
ATTTTGTTTAATTTAAAATCTTTATTTGAAGCACTTTTTTTGAGCATATACGCGAGTGCTATT

AAACATTCATAGTGTTACTAAAAACGTGCGTGCGTAGTGTTGCGAGTAATTCGCTTCTTTAC
CTGCGAACGCTTTTTTTTATCGCGTTCATAAATCTCAATACAGAGAAAACTACATAGAACGC
AACTCGGTACTTTTCGGCACGCGGCCGAATACCTCCGATCATCAATCTTGAGACTTTTCTTA
GTGATCCAATAGTAAAGAAAGAAGGAATATGTGAAAAGAATTTGGAGAAATCTATTGTGA
ATCAAAATCCTATACTGCTGGATTTCATTCGAATACTAAATCATTGGTGGACATAACCTGTA
TGTGTTCTGTGAACAGGAAGTTTCCCGTAGCAAGAGTGGGTGGTACGAAGAGGGAGACGC
CGGTCGAGCCTTAAATATCGATTTTGGTATGGAAGAGGTACGATAGTCCGTCCGTTTCAACG
TGTCTCTTACTTTTGTGTATCTCCACGTTAAGAAGTTTTGACAGTTGAATACTAGAATACTTC
ATCAAGAGGAATCGCGTGCATCGAACGAGACAA

208. >MSTRG.4144.1 Gene=MSTRG.4144
TCTTTTTTACATTTTTAATTTTAGAAATAATTTTATACATTTTCATAGAATATGCTTCACGAAG
GTACGATCACTATTGCACTACAAAATTTTTCGTCCAATTTCTCCTTTCCGCTGCCTGTTTGGA
AATAACTTGAAGTTTTTTTTTTTATTCATTTAGGACACAAGTGCGCACCAAAGCATGACACA
CCGAGTAAGGGTCGCAATTACTGCTAGGTCTTCTGTCTTCCAAATAGCCTTTCTTTTCTTCAG
CAACTCCACGAGGAATGCGAATACTAACATTACGATTGGCCACACCAGCGCTGAAATCGT
ATATACTACTGGTCTCGCATTTTCCAGTCAGACGTCGCTCGTTGTCTTTTCCTCCGCGTGGAT
CATACGCTTGAATGTGCCTTAGGTGTTGTTTGCTCAATTTATCAATTGCTTTCTCAATTTCGG
CAATGCCATTTTCGGCACGCATCGATTTTGTCGAGAAGTTTGTATGTGCACCTGCACCATTC
CACGAACCATCCATAATTTTCGGGTCTAAACTAACAACCACACCAAACTCCTCAGCTACAC
GATGAAGAATAAATCTAGCAATCCATAAGTCGTCTCCGGCAGATATTCCGCTACACGGGCC
GACTTGGAATTCCCATTGTGAGGGCATAACTTCTGCATTAGTACCGGCAATTTTTACGCCAG
CATAAAGGCAAGCTCGATAATGAGCCTCGACAATTTCGCGACCAATTACTTTGTCGGCGCC
TACACCACAATAATAAGGTCCTTGCGGCTTAGGGAAACCATTTTTTGGCCAACCAAGTGGT
CTACCATCAAAATCAAGGAAAGTATATTCTTGTTCAATTCCGAACCATGGATCTTCATCTTT
TATAGCTTCCATAGCCTGATTACATTTGGAACGTTTGTTGGATGTAGTCGGGGTCATGTCACT
GTAGTAGGTATCACATAATACTAATTTGTTATTTCCACGACGAAAGGGGTCGTTGTAAATTG
CCACTGGACAAAGGAAAATATCGCTGTTTGCGCCATCTGCTTGGTAAGTTGAACTACCGTC
GAAGGTCCATATTGGTAAATCTGATGGATGTTTTGGTACGAAATCTACCGTCCTAGTCTTGC
TTCGCAAGCTTTCACCGGTACCATCAATCCAGATGTATTTCGCTTGAATTTTATTTTCCGGTT
GAGGCAGGTCGAGGTATTTATTTAACAAGGTTTTGTCTAAGGCCGCATTCGGCGATTCCCTC
AGTATGACACGTGACATTGTAATGCGCTATTATTGCCGGTCCAGCAATCGTGGAGAGTGTG
ATGTTCTAGATGATCGTTTGATCGATGGCTGTGGAGCGGAGTTGCGCGTCGCGTCTTATCGT
GTTATGTTGCAAAGAGTTAAAAGGCACTGGTCATAAAGGCAACGAGCGTCTGATAGTACG
GTCTGTGATACTACTGTAAGAGCGCGGCTGCGATATTTATGTAGAGAAGCGTTTCGGCGCA
CGCGTCGTTTACCCCGCGCTCTCGACTGTTGCGATTCCCCTCTTCTAGCTTGAAAGTGATTAT
CCGATGTGATCGTAGGTCCACTGGATTGTTACATGCCGGAAAATTCTTGTCTGCCTCTGTTA
CCATGGTACTTGTCAGTTACGAACCATCGACCGAAATTATTCACGATTCTTTTTCAATGACG
TAACTTGAACTTGTCCAAGGTTAAAATAAATCATCGCTGATTATCGTGTGGAAGTTTTAACA
AACAATTTTTCCAATCCTACTAGATGAATTTTCAAAACGGAGCAACTTCAAGAAATAACCA
GATATCGATAAATATCGATAAATAGGTAACTAGTGCAAAATATATTTTTCCCTATTAAGCG
AAAATAAGATTATTAATGAAAAAATTTTTCTTTTTTATCAACTGTTGACAATAATATTCAT
GATGAATTTAAATTATTGTACGATC

209. >MSTRG.4210.1 Gene=MSTRG.4210
GAAAGAAAAATGAAAATATTCCATGAAAAATAATGATCTGTAACAATATATAAATTCATG
AATGATCGGGAAAGAACATTTCGTTCGATTGTTCCCAGACAAGTTGGATCAGCGTGAAAGA
AAGGGGCGGCGCGAGGAACGCGGTTGAAACGCGTGCAATTATCCCATTCATTAGAACTCT
GTTAACTGGCATCACTTCAAAGGCTGTCTGGTCCGTTCTTCTTGCGTTTCCGCAATAGCTAGT
TCACGTTCAAGGGCATCAACGGCACGTATTTGTCGCTTGGTCGGAATTTGTACTGGGAGCAC
AATCGGTTCGATACCGATTCCGTGTAACCATTTCTCTGCCACTGTGAACAGGGACGTCTCTC
CTTAAAACAAGTTACTTGTATCACATTAAAAAAAAGAGCACCAAGAGTATTCGCGACTTCC
GAGTTAAGATTCTGCAAACATCTACGGAACTTCGCATCGCAGTCACAGTGAGACCGTGTGA
AAGGCGAGTTGTTGCATATACCATGAATGCACTCCTTAGGCGATATAGTGATAGGGCAATG
GTCGTGCTCTCTACAACATGCATCCTCGGCTGCATGGTGTCCGAGTTCATCGTACGACTTTG
CTAAAGTACCTGGGCCACACCATTTTGTGCCTGGATAAATCAATCCACCTCCTGCAGGTTGA
TTCTTTCCCTTGTCTCGAGAAGCTTTACACGTTTCTTCCAAATCTGCTACGAGATTGCTCGAC
ATTTGACGGACTTTTTTTGGATCAGCACCAAGAACCATTTTTTGAATAACACCGCGATCGGT
GTACAAGGCACAGAAGGGCGCATCCGCGTTTAATTCCACCATTCTTGACATAGTGGTATCT
GCCACGAGAACACTGGCCTCAGTTTCATCCCCCCAGAGTGTTGGTATGAGTAGTAGAAATA
CTGCAAGCATATTGAAGTTTTATAACTCTCGTGGATGATGATCAATAATTTTTCCTGTCTTCA
CAGGATTATGATCTTTCAACAAAAGAATTTTTGTGATTCCACAGAATTGTGAATCTCAAAAT
AGAGTGGTTGTGCAAAATTTTTTTCTAAAAAAATTCGCCTTATCTCTCCTCAACGGCCACTA
TCAAACTGATCGTTGAAACGGGTCGTCAGGATACTAATATTATGTGGCATACATATATGAA
CCCGTGATCCAGATTACACGGGGGAGATTCCAAATCACGTGATATGACACATGTTTGCAAC
CGTACAATATATAACTTCTGTTGTAAATAGATCGAATTAATTTGTCGACATGCTGGCGATGT
TTTTCCTTCCAGAGATTTTGCAATCATTTCATTTTAATATCTCTCGATACGAACCAATTAAAG
ACAGAAGTAAGAAGTGTATTTTCTTATTATTAAATATCGATGATAGCTGCGAACTAG

210. >MSTRG.4216.1 Gene=MSTRG.4216
CGAGACAAAATGGTCGACAGTGATGTAAAAAAAGGAGAATAGCAAAAATGCTCAACGTCT
GATTGAGGTTAATGTTTTTTTCAAAGTGAAATATATATATGAATAATCCACTTTTTGATAAA
ATACTATTTGATAATTATCGATAAATATAATTATGGCGTCTAAAGTTACTTTTAAAATAACT
CTGACATCTGATCCGAAACTTCCTTTTAAAGTACTTAGTGTACCAGATAACACTCCGTTTAC
TGCTGTTTTAAAATTTGCTGCAGAAGAATTCAAA
GTATCTCCATCAACATCTGCAATAATCACAGATGATGGAATAGGTATCAATCCACAACAAA
CAGCAGGAAACGTTTTTCTTAAACATGGATCTGAATTGAGATTAATTCCTAGAGATCGAGTT
GGATATGGTAGATAATTTGCACAAATAAAAATTCATTGTTTATAAAATTTTCATTTATATGT

211. >MSTRG.4241.2 Gene=MSTRG.4241
CCCTTCTACAAAGCATTTAGCTCGCGTACTCAAGTGATTCATAAGCGACCGCTTCTCGTTGG
CTTCTCAAGCGTCTCTCTTTCTGTTCCCGGCCACTCTCGAATTCCTTCGGCACGAGAAGGGC
AAAGGAAAAGAGTTTTATCGAGCTGCAACGGCTAGAGCAACGTAGTGAGTCACTCTCGGT
GACTGGCTCGTTGTAATTCATTGCGATGATTGAACCCCCACCAACTGTTCCTCGAGTACGGG
TCCATTCACAACGAACGAACTCGGTGACCCTAGAACCAGCTGTTTTTCCTCGCTTGCACTTT
CGATTGATCAGAAAGCTCGGTGGTGGTGATTGAGTTCCTTGTTTATTCATTTTTTCCTTCGTA

TGGGTGCAATTAAGGTGGCGGATTGAACGATTGAGTTGTTCAATGTAATTTAAAGAGGATA
GAAATGGTATGTATATATTGGGATAACAAATTGAGTTGTTGTAATTAAATTGTTCAATATGA
TAAATTGTTTATTTTAAGTTGTTCAATGTGAATGTAGTTTAATGTTGTACTACATGGAAAGAT
GTTTAGAATGAGATATAGATAGATAGTTATGTTTATAGAAAATGGAATTTAGTATATTTATT
GACATAATAAAGAAATAATATTCATACAATGAGGAG

212. >MSTRG.4279.5 Gene=MSTRG.4279
CACGAAGATCACGGAGCACCAGTAGACGGCAGCACTGCGGGCCGCGCGTATCGCAGTTCG
TGTCTGATCATCTGCGCGCTTTTGTCTCCACACTGGTCGACTTGCCGTCGTCGTCTCGAGGGT
GAACGTGCCCCCACCCTCTCTCGGCTTACCCCACCCCGAAGATTTTCTATTGTTGCCGCCGT
GTCGTATCGGCGCGTACGGGTAGTGACGCTTTCGTGCCGGCGTTTATCCGTTGGTTCCGTGC
TTTTTCTCTGGTTCCGAGTAATAACCGGAAATA
GGAATAGTGTTGTTCGCACTGGTTCAGAAACAGGAGAAGCGGTGCCCCGCAGCGAAGCCG
ATGGGAAAAATATAGGATAAAAAAAGGATCGAGAGTGGGGATCTCCGGAGGGAGAAGGA
AAAAAAAGGAAAGGAAAAAAGGTGATACTGTGAGGAAGCCGTCGGAGCTGGTTCTCGAA
AAAGGAAGCTCAACGATACGCGCGGTGTCGCGATTCGCAGTCGCTTTGCCGTGAAGTTTGC
TACGCGTCCTTGAGGCACGCGAACATCGTGTATGTGTGTTGCGAGTTGCGACGAAAATTGA
CCGGTTCGGTTCGTTGGACGCGTGTAATGGGCAGGGGCTCGAATATTCGGAGTCGAGAGTT
TCGTTCGAACCGTTTAATCTGACTTTGTGCTCCCTTTGTCGAGTTTGACACAGTTCAAGTGTT
TCTCGAGTCGCGTCGACTCTGTCCAGCCATATTTGACGCGAATGCTTTCACGCTGATATCCA
AAGTTATTCGGCTCGTGTTCAAAACGGTGTGTGAGTGTTTTCGAATCGCGATAAATCTTCCG
GGAGACGCGCGTGAAAGAAATTGTAATTTACGCGATAGATGCGAGTTGCGAGTGAAATGC
GCCTCGAATTGGCAACATTCCCAGACGCAGTGTTCCCTAACGATGCCGCCGCGTTTCTAACC
GAACGCGATACGCCGGTCGAACGTTTTAAACGGAATTTACTAATTATTTCAAGCTCGATTTA
CCTTCGAGCTCTCGAGAGCTAAACGGGTACGGAATCGTAGAATATTTCGAAGGGTTTGCTC
TCGTTAAATATCCGAGTGTCGTCGCATCGGGTATCAAGAGAGATCAAGATCCGTATCGATA
TGTGAGGCTCGTAGCGAGAGAGAATCGTTCGATCGATGTTGCCAGCTCCGCTTCGAAGGAC
GAGTGAACATCGAGAATGATCGTTACAGTGAAAAAAACACGTCGCATTTCAAGTGGAGGA
TTTTACACAGAATCGTGAGAGGAGGTGGGTGTATATGTATGTATATATATATATATATATAT
ACACATATATATA
TATACATATATCGGTTTCTCGTGACGCGTCACGCGCGATTTTAATTCTAACCTCGACGGTCG
ATGAATGATTGTGTGCTGTGTCAAGTAAAGTGAAAGTCAAGCGCGACGGACGGCCATATTT
CATGGATAAACGTGCACCATAGTGACCGGCTCGAACTGGTTACAATCCCACGGTTCCAACG
CCACGAGACCCGAGACCTGGAAGGAGGCCCCGAGGACGTCCTGGGAACGCCACACAGTTG
CACACAGCGCCTAGGATACAAAGCCCTATTTCTCAGCATCTCGAGAAAGAGAAGAACTCT
GCGCGCAACAGCCGGCGGCGACGGGTGAGCGAGGGTGGTGGAGGCGGGAGCCGCGAGAA
GGAGGCAGCGGTAGCAGCGGGCGAGGGTGGGAACGTATCGTCGTGCCGGCAGTCGGAGG
ACAGGGAGAATAAGGGCCCCGCGTCGTCGGGCAGGCCCGGCAATCGCGTGCCACGGCCCG
TCTTGGTTGCGGAGGAGGCGAACAACACCGCCTCCAAGCTCCGGAAACGCGGCACCGCGG
TCAAGAGCCCGATCGAGAATCAAAGACCCGTGAGACGGAGGCCGAGGCGACTCTCCTCCG
GTTGCCAGGAGACGAGGACAGAGGCGGCCGCCGCCGTCG

213. >MSTRG.4285.1 Gene=MSTRG.4285

TTCGTGCTTCCTCTCACGAACGTGATAATCAGGCACGCTGATAGGCAGGGACCGTTTCTCGC
TGTAGTCCACCGATGCATAACTCAAGTCCTCGAACGGTTCGAGCTTCTCCGCCTCTTGCGGC
GTAACGGAGATGACAGTCAACGTCAACACGTCGCCCCCCGACTTTATCAAATCCACCACCT
GTTTGTGAGTTGCTCCCTCGACGTTCACACTGTTTACTTCTAATATACGATCGCCTTTTCGTA
CGCCGGCTTTTTCGGCAGCACCTCGAGGTAATACGGCGCTGACATGCTGAAGCGGCGCGTA
CAGTTCCCCGTTGATGCTTCGTAGTTGTCCACCCTCGCTGACCTGACCGCGTACATTGAACC
CAAAACCAGTTTCGGTTTTGTAAATAGTGACGCGTCTCGGTCCTTGTCCGATAGGATTTGTTT
GGTTTACCGGATGTTGAGGCACACGTCCGTTGGTAATCATTGTTACATTCGCGTTTCTCGTTA
CGTCTTCGTTCGCGGCTGGCAAGCGCTGCACCTCCGATTCGCAGTCCGCCATCTTACTCTCT
G
ACCACCCATTGATCTTTCTCTCCTACGGAAATC

214. >MSTRG.4297.6 Gene=MSTRG.4297
ATCAAACTCAGTCGCAAAGCGTACGTTCGCGCGTGTGTTCGTTTGCGGGTTTCCCTTGTTCA
AAGACAAAACTTGCTTGCTTGCTTTGCTTGCTCGTCGCGTGACTTTCGTCGTACTTCAACGCG
CTCCTCGCCGGATTCGATTTGTGCTTTCGTTTCCAGTTGGAACGGTTCGTTTATCCATGTGAG
TGGTGAAACGTCGAACAATCTCCCTATAGATAGTGACTTGTTGAACTTATTATTACGAAATG
AGGAGCACGTGGATTTCCGGTGACATAGCCGAGGATCACGATGAGTGTACTGCACGAGAG
AAACCTTGAACTTGAATCGCTGAAGATCGTCGAGGCCTCGCTATCTTAACGACTTGTTCTCA
TCGACGTGTCAAAATCGTGAACAGCCCCGGAAGTGACAGGACAGACAGAGGGTATAGCCG
CGACCACCGATGGCGGCCGCGAGCTTTCCGCCAAACTTCTCGAACGTCGGCATATTGGAGA
ACTGCGCCGGCATGGAGGCGGCGAACGGTGCGATCGAGCACGACTTTCACAATGCTTTGGT
GCCTATAAACGGTAGCCATTCAGGCAGCTCCGGGAGGAGTACACCGAATGGCGGCGACCC
AGCACCCGGCAAACTCTTCGTGGGTGGCTTGTCCTGGCAGACCAGCAGCGAGAAACTCAG
GGAGTACTTCGGCATGTTCGGTACCGTCACCGATGTCCTGATCATGAAGGATCCGGTTACA
CAGATATCGACGAAACCGTGAAGGTAACCGAGACACGGCACGATACAGCAAGAGTACGC
AGGGCGGCGAACGCGAAGAACGCCAAGAGAACGACGGATTAATTAGTTTAGTTTTTTTCGA
TGCCTAGATGAGTTGCCAATAAAGTAGTTCGTTTCAAACTTGTAGCTAACCTGTATTGTGTG
TACGATAATAACCCTTTTCCTCCTTCGTTGTTTCCTCTCCCTCCCCCCTTTTTTCTATTTATTTT
TTTTTTTTTTTCCTCTCTCTCCACAATAGAGAAATACCTTTTTTTCGAATTTCGTAATTCTGTAT
TATTATTAATCGCGGCTAATTATACATCGTTCTATCTCATTTCATGCCATGAAAGATGGGGA
GACACGTGCGAGAAATAAAAAAAATTCTCTGGAGAAAAATCGATCGATTTTTTTACTCGCA
CATGTATTCGCATCCCGAGTAACATCCTTGTTATTATTATTTTTTTCTTTCTTTTGTTTTGAGA
GCTGTATAATTTTTTTTTTTTTTTTTTTAACTTACTCACTCACGT

215. >MSTRG.4299.1 Gene=MSTRG.4299
CACTCTCGCCGGTCAAAGCAACTCTCTCTCCCTCCCTCTCCCTTCCTCCTTCCCTCTCCCTCTC
TCTCTGTGTCACTCTTTATTCCTTTCTCTCTGAGAAGGTGGAGGAAGAAGGAAGGAGATGAG
AAAAAGGATTCTGGATTCTGGATTTTGAATTTGGGAGAGAGAGAGATATATATATATATAT
ATTCACGTCGCCGGGGATATTGTCTTGTCACAGCAGTACGCCTTTAAAGAGTTACTGGTGTG
ATGGGACATTGTCCTCGTCCCGTGGCCCTTCGTCGAGATATACGTCGCTTCAGTGTTCCTTTC
CTTTTCTTCTTCTTTGGATAGAAAGAGAAAAAAGTGGAAGAAGAGAAAGTAGAGGGGGGA
GTGTTTCTGCGTGTACACGTTTGTGCTTCGTGACACGTATACGTACGAGTATAATAATAACG

TTACGTGTACAACATTTCATCGGTTGGTAACTGATAAATGGACGAAGAGGATCAAAGAAGA
AAGAAGAAAAAGGATTTTGTGTATTCGAGGAGGCGTTGCCTTTCGCGAGACAATTCCTACA
CGTCGAAAGATGGAAGAGGACTGGTCTCTAAGGTGTCTCTTAATTCTTGCCAGCGTGGATG
CCGTCACCGGTTTCCGGTCGATAAGGCAGCTTCCGCAAGGTGCGGCTGTTCGAACGAACGA
CCATTTGATTCGCGAAAGAAAGAAAGAAAGAACGTGTATCGCGCTTAGGATCGATGTTACG
TAAAAAAAAAAAGAAAAGGGGAGGGGATAGAAAAAAAAAAAAGAAGCATTTTCATCCAT
CCAACATATCCGACACACTCTTCTCTCGATCCCCTCCTCTCTTCCACAAGAAACAAGGAGA
AGATATCGTGGATCCGTTCCACGAGAGATTAAGTTACCGACGAACGATCGCCAATCAAAC
ACAGTGAAATTTTGAACGGTGTCGTGATGTCTGTACTGGCAAGATGGCGATGCCCGTAACG
TTCCAGCTCTCTATATGGAAGCTACGCAAAATAAAACCCGCCATACAATTGGACAATCGTT
ATCGGTTGATGAGGAAAGAGCCAATGCTGGACGCTGATGGAAAGGAGACCGATGACATAG
GCCCTGACGACACCGCCCCCCCCACTCTTGCGGAGGGATACGCTATAATGGAGGAAAAGG
GATGGGATTACTTTTCACCGGAGAAGTTCCTCAATATTTAAATATATTTTTGTTATCAATTTA
CATAAAATCGACAAAATTATTGAATTATTTTTGGACTGGATATTACAATAAACGATTTTCCT
TTTAAAATTCATCTGTTGGTTGGTATATTGGTATAAGAGAGAAACGAGGAGATCAAAAAAA
AAAAAAAATGTAGGCCAGCTTTTTGAATGGAAAAAAAAGGCGTATTAATCTACGCATTGTG
AAAGACGCGCCATAGTTACTGTTACGAGCCAGGTGTAATAGTTTGCAGAAAGCGAACAAC
AACCCTCCTTTGATGTCCGATAGTGTCTACCTACCGTAAGCTTGCCAGCTGCTGCACGTTCC
ACCACGATATATATATAATAAAAG

216. >MSTRG.4299.2 Gene=MSTRG.4299
GGTGCGGAGGCGCCGTCGCGAGCGAGCGACGCAGTGCCGTCGGGACGCTCGACCGAGCGA
TATCACCCTCAGTCGCAGCGAGAGACGCCTACGAGACGACGACCGTTGCCGCCGCAGCCG
CCGACGCCTGAACAACGTTTTCCCGTAAAGACAGACTCTTCTCCTACTACTCCTGCTCGGTG
GTCGGGTGCCGGGAGCCATCGTCAATGCATCGCTTGCCGCGCGCGAACTCTTCGCGGCGGA
GAAGAGGGGGGAGGAGGAGGAGAGAGAGAGAGAGAGAGGGAGGAGGGGGCGAGCGGC
GGGCAATTTCTTCGCCGCGTCGAGGCAACTTTTTTCCCATCCTCTCTGTGTCACTCTTTATTCC
TTTCTCTCTGAGAAGGTGGAGGAAGAAGGAAGGAGATGAGAAAAAGGATTCTGGATTCTG
GATTTTGAATTTGGGAGAGAGAGAGATATATATATATATATATTCACGTCGCCGGGGATATT
GTCTTGTCACAGCAGTACGCCTTTAAAGAGTTACTGGTGTGATGGGACATTGTCCTCGTCCC
GTGGCCCTTCGTCGAGATATACGTCGCTTCAGTGTTCCTTTCCTTTTCTTCTTCTTTGGATAGA
AAGAGAAAAAAGTGGAAGAAGAGAAAGTAGAGGGGGGAGTGTTTCTGCGTGTACACGTTT
GTGCTTCGTGACACGTATACGTACGAGTATAATAATAACGTTACGTGTACAACATTTCATCG
GTTGGTAACTGATAAATGGACGAAGAGGATCAAAGAAGAAAGAAGAAAAAGGATTTTGTG
TATTCGAGGAGGCGTTGCCTTTCGCGAGACAATTCCTACACGTCGAAAGATGGAAGAGGAC
TGGTCTCTAAGGTGTCTCTTAATTCTTGCCAGCGTGGATGCCGTCACCGGTTTCCGGTCGATA
AGGCAGCTTCCGCAAGGTGCGGCTGTTCGAACGAACGACCATTTGATTCGCGAAAGAAAG
AAAGAAAGAACGTGTATCGCGCTTAGGATCGATGTTACGTAAAAAAAAAAAGAAAAGGG
GAGGGGATAGAAAAAAAAAAAAGAAGCATTTTCATCCATCCAACATATCCGACACACTCT
TCTCTCGATCCCCTCCTCTCTTCCACAAGAAACAAGGAGAAGATATCGTGGATCCGTTCCAC
GAGAGATTAAGTTACCGACGAACGATCGCCAATCAAACACAGTGAAATTTTGAACGGTGT
CGTGATGTCTGTACTGGCAAGATGGCGATGCCCGTAACGTTCCAGCTCTCTGTAAGCTTTAG
GTGCTCTCTCTCTGTGTGCGTGTTTTTCACCCCCGTGTCAACGTTCCTCGTGTCGATCGTAAT

ATCCTTTTCCCTCTTCTCTCCCCCCTCCCCCTTATACGTCGATTTCCATTTACAGAATATTTCA
AAATTTCCTATATAATTTTCCAAGATCGAATTTGCAGCGCGTGAAAAAGAAAAGAAGTTTT
CTCCTCGATTTCTCTTCTCGTCCTCGTGCATCCTTCGAAGAGAACGAGACTGGTGGCCAATA
ATAATTTTTTCAGTTTTCAACTGCTTCGCCGCGACGAGATATCGCCTTGCTTGGCCTAGTTCC
CATAGGAGGGGAGGGGGAAGGCGAGGGGAGGGGAATGGACGGAGAGGGGAGGGGGGGG
GAAGGCGATGATCGGTTCCCCGTGATATCATCCTCTCCGCGTGTCTCCGCAATACACCCGA
AATATCTGCAATCGCGGATATTTACATTATTGAATAATGAATCGGGCTCGTTTGTGTGCGTT
GTTGTGTGTATACGCAGTGTGTGTTGTAACATGGATTAAAGAGGCGCCACGACGGGGCGCA
GTGAAGAACTCGAACGGGGTCGATGAAAAGGTTAACGTCGGGTTTAGGTCGATCGTACCCC
GTACACCGCCGTCCCTCTCTTTCCCTTTCTCCTACCCCGCCCGCCTCCCCTCTCTAATCGACT
CCCCCTCTAGGTTGCCTGGTTTCCCTAGTTTCGATCAGAGTTTCTACGGAGCGGCCGACGTT
CGATCGGTGTTCGAATGACGTACGTACACGCGCGCGCGCGCGCGCATATACACGTATAC
ACACACGTACACGTTTAGCGGCGCTCTACCTTTACCGGCTCATCGCGACGGATCATTTCGAC
CTGTTCAATCAAACCGATTCATGTAAACAAGTAGCAATGGCACAAGAATTCTGATGAACCA
ATCAGAGAGAAGAGTTATATTCGAAATTTATTAAGGATTTGGAAGAAAAGGGCGGGAATA
TTATCGGATGCAACGACCAAGTCACGGATCTCATCAGAAAGGATGATGAATTTTCGATTTG
CAATTACCTCCATTAAAATTCGCCGTTATTCCACCTATTTCGAAAAAATGAAAAAAAAAGG
AAGAAAAAGGAGAAGAAAAGAGAAGAGGGGGTCGAGTCTTCTTCTAGTTACGGCATGC
AGAGATCACTGTGAGAATTGATTAGCCGTTGAATTAACGGGCGCGGCACGTTTGCGATTCG
AGAGAGAGAGAGAAAGAGAATGAGAGAGAATGAGCGAGAGTGAGAGAGAACGAAAGAG
GGAGAGAGACTTGGTGGTCGTCGGAGTCGGATTTCAACGGCAGTGGCGGCGGCGGTG
GTGGTGGTAGTGTGGTGATGGTGGTAGTGTGGTGATGGTGGTAGTGACAGTATTGGTGGCCT
CGTAGATGCACGTGGAGATGGGAGAGCAATGGTGGTTACTAGTGCTGGTGGTGAAAAGGT
GATGGTGTGGTAGTATGCCGGGTGAATGGGGTGGGTTGTCATTTAACCTTCCTCGTCGGTTA
GTAGAGGGCACAGCCGCCAGGCGGCGACACGTTTGATCGCGAGGAAAACGAGAGAGGAG
GAGGAGGTGGTGGCAGGTATATACCCCTTGGTTCGTTTTCACCGGAGTTTTCACCCGGAGGA
CGGACAACGACAATGCTTGGTCACCTGGAGGTCACCGCAATTGTGTACATTTGGCTATTTAT
CGAGAAGAGAAAATTCGTTTGGGAGAAACGCGATCGATCGAAGAATCCGTGAAAAAGAA
AAAAAAAGAGAAGCGAGAGGCCAGCCCGAGAAGCGAGAAGATAATGGAAAAACAGAAT
ACTGGCTCCGCCATGTTCATCGCCCTCGTGAGAGACACCTTAGACAAGAGAAATCCCTCAT
AGATGTGTAGAGCCTAGAATATGGAAGCTACGCAAAATAAAACCCGCCATACAATTGGAC
AATCGTTATCGGTTGATGAGGAAAGAGCCAATGCTGGACGCTGATGGAAAGGAGACCGAT
GACATAGGCCCTGACGACACCGCCCCCCCCACTCTTGCGGAGGGATACGCTATAATGGAG
GAAAAGGGATGGGATTACTTTTCACCGGAGAAGTTCCTCAATATTTAAATATATTTTTGTTA
TCAATTTACATAAAATCGACAAAATTATTGAATTATTTTTGGACTGGATATTACAATAAACG
ATTTTCCTTTTAAAATTCATCTGTTGGTTGGTATATTGGTATAAGAGAG

217. >MSTRG.4340.1 Gene=MSTRG.4340

ATATCTAATCTATGGTTGAATTTGCTCTTAATTAATTCACAACGGTTAACCGCCATATTGATT
CTCTTCATTATCCAATCAAAAAAATGTTTCTCGTTTTTTTTTTATAATGGCATTATTTTATATG
ACGCACATTGTAAATAGCTTATAACATTGGTACTTAATTGATTTACTATGATGTTTTGGCGA
AATAAAGATTTTACAGCATATAAAGTGATTAAACAGCATATAACAAATTAGCAAGAAAAT
AATGTTTATGAATTGCTAATAAATACGCTTAAGATATTGATTCATTATTTTTTTATTTTTTATC

CTAAATAATGTAATCTCTCTCCTTTTATTTATTTTTTTTATTTATGTTTGCTTTTAACTTAGTTT
CACTTTCAATTATTACTAGTATTATAATTAATTATTTTAGATTCAATTAATAATATTGAGAAT
CATAATAAAATAGTGAAAACGTCAAGAACAAAATCTCAAAGAAAGAAATAAAAAATATA
TTTGATATTAAGTCTTCTCTATCTATTCATACTAAAATTAAATCAAAACATATATCGTTAAAT
AATCTTTTAAAAAGAAAACTTTAAAAAAATAAAAAAATTGCACATTGAAAAGTTGAATTTG
AAAAAAAATAAAAAGAAATATCCAGCAACATAAGGCGCTACTATCGTTCGTTACCAATCT
ATTTCTTTTCATCTGAAACTTCCGCTTCCGAAAACTTAAGACTAAGTCACTGAATTATACGG
AAGAATGGCAAAGTCTAAGAATCATACTAATCATAATCAAAATCGTAAAGCTCACCGTAA
TGGTATCAAAAAACCAAAACGTTATAGGCATGAATCTACACTTGGTATGGATCTCAAATTC
TTAAGAAATCAACGTTTTGCCAAAAAACATAATCTAAAACCCAAAGAACAATTAAAAAGG
GCAGAGAAACGTAAAGCTCTTCGTGAAGCTAAAAAATAATTCTTTTTTAATATCAATGATT
TTATTGTAATAAAAATTTAAAAAATGAAAAAAAAAAAAAAAAAAAAAAAAAA

218. >MSTRG.4384.2 Gene=MSTRG.4384
CAAAATTATATTTAATAATTTAATAATTCTTAAAAATTAACTTAACTAAAAATTTTATTTAA
ATACTTTAGAAAATTATAAATATACTTAAATTTATAATAATACAACTTAACCTAATCTAAAT
TAATATATCATAATCTAATACATTTATTATGAGATTTTCTAGTTGAGGAATTACTTACTTTTA
TATTTCTTGGTGGTGGGATCCTAGTTCTCTTAGCAAAGACTGCTGCCGATTCTAACCTTATAC
TTGCTACATTTCTATTTCTTCTCAATTCTTGTTGTATTCTTCCTAACGATTCAATCCTTGATGGT
GAATCTCGTCCCAAAATTTGTTCTCTCATTTTTCTTCTGATTTGTCGTTTATGGAGCCTTGCAG
TTTGTTTCAAAATTTTATCCAATTTGCAGATTTGCAGCCATTTTCGCGATACTCGAGCTGCAC
AGAGCAAAGACTCCTCATCCAAATTGCGAAGAATTATCTGTGATACTTCCAGTGGCAATTC
GGAAATGAAATCCACTTTTGTAGGCTCAAATATGCCAATAGTTTGTAGGAAGCTCTTGAAA
AATTCCATATTGCTCTTATGGAAGGAAATTCAGGATTGATAATTTTTCCAAAAATTGGATGT
TTATATACTCTTTTTGCCGAGACGAAAGGGGCGGGGCCTCGTTTGATTGGGCCTTGGTCCAT
TGATTCAACCAATCACGTTCGACCACGTAGTATCAGTTGTATCGTAACCAATTGTACCTAAC
CAATAGGAAGTAATATATTCTTGAATAATATATGATAATAAGTAGCAGTATTTATATTTTGT
TATATTGGAGTATATTGTAGTTTCATAATGGATAATTAACTTGAATTATTTTTATGTTAAAAA
AAAATTATTTCAAGACAATAATCAATATTACCAATTTAATTATATTATAACAAAGGAACGT
AATATGATGAATGATTAGAATAATTAACAATTCGGAAGAATAACATTAAATTTTTTTCTTTA
TTTCAATTTTCAATTGTTTTGAGAAATTAAA

219. >MSTRG.4384.3 Gene=MSTRG.4384
CAAAATTATATTTAATAATTTAATAATTCTTAAAAATTAACTTAACTAAAAATTTTATTTAA
ATACTTTAGAAAATTATAAATATACTTAAATTTATAATAATACAACTTAACCTAATCTAAAT
TAATATATCATAATCTAATACATTTATTATGAGATTTTCTAGTTGAGGAATTACTTACTTTTA
TATTTCTTGGTGGTGGGATCCTAGTTCTCTTAGCAAAGACTGCTGCCGATTCTAACCTTATAC
TTGCTACATTTCTATTTCTTCTCAATTCTTGTTGTATTCTTCCTAACGATTCAATCCTTGATGGT
GAATCTCGTCCCAAAATTTGTTCTCTCATTTTTCTTCTGATTTGTCGTTTATGGAGCCTTGCAG
TTTGTTTCAAAATTTTATCCAATTTGCAGATTTGCAGCCATTTTCGCGATACTCGAGCTGCAC
AGAGCAAAGACTCCTCATCCAAATTGCGAAGAATTATCTGTGATACTTCCAGTGGCAATTC
GGAAATGAAATCCACTTTTGTAGGCTCAAATATGCCAATAGTTTGTAGGAAGCTCTTGAAA
AATTCCATATTGCTCTTATGGAAGGAAATTCAGGATTGATAATTTTTCCAAAAATTGGATGT

TTATATACTCTTTTTGCCGAGACGAAAGGGGCGGGGCCTCGTTTGATTGGGCCTTGGTCCAT
TGATTCAACCAATCACGTTCGACCACGTAGTATCAGTTGTATCGTAACCAATTGTACCTAAC
CAATAGGAAGTAATATATTCTTGAATAATATATGATAATAAGTAGCAGTATTTATATTTTGT
TATATTGGAGTATATTGTAGTTTCATAATGGATAATTAACTTGAATTATTTTTATGTTAAAAA
AAAATTATTTCAAGACAATAATCAATATTACCAATTTAATTATATTATAACAAAGGAACGT
AATATGATGAATGATTAGAATAATTAACAATTCGGAAGAATAACATTAAATTTTTTTCTTTA
TTTCAATTTTCAATTGTTTTGAGAAATTAAAAAATGAAACAAGAAGTTGGCCATAATGTATT
TCGAGATTGAAAAATAGAATGAAATGATGAATTGCCTTGTAACATTTGTGATGTGAAAGTT
TTTTATTACACATTTTAGATTTGATGATGTGACTATGATTAAGGTAAATTCTCAGATTCCTAA
TTAAATTAAAAACTTTGTAATTATGCATTGCATAAAGTCAATAAATACTAATTTGTAATATT
CATATCATATATTATTGGACATACAATTATTTATTTTTCTTAGCACTAAGATGTCAAAATTGT
ATTTAATGACAAATTCTTAAAAATTAACTTAACTAAAAATTTTATTTAAATACTTTAGAAAA
TTATAAATATACTTAAATTTATAATAATACAACTTAACCTAATCTAAATTAATATATCATAA
TCTAATACATTTATTATGAGATTTTCTAGTTGAGGAATTACTTACTTTTATATTTCTTGGTGGT
GGGATCCTAGTTCTCTTAGCAAAGACTGCTGCCGATTCTAACCTTATACTTGCTACATTTCTA
TTTCTTCTCAATTCTTGTTGTATTCTTCCTAACGATTCAATCCTTGATGGTGAATCTCGTCCCA
AAATTTGTTCTCTCATTTTTCTTCTGATTTGTCGTTTATGGAGCCTTGCAGTTTGTTTCAAAAT
TTTATCCAATTTGCAGATTTGCAGCCATTTTCGCGATACTCGAGCTGCACAGAGCAAAGACT
CCTCATCCAAATTGCGAAGAATTATCTGTGATACTTCCAGTGGCAATTCGGAAATGAAATC
CACTTTTGTAGGCTCAAATATGCCAATAGTTTGTAGGAAGCTCTTGAAAAATTCCATATTGC
TCTTATGGAAGGAAATTCAGGATTGATAATTTTTCCAAAAATTGGATGTTTATATACTCTTTT
TGCCGAGACGAAAGGGGCGGGGCCTCGTTTGATTGGGCCTTGGTCCATTGATTCAACCAAT
CACGTTCGACCACGTAGTATCAGTTGTATCGTAACCAATTGTACCTAACCAATAGGAAGTA
ATATATTCTTGAATAATATATGATAATAAGTAGCAGTATTTATATTTTGTTATATTGGAGTAT
ATTGTAGTTTCATAATGGATAATTAACTTGAATTATTTTTATGTTAAAAAAAAATTATTTCAA
GACAATAATCAATATTACCAATTTAATTATATTATAACAAAGGAACGTAATATGATGAATG
ATTAGAATAATTAACAATTCGGAAGAATAAC

220. >MSTRG.4453.3 Gene=MSTRG.4453
AAACGTCATTTCCTACTTCTATGTAACGAGATAGAAAGTCATTTCCAATTTATGAGTTGGAA
GTAATAATACTTATAGGTGTTTTATATAAATTAATTTTATTCTATATTTAAAAATTAAATGAA
ATGTAAAAAATATTTATATATTCTAAATTTGATTATATCTGAAATAATTTAACTTAAAGAAA
CTTCAGCATGTATACAAGAAGCAGACAGAGTCAAGGCTATGCCAATATGAGAAACCAAAA
TAGTTCAAATGTGGGATATCAAGGTCAAAGCACTGAAATAGTAGCTATACAACAATACCG
AGGAGGTCTATTCAGCCAGGGACTAGTTCGTCAGTTACCCACATAGCATAGCCTCCATAGG
CCATATTTTTAATAACATACTGAACTGATGTTTAAATGATGTACAAATGATTATGATTTTTAA
TAAATTTTATAATAAACTTATCAACATTAATTTGTATAACACAT

221. >MSTRG.4558.1 Gene=MSTRG.4558
GGGGTAGCGACGCGTGTATCGACGTCAGCGTCGTCGGATGCGGCAGAGACATGGGCGCGT
GCATCGGGGCGCTCGTCGGCGGCTCCAGAGCTTTGTCTGCAGATAGTCAAAGATAGGAACG
TACGGGGACCTGAGGATGTAACGCCGGATGCACCAGCGTCGCGTGTTGCAGCGTTCCTGGT
AATTCGCCAGCGGTCGAGTACGCCAATGGATGGTGCCACAATTCAGGACCACCAGGGAAG

AATGGGCTCCCTAGGTGTCCAGTTAGAGGGTGCATCAGAGCAGGGTGCGAGGTGGCTGCTG
CAGCGGCTGGTTGCTTGGGTTTGCTAACCTTTGTGTTACTTTTTGCAAATTCCAAACGTATGG
TTTGTGGCATATCAGGATCGAATCTAACCCCCTGCTGAAGGTCCTGTTTCGCCGCCTCTGCA
CCGGCCCTCGTGTGAAAAGTCACGAAACCCACCGGCGACGCTGTTTTCCCATTTTTGCTTGT
GACTTTCAACAGGGATCCCTCGTACCCCTCGTATGCTCTGAAGAGCAGATAGAGCTCCCTC
GGCTTGGCGTCCATCGGCAAGCCGCTCACGAACAAGGTGCGCACCTCGTCTTCACCGGTGG
CCACCGTGTTGACGGAGTCCATACTCTGTGAGAGCGGTGCGTCCATGATGCAAGCGAGTTT
GCTGGATGGTCCGGGGTTTCCGGGGGCTGCGGTTCGAGCGGGTGGCGGCGATGAGTCCACC
GTATCCCGTCTCGGGCGTTTTAAAGCGGGCGGCGTTATGGTTGTACCGGCGAAGACGACAG
GCGCCTCCAGGACGTATCACTGGAAATAACCCTTTTCTCGCGAGAAACCCAGGCGTTTCGC
CTCCTTCCTCCTTCTCCACCTTTACTCCTTTCCTTTCCGTATCTAAACGCGCGCGCGCACGTAT
GTATGTGTGTCGGTTCTACTTTCTTCCGTTCTACCCTCTCTCTCTCTCTTCTTTTTCGTCGTTTC
GCTTCTTCCGCTTCCTATTATGTTTCTTCTTCGTCGCTTGCACCTCCACCTCCACCGTCAC

222. >MSTRG.4605.2 Gene=MSTRG.4605
AAATTTGTAAAAAACTGATTATTAGATTACAAATTTAATATTTTCGTTTAGTGCGAGGGCAC
CAGTTGTACCAAGAAAATCTCGCTAAAAACGTGTGTTATCGATGTCTATTGATAAGGTTGTT
TATGCCAAAAAATGTAATAAAATATTGTGAAAACAAAGTTATAAACAGGTTAAGGAAATT
AAAATTTTTATACTGTGTGTTTAGTGTATCAAACTGGAGATGGAAAATAGTGGTGAAAGTG
GTGACGATGGAGGACCTTTAGGTCCGACTGCGTTTCTTGGAGGAGGTGGTGTTTCTGCATCA
TATATCAGTGTTCAGTCTGACGATATGGAAGATGATCCTGAAAATACAGATGATTCAAATC
ATGGAGCAAGTGATCCTTTGCAAGGAGCTGGAAGTGGCAGTGTTGGAGGGCCTCTTCGTGA
ACAAGATCGATTCCTTCCAATAGCAAATGTAGCAAAAATTATGAAAAGAGCAATACCAGA
AGCAGGAAAAATAGCAAAAGATGCACGTGAATGTGTTCAAGAATGTGTATCCGAATTTAT
ATCTTTTATCACATCAGAAGCAAGTGATCGATGTCATATGGAAAAACGAAAAACTATTAAT
GGTGAAGATATTCTATTTGCTATGACAACTCTTGGTTTTGACAATTATGTAGAACCACTAAA
AGTATATCTACAAAAATATAGAGAAGCAACAAAAGGAGACAATCCAGGTAATGTTCCAAC
AACAGGCAATGGAAAAACTGAACCACAAGGAACTATATATGAAGATCAATTATTTGCTATT
GCTGCAACTGCATCTAGTGCTACCACTTCTGATACACCTGTTATATATAGTTACACTTCCACT
G

223. >MSTRG.4649.7 Gene=MSTRG.4649
GTTTAGTGACCGAAAGTACTAGTGCTGCTGGAAGTCAAAAACTGTGTTGAGAAAAGAAAT
AGAACCCGCGCAATTGAGATTACTATAATTATATGACTTACATCTTTATTTTCTTGTGCCTCG
ACAGATTTAAATGTTTTATTAATCCAATCTTTGACATCGAAAGTATCTTCGGAAAATGCAGA
TACATCCTGAAATCAACAATTTAAGAGGAAATACTATAACGGAAAAGTCATTCTGGAAGAT
GCTGTTCATCCCTTACAATGATCAGTTTGGTTTGATTGTAACACAGGCTACTTGGATTTCGAC
AAACCATTTATCAGAAGATCATAAATTGAAGAGGATAAGGGAGGGAAAATAGAAAACAG
CATGTTATCGGCCATTTCCGGGCCCTTTGTTAATCGCCACGCCTCCGAAACTGCGTTTGCGC
GTAACAATTTGCGCAACAGTTGGAAATTGGAATTTTGCCTCGAAATAACCTTAATTTCGACA
AAAATAAGAATTGGCTAAA

224. >MSTRG.4672.1 Gene=MSTRG.4672

CTAGTGTGTACGAAATATTCGTAACTTTGACTTTTAAACGGTTGTTATAGAAACAAGCTCTT
TGAAATAGTGATATTAAGTCATTTGAAGAAATGAGAATTTTAATAAAAAATACAAATTAAA
TATTATTTTGCTGTCTACATTCATTATTTTATTTTTAAATTGTAACTTAATAGTAATAGTCTCA
CCGTCAAACTGTCTCACAGCGGAGAAAAAGAAAGAAATCGAGGCAGCATGCCAGAACCT
GTACATCATCAAATAAATGCTGCTCGTAAAACATTCCAAACACTTTATCAAATTTCAAAGCT
ATTAAATACAAATTTGGATCCAACTACATTAAGTATATGCATTCGATTATGTGAGAATGGTG
TAAATCCATATGCACTTGCAAATGTAGTAAAAGAATTACAAAGAGAAGTGAGAGCTATGG
ATAGCATTAATCAACTAGATTCTTCTACTAGCAAATCTAGTTTAAATAAATAATTAATCATT
TATTATTCATATATATATATATATATAGAGACTAGTTTGCCATATATATATATATATATATATTG
ATTATATAATAATTTTACATTATTTAATGAGTGAAATGAAATCTAATAATCTATCATTTTTTA
TATATATGCTAATAATATGTGATTAACAATAAAATA

225. >MSTRG.4696.1 Gene=MSTRG.4696
TATATATGTATATATATATATATATGTATATATATATATACATATATTTTCATGTATATAATACG
AAAATTGTTTGTATATATTTCATTAATTCATTGTCATTTTCTTCGTGATATGGCATATTGTGTC
TTTGATACTTGTCGCTATGGCGAAGGTTATTCCTTTCGAAATAAAGAAAGGAAATGGCGGT
ACATTTAGGACGGATATTAAGACATGAAAACCGGAAGTATAGCATTTCGGAGGTGAAATG
CATCGACAACCGAATTTCTAAGAGCTTGCGAATGTCTAATGATTTCACAATTTAGAGATTTA
TAAATAAATGTACTTTGTTACTTGTTTTTTGTGCGTTATAATGGTAGATAGGTACAATCCGCG
GCATAATCCGTTAATTGATGTTTTTAATTAATAATATCGAGAGCAATAAACATTATTTCTCTC
TTAATTGAATACCTAGTCAGAGAATAATGATATTTGATTAGTATATAAATTTTAATTTTACA
ATTCTTTTTCTTCTTTTTTTAATGCAATATCTTTTTCCTTTACTCTGAAAAAACTGTACTCTTTA
AATCGTAAAAAAAAAAATCATTCAAACATATTATTAGAAAATGTATTATACTCATACTCATA
TGTGTATTAACCCGTCTATTTTAAAGAATCGTTAAATGTTGATTTGTTTTTAAATAGTGGAGT
TTTTTTTCTTTTTTTTTTTTTTTTTTTCGTTAGCGAACTATAATTAACGGTCTATGTCCGCTAAT
AGAAAAAAAAAATGTTGAATGATTAATCGGCTACTGTACTCAAAATAAATATGATATTCTG
TAGAGAAAATATGCTCGAAAATTAATATATTGATCATTATGAGATGATAGAAAAGATACAT
ACATATTGTATGTGAAATAAGCATCTAATAATGTTGACAAGAAAGAAAAGTATATACACAC
GAACTTGTATATGTATACATATATACATATGTTATATACATTTTCCACAAGTATTTTGCACAT
TTGATATGAAAAGAAAAAGAAAGACATAGAAGGAAGATACTTTGCAACCCTTAATGCCTA
AGTACACAACGAGAGCTTGCACTTTTGCGAGCATATATCTTCAAATAATAATACATGAGAA
TTAATAAATTTGTTTGAACAGCATTTAAATACAATAATTATTGCACATACAATTTTTCTATAA
CAATGGATCACAATTAAATTAAATGTGAAGTATAGAGAACGAATATCTATTAATATATCTT
ATAAAATGCTTCATTAGTGAAATTCATAATTAATTGGCAATATAGGGTTGCAATTGTAAAGT
ACAGAAAATCTTCTTCCTAAATAAAACTGCTTTCAAGATGGTCTCAGCCTATGCTTTACTCA
ATCTCAGGATGTATTCTGATATGAACTGTTCTCAAGAGTCATATTTGGTACATTACTGTAATT
AATTAACGTTATTAATGGAATACACAAATGATATATAAATAAATAGTGCCTGGCACCACAT
GTTGCCCTCCTGCAGGTTGACTTGTTCACGTTGTCTGTAAAAGATAACT
TGTCGACGAATTATGTGTAAAAGAAAAGCATATATCTTAGGGAAAGATATGATAAAAGTA
GACTAATAAGTAATAAAATAAAATATATTGAATAAAATGAGAAAGAAAAAATGAATAAG
AAAGAATAGAAAGATTATAATATCCATGTTATAAATTAGTAAAATTATAGATTTTGTTTTAC
AACATTCTGTCGACGAATTAACAACTTGGCAAGTCAATTTTGCTTCTATTATAACCGAAGAT
TATATTATACGTAAGTTAAAGAAGGAAAAGAAAGAAGAATCGTATGAATCTTCGATATTAA

TTTGTTTCTTTACTAATGTTGCAATTAGAAATATATTTGATGACTGTACGAACAATCGGTGTG
TGAAACATAAGATATATAATGTATAGTCACTAATGATTAATCACAAATTTTTAAAATGATC
ACAAAGTGCAATCTCTTAGCCAACACACTTTTTTATCAAAGAAAAAAAAGTATAAAGAAAT
TGTTATTTTTCATAACGAAAAAAGTAAGATTTAATGTATTAAAAAAAAAGTATATATATATA
TATATGTCTCCACTAGTTATTAAACATTTTCCTATTGATGCAAAATTTATTGTATTTGAACTG
ATATTATATATATATATATATATATGTATATATATGTATATATATATGTATATATATATATAT
ATATGTATATTATATATATATATCTTTCTTAAGTGTACATGCCTGAAGTAAATCCTTAAAATA
TAACCTGCGATACGATTGCATCGGTTCCAATTCTTAAATCATTGCAGGTGGATATCTCAAAA
TCTCTGAATAATATAATTACTTCTTAATTATATTATCAATTACTCGATATTATACGATTATTTC
TCGGCGATATTTCAAAAACCCAGTGTTATTATGTGCTTCATTTGTTTGTATAAGACAAAGTA
ATTTGCACAAACTTTTCATCTATAATTAGTAAGAATAATGATATGTAAAGTCTCATAATGAT
TCGCTAAATGAGTTTGGCACAAGATATAATTATTAATCATAAATGAGACAATTGATAAACT
TTTTATATTTTAAAAAATGATTTTATAAAATTTTTTTAACTTGAAATAAAATGAAAGATTTT
GTTTACTTTATATTTTGATGTTACAAATAAAATATAAGATATTGAAGAAGTTTATGGTTTTTT
AAAAATATATTATATCTTAAATTTATGAAAATATATAATGTTATATATATATATATGTTAAT
AAATTCTTTTTTCAATTCTTTACCCTTAGTGCACAAAATCTAAAAATCAATTTTCGATTATAA
TGTATATAGATTTTATACAAAATTATGAAGCATATAATATTCTTTTATAATATATATATGTAG
CTAATCTATCGATTATTAGACATATTCTTGACATTAGACTGTCTGAGAAATCGTTTTACCTGA
TCCACCGGTACTCGTGGTCTCGTAATAAGACAAGAATTACGATTTGCTTACCGATCTATTTT
ATTGTAAAATACCCAAGTGAAAAATAAAAAATACGTAGTGATACCTCTTAAGGTAGGGAA
CTTGTTAAATGGTGTACGTTAACGACACAATTGTGCGTATCATTTTAATGTATATTTTTAATA
TGACGATAAAAAAAAAAGTATGCATAAGTTACACTCACATACACGCAAATACGTATTTGAT
GTGTATGCTCTTGATTGGGGCACAACCGAGATCAATATGGAACCAATAACATTTTAAATGA
TAATATATCTGTTTTATTCTCTTGTTAAAGCATTTTGTCAATTTTAACCTGTTAATCAAAGATT
ATTCTCTTATTTATTGTTATATATTATATCTAATGTTAAATATTTAATAATCCATTATAGTAAT
AATGGATAAATATGAAGTGATACTGCTAGTAACAGTTTATTCCTAAAGTTTGAATATCTTCT
TTAGGTTAAATTAGTTAAATTGATTTAAGAAAAAAATAATAGTTTCGTGTCCTTTAATATTG
ATCGAACGATTGTGCCTTAGCAAGAGTCGAACGAAAAATATCGTAGTGTCGGCGATTATAT
TATAGTTGAAAACTCGAAGACTATAATTGAAATTGTTGGAATGCTGTGAAACGAAATGGAC
CATCATTTAGAATGCATTAAATTATAGGACATACAGGAATTTAAAAAAAGAAAAAAGAAA
AAAAGAATATAAAAAAATATAGGAAACCAGAGTTAGAGCCTTTTTTGTACAAGAGCAAAG
TTGTTGGATAACAGACCATTTTTATTGTGAAACTCAATGGATGCTCGCTTCGATTTTTTAAAT
AATTTCTCCTTATTCTTCTTTTTCTGCTTTGTTTTTCTCATAAAACATCTGTAGAAAAAGATGA
ATTTTATATATAACTAATTATAATCGTTTTAAACGAGAGAAAAAATTTCTATATATATATAT
ATATATATATATAATATGCTTGGCATTTAAATATTCGAAATTTTTTTCAAACGTTTTATCACG
ATGGCACCATCATCATAATATGAAAATTAAAATGATTCGATGATCCTACATATTCGAATAT
ATTTAAAATTTTCGTCATATTTTAATGACACCGATAATTTGATATTTTTTAAATTAGAAATAT
ATATATATATATATATATATATATATATATATATATATATAATGCAAAGAAAAGTGTTGTAAAT
ATATAAAAAAAAATTTAGTTAACGGATCGATTTATTAACCTTTGGTGCCTTTGAAAATGAATC
GTTGTGAACTATTGGGAACCAATCGTTTTTAACAAACGATCCTTTTTATTCAAAATATTACG
AAATGATCTATTTATTCCGCAAAAAAATTGTAAAACTAAAAAAAGGCATCGTACAATTTTTT
GATCAACTTATAGAAAATAAGACTCTTCTTAAATAAATATTATTTATTTATCATTTAAAATG

AAAGAAAAAAATAAAAGATCGAACATAGATTTTCATAGTTTTTCTTGTAAAATATTATATAA
AAATATAAAAAAATAGTTAATATTTTGTAATATAACCAAG

226. >MSTRG.4703.4 Gene=MSTRG.4703
CTCTAGATGGCGTTTACTTATTTAGGTTATTTTATTATAATGACTTAATTTGTGTGCAAAACA
GTGTTTAAATATTTTCAATTTAATTTTTATTGAATTTTTAGAAATATACTGTGTAAAATTAAA
AAGTTAATATAAAAATAAATACGCCTATTGATTAAAAATCTTATGTTTATAAACAGATATA
AGATCATATAGCACAGAATATCGTTGAGTAAAGTGGAATATTCCCTTTGCCCCTGTGTTGTT
CTAAGTGCCAATTTTTCAATTCTATATTATGTTAATACTAACATGGATGCGTTAGTGCAAAA
AATAATTTCAGACTCGGGCGATGAATTTTCAAAAAATATTATTGTCTTTCAGGAAAATTATG
GAAACCGAACAGTATTTGAAGAACTCAATAATGATCAATTACGTGAAAAATTATGGGAAA
CATTGTTTCATTGTCTATCAGACAATTCACAATCATCTCTTCATTATAATTGTTTATCCACTTT
GCGGATACTTAGCAAAGCATTAAAACTTTTCTGACACCACTTTGTGTTATTGCTTTAAATGT
AGACCTAGCTTTTGCTATTATTATTAAGTAATTCTATTGTTTTTCTCCATTAACGTAGCAGAG
ACAAAACAAAGTTAAACGAATTAATCACAGACGAAAGATTAGGTATTATATTAAATAATG
CAGCTCTAAGGGACATAAATATAAATCTACGCACCT

227. >MSTRG.4721.1 Gene=MSTRG.4721
TCTTGTTTCCTTTTTCCGTTGAAGAGGTATTACAACAAAATGGGTAAGGTTAAGTGTACTGA
TCTGAGGACAAAAGATAAAAATGAGTTGCTCAAGCAACTGGAAGAACTCAAAACTGAGTT
GACAAATCTTCGAGTGGCAAAAGTTACTGGTGGTGCTGCATCAAAACTTTCGAAAATCCGT
GTGGTAAGGAAAGCAATAGCTAGGGTATATATTATTATGCATCAAAAGCAAAAGGAAAAT
TTGCGTTTGTTGTATAAAAATCATAAATATAAGCCTTTGGATTTAAGGCCAAAAAAAACTA
GAGCTCTTAGAAGAGCATTAACACCTTATCAAGCCAACAGAAAGACTCTAAAAGAAATAC
GCAAGAGATCTGCTTTTCCTCCAAGGAAGTATGCTTTAAAGGTGTAAATTGCAAGGTATTG
AGGGATAAAATAAAGGTACTTAATATTCAAGAAATAACAGAAGCTGTAAAAATTTAAAGA
CGAATGCCAGGGTTTCTAGAAAGCACAAAGTTCAGAAGTTCATCACTATCCTCACAGACAA
ATGGTTTTCGATGATGGCAAGCTACATCATGCCATTTAATTCCATCATTGTAAAAGTTATTC
AGAATTGATAAACAAGATTCGTCGTTACCCTGCAATATAAATTCATGCATTAAAAAACTAT
TAAAAATAAAATTTGAATAAAGCAAATAGAAAATTCATATATATATATATAAATGATTCCA
GTTATAATTATAAGTGAAATTATAAGTGAAATTGAATTAAGATAAATTTTTTATCAATATTA
TCTAAAACTAATAAATATTTCAAATTTTCATTGCCATTAATCGTTCGAATTTATTTGTTGTGC
GTATGTGTTGTATTTATAAAAAAATAATAAATTACCTGAGCAGCTTCACG

228. >MSTRG.4757.2 Gene=MSTRG.4757
ATTATTTTATTTTTGTAATCAATACAAAATTCGTGCTTCGAATATAACATCAATGTATACAA
GTATGTATATATACAAAGTCCCATTTGTATATTGAGAAATAGGTTAAGAGTAATAGGAGGG
GCGTAATTATCGGATGTATGGCTCTGGCGTCAGTCTTTGTTTATCAGTCGTGGAATTTACATC
GCGTTACATTTCGTTCCATTTTTAATGAAATTATATTTAAAATATATCGTAATTAATCAGTGA
TTAGTGTAGTAATATGATTGAATGATTAATTACTATTTCGTATACAGGTGAGACTTGCTTTGG
ACAAGACTATTTCCGTATATCTTTCTTTAAGAGGAACTGATTTGCAAGCGAGAAGACAGTC
AACATTCAGTGGTTGCCAACCCCAAAGAATTGCGTCTGTGATCGAGATGGAGGAAGAAGA
GATGAGATCCAGCGGATGGATAAGATTATGCTGTCGAGGTGGAAATACTCCACCGCCGGA

CCGACCTGCCGAGTGAGTAATGATCATGGAACAATCGCCCGCATTTAGATCATTACCAGCA
TCGACGGTCGATCCTTCTCGCAACATCATACTAAACAGCTGTTTGACTGGAGCCTTTTTGGA
CTCCTCAAATCTTGGCCAGAAGTTTCCAAATTCCTCTCAGATCATGAAGATGGACTATGAAT
TTTTTTTTAGATAATGAAGATATTTAATCAGCTTGAAGTTTAAAAATTCAAAAATTGAATTTC
AATTGATGATTTAATTAATAATGGATGATA

229. >MSTRG.4809.1 Gene=MSTRG.4809
GTCTCTTGCTCGTTCCAATAATGACGCCCTGTTCGTCGCGTAATTTACGCGTTTTTGCCCCGA
TGGAATGTTGTACCCGCCACGGTGATCGGTTGAATTTGTGGCTGTTCAAACGTGGCCTCCTC
CCGTGACATAGTTCTCTCTCCATAGTGGAGAAGGGAAAGAAGGATCGAGGGAAAACTCAA
ATTCCCACGCGCACGAGAATTATAGAGGGCGCGTGGAGAGAGAATCACAGAGCTCGTCTC
GAACGGCCGCCATTTGTACGAGATGAAGTTATTATTTATGGGATACGGGGACTTTTTTAACG
TTTAAACGTGGCACGCGAATACGTCTCGACAATAATTGTGTAATTAGCATAACCAGATACG
AGGGAAACTTGGATAGTTTGGATAAGTGCGCTTGAGTGGTTCGCAGAAGAAGAGACTCGTG
AAGATTTGTGAGGTTAAATGGTAGACTTTGCATCGTTACGTTAAATTCGGTAGTGATAGTGA
TTGCGTGGCTGCGTTCAACTTTGTATCATTTTCGACGTATAAAATTGAGATTGAGATTGAGA
GATTAGAAAAACGCGATTAGAAGAAGAGTTAATTTGAAGAATCGAGGAATTTCTTTTTTGA
GGTTAGAAACGTTCATCTTTGAGAAATTTCTAGTCTCGTGATTCTAATTTTGTGATTCGTGAT
TATTATTCTACATTGAAATTGAATATCTTAATTTAATTTCCACGTTTATATCTTTACTTTTCAA
TATTAATTCGAGATTTGATGACAGATTTCTCTGGTATCATTCCGTTCCTGTACGACGATCAA
AATAATCTTAACGCCATTCACCATCGTACCCACGAGCAAAAGCAAGCAACGATTATCGATG
TTTTCGATCGTGAAATATCATCATCTCTGACATACCTTTCATTCGTCAAGGGCTTGGCCAATA
GCCTTGACTAGTTAATTGCCCAGCTACGCTCGCCCATGTTATCGTCTGTTATCGATTGATAAC
ACGTCGGCCGCCAGTGAGTTAACTGTAACTTGATAGATCGTACTTCATGGATCACGTTGGTT
TTCCAACGCGATCGACCCTTGGGCGATCTTGGACAGGATGTTCCCATGCCGGTTGACGTTTA
TTGCCTTCCTTTTTCGCTCGTTCGCCGAGAAATCAACGTTGCATTACGCGCATGCTTGAATAC
CAGATCCTACGGATGCCGTGAACTTGGATACGTGTATCTGCTTATGTAAAATATTAATCACG
CGTGAAAATATATTTCGAGGGAAATAATCTTGTATCGCTCGTGGATCGTGTTTTTTTTTTTCT
TTTCTTTTTCTTTTTAACGAATATTGATTTCTGATCGAATTATACGAGACAGTGCGGCTTATTT
TCGTTCTTTTTTCTCGTTAATCGACTGCTTGGAATATTCCTCGTATAATATCTTTGATCACTTT
TTTTGCTTCGATATTGCGCTATTTCGGGCTTTATCGAATTTGAAATTATTAAAATATAAATTT
ATCCTGCTGTAATATATGGTTAATTCGTTCCGTGTCGAGATCGTATTAAGTGAAACGTAGAA
CTAGAACAGAAGAATGTCAACAAAAACAGCGTGATGAAAACATTCTTACTAGAGCGGATC
TTCTATGCCGTTCTCCACCGACTCGAAACGGGGGGAGAGAGAGAAAGAGGATTCGTGCAT
CAAGATCGGATCGATTCTCAAGAACGAGAAAGTAATGGCCCATGAAAATTCACGAGGTAC
GGAGCAGGGAGCGATAGGCTGCGTGTGAAGGAATATCCGTGCGTCGAAAAGGACAATTAG
ACCCTTTATCCGGCCAGGTGAAATTCGAGGATGGAAAAGAGCGGTGATGCAGACGTGGAA
GGTGCAGTCGCAGTGAAACGACCGAATAAACAGCGGCGGATGGAATCTCGAACTTTTAAG
AGTTCGAAATTCTCGAACAACGAGCAACCGAATCGAAGCGTGTCAAACCGAGAGTGTCGA
ACAAAGGTTCGAGCTGCTGGCCTATAGGGAGATAAAAAGATAGAGAGAGAGAGAGAAATAA
AGAAAACCTCGCTCGATTCCACCCTCCCCCCCTCCAACGCTTTATCTTCCTTTCTAGAGCCG
ATTCGAAAGGCCTTAGGCTGCCGGTTCGATTCATCGTTCCATCCTCGAAACCGAGGGAGAG
AGAGGGAGAGAAAAAATCCCTATACACCCGATTCGTAGATTGCGAGTCTCAAGGCGACTT

CTCTCCTCCGCTGGTACAATCGCGGTCGGTCGTATATAAGCCAAGATGAATCCCCCAGCCA
TCGGTACGCGTAATGTCTTCATTGAAGACGTTGCCTCTCCTATCATAATCTCGCTATGATGG
ACGAGCGGTCTTTATGCGGGGACGCGGAATGGAACGAGATTAAACGAACGGTCATCAATA
GCGTGAAAGGCTTGGTATATCCCGCGTTCGAAATGGGGAAAGGGGGTGGAAGGGAAGGGG
CCGGGCGGACGACTCAATTAGTTCTCGAGTATAGTTCTGTCCTTCTCCGATCGCGGCGCGAC
GCGTGAAACTCTTTTCGATGTTGGTGCGCATGCGCGGTTTGCAGCGGTGACAAATAACGGT
GAGGAAGATTGATCGCGCCTAACCTAGACGGAGAGTATCGATGGAGAGTTTTTTCTTTTGTT
ACGTTGGATCTTCTTCGTGGAATAGAATTGATCCGTGTATCTCGTGTACTTTGAAAAGAAGT
ATTCCGAGATTTTATATTGTTACGCGTCGCATCGAAAATCCAACGAAAGGATAACCTCGTTT
TATTTCTCTTGTCCGCTATTACGAAACGTGTGGGAAAAGGATTCCTTTCGCGCCTCGATTCA
AAATCGCGTCTATCGTAACAACGTTGCAACGCACGATCGTATTTTCGAAATTCGAAAGAAA
GCGTTCCACTTCTCGATAATATCGAAGAACGATGTCTAGACTGCATGATAAATATAACCTG
AATACTTGTTATAGACGAGAGAA

230. >MSTRG.4810.1 Gene=MSTRG.4810
ATTAGCCGTACGGCAGCCCGAGCAAAAAGCTACACGACGGAAGAGGTAACGGGCGACGT
CTGAACGCGACACGCGAGTGAATCGCGGCCTGCACGTGGGAGAGCAGAAGCAAATAAAG
TAACGAATAAAGAATAGAGTCTACTACTGTGTGCCAGTTGGACAAGCCGTGCAGCATCATT
AGCCGCGACGTCCAGCTTCTGATTTCCTTCGTTCGCCTACCCGTCGTCCCAAGGCGAAACCG
AACCGACGAGATGCGCAGCCGACATTTTGTTATGTTAACGCGGGAATACGATTTGCTCGAA
GAGAGGGCAACGTGCAGCATCATTAGCCAGGACGTCGAGGACTCGCGATTTCCGTCTGCTC
GTTTGTGGGACGAGACGTTCACGAAGGTAGGGAGGAGAGGGAGGAGGGGGGAAGGGCTG
GCCGCCCTTATCTGGCGAGGGGGGGCCTCCGAGTGAATGGTACCCGGTACTTTGTTCTCGGT
TCCTGGTACAACCGGTGCTCTCCTCTCCTCGACAGATATTCGAACATTCGGGTTCGATGAAC
CCTGGTTCGAGGGGGCCTTCTCCTCCCCTCCCTCCCCCCCCTCTTCCCATCTCCCTTTCCGTG
GACACTGGAGCGGTGGCCAGGGCAGCGCCCATCTGCGAAACGACCGACACCGCCGTTATC
CATCTGCCTTCGGGATTCCGCGATTCGTTAACATTCTTGCCGAATCGCTCCCTCCGACCGGG
GGACCGTCGAAGAAGTTGAAGATCGTCCCGATTCGATGAAGTTGGGTGCGCGGTTTTTTGCT
GCCAGCTCCCTTTGCGACGATTCTCGTCGTGATTGATATATTTTTTTTTTTTAGAAAATTGAA
TTTTTGGACGCTCGATGACGAGGAGAAATTGGGAAGGAAGGGATAATTTAGTTATTATTTG
GTGGAGAATGCGAGAATTGGGATGTTTGAAATTGACGAGTGATTTAGATTTCGAAATTAAA
AGATTTCTTGAAAGGAGAGAGATTCGATATTCGAAGAATTCGAAGTAATTTGTGGATTAAT
TAGAAATATGGATTCGAAGGAATTATTTTTGAAAAATTGTGGATAATTTTGTTATTAAAAAA
TCTATCCGTGTACGAGCGTGTAGGATCGATGAAAAGATCCTTCAGATCCTCGAAGTCTTAAT
CGAAAACATACGAAGTTTCTCCGGCTCGTAAGAGAATAAACAACGAGTTATTGCTGGAAA
GATTTATAGAGAGATGGTAAATCAACATGGCTAACGTTAGCGCGCACCAAGCACCACGTC
CCTCTCTCCTTTCTGTAAATCATTTCAATCAATCTAAATCATCTAGATAATTTGCCGCAGATT
AATTTCAACGAGGATTATTATTACTTCGGTCAATTATCGATTCGTGCATAAATGTCTCTCGC
GAAGATAAATTTTAAACCGAGGAAATATTTTATAAATAGCTTCGAGATAATTAACACGTAA
TATCACCAATTCCACCAGTTTTTCATTTTCATTTGACGGGATCATCGTCTCGATATTTTATAG
TTTTTCTAATTTTAAAAATTATCCGCTTTCGAGGAAAATATATATACGTACACCATTCAAAA
ATCCTACAATCCTACATCCTTTTAACGAAACTATCATAGATCATATTCTTTAATATCAACAC
CTCTCCTATTTTTCTTTCTTTCGCAACGTTAAAAAATATGTAAAATTACGCGTCGTTGGAGTA

ATATCGAGGAAAAAAGAAAATAATTCGAGTAAAGAATCTTGTGGAGAATGAAAGCTTTCA
CCCGGATATATCTCCGCCCGTAAACCACGAGCCGAGGGGACTGGCCTATTGGCAGATCAA
AGCGGCACTTAGGGTTAATCGCGGAACGCTTTTTCGTGGCAACGATGTTCGCCGATAACTTC
GATGCCATTGAATCGTGCGCACGGATCCGGCCATTAACCGGCCAACCGCGGGAGAAAACA
TACTTTAGACATTAACATTGCGGCCGTTTCGTAGCGAGCGGCGTTCCCTCCTAATGGCTTAA
AGGACAATGAAAGGTCGAGGTGAAACTTTATCAATGGCGCGCCTTTCGAACCTTCCCTCGA
ACGTAAC

231. >MSTRG.4869.1 Gene=MSTRG.4869
CTCTCCGCCGATATCCACGTTCCGCAGTTTCTGCCAGCCGTGTTCTGTGTTCGTCTCGGACCA
AATTGGAGGCTGGCTGCTCGTGGTCTCCTTGGAATACATTCTGATCCCGGGCGACCCTTCGC
GAGGGCGACGTTCGCGGAGTGACAAATGGTCCGGCCACGGCCGTTATTTCGTGCTCCTCCT
CCTCCTCCTCCTCCTTCTCCTCTCCGTCTCTCTTTCTCTTTTCTTTCTTCCATCTCTTCCCTTTCT
TCTTTTCTTTTGGTTCACCACACTCCTTCCCGCCTCTTTTTCCTCGGACTCGCCGCCGGCAGA
GGAACGATTCGGCTCCGACTGCACAGCATCCGTGTCCTTGCATCCGGGCCCTTCCAGTTTCG
CGACGTGGCCGTTCAACGGAATGGAGGAATGGATCGAGGTTGATTCCGATCCCCTTGGATT
TTTCTTTCTTGGATCGTTTGTTACATCCTTTGGATCGTTTGTATCGCGTTTTTTGATTTGGTTTT
CTTTTTCTTTTTTCTATTGAAC

232. >MSTRG.4871.1 Gene=MSTRG.4871
TTTGTAATTACTTGCTAAACAATTAAGTAATTATAAAAGAAATCGTCTTCTTTTCTTTAAATT
AATCAAATTATTTCTATTTTAATATAAATGTTATTTACTGTTTTTTCCTTGTGTGTGCAAATGT
TCGCATTTTTCATGATTGTTCTCAGAGTAGTGAAGCATACGAATCCGTCGTATCTCGTCTTTT
TGAGAGCATAATATCTCATATTCAAAATATCTTCAAACGCGTATTTTTGGAGATTAAAAATT
ATTTGACCGATAATTCGTATAAGCATAATTTATGTGATCATTATCACACCTATGCACTTATTT
TTTTTCGAGCGAATCATAAAAAATGAAAATGATCCTCTCAGAAATGTGAAATCGAAAAACG
AAATTGAAAAGCGTAAAGGGATTTTATAGGAATTACGCGATCATTGCGTGAAAAACGCAG
TTACAGCACGGTAGACTTCCTGTTGTTGTAATACTTCATTTGTTGATGCGTTTGTTCTGAACG
CGCATTAGGAAAAAAAAAAGGAACGATATTAAAAAAAATAAGGAAAAGAAAGTAGTTTG
ATGAAAAAGCGCTAAAAAGAAATATAATATGCTAAATTGCAAAGAAAGAAAAACGGAAA
TGTTGATAATTTAGTAATGCGTAATTTTAGTCGTTTCTACGAGACCAGAAAGTTGAGAGAGA
GTTCAGATTGCCGATGGTATCGGGAAAGATGTACATTAAAAATAATAATAAAAAAAAAAA
ATAAAAAAAAATAAAAAAAAATACAAATAGGTATTCTGTAATTACGGCTTTATTGATTCAT
CGCGCCTTACAATATGAAAGAGCTCGGCCAACTTTCATCAAGATGTGTATGCAAAATTGCG
AACACTCTTAGATTCGAAACGATAAAAAGAAAAAAAACCGCTCCTTGGTCGTCCTCGAGTA
AATTGAGACAAGCGTTTGCTAGAATTGTTCGAACTGCAGCACAGAAGAACGGCATATAACT
GTATTGCATTCTTGTTGAGTATAAGAATATGCTAAAGAGGCATGCAAGAATATATATTTGCG
TCAATTTTATGTATAAAAATGACATTTATCAGTTCTTTATATAAATATATATATGTATATAAT
GTATGTTTGCATATATATATATAAATATATACAGACATAATTCTAAAAAAAAAAAATGGGAT
ATTCTATTAGGGCAATGCTGTCCATCAAAATTGATGATCTATTTAGCGCTTCTTTAATATTGT
TATTAATAGATAAAAGAAGCTAGAAGATTCTAATGAAAAAGTAGTTACGCATGTTAATATG
TAGTTGTTATTTAATCATGTTAAAGCCTGTGCTATAACAAAGGAAAAATAGAAAAAAAAAA
GCTAATTTTTAAGTTTTATAGCTTTAATTTAGACAGCATTGTTTTAGTAGATAGAATATTCTT

GGTGAATAAAAAAAGTGATCGCTTATCACATTATTAACAGATTCACTGCTACTTTATTTTGA
TAATTTATCACTGTACTAAACTGTAAAAAAGTACATTTAAAGAGAATAACGACAGTGTACA
CTTATCGCAATAAACTTGGCAGTGAATATGTTAAGATGACAAGTAATCTAGTCGAAATTATT
CTGTCGTTAATTATGACCCTTGAGGGTATAAAGATATATTGAAACAAATATCTTTTTTATGG
TATAACAATATTGGAATATTCTTCAAACATTGTTGTACTTAACACAAAAAATATTTTCAATT
ATTCAAATATTAATTTTTAGAATCCTGACGCAAGAAAAAAGAAAAATTAAGATCTCTAGTG
TTTTTGTATTGAATAATGTTTAGACTCGACATGAAAATGTTATTTCAAAATGAATATTTTAGG
AAAACGAAAGACCAGTATTAACAGTACAACTAGAAACAAGATTATGTGAATACTTTGTCA
TCGTTTTGCTTTCTGTGTTGTTCTCCGTCTTTGAAATAATTAAGTAAATGCAGAAAATACCTA
TTGTTGTCTAATCATGTAAGCCGTTTTACTGCAGAATTATTAATTTTTACGACCAAAGAGAG
GTAATGCGAAAATGGCTTATTTTTTTTCTTTGACCATGACACGATTTTCAAAAGCGGAAAG
TATATCAATAGTTTATATGAGATGTGAATATTTTAATGCAATTTTCAGTGAGTGATAGAAAA
CAAGTGACGTTTGTTAAAAAGTACCTTAGAGTTAATTATTGTATGAGTCGCTTCTGTTCAAA
CAGAATTTATTGTTTTTCCGGTGTAGAAAGAAGAAAAAGAGCATACACAAATTATTTATAT
GCAGCGCGCAAGGCGAACAATTTAAAAAAAGGCGTAAATAATGTTAAA

233. >MSTRG.4928.1 Gene=MSTRG.4928
AAAATCGATAATAACGGTTCATTTCTGCGCACGCATATTCGTTACAAACTAATGTTGGTACG
AATACATTGTGACACATGACACATGATGTAAAAAAAATTAAAAGATCCACGAATTGATTTA
AGCCGGTTTTAGTAAAAAAATTTATCTTAAAAATGCCTGTTAGTAGTAGAGTATCTCTTGTA
TTGTCATCAATTTTGACAGTTCTTATATTTTCGGGAATGCAAATATATAAAGTTTGGTTAGCA
TCATCACAAATTTATACAATATTAGGAGGATATATTGGATCGATATTATTTATGTGTGTGTTA
ACTGCAGTAGGAAACTTGGAATCAACAATATTTGGAAAATCTTTTCAACAAAAATTATTTC
CAGAAGTTATTTTTTCACTAATGATTAGTTTAATTGCATCAGCTTTGGTACATCGTGTAGCAA
CTACAACATGTTTTATATTTTCTATGATTGCATTATATTATATCAATAGAATTTCTCAAGAAA
CATATGCTCTACCAGTACCTCCAATTTCAATTCATATAAAGAAGAGAAAATAAAAATAATT
ATGAAAATCTATGATGTTTTACAATAATTCAAAAATGTTGAATTATTCCTAAAGTTACTTTA
AACTTTATGTAAAATTATTTTGTCTTATAATATATATAATATATATAATTAATGTTTAA

234. >MSTRG.4928.2 Gene=MSTRG.4928
ACTTAAAAAATTTTTGCAGCTAATGTTGGTACGAATACATTGTGACACATGACACATGATGT
AAAAAAAATTAAAAGATCCACGAATTGATTTAAGCCGGTTTTAGTAAAAAAATTTATCTTA
AAAATGCCTGTTAGTAGTAGAGTATCTCTTGTATTGTCATCAATTTTGACAGTTCTTATATTT
TCGGGAATGCAAATATATAAAGTTTGGTTAGCATCATCACAAATTTATACAATATTAGGAG
GATATATTGGATCGATATTATTTATGTGTGTGTTAACTGCAGTAGGAAACTTGGAATCAACA
ATATTTGGAAAATCTTTTCAACAAAAATTATTTCCAGAAGATCTCATTGTGGTTTGAGCTAA
CTGAACTACAGCAAATTCAGGATCATCAACACCATATGAAGCCAAAAATGGATTAACTAC
TCTTAAGTATAATATACCAT

235. >MSTRG.4933.2 Gene=MSTRG.4933
ACAGGATCACAGTACGGACACTTCAGTCTTGAACTCAATCAAGGAATAGTCGCTGTCGGCT
TGAGCGAGCAAAATTTTTGTGTAAATCAATTCACATAGAAAAAAATAAGTATACATAACTT
CCGGGCCAGGATATAACGATCTTTTTCCTTGTGTATTAGTTGGTGAAAGTGTTATTTTAAAA

GAGCTACGAAAATGTCAGACAGAAAGGCTGTGATCAAGAATGCAGATATGTCTGAGGAGA
TGCAGCAGGATGCTGTTGACTGTGCAACTCAAGCCCTTGAAAAATATAACATAGAAAAGG
ATATTGCAGCCTTTATTAAAAAAGAGTTCGATAAAAAATATAATCCAACGTGGCATTGCAT
TGTAGGTCGTAATTTTGGAAGCTATGTGACACACGAAACAAGACATTTTATCTATTTTTATC
TGGGTCAAGTAGCGATTCTCCTTTTCAAGAGTGGATAAATTGGAATCTATTTTGTAATCACC
ATCCTTTCTTCCTCCCTTCACACATGCTACATGGAAATACCTTAAGTCACATCACTGTCTGTA
AGAGAAATTACAATATCGTGTATAATGAAAATTTGTTTTGCAAACAAGATATGCACTCCCA
GTTAAACAAAAAAGTATAAGTATATAACAACCATCAATACTATTGACTTTGCTGTTTTACTT
ATCTCAGGAGATAAGACAATGATGAAAAATTCTGTAGAAGTTATGGGGAAAAAGGGTAGG
GAGATGATTAACTAAATGAGAAGAAAATGAAAAAACTCAAAACTGAAAAAAATCAATTG
ACATACAATCTTACATGTTAAAATGTATCATGTATGCCAGAAAATTTGTTGTGTTCTTGTTTT
ATATTAGGTATATATAATGCGAAGTTGAGTTCAATACTGGCGATTTTAAATTAATAAATTTT
TAGTGTTTAATACACTAAGTGCATCTAAGTATAATGTAATGTTCATACAAATTCATGTATGA
TTTTTATTATCTTACTATGCTTGAGGAAACTGTATCCAGTAGTCATCAATAATGAATGTAAG
CCAAACCTTGATTGATTGCTGCGATCATTATTATTTTACGTAATGTTGTAAATTATCCCTTTA
TTCTGTGATGCTATATAAACAAAAACGGTTACAAAATCAACTTTGATTCGTTTGAATCCTGA
CATGACAATTTTTACATCTATAAAATTCGAGAATTGGAGGTTGAGAGAAAGCTTAATTATC
GATACGCACCGAATTTATATTAAATAAAATTATTTAAATTTACACAGCACTAAGTAAGATG
TTGCATAATATTGAAAGCGAGTCATTATCATAATTTTAATTTTTTATATTGTGCACACTGAAT
TTTGCAAATGTATTATTCATAAGTCAAGATACATTTAAGGAATTTTCTTTTCCCCTCTTATTTT
GTTTTTTGTTTTGAAAAAAATAGATATTCAGCTCATTAAATTCTATCCTTTATGTAACATCTA
ATATCCAATATTATGGTACTTATATATATATATATTAAAATATAGATTATACATATATATTA
AACTATTTATATATAGTGCAATCTTCATTTTCAGTATTTGATTATCACAACGTTGCTACATTG
TTCAAGTTTTAGACTGATTATTAAACATTGTCATAAATATATGACTTGCATGGTTCTTTTTAT
AAAACTTCACAAGTCATTTTTATAATACGCATATGCAACATTATTATAAGAATTAAAGAAA
TGTTACATTAATATCAAAAAGCAGAAACGAAACAAAATTAATCATACTTTTGATTATAGCA
AATAGTCTCAAATGTCAGTGAATATTTTTCTTTGATTATCTTTGATATACATTCCCTTTCGCGT
CTTAACTTATATAAACGAATGAAAAATTGTTCTGATAAAGAATACAAAAAAAACTGATAC
ACTATTTAGTGTTGAAGTTTGTTAAATAAGAAAATTGAAGTACAGTTAATATTATGGGAAAT
TATGCTCAAATCAATTTATGCATTATTGATAAATTATGAATATATATATTTGTTGTATTATCA
AAGATGAAATAAATTCATAAACTAATTCCCAAATTTTTTCATATCAAAATTGTGTAATTGTT
GAAAAATAAAGGAAATGAAAGCACTGCAATTTTATATGACAAAAAAAAAAAAGATCGTAT
GTTATATTTAAAAATATATTTAAAGAAGAAAAAGAAGAAAAGAAAACAAGGC

236. >MSTRG.4983.1 Gene=MSTRG.4983
TTAGAGCCATTCTCACAAGTACTTTTGAATATTCTAGTATATAATAGACGCGTTTCAAACCG
AGGTAGTTATCGCTTCTCGGCCTGATGGCTAAGATCAAAGTGTAGTATCTGTTCTTATCAGC
TTAATATCTGGTACACTCCCCACCGGGAGTCAGAATATTAGTCTGATTTTTGGAACCGGGCG
GAACCCCGGGGCTTGCTCCGCTTCTGCCGCGAGTCGGCTCGGCATTGCAGTGCCGTCGAGA
TCGGCTCAATAAGTTACCCCAAAGATTAAAGAATTTTTCATATATTCTAGGAAATTAAAATT
TAGTATTTCATGAAGTAAAATTTTTTTATTAGAATGAACAGGGAGATATGTAGGAATAAAA
ACTTGTTATTAATGTTCAGTATCTGTTCTTATCAGCTTATCTGATACACTCCCCATCGGGAGT

CAGAATATTAGTCTGATTTTTGGAACCGGGCGGAACCCCGGGGCTTGCTCCGCTTCTGCCGT
GAGTCGGCTCGGCATTGCAGTGCCGTCGAGATCGGCTCAATAAGTTA

237. >MSTRG.4983.3 Gene=MSTRG.4983
TTATCGCTTCTCGGCCTGATGGCTAAGATCAAAGTGTAGTATCTGTTCTTATCAGCTTAATAT
CTGGTACACTCCCCACCGGGAGTCAGAATATTAGTCTGATTTTTGGAACCGGGCGGAACCC
CGGGGCTTGCTCCGCTTCTGCCGCGAGTCGGCTCGGCATTGCAGTGCCGTCGAGATCAGCTC
AATAAGTTACCCCAAACATTAAATAAGTCTTCATATATTCTAGGAAATTAAAATTTAGTATT
TCATGAAGTAAAATGTTTTTATTACAATGAAATGGGAGATA

238. >MSTRG.4983.2 Gene=MSTRG.4983
TTATCGCTTCTCGGCCTGATGGCTAAGATCAAAGTGTAGTATCTGTTCTTATCAGCTTAATAT
CTGGTACACTCCCCACCGGGAGTCAGAATATTAGTCTGATTTTTGGAACCGGGCGGAACCC
CGGGGCTTGCTCCGCTTCTGCCGCGAGTCGGCTCGGCATTGCAGTGCCGTCGAGATCGGCTC
AATAAATCTGTTCTTATCAGCTTAATATCTGGTACACTCCCCACCGGGAGTCAGAATATTAG
TCTGATTTTTGGAACCGGGCGGAACCCCGGGG
CTTGCTCCGCTTCTGCCGTGAGTCGGCTCGGCATTGCAGTGCCGTCGAGATCGGCTCAATAA
GTTA

239. >MSTRG.4984.1 Gene=MSTRG.4984
TCTCCCATTTCATTGTAATAAAAACATTTTACTTCATGAAATACTAAATTTTAATTTCCTAGA
ATATATGAAGACTTATTTAATGTTTGGGGTAACTTATTGAGCTGATCTCGACGGCACTGCAA
TGCCGAGCCGACTCGCGGCAGAAGCGGAGCAAGCCCCGGGGTTCCGCCCGGTTCCAAAAA
TCAGACTAATATTCTGACTCCCGGTGGGGAGTGTACCAGATATTAAGCTGATAAGAACAGA
TACTACACTTTGATCTTAGCCATCAGGCCG

240. >MSTRG.5001.1 Gene=MSTRG.5001
CTTTTCTTTCTCATTCCTTCCTTCTGGACTTTTCTTTTAACAGCGAATAACTATCGGCTATTCC
ATCTAAAAGTAACGAAAATTTCGAAACACTTCCTCTTTTTGACATGAAAAAAAAATATATA
CATAGAACTAGCCAATAGATTATCCGTCTAAAAGTAACGAACGTTTCGAAACACTTTCTCC
TTCTTCTTATCTGACGCGAAAAAAATATACGTATAACTAAAAAAGAAAAGGGGGTGTGTTT
TTCCATCGAAGACGGGGAGACTAGAAAGAAAAACTCACCCTCGTTCTCGTTGTTACCGATC
AGTATCTCTGTGTCCTTGAAGTCCGCCTCCCTCAACAGATCGAGAGGATGCTTGGGAAGGA
AGATACCGTCGATGGTGGGCGCCGAGGGGAAGCCTAGAATCCCCCAATAACTGTTCCACTG
TTGAACGGATATGGTCTTCGCGTCCACGGACCTCATACACGCCATCACCCTGGCCGGGTTCT
CGTTCAGCATGGTCGAGTTGCATCCGCAATCGTCGACCAGTATCGTCGCGACCTCGTTCGCC
TTCTCCCCGCTCATGTAACTCCAGGGAGCGTTGAGGGTGCCGCTCTGCAGTATGCCGCGGC
GCACCAGTCCCCTCGTCACGGGCGAGATGAGGTGCAGGGAGACGGAGCTGCCCCCCGCCG
ACTCGCCGAATATCGTGATCAACTCCGGATCCCCGCCGAACGCCTCGGCGTTGTCCCTCAG
CCATCTAAGCGCGAGCGCCTGATCCCAAAGACCCATGTTCCCCGGCGCCTCCTCGCTGTTC
GTGAAGTGTTTGTTCAGATAGAGGAAACCGAACGCGCCCACCCTGTACTGCATCGATGCTA
TTATAACGTTGCTCGTAGCCGCCATTATGTCCGCGTTGTACACGTCCAGGGTTGCCGTGCCG
CTCATGAACCCACCCCCGTAGATCCACACCAGTAAAGGGAGCAAACCGTTCCTGGGACCC

CCGTTGCCACCCGGCGAACCGTCTCCCTTGTGCCTCAATCTATACTTCTGCGGCACCCATAT
GTTCAGGTACAAACAGTCCTCGGATATGTTGGTGTTCGGGTTCCACATCTCCTCGCCCGGGA
ACCCGGGGAAATACTCGTATCTCTCCTGGTAGCAACTGTTCGGCAACACGGTCGCGTTCAA
CACGCCGTGCCACGGCTCGATCGGCAACGGTTTCCTCTTTTATTTTCCTCTCTCTCTCTTCTCC
GAAATTCTCTTCTTCGCTCGTCTCTTCTTCTTCTTCTTCTTCTTTTTCTTCTTCTTCTTCTTCCTC
GTCTCGGTGGTTCCGTCGTGGTGGTTTCGTTCGTTGGAAGGATGGAAGAGGAGAGAGAGCG
CTGGAGAATTCGATCACTGGCCGGGGGGAAAAAAAGGACGATTCGCGCGACTGCGTCGCG
GAAAGGCGAGCGAAGGGATCGCGCGCAGATCGAGGCGGGCAAGAGGGTAAAATTTTCGC
GCGGCGGCCGGCGAGCCGCGTTGCACGGGGTCGACTGATGGAGGCGGTAGAGGTGGAACG
GAGTCGAGG

241. >MSTRG.5001.3 Gene=MSTRG.5001
TTCCATCGAAGACGGGGAGACTAGAAAGAAAAACTCACCCTCGTTCTCGTTGTTACCGATC
AGTATCTCTGTGTCCTTGAAGTCCGCCTCCCTCAACAGATCGAGAGGATGCTTGGGAAGGA
AGATACCGTCGATGGTGGGCGCCGAGGGGAAGCCTAGAATCCCCCAATAACTGTTCCACTG
TTGAACGGATATGGTCTTCGCGTCCACGGACCTCATACACGCCATCACCCTGGCCGGGTTCT
CGTTCAGCATGGTCGAGTTGCATCCGCAATCGTCGACCAGTATCGTCGCGACCTCGTTCGCC
TTCTCCCCGCTCATGTAACTCCAGGGAGCGTTGAGGGTGCCGCTCTGCAGTATGCCGCGGC
GCACCAGTCCCCTCGTCACGGGCGAGATGAGGTGCAGGGAGACGGAGCTGCCCCCCGCCG
ACTCGCCGAATATCGTGATCAACTCCGGATCCCCGCCGAACGCCTCGGCGTTGTCCCTCAG
CCATCTAAGCGCGAGCGCCTGATCCCAAAGACCCATGTTCCCCGGCGCCTCCTCGCTGTTC
GTGAAGTGTTTGTTCAGATAGAGGAAACCGAACGCGCCCACCCTGTACTGCATCGATGCTA
TTATAACGTTGCTCGTAGCCGCCATTATGTCCGCGTTGTACACGTCCAGGGTTGCCGTGCCG
CTCATGAACCCACCCCCGTAGATCCACACCAGTAAAGGGAGCAAACCGTTCCTGGGACCC
CCGTTGCCACCCGGCGAACCGTCTCCCTTGTGCCTCAATCTATACTTCTGCGGCACCCATAT
GTTCAGGTACAAACAGTCCTCGGATATGTTGGTGTTCGGGTTCCACATCTCCTCGCCCGGGA
ACCCGGGGAAATACTCGTATCTCTCCTGGTAGCAACTGTTCGGCAACACGGTCGCGTTCAA
CACGCCGTGCCACGGCTCGATCGGCAACGGTTTCCTGAAACGGAGGGGGCCGATCGGGGG
TTTGGCGAACGGTATCCCGTAGAACACGTGCACCTCCTTGTCCAACACGGTGCGGGGGAAA
CCGCGCACGAGGCCGCTGGTCGTCTCCACGACCAGGGGGTCGTTGTGCACGTTGCCCCTCT
GCGAGGAGGGAACTGCGTTGCCCCTGCTAGGACGCGTGCACGAGGAGAGAAGGAGGAAG
AACAGGAGTAGGATCCTCGTAGTCATGTCGATTATTTAGGGGATGATGGTTTGGGGGTAGG
GTAGTTTTCTTTTTTTTATTAGGGAGAGAGAGGATATATATATATGTCTGTGTGTGTATATAT
ATATATATATATATATCCTGGCGAGGAAGGAGAGAGAGAGGCTCAACAAGTTAGCGAGTG
GGGGACCGGCCGTGGGAGCCGGCTTTTGCGCGGAATCCTCCTCCTCCTCTCCGTTCGCC
TCCGCTCTCTAACCACGCCGTGTCTCCCCGTGTCCCTCCTCTCTCGCTCTATCTAATTCACCG
TGACCTTACAACACGCACCTCTTTTATTTTCCTCTCTCTCTCTTCTCCGAAATTCTCTTCTTCG
CTCGTCTCTTCTTCTTCTTCTTCTTCTTTTTCTTCTTCTTCTTCTTCCTCGTCTCGGTGGTTCCGT
CGTGGTGGTTTCGTTCGTTGGAAGGATGGAAGAGGAGAGAGAGCGCTGGAGAATTCGATC
ACTGGCCGGGGGGAAAAAAAGGACGATTCGCGCGACTGCGTCGCGGAAAGGCGAGCGAA
GGGATCGCGCGCAGATCGAGGCGGGCAAGAGGGTAAAATTTTCGCGCGGCGGCCGGCGAG
CCGCGTTGCACGGGGTCGACTGA

242. >MSTRG.5031.6 Gene=MSTRG.5031
ATTATACTAACTTCCTGGGATCATTATAACAAATGCAATCCTTCTGTTGGTAGTCTTAAAAG
GAGTAAGACTGTTCCGCGAAGGGAATAGACCGATTGAAATAATACGTACAGTACACTTTTT
TTTATCAGGCTCAGCTTTAATCGGACGTCGTTCACCATAGAAACAGATTGTGATTTTTCTAT
AGCAATTATTATTTCTTTATATGTTCATATATGATATTGACGATATTATATTATATTATATCTA
CATTTTATAAGTAAGTGAGAAATATTGTAGATATACATATACATATATAATATATATATATA
TATATATATATATGTATATATATATTTATTTATTTATTTTGTTTACAACCTACAAATGATATTG
TTCGATTAACTGACCATTCGTTTATCCGACCAAGAGTCCGATTCCGTAGGAGCCGGTTAAAA
GGAAGTATACTCTTAATATTAATATTTATTTTATTACAAAGATGTAAATTCGATAAAAATAT
AAAACAGTTATATATGAACATTTTGTAGGGATATACGCAAGTATATTTCAAAACATTCAAC
AATGTCTACTATTGAACAATTTAAAGAGTGTGCAAAAATGCAAACTGCTAAAGAACTTTAT
GTTGGAAAACAATATAAAGAAGCATTAAAAGAATATAGTGAACTTATTGAATTATATCCAA
ATAAACCATTGCATTATGCAAATAGAGCTGCATGTTATATGATGCTTGATAAATATCCTTAT
GCTTTGGATGATGCAAAAAAATGTATTGAATTGGATGAGAAACTATACAAAGCATATGTAA
GAATTATAAAATGTTGTCTGATTTTGGGAGATATTATTCAAGCTGAAACTACTTTATCTAAG
TTATTAGAAATTGATCCTGAAAATAAAGGAATTACCACAGAGAAAAAAGACTTAGAATAT
GTGAAAAAGTTTCTTAAAGATGCTGATATTGCATATGCTGCTAAGGATTATCGTAAAGTTGT
ATATTGTATGGATCGTTGTTGTGATGTGAGTACTCGTTGTACAAGTTTTAAATTAATCAAAG
CAGAATGTTTAGTATTTTTGGGAAGGTATCAAGAAGCACAAGAGATAGC

243. >MSTRG.5087.1 Gene=MSTRG.5087
TATAAAATGCAAATTTATTTTAAGTGAAAACAACTTAATTTAATGGAATAAAATATAAACT
GATTCCAAAGCAGCCGTGGAATTTTATGCAATTGCCGTGGATAAAAATTATCGTGGGAAGG
GACTTTCAAAATCCCTAATGGCTGCAGGAATTTTTTTCGCAAAAAATAACATCCCAAATGT
AGGATTTATATTTGGAGTATATACATCTTTGTATTCAAAAAAAGCGGCGGAAAAATTAGGT
TTGAAAAGTGTAATGGATGTGGATCTTTTGAATTACAGAGATGACGATGGACAACCTATAT
TTCAAGATACTCCTCCCCATAATATTGTTTCTGTTATGGTTTTACAAGTTACATAAAATTTTT
CTAAAATATAATTAAATAGATTCATATTTTCTTTGTAAATATATGTAAAATACGAAA

244. >MSTRG.5114.1 Gene=MSTRG.5114
TCGAACGCGTCTTTCGTGGCCGGTATTTTTAAAAAAAGTTCAAGTCACATTGCATATTTCGTT
ACAAATAATGAAGAATTTCATCAAACACTTGTTTTTACTGCTATCTCTAATGGCGATACCAA
TCTATGTATCATCGATGAACATTCGAAGTCCACCACAAAAGAAATTAGCACCGAAAAACG
AAAATATTTCACACGAATTTCTTTATTTGCCTATATCTCTGATAAACGTACCATGCAAACAT
GGATATAAAAAAAGCCCAAAAGATAATTTATGTCGTGAGACAGTACCATCAAACTAATGC
GAAAATGCGTCGTCGTAAATCGTAAAAAAGGCCTTAGACCGCCATCTGTTATTACCTTCAT
GCATCTTCTCATATCGACGAATCGAATTTACCGACTCAGCATAATGGACATAAAGATTAAG
AAATAAGATGTAAATTTAGTTTATGATATAATTGTAATTTTTGTTTTTAAAATTGAAGCGTG

245. >MSTRG.5134.1 Gene=MSTRG.5134
CGACGATTTCTCGACCGATATTTTGGAACATGCGATGCTAGCATTAAAAAATAAAATAAAA
TAAACGCACTCAATCGTTGAGAAGAAAAATGGATAACTGGCGTTAAGAATATTCCGATCG
GCAGACGGACGTCGCGTATTATTAATAACAGTTCTGCGATCGACTTTCAGCTGACTCTCAAA

CACGAACCGAGAATGACAGACACGCGTATATACACGTATATACACACACACACCACGTAC
ACGCGCGCATGCACACCCAAAGACATAATAAGGAGAGGGAGACTCACACAGGAGAATTG
GGAAAAAAAAAATAAGGAGGAAAGATCATAGCTGTGTATGCATATACACCACACTGTAC
ACTAAGATTCTTAATGTTGTAACACTGTAACTCTATTAACTGATTCATAATGCAGTTGTATTC
CATAGTAAGTTAAACAAAGAGCAAATTTAAAAAAAAAAACAAAAAAAAAAAGAAAGGA
AAAAAAAAACAAACACGTAAATTCTTAATAACGATTAAATAACACGAGAGAAGAAGGAA
GAGAGAAAAGAAAATACAATGCGATGTATTTATTTTATTCGCGACGAATGTGAAACGTTTT
GAAAAATTAATCGTTCGAAAGTGAAACGAAAATGAATATCTTGAAAAGGAAACGAACGA
GTTAATAGTAATTTTTCTGCCTAGAACAAAAATAGTAAGATCCTATAGACGTGCTGTGTTTT
ACTATTGTACAGTTGTAATTATCGAAGAGGATGAAGAAATCCACGAGAATTTAATCTAAGC
AACTATTTCTCTCCGAATCGTGTCTATCCGGGCTCGCTCGAAGAACGAATGTCTCCAAACGT
TTGATATTTGGCAACGTCGCACGAGTCACGTGACCCAATTACATCCAATCGTTATACGTCCA
CTGCTTTACCGACTCGGATCGACACGTTTTCGGTGCGGACTCGACGCGTCCCGCGCAAAATT
CCACAAACACGAACGGAAAGATCCAACAAATTATGCCGCAAGGCGGACACTGCCACACC
GCAGAAGGAAGAGAGAGAGAGATGCTTTCGGATAGATAGAGAAAATCTTGTAATGTCATC
GTCGCTTGCATTTCGAATTGATCCGATATTAGTAATAATGTATACCTAACCTCTTCTACTCGA
ATTCGAGGCATTGCTGGAAATCTTATCTTGGAAATTGGACACGAAAATGGAGAATCTGGGG
GAAAAATTGATCGAAAGGATGATTAATAAAGAAAAGTAATCAAGATGAAGAGGATCTGTA
CAAAGACAGCCAGCACGCCGACCTTACATTTGCACACACCGTATATATTTTGCAGACACCT
ATACTTTGTTGTACCACTGAAATCTACACTCATGCAGCTAACTAACAACGAGTGCTTTCGGA
TATCGTCGTTTTTCGCTACATCGGAACGGATGAGTAAGTTGGGTAAAAAAAATTGGAAGGG
AAGTTTTTTTCTCAAGCGCAACACGCATCGCAACGACATTGCCTATATTGCATATATATATA
TCTGTAGTATTTGTCGGTTGAACGTCGTTAATCAATCATAGACGGTTCAACGATAATTAAAA
TACAAAAAAAAAAAAGAAAAAAATATTCGTTCGGGCTCTAGAGTCCTTAAAAAAAAAAAAC
GTTAACGTTTAAGCACGCGAGGAAAACACGTGAAATCGTAAAATAATTCGATACAAATGG
CTAACCTTAAATTGTTAATTGATCGCTTTGTAAAGTTGTTCTTTCGTATATAGAGACTAAAGT
TCTCGATCAAGCCTCCGTTTGGATTGCTCAAGCTCGTTTTATTTTATGAATCGTTGCCTCAAT
TTGATTCAAGTTTGCCTCTAGAATAAATTAAACTTGGTTCATTTTTTACTCGATTGACTCGAG
ACTCGTTTTGGGGAACTTGATCGCTTTCAACGAGGAAAGCGAGCAAAGTACACCCTGTAAA
TATTTGATCCAATCTTAAATTCCAGAGCTTGAACGTTTCAAACGAAGGATTAGATTAATTTT
CTTCCGAATGTTTTCGATTCGACGTCAAAATCGTGCAATAAAAAAGAGGAGGGAAGACAG
TCTGTGTATATGAAAGAACCGTAATTCTCAAAAGAATAATATTGCCTGTATATCTTGGAGAG
AGAAAAAAAAAAAAGAAAAATGTCACGGACCGTCGACGGCAGGTCGTCATCGACACACT
TTTTGTCTCGTAGAACCGTTTAGAACCGCCCTTGAAAGGAGCACTTTCCCCCTTTTACACAC
ACGCGCGTGGACCCCTCTCGCCGTTGATTTCGATTTCCAACGCGCTTCGCGCGATTGGCGAC
AGATGTCGTCGCGGAGATATGCAGGAATTCCTGTATGCGGTCGATCTTTTCTCTCGAACAAG
TTTGAAAGATCCAAGCGGGAATCAAGGTGAACCGCATCCTTCGACGAATCGTAAACCGGTC
ACGCGACGTTACGAGACGGAAAAGGAAGAATGGAGAGAGAGAGAGAGAGAGAAGGAAT
GCGAGATAGTACGCGCGGGGAAGGGAGGATACACGCGTACACATCACATATACACAAACT
ATATATATATATATATATATATAAAACACAGACGTAAACATACATACATACATTCGAACGA
AGGAAAAACTACATGCAATTCTACGATCACTGCCAAGGGCGTTAACCGTTGACGTCGATTT
CTCGTTGTTTAAACGTGTCGTAGTCGTCGTTTTTCTCGAATGCAAAATTAAAATGTAATTATG
AAAAAACGATCGGGAAAAAGAAGAAGGAAGAGAGGAGTGTGAAAGAGTAAAAGGGGAG

ATTACACTACTACTATTACTACTACTACTACTACTACTACTACTACCACGCGCGTTATTTGGC
GACATCTAGAGATATTATAAGCATTAACAAAAAAATGAGATAGAGAAGAAAAATGCAAA
AAATGTATGTGTATATACATCATACATATCACATCTATATATATATATATATACACACAC
ATATGTGTATATAGATGTGCGTTCGCTCGAGCGAGTTCGATATACTCGAGCGTGCGAATTGT
TCATGATACTGCCAAGCGTAGAGTGTTTCGAGTTTCTGTGTTTTTTAAGGGACAAACTATAA
TACTCCTTTTAAATGTGACGAACCACCATTTACACTCCATACACATAAGCAAGCAAGCATA
GGCACGTATGCGCGCGCGCACACGCAATGCATATATATGTATGTATATATGTACACACGTA
TTACATGTACTGAACACACACACATACACACACACACACACGCTGATTACGTAGCATAC
GTATGCGCATCGTGCGTCTCGCAATCTCTCTTGTCTGTTTGTCCGCCCAGGCATCCTGATCTG
TCCCGTTTACTCCTAGATGTTTCTTTTCGCCTACACGTATGTAGATTCGTGTGCATGTGTAAA
ATTATTCCTGATTGTGGATCGATCGAGCACGGTGACGACTGCTCGAAGAGGAAAATGATGA
TGATGTTAGAGCGAATTTTTGAACAACTATTTCTTATAAATATATATATATATATATATATA
TATATCACTTTCTCGAGAGTGATCATCTATGTGATTAAAAGTAATATTTTTAAATAACTTTGA
AAAAACCTTTCTGTTTTTTGAAGTTGTTTTGTTGTATCGTTGCTGGAATTTTTTAATTTTCACG
TATAGTTGTATATCGAAAGATAGGACGAGGAGAGTGTTAAGAAAGTTCTATATGAAAACTT
GTAAAAAAAGAAAGAAAAGAAAGAAATGTAAAAATGTAAGATTTATAAAATTTTTAAATC
TATCCAAATTTATATGAAATGTCAAAATCCATATGTGTTTTCGCATACACAGAAGAGAGAA
GAGTTGGATTAAAAAAAATTCCCTAGAAATTGATCTTTGGTAAAACTTTGAACGGTACAGT
CGGTACAAAAGCGAGCAGAGCCTCGCGTCTCCTTCAATCTCGAATACCGCTAAATGTCGGT
ATTTATAGTCGATATAGTGGTCGGTCACCGTGAATCGAGTTTTCGCACGTTGGAGCGAGATG
GAGGATGATTCAATACGCGTTCGTAATTAGCTGACACACGGTTGAGACACGGTATTTTTTCT
CATTGGAGGCAACAACAACGTTTCAGCCCTGCGAAACAGAGAGGGAGAGAAAGAGTGTG
AATGAAAGAGAGAGAGAGAGGGAATGAAAGAGCAAGAGCGAACGAGAGAGAGAGAGA
GAGAGTTTAATTTATTGGAAGCTGTGGAAAACGATGAAAAATGAAAGAAACGGAGCAAGC
TTATAAGCTGAAGATTATTATAAATAAAGTTGAATGATTATACTTTGTTATGGTAGTTTGAT
GCATTATTCTCTGAGTTTATAAATACATTGACGAATTTTCGTTCTCGCTTCTTTTTCTCGCTAA
TATATGCAATTCAATATTGTGTATAATTGTTACTTGTAACGAGAGAGAGAGAGAGAGAGAG
AGAGAGAGAGTGAGTAAGGAGCCCTGAGAATTTTAAAATTCGAAATTCTATATTTTTCATC
CTTTCTCTTCGTTGACTAACTTCCTTCGAGACCAATTTCATGAAAAAGAAAAAGATAAATTT
TGTTGAAAAATTTAGTAAAATCGTTTCGAAATCTTTCTCATCGATCGAAATAAATTTTCTCCT
CGTGAAATTGGTCCTCTTCTTGCTATCTTCGATAACGCTAAAAAATTTGGGATTTTTAACGA
CGAGAATATCGCTCAATTTTTTAAAAAATTAATCGGTGAAAATGGTATTTCTCTAGGAAGAC
TATTATTTACTTGACTTTCTTCCACTATACATGTACGTAAAATTCAAAATTTTCAAAATTTCG
TCCGAATGAAATTCTAGGGATAAAACGAAATTAATTGTGTGGTGATAAAAACTGCAATAAG
CGAGCAAGTTGTTAGTTTCGAACGATAATGAAGATTGTTTTACACTTGTCGCATGAATAATT
CCTTGTAAATAACAAAAATATACAAACGGATTTTGATTTTATTATTGCGAAAACGATTCGTG
AAAATATCGTATAATTAATCTCGACAAAATTTTCGGGGCTGCCAACTTGTATAACACCCGA
ACGTACAAAAAATTTTTGATCGCACTTATCAACTGTCGGCAAAATAGTAACGGTTGATTGCT
ATACTAATTTACTGTAAAAAGAAAAAAAAAAAAAAGAGACGAAAAAACGAAAAAAAAA
ACGAGAATAAATAACATAGTGAGATATTAGAACTGCTATCATCGCTGCTAGGCATAAATCT
CGAAGGATAATGTAAAAAAAAAAGAAAAATGATTGCAAGAGTGACATCGATACACAGTG
AAAGACAAATTGTACAAATTGTCCCAGAATTTTTAAAAGAACCGTCGTTACTTGTCACTTTG
TAGGACCTGCCCTCGTCGGATGTGCACCCAGTCGACTTACTACTACTACTACTATTACTACT

ACTATTACTACTACTACTACTACTATTTATTATTATTATTGCTCCTGTTACCGCTGTTATTATT
ATTACTAACACTCGACACTAACATTTATACTCGCATTAATGAAAATGCACGCGGCCATACA
CACACACACACACACACACACACACACACATACACACACACAAACGACACAACGT
GCGATCAATCGAGAAAGGGTATAGTTGTCGAATCGTGTACAGTTGTCTCGACTCGAGATTA
TTTATTAATCTCGAGCCTGTTTTCGCGAGAAGGAATACAAAACGCTGAAAATTAACGAAAA
TTAAAAAAGATTAATGTATGGTCGATCTGATTTATCCGATAGCTGTTCCATAATAAAATCGA
CAATAGTTAAGATTCGGTGGAGATTTGTTTAGACGAGTTTAATATTAGGCTGAAGCTAGCCA
TTTTTTTGACCGAGGTTAACGTTTACACAACGATCGATGATCCATTTGGAATGAAAATGGAT
GATATAACGATCGTACAAGTATTTACTTGCAAACTTGTGTGTAAAAAAATTGAAAATGTTA
CATAGAATATATAGTTGCGCGCATATAATTCGATTCGACGAGAAATTTTTTTCGATCGACTG
TACCGATCGAGTTACACACACATATATATATAGATTTTCAACTATATACAGAGAGTCGTCTC
CTCGCGTAGTTCGGGCATTTCCATCAACACTATTACTATTTACTTACTCGAAATACGAAGTG
TTATCGCTGGTGCGTAATCAATGTGATATTTTTATCGTCGAAGGGTGTGCGTGTGTGTGTGTG
CTCGTTCCATGCTTGGAAAAAATTGGAATCTTGATTTTCTGAAACTGCGTTATTACATCGAT
AGTAATGGCGGCGTTCGACTATATAAGTTAAGGATTATATTAACGGGCGATGAAGGCTAAT
ATATTATATTATCAGCAGCCTTTACCACCGGATAGATAAGATTATATTTCATTGTCGAATCC
GCGATAATCCGGACGACGTCGAAGCAAAAGTGTATGCTGCCACTATCAACTATAAATTAA
ATTTTACCGATACAATCTATATATATATATATATATTAGCTTTAATAACGAGGAAGCTAATA
ACGAGCACGTGCGTATTTCTTAAATTCGTAAATTCGTCTCTTGACTCGAAAGTAACGTCAAC
GTTTTACTAGTCGTAAGAGTGAGAGAGAGAGTGAGAGAGTGAGAGCGAGAGAGGGAGAG
AATGTTAATACTGAAGCACAACTATACAACTATACTGCTTGAAACTCGATCGATCGTGCAT
CAACAACACGATGTTCAAAAAAGCGTGCGCTTGTTCGATGAAAAAAAAAAAAAAAAAAA
AAAGAAGAAAAAATTTTCAAGCGTGTCTCGTATCTTCTACTATAGCAATTATGTATATATAT
ATATATACATAATCGAAATTATTGTTAAAATTATTGAGAATTCGAAGAGAAAATTGAGTCC
GGTAGGGAAGGGACGAACACTGCTAGGATTTTGACTGCTCCTTTTTTCTTTTTTTCTCTTTCC
TTTTTTTTTCTTTTTTCCCTTTTTTCTTGAGTAGACGAAGCGTAAGCGCGTCGAACGAAAAGA
AAAGAAAAAAAATGCGAGAGATTTTTCTCCGAGAGGAGAGAAAAAGAGAGGTTAGTTCGTG
GATTGTGCGGCCATATTGGATTGCTATTGTAATACGAATTGGTGCTTTTTGGAATGTATTAGA
CTGAAGAAAAAAAAAAAAAAAAAAAGAAAAAACAATTATTGTTGCACGTTTTGGTAGAAA
ACGATAAAAAAGAAAAAAATCGCATAACAAGGGAGGAAAAACATGACTTGAACAACAGA
CTTGACGGGAGTTTTTTTGCTCCGCTATTCGATTGATGTAATTGGTAAAAATGATTATTTCTA
TTTGGAACGTTTTATATATATACAAATATATTATATATTCGCTAGGTTGCACACTGATACAC
ACACACACACACATGACATGCTTACATACATATATACATACATACATACACATTTACGTGG
TATTTTGTGTACAGTGATGGGAATCAAATCGACTTTGTGATTGGAAAGTTGTTTTTTAAGAA
AGTTAATTTAAGAGTTAGTTATCACGGTTGATCGTGAGATCGATGATCCTCGTGGCCCTGGG
ATTCGTTTTGACAACGATGATATTTATCGATGGATCGAAGTTGATATAAAAAAAAAAAAAA
TTAAATTAAATTAATAATTAATCGAATTTGCAGAGTTACTCTGCGGTAACATTTGTGATTCT
AGTAGGTTAGATTTGTTAAATTCGATTCTATTTCGAAATTCTTTTTAGGGACGTATTTGTTGA
AAGAAAAAAAAAAAAGAAAATTTTTCTCGCGGCGTTGTTTCTCATTTTAAAATTTGAAATGT
TCCCGCCGAAAAACGGTTCGAACGGTTTGCGCGCGAACTTTCAAATCGCTTGGAATCGTCTT
CTGAAGTCGTCGACCTCCGTCAAAATTTTCGTCTCTTTGGCTGTAGGAATTTTTTTTAAAAGA
GACACTTGGAAAAAACAAAAAAGGGATAGAAAACGTATGTATATAAAGAGATACGCATTT
ATTACATGAGTGTCTTTGAAAATTTTTCGAGGTCGACGCGTTCGCGTAGAACTTTTTATCGC

AAAATTTTTCGCGTAGTTGCAATTTTTTCGCGTCACTCGTAGAAATTCGAGTGAACGGTGTT
GCAAAATTAACGATACTCTGATGTACTGTGTAGAGTACACCTCTTTTATTATAAATTTTTAGT
AGTCAAATTTTTATCGAGTTACGAATTTGTTCCTTCGTAAAAGAAAAAAAAAAAAAAGGAAA
AGAAAAAAAGGAATTGAAAAGAGGGGAGAAAAATAATAAAAATTTTCAAGGCAGATATTG
GTCGCGTTACGATTCCCAACACTAATCTCACACGTGTGTATACAACATAAACGCGCGCGCG
CGCGCGCGTGAAATTAAACGATGACTTTGGCAAGCGATACTTTGTATGTACAACATGTATT
AGTGGTTGTTCTGTTCGAACTGTGCATGAATGAGTGCATGGATGCGTGTATGCGCGCGTGTG
TGTAAGAGAGAGAGACTGAATCTGTGTGTGAAAAGGAAAGAAAGGGACTCGGTGTGCACT
CCGCTTAGAAATGTGAAAGAGAGAGAGAGAGAGAGAGAATAAACAAAAGAAGAAACCG
AGAAATGGTGGGAAAAAAAAAAAAGAAAAGAATTTCGTCGCGCAAGTGCAAAGAGATTTTT
TTGCGAATTGGCAATGCGGCGATGCAGTTCGACTTATTACGCCCGTGTGCGTCTTTAATAGA
AGTTACATGACGTAGATATTCGATAGGCACGCGCGAAATAATTGTCTCAGTCGATTTGATCT
TGAAAAATTGCAAATCGGATATAAACAACATTAGGATTAAACTTATTAGGAATACTTACGA
TGGAATTACAAAATCCTTTCTTTGTATAAATGTTATATAACGGAGAAAATATTGAGAAATTT
CGAATTTATATAAATTTATAAAATTCGAAAAGAAATATGTTAAGAATTTAGGATTGAAATT
ATTTTGTAATTTCATCGTTTTTTCGGTTGAAAATCCCCGAATTGGTAATTCAAAGTTGTAAAA
ATGTGAATGAAAAGCTACGTCGACTGTGAAGTGGCTCTGAGAAGTGGCTTTCTCGAATACC
TGAAATATTTTGCTGTCCAGGATATCTGAAATTCTAAGGGAAAAAGAAAAATTAAAAGAA
AATTAAAAGTAATTAATCGTTTAAAAAAAAAAAATCATTGAGAAAAATTACCTGAAATAGA
AAAATTCTTCGAAGAAAGGATGAATTTTAAACAGAGAGAGAGAGAGAGAGAGAAAAAAA
GAAAAAGAAACGAATTCGGGTATTCTCGACTAAGTGGGATTCCCTTATATTCACGCGTGTC
TAGTAATTTACGCGTTCTCCGCAATGAATTTGGTGGAGCCACATGTACATCAACAATATTAT
ATTCTAACGATTACTAATTAAATAATGTACAATAATTTTAACGATTGCTAAATGATGATGAT
GATGATGATGATGATATCGATGAAGTCTTTAAACATATATAAATAATACAGATGTTGCACG
ATGAAAATGAAGATATACGAGAGAGATAGTTAAGCGAATAGGAAGCGAGAGAATGAGAG
GGAGTGAGAGGTGATGAGAAAAATGAATCCGAATGCATAGTTGCAAGATTAAACGACAGA
GGGCTAAACAGTAAAACTGGACGAGAGAAGCTATACGAGATGATGTATCTGTCTTTTAAAT
CGATGTGTATTTTTTGCGAAGAAATAATGCGAGAAATATATTGAAGAAAGGACGAAGAAT
GTTCTTGCCAGTTTATGTTCCAGGGGGCTGACTCCTTTTTCAGGTTAGTTGGTATCCCATAAA
TTCGGTCGGTATCAGAACATATTTAATAATTCGAAAATTTATTTAAAATTTACGAAATTTGA
TTATGATTTTATATCGTGTTAATTTTTTGATGATTTATTTATTTTTTTTTTATTTTTTATTTTTTTT
TTTTAAGGTTGACAACAAAGATATCGTGCAATTTCAATTTCTCTGGAAATTCTTACGAAATC
GTTACGAAGAATCATTCATATTCTCTGTGGATAATTTAATTTTAATTTTAATTAATTAAAAAA
AGAAAATTTGTTGTAATATTTGTGAAGGCATGAAGTGAATATCGCAGAAGTTAATTTAAAG
ATTAACTTCCACCTCTTAAGCAATAGTGAATATCAAAAGTGCTCCATATAAGCAATGAACC
GAGAAAGAATGGCTGAAAACCACTGGAAACTCAAGGCTGCTAAGTTGTGCTGCGCGAATA
AAGCTGTTAGGTAAGTGCAAAAATTCAGTCGGTCTTTATTCAACAAATATCCTTTCAACGTA
CATAAGAACAATGATAAAACTTTTATAAAAAAAAAAAAAAATTATAATTGTACGAATGTAA
AAATAATTCATAACAAAAAAATTTGAACGAAAATAGAGAGAGCAGAGTTTCTGAAAAGTGA
AAAAAAAAAAAAAAATTGTTGAATGTTGTTCGAGTACAAAACGTTAATATCTAAAACGTC
AATATTGATTCTTTAAACAAAGAAAGGTAAAAGGTAAAACCGGCAGAATTCTTGCATTTAT
CCGAAATTTCGAATAGAGAGAATCTTACGAAAAATTGTGTCGAAGATCTCTACGGATCTCA
GTTTTATTCCGAATTCGAAATGTCTCAAAACGAACGATACTACGAGAAACAGTTTAAAGCA

AAGATGCGTTGTGCATTTAACTTACTATGTAAATTATAAAGTTCGATTTTTTTTTGAGACGTT
CTCGAATTCCATACGATAGGAAGGATTAAAAATGTACAAGTGTTGATCGCGTTATGAGTGA
AGGAGAAAAGTTTTTTCTTTTTTTTGCAATTTTTAATACACTGCAAGCTTGTTCTTCGATCAC
GCCGATTTTTTAACGAAATGAAAATTGTTTTCGCAGCTTGTTCGTTTTTTTTTTTACGAATTTT
ATCGCGCTTTGTTGACATGCAATACTGTGAGATGCGGGATGAGAAAAGATAAAAAAATTCT
AAAAGACAAAAAGAATTTGAGATTTCCATTAAAAAAAAAAAAAAAGAATGGAATTCGAAA
ACTGCTTTTTGTGATTGAAATTTTTTTAATTGAAAAGAAAAAAGAAACTCTCCTTTTTAATAT
TTTGCAAAACCATTCTGGAAAAGGGGTTCTTTCAGATCAGTGTGTCCCCCCCCAGTGTTAGT
CGAGTACAACACAATTGAGAAAATAGATGAGCGAAGAGGG

246. >MSTRG.5144.1 Gene=MSTRG.5144
CAATGCCAAAAGTGGAGTATGCAATATTGGGTCGTAGAAAACAAAAAGTTTCTCGTGTACG
CTTGGTTACTGTCGTGCATTGACTTGTATCAATCCAACAGGGAAATGGAGGGATCCCCCAA
ATAGTATTGACAGGATTGACTCGCGCAGGCGTTCCAATGCTAGTTGGCCTGCCACTATGCTA
CTGTTCAACAGTGGTATTCTAATCTCATCCATTCTATAATATCCATTTTTATTATTCGAATCCT
CGAATATATTTTCGTTTAATTTTACACCTATTTCATTATACGTTCATCCATGTATTCAGTATAT
ATAGTTGGTTAACCTCACTTCATTTTGAGAAACACTGCTACATATGATGCCATCTTGCCACT
ATAGTGCGATGTCGCTACGGGCTAAACGAAACTACTTTGTTCCGGAGGAACGTAATGACGA
GGGATGGAAAACGGATAGATTTTCAGGCAGTGGGTAAAAAGGAAGTTTTCCAAGCATCCC
AACGTTTACAGCGACCAGCAGCTGTTCGCCCAGCTGAGCGTTCCCCCACCAATGATTTCTCC
TCACCGCCGAATCAATGAGTTATAAGGCCCCTCGTCAAATTTTCAATCTCGACGAAGAGGA
CTCATCTTATAAACGCGTTTATAAAAATACGTGCTACATATTAAATTACGAAACATAGTTGT
TACGTCAATTGACGTTGCAAAAGCTATTCGCACGCGTCCGGGTGTGAATTCTGAGTGGCCA
AAATGAAGGTC

247. >MSTRG.5157.1 Gene=MSTRG.5157
ACAGAGGATCGTTCCGTCAGTCGGGTCTCGGCCGCCGTCAACGTCAATACGACGGCGCACG
AGCTGATCGCCTCGGCTCGGAAGCACCGAGCATCCTCGCGATGCAGTGGGCTGGCCCATAT
ACTCTGCCCGTTCTCGTCGTGTCCTGGTGAGGACGCAGCCGGAAGCGAGGCACGCGCGCAC
ACACACACACACAAACCATCGAAGAGAGAGCGGATGCTTCTCGAAAGGAGCGAGTCCTTC
CACTGTCGACAATCAGTTCCCGTTGCACACTCGCGCGAGAACAACGCCAATCAGCCAAGG
ATCTTCTCCTTCCCTCCTGAACCTGAAAGCTGAAATCAAACTTCGGATCATCCGTGCCTCGT
CGCTTTCCACCGCACGCGCGCGCGCGCACGCACGCACGAAATCTTTCCTTCCGTCTGGCAA
TTCTCTCGCGCCGGTCGAACGTGGCAACCACCGAGGCGGACCGCTCTCCTTCCCAAGAAGA
ATCAATCCGTGCGATGAACGACCGTTGGAAAAGTTTGAGGTTTCGTGATGGGATCGGGATC
CGTCGGAGGCTGTTCAGTTGAAAAGATTTATCGGGCAGGGAGAGGGGGGAGACCGATATA
TAGCCAGCGGGTGAGATCGAGTTTGTGGAAAATCGATGGAGAGAGAGAGAGAAGGAGAGGC
GATTAAAAGGAGCGGTGAAAAGGATGTAGTAGTTGTAGTCAGCAGTCGTAGTAGCAGTCG
AAGGAAATCGATATAGTTTCCAGCACGTGGCTGGCTTTAAACTGGCGGTTTAGTTTACGCCT
ATATACACTGATAACCTCGCTTAAAAGAAAAATAAAGAAGAAGAAGAAGGAGGAGGAGG
AATAATTCGTGCGAGTGTCGTTTGATCGAGTGAATCTCTCGAAAAAATCTTCTTTTCCCTTTAC
GACGTTCTTTTTTCTTTTTCTTTTCTTTTTTCTTTTTTCATGTTAACTGGGAGCACAAGATAGAG
AAACATTAGTAAGGAAGTAATATAGCAGAAGGTGAAAATCGTGGTGTGCCGATCGTGTTCT

CTCTCGACAATCCCCAAGCCAGGGATCTCTCCTCTCTCGGGAAGATCAGTGAAAAAACAGT
GTTGAAAATTGTGAAGTGCAAGCTTGTTAATCGGAGAAAGATCGAGAAAGTGAGAAAGAA
GAAGGAAGATCTTTGAAGAATTGTTTCGAGGAGTCTTAGGAGTCTGGCGCCTTCATCGAGA
TCCCAAACAGGGATCATTGAGAATTTTGTACGCGAATTGAGAGCAAAGGCCTATATTATAT
CAAATTTTCATCGTCCATCCTTCATACTTTCTCGCCTTAATCATCGGTTCGATAGAAAAACCG
AATATTTCGAAAGAAGCTTGGTCGTCGAGGAGCATAGGCGGGATAAGAAATCGATGTGAT
AAGGGAAGAGGAGGAAGAGTTTGAAAATAATTCGATTAACTTCGAATCCGGCAACCCTCT
TTATCGAAGTATCGAGTTGAAAGGGTTGAAAGGGAGGGATTCTCGCGGCTTATCTCGCTCT
AATCCGAAAGGAAACTTTTCAAATTATCCTAGTCGAATAAACTGGGACTGAGAATACCTGA
ACGGTATTCAAAAAAACCATCATCGTGAAAAGGAAGCAACTGGAACGCTGTAACGTAAAT
TAAATAATTGTTCGAGACAATCCAAAGATATCCTCGAATCGAGGCCAGATCTAAAAACTAA
AAATTAAACTAAAATAGGATCATTTCTATAGACGTATTTAATAGAATAGATGTTGGATCTCG
ACTTTCAAGGGGCTCAACAACTTCTAGACTACTTTTTCCAACCAAAAGCCATCCATCCTCGA
ATTTCTAGCAAAGCTATTTCAAACGATACACTTTTATAAATCCTAGACAAGGAATTCTCAAA
GAATTCGACCATCAAGCTGGACCAGGTCGACCAACTCCTCTTGGTAAATTTTTTTAAAGGCC
AAATCCGACTCAGCCTATTAATCGTCACTTGGCAACTCGTGGGATCTTAGTTTCTCAGGCCT
CTGAAACTACTCACTCCATTACTAAAGCTTTAAAAAAA

248. >MSTRG.5224.1 Gene=MSTRG.5224
GAAAATACTGACGTACTATGTAGTACTACGAAATGGCAAGTAGATGGTATCACATTTTCGT
GTCAAGCAATCCAGTCTCAATTATTCTCACTCTATCGTATTACGTTTAGGAGATGGACCCCA
TAAAACAAATATTGCAATAGCTATTGCAAATCCTATACTGGCAAACACAGCGACTTTTTCT
ACATCCATATTTTCGAAAAGTTCGTTGAAATCTGAAATTTTCTTGAAAAACTGGGAAAGCTA
ACCTACGCATTAATTTCATG

249. >MSTRG.5232.1 Gene=MSTRG.5232
GCAGGTCTTAATCTTATCGCTGCGGTTCCACAGCCACCGGTTTGAATACCTTTGCTTAAAAG
TTTCTTAATTATCGCATCTCGTAATTCAGGTGAAGCGCAATTAAACGCAATGAACGTTCCAC
GTCCACGTACGGAATTAATCATAGTAGAAAATTCATTTTGCAAATTCATCAATTTTTTTAAT
ATGTAATCTCCAACAGTTGTAACCCTATGTAAAAGATTTTCCTTTCTTATCGTCTCTAATACC
GCTTCAAGTAATATTATTTTACTTGGATCTCCCATCCAAGTGTTAAAAACACGGAACGATTG
TTTAGGCCTGTAAAATTACATCGATTACATATATTTAGCGAAAATAGAGGCCATAAATTTTT
TTTTAAACGATATTTTTAATAATAGGAAAGAAATTTTATACTTGAAAGATTCGATGTGATAA
TAACCACCCAATTGCATCTTTTTACTAAAGGTTACCAAATCTGGAGGAAATTCTAGATTAAA
ATGTTCGTGACACCACATTTTTCCTGTTGGACCACCACCTGTTTGTACCTCATCGATTAACAA
TGCTGCACCATGTTTCTTCGTTATACGTTGTAATTCTTGAAAAAATTCAGGAGATGCGTGATT
ATCACCCCCTTCTGCTTGAATAGGTTCAACTATCACACCAGCAACTGGAATTTTCTTCTCGG
TTTTATATTTCTCAAATAGTTCCTCTACATGGGCCAAACATCGTTTATCTTCTTGTTGATTTTC
TCTAATATTTTCATCCAATGGATATTTGTACGCGGGAAACGATGCTGTAGGCCAATCAAAA
GCTGGGATATCTATCTTGTGAATGTATTTACTGTGCGTCGTCGAAAGGGATCCCAATGTTCG
TCCATGAAATGCGCCTTTAAACGAAAGTATCGAAAATTGCGGTGTGCCAGGTAGTTGATTC
ATCATACAAGTTTCTATTTCTTCCTTCGTGAACGAATTACCCTTCCTTTGTTTCTCGGCGTACC
AAATAAATATAGTTTTAAACGCGTTTTCATTCGAGCAAGAACCACACATCATAGTTGTGAC

ATGAGACAATCCTGGTGGAGCAATCTCTTTATGCAGCAAAATTTTTTTCAGTTTATCTGGCC
ACTCTTTTCCAGGGAAGATGCCTAATGCAGGCCTATTTGCTATAATTTTCTGATTAACCGGA
TCAGCGAGAGTTTTTAACATAGCTGGATGATTGTAACCCAGAGGCATGGATGAAATTTGCA
TATAAACGTCGAGCAAAATATTCCCATCAACGTCCATTAAATAGTTACCTACTGATTTTTCG
TAGTCTGCGAAAAATTGTACGGAAGAGGCTTGTTGTATCGCGTTAAGTTCTTGAAACAAAC
TACGTGATCGAGGTCCTGGTATTTCTGTCGCGATCACTGGCATATTTGGTTCTTCCGATAAA
GTTACGGTTAGACATCTCTTCCAAACAGTCGCATGCCCTTGGAACAATTGCCAACGTGAAA
ACGTTCGAGCACAAAGCGCAGTTTGACACTTGTTGGCGAGCATCTTCTACGTTCGTTCGTAA
CACTAGCCTAGAGTAATTGACTGATTTTCACGAGGAATCGACAATCGAATTTTGCCGATCGT
GCGATTCGTGCGACGTAAAACGTCCTCGCGAAGGAATGGATTCGCACTGGTCATCGATATA
ACACAGTAGTGGGTGATCCTTGCGCAGGAAATATTTGATCGTTTCCGAGTCTGTACGAACAT
TCACGAATCAGTTCCAGTTTTAACCGCGAATTTTTAACCAAACGTCGCGCGGTGTAACGAC
CGACCGTTTCGTTCGATTCGCAAATTTTTTACGAGTTCGAAGAAACTCGATTAATCGAAAAG
TATAACGAAGCATACGCACGGATACGAACTCGACTGACTTCTCATCCGTGTGCGAATACGT
GTAACTACCGATAAATATCCT

250. >MSTRG.5269.2 Gene=MSTRG.5269
AAGAATGTTATATAAATTATGCATTTATCTTTATAATATGTTCTTTTAAAAAATTGATGCATT
TAAAAAGATACTTTATAATGTATCTATATAAGTTATAATAATTTAACTTTCTGTTTATTGAAA
GTTATTACATAATAAATTCAAAATATATATGGAAGTTGTGAAATCTCAACAAAAGGATCTA
TACCTGTTCTCATTCATCATGGACTACTATCAAGTTCAGCTGATTGGGTATTATTGGGTCCAA
AGAAAGCTCTAGCATATATTTTATGTGACAACAATTATGATGTTTGGTTAGGAAATGCAAG
AGGCAATGCATATTCTAGAAAACATAAACAATATACAACTAAAGACAAAGCATTTTGGAA
TTTTAGTTGGCATGAAATTGGTTATTATGATTTACCGGCAATTATAGATTATATTTTAGAGCA
TACTGGATATAGGAAGCTCTATTATATAGGTTACAGTCAAGGTACAACTGCATTTTATGTAA
TGGCTAGTGAAAAATCTGAATACAATCAAAAAATTAAAGGAATGGTCAGTTTGGCACCAA
TAGCATTTCTCTCAAACCAGAGAAGTCCTTTATTTAAATTTATTGTTCATTTCTATGGATTAA
TGGAGTGGGGATCCTCTTATTGCAATGTACATCAATGGTTCCCCCGAAATAGGTTACAAGC
ACAAACTTTGGGTACAATAATTCGAAATGCTCCAGGCAGTCTTACTAAAGGTTTTTGTGTAT
GCTGGTTTTCTTTAATTGCTGGATTTGGTAGTGATCAGTTAGACAAATCTATGTTACCATTAA
TTTTGGGACATTTTCCAGCTGGAGCTTCTGCAAAACAAATTATTCATTATAGTCAGAGTATA
TTATCAGGATCATTTCGTAAATTCAATTATGGAGCAACAGAAAACTTAAAAATTTATGGAT
CAACACAACCACCAAAATATGATCTTGAGAAAGTAAAAACACCAATTGTAATATTTTATAG
TGAAAATGATTTTCTTACTGATCCTACAGATGTAAAAAAACTTATTGATAGACTGCCAAATA
TTATAGAGACAAAAAAAATTGAATATGCAAAATTTAATCATATTGATTATTTGTGGGGTAG
AGATGC

251. >MSTRG.5279.1 Gene=MSTRG.5279
CGTTAGGATCAATTGTGGACGTAATGCGAGGCAGAACGGTGCAGAGTAACGCAGCGTGAC
GAAGCGCGAGGCAGCTTACGCCGACGCAGCTTCCCACACTGGCGTGTCTCGAACGGCGCCT
TCGACCAGCTTCCGATGTAACAGGTTCATGTAGCAGAAGCTGGAGAATTTCTGAGAGTCTA
AAAGACATGTAATACGATGGCAGACGAGGAGTTAATGCGCCGAGTCAAACGAACTTCCGG
GTCTTCGGCGACCCTCGTTTGTCTCGGAAGCCAAAGAAGTCACAAGGAGCCGTTACGTAGT

GGCATCGAAGCATTAAGGCATCAAGGCGTTCGAGTCTCTCGATTGCCGCGTCTTTCCCATCA
CCAGGGA

252. >MSTRG.5299.7 Gene=MSTRG.5299
TAAGTTCCAGTTGTTTTTATATATATATTACAATTTTCAAGAACTATGGTAAGTAGAACGAA
CTGTTGCTCGACGAGCCACCCCTCCATCCCGATTTCCAGTACTCGTGTGAGAAATTATCGGA
TTTTAATTACCATCCGTACGAATACGAGGATTGAAGTTGGCAGCACTGAATCCACATCTCTT
TCGCGTGTTCTTGCGGTGCGCGAGTCAATATCAACGGTAAAAATTCTTTGTTAAAACGTTGA
AGAAGAATTGTTCAGAATTCATGCAGCGGAACGAGTATTCCCGGCAGAATCTGAAGTATTC
ATCGGGATGATAGAATTTTTACTGAGCTTTGAAACATCGGGAGTTACATCGGTGGAAGCTTT
TAATTTCTGCATTGAAACATACGTTAAAAACATACGTTCTTGTCACAATTGAAAGAAGAAA
ATTTTCATCGAGCAAAAGATCTATATGGTTAAATTCCCGAAACAGAGGAGAAATTACAGAT
CTGTTAAACGTTTACGAATGATTTCAATCGAAACTTCACAAAGGTTAGTTCAGTTATTACCG
AAATTGTGCCAAGTGCAAACATTATCTCTTGACCCTCGAAAGCTTCGCGCTAATTGGCAAC
ATCCAGGGGATGAATGCTACCCCCCTTCCCCCTCCATCTCCCCCTCCCCTTCTCTGATCAGA
CTCTGCCTACAGTGGGGTTTCGAGGATAATAATCGAGAGGCGCACAAACTGCACTGGACA
GCTCGTGTACTCGCCGCCCTTTTCTCTATGAGGCTCTGTGTTTGCACCAGCCACGTATTTCCC
ATGTGACCCGTGTGCATTTCGTGTATAGGTCACCCTACAAATCGTCGATCGTGTTACTCGT

253. >MSTRG.5329.1 Gene=MSTRG.5329
CTCTTTCATAATGGTTGTAATGTTCATGTAGTTTTTAAAATCATATTTTTATGACACAATTTCT
TCTGTGTTTAATCTTTTAGGTATTTAGTCATTATATAGTCATATTTATACTACTAGATATTTTA
TTCTTCATCATTTTATATCTGTTTTAATTGTTTTTATTATTTCATAGTTGTGTTAGGTATTTCAA
CGTTATTTTTTTTTTTTTTTATTTAATGAATTTCTGAAGATATTTTATAATTGATAGTCACATCA
CATTTTTTTTTTTTTTTTTGTTTTTTTTTTTTTTTTTTTTTTTTTTTTTTTTTTTTTTTTTTTTTTTTTTTTTTT
TTTTTTTTTTTTTTTTTTTTTTTTTTTTTTTTTTTTTTTTTTTTTTTTTTTTTTTTTTTTTTTTTTTTTTTTTTTTTT
TTTTTTTTTTTTTTTTTTTTTTTTTTTTTTTTTTTTTTTTTTTTTTTTTTTTTTTTTTTTTTTTTTTTTTTTTTTTTT
TTTTTTTTTTTTTTTTTTTTTTTTTTTTTTTTTTTTTTTTTTTTTTTTTTTTTTTTTTTTTTTTTTTTTTTTTTTTTT
TTTTTTTTTTTTTTTTTTTTTTTTTTTTTTTTTTTTTTTTTTTTTTTTTTTTTTTTTTTTTTTTTCTTTTTTTT
CTTCTTTTT

254. >MSTRG.5329.2 Gene=MSTRG.5329
TTATTTTTTTTTTTTTTTGTTTTTTTTTTTTTTTTTTTTTTTTTTTTTTTTTTTTTTTTTTTTTTTTTTTTTTTT
TTTTTTTTTTTTTTTTTTTTTTTTTTTTTTTTTTTTTTTTTTTTTTTTTTTTTTTTTTTTTTTTTTTTTTTTTTTTTT
TTTTTTTTTTTTTTTTTTTTTTTTTTTTTTTTTTTTTTTTTTTTTTTTTTTTTTTTTTTTTTTTTTTTTTTTTTTTTT
TTTTTTTTTTTTTTTTTTTTTTTTTTTTTTTTTTTTTTTTTTTTTTTTTTTTTTTTTTTTTTTTTTTTTTTTTTTTTT
TTTTTTTTTTTTTTTTTTTTTTTTTTTTTTTTTTTTTTTTTTTTTTTTTTTTTTTTTTTTTTTTCTTTTTTTC
TT

255. >MSTRG.5330.1 Gene=MSTRG.5330
AAAAAGAAGAAAAAAAAAGAAAAAAAAAAAAAAAAAAAAAAAAAAAAAAAAAAAAAAAAAAAAAAA
AAAAAAAAAAAAAAAAAAAAAAAAAAAAAAAAAAAAAAAAAAAAAAAAAAAAAAAAAAAAAAAAAAAA
AAAAAAAAAAAAAAAAAAAAAAAAAAAAAAAAAAAAAAAAAAAAAAAAAAAAAAAAAAAAAAAAAAAA

AAAAAAAAAAAAAAAAAAAAAAAAAAAAAAAAAAAAAAAAAAAAAAAAAAAAAAAAAAAA
AAAAAAAAAAAAAAAAAAAAAAAAAAAAAAAAAAAAAAAAAAAAAAAAAAAAAAAAAAAA
AAAAAAAAAAAAAAAAAAAAAAAAAAAAAAAAAAAAAAAAAAAAAAAAAAAAAAAAAAAA
AAAAAAAAAAACATAAAAAAAAAAAAAAAAAAA

256. >MSTRG.5337.1 Gene=MSTRG.5337
CGCGACCCGCGAGCGCCTAGTCTCCGTCTGGCCGAGCCTCGATTCGCACGAGTTTGGGGGG
GCCGTCGATCTCCTTGCGCCCCGTCGATTATCAAATTTCTATTAGCGAAGGGGAGAATTATT
CGTTGGGGGGTCGTCGTGCAAGTTGGCAAGTTGGCAAAAGGCGGCTCTGACAGCTGCAGGT
AGCTGGATAGAACAAAAGATCGGGCGCGAACGGAGGAGGAGGGAGAATAAACGAGGAG
ACAATCCACGGAATAGCGAACAATTTATTAACCAAGAATGCGTTTTCTACTTATGATCACA
AGCTTTAGCTTTCTTTGGATTTTGTCCATCCATACCTATCCAACAAATGGTATGAATATGATC
TTGAATAGAGATTCCTTATTCTTTCCCGACCAAGCAGAATATGTAAAGCTCGCCATCAAATG
TCCTGAGAACATGATCCTATGGCCAGGAACTCGTCGATGTTACAGAGAGGGTGAACAAGGT
CCCTGCAATATCGATAAAGTTTTAACCTTTGACCGGAAACTGTGGAGACCTGTTTGTTCATA
TGCGGTATTAAGGGAATAAGTAGCAAGATTCATCGTAAAATAAAGTGTATTACAGATAACT
GTTGTTACCAACTGGACAGAATTCTTCAAAAGATGGCAGCATATATCCGGATGTCTTCCATG
AGATATTTTGAAAGATATATCTTAATCTAAGGATATAAACTTTTCATTATAATTTTATATTAT
AAAATACAAATAATATTTAAG

257. >MSTRG.5354.1 Gene=MSTRG.5354
CCCCCTTATACATAAAGTGCAGCAATGCCTTCATTTCCGTTTCATTGACATCCTTCAGGAAT
ATAATTGGATGGGGATGGGTATTCTGAAGGAAAATGCTCTCAAAATATGGACTGCATGCAG
AGAGTATCGTCTGGTGTGCCTTGAAAGTTTCTCCAACACACGCCAGGGTTACGTCGCAGAG
TGCCTCTCTGGCAAGTAAGGAGCTGAGAACGTCTGTGAGGTTGGCTGGATGGTTGTTCCACC
GTAGGCAATACTGTTGGTCCATCTTGTCTATCTCCAATCCCGACCCTTTCGCAAACTTTTTGC
CATTACAACGCCACCCGACCCTTAACGTCCAC

258. >MSTRG.5355.2 Gene=MSTRG.5355
GGGGGATGTGTCCGTGCAGCGTACACGTCGACCTGCATCCACGCCACCCTCTCTCTCTCTCG
GCGCCACCCCTGGCCTCAGTCGCGCTCCGCGGCCAACTTGATATATACGGAAAGTTATTCC
CGAATCGACGTAACTGTGCCCGTTTCTCGTCTCTCGTCTGAAACGAAAGAAAAGAAAGAGA
AATAATAAAGAGAAAAAAGGGGAAAAGAACTGAAAAAAAAAAAAAAAAAAACTCTTCTCT
CACCGTTTCATCGCGCGCCTGATCGTCATGACCGGCGAGACAAGTTTAAGACGCTGCATAC
TCGGCTCGGTGCGAACGATGTAATCAATCAAGTGCCGTTTTATTGCTGCCTGTTGTTGTACT
GTAACTGTCATGTGCGCGGATGTACTGTCTACGACAGAGATCGCAGTTACGTGAACGTGAA
GAGGATATCTCTTGGCCTGTTGTCCAAGGGGAGAAGAATTCGTCTTCGTAAGATCGCCGCG
GAAGATTTTTCCTCGTCGTGAGGGGGAGAACTGATCCTCCTTCCGTGCTGCGTGGTGCGGCT
ACGACAGGCCGACAGGATTTCATGTTCACGAATTTCAGTAAACCAGGTAACGATGCGCCA
AACACGCCTGCATCACAGGCAATCGGCGCGTCGATTTTCAGATTTTCATAACGCCCTCGCGT
CGTCGTCGTCGTCGTCGTCGTCGCCGTCGTCGTCGTTGCCACTGTCGCGAGGACAACGC
GCGTTCGATTTGCGCGCTCGGCCTTCCTTCCGCGGCCTGGCTGCACGCGGCTGCTGTGCACG
CTTAGAGGGGAGGATACGCGCCTATTCTGCAGCTCGCAAAACGGCCTACCAGACCTGCCG

GACCCATCGATCGCGAGATCTAGAGGCATGCGTCGCGTGTTCCTCCCTCTCCCTCTCTCTCT
CTTGCTCTCTCTACCTTCAAGGGAACTTGAAGAATAGGCCGAGAAACCACCACCGAACGTG
AGACGGATGTATTATACTAGGACGAGCGTTGAAAACGCGCTTCTTCGATAAAACGATGCAC
CGCTCGGTTTTTTTCGTCCAATTTCGGTTCGTTTTTTTTTTTTGTTACGCGAGCTGATAATCGG
ATAGGAAAATAAGGGAAAAGAGGATTGATGGTCGGAAAATATGTCGGAATTTCGGCACGC
AGGGGATTCTATACGGATTTCAATAATATACGCTGTAAACTAGACTGGCGTTTATTGGTTTG
ACCCATCCGATTGAAACGTAAACTAATCCTGGCGCGAACAAAAATAGGGGGTGGAAGGT
GGGGAAAGGGTTGTCAATTTTCGAGGACATATATTCGTGGGCGAAATACGCGCGCAATTTC
TGACCGCCGCAATCGTGCAGCGGTTCCATTGTTTCAGATGGTAAAATGAGATAAATCATAT
TCTTTCACACCCTAAATGGGGGATCCGTGAAATGAACGAGGTAGATTTATCGTAGGTCGAA
ATTTCCACGCGCAAAGACTGACTCTTGCTCAATTAAATGATAGAGGACCCGTGTATATAAA
ATATCCATATATTTTTTTATATATATTGGTATGATTAAAATTTAGA

259. >MSTRG.5355.1 Gene=MSTRG.5355
CGGCGAACATAAAATCCCTCGCACATGCCTGGTGCATTCATTAGGGGGGATGTGTCCGTGC
AGCGTACACGTCGACCTGCATCCACGCCACCCTCTCTCTCTCTCGGCGCCACCCCTGGCCTC
AGTCGCGCTCCGCGGCCAACTTGGTACACACGCCACTTTGTTGTTTCTCCCTCTTCGAGTTTC
GTCCGCATTTATATATACGGAAAGTTATTCCCGAATCGACGTAACTGTGCCCGTTTCTCGTC
TCTCGTCTGAAACGAAAGAAAAGAAAGAGAAATAATAAAGAGAAAAAAGGGGAAAAGA
ACTGAAAAAAAAAAAAAAAAAACTCTTCTCTCACCGTTTCATCGCGCGCCTGATCGTCATG
ACCGGCGAGACAAGTTTAAGACGCTGCATACTCGGCTCGGTGCGAACGATGTAATCAATCA
AGTGCCGTTTTATTGCTGCCTGTTGTTGTACTGTAACTGTCATGTGCGCGGATGTACTGTCTA
CGACAGAGATCGCAGTTACGTGAACGTGAAGAGGATATCTCTTGGCCTGTTGTCCAAGGGG
AGAAGAATTCGTCTTCGTAAGATCGCCGCGGAAGATTTTTCCTCGTCGTGAGGGGGAGAAC
TGATCCTCCTTCCGTGCTGCGTGGTGCGGCTACGACAGGCCGACAGGATTTCATGTTCACGA
ATTTCAGTAAACCAGGTAACGATGCGCCAAACACGCCTGCATCACAGGCAATCGGCGCGT
CGATTTTCAGATTTTCATAACGCCCTCGCGTCGTCGTCGTCGTCGTCGTCGTCGTCGCCGTCG
TCGTCGTTGCCACTGTCGCGAGGACAACGCGCGTTCGATTTGCGCGCTCGGCCTTCCTTCCG
CGGCCTGGCTGCACGCGGCTGCTGTGCACGCTTAGAGGGGAGGATACGCGCCTATTCTGCA
GCTCGCAAAACGGCCTACCAGACCTGCCGGACCCATCGATCGCGAGATCTAGAGGCATGC
GTCGCGTGTTCCTCCCTCTCCCTCTCTCTCTCTTGCTCTCTCTACCTTCAAGGGAACTTGAAG
AATAGGCCGAGAAACCACCACCGAACGTGAGACGGATGTATTATACTAGGACGAGCGTTG
AAAACGCGCTTCTTCGATAAAACGATGCACCGCTCGGTTTTTTTCGTCCAATTTCGGTTCGTT
TTTTTTTTTTGTTACGCGAGCTGATAATCGGATAGGAAAATAAGGGAAAAGAGGATTGATG
GTCGGAAAATATGTCGGAATTTCGGCACGCAGGGGATTCTATACGGATTTCAATAATATAC
GCTGTAAACTAGACTGGCGTTTATTGGTTTGACCCATCCGATTGAAACGTAAACTAATCCTG
GCGCGAACAAAAATAGGGGGTGGAAGGTGGGGAAAGGGTTGTCAATTTTCGAGGACATA
TATTCGTGGGCGAAATACGCGCGCAATTTCTGACCGCCGCAATCGTGCAGCGGTTCCATTGT
TTCAGATGGTAAAATGAGATAAATCATATTCTTTCACACCCTAAATGGGGGATCCGTGAAA
TGAACGAGGTAGATTTATCGTAGGTCGAAATTTCCACGCGCAAAGACTGACTCTTGCTCAA
TTAAATGATAGAGGACCCGTGTATATAAAATATCCATATATTTTTTTATATATATTGGTATG
ATTAAAATTTAGA

260. >MSTRG.5409.1 Gene=MSTRG.5409
TCTACATTATGGCGGCGTCTCAAGGAAATAAGATATACAGTTTTATGGAAAAAGAACTAAA
CTTCATAATGAAGTCGATATTTGTAATTTTGAAAACTAATATGGTTCATCGCGTATATCCAA
AATATAATAAATTATAAACAATTTTTAAATAATTTAAACTTCAAACTGTGCATTTTAATCAA
TTTACTGCAGTGAATATGAATTTTCGATAGTGTGTCATTGACTCAAAACGAAGTATCAAAAT
CGACTTAATTTTGTGCTTTATAAAACATTAAGACAATTACTAAAATTTAATTTATTAAAAAT
CATATGATTGTTTAAAATTAAAGTGAATAGTATATTTGTTAGCCAATTATCGTTATTAAACA
TATATTTTATTGCTAGTGTGATTTCCACGGAAATGAAAAGATGTATATGACGCCAATGCTTT
GTTTTCTTTTTGTCGAATACTTTCCGTTGTTTTTAATGCATATTGAAGTTAAATATTAAACGT
CATGGCACAAAAAACTTTCTATGTAATTAACTATTAATTTACGCTTAAAATGATACTAATGG
AAAATTATTATCGAACACTCGTTAAGATGTGTTTGTTTAATGTTGTTTATCACTGATCACATC
TTTGCTGAGGTCAGAGAATGATGCCGGTTCATCAGATCAATGTTAATCCGCCTGACCGGCA
ACCACCTCTGTCGCTTGGTTCTTCCAATAACAATAGCCCTGCTGGTGGTGAAGGTTTACCAG
AATGGACATCAAAAGAATCAACATATCCAACAGTAGTTACAATCATACCAGATCATTATCA
TTCATCAGTAAGCACATTGTCTTCAAGCAATGATATTTGCAGTGGCAGTTTACGCTATGTAC
ATCAAGCTTGCCTTCAACAATGGATAAAAGCTTCAGACACACGTGCTTGTGAATTATGTAA
ATTCACATTTATTATGCATGCTAAGACAAAACCATTTTGTGAGGTATTATTTATATTTTTATA
TATTTTCTATATATATT

261. >MSTRG.5461.2 Gene=MSTRG.5461
AAAAAAATTTCTGATTTGAATTTATTTAGTAATCGATAATAATTTAATAAAGTTACTGTATC
GAATACAATTTCTATACAATACATAACAATACGTATTTCTAGGAACTGTTTATTTTGTAATA
TATTTTTACAGTAAAAATGACGCTAAAACATGTAGTTATAGGCAAGTTTTTTTCTCATTCGA
CAAACTTAAGATAAATTTTCGAAATAATATTGGCTAATAGGTTATATTACGGATGAGGTTAT
GTAATTGTCTCAGGTGGTGGTACAGGTTTTATCGGAACACAAATAATAAATTCATTGTCTCA
TAAAGGTGCTTCTTATACATGCATTTCTCGCATGCCTGGGCCTAATCGAATTTCATGGCATG
ATTTGGAATGTTATGGTTTACCTGAAAATACTACTGCTGTAATCAATGTTGCTGGTCAGAAT
ATACTTGATCCAAAACAGCGATGGTCAGAAGGATTTAAACAAAATGTTATAAATAGCAGA
G

262. >MSTRG.5474.4 Gene=MSTRG.5474
TTTAAATAAATTTTTCAGTTTGCGCATGGCAACTGATTTGGCGCGTAAATACTATATTACAA
AATTTCAATCTCCTTTTGAATTTATCGCGCATGCGCGCACCACTGTCGGCACGCGTTCCTTCC
ACGAGTCGGTCAGTGCGCGTCGAACGAGGAGGAAGGAGGTATCGTATCGGCTTGTCGTCCT
TCTTTCACGAATATTTTCGGTATTTCTTTCGAATTATAATTTTTTAATTACCGAAATCATCGTG
GCGCTATCCGCCTCGAATTGTCTAACGTGCTTGTCAAGCCGAGTCACGATTACGAAGTCATT
TATTCGCGAATGTACAAACGTGAGTGTCATATCCCGCGTATTTGTCGCGTCAGTTCGTGAGT
GTATCGCGAGTGTTCGCCACGGGAATAATTATGAGTGCTATGCTACCGATATTATGTGTTAT
CCTGACCCTTAGCGTCACAACGACTACGGTGCGCGCCGGATTGAAATGTGACGCTGTCAGA
TCGTACTTCGAGTCGCAAGGTTTTCCGGCCACTGACATTCCCAAGGAGGCGATTTCATGTGA
GTATCTTATGCGTGCATCCTCGATACGCGCGGAGATAAAGATCAACGATTCAGTTATCGTCC
ACCTTCGAAGAAGAGGAAGAAAAGAAAATGGAGGAGGATGAAGCTATACGATGCTAGGA
GAAAATTGTCGATCAAAATTGTTGTTATTGTTGTTTTTATTATTATTATTATTATTACTTTCTTT

TAAAAAAAATTTTCCCTCTTGAAACGATCGGGAAACAGATGGTGTTTCGTGCGACGAAATA
ACACGAATGTTAATCAAGTGGAATTTTTGCCGATTAAAATTACAATTTTCATTACGAGTGGT
CCTCGCGAAACGGGATAAAACGGGCCTCCTCGTGTTCCCTCGCAGAGAACCAGTTTCGATC
GTCGATTCGTCGCTTTGTTAACCGCTTCCCCTTCTTTTTTTTTTTTTTTTTTAATGCTTTTTATCC
CTCCTCTCCTCTTCCGTGGATCCACGAGAAGTCCAAAAGAAGGAGGAAAGCGTTTCACACG
ATTCGCTGATTGAAAGTGCATATTTGTACAGGTGCAAATATCAACGCGAACAAATTTGATG
CAGATGGAAGATTATTTACGCACCCCGATATCGATATCGATGCCTTTGATAAATTCGATCGA
TTTAATTATTGCCAAACGTCCCCTAATTGGGCTCGTTTAAGAATTTACGTTTTCAACTAGTTT
GATGAAAGTTGAAGAAGAATATTTTCGTCCCTAGATTTCTAGGGATCACTAGGAACTTTGG
GAGTTATATCTGTACTCGAAAATGGAATGATTAAAAGAAGAGAGAAATTTCAAATTTAAAT
TTAAATATTCTCGAATAGTTGATAAGAATAATTTCCCTTTTACAGATATTGCGATCGAAGTTT
AACTATAATTAACCACGTATGGCGATACGGAGGAAAGATATATACGCGAATGCGTGTTTGA
AATTGCTCCGTCGAAACGAGGCTGAAACCTTTTCTTCGTTGTGAAACCTTGTGCACCTTTTA
GTTATTTTCATCTCGTTATAGACGCATAATCTTAGATATTTTTCTTCCACATTTTTTTTCTTCCT
GCAAGTAATTGCAATCGACCGCAGGTATGATGCAAGCTTTAATTGCAAGTATTTCGTTGCCT
CCCTCGATATTATACGCGGTTCGAATAATTTTCTTTCTTTCTTTCTTTCTTTTTTTTTAAATTAA
GAAAACCTATCTCCAAAGCAAGTTTCCGACAAGCGAAAGAAGGAGGAAAAAATATCACGT
TGACTAAACCGCAAAAATATGTCGTCTCGGGGAAAAAGGGAAGGATGGAAAAAAAAATGA
AGAGATGCCACCGTGAGTTATTTGACGACAGTGTAGACACCGGTGGTGGCGCACGCAAGTC
GAATGGAAAGAGCGTGGTGTTGGTGCGTGCACGTGAAA
CATCGGCCCCTTTGATATGGCAAACGAGACGCGTGGGTTGCGCGCTCGTCGACCGAAGAAT
GCACCTTGTTTGCCCAGCCTTCAGATTGCGCGGGTCAAGGCGCACGATGTCCGAGAAACAG
AGAAGAAAAGAGAAGAGAAACACGATTACACCTGATAGGAGGAGGAGATATGAAAGAA
GCAGGAGGAGGGGAAGGAAGATGGCTGAAGGACAGGCTCGACGATTTTCTACGCAGACA
CCCTCGAAACACCGGGATCGGGGAACACGAGAACTCGTGCAACCAATTCTCCCCCGCGCT
GGCACACGAGCCGCATAGGGTAATGCAATATAACATGTGGAAGAGGGCGTCTCCTAGCAA
CCCTATGTCTTCTCACCCCATCCCCTAAGCTTCGCCCTAAAACTCCCTAAATCCGTCGGTTG
CACGCCGACACCGGCCGTTGAGAAACTTTCGC

263. >MSTRG.5486.2 Gene=MSTRG.5486
AGTTTGCCTAGTGGCCATTTTGTTTTCAATCGCACGGCTGACTAAGCAGCATCAATATGGTT
AAATATACAGAACTTGGAAATGCGACATCAATAACTCCCTCCATCGAATTGAAACCAAAC
ACAGCACCATATGATCCAAGATTTCCAAATCAAAACCAGACAAGATATTGTTATACTAGTT
TTTTGGATTTTCATCGTTGCAAAAAACGACATAGTGAAGATTATGAAGCATGTCAATATTTC
AAAAAAGTATATAATGCAATGTGCCCAAATGCTTGGATAGAAAAATGGAATGAACAGATT
GAAAACGGAGTGTTTCCAGGAAATATATAATACAAGAGGCATCAAATTATAACTGCCTCTA
TAGTGATATTGTTTACATAATAACAGGA

264. >MSTRG.5503.1 Gene=MSTRG.5503
GAGAGAGAGAGAGAGAGAGAGAGAGAGCGAGAGAGAGAGAGAGAGAGTGAAATTCGCGTG
GAAAGAAAGGAGAAACGATGTTTCTTCTGGTTGGGCGAAAAATTGTTCCTTTATTTCTTTCA
ACGTGTTTCATTAGTGTATAAGAAGAAAGGAAACGATTAGTCGATAACGAGTAATAGAAA

GTTGTTCCACTTCTTGTTCGCAACAATATTCGTGCATTTTTATTTTTATATTTTATTGGTGCTTC
CGGG

265. >MSTRG.5505.4 Gene=MSTRG.5505
CGAATATATATATATGTGTGTATACGTGTATACGTATACGATGCGCACAACACGGAGTTATC
AATAATTCATGGGAGCCATGATCGGTGACCGGCTGGCATTTATGGTACACGAATACCATTT
CTCCGTTCAGACGCCGCGCCGACCATCGCCGATTCGTTTCAATCAACGAAAACGACGGGAC
GACGCGTCAACAGTGATGCGAGTCCGTCGCGTTCCCGATCGCCGACCGACCACACGAATCG
TCAACGTTTCGAGGAATCGTTGGGCGCTACGTTATGAGGCACGAGATCGACTCTCGTGAGT
GGAACAGCACTCGGGGAGATTGAAGTACGTAACTTTTCATTCGATACTTTGTACGTAAACG
TCGTGATCGATCGATCCATCGATCGAATTATCATTCGAAAGAAATTTAAAATTTCCTCTCGA
AATTCCGAAACAATTCTATTTTAATAACTCGAATCATTTTCACAGAAATTTGAACGAATTTT
GAAGTTTATATTAATTTATATTAATCATTGAATAGTTGTATTGATTATTTCAGAATGAAATCG
AAGGTACGATATATATTTTATACTAATGTAAATATCGTGGCGATTGCATCAACGAATTTTGA
AGTTTATATTAATTTATATTAATCGTCGATTGAATAGTTGTATTGATTATTTCAGAATGAAAT
CGAAGGTACGATACTTTATACTAATGTAAATATCGT

266. >MSTRG.5517.4 Gene=MSTRG.5517
CCTAGTGACATCTTTCATTAAACGAACATTACTATTTCTATTTCTTTTCAATTGGCATATTAT
ATCCGGCTTACAGAAATTTTGGCGAGTAGACAGTTGTTGCATCAATCTGACAATCTGTAAC
GAGTCAAGGAACAAAATACCTATCAGAATGTCAACTTTTCTTGGGCTTATTAAGGAGATAC
CTGGCATCTCAGTGGATCGTTTTGATGGAGCAAACTTAACATCTTCTATATTTTTTCTTAGTC
ATTGTCATTCTGATCATATGCATGGTTTATCAGATATGTTTTTTGAGCATATAGATGAATATA
ACAAATATCTCTATTGCAGCCCTATTACA

267. >MSTRG.5528.2 Gene=MSTRG.5528
GCTCGACAGATGGCGAGAGCATTAGCTCAGTCCCTGCTACGCACCCGCTGTTCGCCATTGT
ATCGTGATTGCTGTTAAGTTTCGTGTTTGTGAGTGAGAATTCATCCGGTTTATATCAAGTTCT
GGAGTTCTTTTACGCATCGGAGAGTTCAAGTTGCAAGGGAGAGACGTTGTGAGAGCGAAGC
GCAGGCAGTAGCTCAAACAGGGTTTACTGCCGATCCATTCCGGAGGGAACCGCGGCGTAC
ATCGTTATTGAAATAATGAAGAGAGAAGCTGATGCAGGCGCTATGACAGGTTCTGGAGGA
CCGACGAGTCCTCACAAGCGTTACAGACAGGGTGACGATGAACTTCGTCTTCTGATCCCCA
GCAAGGTAGCCGGTTCGATAATCGGCAAGGGTGGCCAGAATATCACCAAACTGAGGAGTC
AGGGTGCTTACCATCAGCTCGGATCTGCCCACCGTACTCCAAGTGTTGAACGAGGTCGTAC
CTAATCTCGAGGAGAACGGATCTCGACACGGGAGCGACGAGATCGACGTGCGCATGCTGG
TGCATCAGAGCCAAGCCGGATGCATAATCGGCAAGGGAGGTTTAAAGATCAAGGAGCTTC
GCGAGAAAACTGGGGCAAGGATCAAGATTTATTCTCACTGCTGTCCCCATAGCACTGACCG
GCTGATCAGCATCTGCGGAAAACCGACCACTTGCATCGAGTGCATTCGCGAGCTGATCGCC
ACTATTAAAACCTCGCCTCTGAAGGGAGTGAACAACCCTTACGATCCGCACAACTTCGACG
ACTATTACGCGGACGACTACGGCGGCTATGGGACCGGCGACGGCGGCCAAG

268. >MSTRG.5536.3 Gene=MSTRG.5536

GAGATTCAAGTAACGGAAAGATCGGTTCCTCTGGTCCAGCGCGGTCCGCGTTCGCGTCTCG
TATCCTTTTCCTTCTAATGCGTGATCCTCACGATCTATCTGTGGATTGGATCGCGTTACGCGG
CCACCACGTGTCAGAGGAATCGCTGCATCAGTCCGGAAAGTGTGAAGTGCGTGAAAAAAG
TGTTCTCGGTCCAGTGTTGTCGCGTGTGTCGTTCCGAGGCCAATTCTGATGAGATTAAGAAG
GTTGCGTGTATCTGTGCGATATAATTCTCCTGTAGTTGGTTACTGGAAACAGAGAACGCGTT
TCTCTACCACGGAAAAGCATTTTTTTCCATAATCTTGGTGATTTTACTTTGAACTGATCCATT
AACGAAGTGAGAATAAGCTGAACAGTGTATGAAGTCACCTATCGTTGTTAGTTCGTTGAAT
AGGATATAATCGAGCGACACGCAGTGTAACAGTGATTCGATGTGGATTTTTTGTCGGTCTCG
AATGATCGATCGGAGTTCCTGTCTTTAGATGGTGGCTCGTCGAAATTGCTCGGATTATCGAT
ATGTTCGTATTAGGTACTGATTCCCTGGAATAATAACGTTTCGTTCCATTCGAAATTCTCTCG
AAACGTGTCGACACAATTTTCTGGAAACGGTGTATATACGAGAAGCAATAAAGAAATGTC
CGCGTAATAACGGAAGAAGAAAATTTGAAAAGCTTTGGTTCGTCCAAAGAGTAAAAAAGA
AAAAGAAAATAAAGAACAGTGGTAAAACGGTGGACGTAATTCGACAGCATCCGGTGCCTT
CTTTTCTCTATCCGCTCCGACGAGCCGGGTCGTAAGGATGAAGCGAACCGGTAAACATCGG
AGCCATGGCTTTCTCTTGGGCATCATTAGGGCGGGCGCTTTTTATTTGTACCGCCATATTAAA
CATGCTATCAGAAACGAGATCGTTTTTTTGTAACTTGGACGATATTTTCAAAATGATTCCAC
CCATTCCTTGGGTTTTCTCAAAACGATCAATTTCACCTCTCCAAACTTGATCCAATCATATTT
TTACATCGAAAAAATCGAAATATAATATGCCATCGAAAACATCGTATAAGAAATAACAAC
AGAGAAGCATACTGCGCAATATTTATGTTTCATAACGTTCGCTTGGGAAATGTCTACGTCCG
CATGTTGACTATTCGCAGGAAGGTAATCAGCGAGTTTTCCAAGAAAGGTGGATAAACATTG
TTTTGAACGTAAAAAGGAACTTGGTTTACATAGCAAACTCGTTCAACTCTCGCCGATAATA
ACATCCAACGGTATCTCCAGAATTATAAAGATGACGTAAATGTATTCGTACGAGAATTCGA
GCGTGATCGCGTTGAAACGGACCCTTCGGGCAGTCATTTTGGAATATCGTGAAGCTCGTTTC
GAGGCCGATCGTCTACTGATGCATCCTGGACGCGCACCAGGTCCTCGTGGCGACGAGGTGG
GCACGATCGCGCGCGTTCCAGCCTCCGGGGCCGGGTAGAAGTGTTCGAAGGGGGAAATGA
AATCTTCGGTCGGATTCGAGTGATCGCGAAAGGAAGCAGGCGTGGAGACGGGGTTTCAAG
GGCGCGAACGAAGATGACGTCGTCGATCCTGGCGCTGCTCGGCCTAATCCTGGCGACCGCC
AGCGCCGAGCTAAGCTCAAGGATCGTCAGGACAAAGTACGGGGATCTATCAGGCGTTATC
GTGACCCTCGATCGCCACCTCGAGGGCGTCGAGGTGTTTCGTGGCGTTCCGTACGCGTCGCC
GCCGATCGGCTCGTTGCGTTTCATGCCTCCCGTGAGCAGCGCGTTGTGGCACGGCGTTAAAG
TGGCGGACAAATTCGGACCGGTCTGTCCCCAAAGGCTACCCGAGCTCAGTGACAAAATGC
CGAAAGGAAGGGTCGAATATTTGAGGAGATTGCTGCCCTATCTAAGGAACCAGAGCGAGG
ATTGCTTGTACTTAAACGTCTACGCACCGGTCCAAGGTAAGAACGAAACGAACGAGTGATC
GAGTGATTCCGTCGTAAGTGGAAAATTGGACGTCATTTCTCGGTTTAGAGGATACTTTGATC
ACGGTGACGCGCGAGGATAGGTAAGGAACTAAGTACTTTATCAACGGCAGATCCAGGGAT
CGGATCACCCTTTGATCCCTTTGACGAGATTAGATCCATTAATGTGAAGACTGGCGCATCCT
CATCTA

269. >MSTRG.5546.1 Gene=MSTRG.5546

AAAAAAAAAAAAAAGAAAAAAAAAATGAAGATTACGAAGCTTCGTTTTTCATAAAAAAA
AAAAAATTCCTTTATCATTAACGTAGTATTATTTTTCACAATGCGATTCCTGTTTAATTCACA
TAAAATTGTCTTCCTCGTTCTTCGCTCGCGATTCACTTTCGTATCGAAACTGCATTATGTTAT
ATATATATACACATGTATGTCTTCTCATTGAAATCTCATTATATTTCTTTTTACGATTATTAT

TTCTTTGCACACAATTTGCGATTTCGTTCTACGTTATGCACGAAACGAAGAGAGAGAGAGA
GAGAGAAAAAAAAAGTTGAGGAACGAAGTTGTCTCGTGACGGCACAATTTTTCAATTTATA
GGACCAAGGTGTCGATTAACGACGTCTCGATGTTATAACGCAATTTCGATGTTTGTTTTTTT
TCTTTTTCTTTCTTTTCTTTTCTTTTTTTTTTTGTTTTTCGTTTGATCGAAATCGAATTTGGTGG
AAATTTCATTTTCGATTATCGCACACGTCAATTAAAACTGACCAAAATCGCGTAAAAATTTG
GAAAATCGAACAAGTGAGCGAGCACACAATTTGTACATTGTTAAAAAGAGCGTGTTTTACG
TTACGAGTCAATACGAGAATTATCACGCGATTATTTTCTGTATATAGTCATTAATAAATTCG
TTAAGGTAGTCAGCGTTTGATGACAAGTGATTCGCAAAACTTCGACGAAGTAATATCAGGA
CCAAGAGTGGGAGAAATAACGAACAATAAGAAAACGCAACGCGATCCTAAGGACGATAG
ACTTGTTGACAATTTTGAGAAAGACTTGATAAAAGAAGAATTTCTTACACGAAATGAATTTT
CGACGAATTTCTTATTGGAATTCTATTTAAATCCTTTTTGAGAGAAACGACACGCTTTTTGTA
TACCAAGAAACATACCAATAGGGGCTTTTGGGGCGCAATTTCTAATATTTCAGCCCCGCGA
AACTTACAAGTCTGATTTTTGTATTTTATAGGAAAGGACCTAAGCATCGATTAGATTATTTA
TATAGGAGCTCTCGAGTTAATAATTGCCAAGTCGTTGATGGAATCCGTGTTAGTTTTCGATG
TGTAGACCAGAAAGAGAGAGAAAGAGAGAGAAAGAGAGAGAGAGAGAAAATTTAGATA
AAAAAGAGTACATTGTATAGTAGTCGATGTTTTGTAAGCTTTTGTATTTTTGTAAATATTCAA
TATCGATGTCTCAGTTCTTGCGGATGGCGACGAAAACCCTGCAAGAATCAATGTTCAGTTA
ATATATTACTGCGTCCTTAATATCATATGTACTAATATATAAAGAAATATACCGAATAAACG

270. >MSTRG.5604.2 Gene=MSTRG.5604
TGTCAAGTCGATATAAACATAACCTAGTCAATCATAATTCAATCTTTCACGATTTATAGAAT
ATTTGTTTATTGTAATTTATTGTCTTCAAAATACAAGGGATGGTGTTTACAAGAATACAATT
AGATTAATTTCGCATAAATGAAAATTCGTCTGATTAGAATTATGACAGTGGAAAAGTGTTA
GTTGCTGCATGTTACCTGCCCTGTATGTATGTATATACTTCAGTCCTTTATTATACACTATTCT
CGGAAAGTTTTGTTTTCTTTCATAAATTCTTGACCGTAACAGAAAAGAAAACATTGTCATGC
CGTTGCAAGTTCACGATGTTCAGCAAAATGTTGTGGAGCCTATGCAAGTTGATGCTCCTGCA
GAAGATAATGATAATGCAGAAGAAGAACCATATATTGTGGAAAACCCAACATTGATATTG
TATGCCAAGGATGTCGATCAACGTAGCACAACTTTCGAGAAATCTATGAGTGTAGGAAAA
GAATATCAAAGACGCACACGCATGTTAATCTTAAGGGCTGCAATGTTAAAACAACAGATTC
AAGTTAAAAGTCCACAGCGTGACAGTACTCAAGGGGGAGAGATGTCGGTTACCTGGAAAC
AATAGTTCAGTGATGACCTGATGTGGTTATTAAGATATGAAATAGTACTTTGATACCATATT
GGGCAATGAATGTTTATGATGCTGATATTGAATTCAAATCTTCGATAAAAGTTACAAAATAT
GTATATAATGCATTATACTATAAGTAAAAGCTAGTATTTTCAAAAATGTGTTTATTATGAAC
AAACCTTTGATAAAATATAATGTATACTAAAAACTTTATTATCAATAAATACACGAATACC
TTTTTTCAGGCATGA

271. >MSTRG.5615.1 Gene=MSTRG.5615
TCAGTAGAGATGCCGCGTCCGCGAGCAAAGCGTATCGACGACGGTGTCAGTTAGCCGTAG
GGGTAGCATCGGGGTGGTTCCAGCCGAAACTACGGCTCCGACCGTCCTCCACTTCCGCTTC
GCCCGTTTTCCACCATTTTTTTTTTCCATCATCCTTCGCCTCTCTCTCTCACTCTCTCTCTCTCT
CTCTCTCTCCCTTCGATCGTTGTTCTTTCCATCATCGACGTGTGTATATATATATACACGTTTT
GGCGACAATCCGTATTTTCGTCAAGGGAGGGAGGATATATTGGTGGTGGATCGAAGGTGGA
AGGAAGAGAGAGGAGAGTGAGATGAGAGACGAGGAGCACGGGAAGGATGTCGAAGAGG

AATCGAGAGGCGAGAACGAGAGAGAAACGTACGATAAGTAGATCGAGGCGATCGCTCGC
TTCCCTTCCTTTTTTTGCTCTATAACCGCCCTCCCCGTACACCCGCGATCGATTTCATTCCCTC
GCCTTGGGATAAACGATTCTTCGTGGTTCGAGCGGCTGCTAACCTTTGAGCGCGGTTAACAG
CAAGGCAAACATCGTTACCTTAGCCAGGAGTGGGTTCTTCCTTGGCGATCGTTTCATTTGGG
AGGAAAAACCTCTCGAGGTTTAAGGAGGGAGGGAGGGAGGGGAAATCGTTCGATTCGAAC
GCGATCGAGGAAAGGAAAACGCGATCGAGAGGTGGGCACGCGATGGATGGTAGATGGAC
CTAGGGGAGTAATTAGGCGCGAGGGGTTGAAAGGCGGGAACGATGACGATACTCTTCCTC
CAGAGCTCGTGGAAAAAGGAGAGTCGCGAGAAACCGGGTGGTCCTTGGGATTCGCTGTAA
TTAGGATCGCCGTGTAACTTTGCAGTTGGCACTCGAGTAGCCAAGAGCGGGTCTCTGAATTT
TTAATTTCAACTCGTACTCGCCGAACATTCTCTCAAAGAAGAATCCGTGCGTGCAACTTTCT
CTTCCGAACATGATGCATCGTGTCCTTTGAGAAGAGAGAGATAAAAATTAATATTTCGAAA
GTTTTCTCTTCGGATTCCGAATAAAATTCGATTTTTTCAGTAGAAAGAGCAGCACCGGGGAA
ATAGAGGAGACCGAAGAGCATTCGTGAGATGGGAAGATTTCAAGTTTGATCCGAGAACAC
GTCCGAAAAGTAACCTGGCTTAATCGTTTCAACGATGAATCGGGAAAACGAGGAAGTACG
TTCAATTCCAATTTTCATAACTTTATTCGAAAAAGTAAACGTGCCGCGAAAGTGAAAGATT
ATCTGAACCGATGATATATGTCTGTGTAGCAGCGACCAGTCTCCCAAACTCTTTGCGGGGC
GTTGCAAAAAGAACCGAATTGCAAATGTTTGGTGTTGTCTGTGTTGATATACGGATGTCTTG
CCGCAGTGACGTGGTGTAGATGCGCGAATGTCACCAAGGTGATAATCAATTTCTCGACGTA
TCCGATTAAAAGCACGAGACATGTGTCCCCTTGCGACGATGGCTATATATACATCCCCGTA
AGTAATCGCAAATTAAAAGTTAATTTCAAATTTTAATTCTTCGTTAAAAAATCGAGGAACG
AATATTCTTTATATTATTTTGAAAATTATGAAAAGGAATTACGAATACGGAGATTGGTTTTC
AGGTGGCTTTCATGGGGATGCTGTATCTGGTCTATCTGGTCGAGTGTTATCACTCGCCGATC
AGAATCGATCTGTTACACGCGGAGAGCCAGGACAGCGTGTTGTCGAAGATAACGCAGTTG
AAATTGGCGCAACCGACTATATGGTGGAAGGCTGTCTCCTACCATTACGTGCGGCGTAAAC
GACAAATTACCCGGTACAGAAACGGGGACAATTACACGACGACGCAGGTAACGCATAGC
GCGCAACGTGCAACGCACATCTCGTAATCCAGACGAAAGGAAATCGCGGGATGGTCTATT
ACGAGAGGATAAACACTCACGCCGCGACATCCTTCTATTATTACGACTACTGCGGGGTGAA
AGACATCAGCAAGGAGTTGATCCTTGATCCAAAAGTGCCGATCACCAAGATCAATCTCAGC
AAGGGATTCGCGTTTTCGAACATGAGATCGGCGACCGAATTCGAGGATGCCAGATCGAGAT
TCTTCGCCGAGCAAGAATTGAGAGACGATTACATGGAGATGAGAGAGGGCCTGGATCTCG
GATACAATCCGAATCCCACGACGCTTGTCGCCGTTCTCGGCAATCCTTGGTTCACCAATCGC
TACGTGTACTGGTGTCTCAGCGCTCTGTTGCTCAGCTGGCCCCTGAGAGTGATCATAGAGTA
CAAAACACAATACGCCGATTATCAGGTGACTATCGACTATCGTTCTACGCAATATCCCCCC
AATATCCCTTCTTTTCTCTCTCACCAAATCACCAGATCACCAAACTGTTCGGAATCAACTAC
GACACACCGAGCGGATGCGAGCCGATACACGCGTCCATGAGCCAGCAAACGATCAATCAA
CCGGGCTCTTACATGCTGGCCCCGAGCTACAGCGAGGCGCTTCTCATGGATCCAGCACCCG
ATCACCGTCCATCTTCGACGGAGGCCCAACAGGAGGAGGAGGCCATAACCGAGATGGTGC
CGAGCTATTCGGAAGCGTTGCTCTATCGACGAGCGGATCAAAGCTGCGTCCTGGATCCCAA
CGAGGATTCGAATCGTCCAACGTCTTTCTTCGAGTGTTCCTGTCCGTGCCACGCGGCCTCCG
TTCTCGAAACAGGCGCGCACGAGGAGGACGACGACGCGACCGGGCTGCCGTTGATGGGGA
CGGCGGCGCGCGTCGAGGCGCGTCCCCCCTCCACCTCGACTTGCAGGACGAGCGGCGATG
CGGGAGACACTACGGCGAGCGGGAAGTGCGCGAGTTGTGGGAAATACTTGGTCGAGGCGC
GGCTGGAAACCGCGGAGATGTGGTCGACGAGCGAGTGTACCATCTCGGGCTCGAGGAGAG

GGTTCCAAAGCGTGAGAAGGGGGCCGAGAACGATGGCTACGGCGATGACCATGCCTCGTG
ACATGTCCGAGCCGAATCTCAGATCGAGGTCGGAATCGTTGGAGGCCGACACGAGATTCTT
GAGATTGAACGGGCAGAGTTTGAGAAATATATTGGAGAGCGAGCAGGAG

272. >MSTRG.5615.4 Gene=MSTRG.5615
TGCCGCGTCCGCGAGCAAAGCGTATCGACGACGGTGTCAGTTAGCCGTAGGGGTAGCATCG
GGGTGGTTCCAGCCGAAACTACGGCTCCGACCGTCCTCCACTTCCGCTTCGCCCGTTTTCCA
CCATTTTTTTTTTCCATCATCCTTCGCCTCTCTCTCTCACTCTCTCTCTCTCTCTCTCTCCCTT
CGATCGTTGTTCTTTCCATCATCGACGTGTGTATATATATATACACGTTTTGGCGACAATCCG
TATTTTCGTCAAGGGAGGGAGGATATATTGGTGGTGGATCGAAGGTGGAAGGAAGAGAGA
GGAGAGTGAGATGAGAGACGAGGAGCACGGGAAGGATGTCGAAGAGGAATCGAGAGGC
GAGAACGAGAGAGAAACGTACGATAAGTAGATCGAGGCGATCGCTCGCTTCCCTTCCTTTT
TTTGCTCTATAACCGCCCTCCCCGTACACCCGCGATCGATTTCATTCCCTCGCCTTGGGATA
AACGATTCTTCGTGGTTCGAGCGGCTGCTAACCTTTGAGCGCGGTTAACAGCAAGGCAAAC
ATCGTTACCTTAGCCAGGAGTGGGTTCTTCCTTGGCGATCGTTTCATTTGGGAGGAAAAACC
TCTCGAGGTTTAAGGAGGGAGGGAGGGAGGGGAAATCGTTCGATTCGAACGCGATCGAGG
AAAGGAAAACGCGATCGAGAGGTGGGCACGCGATGGATGGTAGATGGACCTAGGGGAGT
AATTAGGCGCGAGGGGTTGAAAGGCGGGAACGATGACGATACTCTTCCTCCAGAGCTCGT
GGAAAAAGGAGAGTCGCGAGAAACCGGGTGGTCCTTGGGATTCGCTGTAATTAGGATCGC
CGTGTAACTTTGCAGTTGGCACTCGAGTAGCCAAGAGCGGGTCTCTGAATTTTTAATTTCAA
CTCGTACTCGCCGAACATTCTCTCAAAGAAGAATCCGTGCGTGCAACTTTCTCTTCCGAACA
TGATGCATCGTGTCCTTTGAGAAGAGAGAGATAAAAATTAATATTTCGAAAGTTTTCTCTTC
GGATTCCGAATAAAATTCGATTTTTTCAGTAGAAAGAGCAGCACCGGGGAAATAGAGGAG
ACCGAAGAGCATTCGTGAGATGGGAAGATTTCAAGTTTGATCCGAGAACACGTCCGAAAA
GTAACCTGGCTTAATCGTTTCAACGATGAATCGGGAAAACGAGGAACAGCGACCAGTCTCC
CAAACTCTTTGCGGGGCGTTGCAAAAAGAACCGAATTGCAAATGTTTGGTGTTGTCTGTGTT
GATATACGGATGTCTTGCCGCAGTGACGTGGTGTAGATGCGCGAATGTCACCAAGGTGATA
ATCAATTTCTCGACGTATCCGATTAAAAGCACGAGACATGTGTCCCCTTGCGACGATGGCT
ATATATACATCCCCGTGGCTTTCATGGGGATGCTGTATCTGGTCTATCTGGTCGAGTGTTATC
ACTCGCCGATCAGAATCGATCTGTTACACGCGGAGAGCCAGGACAGCGTGTTGTCGAAGAT
AACGCAGTTGAAATTGGCGCAACCGACTATATGGTGGAAGGCTGTCTCCTACCATTACGTG
CGGCGTAAACGACAAATTACCCGGTACAGAAACGGGGACAATTACACGACGACGCAGGT
AACGCATAGCGCGCAACGTGCAACGCACATCTCGTAATCCAGACGAAAGGAAATCGCGGG
ATGGTCTATTACGAGAGGATAAACACTCACGCCGCGACATCCTTCTATTATTACGACTACTG
CGGGGTGAAAGACATCAGCAAGGAGTTGATCCTTGATCCAAAAGTGCCGATCACCAAGAT
CAATCTCAGCAAGGGATTCGCGTTTTCGAACATGAGATCGGCGACCGAATTCGAGGATGCC
AGATCGAGATTCTTCGCCGAGCAAGAATTGAGAGACGATTACATGGAGATGAGAGAGGGC
CTGGATCTCGGATACAATCCGAATCCCACGACGCTTGTCGCCGTTCTCGGCAATCCTTGGTT
CACCAATCGCTACGTGTACTGGTGTCTCAGCGCTCTGTTGCTCAGCTGGCCCCTGAGAGTGA
TCATAGAGTACAAAACACAATACGCCGATTATCAGGTGACTATCGACTATCGTTCTACGCA
ATATCCCCCCAATATCCCTTCTTTTCTCTCTCACCAAATCACCAGATCACCAAACTGTTCGG
AATCAACTACGACACACCGAGCGGATGCGAGCCGATACACGCGTCCATGAGCCAGCAAAC
GATCAATCAACCGGGCTCTTACATGCTGGCCCCGAGCTACAGCGAGGCGCTTCTCATGGAT

CCAGCACCCGATCACCGTCCATCTTCGACGGAGGCCCAACAGGAGGAGGAGGCCATAACC
GAGATGGTGCCGAGCTATTCGGAAGCGTTGCTCTATCGACGAGCGGATCAAAGCTGCGTCC
TGGATCCCAACGAGGATTCGAATCGTCCAACGTCTTTCTTCGAGTGTTCCTGTCCGTGCCAC
GCGGCCTCCGTTCTCGAAACAGGCGCGCACGAGGAGGACGACGACGCGACCGGGCTGCCG
TTGATGGGGACGGCGGCGCGCGTCGAGGCGCGTCCCCCCTCCACCTCGACTTGCAGGACGA
GCGGCGATGCGGGAGACACTACGGCGAGCGGGAAGTGCGCGAGTTGTGGGAAATACTTGG
TCGAGGCGCGGCTGGAAACCGCGGAGATGTGGTCGACGAGCGAGTGTACCATCTCGGGCT
CGAGGAGAGGGTTCCAAAGCGTGAGAAGGGGGCCGAGAACGATGGCTACGGCGATGACC
ATGCCTCGTGACATGTCCGAGCCGAATCTCAGATCGAGGTCGGAATCGTTGGAGGCCGACA
CGAGATTCTTGAGATTGAACGGGCAGAGTTTGAGAAATATATTGGAGAGCGAGCAGGAGG
A

273. >MSTRG.5651.1 Gene=MSTRG.5651
ATTTGTGCTATTTCTTCTTCGCGTAGAAGAAGGCTAGATCTATTACCCATATTTTCTTTGATT
GATCAGGAAGTGACGTTCATACAGCATAGTGTCCACGTAGCGCCTCAGCTTCTGACGAGCG
TAGTTTTTCGTACGTCAAATATAACAGTTTGTGAAGCGTTCTTCATTTATACATATTTTACAC
AAATACAATGGATCAAGTGAAGAAACTTACCGAACCTGGACGTCAATTTGCGAAAGATAG
TATTCGTCTTATTAAACGATGCACAAAACCTGATAGGAAGGAATTTCAGAAAATTGCCATT
GCCACTGCCATTGGTTTTTGCATAATGGGTTTTATAGGATTCTTTGTCAAATTAATTCATATT
CCAATTAATAACATCATTGTGGGTTCATAAACTCATTGAATAGGTATATTTAAGATTTAATA
AGATTATATTTCCTTCCCTTTTTCCAAAGTATCTTTATTTATAAAATAAAATTATGAATTTTGT
TTTGAATTGATTGTGACTGAGATATGAGAATGTAATATTAATATTATTGAATTCTATAATAA
AATGTGTGTGTAGTA

274. >MSTRG.5728.1 Gene=MSTRG.5728
ATTACGACGCCACATTTTTCGGCGTTTGGCTATTACAAAGCACGAGTGTATTAAACGTTGCT
TGTTAATTTTTGAAATTATCACAAAAATGTCGTACAAAGGACCTCAAAAAGTTCAAAAAGT
GATGGTACAACCCATCAACCTTATATTTCGATACTTGCAAAACCGTTCGCGCGTGCAAGTTT
GGTTATTCGAGAACATCAACCTACGTATCGAAGGTCACATCGTTGGATTTGACGAATATAT
GAATTTAGTACTGGATGATGCTGAGGAATATCATTTGAAGACAAAGAACAGAAAACCACT
CGGACGAATCATGTTAAAAGGCGACAATATAACGTTAATACAAAATACGAATCCAGGAGC
GAATTAAGTTACTCATAACCTTGGAGGATGAACAAAAAAGTGCATTGTTTTATACTTTGTGC
TATTGAGCATGTAAACTACATGTTGTGTACAAGTGACTTAAAAACATTTAATAAGGGCAAA
GTTAATATCTTTCAAGTTCACCATATTTGTGATACATAATTCAATGTAATTAGAAAGAACAA
TGGTGTAAATATTGTGATTGAGC

275. >MSTRG.5737.1 Gene=MSTRG.5737
TTTCTCTTTCATTAAAAATAATTCAATTATTTAATTTCCAATCTTTGAACGTTCCACTTGCTCA
TGTTTTACTATTATATATACATATGTTATATAACTATTTTATATACGCTGTGCCGCGATGTTCC
TCGTTGAAAATTCGGATGGAAAGTATTTCTATACCGAAATACGTAAAAATAAATGACGTAA
AAATATCGTCGACCAAGACTGCGATTAAAGCAAATACCGCAAAGTTTTTGTCAATTAAGGC
AATACGTTCGTTAGGCTTTCCTTCGGACTAGTTGTTTAAAAGAGTATATTTTCACGCACATTA
TTGCTTGAATAAAATACTTGCTTAAAGCCGAGGGAGTTTGGAGAGAATAGGGGGTCGATTC

AATTTTCGTAAAAATCGAACGAGTTTGAAATTTCCCTTCGTGCGATATATTCCTGGTGGCCT
CTAGTTACCTAGGTCAGACAAAACGTCGAGCTAGTCTCTAGACGCTAGTCTTTACGCTGT

276. >MSTRG.5747.3 Gene=MSTRG.5747
GTTGCATAAAGCGCGCAAGAAGTATGGAAGTAAATTTGAAAAGTTTTAACGAAGCTTAATT
CAATGAAGTTGACAGATTTCCACGAGACAATAATCGAACAGTGTACAGCACAAAGTAGAA
AATTTCCCTCAACTACTTTTATATAAAGGAATCAACTATATTCTACCAACTTACTCAGTCTA
CTACAACTCTTCGATCAAGCTTGCACAAAACGCTCGAGAAGAGAATTTGAATAGAAGCGTC
TCGAAAGATGTTTAACGGAGCATAATTCAACAAGAAATTAACAAATTTCTAAGGAGTAATA
ACCGAATGGAATAAAAATTAATATTCAAAATTTTCCTCAAGATCTGTCAATGAACGCGAGT
AGCTACATAGTGTCAGTGGTGCTCGTCCTGATAGTCGGAGCACTGGTGGGTGTCCTTGCGAC
GGTATCGCATCATCTTCATCATCGTAGGCTGCTACTGCACGAGCCTGCTTCCGCCTCGATCT
TGCACCACCATCGGCATCATCATCACCGCCATCATCATCATCATCATCATCGCGAGGAACTT
CCGGCGTCGCACCAACGACGCCTCCTGCTGCAGGACCACCACCGTCATCCTGGGACGCCG

277. >MSTRG.5776.3 Gene=MSTRG.5776
CAGAAGTACGTTCACTCGATAGGACGATTGTCGTCTGGCGCACGACAAGCTTCAAGCAATA
TGGCGAAGTGTGTCATGGGCTCGGTGAGACGTAGTGTCGTTTGAAAAGATGAAAAGTTCGC
GGAATGGGTTGAGAATAATGTAGCGCTATGAAGATCGTGTGAATATTATATTTGTGTATTCA
AAAGCATTGAGTTGTTCCTTGTGATCGGGATATACAGTGCGCGTTACCGGTGTGAAATCATG
GCTACGGATAAAATAAAGGTTGCCGTTCGGGTTCGGCCGTTCAATCGTAGAGAGCTGGAAC
TCGGTACACAATGCGTGGTCGAAATGTCCGGACAGCAAACCATCCTTCAGCATCCCACCAC
GATGGACAAGATCGAACGGAAAATTTCATCCCTGGATCTTCAAACGGATTTTTATTTTTGTC
GATTACAACTTTCTAAATATTTAATTATACAACG

278. >MSTRG.5788.1 Gene=MSTRG.5788
ATGCGTAGTCACCTCCTCGTCGGTGAGACCCGGTAATTGGCATCGCTTTCCAAAATCATATT
TCACAAAATTTATTATTTTTCATTAAAGTTTCGAAATATTTTAAGCTTAAAAATATGAAATA
TATTTCATGAATGATAGTGTATGGAAAGCGATGGCAAGCTAAGAACTACGCAAATCTGCAG
TCCAGTGTAAACTTTGCATAATTCATTTAATTATTTTGACTTTTAATTTTTAATTGTTTAAACT
GGACTGCAGATATTAGAATAAGTTTATTGTAATAAGTGCGTTTCCATCCGTATCAAGATATA
CTCCATAATTTAAAGCGAAAACTAAAAATTTTTAATTTTTTTTACTCCTTTTTTATATTCTCGT
TTTTCGTTTTCATTTCGAGTTTTTATTCGTATAATTATTGCAATCGTTTGTTACTTTCTTCCTCA
AAATATCCTTTTAGTTATTTTATATTTTCGTCTTCTCTTTCGTCACTTATTTCAAGTCAATCTCC
TGTCATGCAACAGTGGTCGAAATCGTTGGGCCGGTGCTCTACTTCCGGTGGCCAGTTGAGG
GTCCGCGCTCTAATCGATTGGTACCTTGGTACTCGATTAGAGTTAGGTCTTGTGGTGGAACG
T
CGTCTTTGTAATCCGGCTCAGGTGGTCAAGGATTTACTCCTTGTTCCTTCCTGTGTCGTTGGG
ACATAAGATGTTTGGCTATTAGGCCGCTGTCGTAACCCTCGATTGGTCACGACTGGCAAGT
ATCATCTGTCCATCGATTTTCGTAAATCCATTGATGTAAGTCGTGATATGAGAGCGACAGAT
AAGGGTTGATTTTTCATCGATCACGTATCTTAAGTTCTTCTTCAAATGCTTATCATTTTCTTAG
CCTGCATTTAGTTGCTTTTTTAACTTTTTAAGTTTCCTCTTTTTTTTGTGTGTTTAGTATGAATG
ATTAGCATCTTCTTCCTCAATTTATCCTTTTAGTTATTTTATATTTTTTTCTTCTCTTTCGTCGCT

TATTTCAAATCAATCTCCTGTCATGCAACAGTGGTAGAAGTCGTTGGATCGGTGCTCTACTT
CCGGTGGCCAGTTAAGGGTCCGCGCTCTAATCGATTGGTACCTTGGTACTCGATTAGAGTTC
GGCCTCGTGGTCGAATGTCGTACTTGTAATTCGACTCCGGTGGTCAGGGATTTATCCTTGCT
CCTTCCTGTGTCGTCAGAACGTAAATCATTCTTCTGCGAGGCCAGTGTCGTAACCCTTGATT
GGTCACGACTGGCAAGTATCATCCGTCCATCGATTTTCGTAAATCCATTGATGTAAGTCGTG
ATATGAGAACGACAAAGAGGGTTGATTTCTTCTTTTAATGTATTCTTCTTATTATTTTCTTGAT
CTCCATTTATAGTTGCTTTTTGAATATTCTTTCTTTTTTTATTGATGTTTTATTTATTATAAATG
ATTTAGTATAAATGATTTTGAATATTTCTTTTTTTCGCTTTAAGTTAGACGAGAGTATTCTTGA
TACGGATAATATTAATAGAAATATTATGTATTGTAAATTATAAATTTAAGATTTTAAGTTAT
AGATTAGGTCATTATCATGGTTGGACGACGTGGGACGACGATGGACGACGATGGACGACG
ATGGACGACGTGGGACCTAATCTTTAATTTTATTCTATATTTCTTATAATTAATGCAAGCGTT
TTAGATATAATATAATATAAAGGGAAAAATGTAAAAGATTTTTGTAAAAATTTTTTGCATGT
TTATTGCATGTTTATTGCATGTTTATTGCATGTTTATTGCATGTTTATTTATTTTTATTTTTTATG
TATTTTATTGTTAATTTTATTGTTTTATTTTTATAATTTATGTAATTAATCTTTATTTTATGTTTC
AGATTTGTTTAATTTTTATATAATATTTTTCTTATGTATTTTTTATTTATTAATTTTAGGTCTCG
ATGTATAATCATCTCTTCTTTTTTTCGTGACTTTTTTCTCATGAATCATCATGAAAAAATTGTA
AGTGTTCATCGGACTGATGCTCAACTTACGGTGGTCAGTCGAAAGTCCGCGCTCTGATCGAT
TGATATCTTTGGATATTTGATCAGAGATAGGTCCTATGTTGAATGTCGTCTTCGGTAATTCGA
TTTCAGTGGTATTAGAATGATTTTTAGTTCCTTCCTGTGAGTCGTTAGGACTATGTCTTGGTA
ATTTCGACTCCGGTGGTCCTGAAATGATTTCTAGTTCCTTCTCGTGGTCGTTGTGGACTCTGT
GCATTCAGCTACTAGGCCACTATCGTAACTCTCTGCTGATCAACATGGTTATCATTTGTTTGA
TGACTCTTGTAAATCAACTATTGTAGAATTTAGATTTTCTTACACTATTTTTTTATTCTGTCAA
TAACTTTTTTTCGTTTGAAATAAAACGTGAATATCCTTAATATCAGTGATACCAATAGGATG
TATGACTCTTTGATTTAGTATTATCATTAGGTTTATTATATTAAGATAGATTTAGTCTCATCGT
TGGACATTGTAAAAAATATAAAAAAAAATGCTGTAAAGATTATTGTCAAATTTTATTGTATA
ATAATTCAAATGATTATTTTATTTTATGTATTTGTGATTAATATTTCTTTTACATTTCAGATTT
ATTTTATTTTTATATATTTCTAATTTGTTCAATTTAATGATTAATTTTTAATGTTTTTTTTTTTTT
AGGTTTAGGTGGGTCTCGATGCGTAGTCACCTCCTCGTCGGTGAGACCCGGTAATTGGCATC
GCTTTCCAAAATCGTAAATCACAAAATTTATTTCATTAATGTTTTTGAAATATTTTAAATTTA
AAAATATCAAATATATTTCATCAATAATAGTCTGTGGAAAGCGATGGCAAGCTAAGAACTA
CGCACACCTTCACTCTAGTCTAAAATATGATTGCTTTCAATTATTTTGATTAATTATCAATGG
ACTAGACTGAAGGTACTAATAAAAATCTGAACAAGGATATTCTGGTTAATCCTTTCTTGTTT
CTCAATTTTTAACAAATTGAGCAACGTTATCTCCTTCCA

279. >MSTRG.5788.2 Gene=MSTRG.5788

ATGCGTAGTCACCTCCTCGTCGGTGAGACCCGGTAATTGGCATCGCTTTCCAAAATCATATT
TCACAAAATTTATTATTTTTCATTAAAGTTTTCGAAATATTTTAAGCTTAAAAATATGAAATA
TATTTCATGAATGATAGTGTATGGAAAGCGATGGCAAGCTAAGAACTACGCAAATCTGCAG
TCCAGTGTAAACTTTGCATAATTCATTTAATTATTTTGACTTTTAATTTTTAATTGTTTAAACT
GGACTGCAGATATTAGAATAAGTTTATTGTAATAAGTGCGTTTCCATCCGTATCAAGATATA
CTCCATAATTTAAAGCGAAAACTAAAAATTTTTAATTTTTTTTACTCCTTTTTTATATTCTCGT
TTTTCGTTTTCATTTCGAGTTTTTATTCGTATAATTATTGCAATCGTTTGTTACTTTCTTCCTCA
AAATATCCTTTTAGTTATTTTATATTTTCGTCTTCTCTTTCGTCACTTATTTCAAGTCAATCTCC

TGTCATGCAACAGTGGTCGAAATCGTTGGGCCGGTGCTCTACTTCCGGTGGCCAGTTGAGG
GTCCGCGCTCTAATCGATTGGTACCTTGGTACTCGATTAGAGTTAGGTCTTGTGGTGGAACG
T
CGTCTTTGTAATCCGGCTCAGGTGGTCAAGGATTTACTCCTTGTTCCTTCCTGTGTCGTTGGG
ACATAAGATGTTTGGCTATTAGGCCGCTGTCGTAACCCTCGATTGGTCACGACTGGCAAGT
ATCATCTGTCCATCGATTTTCGTAAATCCATTGATGTAAGTCGTGATATGAGAGCGACAGAT
AAGGGTTGATTTTTCATCGATCACGTATCTTAAGTTCTTCTTCAAATGCTTATCATTTTCTTAG
CCTGCATTTAGTTGCTTTTTTAACTTTTTAAGTTTCCTCTTTTTTTTGTGTGTTTAGTATGAATG
ATTAGCATCTTCTTCCTCAATTTATCCTTTTAGTTATTTTATATTTTTTTCTTCTCTTTCGTCGCT
TATTTCAAATCAATCTCCTGTCATGCAACAGTGGTAGAAGTCGTTGGATCGGTGCTCTACTT
CCGGTGGCCAGTTAAGGGTCCGCGCTCTAATCGATTGGTACCTTGGTACTCGATTAGAGTTC
GGCCTCGTGGTCGAATGTCGTACTTGTAATTCGACTCCGGTGGTCAGGGATTTATCCTTGCT
CCTTCCTGTGTCGTCAGAACGTAAATCATTCTTCTGCGAGGCCAGTGTCGTAACCCTTGATT
GGTCACGACTGGCAAGTATCATCCGTCCATCGATTTTCGTAAATCCATTGATGTAAGTCGTG
ATATGAGAACGACAAAGAGGGTTGATTTCTTCTTTTAATGTATTCTTCTTATTATTTTCTTGAT
CTCCATTTATAGTTGCTTTTTGAATATTCTTTCTTTTTTTATTGATGTTTTATTTATTATAAATG
ATTTAGTATAAATGATTTTGAATATTTCTTTTTTTCGCTTTAAGTTAGACGAGAGTATTCTTGA
TACGGATAATATTAATAGAAATATTATGTATTGTAAATTATAAATTTAAGATTTTAAGTTAT
AGATTAGGTCATTATCATGGTTGGACGACGTGGGACGACGATGGACGACGATGGACGACG
ATGGACGACGTGGGACCTAATCTTTAATTTTATTCTATATTTCTTATAATTAATGCAAGCGTT
TTAGATATAATATAATATAAAGGAAAAATGTAAAAGATTTTTGTAAAAATTTTTTGCATGT
TTATTGCATGTTTATTGCATGTTTATTGCATGTTTATTGCATGTTTATTGCATGTTTATTTATTT
TTATTTTTTATGTATTTTATTGTTAATTTTATTGTTTTATTTTTATAATTTATGTAATTAATCTTT
ATTTTATGTTTCAGATTTGTTTAATTTTTATATAATATTTTTCTTATGTATTTTTTATTTATTAAT
TTTAGGTCTCGATGTATAATCATCTCTTCTTTTTTTCGTGACTTTTTTCTCATGAATCATCATG
AAAAAATTGTAAGTGTTCATCGGACTGATGCTCAACTTACGGTGGTCAGTCGAAAGTCCGC
GCTCTGATCGATTGATATCTTTGGATATTTGATCAGAGATAGGTCCTATGTTGAATGTCGTCT
TCGGTAATTCGATTTCAGTGGTATTAGAATGATTTTTAGTTCCTTCCTGTGAGTCGTTAGGAC
TATGTCTTGGTAATTTCGACTCCGGTGGTCCTGAAATGATTTCTAGTTCCTTCTCGTGGTCGTT
GTGGACTCTGTGCATTCAGCTACTAGGCCACTATCGTAACTCTCTGCTGATCAACATGGTTA
TCATTTGTTTGATGACTCTTGTAAATCAACTATTGTAGAATTTAGATTTTCTTACACTATTTTT
TTATTCTGTCAATAACTTTTTTTCGTTTGAAATAAAACGTGAATATCCTTAATATCAGTGATA
CCAATAGGATGTATGACTCTTTGATTTAGTATTATCATTAGGTTTATTATATTAAGATAGATT
TAGTCTCATCGTTGGACATTGTAAAAAATATAAAAAAAAATGCTGTAAAGATTATTGTCAAA
TTTTATTGTATAATAATTCAAATGATTATTTTATTTTATGTATTTGTGATTAATATTTCTTTTAC
ATTTCAGATTTATTTTATTTTTATATATTTCTAATTTGTTCAATTTAATGATTAATTTTTAATGT
TTTTTTTTTTTTAGGTTTAGGTGGGTCTCGATGCGTAGTCACCTCCTCGTCGGTGAGACCCGG
TAATTGGCATCGCTTTCCAAAATCGTAAATCACAAAATTTATTTCATTAATGTTTTTGAAATA
TTTTAAATTTAAAAATATCAAATATATTTCATCAATAATAGTCTGTGGAAAGCGATGGCAAG
CTAAGAACTACGCACACCTTCACTCTAGTCTAAAATATGATTGCTTTCAATTATTTTGATTA
ATTATCAATGGACTAGACTGAAGGTACTAATAAAAATCTGAACAAGGATATTCTGGTTAAT
CCTTTCTTGTTTCTCAATTTTTAACAAATTGAGCAACGTTATCTCCTTCCA

280. >MSTRG.5796.4 Gene=MSTRG.5796
GTTTGGCCTTGAAGAGATTTTCACTTAGTGGATTGATTTTTATATTTTTATTCTTTTCTTATTGT
TCTTGATATATTATTATATAAAATTTAGTTGAATATATTTTTAGCATTTTTTCAAAATTATAAC
GTATTTTATTTTTTTATGTAAAAATATATAGTATCACTTCTAATGGTTATGGCTGTGCCAGAC
CTGGACGACCTGGAGTCTATACGAAAGTGCATCATTATCTCCCTTGGATTGAACATGTAATT
TCCAGAGAAGATATTCGATCTTCAATAGCTTCTTGCAAAGGTCATCGATGTCCTTTAGGCGA
GTGTTTGCCAAAATCTCGAATATGTAATGGATTTCTAGAATGTTCAGATGGTAGCGATGAAC
GTAATTGTTCTATTAATTTATAGAAATGAAGACTGATTGTATTTGTATTTATTCGTTCCATTTT
ATATATTAAACGAACGAATTAATTAAAATAAAATTTCAATATATAAATTTTGAAAACAATT
AAAGC

281. >MSTRG.5812.1 Gene=MSTRG.5812
GCCGAAATTTCGACGTTTACACTTTTTTTTTAATATCTCGCTTATTATTCACAATATTAAAAA
ATGTTTCAAATAAAAGTTGATTGGTTTCTAAAGGGCTATATTATGACGCTCTCAGTTCTTTAG
TAGGTTGACCAATAAGGAGCTTTTTTAGCACAACTTTGTTTTTTTTACTGACAACATGTATTT
TTTAAAGCGCCAATCGATAGAGCATAAAATTTCCTATAAAAAAGTATTAACCCACTTATAG
CGAAAAACCAATAGTTTAAAAGATATAGCAAAAATTAAAAATTTGTAGGAGGTCGACCTC
CTGTCATATCATGTTCTCCTAATACAAAATCGAGTTTTTGGCCAAAAATTTTTCTTCGGTCGC
CGAAATTTCGACGTTTACACTTTTTTTTTAATATCTCGCTTATTATTCAGAATATTAAAAAAT
GTTTCAAATAAAAGTTGATTGGTTTCGAATGGGCTATATTATGACGCTCTCAGTTTTTTAGTA
GGTTGACCAATAAGGAGCTTTTTTAGCACAACTTTGTTTTTTTTACTGACAACATGTATTTTT
TAAAGCGCCAATCGATAGAGCATAAAATTTCCTATAAAAAAGTATTAACCCACTTATAGCG
AAAAACCAATAGTTTAAAAGATATAGCAAAAATAAGAAATTTTTAGGAGGTCGACCTCCT
G

282. >MSTRG.5812.2 Gene=MSTRG.5812
TTAATATCTCGCTTATTATTGAGAATATTAAAAAATGTTTCAAATAAAAGTTGATTGGTTTC
GAAAGGGCTATATTATGACGCTCTCAGTTTTTTAGTAGGTTGACCAATAAGGAGCTTTTTTA
GCACAACTTTGTTTTTTTTACTGACAACATGTATTTTTTAAAGCGCCAATCGATAGAGCATA
AAATTTCCTATAAAAAAGTATTAACCCACTTATAGCGAAAAACCAATAGTTTAAAAGATAT
AGCAAAAATAAGAAATTTTTAG

283. >MSTRG.5855.1 Gene=MSTRG.5855
ATCGGGTTGAACTTTGATTTAAACATAAAGCCACTTTGTTTTAAATATTAACGAATGCGTAA
GACGTAAGATCGAAAACTATATATAGCGCTGTGTATAGGCGTGTAAATACACGAGTATTGA
TATATTGCACGTACAATCGGATTCTTATATCTCATCATTGTTACGATATTAAAGTTCATCAAA
GACGCGATAAAATCGTAAGAATCGATCTATACCGAAAGAACTGCAAAAATAAAGTTTAGT
TCCGTTATTATTCCCATTTTCGCCAACTTCTAAATCCGAACCGCTACGCAAAGGACGTATTA
TTTTTTTTTCTTTTATTCCTTCATTCTTATTTCTTTCTTCTTTCTTTTCCTCACTTCTCACGTGTCT
TTTTTCCATTTATTTTTCTTTCTTCTTTAAGGCGAGAACGGACAATTAAAGATCAATTTCAAA
TAAAATCTCTTGTAGACCGAGGTGATTAAATTATTCTTTGATCTCGAAATATTTATATGTAG
GATTTTCATATCCATTGATTTGCATGTTTGCAACATGCCGCTCCTCTGGTGTGGCCGCTTGAT
CAACTTCAATAAATCCCTGACTATGAGGGCTTCTTGCACTGCGCCTTTTGAATACTGCTACA

CCTACTACTGCTGCAGCCATAAGTGCAATTCCAGCAAATGCAAGTGTAAAGTAGACAGATC
GACTTTCTCTGTGATACATCTCCCTTCTTACAGAGTAACCCGGTTCAGAATGGGAAATGTCA
TGTGTCAAACTATGAGCTGCCCTAACAAGTCCTTCGCTGTGCATAGCTGCCGGTTGCTCCGG
TTGTTGTTGAATATCTTTGTTATCATTTATCGAATCGCTTTCTTCTTGTTGTGCTTGTTCTGTAC
GTAATTCACCACGTTCAGCTTTGCGTTGCTCCTTGCGTACTCGTTCCAGTTGTTTTTGATGCTC
TTTTTCTTGTTGCTTACTGGTAACATCGGCCTGATATTTATCTAAAATGGCTGCTTCCGCTTCT
CGTGTCATCGCTAACAGCAGTCCAGGAGTCTCATCTTTGCTCCTGAGGGCCAAAACGTAAT
CATCCATAAGGCGGCCAATTTTTGCGCTTAAATCAGGATACCGTTCTAACATCAACAGGCT
CTGATTGGTGATTCTATCGATTTCCGCCAAGTGCTCCAACGTTGCTGGTTTCTCACGTTGAGC
TTGTTCCAAACTGCTGTCCAGCAGATGTTTGTAATGAGCAATAGTATGATGCCTATCTTTGT
GAAGAGCACGAAGCAACTTTTGGAGACATTTTTGAATGCGATGCATATTTGGTGGTGTCTCG
TTCAAAGCAGCTGTATAACATGACATCGCTTCTTCGTTCCTTTGTTTAACAGCTTCTTGTACT
CTTTGCTGATGCATAGCGCTTAATTGATGTTTCTCCGCGGCACCGCTCTCTTCTAGAGCAGC
AACAGTTTCCTGGAATCGCGCGGTCATCCATCGTTTGAAAGCATCAGCAGCAACTGGGTCG
TGGGCACGGATATCTTGATACTTTTCCTCGAGATCACTCCAGTCCTTCATCACTTTTGTTACT
TTCTCCTTGTGCACCTCTTCGAGTCTCATTTGTGCTTGCTTGTAGCTCTGGTGCTCAGAACGA
GGATCGAAATGAGTTAGATATGGATCCGGTGTGCTCTGTGACTGCGAAGATTGTTGTGTGCT
GGTACTAACTGGCAATGGCATTGCAGTTACGTCTTGTTTCGGTTTTTCCGTGGTTGAAACTGT
TGTTGATAATCGACTTGTTGTAGTGTCGTAATTGTAATCATCGTTGAGATATTCCTCGTCACT
TTCATCATCATCAGGTTGATCATTGAGTCCGTCTTCGGGATCAGCTTCAGAATCATCTTCATC
ATCTGCATCAGTATCCAAACCATCATCGTCTTCAAGGTCGTCTTCATCTTCGTCGAGATCTTC
GTCCACACCCTTTGGAATCGGCAAGTCGATCGGTTTTTTGGCTTTCAAATTTTCTTTGAGGTG
TTTCGGACAGCACACGAATTCGACGCCAGAGAACAAGGATATTCCGCAGGGCAACAGCAT
TGCAAAGCTCCTCAGATTCATATTCCTCTCTCTGCAGGTGTCTGCTGCGGTCGCATTCCACCT
GTGAGATTCCCAGCATTTCGTCTGATTGTGAAGGTGGTCGAACAGGCAGCCTTCCGGAACG
AGCAAAGCGTCGCTTTGGAACGGCCCCTCGAGACATCTGTACGGCTTGACCCAACGTGACA
ATTTGCATTTGGTTCTTCCTGGCTTGCACCATCCGCTCACCCTTACATAGTGCGACGACTCTA
CGATGTTGGTGATGTCTCTTTTCGGGTAAGCCTTCTTGCAATATTCGAGAATCTCCAGCTTGT
CCGTCATGCAACTCTTCTTGTCGGTCGAGGAGACCCAGCGTCCCTGTTCTCCCATGTGCTGT
GCCAGATACACCTCTCCAGCCTCGCACAATGTGGCAACTTGCGGCTCTGATCTTCCTCCAGC
TGGTGCCAACTCGAGCCTCTCCGACACCGCTTGAACCCGGCAGACCATCAAGACGCCCAGC
AGTGCGGGCACCAGCATCCCCTTCATCTTTCTTCCTTGATTGACTGTTCGTAAAACTCCCGC
ACGATGTGTCGTTTGGATTCCTCTCGTAATGATTCCTGGCTTAGCTCGATCTTCTACGAAGAA
ACGACGATGCACTGACTAACAAACGCCAGAAACGTTGCAGAACAAATTGCTATATGCAAG
ACACCACTCACCCGCCGTATACGAGAGCACAGCGTACTACTGTGACACTGCTGCTGGGTGG
GTCGCTTTACGAGAAATCAAGATGGAGGTGAAACGCGGCGCCGGTGCCGCTTCATCTCTTT
CTTTTTTTCAGGAAAGCATGAAAACGCACGAGCAGATAGCCTTGGCCCATACCTTTGCCCG
TGTGTCGGCCACCGTACCCACTGGTTACCCTGGAAACGGTATCGATTTCAACCCTCAGCTAT
GCAGTAGAAACCAGCGGCACACCATGGGGGTCTCTTCATCCCGAGTTTCGACAAGACAGC
CCGTTTAAACTACCCGATTACACCAACCTTCATCTCTATTACCGTCTTACATTTCGTTTCGCG
TACTTCACAAATTCAGTTCTTCAAACGATATTTGAGGTTAGGGGACACTGTGAAATTAACGG
CGAAATTTGGAACTATTACGAACTATTTTTTGATTCAATGATTTCCGCTTTTGTAGGCGGCT
AATTCGGCGTATTTATTTATGTTAATATAAAATCTTCTTTATTATATGAAAAATATATGTTTA

TAGAAGAATATTACATTCAGTTGAAAAATAAATGCATTACATTGGCTGACACAGAGATCTA
AATGTCCCAACAATAAATACAACATATTCTATATGTATATATAGC

284. >MSTRG.5855.3 Gene=MSTRG.5855
CTTTCTTTTAGATTTGTTGTCGTCATCATTATGAGCGCTCCTTCCTGTGTTTATTTGTTTATTTA
TTTTGTTAATAATAACATGTATGACAAACGAAAACAACGTGTATGAGATACTTCCGAAGAC
AACATTACGCCCTTCCCATAATATGGCACATTGGCATGTTGTTTCCCTTTCGAATTGTCTTGT
AGATAATTCTGATACGAGCAGTATGAATCGACTCATAACATTTCTATCTACTAGATGTTATA
TGAAAATTGCTTTATACATATATCATAATAATCGTTATATTTTCGTATAACGAGTCGCGAGTT
ATTATAAAGAGACGTGACACATAATGATATCGTATCGCAATACCTATTCGTCTATGGACAG
ATTTTATTCCATATATCACTAAGACATAGATTTTTTTTTTTTTTTTTTTAAATATATTTTCGGA
ATCTTCGGAAGAACCTCGAACCTGAAAGATATCTTACACTAACAAAATACTATGAGATTCT
TGAAGAACATACGCGAGAACGGACAATTAAAGATCAATTTCAAATAAAATCTCTTGTAGA
CCGAGGTGATTAAATTATTCTTTGATCTCGAAATATTTATATGTAGGATTTTCATATCCATTG
ATTTGCATGTTTGCAACATGCCGCTCCTCTGGTGTGGCCGCTTGATCAACTTCAATAAATCC
CTGACTATGAGGGCTTCTTGCACTGCGCCTTTTGAATACTGCTACACCTACTACTGCTGCAG
CCATAAGTGCAATTCCAGCAAATGCAAGTGTAAAGTAGACAGATCGACTTTCTCTGTGATA
CATCTCCCTTCTTACAGAGTAACCCGGTTCAGAATGGGAAATGTCATGTGTCAAACTATGA
GCTGCCCTAACAAGTCCTTCGCTGTGCATAGCTGCCGGTTGCTCCGGTTGTTGTTGAATATCT
TTGTTATCATTTATCGAATCGCTTTCTTCTTGTTGTGCTTGTTCTGTACGTAATTCACCACGTT
CAGCTTTGCGTTGCTCCTTGCGTACTCGTTCCAGTTGTTTTTGATGCTCTTTTTCTTGTTGCTTA
CTGGTAACATCGGCCTGATATTTATCTAAAATGGCTGCTTCCGCTTCTCGTGTCATCGCTAAC
AGCAGTCCAGGAGTCTCATCTTTGCTCCTGAGGGCCAAAACGTAATCATCCATAAGGCGGC
CAATTTTTGCGCTTAAATCAGGATACCGTTCTAACATCAACAGGCTCTGATTGGTGATTCTA
TCGATTTCCGCCAAGTGCTCCAACGTTGCTGGTTTCTCACGTTGAGCTTGTTCCAAACTGCTG
TCCAGCAGATGTTTGTAATGAGCAATAGTATGATGCCTATCTTTGTGAAGAGCACGAAGCA
ACTTTTGGAGACATTTTTGAATGCGATGCATATTTGGTGGTGTCTCGTTCAAAGCAGCTGTAT
AACATGACATCGCTTCTTCGTTCCTTTGTTTAACAGCTTCTTGTACTCTTTGCTGATGCATAG
CGCTTAATTGATGTTTCTCCGCGGCACCGCTCTCTTCTAGAGCAGCAACAGTTTCCTGGAAT
CGCGCGGTCATCCATCGTTTGAAAGCATCAGCAGCAACTGGGTCGTGGGCACGGATATCTT
GATACTTTTCCTCGAGATCACTCCAGTCTTTTGTTACTTTCTCCTTGTGCACCTCTTCGAGTCT
CATTTGTGCTTGCTTGTAGCTCTGGTGCTCAGAACGAGGATCGAAATGAGTTAGATATGGAT
CCGGTGTGCTCTGTGACTGCGAAGATTGTTGTGTGCTGGTACTAACTGGCAATGGCATTGCA
GTTACGTCTTGTTTCGGTTTTTCCGTGGTTGAAACTGTTGTTGATAATCGACTTGTTGTAGTGT
CGTAATTGTAATCATCGTTGAGATATTCCTCGTCACTTTCATCATCATCAGGTTGATCATTGA
GTCCGTCTTCGGGATCAGCTTCAGAATCATCTTCATCATCTGCATCAGTATCCAAACCATCA
TCGTCTTCAAGGTCGTCTTCATCTTCGTCGAGATCTTCGTCCACACCCTTTGGAATCGGCAAG
TCGATCGGTTTTTTGGCTTTCAAATTTTCTTTGAGGTGTTTCGGACAGCACACGAATTCGACG
CCAGAGAACAAGGATATTCCGCAGGGCAACAGCATTGCAAAGCTCCTCAGATTCATATTCC
TCTCTCTGCAGGTGTCTGCTGCGGTCGCATTCCACCTGTGAGATTCCCAGCATTTCGTCTGAT
TGTGAAGGTGGTCGAACAGGCAGCCTTCCGGAACGAGCAAAGCGTCGCTTTGGAACGGCC
CCTCGAGACATCTGTACGGCTTGACCCAACGTGACAATTTGCATTTGGTTCTTCCTGGCTTG
CACCATCCGCTCACCCTTACATAGTGCGACGACTCTACGATGTTGGTGATGTCTCTTTTCGG

GTAAGCCTTCTTGCAATATTCGAGAATCTCCAGCTTGTCCGTCATGCAACTCTTCTTGTCGGT
CGAGGAGACCCAGCGTCCCTGTTCTCCCATGTGCTGTGCCAGATACACCTCTCCAGCCTCGC
ACAATGTGGCAACTTGCGGCTCTGATCTTCCTCCAGCTGGTGCCAACTCGAGCCTCTCCGAC
ACCGCTTGAACCCGGCAGACCATCAAGACGCCCAGCAGTGCGGGCACCAGCATCCCCTTC
ATCTTTCTTCCTTGATTGACTGTTCGTAAAACTCCCGCACGATGTGTCGTTTGGATTCCTCTC
GTAATGATTCCTGGCTTAGCTCGATCTTCTACGAAGAAACGACGATGCACTGACTAACAAA
CGCCAGAAACGTTGCAGAACAAATTGCTATATGCAAGACACCACTCACCCGCCGTATACG
AGAGCACAGCGTACTACTGTGACACTGCTGCTGGGTGGGTCGCTTTACGAGAAATCAAGAT
GGAGGTGAAACGCGGCGCCGGTGCCGCTGAAACGATTCGACCAATCAAAAGCTGTCACTA
GATGACCGCGCCGGAGGGTGCGTTCACATGCGCGCAATGGTCAGTCGATAATGTGGCGGG
AATATCGTGATTTTAAATGATATACTTCACCGTGCTATGTAATTGTCAACTTTTGACAGATTT
TCCGGCGG

285. >MSTRG.5855.2 Gene=MSTRG.5855
CTTTCTTTTAGATTTGTTGTCGTCATCATTATGAGCGCTCCTTCCTGTGTTTATTTGTTTATTTA
TTTTGTTAATAATAACATGTATGACAAACGAAAACAACGTGTATGAGATACTTCCGAAGAC
AACATTACGCCCTTCCCATAATATGGCACATTGGCATGTTGTTTCCCTTTCGAATTGTCTTGT
AGATAATTCTGATACGAGCAGTATGAATCGACTCATAACATTTCTATCTACTAGATGTTATA
TGAAAATTGCTTTATACATATATCATAATAATCGTTATATTTTCGTATAACGAGTCGCGAGTT
ATTATAAAGAGACGTGACACATAATGATATCGTATCGCAATACCTATTCGTCTATGGACAG
ATTTTATTCCATATATCACTAAGACATAGATTTTTTTTTTTTTTTTTTTAAATATATTTTCGGA
ATCTTCGGAAGAACCTCGAACCTGAAAGATATCTTACACTAACAAAATACTATGAGATTCT
TGAAGAACATACGCGAGAACGGACAATTAAAGATCAATTTCAAATAAAATCTCTTGTAGA
CCGAGGTGATTAAATTATTCTTTGATCTCGAAATATTTATATGTAGGATTTTCATATCCATTG
ATTTGCATGTTTGCAACATGCCGCTCCTCTGGTGTGGCCGCTTGATCAACTTCAATAAATCC
CTGACTATGAGGGCTTCTTGCACTGCGCCTTTTGAATACTGCTACACCTACTACTGCTGCAG
CCATAAGTGCAATTCCAGCAAATGCAAGTGTAAAGTAGACAGATCGACTTTCTCTGTGATA
CATCTCCCTTCTTACAGAGTAACCCGGTTCAGAATGGGAAATGTCATGTGTCAAACTATGA
GCTGCCCTAACAAGTCCTTCGCTGTGCATAGCTGCCGGTTGCTCCGGTTGTTGTTGAATATCT
TTGTTATCATTTATCGAATCGCTTTCTTCTTGTTGTGCTTGTTCTGTACGTAATTCACCACGTT
CAGCTTTGCGTTGCTCCTTGCGTACTCGTTCCAGTTGTTTTTGATGCTCTTTTTCTTGTTGCTTA
CTGGTAACATCGGCCTGATATTTATCTAAAATGGCTGCTTCCGCTTCTCGTGTCATCGCTAAC
AGCAGTCCAGGAGTCTCATCTTTGCTCCTGAGGGCCAAAACGTAATCATCCATAAGGCGGC
CAATTTTTGCGCTTAAATCAGGATACCGTTCTAACATCAACAGGCTCTGATTGGTGATTCTA
TCGATTTCCGCCAAGTGCTCCAACGTTGCTGGTTTCTCACGTTGAGCTTGTTCCAAACTGCTG
TCCAGCAGATGTTTGTAATGAGCAATAGTATGATGCCTATCTTTGTGAAGAGCACGAAGCA
ACTTTTGGAGACATTTTTGAATGCGATGCATATTTGGTGGTGTCTCGTTCAAAGCAGCTGTAT
AACATGACATCGCTTCTTCGTTCCTTTGTTTAACAGCTTCTTGTACTCTTTGCTGATGCATAG
CGCTTAATTGATGTTTCTCCGCGGCACCGCTCTCTTCTAGAGCAGCAACAGTTTCCTGGAAT
CGCGCGGTCATCCATCGTTTGAAAGCATCAGCAGCAACTGGGTCGTGGGCACGGATATCTT
GATACTTTTCCTCGAGATCACTCCAGTCCTTCATCACTTTTGTTACTTTCTCCTTGTGCACCTC
TTCGAGTCTCATTTGTGCTTGCTTGTAGCTCTGGTGCTCAGAACGAGGATCGAAATGAGTTA
GATATGGATCCGGTGTGCTCTGTGACTGCGAAGATTGTTGTGTGCTGGTACTAACTGGCAAT

GGCATTGCAGTTACGTCTTGTTTCGGTTTTTCCGTGGTTGAAACTGTTGTTGATAATCGACTT
GTTGTAGTGTCGTAATTGTAATCATCGTTGAGATATTCCTCGTCACTTTCATCATCATCAGGT
TGATCATTGAGTCCGTCTTCGGGATCAGCTTCAGAATCATCTTCATCATCTGCATCAGTATCC
AAACCATCATCGTCTTCAAGGTCGTCTTCATCTTCGTCGAGATCTTCGTCCACACCCTTTGGA
ATCGGCAAGTCGATCGGTTTTTTGGCTTTCAAATTTTCTTTGAGGTGTTTCGGACAGCACACG
AATTCGACGCCAGAGAACAAGGATATTCCGCAGGGCAACAGCATTGCAAAGCTCCTCAGA
TTCATATTCCTCTCTCTGCAGGTGTCTGCTGCGGTCGCATTCCACCTGTGAGATTCCCAGCAT
TTCGTCTGATTGTGAAGGTGGTCGAACAGGCAGCCTTCCGGAACGAGCAAAGCGTCGCTTT
GGAACGGCCCCTCGAGACATCTGTACGGCTTGACCCAACGTGACAATTTGCATTTGGTTCTT
CCTGGCTTGCACCATCCGCTCACCCTTACATAGTGCGACGACTCTACGATGTTGGTGATGTC
TCTTTTCGGGTAAGCCTTCTTGCAATATTCGAGAATCTCCAGCTTGTCCGTCATGCAACTCTT
CTTGTCGGTCGAGGAGACCCAGCGTCCCTGTTCTCCCATGTGCTGTGCCAGATACACCTCTC
CAGCCTCGCACAATGTGGCAACTTGCGGCTCTGATCTTCCTCCAGCTGGTGCCAACTCGAGC
CTCTCCGACACCGCTTGAACCCGGCAGACCATCAAGACGCCCAGCAGTGCGGGCACCAGC
ATCCCCTTCATCTTTCTTCCTTGATTGACTGTTCGTAAAACTCCCGCACGATGTGTCGTTTGG
ATTCCTCTCGTAATGATTCCTGGCTTAGCTCGATCTTCCGCCGTATACGAGAGCACAGCGTA
CTACTGTGACACTGCTGCTGGGTGGGTCGCTTTACGAGAAATCAAGATGGAGGTGAAACGC
GGCGCCGGTGCCGCTTCATCTCTTTCTTTTTTTCAGGAAAGCATGAAAACGCACGAGCAGAT
AGCCTTGGCCCATACCTTTGCCCGTGTGTCGGCCACCGTACCCACTGGTTACCCTGGAAACG
GTATCGATTTCAACCCTCAGCTATGCAGTAGAAACCAGCGGCACACCATGGGGGTCTCTTC
ATCCCGAGTTTCGACAAGACAGCCCGTTTAAACTACCCGATTACACCAACCTTCATCTCTAT
TACCGTCTTACATTTCGTTTCGCGTACTTCACAAATTCAGTTCTTCAAACGATATTTGAGGTT
AGGGGACACTGTGAAATTAACGGCGAAATTTGGAACTATTACGAACTATTTTTTTGATTCAA
TGATTTCCGCTTTTGTAGGCGGCTAATTCGGCGTATTTATTTATGTTAATATAAAATCTTCTTT
ATTATATGAAAAATATATGTTTATAGAAGAATATTACATTCAGTTGAAAAATAAATGCATT
ACATTGGCTGACACAGAGATCTAAATGTCCCAACAATAAATACAACATATTCTATAT

286. >MSTRG.5855.4 Gene=MSTRG.5855
AAATAAATCTGGCAGCATATAGTATATATCCATATACGATCGAAAGAAAACATTGTCGATC
GAAACGACAATTTATAGATGTACTTGTCTATCATAGTCACCATTACACACCGGTAATAACA
GTATGAATAAAGAAAGAATAATATTTCGCGCTATGACATCATTTACATTCGCCTCTAATACT
TAACCATGCTGTAATACGTAATCAAGACGAGACAGAGAAAAAGACAGAGGAGACAGAGG
AAAGAAGACGAAATCAAACGGAAAGCATGAAAACGCACGAGCAGATAGCCTTGGCCCAT
ACCTTTGCCCGTGTGTCGGCCACCGTACCCACTGGTTACCCTGGAAACGGTATCGATTTCAA
CCCTCAGCTATGCAGTAGAAACCAGCGGCACACCATGGGGGTCTCTTCATCCCGAGTTTCG
ACAAGACAGCCCGTTTAAACTACCCGATTACACCAACCTTCATCTCTATTACCGTCTTACAT
TTCGTTTCGCGTACTTCACAAATTCAGTTCTTCAAACGATATTTGAGGTTAGGGGACACTGT
GAAATTAACGGCGAAATTTGGAACTATTACGAACTATTTTTTTGATTCAATGATTTCCGCTTT
TGTAGGCGGCTAATTCGGCGTATTTATTTATGTTAATATAAAATCTTCTTTATTATATGAAAA
ATATATGTTTATAGAAGAATATTACATTCAGTTGAAAAATAAATGCATTACATTGGCTGACA
CAGAGATCTAAATGTCCCAACAATAAATACAACATATTCTATATGTATATATAGC
287. >MSTRG.5872.1 Gene=MSTRG.5872

TAATAAGGGAATTAGATATTAATTATTCATGACTTTTTATCATATGATAATAATAACACGTG
CAATAGATGATTCGTAATCGATATCACGATATCTTTCTCTCTGATATCTTTAGACACACTTGC
GTACGAAAAGCAGATTAAACACTGCATTTAACAGTGTAAGATATAAATTACATCGAATCA
AGATTGACACGTTCGTATTATTAGATTGTAGTTTAAATATAAATACATAAGCTTACCTGTGA
CAGAACACTCCTCGATAATATTTCGATGATTATTATGGCGATGAAATGATGCGTAATCTATT
CTTATGGTGGCAACAAAATTATTCACTGAAATACACTTACAAACAATCCTTAAACAATTGA
ACATGAATAAATAGAAAAAGACGAATACCAAATTTTCAATAATCATCTTAAAAAATATGA
ATAAAAAAAAAAAAGAGAGAGGACCAAGCAATACATAACATGGAAGAACCAAGTGTTTA
CCACAGTACAGTACAGGAGAAGATGATAACGAGTTCGAAGACTCGATAATCCATGGAAAG
GGGAGGGGGGAAAGACGCTGTATGATGACGACCCCTGAGGGTCGAGCATCTCGCCATTCTT
CGTTTGTCAGTCGAACTTAATTATCATTGACAGAAGGGGCACGAGAGCTTCGGTGTGCTTGC
TTCCGTGGAAATCGCGCGGAATATTGAGGACGAGGAGGAAGGAAGGAAATGAGCTCGAG
AAAACCGAGAAGGGACTCGGACGTCCTGTTGTCCAAGTTCGGTCACAAGGAGTTGCTGTTC
AAGTTCCTCGTAATCGGTGATTATGGAGTCGGAAAAACAGCATTGGTTAGGAGATACACAG
AAGGAAAGTTTACGTCGAATTACAAGATTACCATAGGAGCCGATTTCGCAATAAAAACGCT
CGACTGGGACCCTCTTACTAAAATAAATTTACAATTGT

288. >MSTRG.5885.1 Gene=MSTRG.5885
GCGAAATGAATGGAACCAATCCACCAATGAACAACATTCATAACATTCATCATAACATTTA
TCCCAAGTTGCTAGGCTCGGCTGGATGGTCCTCGATAATAATAAACAGGCGAAGAGGATAG
CTCCTCGGCGTAGGTGGAGGTTGAAGTTTTCTCTCCTTCGCTTGGTGGTGGAAAACGGCGGT
GGTGGATGGAACGGTTGATATACACCGGCGAAATGAATGGAACCAATCCACCAATGAACA
ACATTCATAACATTCATCATAATATTTATCCCAAGTTGCTAGGCTCGGCTGGAT

289. >MSTRG.5886.1 Gene=MSTRG.5886
CTCCGCTTGAAGGTGGCAGCACGGTCTTCGTTTCCTGTTTCGTTCTGACGTGCCGGACCCCG
CGATCTAATTAAGTGCGACGAAGTAAACGTCAATGTGCTGTGAAAATACTTTAAACTGTGT
CATATTGAAGACAAACTCCAAGTTTAACTCGCTAAAGTGGAAAACTAGTGGTTCCAAGTGG
CTGACACGACATTTAAGCCGAAGTTGAAAGCGTCGTCAACGAGCCACGACGAAACCGAGA
GTTATACACGTCGACATTAACGGTACGAAAAGTATAAACAATTCCTACTACGCGTCTCCTT
GAATAATCTCCGAATATATCAGTCTTAATTCGACTTCGAACACGATAACATCGGGCGCCGA
ACCAAAACAAGAGAAACTGCGACGTTCTCGAGACGGAGACCAACCACGGCGATCCTGTTG
GCCGCCGTAGGAGATGAAAATGCGATCTGTCGGTGGAGGGGAGGGTGTATGGCCGGCGCA
TCGACGCGTATCGACGGGTCGGAAAAACCGCGGCAATCTATCCGGGGATTGCTCGTGGAAT
CGGCTCGAGAAGGCGGCCCTCTCTGATTTCCTGGTGTTCTTGCGCGGCGATGTGCCGCTTTG
GCCGCTGCCAAGGAACGAAAGATAAACAGGCATAGCAGAAGTAAGTTTAGGTGAGAAGC
GTGGTCGTCTGTTGGGCTCGTACGAGGGGGAGGGGGAGGGGGGGTGCAATTAAACGAGAA
TGGAGGAGAAGAAAGAAAGAAGAGGATAATGAACGAGAGATCGAGTCGAGCTACTTATA
GTCCACGCCCTTTCGTGCGGAAACGCGAGCCCGAATTTTTAAGATAGTCTTCCATGGTTAAA
CAAGTCCTTTGTGTCTTTAAATGCAAACAGGAGTTTAAATCCAAACTCCTGGGAAGAAGCA
TGGATTCCAGAGAATAAGCAAATTCGCAGGAAATAGAGGTGAAAGGAAGGAAGAATTTA
GAATTTCTATAAATCATTGCCTCTTATTAATCCATTTTTTAATTAACAGTCAAGAAGAAGGA
GTTAAAAGAGGGGACGTTTAAAATTTCGTAAAATGGAGTTTCGTGAAATTAACACCGTTTC

TATTTGCAATAAAAATACGAAGAGGGATAACGACATAGAATAAAATGGAGAGAAAGAGA
GAGAGAAAAGAAATAGGCGAGTCGTTAATAATAAAGGATGGACATTTTTATTGGTGGACCT
TTATATTTCAACGATCGTCGACGTAATAACGCCCTCTTGCGACGTCTTGCAACGTAAAAAAT
ATTGACGTCAATGGCGTACAATTGGCGTACCTATCATCCCTTGATATCTGTTCGCAGACGTA
CCGGAAAGGAGGCTAATTAAAGTAGGGGTAGGTATCCCCCCCTCTCTGAAATCGTTTCTCT
AATCTTCGGATTCTTCCAGTTCAGACCAAGTTCGAACGCCTAAGCTAGCACATCCTATTCCA
TCTGGTAAGTACACTTTCTTTATTGATCTCTAC

290. >MSTRG.5886.2 Gene=MSTRG.5886
CTCCGCTTGAAGGTGGCAGCACGGTCTTCGTTTCCTGTTTCGTTCTGACGTGCCGGACCCCG
CGATCTAATTAAGTGCGACGAAGTAAACGTCAATGTGCTGTGAAAATACTTTAAACTGTGT
CATATTGAAGACAAACTCCAAGTTTAACTCGCTAAAGTGGAAAACTAGTGGTTCCAAGTGG
CTGACACGACATTTAAGCCGAAGTTGAAAGCGTCGTCAACGAGCCACGACGAAACCGGTG
AGAAGCGTGGTCGTCTGTTGGGCTCGTACGAGGGGGAGGGGGAGGGGGGGTGCAATTAAA
CGAGAATGGAGGAGAAGAAAGAAAGAAGAGGATAATGAACGAGAGATCGAGTCGAGCT
ACTTATAGTCCACGCCCTTTCGTGCGGAAACGCGAGCCCGAATTTTTAAGATAGTCTTCCAT
GGTTAAACAAGTCCTTTGTGTCTTTAAATGCAAACAGGAGTTTAAATCCAAACTCCTGGGA
AGAAGCATGGATTCCAGAGAATAAGCAAATTCGCAGGAAATAGAGTCAAGAAGAAGGAG
TTAAAAGAGGGGACGTTTAAAATTTCGTAAAATGGAGTTTCGTGAAATTAACACCGTTTCT
ATTTGCAATAAAAATACGAAGAGGGATAACGACATAGAATAAAATGGAGAGAAAGAGAG
AGAGAAAAGAAATAGGCGAGTCGTTAATAATAAAGGATGGACATTTTTATTGGTGGACCTT
TATATTTCAACGATCGTCGACGTAATAACGCCCTCTTGCGACGTCTTGCAACGTAAAAAAT
ATTGACGTCAATGGCGTACAATTGGCGTACCTATCATCCCTTGATATCTGTTCGCAGACGTA
CCGGAAAGGAGGCTAATTAAAGTAGGGGTAGGTATCCCCCCCTCTCTGAAATCGTTTCTCT
AATCTTCGGATTCTTCCAGTTCAGACCAAGTTCGAACGCCTAAGCTAGCACATCCTATTCCA
TCTGGTAAGTACACTTTCTTTATTGATCTCTAC

291. >MSTRG.5886.3 Gene=MSTRG.5886
CTCCGCTTGAAGGTGGCAGCACGGTCTTCGTTTCCTGTTTCGTTCTGACGTGCCGGACCCCG
CGATCTAATTAAGTGCGACGAAGTAAACGTCAATGTGCTGTGAAAATACTTTAAACTGTGT
CATATTGAAGACAAACTCCAAGTTTAACTCGCTAAAGTGGAAAACTAGTGGTTCCAAGTGG
CTGACACGACATTTAAGCCGAAGTTGAAAGCGTCGTCAACGAGCCACGACGAAACCGAGA
GTTATACACGTCGACATTAACGGTACGAAAAGTATAAACAATTCCTACTACGCGTCTCCTT
GAATAATCTCCGAATATATCAGTCTTAATTCGACTTCGAACACGATAACATCGGGCGCCGA
ACCAAAACAAGAGAAACTGCGACGTTCTCGAGACGGAGACCAACCACGGCGATCCTGTTG
GCCGCCGTAGGAGATGAAAATGCGATCTGTCGGTGGAGGGGAGGGTGTATGGCCGGCGCA
TCGACGCGTATCGACGGGTCGGAAAAACCGCGGCAATCTATCCGGGGATTGCTCGTGGAAT
CGGCTCGAGAAGGCGGCCCTCTCTGATTTCCTGGTGTTCTTGCGCGGCGATGTGCCGCTTTG
GCCGCTGCCAAGGAACGAAAGATAAACAGGCATAGCAGAAAGGGCGTTAACCATCGAGC
GGCTAATTGCAGTGAACAAGTGTGTGCACACGAGTGGCCCCTAAATGCAGGAAATAATGC
ATTTCGTGAGCCAGTCAATCGGTCATCTTATTACGAACCGAG
ACGATGAGCGGGTATATGACGAAGGTGGTCGGATCATCGGGACCAGACAATTTAATTGTTC
GAACTTAAATGGGGAGCAGCTGCCTCCTGGTGGCCTAACAAGCTGGTAATAACCTGCCTCC

TAACCTATCCCCTTCTTCGTCCGATCCACTCCACCACCGTTCGTGGATCCTCGTTTGGAGGG
GTACGGTTAAAGGAGAGGAGCATTAAACGTTGAAGGTTATTTATTTGAAAGCGAATTCATC
GAACGAATATTATTTTTCGAACGGGTCTTTCTCTTCTCTCCTTTTTCGGACGGGTAATTTTTTC
GATCATTGAAATTAGAAATTATTATAGGGGGAGGGATTTTGATTTTTAAATTTTGACGATGG
ATGGAATAATTGGTGTATGAATTGTGAAAAATTTGTATATTTTATCGTGATTGATATTCGATA
AGGGAAAGGAAGGAAAAACGTGAAATGTGTTTAACGATCTTGAAAAGTTTGGTACAAATA
TAAAGGAAGGATTATAGAGGAAAGTGGTTGTAATGATCGAGTAATAATAATACGTGTGAG
AGAGCGGGGAAGGAGGAATGGAAGAAAGAAAGGTAAATATTAAACGGGGAAACGTGAA
GCCCACGAGGTCAGTACGTGCGAGGAG

292. >MSTRG.5911.5 Gene=MSTRG.5911
TACCATTTCCGGTTTGGGCGTAACATCATCTTCTACGTAGTTAGGTAAGAATAGTTTTACTTT
AGTTAAAAATAAATTTCGGAGTTATTTAACAAAATTTAATAAAAAAATTAACATTTAAATA
ATAGAAATAATGTTTAAATAATATTTGATTACAGATGGCAGATCAACAAGAAGATATCCAC
GGATTTTGGACAATCTTGTAGCATTTGCCGATGCATAGCCTATTTTCGACAGAGCATAGGGC
CACATCGTTTTTGTGTATAGGAAATGTTAAATTTCTTATACACAGCTGTGATCATATATTTTC
CATAAAAAGGATTTAATTAATAATCAAATATTTACGTCATGATCCTATGATTGTACTCCAAT
ATGCGTTAAATCATCAAAAATATCATCAAAATGGTAAGTCATATTATTTTTTAAAATTCACT
TATACTCTTTCTTCGTAAATTTTTTGAGAGAGGGCGCCATATACGAGAAATTACGACAAATA
TAATGGAAGGGGAAAGCCTTAGGATCAATAACCTTCGTTCATATGACCTCATATCCTATTTC
TGGCGTCATCAACTTTTAATGATCGTATAATATGAGCGTTTATGTAGTTTTATGAAAGTTTTT
ATAAAACTAATGTAATATATATTTGAATACTGATATTAAACAAAATGGGGAAGGTAAATGA
TCTTTGCGCTTTTCTTTTATATGCGATGTTATGTGAT

293. >MSTRG.5913.1 Gene=MSTRG.5913
AATCTTATATATTATAAATAATATGATACAAAATTCAATTCTTTAAATATGAGTATAACCTA
TCAAACTAAAATTTTGTTTATGGAAAGTGACTAATTTTTGGCCAACTAGCACAAATCATTAT
TTCATAAACTAAAAGTCTAAATTCATAATCAGTAAATTATTTAAAGCCAAAAGACATCATG
GAAGAAAAAAATCTAACTGATGAACAAAAGAAGTTTTTGGAAGAATGTGAAAATGAATTC
AAAGATCGATATACAGAAAAAGATATTGAATTTATGAAAGTTAAAAATATGGAATTAAAA
AAACCTCCAATCATTGATCCTTGGTATCCTAGAAGAAAAAGATATTTTTTTCCTCAAGAAAG
AGGTGAAATATATAGGAATTCTGATAGATATTATTCTCAACGTTATAGACCTT

294. >MSTRG.5929.1 Gene=MSTRG.5929
ATTGAGTACCTCTATACGGAAGTGACAGGAATTGCGATTGGTAGGGTAGCGTTATTTTATTA
CGTGTCATATACGCAAATCTTTTCTCTATTGCTTGAGAATTCCAAAAACGAGTTTTTGGGAA
GTTTTTTAATATTTGATTATCTTAATTATATACATATTAAAGCAGAAAGATGCGTCAATTGA
AAGGAAAAACAAAAGAAACAAATAAACAGAAGAAAGAGAGAAAAAAGGAATTTCTGGA
AAATAAACAACGTGTTTTTACGGTTGTGTTACCAACAATTGCTGCAATATTTGTTATAATAG
CAGCATACGTTTATGTTAAAACCCGTCCGAAAGTTGTTGAGTATTAATTGGCGTGTGTTCAT
TATTAATCATTAAAATACAATACCAATGTATTTCAAGAATGCTTGGACAGTTCCTTGATACA
TTTGTACAATTAAATAAGAATTATTTAAATAAGAAAGCAGATTTGAATGCTGCTCATATGAT
ACTTCATTCCTAACATTTAATAATCAAATATGAACATTGTGC

295. >MSTRG.6000.1 Gene=MSTRG.6000
GTATAGTTCGAGACACATTTAAGACGAAGCAAAATCACTCGTCTTATTCTTAACGCAAGAA
TATGCGGCACAACAACGGCTGAAAGCATACTGGCAAGAAGAGAGCAAATTGTAATTGTTA
ATAATTAATATCTTTTTTCCAATTTTTTTCGATCTCCTTAAGAAATTTATAGTATGCTCACTAA
GTAGGGCATAAAACTTTCAATATATAATCTGTTAACAAAGTAGGTACTGTTGGACTGTCTTC
ACTAATTGCTTTTAATATTTTTATAAGAACTTGTAATGCTTGATTGTCTGGTGGGCCACTGTC
TGTATGGTAAGGAATTACGGTAGTTAAATACTTAGATTCTTGAAGTGATCTATGTTGTAATA
AAGGACTAGTACCATTTTGCATTTGATTTCTTTTTTTTGTAACATGATGCTGTCTCCGTCGATT
TTGTACTGCTAACAACAATGAAATAAATTGATTTTTTAATTCTTTTTCATATTCAAGTTCATC
ACGCAATGCAAGTTCCGCAATGAGAGTTTCACTAAGCGCTCCCACCAAGGATTCCATTTCT
CCAAGTAGCTCGTTAAGCTGGCTGGAGGTAAGATGTTTTAACTTTTTTTCATGCAGCGGAGA
GCCCAAAACTTCTCTGCTTCTCTCCAACGCTTCGTCGCTATCTAATAAAGATCCATCAGAAT
CTGGAGATCTTTCCATGGATGGACTTTCCTGCATTATATCATCTATTTCACGGAGTACTTCTT
CGGCAGTTTTCAACGGTTCCTCTGGACTATGAGTATCGGTGCTACTGGACAAGATCAAAGC
ATGCATGTCCAAATCAGTTGCAACAGCCTCATCTTCGCTACTTAAATCCTCTAGTTCAGGTC
TTTCCTGCGAAACCGGAGCTTCGTTCAAATTAAGCGCAGGCATATGCATTTTCCTAGCATAC
GATTTACTCCAATCGATTGGTAATATGTTACCAAACGTACCGGTAATCGTCCACCACATTTG
ACATTCGTTCATAATTTCTTCTTGCGTTCGTACTTGCACGGGTGCTAGGGATTCAACGTTCGT
GCCGTAATCGCGGAAGCAGGATGTTATCTTCTCATCGAAGGTGTTAACGAGGTCCTCGAGA
CTGCCGCTGAAAGGGCTGAATGTCCCCTCTTGCAAAATATCAGTGCCATTTTCACGCGTAGT
CGCTGGCTCATTTGCGTTACAATTTTCTCCATAAGTCGTGTTTAAACTTGTATCCAGCAATTG
TTCTGTCAGTAAATTCATATTCTTCAAGCTGGCCGTGTCTTCCTCCTCGAATCGTGCGAGGGG
TGCCTCGAACTTCAACTCGGCGATCTTGCCCGCCATGTCCCTCATTATTGACCGTGTAGAGA
AGAACCGGCAGCGAAGTTTATCACCGTGATGAAATTGGGCACCGTACACTTGCGGCAGCG
CCCCCGAGGCGCACCGTTTTTGTCGTTTGCTTTATGATTCTTTTACAAGACAATAGAAAAGA
AAAAGACGATCGACATTAAAACCAATTGCCCGACTATGCAATATCCGCTGCCTGCGAGGCT
TCGCGAACGAC

296. >MSTRG.6051.1 Gene=MSTRG.6051
TGAATGTTGAGCCTAGTGTATGTTTGAGTTCGCAGAAATATTTTATTATGTAAATATACCAA
TTATATATATATATATATATATATATATATATATATATATATATATATATGTATATATACA
TATATATAATGTAAATTATATTAGCACGAAACATTTTAGCATGTCTTATTCATTGCTCAGTTG
TTAAATTTTAATGATACAATCAAATTATATTAAATTGAAAATATTCGTACGTGATACATTTG
TATTTTATTTTGTTAATATATTGATATAAAATTAATATTATGAGAAATGAAGAGAAAGAAAA
AGTATTATTTTTAATTTATATATAACATTTTACGCAGAGCGTAAATGTTTCTGTGGTTATGAC
TGTGGAGATTTAAATTAAACTTTTCAGGGTTGGTATTGTATATTTACGGCAATTTTTTTATTC
ATTTATTTATGCCGTAGTAAAGAACAGCCAATTGATTTAATTCTTTATCGTTGCTCTATTTTT
ATAAAGAATTAAATGATTGGTAGGATCGTAGAATTATACGAAGAAATAGATTGTAACATTA
GACTCACTTCCGTAACAAATTATGAAAATGTAAATTTATATAAATTTAAAAAGAAAAAGTT
TACGAAATTGAGTTATAATGTTACTCTTTGAAAAGTTTTGAAACATTCTCCCTTTTAAAGAGT
CAACTTGCTAATGCTAGTGAATATAAAAAGATATATATTTTATTATTATTTTATTTTATCCAA

CGCGCACGTGTGCATGCGTTGGTTATAGTCTGCGAAGGCACAGTATTTATATGTATAATTCA
CTTCAAAAATAGAATTTATGAATGGAATATTTATTGTAC

297. >MSTRG.6062.1 Gene=MSTRG.6062
GGAAATATTCGCGAATCGATAATTCGCGAACAACTGCAGCAACAACAACGGGTTAGTCAC
GCGAACGACTAGCCTATTCGATTATTATTCGAGCAAATCGCACAAGGATAAATTATAATAG
TCATCATGATGGAAAATTTGTTTTCATAAAAAACAGAAAGAATATAAAAAGAAATAAAAT
ATTCCGCAAAAAATTGTTTTATCAAAATT
TCATTATCTATGATAAATTTTTATTTTTTTTTTTT

298. >MSTRG.6104.1 Gene=MSTRG.6104
CAGTCTACGGCCCGGGACAGAGGGACCAGGACCAGCCTCGGGAAGGAGAGCTCGTCGTGG
ACCCTCTTCTCCTCTCGTACACTCGTATCGTGATCTATGTATTGTATCATCGTTCAGTGTTGCT
AGTGTTAACCTTGAATGTAGCGCAAGGTTCACGATTCAGTGTGATAATGAAATAGGATAAC
AAACTCGACAAGTCATCATCGATCTTTTGATGAGATTATATCGCTAGTGATTGGTAGTTGTC
TTCGATAGATAAATATTATTATCGTGATAGATACGTATCGAACGTTGTCATTGGTGGATTAT
ATCGAAGAAACCACCAATTATTGGTGGAGAAGTTTGGAAAGAAGATTGAGAAGACGCGAA
CGATGAGCGGAGTGGTTCGGCCGGGAACTGCTCGGAGCACGTGCCTCGCACGCAGAAGGC
GCTCCGAGCGGCACCAGGTGCGCTAATCGAGGACCGATTTGTGGGAACGAAATTGTGACG
GTGGTATCGATAAATAGAACGTTTCAAGGCACGTACACGCGTTCACGCGTTCGTTCTTTTTA
ACGAATTTCGGCGCATCGGTCACGTGATCTACGATTACTCGACGAGGGAAGGAAACCGGC
AAGCAGGAATGCCAGCAGCAGCTCGTGCCGTGAACACTTCGAGGACGATCTCGATGATCG
CTTCGTTGACCACTCTTTTTTTTACGTTCATCGGAATCGACGGCATGGGACATGTCAGTGGA
CACAACGTTGAACGGAATACTTTTCATCAAGAACTTCGAAAATACTTTATAGGATATTTAA
ACGTCCGACTCGAATAACACCTGAAACTCCATGACGATTGATTTAATCGAAAACTTGCCGA
CGAAGCCATTGAATTCTCAATACCGGCCGTACCACCGGTACCATTACTAATGAATCATTTCC
AACTTTGGACACAACGGCAGCTCGAACATGGACGAGCGTTCCAGTTACCACTGCCGATCGT
ACAGTTTCACAGGCATGGAGAAAGAAAGAAAGAAAGACAGATAGATGGACAGAGAGAGT
GAGAGAGAGAGGATGGAAGGGAAAAAATATGATCGATCGTGAAACGGAGGTCCACATTG
TGCTTGGAACGACGAAAATAAATTTGGCAAACGATCCATTTCCCTCGAGGTAGCTGGAAAT
TCATATCGACGCGGTTTCGCGTTTTCTTAGCTACGAAACTCTACGATTTTTTTTCCTTTCGTTC
TTTGCTAATTTACCAGCCACACATTGAAGTGATATATCACACGATTGTAATATAAAAGTTTG
ATTTATCCCGACATTGGTTGTAATTTGAGTTAATAATAG

299. >MSTRG.6147.1 Gene=MSTRG.6147
ATCGTCGATATCAGTTTGGCGCGATGCTCGATTTTCGAGGAACTCTTACAAAAGGAGGAAC
GTATATTTTTACGATTTAATATATATATACGTACCTGTTTGTTCTCATATCAAATGTTTCGATC
AAATGTTTCCGTCTCGTCGAGTATTATACTCTGTTTGCCGTAGATTTAAGGAAGCGATTTTCC
TTAGAAATCGATTTTTTTTTTTTTTTTTTTTTTACGTTTCCGCGACAACGATTTCAATGATCAA
AGAGACGGCATAAAACGAGCAAAAGAATTTAATAAAAATTCGTATTTTATTTACAAATATA
TATGCATAACGCTGTACAATCGTGCAAATATTTTTATCCCAGTCTGGGATTTAATTGGGGAA
AAAAAAAAAAAAAAATAAAAATAAAAAAACATTATCAAAAATATTTGTCGATATTTTACT
TTCGAATCATGGTCGGCTGTCTTTATTATATATTTCATTTCAAGTTTACCAAAGATTTATCCCT

GCGCGAATTCGATAAATACCATCGTCAAATACGGAAATTTTTCGCTCCGTCTCTTAATTTAC
ATAACAAAGAATAACACGTAAAATTGTTATCTAAAAAACGTTATAAACGATCGGTTAAAA
AACGATCGTATCTCGGTTTACAGATTTCCGTTCATTTCCTTCTTCTTCTCGCCGTCGGCAGTTT
CTCGTGGCGTCTCGTCCTCTTTAATAAATTCGCGACGCCTGTCGCACTCTTGGGCGCCGTAA
AGATCCAAATTCTTCCCGAGATAACAAACCGCTGCATCGAACAACACTCCTATTATCGAGA
ATCCTGCAGAGGTTATGTTGACTAAATATCGAAAGTTCCTTCCATGATGGAACCAGCAATTT
CCCCTAGTCCCGCAGGATTCTTCCCAGATCAAGCAAGTCGAGTCAATAATGGCGCCATAAA
TGATCGGACCCGGTATCAAAGCGAACAACGAGATAATCATCAAAGTAATACCCTGAGCGA
AGCTCTTGTCCTTCTTTTCGACGCTACGATAATTAACCAAAACGTTGCCAATCTTTCCAGAA
CACGCTAACGTTTGTATGATACAGGTGAGAACCATGAACAGAAGGTAAGGGCGACTGCAA
TCGTTGGTACAAGGGCCAGCTCTGACTTTGTCGAAGATCATCGAGTGTGGATCGCTAATGA
AGATCGATTGATCCCTCGAGTCGCCATCTGTCGTGTAACCGAAGTGATTTTCTAAATTGAAC
GTGGATAAAGGAAGGCAACTACAGTTGCCGAACTCTTTGTCGTTGATTATCGTTCGACAAC
CAGCATGGCAGGCGGAATAAAACGTTATCTTGGACGCCACGTGACAAACAGGAGAATATT
TAACACCGTCGCAATTGCAATCTATATTGCAATTTGATGTCAAATTCATTTGCATAGTTTCTA
AATTGAGACCTTCGAACCCGATATCGGAACAACCGAGAAATATAAAGAGTATCTGACCGG
CAACGAAACAAATGCCGACGAATACATTCCATGCTAACAATGGTCTTGGACCAGGTTTAAA
TTTGCTAATTATTAATCCGGAGAGTAAAAATCCTAGTACCATACCCACGAGTGATATAGGA
CCTGCGATTACAGTGCCACCAGCTGCAGAGGTACTGTATTGAACCTCGAGATATTTTGCCA
AAAATGTTATGTATGCCGATGCACCTAACACGTAAAACACTCCGCTCAGATTATTGAAAGT
CAACAGCCAATTCGTCAGTAACCTCTTCATCGCGTTTGGAAATTCACGCATAGTTGGAATAT
ACTCTGTTTCCAAAGACTTTCTCGGTTCCATCGGTTTCATGTATTGTTCCTGCACGGTCAAAT
GCAGTTTCAAGGGGATACTACCGTCCTTTTTCACAGTTTCGAGTGCAGTGTCTTTTTCTGTCG
GTAACTTTCGCGGAAACATGGCTATTAGTGTAGCAAACATGCCCATAGTGACACCTAGAAT
AATCCAACCGAGCCACCACGCACCTAACCATCGTGGATCCTTCTTTGTGATCACAGGATGT
AAACTGGGATCTATGTACAAACTGAGACAACCGTAACCTAGTAAAAATCCTATTGCTGGTC
CGACTGTTCTCAGAGCAAAGGTAAAACCTAAAAGCATAGGAGTATTCTTCTTCTTTGTATTG
TCATCCAGGTACGTTTGACCAAGACCGTAATACAAAGTGGTACCAATTCCAAGGATAAATT
GGGAGAGGAAGACCAAGAGTCGAGGTAGAATGCTGACATCCAACAGTGTCTCCTCGTCGC
AATGATCCGGTTTCACGACTCGTGGACAAATGGCCAAATCTTGAGGAACTGTGGTGGCGTT
CAGCAAAGTTTGATCGAGATATTCCTTGGTCAGCGCCAAAGCGTCTCGTCCCGGTCCGTATA
GAAAGTGTGGAAGAGCAAGGACGAGGCACGAGAGGGCGCTGAGACCGACACCGATCGCG
ATCCATCTTGGACGGTGACCAGACCCTCCATAATAGGTTAATATCAAAGACAAGATTTGCG
AGATTTCGTTCCCGGAGAGGATCAATCCAGTTGTACGACTAGGTATTTTGAATCTTTTTTCCA
AAGTCGTGAGGGTGACAACGATGTAAATAAAGGCCATTGCCTGCACGGTGCCGAGGAGGC
CGTACACCGCTATGAAATTTTTCGGGGTGGCACGATCACGTAGCCATCGGGGATAAATCCC
ACAAATTCCACAACCTTGGCTCATCCCTCCGACTGCACTTGACCTTAGGCCACGTGCTTCCG
CATGGACTCAGGAAGGACTTCCAGGTCGCGAGTCCTGTCTGGATCGCGATCCTCGAGAGTC
TTCCCTTTCTCGTCACTTCCCTCGTTTGACGGAGTAACTTGACGGGGGAAAAAAAAAAAGTC
ACCGAGCGCTATGAGCGCTCTAGTTTACGCGAATAAACGCGGGGTAACTGAGGC

300. >MSTRG.6161.1 Gene=MSTRG.6161

TGTTAACCTTCTTTAAATCCAATTTTTCGAATCTCCACCATTTTGACAATCGAACGCAATCCG
AGAGGAAATCTTTGTACACGATACAGAATATGGAACTTGAATGTAAACATTGTCGAGAGA
ATAAAAAGAACGCTCGTCTCTCCATTGTTAATTTATTCGGTATTTTCCTGCGGAATTGCTCTC
CAAGGAAGGGATAAGTATTCGAACAGGATGCACATGATATAATAATATAATGACGATCAC
GAGGTTATGAGAAAAGAAATGCGACCTGATACGATATGAAACACAGGCTTTCATTTTATCC
TCCGGAAAATAAACAGCGTGGAAAAATTACTGAGAGAAGCTTATAATTTTTTTAAGATTGA
ATAACTGTTACTAGTCATCAATTAGAGAAGACAATTGTTGCGTAAATTTTCTGATCGAAACT
TGAAAAGAAAAAATTCGAAGGGCAAAGAAACGATGAATGGAACCCTCCTCGCAGCTGAGG
GTAAAAAAAGGACGAACAAGATGGTGCCGTGAATGACGTTGTGTTGCTCTTTCGATGTGTA
TGGAATAAAGAATGACTAGTCTGAAATGCACCCTGTCTCGACACGATCGTACTGGGTACAC
AAGGGATTGAAGCAATTTCAAATTCCCCCTCCCTCCCGTTTAACATCTAACATTTGGCTAAG
CCGCTGGTGCAAATGAAGGAGAGGAGGGTCGCGAAAGCTCATTGAAATTCATTAAAACCC
TCCGCTTCCGCCTTTTCCTCTTTATCGCCCCCCCCCTCCCTCTCCCCCTTTTTCTTAATCCGAA
TTTAACAACGAAGCACATAATAGGTTAGGCGTCGATAATTTTTACGATCCAACTTGAAAAT
AAATACTTTCCTCCGCGAATAATAAATGTAATTTTATTATTCGAACGATCGAACGTTTAATT
TATTGTACGTGGTTTTCTCGCTTTTGCGCGCGTTCAGGTTTGCCCGCCCTTTTTCCAAGAAAT
GCGGGATTCGAGTCCAGGCTGTATTTATCGCCGATGAAGCGTAGATGAACGAAACGGCCA
ATGTACCGTGCCTAATCGGACTCGTATATTATTGGAAAATCACGAACCGTTACGAGCGAGG
GAGTAAAAATGTAGCTTCTTCCGTCGACAAATATATCCTGTAATAAATATTATTGCTTAATT
TAACTCGAAACGATGAAATAACTCGACTTTTAACGATCAGTTGTATTATTGAATAGAAAAA
AAAAGAAAACTCTCGATATCGATTATAAATGTTATTTCTATATCAATATTTCTCTGATAAAT
ATTGTAATAAATTCTCGCTTTAGATTTATTCAATTTTTAACGATTGTCCGATC

301. >MSTRG.6173.1 Gene=MSTRG.6173
GTTTTACAACAGTTATCTTAAAGTCTATAAATCATGGTTATTAGTACTCGTAATAATGAGAA
CTCTTAATGGATTCAATGCATCGTATTGAAGATTTTGGAAATTTGCCAAAAGCAATTGATAA
TAATGTTCTTTCATGCAACAGATATGTGAATATATGCACATTTTCAAGGAACAATCGATACA
AGTATTTAATTTCAATAATTTCATTAAAAGTATAATCGAACACTTCCTGAAAAGGGGGATTT
ATTAATGTTAATCAATTATAATTTCCGCGTGTATAAATGACTATCAAGGTCTCGCGCCATTA
TCATCGATTTAAAATATCAAAGTATTTATAAATGCGCATGCTCGGACTCAAATGGCGCGGC
AGAACACGTACAAGAGTGATCGAGACAGAATATTAGCGGGACTAGCGAAGCTGAGAAGG
ACATCGAAGTAAAATGGCTGCAGCAAGTGCCAACATATACTTACACTTGTACTTTTCACGT
ATCTTCTGGTAATTTTCGAGCATGATCTCGCGTGATATTTGACCACGATTAAGGGCACGGTC
ACCAATGGAACGCATGGACACAACATGGCTGCTAGACGCGCGAAGCAACCAAGACGACTT
CGCTTCGATACTGAAGCACGATAAGTGCTACGCACGCCACGTATGACCGACTGTCACTGTC
TGCTGTGCTCGCTCGAGGGGATGAAGGGACTGTGTATGGTTCTGGTTCAATCATGTTTCACG
CAATGTCGCCGATACATACGATCAACTGTCAAGTGGGGAAAGCGGTAATACCTAAAAGAC
GGAAATAGATATAAATTGTCCATCTGCCGGGACGTTTTGAAAAGAAAGAAATGATCCGCA
CGGGAGCGAGGCGAAGCCTTATCTCGAACCGAAGTGTCACGTTACAAAACAGAAACATTG
GGGATTCGACGATGTTTACCATGGAACGGCAACACACCACGAATCGGCGTGGAATCGAAT
GAGATAAAAGTCGAATGGGTGTTTCGTCCGGACGACATGGACGCCTCGTCTTCGACTCGTTT
CTTGTTGTCACACACGGTCCCGAGCCGTGTACGCTCACGGATACACACACACACGCACACA
CGGTCCCGAGCCGTGTACGCTCACGGATACACACACACACACGCACACACGGTCCCGAGC

CGTGTACGCTCACGGATACACACACACGGTTGGGCCGGTATACGTGGCAGGCAACACACG
TATAGGAAGGCGAGGAACGCAGATTGCTAGAGGCGCTGTTCCGCTGACCTACTCTACGATT
ATCACTCACTATAGGCCGATCGCCGGTGCAGGTGCGTGTGCGTTAACTAGCGTTGCCAACT
ATACCTCCTCGAGGAAAGGAGGACGCGGTGCGGGGACGTAATCAAATAAACATTTTCGGT
CCTTTTACCGATTTCTTCTCCTCCAACCCGATCTCTTTTCATCCAAATGTAATTTCACATCCCA
ACGAATATTTATCTCACGCTTTTTTACTCCTCAGAATTGCGATTATTGGACGATAATATTAAT
CTTTCTCTTGACGAGTTATATTGAAAGATTTTTCTTTGAAAAATTCATTATCTCGTGAAATTT
CTACCGTGATACGCGTGTAATACGTCCATATTTCATCCCGTCAATATTTTCGATCGTTACATC
GATCGATTGCAACGAGTTTATCCATTTATCAGCGTAAATGGACGTACCTCGATGCTTCGAAC
AACGTCTAATTTTCATCTTTCGATTGGACGGACGCAACGAGGACAGTGTAGCCATTGATTGA
ATCTAATCAATGATCGTTAGTAGTCTCAATGGTCTTACATTATTTACAGGCGGTATATAAAG
TGTATTCACGAATAACTATTCTCGATATTATTCATAGATTTTCTTGGGCAATAATTTGTAACT
AATAATAATCTAAATATCGATATAATATATATCGATTTTATCTCTTTTAAATTTTTTAAATTT
CAAAGTAATTAAATTATTTCGTAAAACAATCAGGAATATTTATTTTGAATAATATGAATCTT
TTTAAATTATAAAAAAAAAATAAATGCATACATATATATATATATGTTTCAGTATTCCTTGGA
AAGATTAACCCAAATTGCCAGAAGAGAAATGATTAATCGAGCGAAATTTTATATATATACG
GAAGAGAACGAATACGGGAAAGAATATAGAAAAATAACGAATCAATAAGGAATCGATTA
TATTTTACTTTATGCACGTATCCGAGCGTTCTAAAATTCTATTCCAACTCGAAGCGCATAGA
GGAGAAGCGAGGAGAGCAGAGACATCTATCTCAACATCCGTATTCTTTCTATCCGCGCTCA
TTTTTATCGTCGATCTCCTTCACGGCTCATCATCGGATTAACAGTTAATAATAAGAGTGTGC
GCGAAGGTCGATGATTTCTGGCAAGTATCCGCGGCGAAAGTATGGCAGATCAATTTCGCGA
TGCGTGACCGACTCGAAAGCGAGCATGTTTTCTTCCCCCGATCTTTGCGCTTCTTTAATCTGG
TCTATCCTCCTTTTTTCCCCCCACTTTTTCTCTCTTCCTTTTTTTGCCCATGCGCGCCAACGTCT
ACCAATGTTTTTCTCCAGCCTCGCCTCTGGACATACTATTCCGGCTTACTATACTACTCCGTT
TCCATCTCTGAACAGAGCTGACAGAGGCGGTTCTGAAAACTTTCTAGCTGGCAAAAAGCGT
TCGTCCCATTCGAATGCTCGTTGAAAATTCGGCAGTGAGTTTTTTTGCACCAAGTTGCGGTG
TAACGAACAGCTCGGTTGCACGCGGTAGACGGTGTAAAAGGTTTGCTTCTCTTTTTTTTTTTT
TTTTGGAGAAGAGAATTTATCGAATCAAGGTGACCTCCTTTTCGTTATGACACACCGTTCTG
TTGTCGTCTATTTTCAGCGTTCTTTCGTTTCATTTAGATACGTAAGAGAGTTTTTAAATTTATT
ACGAACAACTAGGAGTATAAATATTCGTGTTATTTCTAAAAACGTTATCGAAAACGTTAGC
TATTCCTAAGCTCGAGCAAACTCTAATTATAGTTCATAGTTCATGATACATTTAGCTTGTTAT
TAACGTTATAATTCCGCAATCGTGTTTATAAATAAGGGAGGGGGGAGGGGGAAAAAAGAA
AAAGAAGAAGCGTAAACTTCTGAAAGATAAAACTCTTCCAATATTATTTTATGACGTCTCC
GCGAACGAAACGGAGAACGATAAAATTTAATACACACAAGTTATACAAGTTCTACATTAC
GTTAGATAATAGATATTGTTATACATGTAGATAAAATTACGCCATTAAGGAAACCTGGTTGT
GCGACGTGAACGTGTTACACACGTAGAGTAGTGTATTTTCAAACGTGCGCCTATATAAACG
TGTTTTCGAGCATATTTTCAAACATATGTTTCATAATGCATCACGACTTTTTTATGTCGCGTC
ATCATATTTTCTCCCAACCCAGTGTTCTCTACTGCTAGAATTACCTCTCTGCAATATCTCACC
GCGTGTAAAATAAAATATTACGATGACCAACATTGTGTGTGCGGCCAACGCAATATCGAAT
ACTTGCCGTCTAAATCGTTTTCGCGAAAAAATACGAGAGAGAGAGAGAAGTGGACAATGA
TTCGATAAATAATTCCAAGTAACTTCATCGGTTTGGAAAGAGCGCGCGTTGTTATTTAATCC
GCTGGCGTTGGTCGCGCAGAAAATAGACCGTGTCGACGAGCGCGCTATTAATAGACCGTTT
TTTTGGACAAGCGTCGTAAAGATAACGCCGAGCCGTTGCCAAATTACAAGCAGCGCGGTTT

CTCGCGAAACAAAATTGTTCGATTCCAGGGCACGAAGGCCGGCTAAAATGCTTGGCACGC
GGATCCTCACCGCGGGTATGAATGATAAAATATTAAAATAGAAACGTGTCGAGCGGGGAA
CGCTAGTTGCTGTGACGGTGTTTTCCAGATTGAAAAGCAACCTTGACCCACTACTGGGAGG
AGGCCACGTGGAGTTGTGCTATCGGGAAACCCGTACTTCGCGGTGTATACCTTTTCGGAATT
ATCGATTTCCTTTTTTTATTATTCGATTTGAGCGAGCGACAATAAGGGACCGGCATTCTTGCG
CTCTGCATAACGACCAACGGAATGATGTAATCCGATCCTCTTATTATGAATTCCGCTCATCG
CTACGCTTTTTTCTTTCCCCCCGTAAAGCGATGTTTATAAAATATCGACCCAAAATTTTCTTA
TTCGAATTTTGCAAAATAATCGACGTCTCATTTTATCCTCTTTTTTTGCATATCTAAAAAACC
TCGTTGAATAAATCATAGAATAATCCTGGCTCGCTAATGGAATTGGACGGAGAGTTTGGAG
TGGAAAGGAAGGAGAAAGAAGAGGCGTGAATTCCAACGTGTGTCCAAGAACGACGGTGA
CCCCGGTATAATAGGGGAAAGGGAAAGAGATACCTGTATATATAGAAATAGCAGGATCAT
CAATAGGTCACTTGTTCTCGAGTCATCGGACGATCGATCGCCGTTTGGCAAGATCGCCGGCT
GTTACCTAGATTTTTCGCCCGTGATTCCAAGAAACGATTCCCTCCCCCCCCCCCTCCTCGCC
CGTTGTGCCGGTAAACGGATCACTAAGGCGACAGCCGTCGTTTTATCTCCCGTCCCGGGAG
GAGGTAGCCAGTGTCAATCGAAACGGAGTCGAATCGGGCGATATACCTCTGGCTGAAGGA
TGGAGAGGCGCGCAACCGGCGCGCAGCAGCGTGGCTCGGGAGTATGGACCTTGCGAGCGG
TCAAAGTCGTGGCCTACTTCACTTCCTGCCTTCGAAACTTTCTCCTCGTGGCGTCGTTCGTGC
GTTCACGTTCGAGTGCCAAGGCCTAGCGATGGGATCAAGTTCAGCGCTCGGTGTATAAATA
TAATTCCCTATCGATCCCTATCTCTTCCCAAATTAAGCAACGGAACGGAATCCGTCTCTCTC
CATAAATTTTACTCGATACTTTTTACTCGATATTTTTGGAGAATCGGATTTTCGGAAAAGCG
GCAAATTCGAAATGCACTCGCTCGATCCCATCGCTATTGCCAACCTTCCATCTCCTTCCATC
TCTTCCTTCCTCGCAATATGGATGATTGCGCCAATTATGAGAGAGACCCGTCGGATCTCGAG
AAATCATTTGGATGTCCGGTTCCAGTCGATCGATTTATTTTGTTTCGAAGAGTATTCGGAAA
AATTCATTGGAACGATTTTAATATATTTTTTTCCCAAAGGATATAAATAATTCAAAATTTTTT
TTTATTATTATAATTTTTATCGTTACGTTTAAAGTAAATTTTTTTTTAGAGATTCGAGAGAGAT
CGTTTGATTTTGAAAGCGATAAATTTCATTTACCGATAAACGTTGCTCGAAGCAAAATCGA
ACGATTCTTCTCAATTCTTCTTCGTATGCGCGTAGGATGCGCGGCTAAAAAGTATTATTTGG
GACGTTCCATTGGCCGAGTATCCGTCGCATCCGAGTATGGGCCACGATCCATAAACCGTGT
ATACGAACCGTGCACTGAACGCGTTATATTTACGTTTCGTTTAAAAAAATTCTGAACCGCGT
CTAAAACTCGCCCCCTCTTTGTCAATCCCACTATCTATCTCCACAATTAAGCGTACGTTCATC
GAGGTAAGGAAAAAAGGAGAAGAGAAGTAATCGAAATGAAATTCGGCCTAAAAATATTTT
AAAAAAATATATTTTCCAATTGACAATTTGGTTTTTGAATGGTTTCGTTATCGAAAAACCGA
GAGAGGAGAAAATAATAGATTAATAATAAAAGGTAATAATAATATTTCCTTCATCTCGAGT
CTACTTTCGTTTATACGCTCCTTTTACATTTGCTCGACTTTCTCTCGCTCGATAAACGGTGGTA
TTCATTTCCAAAATTATTCCTTTTCCAGAGAAGAAAGAAGATTTTCCGTATTCCCTTCTCGCC
TGTTTTGCTCGCCCAGCTATCTATATCGAAATTCATTTTCGTATCCCGCTCGCCGCGATCGCC
ATGTTCTGATTACTCGAGGAATCAGAGCGTCCTCTATAGACGGCCGCAGTAGATTAAATTTT
AGCAAGTCACCAGTACACGATTCATCGCCGTGTTTCTCGTAATTAGCCACCGGTGAATGGA
TCCACGGTATCCGGAATATTTTTCCTCGAACAGAAGAAGAAGAAGAATAAGCGCAAGCGG
TGGTTCGAATAACGTGTATTAAAAAAAAAATTCATTTTCAAACAGGTGTGTAACAGGTATAC
GTTAAAATTTCAAATCCTCTTTTTTGAACGTTTACGGATTAAACGTTAAATATATGGGCAGC
CGAGTGGATCGATAATTGGTCGGTAACGTTTCCAATGGTAATGTCGAGAAATGTAAATTTCT
GTAAATTTTTTTAAAAAGCGTTTTAATTCCAATTTTATACAACATTTATCTTAATTTAAATCG

TTATGAATTCGATCGATAAGTTTCTTTTTGTTTTAAATTAAAAAAAAAAAAAATTAAGTAATT
AATAATAATAGGAAATTCGCGCGAATTTTCAACACGTCCCACATGCGTTCCACGCGATAAG
AGTCGAAGAGTATGAAAAGTGGAAACTTTCTACTCTTTTTTTTTTTTTCTTTTTCCTTTTTTAA
ACTTTTATATTAATTTTAACGTTCACGCGCGTGATATATACTCGCCA

302. >MSTRG.6173.2 Gene=MSTRG.6173
GCAGAACACGTACAAGAGTGATCGAGACAGAATATTAGCGGGACTAGCGAAGCTGAGAA
GGACATCGAAGTAAAATGGCTGCAGCAAGTGCCAACATATACTTACACTTGTACTTTTCAC
GTATCTTCTGGTAATTTTCGAGCATGATCTCGCGTGATATTTGACCACGATTAAGGGCACGG
TCACCAATGGAACGCATGGACACAACATGGCTGCTAGACGCGCGAAGCAACCAAGACGA
CTTCGCTTCGATACTGAAGCACGATAAGTGCTACGCACGCCACGTATGACCGACTGTCACT
GTCTGCTGTGCTCGCTCGAGGGGATGAAGGGACTGTGTATGGTTCTGGTTCAATCATGTTTC
ACGCAATGTCGCCGATACATACGATCAACTGTCAAGTGGGGAAAGCGGTAATACCTAAAA
GACGGAAATAGATATAAATTGTCCATCTGCCGGGACGTTTTGAAAAGAAAGAAATGATCC
GCACGGGAGCGAGGCGAAGCCTTATCTCGAACCGAAGTGTCACGTTACAAAACAGAAACA
TTGGGGATTCGACGATGTTTACCATGGAACGGCAACACACCACGAATCGGCGTGGAATCG
AATGAGATAAAAGTCGAATGGGTGTTTCGTCCGGACGACATGGACGCCTCGTCTTCGACTC
GTTTCTTGTTGTCACACACGGTCCCGAGCCGTGTACGCTCACGGATACACACACACACGCA
CACACGGTCCCGAGCCGTGTACGCTCACGGATACACACACACACACGCACACACGGTCCC
GAGCCGTGTACGCTCACGGATACACACACACGGTTGGGCCGGTATACGTGGCAGGCAACA
CACGTATAGGAAGGCGAGGAACGCAGATTGCTAGAGGCGCTGTTCCGCTGACCTACTCTAC
GATTATCACTCACTATAGGCCGATCGCCGGTGCAGTATTCCTTGGAAAGATTAACCCAAATT
GCCAGAAGAGAAATGATTAATCGAGCGAAATTTTATATATATACGGAAGAGAACGAATAC
GGGAAAGAATATAGAAAAATAACGAATCAATAAGGAATCGATTATATTTTACTTTATGCAC
GTATCCGAGCGTTCTAAAATTCTATTCCAACTCGAAGCGCATAGAGGAGAAGCGAGGAGA
GCAGAGACATCTATCTCAACATCCGTATTCTTTCTATCCGCGCTCATTTTTATCGTCGATCTC
CTTCACGGCTCATCATCGGATTAACAGTTAATAATAAGAGTGTGCGCGAAGGTCGATGATT
TCTGGCAAGTATCCGCGGCGAAAGTATGGCAGATCAATTTCGCGATGCGTGACCGACTCGA
AAGCGAGCATGTTTTCTTCCCCCGATCTTTGCGCTTCTTTAATCTGGTCTATCCTCCTTTTTTC
CCCCCACTTTTTCTCTCTTCCTTTTTTTGCCCATGCGCGCCAACGTCTACCAATGTTTTTCTCC
AGCCTCGCCTCTGGACATACTATTCCGGCTTACTATACTACTCCGTTTCCATCTCTGAACAG
AGCTGACAGAGGCGGTTCTGAAAACTTTCTAGCTGGCAAAAAGCGTTCGTCCCATTCGAAT
GCTCGTTGAAAATTCGGCAGTGAGTTTTTTTGCACCAAGTTGCGGTGTAACGAACAGCTCGG
TTGCACGCGGTAGACGGTGTAAAAGGTTTGCTTCTCTTTTTTTTTTTTTTTTGGAGAAGAGAA
TTTATCGAATCAAGGTGACCTCCTTTTCGTTATGACACACCGTTCTGTTGTCGTCTATTTTCA
GCGTTCTTTCGTTTCATTTAGATACGTAAGAGAGTTTTTAAATTTATTACGAACAACTAGGA
GTATAAATATTCGTGTTATTTCTAAAAACGTTATCGAAAACGTTAGCTATTCCTAAGCTCGA
GCAAACTCTAATTATAGTTCATAGTTCATGATACATTTAGCTTGTTATTAACGTTATAATTCC
GCAATCGTGTTTATAAATAAGGGAGGGGGGAGGGGGAAAAAAGAAAAAGAAGAAGCGTA
AACTTCTGAAAGATAAAACTCTTCCAATATTATTTTATGACGTCTCCGCGAACGAAACGGA
GAACGATAAAATTTAATACACACAAGTTATACAAGTTCTACATTACGTTAGATAATAGATA
TTGTTATACATGTAGATAAAATTACGCCATTAAGGAAACCTGGTTGTGCGACGTGAACGTG
TTACACACGTAGAGTAGTGTATTTTCAAACGTGCGCCTATATAAACGTGTTTTCGAGCATAT

TTTCAAACATATGTTTCATAATGCATCACGACTTTTTATGTCGCGTCATCATATTTTCTCCCA
ACCCAGTGTTCTCTACTGCTAGAATTACCTCTCTGCAATATCTCACCGCGTGTAAAATAAAA
TATTACGATGACCAACATTGTGTGTGCGGCCAACGCAATATCGAATACTTGCCGTCTAAATC
GTTTTCGCGAAAAAAATACGAGAGAGAGAGAGAAGTGGACAATGATTCGATAAATAATTCC
AAGTAACTTCATCGGTTTGGAAAGAGCGCGCGTTGTTATTTAATCCGCTGGCGTTGGTCGCG
CAGAAAATAGACCGTGTCGACGAGCGCGCTATTAATAGACCGTTTTTTTGGACAAGCGTCG
TAAAGATAACGCCGAGCCGTTGCCAAATTACAAGCAGCGCGGTTTCTCGCGAAACAAAAT
TGTTCGATTCCAGGGCACGAAGGCCGGCTAAAATGCTTGGCACGCGGATCCTCACCGCGGG
TATGAATGATAAAATATTAAAATAGAAACGTGTCGAGCGGGGAACGCTAGTTGCTGTGAC
GGTGTTTTCCAGATTGAAAAGCAACCTTGACCCACTACTGGGAGGAGGCCACGTGGAGTTG
TGCTATCGGGAAACCCGTACTTCGCGGTGTATACCTTTTCGGAATTATCGATTTCCTTTTTTT
ATTATTCGATTTGAGCGAGCGACAATAAGGGACCGGCATTCTTGCGCTCTGCATAACGACC
AACGGAATGATGTAATCCGATCCTCTTATTATGAATTCCGCTCATCGCTACGCTTTTTTCTTT
CCCCCCGTAAAGCGATGTTTATAAAATATCGACCCAAAATTTTCTTATTCGAATTTTGCAAA
ATAATCGACGTCTCATTTTATCCTCTTTTTTTGCATATCTAAAAAACCTCGTTGAATAAATCA
TAGAATAATCCTGGCTCGCTAATGGAATTGGACGGAGAGTTTGGAGTGGAAAGGAAGGAG
AAAGAAGAGGCGTGAATTCCAACGTGTGTCCAAGAACGACGGTGACCCCGGTATAATAGG
GGAAAGGGAAAGAGATACCTGTATATATAGAAATAGCAGGATCATCAATAGGTCACTTGT
TCTCGAGTCATCGGACGATCGATCGCCGTTTGGCAAGATCGCCGGCTGTTACCTAGATTTTT
CGCCCGTGATTCCAAGAAACGATTCCCTCCCCCCCCCCCTCCTCGCCCGTTGTGCCGGTAAA
CGGATCACTAAGGCGACAGCCGTCGTTTTATCTCCCGTCCCGGGAGGAGGTAGCCAGTGTC
AATCGAAACGGAGTCGAATCGGGCGATATACCTCTGGCTGAAGGATGGAGAGGCGCGCAA
CCGGCGCGCAGCAGCGTGGCTCGGGAGTATGGACCTTGCGAGCGGTCAAAGTCGTGGCCT
ACTTCACTTCCTGCCTTCGAAACTTTCTCCTCGTGGCGTCGTTCGTGCGTTCACGTTCGAGTG
CCAAGGCCTAGCGATGGGATCAAGTTCAGCGCTCGGTGTATAAATATAATTCCCTATCGAT
CCCTATCTCTTCCCAAATTAAGCAACGGAACGGAATCCGTCTCTCTCCATAAATTTTACTCG
ATACTTTTTACTCGATATTTTTGGAGAATCGGATTTTCGGAAAAGCGGCAAATTCGAAATGC
ACTCGCTCGATCCCATCGCTATTGCCAACCTTCCATCTCCTTCCATCTCTTCCTTCCTCGCAA
TATGGATGATTGCGCCAATTATGAGAGAGACCCGTCGGATCTCGAGAAATCATTTGGATGT
CCGGTTCCAGTCGATCGATTTATTTTGTTTCGAAGAGTATTCGGAAAAATTCATTGGAACGA
TTTTAATATATTTTTTTCCCAAA

303. >MSTRG.6206.3 Gene=MSTRG.6206
TTGTCATTGGATTTTTACGTGACAAAAGTGAGTTTTCGTAGAAAATTTGACAACGAATCGCG
TGAAAATGCCGCCGAAAAAGCGGGAAAAAGAAGTGGATGGACAAAAAGGTCCTCGAATG
GATCAAATACAAGCAGACCATGAGCTCTTCCTACAGGCCTTCGAAAAGCCAACCCAAATTT
ATCGATTTCTTCGCACAAGAAACCAAATTTCAAAACAAGATTCAAGAG

304. >MSTRG.6223.1 Gene=MSTRG.6223
GAAAGATCTGCACACTCGAGGTCTGTATCTTCGACACTTGCATAAAACGTAAAAAAAGAAT
ACGAGATGAAGGTAGTGATATTTATCTTCGCACTACTCGCCACGATATGCGCAGCATTCGC
GTATGTACCATTACCAAATGTACCACAACCAGGACGTCGTCCGTTTCCAACGTTTCCTGGTC
AGGGACCATTCAATCCGAAAATTAAATGGCCTCAAGGCTATTAATCGTCCAATGGACAAAT

CAATGGAATATACTTATACGGTTCACGTGATAAATCTACTGTGGTGAAACAATCGATAATT
GATGGGGTATTATAATATAATTATGAATTGAAATAAATCGTATAAGTATTTATCGA

305. >MSTRG.6244.1 Gene=MSTRG.6244
GCCTGATCCTGAGCAAGTCGCAGAGATGCCTGTAGTGGCCCAGAATTGATTTCAATGTGGT
CGCGTTGCAAAACAGATCGAACTGCTCGTTAGCTTTCGCTATCTCCGAGCTCATCGTTTGCT
TCCGATGCTCCATTTTCTTTCTTCCTGTCCTTCCTGCTTTCGAGTCAGTTTGTCATTACATGTG
TATGTACACGAAGGGCCGCTCTGCGTGCACTACCAATCAGACGTAGAGTCACTGCACTTTTT
TCTTAGCTTTTTTACTTTTTATCCACATTACCGCGCGTGTTTACAATCCGATTATGCACCATAT
CACGTCGATTTATCACCATTCACATTATACCGTTGTATCGGAGAACATCGCGCGATTCATTG
TCCACCCGACATTTGCTCTTGTGCTCCGCTTCGACTGGCCTCCTTCGATTGCGCGTCAAACAC
TGTACTGCGACCAAACCAG

306. >MSTRG.6246.2 Gene=MSTRG.6246
CTTAATAGTCTGCAAATTGCAGGTTGCAGTGTTCCATTTTATTGTGTGTGAGAAGTGTTTAAA
CACGTTGAATAATTATATATATAGCGCAATTTTTTTATATCTTATTATAATATTGTGATTTGA
AATTCATATTAGTTAAAATGTCGCGTATAACTATTTTATTATTACTTGCTTATTTCAATGTGG
GATTATCTTATGGAATGGAATGTTCATTTGGAAACAAGAACATTATATCGAAAGTGATCGA
TTCATGTCCAGGAATATTAGATTCGAGCGATAAATCTTATTGTTGTTATGATTTTGAAAAGA
ATGAAATGTATTGTTGTGATGCAGTGGAATTTGCATCGAAATCATCATGGATTCTTCTCACA
GTGATTTGTGCGGTTGCAGTCGTGTTTTCTGTGATAATATTTTGCATATCGTGTTTGTGCTGCA
GTTGTTGTCCATGGTATCGCAGACGTCATCAAGGAACGGTCTATGGAAGACCACCTATAAT
ATGAATCCATTTCATCACATCTTCCAATCTCCTGACAGAGTATATAATGTTTCCTATAATTGT
CAAAGTATATGTATTTTTTTTAAATATTTTTTACTTCGCTTCCTATATATTCTAAACGCATCAA
AAATTTTGTGCTTACGATTTAATTACGAATAACGTATATAAACA

307. >MSTRG.6266.1 Gene=MSTRG.6266
GAGGTGACTCGCTTTCTCGCTCGGCCCCGCTATCCTTCTGTCTTCCTCTTTCCTGCTCGAACT
CGTGACTCGTCGGTGTTCGGCCGGTGCCTCTCGCGGCACCTCTCGCTTTCACTCGTCCGTGTG
AAGAGGCTATCGCGGGCCCTGAGAGCTCAGTATCCGCCGGACCGTCGCGTGGTTCGATGTC
GTGCGCGCGTCCACGACCGGTGCCTCGTCTCGGGATAGAAAGAAAGTGTTTTTTTTTTTTTT
TTTCCGCCCTTTTTCCCTCCCCCCCTATTTCTCTCGAGTTTTGCACCGAGTTTTGTTCCGTTGTT
GTTTCGCAGCACCGCTCGAGGGTATCGTGTTTCACTGTTTTCGGGCCGGCCACATCTCTCTG
CGGGCACACGCGTGACCTTTGTAAAAATAGTGTTTCGTTCCTTCCTTTCGACAGTAGATAGA
GAAGGAGAAAGATCTCTGGCCTAGGGTTTCTCTCGCGTTGCATCGAACATGGGGAGACTCG
ACTCGTTCAGATGCAGTTTGGGATTACTGTCGATCGTAACTTTTACGGCGCTAGTAGCGATC
GCCTCGACGACGACAGTCCATCAATTCGATATTTCAGATTATCTGTACGGGATAGATCACG
ATTTACAGGCAAGAGCACGTGCAGGGATCGAGGCCGAAAGATTTACCTATCAGACGAAGG
CTAGGTG

308. >MSTRG.6347.1 Gene=MSTRG.6347
GTCAAATTTACATTTATATAAAAATTATACTACAAATATACTATTCTATCTTTGAAACTTATT
ATTTTTATTTATAAATGAAATACATAAATTTTAACAAGTATTTCAGTCAATCAATAAATAAA

TTACATATAATAATTTTTGAACATTCTTGAATATTATAAATTTTAACCTCTACAAAACGACAT
TTCTCAAAAGCGTCGATGAATGTAATGTCATGAATTGTTATACGAATATGCAATGTAATAAT
AGTGTTTTGCAAATTAATGTGGCAAATACAAGGTTAGATTGGAATATGACATTTGTTCTGAA
ACTATTATAATTTTTATCTTCTTTATTTTTACAATAAATCCATTGAGAAATTCAAGAGGAATA
TCAATATCAAAAGTTATAACATAATTTTGTATGATTAAAACAATTATGAAAACAATGGCAT
CCGAGAATTTTTTTGGAAGTGGTGGCACATTTCATCGTGCTTTGATGTTTATGCTCAAACTTG
GTTGGCAATCACGGTTTCGTCTCCCAATTCTACTAGTTATTGTACTTGTCGCTATGGATTATA
GACTTCAATTAGAAATTGAATTTGAAGTTAATCCCGAGCAACATAATCATTTGTGATTTAAG
GAAAATTATTTATAAGTTAATTCTAGATTATATCATTGAAGAAAATTTTAAATCATCATAGA
AAGTGC

309. >MSTRG.6349.3 Gene=MSTRG.6349
TTATTCTTTTGTCGAAAAATATTCTTAATTCGTATCCATGTTATTTAGCTAAAATTGCTTTAAA
TTCGAAATAAAAGTAATTAATTAGTTTCGAAAAATTGAGATTAAGTTATGAGGCTAATCTTG
TTGATTGTTCTTCGTAAGGCTTCGTAAAATGATGATTTCATCGGATATCGAATCAAGGATTA
TTTTTCGAATTTTGACTTAGAGGAAACAAACACTGCCAGCGCAGGCGGAAATTCTTGCGTTC
TCTCTTTCACGACTGAATGATACGCACAGAAATTAATTTTCTCACGATTCGTCGTTTCCTCTT
GAATATCGTTGCAATATCGAAGAAAAATATATTCGCGAGAAGAATCAAAGAAATAATCTC
GAGATGATCGGTGCCAGTACAAAAAAAAAAAAAAAAAAAAGAAAAAAAAAAGAAACGTCA
GCATATATTATTTTTTCCCCCTTTTTACAATGGAACAATTTGCGCCACGCGAACTATGTTCGA
TTAAATTGAAATGGAAACGTGATAAGAAAATTTTGAACGAGAGAACGATGAAAATCCAAA
GGAGGTCGTTGACGTAAATTGAGTTAAAAAACAAAAGCTAGTTAATAAAATTGGCTAGTA
AAATAAGAAAGAAAAACGAAAAAAAAAAAAAAAAAGAAACGATAAAGAGAAAAAATCG
CGCGCGCTTGATTTCGCTCGGCCTAGTGGAGCTTCTTTTTCTTCTTCTTTGTCTTCTTCTTCTTA
TTCTATTTCTTCTCGAGTCTGTAAGGAGCCTATAGCAATTCGTATGTGAGACTTCTTAAAAA
CGGAGAAGAACTGCGGAAAGAAAAGGAATGAAAAAAAGGAAAGAAAAGAAAAGCAAG
AGCAAAAGTAAAGAACATGCGAAGTAGGGAAGAGTTTACTCGAAGACATTTACGACGAGG
CGTCACGACGATCACGGCATTGAGGTATATATATATATATATATATATATATATATATATAT
AAATTCGCGATCAATCATATTTTAACTATAATCGAAATTGTATCGATTCGCGAATGGTTTGT
ACATGTATATGTATATATATTTATACGCGTATATACATATATGATCCATTCGAAACAAAAAT
AACTATGAGATATAAATGACGTACGTGTGCGCATGCGTGTATGATTTGCGGTGAGTGACGG
TGAATTCTTTTCATGTAAGTATGATTTTACTTATTCGAACTGTACTACTACTACTACATAGTG
CGTGATGCAACGAGTATGTGAGTGATATTGCGAAAGGAAGAATGGGGAAAAATAATTAGA
TGAAATGTCCGAAGAAGCAAAGCAAATATCGACAAAGAGTTGATGAAAAGCACGAGCGG
CTAACTGATCGACTAAACGTCTCTAGGCTGCCCACGGATTTAATCGTCGAGTTTAATCCGGT
GGCGAGAGTTTGAGAGGGAGAAAACGCCTAGCCGGAACGGCGAGCGTATACGAGAAATG
TCGAACTGTCATTTCAGTTGTGTCTCTCCCGAGGAGCCCGATCCCATTTTCTCCTTGGAATCG
ATGTAGAAAAACCGAATACAATAACACATATAACACTGACGCTTTCGCCACACCATTTCTT
TCTTAGTTTCTCGTCGTGTATGATATTTGTGCGTGGTGAATGATCTTCGAGGATGGCAAGAG
CGAATAGGTCGAAAAAAAAAAAAAAAAAATCAGAAAAATAAAACCTGTGCGCTTGATTATAT
CGATCGATAATCCTGCATTTTGAAAGGGGAAAAATGCAAAAAAAAAAATTTATCGACGAGA
TATTATATTATTATTGCGAATGTGAGAGTAACGACGAGAAAAAGAAAAAGAAAAAAAAACG
TATAAATTTGCGAATATTTAACCTTAAATCGCCGCGGATTGTCGACCCATTAAAGAGCTGTT

GTCCTTCTTCTTCTGAACGAATAATAAAATAGAACATTTTTCAGTGGAAAAATTGTCAAATC
CGAGGACGGATTACTTTGGTGGGTCGATCAGAAATTAAGGAAATTAAGCTTGCATTATTCG
AAATTCTTGATGTGAGTTCGTTAACTCCTAAACTGAAGTGCAATTGTAAAAAAAAAAAAAGG
AAAAAAAAAAAAAAGGAAAAAAAAATGACCGTCGTTGTCCGTTTGCTTGTTCGTCTATCAAAT
GTATGTAATTGACGACGATGCGATGAATGTTGCGTGAGATGTTTCGAGTTTTTTTTTTTTTTC
CGTTGTCTTCGTACTCTTATTCTTTTTCCTCTATTTTTCTCCGTTTCTTCTCTCTCTTTTTCTTTTT
TTTTTTTTCTTTTGATAGCCTAATGCATGTCTTGAAGTTAATCCTTTATGATGAGTGATGGAA
ACAAGGGCCATAAGAACAGTGCGTACACAAAATGACATTCTCCGAACATAATAATAATAA
TAATAATAATAATAATAATAATAATCAAAAATAAGGCAGCTATTTAATAACCGCCGTA
CTCTTTGGAGATTCCCGAGTAGCGTGAATAACACGAAGGTGCATTGAGGATGAAACAAGC
GTTTCTACGTGTTAATCGATTTTAATACTTGTACTCTATCGAATCGTAGAATAGTGATTCTTA
ATCTGCGAACGTGTAATCATGTATTTTTTGAAATTTACGAAAGACACTTGATATGGTCTATA
ATTAATTTTTTATATAATACAAAAGAGAAGGCTATATAAAAAAACTCTTAAGGAAAGTGAG
AAAAGTGAAACTAAGGCGATCGTCCGTTTTACTTGTTAATTTTTTTTTTGCTTTCTTTTTTCTT
ATTCCTTTTTTTTTTTATTTTCATTTTATTTAGTCAATCTTTTTTCCCCCGTTTGATCGAGTGGCT
GCGAAACAATTTTTGCGAAAACGATTAAGAATTATTATTAATGGCATAAAGGGTTAATTTT
GGAAATGGAAATGCTTCGCTCGTAACGTTGATGCGCGAAAGGAGATGTGAATCGAATCAG
ATGCAACAATGTCTGAATCAGTTTGAAACCAGTTTCTTACCTGTGCAGGCAGTTGTTTAATT
TCGCGTTTTTCTCCGCCACTTTATGTTCACTGTAGGGATTCAAATAATTATTGAATTAGAAAG
AGAGCCCAAAAAAAAAAAACGAAATGTTTGTTTTAAACCGGAAATGGCGGCGATGGGGGG
GACTTCGATGAAACAATGACACGTCTCGTCTTTAAATATTTTCGGGAAATAAACGAGTTTTC
TTTCGACGGCGAGACGTTCATTGTTTCAACGCCGTTCCCTCACCGTTTGCTCACCGAATTCTA
GTTAAATAATGAAAAAAGAAGAAACGAAGC
GTTTTTTAAATTAAACGAGATAAACGA

310. >MSTRG.6380.1 Gene=MSTRG.6380
CACCGCCACGACTTAGAGCCTTGTTTAATAGTGTGTAGGCTATCTTGTGAGAACGACTTAGA
AGTTTTCCATAGGTCAATGTTACGCAAAGCTTGCCATTTGGATCAAGAACGGTCGCCACTG
GTGCTTTGTATGATGCTGAACCATACCTCTGTATGGCGACCTCGAGTGACCTCGGCAGGCCC
GCGGCGACCGATACTGGTTCACCGACCGCGGAGGTCATAGAACCGCCCTCGGGTTTCGGGG
CGTTCGGGTCTTTGGGATTGGCCGCGATTTCGAGCTCGATGTCGTCGTCCTCGTAGAATTCG
GGCAATGGGCGACGTTTCGGCCGTTTCAATGTATTGAGCAATTGTTGAATTTTCGCTGACAC
TTTCCAACGGCCGGTAGTCCCGGTGTTGTTCTGATCCTCGACCACATGGTAACGATTGACAC
GGTCGGCGCCCGGTCGCCTGGCCGACTGTTGAGCGGCCGGTGTGTTGCTTGTGTGCGTCACG
TCCGGGGGCTGGATGTTGCAGTACGCGTGGGGCGTGTATTCCGCGATGTCGGTAATGTCCG
CACCAGGAGGTCTCCTAGCCGGTGGTCTCGGAGGCGGAGGCGTGTCTCGCGCCGAACCGGT
GCTGCTGGTATCCGACAGTCCTGTGCCCGTTGGACCACCAGCACCAGGACTTTCTTCGGTAA
CAACGCTGTCTTCGTCGCTACTCGATTCTCCACTATCGCGGCGCTCTCTGTCGGGACTTCTGG
CCATCACGGAGGTTCTTTTCGATGGCATCGGTAAAGATGGTTTCGGACGACCCTGCATTGCT
GCCAAAGCTTGTTGCACCGCCTCCTGGCGCACCTCGGAATGATAGCGGCTCTCATTGCGCG
TGAGTCTGCGATTGCCGCGTCTCGCAGCCGCCGAGTTCGAATTCCCGGTGTTTCCGGTGGAG
CCGCCAGATCCCGGGGGACTGGCGATTCCTCCAGGTACTAGCCGGCCTCCTGAATGATAGC
GCTTCTCGTTGTGAGTGACACGGCGCTGCGTGCGGCGTGCACGTGGCTGTGGTCGGCCACC

GACACCGATACCGACCCCGACGCCGACGCCAACGCCGACGCCGACGCCCCCACCACCAAC
ACCACCACCACCGCTTCCATAACCTGTTCTCTCGACCCCTCCGATACCGAGGACGCCGACA
CCTCCTCCATCCTTTCCAGCCCCCAGTTGCTTTGAGGCATACTGTTGTAATAGACGTGTCCGT
TTCTTCTCATAGCCCTTTTGCGTAATATCCCCTTCAGATAACTCAAGATCCAGCTCAGCAAG
TTTCTCCCGTACGTCCTCAGGGAGCTTGCCGATGTCGATGTTGAACTCGGCCATCACTAAGC
CATCCGCGCCGAGATCACCAATTCTCCAGCCGTACATTCCACGACCCGTCACCACCGCTCG
GCCATCTTGCC

311. >MSTRG.6397.1 Gene=MSTRG.6397
AAGAGGAAGCATGAAACTCCATTCCCCTCGATGACCTTTCTCTTTCTGTCGATTATCATCGA
GCGTGCGCATATGCGTCCGCGATCACGCAGTAACGCACGACACGCATATACTGTTCATTCT
CCTTGAGAGTTGCACAACGCACCGCCTTCCCATTGTTTCGACCTCACACGGTATCGTTCATA
TCCATTGAATCTATGGAATCCATCTCGGTGCCTGATCAAAATATAAGATTATTGGAATCTTA
ACTCTTTCGAGAATCGTCTGCAGCAAGTATATAATTTCCTATGGAATCTCTTTCATAATGCTT
TTTGTTTTAATATTTTTAATATCGAAATATGTATTATTTCGTTATATAAATAGAAATTAATTTT
TTACATTTATAAAAAAAATTGACTGGAAAATATTGAAAATTGAATTATTTTTATTTTAATCA
TTATAACTTACGATTAACATTTTTTCAAACAAGTTTAACGAAAGTTTAATATTATCGAAAAA
TAATACGTTTCGGAAGCGATTGATCCAAACGAATTAATATATGGTGAAATCATATAGAACT
TTGGATGTACTAAATAGATAATGTGCAGCTCATTTTGCAGAGTGCATAGAAAGGTGCGTGT
GAGCAATAGTCACGCATACAGTGTAGTATTCGTAAAAGAGCGCGTGTCCGTCGTGTTCTGC
GCGACATACACGCGCTCACGAACAAAGAAAAAGCCCTAGCGACCAGCAATGCGGCAACG
GTGAGCGCCTGCGCGCCACGCTATGTGAGTCAAGGACGACACGTGCGCTTGAAACGACCT
ACTTATATCGCCGTTGCTTCGTGCACGATCGGTTCGCAGCACGAACTTTCCCGAACGTAGAA
ATAGGACGACCAAGTTCCAATGTTCTGTCGAGCTACCACCCCCTCCTCTTTCCTATGCGCGC
AAGCTTCGTTAATCGACTAACCGCTATTCGCATAGCACCGTTCTTTCGCGTTTAATCTTTGAA
TCGTAAAGATCGTTTCAGTTTCATTTTAATTTTTCTTCATTTTTTTATTCCAACGCATCGATCG
TACATTTCTTCAATTTTTTACAATTTTACGTGTTCATGGATATTGTGTGATCATCGTTTTATCT
ATTCATTAAATGGTAAGTTAATATTTATCTCTGATCTTTTACGTTTCAAATGAAAAAAATAA
GAAAAAAAACATTAGTATCGATACGCATATCACGGATTACGTAATGTCAATTTACGTTCAA
CAATTACTGTAGATTTTCAAAATCTTCAAAATTAGCAATCAATAGTTTTTACATCGAATTTG
AAAAGTTAAAGTTATTCTATTCGATTAAAATAATGATTAAATCTAATGATTAGCTCTACAAT
AGTTGGAATATCTGGAGAAAAAAATCTTAATTGTTAGAAACCATTATGGTGCCCTATATGT
AAAGATCGATAAAAAAATGGGTCAAAGGTTTCAATAACGCTTTCAATATAAATTAAAATA
GCAAAATTTGATTATTTTTGTCAGAATATTATCAGATGAAAAACGATACGGAAAAAAGACA
AATTCGTATCTATGCTAACATATTTATCATGGTTTTGCGCATAGCAGCAAGAAGGGTTGGTT
GAAATCCATCCCCGTGTTCGTATTCAAGAAATATATACACACTACTACAATGATCTTTTGGA
CCTGTAACATGGAATCCAAAAGCGGTGGGTATGAAGGCTGTCTCGATTCGTCGGTTTTCTTC
TCATCGACAGCATAAGTAGCGCGTTTCCGCGCGGCTTGTGGCCAGTTCCTGTTCGACCATCA
TACGATCGTAGCGAAGCGCGGGCGTGTGGAATCTTCCAATATATACCACACACAAAGATAT
GTCGATGGCTTCTATGTGTGCGCACACCTCTCGCAAGTAGCAACCATACATACACGTGTGTG
TATTGTATACATACACATATGTATATATATATATGTATATTCATGTGTATGTGGTGTCCTCGC
AGAATATCGTTTGTGTAAGATTGAGAGTGGCCTCGTGTGCTTCCTACGTATGTGTAATTGTG
TGCCTCATGGATAGTGAACGCCGCGGGAATTCGTGGTGTGCAATCGACCAATGGCAGGCCA

AGTAGCAACGGCTATGCACCAATCAGAGAACAGAAATGCTCGCGAATGCGCGCCACACTT
ACCACCGGATGCCGATCATACTTGGTAGTGGGGATAAACTGAGAGTGCGGCACAGGCTAC
GCGCTCGAGCGCATCCTCGTTCGTCGCTGCTCGCCGTCGGAGTCGGTCGTGTTCGGTACTTG
AGTCCTCCTCTCCGACAATTTTCCGAAGTCTGAAGAAAGCTCTCTCGCCGTTTGCCAGAGGC
TCGAACGCCATCGTGGTGATTACCACGTTCTCCATCGAACGATTGTTCCAACACTTTTACTT
CTCATCGTTCAATTTGGATCAAACTGCGGTCTGTGCTATCTTATCACTCCTGGGGACGTTTAA
AATACACGACCTTATCGTGATAAAAAAGAATTCGTGTCGCATATCCGTTCGATTTTTATTGT
GTCGTGGCGTGGTCCGTAAATTGCGTGCGTGGCGTGGTATCGAGACCGGACTTAATTGGAA
GCGAGCACCAAATTTCAAATAATTGGCTGTGAAATCATACATACTGCCTTGGACTATTCACT
GTGCACCATTTTTTTTTTTATCTTTTTATAATTTTCCTGTACAAGTGATCGCGATCTCTAAAGA
GTAGTTATTGCGGAAATTGTGCATTTGCATCTAACGAGCGATTTTAATAAATCGAATCATTT
GAAAATTCAACTAATCGGTCATCGGTTTTATCATTTAATTTGTATTGTGTTTCTTTTCGAATA
ATAAATTATAATTTTTTATTTTCATAATTTATATATAAACATATCATATATAATTCATTGTAA
AAAAGAACATTGTTTCATTCAATATAATAGCACATGTCAATCTATTTCGCATATAATTTTTCC
GATTTATATCGTGTGCAGTGAACTCCGATAAGTGCCAGCGCGGCACATGGAAATCTAACAG
ATCTTTTTTAAAATCAGTTATAATCGTGAAAGCGAAGAAAAAGAAAAATATGAAATAATGC
AGGAAGATTCTATTAATTTGGTGGATAACGATCTGACGACTAAAGATTTACTCTTTGTGCAC
ATAAAAAAGGATTAAGCAGAAACGTTGTTCATACGTTGGCGGTGCTCGTGTCCGTGTTCCTC
TGGCTATCAGTGGGTAAAGATGGCGGTCTCGTCGTTCGGTTGGTTCGTTGGTTGAGTAGTGA
GAGGGGCAACTGTGACGCAACCTAACGTCACGTGATCAAACCGTGGCTCTGCTCTCGCTGG
GCCAGTGTGTGATCTAACGTTTGTTACGGCAGTTGGTGGAAGATAACGTCTTGAAGAGTGC
AGGATAGACAAGGAGTACTTTTTTCGACGTTGCTCTCGATGGCTCACTCGTTGCCCGACGCA
ACACCTTGATCTTTTTCTCGGTCGCTAGGTCAGTGGGGGTGAGAAACTGGCTTGGCTCTCTG
TGCTACGATAGTCAGTCTTTTTCTCTCTCCTATCGGCTTTGCGACATATCTCTCTCGACGTCA
ATCGAAGACAGTCTCATATTTACAAGAAGATAATACATTTCATTTTTTTTTTTTTTTGTATATT
TTAAACGAACGATAATTCTATATATATATATAATTCTATATGTATATCTATATACATATATAT
ATATACATAGAATTGTTTACTGGTATTTGTGGGCTCAGCAGTGAAAACCGTGTTCCCCCAGC
TGTAATCTTCTCTGTTAAAAAGGCTCTGGGATCAGGGAAAACACTGCGCGAGTACTGACTA
TTCTTCAAGCGTATCATCTTTTCGTCTTTCCTAATCGTGAACAGCTTAACACATATATATAAA
TTTTTTTTCCGTTGTTAAGGTGTATTCCGTATTTAAAGCGTAAAGTGCAAAGTTCGACCAAG
AAATTTAATTCTCGAAATAAAGGTAAATGAACTAATACATGCAATTGAGAAGTTTGGAAAG
TAACCACTCCTTAGGAAGAATGGGCAGGTAGGAGACGATGATGGAATGAGATATACTGTG
TGTGCACATACATACAAGGAGGAAAGGGAAGTGGGACTGGCTTGCCTTGTGCCACTTTATA
CATCGCGGATCATCACGAAGAGGTGGGGTGGGGATAAGGAGCGTGTTGAGCGACAGTGGG
ATCGACGATGAAAAGGAAGTTAGCGATAGCCTTAAGATACGTCAAATTTCTTAAATACGTA
AAACATAAGTTAATTACGATTATATGTTAATAATTCAAATGATATTAACAGTAGAAATGCA
ATTAATAGGAGAGAAAATATTATATAACGTATTTCATCTTGTTAATATATCTTTTCCATTTTT
TTTTTACATGTTATGTTACGGAAAAAATTGTCAATGGCTGTTTTGCGAGATTGACATTTACAA
CATCTAGATCTTCGATCTTCACGCTTGTAAATTCAAGAATTTAAGAGGTGAGGAATTGAATG
GCTATCGCTAGACACAAAAGTCCAAGGCGGTTAAAG
AATAAGTTGGAGAGACAAAGAGCGGAAGAATAGAGACGAAGAAAGAAGAAGTGAGGTTT
CTCTACGTAAAGAACTTGGGGAGAGGGGAGAGGAGAATGAAATGATGTGATATAAGTATG
AGAATCCAGTGGTGGGGTAGACACCAGGCAACCAGGCCTTGAACAAGCGAAAGAGAACA

AAAACCTTCGGATCCTCGACCGTGCTATACGCGCTATATTTTACATTTCATAAAGTTGTTCA
AAAATCGACTATTACTTCCTCTTTTGTCAAATCGTCGTTTCCAGTTCATCTCTAAAATATATA
ATCGATATTTTTTATCAATTCAAATTTCATTCCATGTTTAA

312. >MSTRG.6397.2 Gene=MSTRG.6397
CTTGAGAGTTGCACAACGCACCGCCTTCCCATTGTTTCGACCTCACACGGTATCGTTCATAT
CCATTGAATCTATGGAATCCATCTCGGTGCCTGATCAAAATATAAGATTATTGGAATCTTAA
CTCTTTCGAGAATCGTCTGCAGCAAGTATATAATTTCCTATGGAATCTCTTTCATAATGCTTT
TTGTTTTAATATTTTTAATATCGAAATATGTATTATTTCGTTATATAAATAGAAATTAATTTTT
TACATTTATAAAAAAAATTGACTGGAAAATATTGAAAATTGAATTATTTTTATTTTAATCAT
TATAACTTACGATTAACATTTTTTCAAACAAGTTTAACGAAAGTTTAATATTATCGAAAAAT
AATACGTTTCGGAAGCGATTGATCCAAACGAATTAATATATGGTGAAATCATATAGAACTT
TGGATGTACTAAATAGATAATGTGCAGCTCATTTTGCAGAGTGCATAGAAAGGTGCGTGTG
AGCAATAGTCACGCATACAGTGTAGTATTCGTAAAAGAGCGCGTGTCCGTCGTGTTCTGCG
CGACATACACGCGCTCACGAACAAAGAAAAAGCCCTAGCGACCAGCAATGCGGCAACGG
TGAGCGCCTGCGCGCCACGCTATGTGAGTCAAGGACGACACGTGCGCTTGAAACGACCTA
CTTATATCGCCGTTGCTTCGTGCACGATCGGTTCGCAGCACGAACTTTCCCGAACGTAGAAA
TAGGACGACCAAGTTCCAATGTTCTGTCGAGCTACCACCCCCTCCTCTTTCCTATGCGCGCA
AGCTTCGTTAATCGACTAACCGCTATTCGCATAGCACCGTTCTTTCGCGTTTAATCTTTGAAT
CGTAAAGATCGTTTCAGTTTCATTTTAATTTTTCTTCATTTTTTTATTCCAACGCATCGATCGT
ACATTTCTTCAATTTTTTACAATTTTACGTGTTCATGGATATTGTGTGATCATCGTTTTATCTA
TTCATTAAATGCAGCAAGAAGGGTTGGTTGAAATCCATCCCCGTGTTCGTATTCAAGAAAT
ATATACACACTACTACAATGATCTTTTGGACCTGTAACATGGAATCCAAAAGCGGTGGGTA
TGAAGGCTGTCTCGATTCGTCGGTTTTCTTCTCATCGACAGCATAAGTAGCGCGTTTCCGCG
CGGCTTGTGGCCAGTTCCTGTTCGACCATCATACGATCGTAGCGAAGCGCGGGCGTGTGGA
ATCTTCCAATATATACCACACACAAAGATATGTCGATGGCTTCTATGTGTGCGCACACCTCT
CGCAAGTAGCAACCATACATACACGTGTGTGTATTGTATACATACACATATGTATATATATA
TATGTATATTCATGTGTATGTGGTGTCCTCGCAGAATATCGTTTGTGTAAGATTGAGAGTGG
CCTCGTGTGCTTCCTACGTATGTGTAATTGTGTGCCTCATGGATAGTGAACGCCGCGGGAAT
TCGTGGTGTGCAATCGACCAATGGCAGGCCAAGTAGCAACGGCTATGCACCAATCAGAGA
ACAGAAATGCTCGCGAATGCGCGCCACACTTACCACCGGATGCCGATCATACTTGGTAGTG
GGGATAAACTGAGAGTGCGGCACAGGCTACGCGCTCGAGCGCATCCTCGTTCGTCGCTGCT
CGCCGTCGGAGTCGGTCGTGTTCGGTACTTGAGTCCTCCTCTCCGACAATTTTCCGAAGTCT
GAAGAAAGCTCTCTCGCCGTTTGCCAGAGGCTCGAACGCCATCGTGGTGATTACCACGTTC
TCCATCGAACGATTGTTCCAACACTTTTACTTCTCATCGTTCAATTTGGATCAAACTGCGGTC
TGTGCTATCTTATCACTCCTGGGGACGTTTAAAATACACGACCTTATCGTGATAAAAAAGA
ATTCGTGTCGCATATCCGTTCGATTTTTATTGTGTCGTGGCGTGGTCCGTAAATTGCGTGCGT
GGCGTGGTATCGAGACCGGACTTAATTGGAAGCGAGCACCAAATTTCAAATAATTGGCTGT
GAAATCATACATACTGCCTTGGACTATTCACTGTGCACCATTTTTTTTTTTATCTTTTTATAAT
TTTCCTGTACAAGTGATCGCGATCTCTAAAGAGTAGTTATTGCGGAAATTGTGCATTTGCAT
CTAACGAGCGATTTTAATAAATCGAATCATTTGAAAATTCAACTAATCGGTCATCGGTTTTA
TCATTTAATTTGTATTGTGTTTCTTTTCGAATAATAAATTATAATTTTTTATTTTCATAATTTAT
ATATAAACATATCATATATAATTCATTGTAAAAAAGAACATTGTTTCATTCAATATAATAGC

ACATGTCAATCTATTTCGCATATAATTTTTCCGATTTATATCGTGTGCAGTGAACTCCGATAA
GTGCCAGCGCGGCACATGGAAATCTAACAGATCTTTTTAAAATCAGTTATAATCGTGAAA
GCGAAGAAAAAGAAAAATATGAAATAATGCAGGAAGATTCTATTAATTTGGTGGATAACG
ATCTGACGACTAAAGATTTACTCTTTGTGCACATAAAAAAGGATTAAGCAGAAACGTTGTT
CATACGTTGGCGGTGCTCGTGTCCGTGTTCCTCTGGCTATCAGTGGGTAAAGATGGCGGTCT
CGTCGTTCGGTTGGTTCGTTGGTTGAGTAGTGAGAGGGGCAACTGTGACGCAACCTAACGT
CACGTGATCAAACCGTGGCTCTGCTCTCGCTGGGCCAGTGTGTGATCTAACGTTTGTTACGG
CAGTTGGTGGAAGATAACGTCTTGAAGAGTGCAGGATAGACAAGGAGTACTTTTTCGACG
TTGCTCTCGATGGCTCACTCGTTGCCCGACGCAACACCTTGATCTTTTCTCGGTCGCTAGGT
CAGTGGGGGTGAGAAACTGGCTTGGCTCTCTGTGCTACGATAGTCAGTCTTTTTCTCTCTCCT
ATCGGCTTTGCGACATATCTCTCTCGACGTCAATCGAAGACAGTCTCATATTTACAAGAAG
ATAATACATTTCATTTTTTTTTTTTTTTGTATATTTTAAACGAACGATAATTCTATATATATAT
ATAATTCTATATGTATATCTATATACATATATATATATACATAGAATTGTTTACTGGTATTTG
TGGGCTCAGCAGTGAAAACCGTGTTCCCCCAGCTGTAATCTTCTCTGTTAAAAAGGCTCTGG
GATCAGGGAAAACACTGCGCGAGTACTGACTATTCTTCAAGCGTATCATCTTTTCGTCTTTC
CTAATCGTGAACAGCTTAACACATATATATAAATTTTTTTTCCGTTGTTAAGGTGTATTCCGT
ATTTAAAGCGTAAAGTGCAAAGTTCGACCAAGAAATTTAATTCTCGAAATAAAGGTAAATG
AACTAATACATGCAATTGAGAAGTTTGGAAAGTAACCACTCCTTAGGAAGAATGGGCAGG
TAGGAGACGATGATGGAATGAGATATACTGTGTGTGCACATACATACAAGGAGGAAAGGG
AAGTGGGACTGGCTTGCCTTGTGCCACTTTATACGTACGTACCGCTATCGAGATGTAGACTC
GATACTTTGTACCGGTCTTGTAATATTACCAGATTTGTTCTTTCTTTCTATGTCACCGACATTG
GCTTCACTTGTTACCAGTGTTGTACAATTATAGCGAATTATTATATCTTTTCGTTTTCTATTTT
TCTATATAAAATTTAAACGAATCCTTTCGTTCGATCAATATAATAATTTTCTATTTTTTATAA
AAAATTCAAATAAAATTCAAAACTTTGTCCTTTGTCTCTTATCACTACCATTTTGGTATTTTA
AGAATTATGATTCATTTCGGACACGACAGTAGAACGAGTTGATTCCATTGGTCAATTCCGA
AGAGTCGTAAAAGGAATTGGGAAGTGGCTTAGAAAGGAAGGAGACGTAAAGCCGTAAAT
ATTTTAAAATTCGTGAAAAGAGGCGAGCGACGGGTGGTCAACAACCGCCATTTTATGGACG
CCACCGCATTGCGGCCATCTTTTGTATAGTAACCGAAGCGTTTCTATTTCCATTTGGGGTTGC
TCGATTGGCTCGGATTCCATTCACGCGCTGTCTAATTTTACTCGTTGGCCTCGAATCTACCAC
GACATGGCTCGTTGCATGCTACCTGAATCCGAAAAAAGACATTTCTAGTTAATATTTTCCGA
ATAATTTAAATCTTTCGAATCCATTACTATCTTCACATATTTTTGCGTGGAA

313. >MSTRG.6401.1 Gene=MSTRG.6401
GAGCCCGGCAGTGAGGCGAAACGTTGGGCTCCGCGCGGGAGTCTCGTGGCCGCCCGCCTTT
TCTTTTAAGTCGGGGTGCCTTTTTCCTTACACCCACCCCCCGCCCCTCTTCTCGTCGCCCTCG
CGGCTCTACGGACCCATCGTCGCTCTCTTTCTCAAGTCACACGCGAGTTTATCACTCGCCTT
GCGACTCCGTTGATCGTTTACGTCCGCGTTACGATCACCACGGTTCGTTTTCTTCTCTTACGA
TCTCTTCTTTCCTTTTCTCCCCCCCCCCTCTCCCTCCCTCCCTCATTTTTTTTTCTTTTTCTTTAA
TTTCTATCTTCGCTTGTTCTCAACGCGACACTCGATAGATAAACTTCTTTTTCTTCTTCTTATT
CTTTTTCTTGTTCTTCTTCTTCTTCTTCGTGAAGATGTACATGTTATACGGGTTACAATAAAAC
ACAGATTTGCAGGCTGATATCGGATTATAATTAAGACTAAGAGGGAAGGAGGAGAAAGAA
ACGGAGAAGGGGTAGAGAAAGGGTTATCCTATAATCGTGGAACGAAAAGTTTGCTGCGGA
GGATCATCGCCGGTGCGTGACCTCCGGCCCCTAGTCACATGATCGCCGTGCGTGCACGGTG

ATTTCGTTGCTTTCGACGAGAAGAGGATGCACAGTGATCGTTGGAGAGGATTCTAGTGTTCC
GATCGGTGTCGTTCGACAGTGGTTTGAGTTTGGGAACGGTAAAAAAAAAAAAAGTCACTCG
TTTGGTGATCGTTCGACGTATCCAAGTGGACGAAGAGAAAGAGGAGGACGTGCAACGTGA
TTTCGTAGCGCGCTGCCGCGTTGCCAATGTCAATGTTAAACAGTGCTTGGAAGACGGTCAC
GGTGATACACAATGGCAGTCGAACCGGGAAGACGAGCAAGTGGCAACGTCGCAATTAGTT
GGAATTAAATGTCCGCGCGGAAATGTCGATCGGGATCGGGAGGTGGATGAAGGTCGAGAT
CGAGCTTGTGCGTGACCACGTATCCTCATCGTGGAGAACAACGACCGTGTGGCTGGCTTGA
GGCCAATACAAAGTCTAGTTTAAGGGGGTGAACGTGAAAATCAGGTGGATAGTGGGGGTG
GAGGTGGACTCGAGGAGAGGGAGAGTAGAAGGCGAGCAGGAAGGAAGGAAGGAAGGAA
GGAAGGAAGGAAGGAAGACTTGGTGGGCGACAAAGACGTTTTCCTAGTGAATTTATTCGC
GGTTATTTTGATTGTATCCTCTGTGTGCAAGATGCGCTCGCGACGCCGTGACGGCGGCTGAA
GGACGAAGAAAACGAGCGGCGCTCGTACAGCCCTCGATTCCACGTGTCGACCCGTCCGTTT
ATTTTCTTTTTCTTTTCCAACGAGAAAGCGCACTGCGGAGTCAACCCGTCCTCGCTTATCTCC
TCGACGATTTTACCGATTCGCCGCGAGAACGCAGTCAAGATCACGCAACGCGAAGAAGCC
GTGAAAGAATAACCCAGTGGCGACAGACACGGAGCCACGAAGAAACGCGGGATTGCAAA
AGGAGGGGGGGCGGAAGGAAGGAGGAGGGGGCGAGGCGAAGCGAAGAGCGAGCAAGTG
GAAGAAGCGGAGAAAAGAGGGGAGAGGGCGCGATACGAACGTACGTGCGTAAAGTTTCA
GAGTCCAGAGGAAAGAGAGAGTGAGAGAGAGAGAGAGAAAGAAAAAAGCCAGACACAG
AGTAGCATCGAAAGTATAACATGCAATTGAAGGGGAAGGGGTGCGGGGCCGAAAAAGCC
TTGTGAAGAAACTAAGGATTAGATGGTGGAGGTGCTCGAGGGGCGGGCTAGGGAGCCTAC
GGGGCCGATCAAGGCACCTCGCACCTACCCAGGGCCGGCCAGCCCTGGAAACAGCCTCCT
CCAGGCCCTCGACGCCGAAACAAATCCGCGGCTGCGTCCCTCTTGCCTCGCTTCCGACCTC
GCTCTCTTAAGGCCCCTCGCTCTTCGCCAGATCGTATCGTCGAGATCCCGATTCTTCACGGC
GAGCGCGGTGGTCGAGGGCAGTGACAACAAGCTCGTGGACAAGCTCGGCCTCGATAGGCC
GATCGCGGCGCGGGAAGCCGCGAATCGGTTCGGTGGGGAGGGGCAGGAGCGTGGAAGAG
GGAGGAGGCGGGGAGTAGGGAGGAGAGACGGGTGTTGGGCAAGGAGCGGATTCGACGGC
GAGGAGGG

314. >MSTRG.6410.2 Gene=MSTRG.6410
ATATTATATTGAAGTTTGCAGCATATGATTGCATATTTGTTGCTAATCGCGTATAAGTTTTAC
GTGTCCATTGAGCCACGTCAAAATATTTATTTGATACAATGGTGGAAGTCGAATCTATGACA
AGATATGTCTCTCCTATTAATCCTGCAATTTTTCCATTATTGGCAGTGGTATTACTAGGAATT
GGAATATTCTTTACAGCATGGTTTTTTGTATATGAAGTTACTAGTACAAAATTTACAAGGGA
TATGTTCAAAGAATTATTAATTTCTTTAGTGGCAGCATTATTTTCCGGTTTTGGTATATTATTT
TTACTTCTTTGGGTTGGAATTTATGTATAGGTACATTATTTTATAGAAATTTAAATCAAAAAA
ATTAAACAATACATTGAAATACATAAATTTCATTTTTTTGTTTCAGATTCATCTTATAAATGA
AAAAAATTAATTTTAAAATTATATGAAATAGCATTTAATAAATACAATTATATAGATATTAT
AAGAATAAAAATAATGTATTTTTACTAAATCATGCAATTATAATGATTAATCTATATATTTA
T

315. >MSTRG.6422.2 Gene=MSTRG.6422
CACTTTGTGCACTAGTGGTGCCGCTATCGCGGTTCAAGATGTGTCGTTTTTAGGTTTATTTTTT
GTGAAGTCATGATATCATGTTTCGACCTATTTTTTTCATAGACATTAGTGTAAATTGTGTGTA

GAAATGTGGAAAAGTAATTATTAAACTTATTAAATGTGATTAGTTTAGTTAAAAGTTAAATT
AACTAGTGCAAGGTGTGACCGCGCGTGATTCGAGGTAAAGGATAGCTTCTTTCTACTCGAC
TACCTTCCATACTAGCTTTTACAGGTTGCCACGAGATCGTTTCATATAAATGTTCTTTTTTA
AGTAGATCGTCAAGGTTTTCCTACTGACTTAGAGTGATTTAAAAATCAGAAGAAATAATCG
AAATGTTGATAAAAAATAATAGAAAAAATAAAACGAAATTGATAGCGGGAAAAATTGAA
TCGTATATTACGATCTCACGATA

316. >MSTRG.6517.1 Gene=MSTRG.6517
GTGATATTTTAAGATGTCTTTTCTAACATCTTGTGTTATATTAAATAGTGTCTCAATTTATTTA
TGTTTCGGTTTTAAAATATTTTAAAATAAAGTTAAAAGTTCTGTTTATTTTTTAAAACACAAT
AATATAAAAGGATCATTATGAATATTCCTCCAATTCAACAACCAGGAGCAATTGGTGTAGG
ACAAATGACACAGTCTGTAAATGCTCAACTTCCTCAAAATCCACAACAAACACAACAGAC
ACAGGTTCAACAGCAACAGCAACAACAACAGCAACAACAAACTCAAGAAAAACTTGATA
ATATTTCTAAAGTTAAATCGTTAATTGTATCATTAAGAGATTCATTGGCCATAGCTCTTAAA
ACTGCAGCACATACATTACATCAAAATAGTTTAGTAGATGTTGGATCTCTAAAAGGTGGTA
TTGATCAGCCAGATCATAGATTCAATAAAAATATAGAAGAGTTTTATTCCATTTGTGACCAA
ATAGAATTACATTTAAAAACTTCAATAGAATGTACATCGCAAAATTCTAGTTCTCTAAGGTA
TCTTCCAGTACCTGTCATGCCACAACGTACTGATACTGTTGGTACTCAAGAAGCTTTAACAT
ATCCTCAATTTTTAATGACTGTAAGAGCTCAGGTTGCATATGCTCGTGAAGTTCATGATACT
TTAGTTTCTGCTGCACAATCAATAGCATCAGGTGA

317. >MSTRG.6565.6 Gene=MSTRG.6565
AAAAATATCTATGCTCTGTATATGATATGATATGAAAATATATCACTATTTTTTTTATGAAA
AATATTAAATTTATAGCGAGTAATTTTTTTTTTATATAATAAAGAAAATATTGCGCTTTCTT
TTTTTATTTTTCATTTTACTTTTCATTTTTGTTTATATCTTTTATTTATTTATCTATTATATAATTA
TGTTAAATATTTAAATTTATACATTTTACATATTTTAATTTTCCTATCAAATTATGAATTTATT
AATGCAATCTATTTTGTTAAAAAAGAAAGGAACAGAAGGAGATAAAGAATCGAACGATGC
GCTTGTGTTAAAGAATTGAGAGGAAAGATTGGAATAAAGGGAAAGTACATTAGTAATAAA
TGTGGGCATCCTTTAATTTATGTTAATCAGTCTGGTTGTCCTTTGTTTCAGAGGCACATGGTG
CACAAATTGTGTTGAGTGTGCGATGAAAAACGATCGTTCAATAAAGAAAACATGGCTCACT
CATTGTACATGCGCAATTCTGTTTTGCACATAATATTGTACTTAATGTGCCTCTGTGTTCAGC
CTGTGTACCACGCAAGTAGTAGAGTGGTGCGCGGGTGATACTGTTGGTTGGTTGTGAGATA
AGACCATGGTTTACATTTTGGTACAACCATCACACTTCATAGCCAATGCTCAGCCCGAGAC
AACAATCTATTGATTTTGCTACTATCGCTACTCTGTGATGATGCTTAGGACACGACGTGATA
TGAAATTGCATTTAAGAATAGCATACATCGTGGGCGCTCTGTATGCTGTCATAATATTCAAC
GATTAAACATGATGTTGACGATGTAAAGTACGTGATCAACTTTGATTATCCAAATTCATCTG
AAGACTACATTCATAGAATTGGTAGAACGGGCCGTTCAAATAATTCAGGAACGAGTTATGC
ATTCTTTACACCACAAAATGGTCGACAGGCTAAAAGTTTGGTTAATGTACTTAGAGAAGCA
AAACAAATTATCAATCCAAAACTAATGGAACTTGCAGACAGAAATGGAAATGATATCTCA
CGAAATCGTTGGGGTTATGGAACTTATCGTAAAAGAGAAAATGTTTTCCCTAAAACACATA
AACGATTTGAAGTGGTCTAGATTAACTTTACAAAATTTCTAAATGTGTAACTTATTGGAGAT
GGAATTTGTTACAATCTCTTTCTCTGTGATTAGAAAAAAATTGTCCATCAAGGCTCTATGTTAG
TTCCGTGCTCGTTAATTTTTTTATTTTTATTTATAATCTTACTGTTCCTAGTAAGTAATTATTCT

TAGTATGAAATTAGTATAAAATGAGCTATGCAAGGAGAGACTCTTTGTTAAATTTCCATTAT
ATAACAATATTTTGCATTAAATTTTTATTTTTCCATTTATTAAAACATGAAAAGAGTGAAAA
ATATGTGATATGAAATATGCGTGAAAATGTAAGTATAAAGAAATACATGAATACTTACATA
CTGAA

318. >MSTRG.6586.1 Gene=MSTRG.6586
CGGTTCTTGAATCAGTCGAGGCAACGCCGTCGAGACGAGTGTTGCTTCCTCTTTCCTTCGAT
CGATCGATCGCGATCGCGGATTCGCTTCGAACGTATTTCGACTTTGGCTCGAGTTTTTTTAT
TATTTTTCTTTTCACCTCGAAGGTTTCGAGGTGATTTAACATCGTGACATCGAAGAATCATCT
GGTTTAACTGGGACGATTCGAAAAGATTTCGAGGAGGCGCAAACAGGATTAGAGGAAGAA
AAGGGGAATCATGGAATCCTGGCTGAGCGACGTGCAACGCGTGAGAATTCCACGGCCGAA
ATTCGGCAGCGGGAACTCGGTGAGGCGGTCCAATTCAACGGTGCAACACGTGCGGCCGGA
AGTGCTTTCGCGGCAGACCCAGGACGTGGCGAAGGCCACCGAGGAGCTACGACAAGCGCT
CCAGGACAAGCTGTGACTAGCTGATCGTCGATCGGGGGCCACCGTAAATTGATCGAGCAA
ACAAACCCGTGTTCGAGGATCATCGTTCAAGGATTTCACGCGCGCCTCGAGTGTACAATAC
CTTTCTCTCTCTCTCTATTTCTCTCTCTATCTATCTATCTCTATCTCTCTTTCTTCCTCGCCTTCG
ACAACGAGGACGAGTCGTTTATATTCGTGTTATTCCGTGACAAGTTTCCGGCTTCTCAGAGA
TCTCTGGATCCTCGAAGCTGGCTTCTTTTTACTCATTTAAATGCCGCGGTTCATTGACTCTTG
CCGATCGCCGATGTCCGATCGGAAATCAAATTTACCGGGACGAAAAAAAATATGGAACGG
AATTGGAAGCGAGCAAAGGTTAAGAGTTATTCCGGATAAAGAAATGTAAAGTATGGAA

319. >MSTRG.6592.1 Gene=MSTRG.6592
TTGATATCGAGGGGCAGGGCTAGGTGGGCGAAGACGGAGAGGGCGCAGTGGTCCCGCGAC
CATCAAACCAACAACACCGTCCCGACGACACGAAACGGCGCATCCTGCTGCGCCCCCTTCT
TCTTCCTCTGCCGATCCCGCTTCTGCCGCTTCTTCTTCTTCTTCTTCCCTCGGCCGGCCGT
CCTCCTGTTCCATCGTAGTCGGCCCACCGCCACGTCCTTGCCGCCTTCGTCCACGCGGTGTC
GAACGCGATGGATCTACGATGGATTCCGCCGAGATTTTCCGGGAGATTCTGCCGAGAGCTG
GACCCCCTCTCCCCCCCCGAAACGAGGAGGATCGAGTCGATCGCACGGTCCTCCGTTACGG
CCAACACCGTATATACGGACGGAGGAGGATCTACCGTCGATCCACCGTTGGATCCGTGGAA
ACACCAAGTTCGTTGGCAGATACCAGGTAGAACCATGCGGTTCGTCGGCCCGGTACCAGGG
CTCTCATGAATGGAGGAGGGTTCGCCAGGATCGATGAAGAGGGCGTGCCTTCAGGAGAGG
ATTGGTATCCGTGACGTTTTCAACCCCACCTGAGAAGGCCGCAAACGGAGTGCACGTGCTC
GATTACGTCACAAGGATAGCCGGGAACGGAGAGTAAAGCCATTCAGTGATCCTGCCTTGG
GCCGCTCGACTCGGTCCTGCGACTCTACTTCCGCTTGTGACCGTAACCGTATGATAATTAAC
ATCAACGTTGCGGTAAGAGTCGCTGCCCTCGCTCTTTCTTTCTCTCGTTTTTTAATTACGGA
ACGAGGAGAGATTCTTTGATATATATATATATATTATTCTTTCGACAGA

320. >MSTRG.6636.1 Gene=MSTRG.6636
AGCCATTTTATATCAAGAAACTAACATGGCACCTTCCTTATACTATCCGAACGAATTGTGTT
AAGGATTTACAAGTTGTAAAACACGAAAAACATTAAAAAAAAAAAAAAAAAAAAACACA
TTACATATTCATTATAAGTTTTAAAACCAAATATTTAAATTAAAATGCCGGCCGTACCAGGC
ATGTATCAACAAGCTCCTTCATGCTGGGATAGGGTAAAACTTGGTTTTATGATTGGATTTTG
TGTCGGAATGGCATCTGGTGTTCTATTTGGGGGATATTCTATTGTTAGATATGGATTAAGAG

GAAGGGAATTGATAAACAACGTTGGAAAAGCAATGCTTCAAGGTGGTGGAACATTTGGAA
CATTCATGGCCATTGGAAGTGGAATAAGATGCTAGTAATTGAAGAGTGTTACGTTTCACATT
TAGGATACAAAAAATTGTAGTATCAAAAAAATATTCTATTAATTTCTTAATAAAATATTTCT
ACGGAGCAAGGGCATACCTTCCATTGCAACTACAGTGGTATCATCCATAGGATTTGTTTTTC
CTGGAATATTTAGTCCAAAACTTAAGTGGCGAATCTTGTGAGTCATATTAAATTGTGTTGAT
GTGTATGGTTGGACATCATGTACATGTACATGATTTACAGAGAAGCTATCTCCTGGTGCAAT
ATGAAAACTTCCACCTACTCTATTTACTTCCATATAACCATAAATTTGACATCCTTGAGTAA
AAGCAGTTTTCATTTTTTCCACTGATTTATCATTTTGACATTGTTTTATATTTCCTAGAACAG

321. >MSTRG.6687.1 Gene=MSTRG.6687
CACCGTATTTCTTAAAGTAAAAGTAGTCTTTCTTGTTCGCCTCTCGAGCACGTATCACGCCGT
ATTCACCAATAAAACAATAGACAGAATAGTAAAAAAGAAGAGAACGTTAGAGAGGGAAC
GTTCGCCAAGAAAGCGCAAAATTTGTGTTCGATTTCGTATACGTGAGTCCGAAAACGTCAG
TTCGTTACCCCTCTTTCTCGTGCTGTATTTATTAGGAATCTTTCGCCTTTTTCGTTTATAATAC
GTTTTCGATCACTTGTTATTAATGTTTCAGTTAATTTAATAGACGAGTCGGTGTTGATAAGGG
TATCCCCTCGATTGGGAGCTTAATTGAAATTGAGCCGCGTTGGAGTAGGGATCGTACGCCT
GCGAGCGTTTCCTGGAGATCATAAACCTTTTCTCGGGAGCACTGTAATAAGTCTGTTGAGGA
TAAACCAAACCGTATTGAGGGCTGCCATATTGAGACGTATCGTATTGTTGCCCGTATTGGTT
CTCGTATTGTTCGTAATAGCTGGATGGATGGGTACGAATGATTCCAATGTCCGACGGTGAC
AAAGAATCTAGGCTGTAGTGACGAGGAGGATACACCGAGGAATCCATCCACGGGATAATG
TTTCGAGAATCCTCTTCCTCGAGAAGATTGTCCTCGTCATCTACATTTTCGTATTCCTCGTTC
ATATATTTATGCCAGTTTCGTTTGCGACGTTCGTTTTCCCACAAGGAGAGAAGAGTTAAAAC
GTCTTCCGGATCGATATCCAATTCCCTCTTGAATCGTGGTTCTTGATAAATGGGAACGTTTCT
CTTCACTTGCGTCTGAACAGATTGCTGAGTGGATGTTGGGGACTGGAACGAAGTGGTGGAT
GGTTTTATGGTCGTCGATGATCCCGATGCAACCGCCGATTTCTCCGATCTTTTTCGCCGATCC
TCGTTCGTGTAATAACGGGAGTAGGGCGAATTGTCCCACACCTGGGGTATCTCGTATCCCA
GTTCATTCACAGCGCCATAATCGTAATTTTTACGATCGTAGAAGGCATTGTTGAGCGTATTG
GAACGAGATGGTTTCTCCTCTTCGATGAGAGCTGCTTTTAATCCGCCTAATCCTGACGCGGC
TAGAGTTCTCTTTTCCGCGGTGGACGAATAGGACGAGCTAGGATTGTTCTCTGGTTGATGGT
TCTGTTGGTTGCCGAACATCAACATTTCCTGTGCCCGTTTAGATTTTGGTCTCATCGTTTGCTC
AGTCTTTTTTGTGTCTTCTGCCAGGTTTGTTGGCAAGGATTGGACAATCATGAGGAGGGAGC
ACAGAGTGAGTAGTAACCACACCCAATCGCACATCATCTTTCTGTTCACTCTGGGACTGGA
CTCTGTTTTCTAGATCTCTTCTTCTTATCCGGATCTTTCTCCCAAGGGTGAAGAGAACCAGCT
AGTTCGGCTGATCGGGGTAGAATGAACGTTGGGGCTGAGCA

322. >MSTRG.6703.2 Gene=MSTRG.6703
ATGCGTAGCCACCTCCTCGTGGGTGGGACCCGGTAATTGGCATTGCTTTCCAAACAAACAC
TTCTTCCAAAAATATAAATTTTATTTTAGCTTAAAGATTACATTTACATTACTACATCCTGAA
AGCAATGGCAAGCCAAGAGCTACGCGCACCTGCAGTCCAGTCTTCCAATAATTTTAATCAT
TACGAATTATCACCAGACTGGCCTGCAAGTCCACTAGATTAACATCATTGTAAATCCGTATC
AAGTTGGACTAGTATTGTTCTCGATACGGATAAATCGTAATGTTAAGTTTAATTTTAAGCAA
GTAATTTTAAGTAACGCGGAGTAACGGATGGAAAATTTTTTACATTTTTATAAATCAGAATT
TATTGTTTAAAATTTATTTTACCTTATATCGTAAAATTTTAGAAATTAAAAATTCCATCCGAT

ACGCCCGCCATTGCGAGATGCGCGTCTTAACTGTAACTGTAAGATAATTTAGATAAGATTA
ATTAAGTTATTTGTAAGCGCTTCCCGTACAAAGATACGCCTCCTTGAAAATTATGAAATCTT
GTATTTTAATTCATTGTTTCGTTCGAATATTCCTCTAAAATATTCTGCTCGTATTTCCGTCCTC
TTCGAGTCGAGTCGCGCAGTGCCGTATCTGGGCACGTTGTCGTAGGGCGCAAGAGTTAGAG
TCGATCGAGTATTCCTTTTTTCCTTAGAATTTTCGATCGAAAGTATTTATTTTTCTCAAAGATT
TCTCTTGTTTCGCTTTTTCAAGGAGGATGTATCTTTGGA

323. >MSTRG.6724.1 Gene=MSTRG.6724
CTTTTGAAAGCATCCAAAGCTCCTTCGAGCTGAAGACACCTGTCGCTAGTATCGTGTCATTG
CAGACAACACCGTTATATAGTTCGTGGTAGCCTGCACCTTTTCGCCGCCTCTCCTCCTCCTTG
CTCGCGTTGCAGATACCGTTTAGCACCTCGTGGCTGGTGATAAAATTGGCGAGGTAGAGAT
CGAGTTCCCGGACGGAGACGTTGATGTGATAGGGGTTGACGCAGAGGCCCGGCTGAGCGC
ATTCCGCAGTCTTCTCCAGCCGTTCCCCGTCCGTCGACTCCAACGGAATTGCCTTGAAGAGG
ATCACCATTACTAGGTCCAACCTCCATACCTTATCGGCTTGACGCAGGCAGTCGATACGGC
GCATTTTACCCTTCTGATCGGGATTACTAAGGACGCACATGGCCGGCCTCTTCCCCGTTATG
CTCAGCACGAAGTCCTCTCTGTACTCCTGCGTGATATCCTTTCGCAACTTGCCCAAAAGCCG
CGAGGCCCATTTCTGTTTCACCTCCAGCTTCTCGTTCTGAAGCTCCTCCTTGCACTGACGTTC
CTCTTCGAGACTCATCCGTTTTTCGTGCTTCTTGTAATAACGCCTCTTGGCGGCTTGTAGGTT
GAACCACGTGTACGAGAAAGACTTCACGAATGGTAGAAGGGCCTCAATGAATGGATGGAA
CTCATCCTGTCCTACGGAAGGGGCCTGCAGGTAAGTCGAGACATCCGGTTCCACCATGCAG
CTCCTCAGTGTATCCGGGATGAACATGAGTAGCTGGGACGTCGGCCGCGGTACAGGCTGAA
GCCGGCTGCGCTCCTCCAGCGAGCCCGTGCTCGCCAGTAGCGTCGTCCCGACGCCGCGCGA
CGCCGACGAACGCCGCCGTGGGACGGTGCTCCAGCTGGTCGGCCACCCGCTCTCCGGCGG
ATAATACGGCGGCACGGACCTGCTTCCCTCTTCCCCGGCTACACAACAACAATCGTATTCC
ACTCTCCGCCGAGCCAGGGAGCAACGGGCAACGTGTTATCCCTCGACCGAAGGCTCCAAT
ATATCCTCGAGTCGTCCCTCTTCGAAGGAAGGTTGGAGGGACCTTCAGGGGCGAACGGTTG
TCTCCGCGCGTCCGTGTTGCCGAGACACCCGGCACCGTGGTGCTCGAGGCAACCACCAGCC

324. >MSTRG.6781.1 Gene=MSTRG.6781
GGTTGGTATTTATGATAGAATCTCTCCAGAACAGTTTCTCCACTTGCAATATAATCCTTATA
AAACTTAAGGAAATTGCCAATGGTATCATAGCGATGCTCACGCAGTAAATGCTGCGTTTCT
AATTCAACGCTGCCTGCTAGTTCCTCATATTGTTCGACTGTTAAAACGGTCAAGGCTGGTGC
CTCATAATGGGCCCATACCGGCAAGGGTAATGGCACACCCCTGATCCAGGGCCCTCGTGCC
TGCCCTTGTGGCTGGCACCATGTTTCTGCTGTTTCGAGTCAACTTGGCCATTCACTTCCCCAA
CGGCCCACTGTTGGCACAAAGCCATGCTCATCCCTGCAGCTGCTTATTGGCTCCACAGGGCT
CTCTCCGTGTTTTATACTCTTCTCTCGCTCCATACAATGAGCGCACCAAACAAACAAAAACT
TTCCCTCTTCGGAAGCTTGGCACAATTTACACGTCCGAAGTCCAAGCAGAACTGGGTAGTG
GAATG

325. >MSTRG.6803.1 Gene=MSTRG.6803
TCTTTTCGTTTTTTTAAATTAGTGCTTGATGCAGCGTATAAAAAGCGTAAATATATCAATATT
TGATGTTGAATTAAATTTTATTATGATCGGACTCCCATTCTACTCGTATTTTATTATATTACCG
AAGCTGTGCTTTGGTTTTTCTATAATTTTTAATATTCGTAATATTCGAAAACACGTGCATATG

TAAATTTATGTGTAAGTAAGAATCTATCTTGCAGTTCAAACATTGAATACTGCATGTGTTTCT
CATTACAGAATTATTGTATACAATATACATATATATACGTATATAGTATATATAAGTATATA
TACATATATACACTTATACATCATCTATTGCACTTTTACATGCTTGTAAAACTAAATAATTAC
TGCTGGTACAGTACCGTATGTAAAGGTCCTTCTACGAATTTCAGTTTCTCGTCGTATCGAAG
TTGAATTGTTTCTAGATTTTACATCAATATTTCGTTGACTAATATTTAATTTAGATTAAACAT
ATTTCATGAAATATCATAAAAATATACAATTATCTACAATTAAATAAGTGAAAGACAATCA
GATAAGTCTTCTTCCACTATATGTTTAAAAAAAAATTATTTTAATATTTAAAACACAAGATA
AAATGATCAAAGAATATTTAGAATCTATAAATATCCATTGTTGTATAGATTGCAGCTATTTT
TGTTTTTTTTGTTTTTTTGAACGAAATACTCATATTCCTCGATACACATCAAACAGAAATAC
GTAGATAAACCAGATTCATGTAGTTCCTTCTCGTCAAACTGCTCGTTATAATTTAATTGTAAT
ATTTTGTAAAAGACTTACCTTGATATATTCTTTCGTTATTCATTATATTCTTTAACATAAAAA
GATCAATGCAAGTCTTCAATAATCGGAAAATCTTTGGATCGATCATTCTACAATATAATTGT
TTTCACTAAAATATTATTGGAACGTACAAATTTTTATTTAAGTAAGAAAAAAATACGATAC
GCAAGAGGATAAAAAGAATATTTCAATTAATTTATATAATTTTTTGTTTACTTTTTTGTTAAT
TTAAATTTGAAACACATTGTAGCAAGATTATTTATATAGTTTAAATTCCTTGCTATGCAACTG
ATTAACTTTAGTCCAATATAAATGCAAATATACATTTACGTATACGTACATACTTATATACG
TATATGTATATAAAAAACATATATACATATGTCTATTTTAAACATGAGAAGTGAAGCAGGT
ACAAATCGAGCATAGTATCGCGTTCCTTCAGGGTATATGTTCTTTACTTAAGAAGACGATGG
TAACAATGATAATGACAATGAAAAAGCAAGTATATAATGCTATAATGTATTAATATTTTCA
TTATGATAATGCAACATAACATATCAATATAAATACATATCAATAATTTAATCTAATTTGGA
GATGAGTGTCGAATACTCTCTCTAGCAGTTGGTTCTCCATTTTAATCTTTAAAAGTTAGAGA
ATGTTCATAGTTTTCACAAAGTTTACAAATTTGGAAACTATAATAAACTATATTAATTATAT
CAAATGCAAAATGGAGAACAAACAATCAACTCTGCTTTAGAGAGACTTCAATATATGATG
AAGTGTATCTCTATTTATTAAGCGTAATATCTTCTAGTCAATCAATGATATATCTTCTAATTA
ATAATAAATAAGTCTATACATATATAATTACATATCAACAGAGGAGTCACTTTTATCAGAT
AGAAAATATATTATTTTGATATATAAAACAAAAGTGAAGAAAAAATGGCAAAAAGAAAG
AGATGTAATGTTCCCTCTAGATGTCAATTACGGTTCAATGTAGGGCCTGGACCGAAGACAG
AATCCCGAGGGAAATTCGTGTAAGCTTTAAGAAAAGCTTTTTCTTTTTAAAGGATTAAGACG
AACCGAATCTCCGCCTCGAAAACGTCTTGATTCTTTTTAATCTACTCCTCATCTTCTTCCACT
TTTCTCCTTTATCCCCAATTTTGATGTGTTATATTTAAGATAATATATCATTCATCTCTGGTGG
CAATTAAAATGAATCTAGACGCGTAAAATGTGCGGCGCAGTACATTTTCATTGTTTTCAAAC
GACAGATTCTAAGATGCT

326. >MSTRG.6803.3 Gene=MSTRG.6803
CTCGTATTTTATTATATTACCGAAGCTGTGCTTTGGTTTTTCTATAATTTTTAATATTCGTAAT
ATTCGAAAACACGTGCATATGTAAATTTATGTGTAAGTAAGAATCTATCTTGCAGTTCAAAC
ATTGAATACTGCATGTGTTTCTCATTACAGAATTATTGTATACAATATACATATATATACGT
ATATAGTATATATAAGTATATATACATATATACACTTATACATCATCTATTGCACTTTTACAT
GCTTGTAAAACTAAATAATTACTGCTGGTACAGTACCGTATGTAAAGGTCCTTCTACGAATT
TCAGTTTCTCGTCGTATCGAAGTTGAATTGTTTCTAGATTTTACATCAATATTTCGTTGACTA
ATATTTAATTTAGATTAAACATATTTCATGAAATATCATAAAAATATACAATTATCTACAAT
TAAATAAGTGAAAGACAATCAGATAAGTCTTCTTCCACTATATGTTTAAAAAAAAATTATTT
TAATATTTAAAACACAAGATAAAATGATCAAAGAATATTTAGAATCTATAAATATCCATTG

TTGTATAGATTGCAGCTATTTTTGTTTTTTTTTGTTTTTTTGAACGAAATACTCATATTCCTCG
ATACACATCAAACAGAAATACGTAGATAAACCAGATTCATGTAGTTCCTTCTCGTCAAACT
GCTCGTTATAATTTAATTGTAATATTTTGTAAAAGACTTACCTTGATATATTCTTTCGTTATTC
ATTATATTCTTTAACATAAAAAGATCAATGCAAGTCTTCAATAATCGGAAAATCTTTGGATC
GATCATTCTACAATATAATTGTTTTCACTAAAATATTATTGGAACGTACAAATTTTTATTTAA
GTAAGAAAAAAATACGATACGCAAGAGGATAAAAAGAATATTTCAATTAATTTATATAAT
TTTTTGTTTACTTTTTTGTTAATTTAAATTTGAAACACATTGTAGCAAGATTATTTATATAGTT
TAAATTCCTTGCTATGCAACTGATTAACTTTAGTCCAATATAAATATACAAATGCAAATATA
CATTTACGTATACGTACATACTTATATACGTATATGTATATAAAAAACATATATACATATGT
CTATTTTAAACATGAGAAGTGAAGCAGGTACAAATCGAGCATAGTATCGCGTTCCTTCAGG
GTATATGTTCTTTACTTAAGAAGACGATGGTAACAATGATAATGACAATGAAAAAGCAAGT
ATATAATGCTATAATGTATTAATATTTTCATTATGATAATGCAACATAACATATCAATATAA
ATACATATCAATAATTTAATCTAATTTGGAGATGAGTGTCGAATACTCTCTCTAGCAGTTGG
TTCTCCATTTTAATCTTTAAAAGTTAGAGAATGTTCATAGTTTTCACAAAGTTTACAAATTTG
GAAACTATAATAAACTATATTAATTATATCAAATGCAAAATGGAGAACAAACAATCAACT
CTGCTTTAGAGAGACTTCAATATATGATGAAGTGTATCTCTATTTATTAAGCGTAATATCTTC
TAGTCAATCAATGATATATCTTCTAATTAATAATAAATAAGTCTATACATATATAATTACAT
ATCAACAGAGGAGTCACTTTTATCAGATAGAAAATATATTATTTTGATATATAAAACAAAA
GTGAAGAAAAAATGGCAAAAAGAAAGAGATGTAATGTTCCCTCTAGATGTCAATTACGGT
TCAATGTAGGGCCTGGACCGAAGACAGAATCCCGAGGGAAATTCGTGTAAGCTTTAAGAA
AAGCTTTTTCTTTTTAAAGGATTAAGACGAACCGAATCTCCGCCTCGAAAACGTCTTGATTC
TTTTTAATCTACTCCTCATCTTCTTCCACTTTTCTCCTTTATCCCCAATTTTGATGTGTTATATT
TAAGATAATATATCATTCATCTCTGGTGGCAATTAAAATGAATCTAGACGCGTAAAATGTG
CGGCGCAGTACATTTTCATTGTTTTCAAACGACAGATTCTAAGATGCTTGTATACGCCGCAT
GTTTTTTTTTTTTTTTAATCTTTATTATAAATAATCGTCCTTTTTTTTAATTCCATTTAAATTAA
AATCAATTTATCATTTCTTTTATTAATTATATATTATAAGAATTATAATATAATATTAATTAT
ATATTATAAGAATTATAAGATATATTTGTTATCTTAGCATCTATGATATTTTTAATTAATTAA
TACACTGTACAAGATATATATCATAAAGACAGAAATTAAAGTAAAAAACAATAATTTTAGT
TGAATGTGTAATTTATTTTATTTAATAAAGCATTATAAAAATTATAACAATTGTAATAGAAC
ATCTATATAATCCGTCCATAGCATTCAACTAAAAGGATTATTATTTGAAATAAAATAAAAA
AAAATTCAATTTTCAATTTGTATTAAATATTTAAAAAATAATTTTTTTTTTTATTATATAAAAT
CTATTACAGCGTGTACGAGCATTATCAGATATTTCTGTCAATTGATGAGGTGTACAGCGTAT
ATATATTACGTATAAGCACTTTTAATTATCTTAGTTATCAAGATTTAAATAGTGTGGTAATGT
AATAAAAAAATAACACACCATTGTATCTATATTACTTTCAACTTCTGATTGTTCTACTGCCA
AAGCATTCTTATTATACGGCAACTGCGACGTACCCGTACATTTGTAAGCAATCTTGCTTCTT
AAAATTTTGTACTTCTATCAACTAAAACAAAATACAATGTTCTTGGAAATAAGTTCAACGC
GCGAATATCATTAAATAAATGTTTCTTCAGTTTTATTTTACGAAATTGAAATTACCTGA

327. >MSTRG.6803.4 Gene=MSTRG.6803
CATTGAATACTGCATGTGTTTCTCATTACAGAATTATTGTATACAATATACATATATATACGT
ATATAGTATATATAAGTATATATACATATATACACTTATACATCATCTATTGCACTTTTACAT
GCTTGTAAAACTAAATAATTACTGCTGGTACAGTACCGTATGTAAAGGTCCTTCTACGAATT
TCAGTTTCTCGTCGTATCGAAGTTGAATTGTTTCTAGATTTTACATCAATATTTCGTTGACTA

ATATTTAATTTAGATTAAACATATTTCATGAAATATCATAAAAATATACAATTATCTACAAT
TAAATAAGTGAAAGACAATCAGATAAGTCTTCTTCCACTATATGTTTAAAAAAAAATTATTT
TAATATTTAAAACACAAGATAAAATGATCAAAGAATATTTAGAATCTATAAATATCCATTG
TTGTATAGATTGCAGCTATTTTTGTTTTTTTTTGTTTTTTTGAACGAAATACTCATATTCCTCG
ATACACATCAAACAGAAATACGTAGATAAACCAGATTCATGTAGTTCCTTCTCGTCAAACT
GCTCGTTATAATTTAATTGTAATATTTTGTAAAAGACTTACCTTGATATATTCTTTCGTTATTC
ATTATATTCTTTAACATAAAAAGATCAATGCAAGTCTTCAATAATCGGAAAATCTTTGGATC
GATCATTCTACAATATAATTGTTTTCACTAAAATATTATTGGAACGTACAAATTTTTATTTAA
GTAAGAAAAAAAATACGATACGCAAGAGGATAAAAAGAATATTTCAATTAATTTATATAAT
TTTTTGTTTACTTTTTTGTTAATTTAAATTTGAAACACATTGTAGCAAGATTATTTATATAGTT
TAAATTCCTTGCTATGCAACTGATTAACTTTAGTCCAATATAAATATACATAAATGCAAATA
TACATTTACGTATACGTACATACTTATATACGTATATGTATATAAAAAACATATATACATAT
GTCTATTTTAAACATGAGAAGTGAAGCAGGTACAAATCGAGCATAGTATCGCGTTCCTTCA
GGGTATATGTTCTTTACTTAAGAAGACGATGGTAACAATGATAATGACAATGAAAAAGCAA
GTATATAATGCTATAATGTATTAATATTTTCATTATGATAATGCAACATAACATATCAATAT
AAATACATATCAATAATTTAATCTAATTTGGAGATGAGTGTCGAATACTCTCTCTAGCAGTT
GGTTCTCCATTTTAATCTTTAAAAGTTAGAGAATGTTCATAGTTTTCACAAAGTTTACAAATT
TGGAAACTATAATAAACTATATTAATTATATCAAATGCAAAATGGAGAACAAACAATCAA
CTCTGCTTTAGAGAGACTTCAATATATGATGAAGTGTATCTCTATTTATTAAGCGTAATATCT
TCTAGTCAATCAATGATATATCTTCTAATTAATAATAAATAAGTCTATACATATATAATTAC
ATATCAACAGAGGAGTCACTTTTATCAGATAGAAAATATATTATTTTGATATATAAAACAA
AAGTGAAGAAAAAAATGGCAAAAAGAAAGAGATGTAATGTTCCCTCTAGATGTCAATTACG
GTTCAATGTAGGGCCTGGACCGAAGACAGAATCCCGAGGGAAATTCGTGTAAGCTTTAAG
AAAAGCTTTTTCTTTTTAAAGGATTAAGACGAACCGAATCTCCGCCTCGAAAACGTCTTGAT
TCTTTTTAATCTACTCCTCATCTTCTTCCACTTTTCTCCTTTATCCCCAATTTTGATGTGTTATA
TTTAAGATAATATATCATTCATCTCTGGTGGCAATTAAAATGAATCTAGACGCGTAAAATGT
GCGGCGCAGTACATTTTCATTGTTTTCAAACGACAGATTCTAAGATGCTTGTATACGCCGCA
TGTTTTTTTTTTTTTTTAATCTTTATTATAAATAATCGTCCTTTTTTTTAATTCCATTTAAATTAA
AATCAATTTATCATTTCTTTTATTAATTATATATTATAAGAATTATAATATAATATTAATTAT
ATATTATAAGAATTATAAGATATATTTGTTATCTTAGCATCTATGATATTTTTAATTAATTAA
TACACTGTACAAGATATATATCATAAAGACAGAAATTAAAGTAAAAAACAATAATTTTAGT
TGAATGTGTAATTTATTTTATTTAATAAAGCATTATAAAAATTATAACAATTGTAATAGAAC
ATCTATATAATCCGTCCATAGCATTCAACTAAAAGGATTATTATTTGAAATAAAATAAAAA
AAAATTCAATTTTCAATTTGTATTAAATATTTAAAAAATAATTTTTTTTTTTATTATATAAAAT
CTATTACAGCGTGTACGAGCATTATCAGATATTTCTGTCAATTGATGAGGTGTACAGCGTAT
ATATATTACGTATAAGCACTTTTAATTATCTTAGTTATCAAGATTTAAATAGTGTGGTAATGT
AATAAAAAAATAACACACCATTGTATCTATATTACTTTCAACTTCTGATTGTTCTACTGCCA
AAGCATTCTTATTATACGGCAACTGCGACGTACCCGTACATTTGTAAGCAATCTTGCTTCTT
AAAATTTTGTACTTCTATCAACTAAAACAAAATACAATGTTCTTGGAAATAAGTTCAACGC
GCGAATATCATTAAATAAATGTTTCTTCAGTTTTATTTTACGAAATTGAAATTACCTGAC

328. >MSTRG.6840.1 Gene=MSTRG.6840

CACAAAGGGTGAGTCTCAGTTGCCATTCGGACAGCCTACTATACGGCTGGGTTTCGAAAAC
CCTATGCGCGCGGGTATATATGCTGATCGGCGCGCGCGCGAGTCTCGTCGCATACGTACGC
ACGATTAGATAAGGACGATCCAGGCATGTGTAGACTAGAACGTAGACGTTTGCTACTTTGA
AGGATCAAGCTTGGAAACGGGGACTAGTAAAAGTAAAAATTTGGAAAATTTTGCAAGAAC
CTGGGAACAAGAATCGATCCTCGATGGAAGATTCGCCTCGATATCGCCCGTCTCGTTAATC
GCGATTCTCTCTGTTCGGAGTTGGCGAAACGTGTCCGGGACGCGTTGCCCGAACGCGTACC
GCAACCGGTCAGCGCGTGTGCACCCGACCACGATCGATCCTCGGGGCGATACGATCCAGA
AACGCGATGGACGGAACCACCCTGCTTAACCATCACAGATAACGAAGATCGGCCACGTTT
CATAGGATATTAAATCTTTATCGATTATCTTTACCCAGAAGAAAAATTTTCACGCATTGGAT
ATTCGAATTTTCCGAATTTGAATCATCCTCGAAGTTGACACCGCTACCCTCGAAGGAAGATC
GCGTCGATCGTGAAATCGGACGATGAAGATGTGATCGAGAATTTTTATCCGACTACGTTCC
CCTGTTTAAGGGATGTCCTCCTCCGCGGGCACGAGCTGCACCGACGCCGAGGAGGACGCCG
AGGATGTGGAGAACGAGGTGGCAGGTAGCGCGCAGTCGTCCAATTACTTGCCGTCGGGTAT
CCGTGATAGCGCGCGCGATCACCTGGAAGGACTGCGGTTAGATGGGTCATCGATCGTGCGT
CCGCCAGCCGAGGACACTTCCGCGAGCCCATCGTTGTCGTGGTCGGTGCCTCGATAAATCC
GGATCATCGCCCTCCCCCACCTCCCCACCCGTACCCCCCTCTTCGCCCGCGAGGAAGATCTT
TCACCGAGGCCCAACGCCAGCTTCCCATCCTTCATCACTGGCCACATCCACGAAGGACGCG
GAGCTGGAGCACGCGACAAGCTGGGGGAGGTGTGTGAACGCTGGTCGTTGGACCAACGGA
GGACCGTTCGTGTTCAGGTGACGATAGAGACGTCTAGGTTAAAACCAGGCGGATCCTCAAA
TCGTCCGAGATTCGTTCCTGGATAATTCGCGTTCTACCACCCGTTGTATTTCTTCTTTCTTCTA
TTTTTCTCTTTCTTCCTTCCTTCCTTTCTTTCCTTTTCCCTTCTCGTATCTTCTTTCCGCGATATC
CGATCTGATAATTCGTGTATTATATGTGTGTATATATATATATATATATAGAGAGAGAGAGA
GAGAGAAAGAGAGAAAACACTGGACATCGCGCTAGAGAGTATCGTGGAAGGACTGTGGA
GTGACGAGTTCGAAGGTCCCGGTTCACGCGCCATATAGATCGTGCGAACTAACTCGGCAAG
GATTAACGCGAATGTGCGCGGGACAATTGGTTGACGTTTGATATATATCGTACAGTGTGTTT
GGTAAGGGAAGACGGCAGAACGCCACAGTGGTTGTTCTCGTTTGGACCAGTTCCAGGCGG
ATGTGAAACCGCTGCCGACAAGTGATATTACGGCCGATACCACAATGGTGAACTATCCTCG
GATCATGACATTCAGTTAGTGATCGTGTGCATCCCCACGTTGTCGCGCGTGTGCATTTGTGC
GGATAAAAAAAGAAGGATAGAAGGGAGGGGGAGGGGGGAGGGGGGAGTGATCGATGAA
AGAAGGTTGTCGTTGTTGGACATGAGGCGAGGAAAACAGCACGTGTTCGTCGTAGCCAGA
GGCTGGACGAGATCCTGATAGCCGCAGCGACCAGGATCCGGGATCATTCAAGGAAGAAAG
GATGCCAGGGGGGCGCAGGGGCCTGGTCGCCCCGCAAAACACATTCCTCGAGAACATCAT
ACGACGGAGCAGCAGTCAACCTGACAGCAGTTTTCTCCTGGCGAACGCTCAGATCGTCGAC
TTCCCTATTGTCTATTGCAACGAAAGCTTTTGCAAAATCTCTGGCTATAATCGTGCCGAGGT
TATGCAAAAGTCCTGCCGCTGCGGATTCATGTACGGGGAGTTGACGGACAAAGAGACGAT
CGCCAGGATCGAGGAATGCCTCGAGGGCCAGATTCACGATCAATTCGAGATCCTGCTGTAC
AAGAAGAACAGTGCCCCACGAGGTATGTCTAGCACAGGTTCGCAAAAGAGTGCTCGAAAA
ACCCACCAGTAAGTCACCCTCGAATCTAACTATACAAAACACCATTTATATATAATTATAT
ATAATTATATATGTATATGTATATATATATATATATATACGTACATATATTTATATAGAGGTT
ATCTTGTTATTTGTAGCACAATTCAGGCAAAGAGAAAGAGTGATTCCTCATGAAAGAGTGG
GTAGAAAAAAGAAAGAATAAAATTCTTTTATTTGAAGCTTCGTTTTTAAGAGAATTTAGTTT
GAAAATATATGTGAAATTGACTAGATA

329. >MSTRG.6859.5 Gene=MSTRG.6859
CTGAAATAATATAAAAACAATTTCGAAAAAATATAATGACATAATAAAGAGATTAAAAAG
AAAAAAAATATAGTTAAAATGGTTCTTGCGTCAATTGTAGGTGAAAAGCTTCCCTTTTAGA
GACCGATTTTAATGCAATAAAATCCCCCTCTCTTTATACAGGAACTGAAATATAGGTAGTTA
CCGCGGAGTGGTCAAGTTCAAATCTTGAGTTCAGTCGGCCATCAGCTACGATTGACGTACA
GTTATTTCATATATAGCTTTTCTCAATTATTACAATTAAAAATTAATATTCGTGAAGCAAAA
ACAATGAACATTCATAACATCCAATCGATTTACTTAAATTTTAATTTAAAAGCTAGATTGTT
CTGTGGTCTAAATGTGTTCAAAATTAATTATGAAAAATGATTTCATACATAAAAATTTTCT
GTTGCATTTTTATATTAATTTTACGCTAATTTTTATTAAAATTGTTAATTATTTTTATGTTATCA
GTCGCAAATAAAATGCAGAGTATAACATCAAAATGGACAGAATGAACACAGAGTCGAAC
GAAACACAGTAGCAGCACAGATAGGCTCAAGTGGCCAAGGGGCACTTGAACTGACGAGAT
AATGGACCGATTGTCGAGAAAGAGAAAGAATCAAACGATACCGAGCACTATGCGTGATGG
TTGTTGCAATGCAATCGTGATCATTATCGCACTTTCTTGTTTCTTTACTTCCAATGACGCACTT
GTGACGTCAATCGAAAGAGCAAGAAGCAACCCTGGCATGTCTTCTGTGCATCTTTCTCTTCA
CATTTCTTCGACCCGGATATACGTTCCTTTGTTTTGTTTCCTAATTTGTTCTTTGATTCTTCCTG
CGATATCTATTTTTTTTAAAAAATAACTTTTGTGCAT

330. >MSTRG.6862.4 Gene=MSTRG.6862
CGTTGATTTCAATATTTTTATTATATCCTTCTCCCTATTTGCATTCTTTGTGCATTTTTTTCTAT
AAATTCTAGATTTTTATATTATATTATGTCTTTATCATAATAATTATATATATATATATATACA
CATTTTAATTTCATTGTGCCACTATTAATATTACAATCTTATTCCGTGCCGAATCGATAATAT
TATAAATGATATTAAAATGATTGAAGAGTACAACAAATCATCATGTCGAGTCCGTCGTTGA
AAGACCTGCCAAAAGTAGCCTTAGATTTGAAAAGCGAATTGGAAGGTTTCAATCATGGCTG
TATGAAAAAAGCCTCAACTGCTGAAAAGAATGTTCTACCTTCTGCTGAAGGTATAATATAA
ATTTCATTACCTTTTCACTAAAATTTTTGTTGCATTATTATTTTAATGTTTTTATGATTTAATAC
CTATAATATTATAGACATACAGAGTTCTAATTTCTTGTAATTTGTTTCGACCGATTATAATGC
ATGTAATATTGTAACATATAGACGTACGACAAGAACGACAGCATTCTGAATTAATCCACGA
TGTGGAAACATTTAAACCAGATCAATTGAAACACGCCGATACGAAAGAGAAGATCATATT
ACCCAATGCCAAAGGTATCACTGTTTATTTGATTCCTAGGGGAATATTTCATTCTCAGGTTCT
CTACTTCTTCCTTGGCCGGTAACAAAAGGAGGGTATTTTGTTGCTTTTGGTGCTTCGAGGTGA
CCGAAACATACGTGACCAAACCAAGCC

331. >MSTRG.6897.1 Gene=MSTRG.6897
AATGTAATTATAGTTTGCATTCTGTATATGTTACATGTTATATGCACTATATTGATTTGATAG
AGTGACGTAATTGTTGAGAATAGTTTATTACATTATTATTTAGATCAAGAAATTTATTCAAA
ATGTGGTATATGACTTTACCACCAATAATTATAATAGCTGTATGTTCAGCATTACCAGCATA
TGTGGCACCATATATAACTAAATATTTTCACGGAAATACGCAAACGAGACTTTATACTACT
CATTTAGAATTACTAGGATATTCTAGAGATTATGAACTAGGTGGTAATAAGCATTGGAATTT
TAAGGGTTTGGAATCCATTCCAGATAAATAAAAGCATATTGGCTTATGCTATATTAAATAAT
ATTCATTATTTTAGTGAAGTTATTATGTAAATCATATTGTAACATA

332. >MSTRG.6912.1 Gene=MSTRG.6912

AATAATAAATTTTTTTATGTAATTTTAAATTATCATTAATAATATGTAATAGTATAAAATACT
TTTGTCGAAGGTTACTTCATTGCTGCTTTTCAATAATGGTATTTAATAACATAAAAATTGTTT
TTGGAAGAAATATTTATTATGTCTGATTTTAATAACTATAAAATATGAAACTTTTGCCTACTA
TGTATAATATTAAATTGTAACACTTCTGTTCCTCTGAAAATATAATTATTTATTTTTTACAGG
ATAGTGTACCACTTGGCATTGATTTGCTAGCGACAATCGTGCAAAAAGTTGAGCTTTGGCAT
ATCTGACACATTCTGTTTATCATTTGGTGTTACATAGTATTTTGGAACAATTACAATTAGAAC
TTATTGGAAAAGTATATGGAATCATGATCGCCCCCTTCTCTTAATATAAAGTATACAATTAA
TGAATAGCTGCATCCCAAGGATTTTCAACCAATATGGAACATTCCATCCACTGATCGAGTTT
TATGGTAATTGAAATTGAAATTAAAGAATTAAATAATTAAGTAATATAAAATATTTTTCATA
AATTATATACTATAAATTAGAAGCATACAAGATTGAAAATCTTATTCCTTCTGATTCTTCAT
AATTTTTCATAATGAAAAATTGTATTGTATTAATTATAACAAAATTATGCGATATGATAAAA
TATTTTAATATTAAGTCTTTTAGGATAAGTAAAAAAAACATATTTTTTTGTTTCTGGAGGA

333. >MSTRG.6920.10 Gene=MSTRG.6920
CGAATTTTTTTTTCTTTTTTTTCTTATCGAACGGAGAATTTGGAAAGCCAATGAATCCCACTC
GAGGAATGGAAATTGCTCCGCTCCCTCCGTTTTCGCGTGTCTCGGTTCGAGGTAAAGGGTGC
ACCGCGCAAGAGGCTCGACCATTCTCGATCCCACGGCCCATCATCCGCCAACCATTTCATC
CATCTTGTTTCTTTTACTTGTGAAATTGTCGCTTGCGCCCCATGTTGCATATCTTGAAGTGGC
ACAACGTCATATAGCTGATCATGGTACGTTTGCCGGAATGCTGCGGCTCTTGTCTGACCACG
GCATTCGGTGTCGGTAATTTCTCAGCGTAGTAATCCGAGTCCGATTGTATCTCCGATTCCTTC
CCCTCCATTATTATCGACTGGATCTTCATACGTTCCTTGTCCAACTCTTTCTCCCGTTCTGCCA
CGTACTGTTTCTCCTCGAACAACTCCTTCAGCTTGTTGAGCAATATGAAATCGTTGTCCGAG
GTTTTGCCGTAGAACTCGGACGGTATCGGTTCCCCGCCGACGGACGACGGTGAGAGATGGA
AGAACAACAACAACAACAGTGCCAACAGACACGGGGCTGTCCTACGTTTCGCGCTTGACC
TTCCCGTCATTCCCTCGGTCCTGTTTTTATCGTCGACTGTCGAAAGTCTCTCGGTTGTTACGCT
TTTTCCTTTTCCCGATGTTTCGAATTTGTCGTCAGTCACGGGAGAAAAAAGTCTCGATTGAA
AATTCGATCGATTTATCGTCTCGTGATATCATCCAACGTTATCACTCGAATGATCTCGTCACT
TATCTCGTCGAAAAATGAAAAACCGTTTTTACCGTTTCTTTATTATTTTATTATTATTTTTTTTT
ATTATTATTATTATTATCGTTGTTTTTTTATTATTATTGTTATTACTTGTCTCGCGGAAAGCGT
GAAGGCTAGGTGGTTCACTACACATTCACCGTTTCGCGACTATTAGTCTCGAAGGTTGGTGC
TCTGGTGTCTTATAAGCTCAGCTGTCCCCACCTAATCAAGTTCGGTTCGTAGACGCGGTGTC
GAGCCCCTTCTCGTGCAGCCAGCTTGGCTCCAGACGCTCGCCCATTCTTTCCCTCTTTCTTCA
TGCGCCTCTTTCTTCCCTCC

334. >MSTRG.6981.14 Gene=MSTRG.6981
CTAACCTCAGTGTGCGGAGAGCGACCGCAGTGTACACCATCTCCACGTTTTGGCAAAGAAG
AGGGCAGTTATCGTTGCCGAGTGAACGAGTTTCGATCCACCGTCGTACTTTCGATCAGCTGG
ACCAATCAGTGTTATTTATCTCGCCCGCCTTATCTCCCCTGCCTTCTCCGTTCCACTCGGATTT
CCATTCGCCGGTGAACAGACTTGGCGAATGAGACGCACGTACGTTGGGCGAACGTACAGG
ATTCGATCGAGTGATATAATAATTGGAACGCGCCGGAAATCGGAGCCCGCGATTCCATCCG
TCGATGGCGAGTTTTCCCTCTATGGAGGTGGAGGAGTTTCGTTCGTAACGCGCGAGTGACG
GAATCACGAACGGAATCGCGTTTACACGATTTTGACGGACGAGGTGACCGATCTCCTTTTCT
TGATGAATACGACGACGTCGAGAAGAAGCACGGTCGGTAGCCACGCGGTTGACGCTCTCA

CCGCCGGTTGCACCTCCGAAGGGGTTGACACCTAGCACGAGGAGCGTCTGCTGCGCAGCTA
GCGAGAGAAACGATTCCGAAGGGATCACCATCTTCCTTCGCATGGTGATACAGTGGTGTTA
TTGTTATTGTTGTTGTTATCGATATTTGGTGATACACGTGCCCCGGTGATACACGAGTCAAA
CTGACTACACGTTTCTATGTTTACGTAATTTCAAATTGTCGTTTCGAGAGCGTGGAAAGGAA
GAGTCGCCCGCCTCCTTCCGCTTTCCTTTCTCAAGTGTTCTTTTCCGCTGTGTTTTACCGTCGT
GGGTCGGGAGGCGCGCGTGTCGAACGACGTGTCGGATACCGGGCTGGCGAGATCGGCAAA
ACAGTTTCAGGCTTTCGATTTCGAAGGAGTTTGGAATCGTGTGATCGTCCGCGCGAGATCGA
CGACACGTGGATTCGGATCCACAATTCTCGTTCCTAGCTCACGTGGTCGTCGAACTACTACC
GGCGACGCTCTTATAGTGGCGCTTCCGCGATATAACGTATCACGGATGTTCCTCTCGAGTTG
AAACGAGTTTGCTCGTGATCAATTTGAAAGTTGTCGGGTTATCTGTGTGCTCGTTGAATTTTT
ATCCATGTTCCGTATCGACGTTGCGACAACATCGGACGTGATCGATGAGTGTTCGGCCGAG
TGAATAGGAAAGAAAGAAAAGTAGGAAAAAGAAAAAAAAAATAGAGACTCTCCGTCAAA
CGATTCTTCCGTCCTGTTGTCGAGAATCGAAACGCGTGTGTGCCACTTTCATGTTGCGAATA
GAAGGAGAGGGATGACGGTGCATCATCGTTGTGCTGCAGACACCGTCGAAGAGGAGGAAG
AAGACTCGTGAATTACGTAAACGACGATCATCGGTGCTGGAACGAGACATCGTTAATAGTA
CATCGTTGTTTGCGATTCTTCGTAGGATTTTTCGGATCCGCAAGCACGCGGAATCAAGCAGG
CAGCGTGGAGCGCTCGGCGATCGTGTGGCCGCGCGCCGCGGACCTCTATCTTTATCAACCA
GGCTGTGCATTTCTCACCTACTATAGTCGTGACAGCGCAATCAGTGCCCAGAACGCTCTGC
ATGAGAAGCGGACTTTACCAGGGGGTGTGACGGGCGACCGGCTGGCCGGGGGCGCCGCAC
ACCTCCTCCATCTCTTCTTCTTCTTGCCACGACCACAACACACAATGTAACAACAACTACCA
CTACTACTAGAGTCGTCCGCGTGAACCACGTTCTCAATGTACTCGATGTGAACGGTGGTGG
GATGGGAAGAAAGGACGTGGAGAAGGGAAAGAGAGGGCGTCATGCTGCTCAAGAACGTT
TTCGATGTATGTGAAAGAAAGAAAGTGTGGACCGAAGGAACATTTATTTGCTGGCAGGAAT
CCTCCGCAGTTTTTCAACCAGACCATGCGGAGTTTTGCAAAAGGCAATTCGATTTCAAGCCC
ATTCGGACAATATTAAAAGGTAATTGGATTTCGACCTGGCTAGTTGTTGCCAGTGTGCAACT
CGCTCGATCCCAGACTGGCAACGGGGATGGGGGACAGAGTAAATGGTGTAAGGGGTGTCG
GGATTGGTAGAAAGAGGAAGATAAAAGTAGGGAGCCTGTACGCGTGTTCGCAGACCAGAA
TAATGGATAGTCGTTAAATTTAATTGGCACATAATTATCGGGAGTTCCGGCACCAAGCAGA
TCGTTGCACGCCACCGATCCCCGTAATCCCGTTACCTTGGCTTAATATCGAGAAATATAAAA
ACCACTGTCCATTTTACAATATATCGTCCATCATCGAACAGTGAAAAACTCTATTTTCGTAA
ATCAAACCTGGATTGATCTAATCAATCCTGTGTGACTCTTATACAAATATAAAGAAATACA
GATAAAGTCTTCATTAGCAGCTATCGCTTAAATTCGCAATGGAACTTTGACTTTTTTGCGTG
AAAAGCTCGAGAAAAAAACTTTAGTATCAGAGAACGGTCAGGATATACATATAAAGGAAAG
TATAGCATTGAAATCACTTTTCGATATCTCGATCGGTTATCGAGATAGGAGTGTTAAAAGTT
TCCATAGTATTTAAAAATCGTTTTTCCAAGAATCGCGTCTCTCAGTTAGTTTAGAATAGGTA
AAGATAGGTCAGTGCAAACAAGTGATTGAAAATCATTATTTTCTTTGCATCATGATATCTCA
GGAATCGGAAATTATATAAATATAAATGAAAAAGCATTTTAAAGGGCAAGATTCCGCGCTT
CCAATGATCTTTCATCCATTGGCCGAAAGTTATTATCTTCGGTTTAGTTATAACGGCCTAAA
ATTTTTCCAATTTTAATAGAATTTCTAAGGTG

335. >MSTRG.7015.1 Gene=MSTRG.7015
CTGAACTAGATCTTTATTAAACAATAAATTAATGAAAAATTCTATTCTAAATTTTTTAAAAA
CCTATTAAATTATTATTTATATATCATATAAATAAATAAAATTATGTATATTTGAGCAGTCTA

TTAAAAATATAATGTGATTTATAGATTGAGAAACATTGCATTATAGATTTTATCAGAAAGCT
ACTTAAATTAGAATGATAAGGGAATATTCGATGAAAGGATACATTAAACTTCCTCTTAACG
ATTTGCAAAGATTGACTTACGATAAGAGATACTATGCAATTGTTTGCGATATCTTATATATG
ATATACATATTATACTGCAATAAAAGCCACTTCGAAAAGACTCGAGCATTTCTAAGAACAT
TTTTCCGATCTGAAAAGGAAATGGCGGTAACGTGATACGCTTAAGTTATTGAAACAAGTGA
ATTCTCATTTTTCGTGATCACCTAGGCAACCTAGGCAACTAGCATGGCAATTAAAAAAATG
GAGATATAAAAAACAAGTCGCATAATATCTGAAATCATCGTTTCGAATTATTAATTATTGTA
ATTACCGACCGAGAGTAGGTGTATTTTTGTTAAACCTATGGGGAATAATGCTAATTTTCAAG
GCTATAGTTTATAACGTCGAGCGTCGATTTATTCGAAAAAGCGCGGGAAATAGCGAAGTTC
CGATGGAACTTATCGCGCTAGGGGAATCCCACTAAAATGAACTCGAGAAGAAATAAACAA
TAGTAGTGTATTACGCGCAACCATTATTCAATGCATCAAGTGTGAATAAAATTAATTCACAC
GATTGCATCGAATAAGTTTCATATTGTATTTAAACGATAGGATACGATCAATTCGTTACTGT
AGATATATAAACTATCTCTACTTGTTGTATACGTCAGAATAAATACTTAGATCTTACAACTG
TCAGTTTACAGTCAGTTGAAAAATAAGGATCGATAAAAGAGGCAAAATGTTTCGATGGGA
AATATCGAAATATAGGGTAAATGATTTCAGAGAGATCTGTTCTCTCTTTCTCTTTTTCTCTAT
CAATTGTGTCACTCTTGAAGAATTCACGCAATCTATACTAAGAAAGTATATAAGAAGTATT
ATAGTTTACTGCTTCCATACTGATTATATTATATACATACTTCGATTTGATTAATTACCCAAT
AATTCACAAAAAGATTAGGTACACGATATTAAATTGCGAATACATATTAATGTATAACGAA
TATTCGGTATACTGTCAGACATAACAAGAGAGCCATTAAGTATAATAATATTTTTGTTTTTA
AATTTACATTCTGGGCATTTTTATTCAA

336. >MSTRG.7023.4 Gene=MSTRG.7023

CGTACCATCATCCAGGTCCAGAACCTATACCTGTTCTTTCTCTCTCTCTCTCTCTCTTCTCTTT
TACCACCGACCGACCGACCGACCTAACCCAATCGACCCATCCACCGACACCACAACCTGG
CGAACTTATTCGATTGCCACGTTGTCGAGTCAAAAGTCGATCAATCAGTGTTTCACCGTCGT
TGTCGTCGTCGTGGTCGTCATCGTAGTGGTGGTCCTCAACGTTCGATCCATCGACACGCTGG
ATTAACCTTCACTGGCAAGTCTATATTTATTTATTTCCTTTTGTGTACACATCTTTCTTTTACC
ATTTTTCAACGGTTCAACGTTATCGATCGAACGCTTGCCGTGACGCGTTTACGCGACGCATA
GATCCTCGCATCAACTGCCATTGATTTCGAACCGTAGTCACGTGACTACGGTGCAACGAAT
CCTTACGCAGCCACCTGGTATTTCGCGACGTAGTCGAATCGGTGACAGTGTGCCGTGTGAA
ATTTACGCGTAAATTACATCAGGAATATTCGAAAGTGATATCGAGACTCGCTGTAATAGCG
AAGTTAAGTTTTTTTTTTTTCTTCAGTTGAAGCGTGATTTGTTCACGTGTTATTGCCGACTTAG
GTCCTTTGACAACAAATACACGTGTATACCTACTCGGTCTTATACACGGAATGGGAAATGG
GAATGTACGAAAATGACTGTACTTTTAAACACATGCAATCTTAATTTTAAAAGAGTAATTG
GTTTTTCGCTCGAACACCTTTAACTCCGCTGTTTCGAGGATGTTGAATTAAGG

337. >MSTRG.7023.12 Gene=MSTRG.7023

CATCTTTTGTTTGAAAAGAAAAATCCTCCACAATTGGAGAATTTTATTTTCCAACAAAAGTA
TGCTCGCGAAAAGATTGTACTTCAATAATATGCTACATCATAAGAAAAAGAAATTATCACC
TGGGAAGCTCTAATTCATCAAATAGCACCCAAGTGTTATTTTCTATAATTCTGTTGGTTTTCT
GCGTCGATCGAGACGAAGTAGAGGGGTCTTTGACTTGATAATTAATTAATTTTTGAGACAAT
AGTCTTCTTGTCCCTTTTCAATGAAATTGACCATCTTTTAGGAGATTCAATATAAGTTCTTCA
TCTGCTTGAAGCCATAATGGAGACTCGACAGAGATTAAGAGGAGATCTAGAATAAAATCA

ATATGCTGTTTTAATGTGTTCAAATTATCAGAGATCTTATCAAATAGAGTTTAATCAGTTTAA
AAACAAGATTCTTATATTAAAATTTGTGCCATTTTGTAAAATCTTATTCCATTACTATCATC
CATAATGATAATTAATCCAATCATTAAAGTTTTGTCATTTTCCATTGGTAAAATTTACTTAAA
ATCGTAATAAAAAGAAAGGAAGTTTTCAATTGTTAAAAATATTAATATTTTCTATTTTGATG
AATTTTCAAACAAATGAGACCTAAAAAAAATATTTTAATTATTTAATCCGATGATTAAAATT
TAATTTTAATAAATTAAAATATATTATATCATATTACATACAAAGATTTGAAAATTAATACT
AAAAAGAAGAGAAAGCATTCTTTTATCTCGAAATGGCCGTAGCTGGATCTCTCGTCGAGTT
CCTCTTTGAAAACATCTTAAAAAACCTCAACGCCAGAGTTTGGAACCGGTCTCGAATCCAG
TATGGCAGATCTTTAAAGAAAAAAAATAAAGAAAGAAAAAAAATATAAATGACAAAATT
GTCGAAAAAAGGAAAAAAATATTGTCAATTCGATTCATTAATCGAAGATAATATTCGATAA
TATTATAAAATGTCGAGTTGATGATAGGCGTTCTTCTATTGAAAGAACAAAAATAAAATTA
TGTGGAGTATAAATTATACTACTATATAAAAAAAAAAAGTGAAGAGAAAAATAAAAAGTT
AGAAGAGATAAAATAATGAAAAGATGTGCGAGCTTAAAAACTTAGTTTTCATCAAGAAGC
ATTTTCTTCTTTATCCTTTTTTTCATCTTCAATCTTCAATTCAAACTATTCTTCCGCCGTTTATT
AGATCTTTTTTCATATAGAAACTTCGTTTGTCATAACCTAGAATAGGGAACTATAAATGAAT
TTCTTCAATGTTGGATTGCACGAACTATTCTTATGTTAATCAATTAATAGTACACAATGAAA
CGTTAATAACGTTAAGAATGAGAAAAAAAAAAAAAAAAGAAACTTTAAAATTTCAATATAA
ATTATGAAATCTATGATACAGATTGTAAGAGTCAAATCGGTATATATAAATGACTCTGCAG
ACCAAGGATGTATTATTAGTTCATCATTTTTAAACTAGAGCGTGCTTTTATAATTCGCTTCAA
TGACATCTGGCGAGTAAAATAGGATTCAGATTTCTCAATTATTCTCTGTGTGTATATTCATTG
CAGGGTAAATTCAGTGTCTTCTAGAATCGACGAGCTCTGTGAACGAAATGAAAATAGTGAA
ATCAAGAAAGATAAGAATGGTTAAATGGAATTTTTCTTGATCTTTATCTTGATCGTTATTCGT
ATATATATATCCAGAGTTCAATATTAATTCCCAATACAATATGTGTTTCAATACACGGTGCA
CGATGGTCAGTACACATGCATATATGCATAAGCGTGCGTTTCGTTCGGTGTCCATTTTTTAG
ATGATTGTAATGATGTTCGATGATGATACGTATATTCGAAGCAAGACTGTCGAACAATGTTT
TCTGAGTTAATCTAATAATGATTAGATGCCGATGTTATCTTACTTAGATAGCAAAATAAATC
AGCAATTAAATATTTTTAAATGATTAACCCTTTCGTTAATATAAATATTTATTTGTAATTGAA
AGATCATTTATAATGTTCTCTTGATATTTACTTATTAAGGTTTAACAATAATTTCTGATATTAT
TAATTTAAGTCAGATTTTTGCAAATCTTTTAATGAATCCTTAATTATTCATAATTATTATTATC
GATCGGAATTTAACAATTTAAATTTTATTGCCAGAAAACATAAACATGTCAAAATAACATC
ACTAAAAAATAAGTCCATTTGCAACAATAATTTGTATTAAACTCGAGCATATTGAAAAGGT
TAATTATTCATTATGAACATAAATGTTGATATTTTTAAAAGAAAAATAAAAATGTTACACTA
ACTCAGAAAACATTATTTCTATATCGATCAATCGATCGTTCGATAAATCGTCGATGAATCTT
CGATTGAATCTTTGATGAGTTTTTGATGGAATCTTCCAATGTGTGCCAAAGATTCGTGATCC
GCGCGAAATCCTCAATAAAGCTCGTCGTTCTCGCTTAGAGAATATTCGCCTTCCGCGGATCT
TTTCATGTTCTTCCTCGTCCTCTTTACATAATCAGCGTATTTCCCTCGCAAAATTTGGACTATT
TTTTAGACGATTATTTTTCCCAATTGCGTGCGTCATTGCTGTTATTGACTTTTTAGCGAAGAA
AAAGATTCTTGATTGTGTCGTCTTAAAAAAAATAATTAGAAAGATCGACATTAAATCACAT
TTTCTTCTCTTCGTTTTAATGTCGAGGACGCGAGAATGATGTGCAATATGGTATCGAGCTAA
CTGAAGTTAGCTAGTTCAGCGATCGAAAATTTTTTTCTAGCTTTATCTTCTTTCGATCGCTCA
AAATTTGAAATCTTTTTGTAAATATTTATATTCATGTCGTTGTATATATTTATCAATTTATTCA
TGTTTTTTAATATCATTCTTTAAATGATATGATATTTCATTAATTTCATCAATCGGATTAGCAT
TTGTTTTCTATTTATAATATTATTTTATTACACTTTTAGCCCCTTACATGATCAATCCATGA

TAGTCATTATTATTCTAAAAAAAAGAAGAATTATAAGAAAATCAAAAAGAATTATTCCTTA
GAAGTTACATGTATTTATATTTTTTTAAATTTTATTTTATTTATCATTATTATATTGCA

338. >MSTRG.7028.2 Gene=MSTRG.7028
CAAGAAACTAGTTAATTAAAGTAATAAATAACGTTACATTATTTGGATCTTAAGGGTGCATT
AAAAAGTATGTCAGAAAAAGTAGTCAAGTTTAATTAAATTTTCTTAGCTAATAAGTAATAA
GTACTTTCAAAATATTTTAATATTTGAGATAAAATATGATATTTAATAATCACTTGTGTCTTT
AGTTAAATAAGTGTTTTTACTTAGAAGGCTGTGAGGAAAAATGTTCATTAAAAATATTATAT
ACATTTCTACAAGTTTAATGTATTATATACAATATATTATTTAATTTTAAATTATATATAGTA
TTAATGTGTATGCTCAATATAAACTAATACCATACATTCAGTGTTAATATTCTTGTACAAGT
ATATATAATATACTTGGATAAGTATAATATTCTTTTGCAGTGTATTTTAAGTCACCCTTAAGT
ATTCCAATTACTAATAATAATACAATGTGAAAAGTAATTTAAAATTGTTTTTGAAGAATAAC
AAAAAACAAATTTATTTTTCAAATAATCACAAAAAATTATGAGTTGAGTGAAAAGTTCATT
GTGATGAAAGCAAGAGCTATTTGTGTTCTCAACTGTCCTACTGATTTTGGATAGTTGATACTT
CGCGCGAAGAACGATGGCTAATGCGATATTTCGTCATTTCTTTTTAATCTCGTTGTATATTCT
CAAATGAACGAGATATCGTCACTATCCATTGTGCTTCAATCAAACCAAATACTTAAAAAAC
AAAAAAAAAAAAACGTAAAAAAAAAAACGAAGAAAAATTCACTTTATCAAACTTCGTTGAT
GGTTTTTCTCATTCAATCGTCTGATTAACATAAAATCATTTTTGCCCGTGATTTCCATTATAT
AATTAAGGAACCATCATCGAGGCTGATGTACACCAGAAGCATATCCGGTGACACATATGA
CTAGGTTACGAAATAATTAATCTCTCTTCTGCTCCAATAATAATCAAGCTCCGTATATTTATT
CAGCGTGTATTATCTCGTGAAATGGAAAATCTGTAGACTAATCGTAATTAATTATTCGGGAC
GTTTCTAACTCGTAGCATATATTTTCGAATCAGTCGATTTTTTCCTAGTATATAGTAATTGCT
ATAACTCGTTTGCCATAACTTAATTTCTTTTGATCTACAGTGACAAGGAGCCACATTTCTAA
AGGGATCAGAACGAATAAAAATAGGAGTTCCATTAGCTTCTTAAAGGTCAAATTGTTTTAC
AAAATTCGATAAAGATATATATAATTTTATCTTAAAGTTTCAAATTCAAAATAAAAGAGAG
AGAGAGTTAATCTAACTCATGATTATGATTATACTCAATTTTGTATAAATTTAAAATGAAAA
ACCAAATTAACTTGAGATAAATGTAATTGAAAGAAAATGAAATGATTCAAGTATGGTCTTT
AAGGGGTTAAGAATAATGAATATATACAATTTATCCAGAGGCATTCAAAATGATATCAACT
GTTTCATTTAATCCTTTTCAGATTACTGCTATTATTTATGATCATGTAAAGATGATTGCTGAA
ACAGAAAAATAAATCAGAATAAAGAATGCAAAAAAAAAAAAAAAAGAACTTTATGAATCA
TAACACTTGAAATTTGTACTCTGAGAATCTTAATCTTAGTTTAAGATTTATAGGTTGAATTAT
GAGTTTTGATTTATCACAATAATTCTGGTTCTTATATAGAATCAAATATTCAACAAAATGAA
AAATTATGATCAATACCTCCAGTATCAAATATATTTTATGTAAGTTAGATTTATAAAAATAG
AATGATATAGGTTATATTACTGAAACCCATAAATTTTAAATAATCTAAAACTTTGAATGAA
AATTACAAATTACTACCAACAAATTTTATTTAATTCTTATTTAATTATTGATTCTATAATAAT
GTGTGTAAAGATGTTTCTGAAACAGAAAAATAAATCAGGATAAAGAAGTAGAAAGAAAA
GATTGAGAAAAAAAAGAAAATATAAAATAAATCATGGACACTTGGAACAGCCTATACCTC
TAGAATCTCAAGCTCCTTCAAAATTTGTAGATTATATACTTCACGAATTTTGATTTTTGACCA
GGATTTCTACGAAATTATTTAACATTTTTATTCTCGAATATTCAATATGATTCGTAGAATTCC
TGAAAAGTTATAAGATATCTTCAGCTAACGCCAACTATACTACAAATGAAATGGTATAGGT
TAGGTTATTAATTGAAATTCGTAAATTTTTCAATATTATCCTAAATGAAAATTGGATCATTTC
AACGTTTTCATTACAAAAATTTACGAACAAGACACAAATTATTCGTCAAATGATCTTTCGTG
CAGAACAAATCGTTGCATTTTTTTGAACCCTTCGTGTTTCTTTTTTTCACGATTCCATTATTCG

CACGATTCCAGCTATATTCTAGTACGAGCAGTTTGATCAGAAGAAAATATATCGCGTATCG
GTCTTTTGTTTCTCCACAAAGTATAATAAGAAGAGTTATTAATCGTAGATTTTTCTCGATCCC
GATTCTCTTCGATATAATCCTCCTTATTCGTCTCGCAATTTGCCCTCGACGAACGAAATTTAT
TGAAACAAAGCGAGAACGTTGCACGCGTCAACGTCGATAGAAAACGCGATCGTATCGTTG
TGGCGTAGAGCAATCTGTTAGTGCAGGGGCCTTATCCTAACTGCGCGGGTTCTGATTGGTTG
TTATCGCGGTACCATGTGACCATTACCTTGTATCATGGCGATCACTTGTTTCGAGTCGAAAC
TAACGCAAAACATTTTCACTGATTG

339. >MSTRG.7029.1 Gene=MSTRG.7029
ATTATTCTAAGAATATATCTTCTTCAAGCATATTAGAAATGCTTGATAAGATATTTTAATTGA
GGAGGTAAGATTAATTATTCTTGGATCTTGTTTGTAGATATATTTGTACGGATAGATAGATTT
CCTTTCTTTTCTAATGTTTTATGCGGAAAGAGAGTGATAGATTTTTGTGCAAAAGTGGAGTA
GGATTTGTACGTAAATAGTGGCTTCGACAGGGAAAGACAGGAAAAATCGGCATCGAGCGA
GGGAAGAGATTTGGTTGGATTGCGACTTATAAAATGTTCCTTTGGAAGAATTTTTGTAGATT
TTTCTCTAATGCTCGATTATATTCTTCTTAAATGCTTTGTTGGTAGAGAAAAATTTATAGAAA
TTTTTATTAATCCTGTTACATTTTACATAATATTTTTTTTTTTTAATTCAATTAATTAATAGATT
TTAGAAAAGCATCTGTCTGTTAATAATTATCAGAATTAATTTTAATATCAATAATATTTGTGC
CAAACCAATATAATTATCTTTATATCTAGTCTTCCTTACAATTTATATTATTATATATAATTTT
ATCACAGAATAAAATAAATTATTATTGGATTGCATTAATTGGATCATTGTAATCTCTTTAAA
ATAAAATTAAAAATTAATATTAAAAATATTAATAAAATATTTCTTCCAAGTAAATAATTATA
AAATTACAAAAGTAAAGAGATACAATTTTATGTAAATATCTTACAGGAACCTTCGAAAGAA
CATTTCATCAATTGCAATCGATCAAAAAAAAAATAAAGAATTTTATCGAATCATACGTAAA
TTAAGAAAGGCCCAAAAATGGGTCTTGAAGGACAGGGATTATATAAAAAAAAAAAAAAA
TATACGATGGTACGATCTTCTACCGTTGGGATCTTTAATTGTTTGCATCGATGTTCACGATCC
ATTTCGAAAGATCGTCTCGTCTAATGGAGATAACGGGGTAAACGCGCTTCCGTTACACGAA
CCAAGCAAGTTGATACACCTCCTCCCATTTATGTGATTTTTCACCACTTGTCTTCCTTTTTAGT
TACGCTCGTCTTCCTCGATAGTTGCACATTGAATTCACAACATTGACGAATTTCCACCAGCG
TTGCTCGTTGATCCATGATCCCATAAAAAGGGGAAATGTAATTTGAAATGAACTAAAAAAA
AAAAAAAAAGAAAAAAGAAAAAAAAAAGTCTGACTAATAATTATAAATAATTGTTCTCTA
GAAATTGCTTCATCGTATCTCATTGCATATCAATTTTTTAGGGGAAAAAAAAAAAAAAAAA
AGAAAAAATACGACAGTTTTCGTATATCACTACAAGCAGCTGCACCTCTTTTTTGATTCTCA
CACACGCACTCACACACGATCTTGAATCAAACATTCTTTTTTAGCGGTCAGAGAGAGGGAT
CGCGGATTTACCACCATTCGAATAAAGATTTAATGTTTGTAAGGGCAATAACGATTGTAAA
TTTTTCGAGTACACTGTATTTGAATGTGTAAGTTGCATAAAAAAGACAGATTTTTTAGATATT
GCACTATAATATTTATTATTCTAAGGTAACGGAGACGGTCGCGATTCGCCGCGCTCGCGAT
AGCTCGCCCCGCCCCCCCCCGCCCCCCTCCCCCTCCCCGACCCTCAACCTTCTTATGGCCTC
GATCGCATCGAGAATCGATGCCCGGGATCAATAGCGACGAATTATCGATCGCTTCCGCGCG
AAAATGTTTCCGTGCGAGATATGGTAATTTCTTCAGTCGCGATCGACGCCTAGTCGCAAGC
GCGGCTGAAATTCGCGACGATCATAAATTAATATTAATTAATAAAGAGACAAAGCGAATG
CATTGTTCTTTCCAATAAAAGAAGAAGCAAGATTTTTAGTGTTTACACTACAATTGATAAAC
GATTCTATTATTACATAATAT

340. >MSTRG.7037.1 Gene=MSTRG.7037

TCCGTCTCTGTCGTAGCGACACTGCGGGTACACGATGTGATAGTATTTTTCGAAAAATGTGT
CGATCTTTCGCGCACTCATGCATCCGCTTTTTCACGTGAGCCAGCCGCGATCGCCGTGTGCT
TTATCTTGTGCTTGCCATCGCTGTTGGCGTGTGAATAATGGGCCAGTCGTCGAGTAACACCG
GACACAGCGGTAAGTCTTGCAATTTTGCTCTTTGAAACTAAAACGCCATCTAGTCATTTTTC
TATGAGGATGTAAGGGATG

341. >MSTRG.7097.1 Gene=MSTRG.7097
AAAAAAAAAAAAAAAAAGAACGAAAATCCAAAGAATCGACGATTTATAAAGAGTGCTATA
ATGTTCGAGAAAAAAGAAAAAAAAAAAAAAAATTCCTCCAGAGTCGAAACAAGGTTGAAA
CACTCTGTCTGTCTGTCTGTCTGTCTGTCTGTCTGGCGACGAGAGCGCGAGCACCGATGCCC
AAGAACGCTTCGTTCGGCGAAAGGATTGGAGCGTGTAATAACAATACTTTTTCTAAGTGGC
CAGGGTTTCGACATCCGCAATAAAACATTTACGTAAATGTATATTCGTGAACACGTTACGA
TGTATCTACCTAAAGGCCATGCCTGTTGTCGGTTTCCGTGTCGAGTTGTTGCCCCCTTTTACA
AATTGTACAAAGTGTTTCCTTTTCCATCAAGTGGCGGCGCCGCCGCATCGCGGAAGTCGAA
AACAGGAGGAGAATCGGAACTCGACACAGGTTCGGCCGCGTTCGAAGCTCAATGCGAAAC
TAAAGACACATGTTTTGGGTGTAAATCAACGCGGATACGGTTGGATCGGCCTCGCTTTATG
GCTACATTTTCTCGTAACTTTTCAGATGCGATCATTTCGAATTGCGTTCGTACGATATAAAAT
ATAATATCGTACGTAGTTTCGACGAATTCGTGAAAATTGCATTAGTAAAAAAAGTAGATAA
AGGATCATCGTGATCCAACGATTTTCTCTTCGAGTGAACTTATTGGGTTGGTGGAGTAA

342. >MSTRG.7194.2 Gene=MSTRG.7194
ATAAAATGTACAAACGGCATAACTCGGAATTTCTCGGGCTAACCTCGGAGATGCATTTAAT
AAGTGATATAACTGTACGAATCTTAGAGAAGTTTTATCGAGTAGCTTAGTTGTCGCATTATT
CAGAGATGTCTGTGCCTTGAGAAACGGACTGACGGACAGACAGACGTCTCTGTCAAGTTTT
TGTGATTCCCGACAAAATGTTGCTGTTTGTAAAATTCGGGAACTCTCCGGAATGTATTTTATT
TTTTATAGTGCTAACATTCTATCGGGTGCGTGATTGTTTTTAAGAAAAGAAACGAATATATA
AAAACGTGGTGGTGTTGTCAAAGATCCTGGAAAATCCTAGGGTCCCGAAAACGAAGTTGG
AATTTTCTTTACTTCTAAAAACTTTTGCCAACCACGCCCTAAAAAGGTCGGATTAATGGCAT
TAGAAAACATGGAGTAATTTTAAAATCAAGAAATAAAATCGGAATTCTTAGAAAAAAAAA
AAAAAAAGAAAGAAAGGTATTGTTTTAGTTCTATTGGTTGAGCACAGTTGTTGATCCCTGCC
TTATATGCTTGAAATATGAGGTTAGGCTTGGATCTTGTATTTGTAGAAACGTGTATTGGAGT
AACCACTTGCGAGAAAATTGTAGACATCTGGCGGTGGCGGGCCGAATTACCGCCGGTCGCT
GGATCCACACACGTCCGGGGACGTGACAGTGGAGATGTTTTCGTCGCCGGCGTCAGTGGTA
GGCGAGCCGTCAGGCTACGACTTTCAGAAAGAAGAGAACATCGCCAAGTGGAAGGGAGT
ATTTGTCAACTCCCTACGCAAGGTTCCACCAAGCTTGTTGTGTCCCAAGAGCGTAACAATA
ATATTCGATCCTGGGATTCATCTAAATGTTATGTTTCATATACGTCATACGAAATAAATATTT
TGGAAATTAACCACTGC

343. >MSTRG.7208.1 Gene=MSTRG.7208
AAGAAGTTTCATCGATATGAAAATATCAAGATGATTATTTACATATTATTGTTACGAGCGAA
TTATTTCGATTATGGATTATTTAATTATTTGACATTTATTTGACATTTATCCAATATTAATTTT
CCTCTAAAAAAATTTTCTTCTAAAAATAATATATAATAAAAATTTAATTCGATATTAGATAC
TAAGTTTGATTTAAACGATACGCCGAATGGAATTCTTTGCAGGCCCTACATATAGCTTAGAA

TCCGCAGGAGGCATATCAAAAATAATTTTTTCCAAATATTAAATTAATATCGATTATTAAAT
TTCTAAAAGTAAAATGTTCGCTCGAACAATAGTATGTGAATGATCATAAGTTGACAAAAAA
ATATCAGTAGATACAGAAATAAAGAAAATAGTAACGTTAGCATAGATGCACTTATCCAGT
GAGACAGGAGTTTAGATCCCAAGATTAAAGACTGAATCGAAGGGAAAAAGTAGAAAAAT
AAACAAACGTATTTGTTATGATGTTAATTATAGCAATACATTCTTAATTTTCTATTTCATTTT
ATTTATCGACAATAGTATATATTTATTTATTAAATTAAAAAAAAAAAAATTTGATATAATTAA
TCTGATATATTGATAATTTAATATATGATTATAATTATAAATTTCTTCTTAATTGTATTAATTT
CTATATATTGATACTAGCACTAGTGATAAAGAACACCGGGACTCCTGACTCAGTGTAGTGA
AACATATCCTTTTTAGGTGATCGTAGAACGATGAAATGGGTTAGTCACTTGCGCATATTCGC
GTTGAAGGCACCCAAAGAACCATGTCGAGACATATCTCTGATGGCCGTAAGTTTTAGTCTC
GCCGGCTGCGTTGTTATTATTACTAATTATTATAAGCGGTATTTTTGTACAGTGTCTTACAAT
GATCGAGTCGTTGCAAGCGCAGCCGACGAGCCATTCACGGCATAGCCATATTATACATAAT
ATAAATATATATACACATATTTATTTCAATTATATATAGCGCGTAATACTCTGATAATCTCTC
TCTTTAACGTACACGTAATACATACAGAGATGCGTATACACGTATAATATTAAAAATATAA
ATTAAAAGAGACATTAATAATTTAACGAAACCAAAAAAAAAAAAAAAAGAAAAAAAAAG
AAGAAAAAAAAAATGAACGAACAAGTAAAGCGTATAAAGAATATAAATAAGTCAAAAAAT
CCTCTTTACACCTGAAAGACCGAGCTATGCTCTATTTCCCAAATCTCTTTGTCTCTGTTTGTTT
TTACTGTGGATATTTCGGTGGATATCTTCGCTCTAATATATCTCGGTGCTCTCTCGATACTGT
CCGCTTCGTTACTTATCATACGACTCGTTTGAAGAGACTCATTTTCTCACTTTAGAATTATTG
CATACTGCTGATTATAACACATATACAAACATAGACACGTTTATTACACGAGCATGCAAGC
ATATAATTTTACAAGCATAGGATACACGTATAATAGATTCTATGGATATTATTACTATATAT
ATATATGTATATGTTATTTCTTTTTTGATTAAGATATAAGTAATTAATCTGTAATAAGGAACA
CATAAAGATTGTCTGAAATAGGAACAAGTATTGGTACACATGCGTGCATTTATCACAAAAA
AAAAATACATACATGTACACACGCACACGGGAAATCCATGCCTAATAAAATGACATATCG
GTGTTATAACGTATAAATATAAATATGAATATAAATATATAAATATAAATATTATATAAAA
CATAGATACACATGAAATTCGTCTGTATATGTCTATAAATATTGTTACATATATATATGTAT
ATATATATAATATATATATATATATATATATATATATATATTTATATATATGGTCATTATAATGA
TAATGATTACGTGATATGTATTATGAAACATCTTTTTCTCGCAACCAGTCCGCGGTACGGGC
ATCTACTCAGATTCTAATGTCTATATGTCATTGTTTATTATGTCGTTAATAATTAACGTGTAT
ATGTGAAACTTTCAAAACGAATAAAAAGTACCTAAGCATA

344. >MSTRG.7264.1 Gene=MSTRG.7264
TTTCTATTTTTTATTCTATTATTTAAAAAAAATTGAATATTATTTAATAAAATATTGCTTTTCCC
GCGCGATTTAGAGTAGAAGAAAAGTTATGCGACATCAGCCAATCAGAAAACTGCTTGATG
TGCTATTACGCGGGAAGTCATTCTACTGTCGAAAATCATGGCGATAGAATGTGTCATCCCG
CTGAGGTGTATGACTTCGATTATCAACGTCGTATTTTCATTCCTAACTTTTCCAGTTTATAGA
TTATTATATTGTATAATTGATCGTGAAGATGAGAAAAAGAAAAACTGAGAAGGGACAAAG
GCATTTTGAACGATATTCAACGAATTCGATAATTTTTCATGCAGAGCTGCCAACTTCTAATA
AAGACGTATGTACACGTTGGCATTATTTTTTAATTAGATTAGTAAGAAATATTATATATAAT
TGACATACATACATATATTGTACGAATGATTCAATTTTAGAAATAACTTTTATATTTCGTAA
ATATTTTGTAAGATTGTAATATCGTCATTCGAGTTTCATTGTTGATTGTTATTTTCATGAAATT
GTAAACACAGGTGCGTTTAGTTTTCGAAGAAAAGTTGATGTATGTATGTATTGATGTATTCA
TGATAGAATATATCTAGAAAAAGAAACGAATATTAGAAGAAAAAGAAAAAAAAATACGAA

ACAAATGTTTTACTTTTCTTTTAATTTTTCTTCTTCTTTTACTAATACAATTTTATTTCCACAGC
ATTGTTGTGATCATGTGCAATGCGACCATTAAAATCAGTGTTTGTTGCGTAATAATATTTTTA
ATATTTTTTGAAATATAACTTTTGTATTAATTATACGTTCAATTATAATAGTAAAAATTAAAA
GCACGCGAAATTAGAGTCAGTGATTGTTCGTTAAACATAATTCGAACGCGTTAATAGTCTTT
TATTTATTCATTTCTTTTTTCTTTTAAAATATTCGTTTTCGGTAATAGAGTCAGTGTTTTCAAG
AGAATTCAGAAATCATGCCTTCGGAAATCAAAAATTTTTCTATAACATTTGGTGGTTACTAG
TAACGTTGATGATAGGAGCATCTATAATAATATTCTCCGCATCTAATGATTATCGATCGTGA
TCGACGAATGAGAAACGAGGCGTAAAAAGAAAGAAAGAAAGAAAGGAAGAGGAAGACG
AGAATTGGACGATCGACGCATTCACCCCGGATCGGATAAGTTCTCGTAACGCGAACAGTGA
CGGAATGGGTGTTACTTTTGCACCGACGTATATCACCGACGCGACGTTAATTGCGAGGCCC
CCGATTGTCTCGTTTAATGAGCGCATCCTTTCGCGCTCAGAAATGTGCACCAATTACGATTG
TCTTCGGCAAACTTCGCGGTTAAACTCGATTCAGAAAGATTTATCTTTAATTAGTTCGATTC
AAAGCGGAAACGGTGAAATCTAATAGAAGATAGAAAGCTCTTTGCCTTTAGATTTAAACTA
CGTTACAAATATCGCATCTCATCGAGGATATCATTCTCCAATTATCTTCTCTTCTTATCGTGG
TTTATCAACTCGTATATTCTCTTCGTTTCTTTCCAATCAAACATCGTATCTCGTCATTCGACTT
TCGTCATTAACAACACGATTTAATAAAGATTATTATTTCAAAAGCGATCACGATGAAAAAT
ATTTGTATAGGCGAAAATTAAGCTGCTAAATTTCTCGATCCCGAAATAACACCGTTCCAAG
GATCCTCACAAATTTTCCTTTATACTTTAGAAGCGATCCCCAAATTTCACGGATACTTTACG
CTTTCGAAGAGGGATTCTAACCTCTCCCTCTCTCGTGGGCGTCACGCTGTCTCGGTAATTTA
GCCTTTAATAGCGGATTAGCGCGCGATATATATATATATATATATATATTTATTCGAACTTG
GCGTGCCTCGAATGATTTACGCTCGCCTCGAACGATTCGCGCCAGGAGTTATACTGGATAC
GTATAGATGTGTTTGTCACGTCTTCCGTGGCTTTCGTTCCACGGAACGTAATAATCATCATCC
CGGAAACGGATACCCGTTCGAATTCTCGATCATCGTCAACTCCTAGGATCGTGAAACGATC
TTTCTCTCTCTCTCTCTCTCTCTTGCTCTTTCGAAAACTCTTAACTGCAAATTTCACCTGGAAC
TCGAACTAGGTACCTCCACGATACCGGTACTGTCATTTACCATAACTTATTCTCACGCGCGC
GTGCAAAAAGTGGAGGAAAGGGCGAGGAGGAGAGAAGAAGTGTTGGCCTCGAGGGAGCG
AGGAAGGAAGCATTGGGCAAATTACGGCAGCGCGAAAGGGGAAACGAAATGAAAACGGA
CTCCCAGGGGCGAAACGAGCTCTCCGCAGCCCATCACCAACCTCGCCGATGATTTTCACGC
CGATGATCACATTTCTGCCTCGGCGAATCTCGCGTGTTTTTCCGCGAATCGTCTCGTTCCTTT
CTTCTCTCGCGACTGATCTATCGATTTCCATTCCGTTTCGGTGGCAGCGTTTAGTTTCGAAAG
AGAGAAAGAAAAAAAGAAGAAGAAAATAATCATTTTTCAGCGAGGTTGCACGAAAATCG
CGATGGTAATACGAAAAGCTCGTGAGAAACGGTCGTACGTTAAACGATAAATCCGTGCGA
TAGAGGGAGATAGAGGGGGGGATACCGTTGGAGAGTTTTCGCCGGCCGGAACACGTGTTC
GCGCGAGCGCAATAACGCCGATCACGTATACGTGGCGCATATTAATCGCCTTGGTACGTGC
GAGTTGCTCATCGGCCACGCTCGCGATGCTGTATACGTAATAGAGAAGAGAAGAGGAGCTT
ATTACGAGTTAACGCCGTGGCGGCGTTACGTAAGAGAGCGAGGCGTACGATCGTTGAGATT
TCGTCGCACGAAACAGGGTGTAAAAATTCTTAATGGAACAGGTAAAATTTAGATTTTTTGTT
TTCTTTCTCTTTCAAAAAACGTT

345. >MSTRG.7265.4 Gene=MSTRG.7265
CGAGGATCGAGAACCAACCGACAACTCAGGAAGGCAGTGCACGCCCGACTGCCGACACG
AGCGGATGGCGTACGTTGTCGTTCGCGCCACGATTACCTCGTGATTTTTCTTTCTGTCAGATA
CGGTCACCGTCATCGATCTTGTGACGAGTCGCACAGTATTTTCAGTCATCTATTGCGACTTC

ACACAGTCGGAGACTTCAGGTTTGTTAGTGACATTGCGTGCCGCGTTCGATCGCGAGATAG
AAAGAACAAGAAAAATAATAAAAGGAAATAAAGAAACAGAGAAGACGAAAGATATTTGT
TTTCCTTCGATATTCGTTTCTATCGGATTTCATCATCGTTTTGTTTTTGTCATCTACGGACATA
TCGCATACCGCGCACGTAACCTTTTAACCACGTGTGTGCGCACGCACGCACGATTTCAACG
ACGCACTTACACGTTATCAGGTCTGGGCGCTGGCGTATGGGGCGCCCTCCAAAAGTCGCGC
ATGCAACCCTACCGGAGCCCGCTGACGTTGGTTACCATAGTAGCGCGCGTGCCGATCAGCT
GATCGTTGGGATCCGAGCGTGTTCGACCGTCGGCAGTCGGCCAGCAGCGGCTCCGTCCATT
CGTTTTATGCGTTATCTTCCCAAACGTTCGCGCAACTTGATCGCCTTTCGCGTCGTGATTTAG
TGATGAAAATAACTAACTATTTCGCGAACATCTGACAACGCAGATTCCGAAGAAAAATTA
AGAAATGAAACGAATTGAATAGAAAATTTATTTTACTCACCGCTCGTATTATCCACCTATTT
GTTTTTATCGATGTTCGAAGGATACATCGTTACAGTTTATTACTCGATCGATCGATACATGTT
CAGATGATGGAGTCAAACACGATTTCGGATAAATAAGTGTCGAACAAAGTGTATTATTTCA
ATAATCCATGGTACGTGTATATTTGTGATAAAAAAAAAAAAAAAAAATCAATAGCATTTTTG
TTAATCATCGTGCACAGATTGAGATCGAGACACATTATAATATATGATTATTAAGATTTTGT
GATTTACATCACGTATTTTAAATACAACCGTCCAAATATGAAATTTAATGGAAATATATGG
ATAGTGGATTTAAATATTTACTATCATGCTCTTTTGTTGTAAAGTATCTCGAATGAAATATCG
AACCATTTTTGTTGTCAAATACACTTACGAGATAAATTATATTGAACGCAAAAAGCAATATT
ATTAATGATAAATAAAAGAGATGGTGGATAAATAATATAATAATATATACAATTTTTCGAA
ACAGCATTTTAATAATAACCATCGGATGATTTTACGAATATGATTGACATTTGGTGATCATT
TATGACATGGTACAAAATAAACGAAGCAAACTTACGAAAAAACGATCACTCGTTGAAATA
AATATATTACAAAATTATAAAGAATATTTTATATTCATTGTTTATTGTATCAATTAATTTGCA
AACGAAACAAAATTTGATTGCTCAAATTAAATCGATTATATCTTGGAATACACTTGCACTGT
GAAGATGGAACATAATCGTTCCAAATCGATTGATCAAAAGATGATGTCATAGCGTGTCTTC
GAAAAAATATCGATATATCGATATTGTTTCGTGTCAGATGTCGCTAGACTATCGCGATGGCC
GCGGAATCTTCGTCAGTACGAAATCGCGACAAAAGAACGAATTGTGAATTAAATAATAGT
GATCGTAGTGATTGATAGTTTTTGTTGGAATAAAATTCGGACTTGTTTGGTAACGGGTTATC
GCAGGATGTGAATGTTTCTTTGTTACGAACATATTTTGACAATCGTTTGTACCTGTGACGAAT
CAATCGTGTGTACCTTGACTGCATTAATCAGACTTTTTGTGAATATGTGAAGTGACTGAGCT
TTTTTTTCCGTCGAACATTCGTCAAAAGTGATTTTTATTTTGTTTGGCGTGATCGCAATTACAT
TATTATGTACCGATCGCAACAATGACAGTTCGAGATATTTTCTCGTTCATACGGAAATTCTG
TGATAGCCAATGCCAGCAACGCCATTACTTGGATTAAGTGTGAGGAGAACGCCGTTCCTGA
AATCATCTTGATAGAAAGCTTGGTGACGTACGCCGAACGGGCCAGTATTCATGCCAGTATG
CCAATCAGATGCCAGTAGAGAGCCAGTATCTGTATCTCGTCGCCACCGATAGCCAATTAAT
AAGTTACTTAGATAGTTAAATTAATAGGCAATACAAAAGGGAAAAAAAAAAAAAAAAAGA
AAAAGGAAAAAAAAAACGTCAACGTTTTTTTTGAATAATTGTCGTTCAGAAGGCGTCGTTCA
ATAAGCGTGACGGATATATATATATATATATATATATATATATATATATATATTACGTGTATTC
AATCATACACGTAGTCTACGTATATCGATATATATAAATATAATTAAACGTAAAAAAAATA
TATATATATACAATATATAATATATATATATAATACAATCGAGAGAAGCGATAAGTGTCGG
GAATCGTGCGAGCAATTATTTTTCGCATCGTACTTTTTTCTCTCTCTCTCCTCCCCTCCCATCC
TCTCCCCTCTCTTCTTCGACCATATTTTTCTCCGCTCGGTTGATCGGTTTTCTTTCTTTCTTTCTT
CTTTTTTTTTTTTTTTTGAATTTATATCGGTGACCGCGTATATGATTCCCCGCGGAGAGTATATC
GACAAGATTGGACAGAGAGTGGAATCGAGTTGTGATTATCGAAGTGGACCGACCGATCGA
TTTCTCGGCTGTGAAGTGAATTAATATCCCAGGAAAAAGGATTGAAAACGGAGAAAGGAA

GTGAGTACAAGCAGCGAGTACACGGGCGAGCAACGGGTGGCTGGAAACGGTGACTTACAT
CGAGAGCGCCAACCTGCAATCAAGGTTCATGGTTTCATGGATGAGAGCGAGCTCTCGAGC
AAGCGGTGGGCCAGCGCGCCCCGCCGCCCGGACAGCCCCTGCGTCCTGGGGGCACCAACG
TCTAGGGAGGTGCGAGGTTTCGCGGCGGGGTCAAGTATCGGGGATTTTTCCACGGAAAGCT
CGGAGATTCGCAGATAGCGGCGCTCACCTTTGCGCTCCAAGGAATTTCTGTCAACGGGAAG
AAAATAACGG

346. >MSTRG.7273.1 Gene=MSTRG.7273
AGAAAATTGTAGATTGTAGATACCTGAGAAAGTTGCAAGAGTCACGATAGACATATGGAC
ATACTGTAGTCACAAAGTAGCTTTCAACATTGAAAAGTCAAGTGAAGAAATTATTTATTAG
AATTTTAGAAAAATGGCACAAATGGTGGATAATTTCGCTCAATCTCACATAGGTTCAGTTA
CTGATAATTTAGTTCAAGATTGGGTTTCTTCTGAACAAAGTACAGGAACATCAATTATGGCT
TGTGAATTTGATGGAGGAGTAGTAATTGGAGCAGATTCTAGAGCTACCACTGGGGCTTATA
TCTCAAATCGTTTTGCTGATAAATTAACTAAAATCACAGATTATATTTATTGTTGCCGTTCTG
GCTCTGCAGCAGACACTCAAGCTATTTCTGATATTGTTGCTTATCATTTAAGCCTTCACAAA
ATGGAATTAGGCATAGAACCTTTGGTAGAAACAGCAGCAAATGTATTCCGTGAATTATGTT
ACAATTATAGAGATTCCTTAATGGCAGGAATTTTAGTGGCAGGATGGGATAGTCGTAAAGG
AGGTCAAGTTTATAGTGTACCTATAGGTGGCATGTGTGTAAGGCAACCAATTTCCATTGGA
GGATCTGGTTCAACCTATGTATATGGTTATATGGATTCGCAATATAAACCTAATATGTCAAA
AAATGAATGTGTTAAACTTGTTGAAAATACATTGGCATTGGCAATGTCTCGCGATGGTAGC
AGCGGTGGTGTTATTCGAATTGGCGTTATTACGAATAAAGGAATTGAAAGAAAAGTTATAT
TGGGTAATGAATTACCACGATT

347. >MSTRG.7330.3 Gene=MSTRG.7330
GGTCTTTCTCGCGGATGTTGATTACTGTTTTGGTGAATGTCGCTAAAACTTACCCGGTAAACT
TATAAAAAACATATTGCGTTTATGAAGCATAAAATATAAAAGTGCATTACAATTATTTACA
ACAAAAGTTAGAGTATTTCGATATTAGTGTCATTATAAAATAAGTGCGGATATCGCGATAA
AATATTATTGTTTTGTGAATCTACTAGCATAAGAGAGATAAGGAGAGTGTAGAGTGTATGC
GAGTGCATTTAACATACTCACACAATTGGTATATTCGGCATGTTCGTGTACAATGGCAACCT
TGACACTTGGTGATCGTTTTTGGCGGCTTCGTCCCGCTTCTACTGAAATCGTGTTTGGTAGGA
TCGCGTCGCGAGTGAAAGCAGCAGTGGCCATTCTGTAAAATCTTTAAGAGTTTGGCAGTAA
AGAATCATTAAAACATTTCGCGCTTCAGATTATAATATCTCATTATTCTGTTTATTTTCGTGA
TTGTAGTTTCGTTCGTGAAAATCTGTATATATGTAAACATTAGTGGC

348. >MSTRG.7398.3 Gene=MSTRG.7398
GGCCGCAGAGCGGCGCACCGTGTGTCGAGCCGAGCCGAGCCGAGCCGAGTCGGTCAATAC
TCCGGGCAACGCGGTGGTGAGAGCACACGACCACGGTCGCCCCGTACTCGTGCACGCCGT
CGCTCGGAAGCGGCGACTACGCACTCTCTCTCTCTCTCCTCTCTCCTCGCGGATTCGTGTCGT
CTCTCGGCCGATAATCGAACGCGTAATCTTGGTGGCGAGCGTGGATCCGGAAGGGACGCC
AGCCGGAGGACCGGGCGCCGATCTATTCTCGCCGTGATCTACCTCCGCCGTGAACGGACGC
TCGCGATACGTACACACAAGCGCACACACAAGCGTAACACAGGCACGCAGAGAGGAGGA
CACGCGTGGCAGGCAGCCGAACGCATCCCATCGCATCTCGTCCCATCCTTGTTTCCCAACGT
ATATACGCGGTGCCCGGTTGGCAGCGTTCTTCTCTCAGCCTCGAAACGGCCGCGTTCCTCGG

CGAGAAACGCAGCAACGATGCCACGAGCCTGCTTCGTGTTCCAGTACGGTTCGTAAGGAA
GGAAGGAAGGAAGGACACGAGTGTGGGATCAGGACGCTGGTGACACGGGTCCCGGGTGTT
CCATCAACATTCCCGCTTGTGCTCGCTCGTGTTCAAATTATCGCGGACAGTGATATTGTGTG
CGACGAATTCCGAAATAGTCGAGGATCGAGGATCGAGACCCGGAATCTCAACATATACGC
TCAACTATGAATCCTGGAGCGTGATGGACAAGCTCGAGTACGCGACCACGACCAACTTGC
GGCTACGCCTCGCCGGCATGCTCATGGATATGTGGATATTCTTCCTGTTCGTCGCCATTGCC
GGTGAGTGTAAAACAATCCTCTTTTCTTATCTCTCGTTTCGCTCGCGTTACGTTACGTTTCGA
TTCGATTCGACGCAATTTTCAGATTCTCGACCCCCTTGTGCGTGCGTGTATGCGTGCGTGCGT
GTGTTCCCGATCGTGTGTATCGATTCGACGAGTGGCTCGCAGCGTGACTTGGTCCGCGTGCT
GTCACTCGACGTGTCAATACGCATTACGTATGCACAACAGGCACGTGCGGCACGCTGAGTA
AGTGCGTCGTATCTGTGCCATGGTTGAGACTCGATTCATTCAAGAGATCGGAGAGAGGGAG
AATCGATGCCACTTTCGTTCTTAAACCGGTGAGCCACGGATATTCCGTTTTTGGACCGATTC
AAGGCTCCGATTCGAGCCGAGCGGGGGGGCGGAGGGAGAGGGATCGAGGCAGAGAAGCA
TGCCGCGTTAAAAAAAATACGAGAAAAATATATTTATGCACGTTGGGGACAAGAAGAAC
CGGTATTACGCGTCTTTGTACAATTTAACGTACACAATTTCTCCTTTGTGTTTATCTCTGTTTA
TATACGCGATTATATACACGCCTGAAGGTTTTTGTTTTTGTTACTATCTGCGACCTCGTGGGG
AGCCGTAAGCGCTTCGCCCGTACGACGCAACATTGTCGAACAGTCGATAGAACGAGCCAG
ATGGTTGAAATTCTTACCGATGGGCGAGGGGATTCGGGGAACTCAGCCTCTTTATCTTGGTC
GTTTTCTCTTTATCCTTTTCTTTTCTTCTTATTCCCTCGATCCATCAGTCTCCTCGCTGCGTACT
ACCTTTCGTTTCGACACGTTCAGGGGAATCCTCTTTTTCCACGTCTCTCTCCCTCTCTCTCTCT
CTCTCTCTCTCTTCTCTCCTATCTCGATTAGTTCGTCCTTCTCCCTCGTCGACTCTACTTCGAC
TCTTTCTTTCTCGCAGCCGGTTTTACTCCGTAATCCGCCCTTCTCGTCCCCTTCTCGTCTCGAC
TCCTTCCCAGAGTCCGTATCTCTACGCTTCTCCCGGTTCCACCCCGATATACGTATGTATGTA
TGTATGTATGTACCCTCTTCTATTTCCACGGCAGGGCACCTCGATGCAACCGTGCGTGAGGA
ACGGCGGAAACCGGGGGGATAACTATCGGCGACTGTGCTCGTCGAATCTCATTTACGATCG
TGACGATCCGAGGAGAGGATCGTAAAACCGCTCCTCACGTACTCGATACCCACTTTACCTT
GGAGCGTCGCGCCGGTTCGTGTAATTGCTTCGTGCCTCGACCATCGAAATTCTATCGCGATC
CTCGATTTCGAAAACGTCTCCTCCATCGAATCGTCTCCTCGTCTCGCTTTCTCTTCTCTCTCTC
TCTCTCTCGCTCGTTCGCTCTGTATCCGTAAATGGAAGGGACCCAACTTTTCTGTGTTTGCGC
CGAAGCCAGGCGTCGGCTAAAAGCTCTCTCTTTGATACCACCTGCGTCTGATTTAGAGAATT
TCACGAGGGGCAAAAAAAAAAAAGAAAAAATCTGTCTGGGATGAAAACTCGGTTCGGGC
AACAGCTTCGGATGGAGATTTGCTTTATACACGGGTAGAGGGGAGGGTATATGTTTCGCGA
TAGTTGGGGCAGAAAAAAATACGGGAATACGATATTTTGGACGTATGCCGCGGATCTTTTC
AATGCGCGAAAGGTACACATCTCGTATCATCATCTCGTCCGTCGTTCCTGTTTTCCACGAAC
GAATTAAAGGGAAAATATGCCGCGTCGAGTGAAAATTAAGCTTTTCAGAAATTGCATCGTT
CGGCTCGCGTTCGTTGCATCGAGGTACAAACAGGAGTCCTTTTTTTTAGGGGAATCGATCGA
GGCTCGCGAAATCATCGAGCAGCGATCTCGAATACGCGTTTTCTCCCCCCTCCTCTTCATTG
GGACGATGTCGAACTCGTTAAAGTGTCGTTCAAGCTCATTTTCAACGCTTGAAAACAAGCT
CGACGCGAAGCGTTAAGCGAAATGGTTAATTTCGCTTGCTCTTTCCCTCCCCGATAGTTAGG
CGAGAGAGGAAATATCGCGATTCTTTTCAATGGTTTCTCTCGTTACATGAATCACGCTCCTC
ACAGATAAGCTTCCGTTTCGTCTTGTTTCATAGATATTAGTTTTCATTCATCGAAACGTTCTC
GTAACGAATCAATTGCATCGGACTCGATGCCGTATTTCTGCCTCTTGCTCGAGTCAGTCTCA
AGATCGTATCGTGCATTATTCATCGTTCAACTAGTATTTAATTGCATTTATCCGCGTTTATT

ATTTCTTACCCCTGTACACGCGCGTCCACACAGGTATTCATCTTCCTTCCGTCGTTCATATCC
GTTTCATTCATTTCGATTCGAAAACTTGGCGACAATATGCCATATTTGTTCCGTTTTATCTTC
GAAGATTAATCCCATTCTAATCGAATCATTCGAATTGCTTAATCCTTGTTTCGAATCTTTTTC
TCCTCGTCTTTTTCTTCTCTCGCTTATTTTTGAAAAAATGCGTTATATCTTGGAACATCTCCGC
TTGTTTCAGGCCAATTCTTTCCGTTTCATTATACGTACGAGCAATACTTCATACTTGACTCGC
TCACGACCCAGGAATTCTCATTACCTTTCCGTGGCACGAGCACATTCATCAGGTGTCAAATA
AAATTCTCGCGAGCCTTCCTTACTTAAACTTTAACAAAGATCTACTCGTAGGTACAGGTGGT
GCGGGCGACGCTATCCCCGGTTCAGATGAATTAAGGAACGTCCTTCTCGTCGTCGGGGCAC
GGCGATAAACCGTTATCTTTGTTTCAACCCGTCCACCACCTCGCCCAATCGTCTTCAACTTTC
ACTTGTGAGACTGAAGTTCGCGACGCTTGCCATCCCTTCGATTCGCTCTCCCGATAGTTTGC
CTCCAACTTTGAATAAATAATTCGATACTCCTCCTCTCTTTTTTGCAGAATTCCGCGCGCGTC
CAATCCTTTCTTTCAAATTTCTCCGTTTCGAGGATGAGAGAATCAGCGTGAAAAAAAGGGG
AGAGCTCGATCGACCGTTGCCTGGTCAGGGTCCCTCTTAAAAATTTCGCGATACGCGATAG
GTCGTCCCAGCGAGCGAAGTCGAATTTACGGGGTCTCTGGTGGCAGCTACATCGACCGGTT
GTAACGAACTAGAATGAACGAAGGGGGGAAAAAAAAGAGGTGGGTCGGTAGGTAGATAG
GTATGGATTGGTATAGGCAAGGCGAGCCAAGGGCTCCTGGCAAACTGAGCGGAAACGGGA
GACGACGACCATCGTGACGACCTCTCATCTGGAACTTCGTATCTTCGTTTACAACGAGAAG
CAGGTGTTCAAGAAGCGCGTTATCCTCGTGCGTTTCTTTTAATTTCCTTCCTTTTTTCCTTTCA
TACCGCAACTTTTTTTAACAGCTGTTTCGAAACTCGACCCGAGATTTACCGATTTTTCCTCCA
CTTTTCCTTTTATCCACAGGTTTTTATAAAGAAGGGATAAACGCTCGTTTCGTTATTCGAAGG
AAGGAAGAGAAGGAATGTTGCGTGCATTGGAGCGAGCCGTTGCCCCACCACGCTTGGAAA
CCCTTTTGAATCGGCGGCGGTTTGCTCGGTGAAATCGAGGATATCTGGGCGGACGTGGGAG
GAAAGAGTCGAAAGTCAAATATAATAGCTAAAGGCTCTCTCTGCCTTTTCCGCTTTATTTGA
AATCGATCTCTCGGCCACTCGAGATATCCGTATCCGATTCGACGAGCGCGAATCCTCCGTTA
GTAGTCGAACGCTTTTTCCTCCTCGTGCTTGTCGTATAAACGTTTCGTGGATTTCACACGGTC
ATCGCGAGCAAGCTATTCACAAAGCTCCCTCCTCCCTCCCTTATCGACCATCTAAAACACGT
TTGATCTCGTATCCGCGCCTCCCCTAATCGAATTAACTCCTGTATCGTGCCCTGCCTCCTGTT
TACTTTATCGATTCCCATTTTCTCGTATCGCGTGCGAGGACACAGAGACTCTTTCCTCCGGA
ACGGAAGCACGCGATCGTGATTTTCGTGAAACTTGATGGCAGCCGCGCAAGCGATGATTAA
TGGAGGAGGAGGAGAAGGAGGAAGGGCAAAGCAGTGCCGATGTTTCGAATAGAAACGTG
GTCCCGCTCGGAATTCAGAAAGACGAAAGAATGCATTTCCCCGTGTCTGCGATGCGACGGC
TGTGAAATTGCACCGATGCGCTACGCTTTCGTCCGCATAGAAGAGCACGTACTTCCCCCGC
GAAATTCTTGTGCGGAGGTATCACGCCTGGCTTCCTGAAGACCCTTTTGAACGTGTTCAATT
TCGCGGAGAATGTTTGCTTGAAGGCCATGCTGGAGAAGTGGCGGGGCCTCGCTCTGCCCGA
CAAGACCCTTTTCCAGCTCATGCTGGTGGGCAAATTGTTGGACAAGAAAGAGTGCGACTTT
TACAAATTTCTCGCCATCGCTTGCGGATTCCTCGGTAAGGTAAGACGAAGAAGAAGAAGG
AAAGACACGTATCTCTACTCTATTACTCGAAATACTCGAAATAGGACATCTCCCTCTCCGCC
TAAGCTCTTTCATCCCCTTCGTTTGCCATCTTTATCATCGCGTCTTAAGTAGGATACTTTTAG
AAACGGTCGTTAATCGATCTGATATTCCTCTTCCCCCCTCGGCTGGGATCCCGTGATTGATG
AAGCGTTTTTATCGCGCGGGTGTATCGCCTGGCACGGATAGTGTTATTTTTTTCTCTCCTTCTT
TTTGCACCGCGAACGATCAGCGGGGACAGTTCTTCTCGCGCCGAGAAGATCGTTTTATTATT
ACCGCTTAATACATCATTTCCTCCCAGCGAGGAGGAAAAAATTCGAGAACGGATGCTCGCT

CTTTGTTCCTCCTGGATTCGCGGAATTTACAACGGCAAGCTTTTTTACAGGGAAAAGAGAA
ATGGGGGGGAGGAAAAGACATTCTAACCGGTAATTAAATTTTTTCC

349. >MSTRG.7430.1 Gene=MSTRG.7430
TATCGCATCTTATGGTTCGCTCAGTGTTTTATATTTCGTTTATGGTAAGGGACGTATATCGTG
TTAAATATGACTCAGTTAATAAGAAATTCTCTTCTTTTATTATTTACGGAAGAGTGTTTAGAT
GAATATTTTGACGACTTGAATTTTTTTCACGTTGGATGTTTTAAATCGACGCTGAGCAAAGTT
TTAGGTCTTGGCATTATCAGTGGATCTCTTCTTGTGTATTATTGGATTTATTTGCCATCACTGC
AATGGTATCTTACAGTTTTATAAGTGGTTTTCCATTTAGTTCATGGGGCGATGGAGTTTTTTT
AGGGTTACAAACATTAGCAATTGCTGTTTTAGTTATACATTTCAATGGTAATACAACACACG
CAACGGCATTTCTTATCTGTTATTTAGCAATATTACTTGCTATTATCAGTGAATTGACTCCAA
TTAATTTTCTTTGGACTTGTCAAGCGTTAAATATACCTATTGTATTAATTAGTAAGTTAATCC
AAGCTTATACAAATTATACTAATGGAAATACAGGTCAATTATCAGCAGCGACAGGTTTCTT
GTTATTCTTTGGATCTCTTGCTAGAATATTTACTTCTATTGAAGAAACAGGGGATATGACGA
TGATAATAATGTATATATGTTCCACTACTGCAAATGCTATAATAGTTGCTCA

350. >MSTRG.7454.1 Gene=MSTRG.7454
TGAAAGGTAGCCAAGCTTCGTACGCCAATCTCTGGTTTCTGAAGTCGGTTACCAATGAAGA
TACGATCATGCAAAATACCGTAAACTCCACCATGACCGTATAATAAATAGTGATCGACCTG
TCAACCGTTTGATGAAAGCTCGTCATTCAATCATCTATTTCGTTGCTGATCGAGATGTGTTCA
GTGCGTTCGGTGCGTTCGATGCCGGAGAGTCCGCTGGAATTACGGCATTAACTCGAATGAT
AACTGAGAGTCACCGTAAGAGCGCAAAGGCTTAAAAAATAAATATATAAAAGAAAAATC
ATCTTCAAAATGGAAGAAGTATATCAAGGCAAGCAGAAGGAAATATTATGCGAATAATTTT
TTAAAGCTATCGAAAATTTTTATAAAAAAAAATGTAAATAAATGTAAATGTAAATAATATA
ATGTAATAATATGTAAATAATATATAAAAATGTAAAAAATATAAAATAAAGACACCAAT

351. >MSTRG.7474.1 Gene=MSTRG.7474
CCCCGTTCTATCTAAATTTAACCAAAATTGCAAAATATTACTATTAAAATCTGAAATTGCAA
CCGAACAGAGATCGAATTTTCGACGAGATCGTGGATCGTCGTGCGGTTTCTCAGTGTTGCTG
GACGAGAGTAGGTCCACTACCAGGATTTCTCCAGGGATCCGGGATGTCGAAATGCGGTGG
GACTGTTATCAGAGCATCATCCGCGCTTCTATCAGCGCTACAATTATTCGGCTTGACACGGG
TAGTGTTCACACACTCGATCGGTAGCTTGAAAAATTCGAGCGTGTAATTATAACCTTGGCTG
CTAACTCGGATGATAGTATGCAATGGTACTTCCATGTAAGGCAGCTCCTCTGGTTTCTTATC
CATGAAGAAACCGATAATGCAACGCAGAATAGCTTGATGGGATACCACTAACACGTTGTC
AGATCTTTGCAATTCAGCAATTACCGGTTCTACGCGTTGCATCGCATCTATATAACTTTCTCC
CCATGGATAACGATAGCGCAGCTTGTCCTGATCGCGCCATGCAAATTCCTGCGGATAATGC
TCTTGCATCTCCTCATAAGAAAGACCTTCACAGATACCAGCGTGGAGCTCGTTAAGTGCCG
CTACGTGTTCTTGAGGTGCCTCAACCCCACGTGCAGTCGCGATAGTCCTTCGAAGACGACTG
GTCAATACGCGCAGATCAGGAATTCGCATAGCATTGAATTTTGTAGCCAATGCTTGTGCGTA
TCTTTCGCCACGCGTGCTTAATACCGCATCGCCACCGACCTTGCCCAATACATTGAACTCGC
TTTCACCGTGCCGAGAGAAATAAAGAGTATGCGGTTTGACCGTGACACTTCCAAGATATCC
AAGTACGCTTGACTCCACATGGCCTGACACCTTGCAAGTCTCGATGTCCATGCTGCCAGTGT
CGATACGAATTCGAGGATACGTCTTCTCGTCCATCGGCTCGTAAGATTTAATATAATGAGCT

ACCTTGAGCCGTAAATCCTCCTCAGCTCGTACCGGATCCATGCCAGCATAGTCGGCGCTGT
ATCGTAATATGTCTTTATAATTTCTTTCCAGAACGACTGGATCGTCGCAGACACACTCGATA
AAAAGGACACGATAACCAAGTTGTCCAGAAAAATAGTCGAAGACCTCGGCGCGCTGCGCT
CGTGTTACCAATGTGGCGTCCAGGATAGCTACGGAATTCCCGGCTGTGAGCCATGCGGCAC
AGTCATGCATCGCGTCTCGTTGGGCCAATGCTCTTAATGCTGTATTCGCTGTGTGATCAGGG
CGAAATAGCTCGTGCGACACCGCTTCACCGTATGGTTCCAATCTTTTTCTCCGGTAGTCGCT
GACGCTCATTATTTTGGTGGAATCGCCATTCCAGTTGAGCCTACGCGAAAGACATTGAGCC
ACTTGGCTTTTTCCACGACCTGGTAAACCACACATCGCTATCACAACTCCTGCGAATTTACG
CGGGGATTTCATTATTCCCTGCGTCCTTATACTGATTTTATCCTTATTAGGTTCTTGGCGTTGT
TCCTCGGCACTCGGAGCCATGATCTTAGTGCTCAACGAGTTTTCCTTCGATCTATTCGACGT
ATTGGATGGATTAGCTGCCTGTGGTACAATTGATTTATTGTTCTCTTCCTGAAAAATTCCGGC
TTGCAGTGCTAATTTTCTTATGGCCAGACAATACCCAAGAATCGGTGCCAACAGATCAAAA
CAAGTAACAAGCCAGGGCATAACACGATAAAAAATTTAATTAGATTTATTTCTTCTCCCCA
AGATGAAAGCGGCCAGTAAGTAGACAAGAATTAAATGATAGTAAAGGTAAATGAAATCCT
GGCAACACTTTTGTTTGAAATATCCAGCGAGCGAGAGCCCTCTATTTGTATAGAATACTCGT
AAACAATGGCCTATTGCTAGTTTTCTTTTTATTTCTTCTGCCGTTTTCTTTTAGGCTAAAGTTC
TTCCGAAAAATGATCGCTGCTTGTGTGCCGGATTATGGACAACAAGTCCTCCCTCATTCCAA
TTTGCTTGATCCTCACCTCCGGAGCCAGTTTGCGTGCTTCTTTGTCTTTGTTTGAAGAAAAGC
CCAGTTCTCCGCTTTTTTTTACTTTTCTTAGTAACCCTCTACGTACCTTGATTTATTTAATCGTT
TTATTGTCAACAATTTTTTGATCACGTTGCTATTTCGTTGCTTCGTATTGTTACTAGAGAATGT
GCTCGGCTATGCGCACGCGCTGCCACAAGTATTTGTTAGTAGTTTACATAGTCACGGTGCTG
TTTCACCGTCCTCTTGTACGCCGGTGTCCCCTTTCGTCTCCTCTACCGTCTCCAATTTAGGTAT
ATTTATATTGTAAAGTATGTCTCACCCTTTTGTTCTTTCTTTTTTCTTTTTCCACCCAAGACAA
CGTAGTTATATTTGGACACTGGTTAAATCAATTTTGAAATTCGCTTTCTTTTAATTTTATTCAC
CAAAACTTTCGAATATAACTAGTGATATTTTAATGTTGTGCAATTTTTCGATATATTTAATAC
CGTATGTTGGAAATCTGCCTGGTGTCTCACAGTTATGTATATATTGTGATCAGCGAAGAAAG
TGTTTAAAAATCAAAGTGAAAACGAAATAGAAATGATTTTTATGAGACAAGAAAACACTA
TCTCTATTCAAGACGACGAGGTTTCAATGACTGGTGTTCCTTGACTTGCTGCAGCTTTTAGCA
ACATCGACCCGGACTCGGTAGGCCCTCTGTTGAAACTAACCAATCAGAAATACGTTGACCC
CTCCACTTTGGTGTGGCAATCATGTTGAGCATATGTCGGATGTATTCACGTTTGCTGACAAA
ATCTTAATATTTATTATACTAGGATAATTTTTGTCAAAGTGATTTTTCCAAATAATTTTTTGGA
AAAAATTAATCAAAACTAACAAAAATATGAAGATATTTAGTGGTATTTTTTTTGAATAGCAT
TGCATTAAAATTGTCTAGAATAAATCAACAAACTATTCCATCGAATATGAATCATAACTTTC
TTCAGTTTCTGATAGAGCCTAAAGATGGCAAATCAATTTTAAATCGATATATGAAGTTGTAC
TCGTCTGCCATCTCGTGGCGCTACGTAAAAAATATTTATCGTACCGATATAACATGGCGTTC
ACGTTGTGGGCACTTTTCGAGGTAACCGTGTTATGTTTGAATGCGGTCTGTATTTTGAACGA
AGAGAGATTTCTTGCAAAAGTTGGCTGGGCATCGTGGCAAAATGTTCAAGGTTTTGGAGAA
ACTGCTACAGCTAAATCACAAATTTTGAATCTTATTAAATCGATACGAACGGTAGCACGAG
TTCCATTGATATTTTTAAATATCATAACAATAATTGTGAAACTGGTGCTCGGTTGAAAGAAA
CAATTGATGATGTAAATAAGAGGATAATGCACAGTGAAAAGTCAAAGCGTGATGTACATA
AATGAACAATTAAATTCTTTTATTTTTATCGCTGCATTAATAAGTGAAATGGTTCTAATTATA
ACACATTCTTAAATATTTTTATAATGCAACTATTTTAAAAATGTAATTCTTTTCATTTCTACA
AA

352. >MSTRG.7501.2 Gene=MSTRG.7501
CGCGCGCGATCCAGACTCAGTTACGCGGCGGCCGGCCACCCGTGCGAGAGCGTATCGCTTT
CTTGCAAAACGTGCCCGCGCTGTTTTTCACTTTCTTTCTCTCTCTCTCTTTATCTCTGTCTCCTC
GCTATTTCTCTCGGTTCAACTTCCTTTCTCTTTCGTTCTTAACCGCGATCAACACCGCCGTCA
CAACACACGAGTCGCGGTGTTATTTCTTTCGATCCGTGTCTAAGGAAAAAAAAAGAAAAAA
AAGAAAGAAAGAAAAAGAAAAAGAAAAAGAAAGAGGAAAAGGATTGTGCATCGAGTTC
TCGTGTGCAAACGGATAATCAAGAGACAGAGCGTGTCAAGTGCGAGGAAATCAGGGTTTA
CGTACCCTGAAGAAGGAAGGAAGGAAGAAAGGAAGTAAGGAAGGAAGGAAGGAAGGAA
GGAAGGAAGAAAAAGTGGTGTAGCCAAGGACGTTTGTGTTTGAGTTTCGCGTGACCAACG
GAAGAGAGGGAGATTACTGGCGCGTTTGGAACACGCGTGGTTCCGCGTGTTACGGGACTCT
GTGCGTCCTCGCGATACGTTCTCCAACGCAATCCAACCCGTCGAATTCGTGATATCATCCCA
GTAAGAAAAATCTGTTGAGATCTGGTTTCGAGAGGAAAGAGTGCACGCCGTGCCGATGCC
GAAAGGATCCGTCGACACGATCGACTCACGAACGAATTTCACGAGAATTTGGGAGAACGC
GACGACGTTGATGACAGTGACGACAAGGATGTCACCGTTCGGGGCGGAGGAGCGATAAAT
TTTGGAAGGAACGAGGGAAAGATATAACAGGATTCTGCATGCGAGAAGTGTTCGAGCTCC
TCGGTGGAGTGCTGACCTCCGAAGACGTATTCCGGAAGGATTCGTCTAGCCCTACGACAAC
GCAGACAGGCATCTGGTCGTCGATCAAAATCACGATCGCGATCACGATCAACGAGATCGA
TCGACGACGATCCATCTACCCACTACGCCGTACCACCCGGTCACCATTGCGGCGGACACCA
ACAACAATCATGACCATCATCGCAACTTCGAGCATCACGCGGCGCAACAACGCCACGATC
ACTGGAACAAGTGTAAGTCATCTAGTAAAAAAAAAGAAAGCGAAAAGCAAAAAAAAAAA
AAAGGAGGGAAAAACGTGAAACAAAGACCTTGAGACAGTCCTGTTTTAACCATTGTGCAT
CATTGTAAAAAAAAAATTGTACAATGCTCGCTGATGTCTGGCAGGTTGTGGTTAAAAAAAG
ATGGTGGTGGTGGTGGTGATGGTGGTGGTGACGGTTGGATGATTGACAATGATTATCGATC
GAGGAGATGGATCGACGTGTTCTATTTTATTTACTTCGCGAAACGATCAATCGTTCGCTTTA
TTCCAAGTTCGTTTGCGTTTTCTCGATTCGATCGATCTTCGAACTCGTCTTTTCGATTCGATTT
TTCTCTTTCCATCGGGGGACGGGGAGGGAGGGGAGGATAGTTCGACGAGAGACCTTTTGAT
CGAATCGATCGAGAGATCTAAATACAATGGCGTACGTGCAACGAATTCGCGCGCGTGTGTG
TGCACCGCTGCTGTTTGGGAAACGAAAAAGGTTAATGGGGATGGCATTAAAGACGAAGGA
ACGAGCGAAACGAGGAGATGAGGTGCGAACGATAGAGCG

353. >MSTRG.7508.1 Gene=MSTRG.7508
TGTTTCTCCGCGTAGAAGGATTTGCCGCACAGGCCGCAGGTCCATTGCGATGGCCTGTGCTG
ATGTTTCGCCTTTTGCTGGGGCCGAAAAATGTCCCGACTTTCGTGGAACGGACACTCCAGCG
GCAATTCCACTTGATGTTTCTTCAGGATTGGCATCCATCTCTTCTGCACTATCCTCCTCACCA
CGGACGCTCGATCGCGAGGGCATGATATTTTAAACACGCTGGGCCCTGGCCACGGGATTAC
CGATAATAGTAACACTATCAAGACCTCGATATGAAAGGGTTGGGTCTTCATTCATAGCCCA
AATTCGCCTAGCGACAGTGACCCGGTGGCAACGATAACGGCAACCTCACGGCCCGACGAG
CAACACGAAAATCCCGTTCCCGAGTTTTCTCGTCGCGATTCACGGTCAACTGGGGACGTAA
CGGGGGTTGCGGCGAGGACCAGGTACAGG

354. >MSTRG.7538.1 Gene=MSTRG.7538

GGATGGGATTATTTATATAAATATTTACAAAGCAATTATGTCACTTATAACTATATGTAGCC
AATAAATCGCTGAAGTCGAGTCTTTCGCGCACTTTTGATCGTTATTTCCGGATTATTTCCACG
TTCACGTCGATATATCGCGAAGAATTTCCGTTTGTTTCATCCTTTTACGATCCTACAAAAAAT
TCCATAATCGTTATGGAATTACTGCCATAATTTTTTGCACGACAGACAGGGATATCGCGTAT
TTCGTCGAAAGTGCGCGAGAGACAAAATTTGCATCGCTCAGGCATACTAGAAATCTACGTC
TTATTGTCGCGTCACAACTTTTTTCGAAACGGCACACTATATAAATAAACGAACGCACGTTT
TTTTTTTTACTCTCGCTCGAAGGAACGCGAAGGAAGATCATCATTTTGCAGCCATCGATCAA
TAAATAATCGATTCCTCCCTCGACTCCGGAACGTGGTTGCGTCGAAAGAAAAAAAGTAATA
ACAATCGCTTAAACAACAATTGATACTCGCACGCTTGCATCTGGATTCTGCACATTCTGGTC
TGTCGGATCTTAACAAGTCCTCGAGGCTTGCGGTGTAAGCCGCACGCGGAGTAGCAAAGAG
ACTACGAAATGGAAGACATGGATTTTGTTTCGTTTCGCTTAACAAACGTCTTAAAACATCTC
CTTTTATTTTCTAATTCCCTTATTTAAATACAGGTGTCGTGTAAAAGATTATCCATTTACGAT
AAGAAGAAATCTTACAGAGCTATTACGCGATGATGACGCGACGATGCACGCGTTAGTTGC
ATCGCGGCCACGTTATAAATATCGTTTTATTGCATAATACACACATCGCACTTTAGCGTCCC
GTTCACACGCCTCGAGTCGGAACAATGCTCGTAATTGTGTATCGCCTTCAGAGTTTCCTGGA
CTGTAAATGCGCTGCGAAGGAAGTGTTCTTTCTCTTATCGTGGAGACGGTCGATAGTCCGTT
ATCCAATGAAACGCGTCTTTGTCTCAAAGAAAAATCCCTCTAATATTGCAGTGCGGTGAAA
AGCTTTTTCCTTGGACGTTAAGCTCCCCCCCATTAAGCGCGTGAACCGGACCTAGCGTCTAG
AGAAAATCTTAAGCCTAAGAAAGCAATTAAGCCTAAGGAAGCCACGATGATCGTTTAAAA
AAAAGAAAAAAGAAAAGTTCCAATTCTGAATCTCGCCGTGATCTCTCGTGATCGATGCAAG
AGAGCATCTTCGGATAAAAGGGGAGCGAATTTCCCGTATATATGTATATATATATCTTATCA
TCTCCTATATCTTGCGATATGGACAAATATCTAACACGATCGAAATGCACGCCCCTTCTCTC
GACGAGGATAATAGATTAAAAAAAGAAAAGAAAAGAAAAGAGAAAAGACACGAGAGCG
AAACAATGCGTTTTTTATCAACTAAATAAGACTAACCTTACTTCCTATTATCGGTACCAATC
GTCCCGAGACTAATCTACGAGATATGTGAATTCGTCCATCTCCTCTTCATCCGTCCCGTCGC
GTCCCTCCTTCACTTCAAGGTATAATGTCGCAATGACAGGCGCTGCCACCCTTACCGTTGTC
GCACTGGCTGATACACCATAGATCCCAATCCATGGAATCCTCGTCCATGGGCGAATCATTA
GGGCTCAAATTATCGGCGGTGGCGTTCTTCTCCGCGCTCGACGTGTCGATCCCCATTTCCTC
GCTCCTCCCGGAAAGATTCTCCTCGCTGATCCCACGCTGATCCCCGACGGAAGAAGGGAGA
AGTGGCACGGACCAACGCTCGTCGATCTTCCTCGACGCGGTTCCGTCGATCTCGTTCAACCG
ATACCTCTCCAATTCCTCGGCCCGTCGACCACGATCCTCCTCCTCCTCCTCCTTCTCCTC
CTCCTGCTCGCCGTCGAGCTTTCTCGCCCTCTCATCACGATCTACGTGGCGACAGGAGCAAA
CGTGGCGCTCGACGAGCGACTCCCTGAACGCACGACAGACGGGCGTGTCGTCGTAGCGGA
GGGTATCCGGCGAGGCGGCACCATGACACGGGGTCAAGCAGAGGACGAAGAGATACGGG
GACCGGGCTGCGCCGGACTGAAGGACGAGGTGGTCCCGATGGTGGTCGCAGACTCCCGTG
ATTTCGGGGCTGGTGGTGTTCCGTGCATGGCAGAGGTGGACGCATGCGTGGTGAACCGGAT
GCGCGGCGACCGTGGCCAACAACAACGACAGCAACACCCACCGAGGTAGAGGCGTCATCT
ATGATATTATTGAAAAACGAAAGAACAGCAAGAATAGATTCTGAATGATCGTGAAGTTGA
TCGTGTGAAATAATCAGAGGAATTGTTTTAACGACCCAGGCTACTGGAACTCGACTGTTTG
ATCATCGACGATTAACTGTCATTGGTGATACTGTTTTATTACGTATAAACGCATTCAGGAGG
CGCGAATAACGAAAGATAACGATGGACGATTCGATGTTCATTTTTTTTTTTTTTCAAGAAGG
ATCACTGTTCCCATTCGACTGATCCACGAAAATTAAACTCGAATGGCGTACTCGACGTACA
ACGATACAAATTATCGGATAAAATGGAAGCAAGATTAGTGGTGGAACAAGGAGGATCGTT

GAAAGAAAGGAACGAATTCGCATTATCCGGCGGTGTCTGAAAAACGTACGGGAAGAGGA
G

355. >MSTRG.7547.7 Gene=MSTRG.7547
CGCCGCGACGCCTTCGGAGACGTATCGTACATATACGAAACGGTGGAACGGTGATCGCCG
GTGAACACGAGGCGATTCACATTATCCCTCGAGGACCTCGATTGTGCTGTGTTTTTGTGAT
TGATCCACCACTATAACGGATTGGAGAACGGAGTGGAGAAAGGGAGAAACGAGAAGATC
AGTTTGCAAGGGGAGGCGAGTTGCGAGCAGGGGCGGAGGAGGACTTTGGAGGAGCTGGG
GAATGTTCACGCCAGCCGTGCGGAGATACAGGTGAGACCGTGGTGAAACGACACACGTGC
TCCAGGGATGCGGGAAATCGCCGCCCCATTAGAGTTCCATCCCGTGAGGCGAGCGTTGCCG
AGCGGAGCGTGAAACGAAGCGTATCCACCTCCCTTACCGGAAAACCGTGAGAGGATGCGA
TTCACGGGAAAGCTCGAGGGATAATAAAATATCGGAATCCTTCCTTAAACCATTTTCTTAA
AACCCCCCCGACCGCTATGAAGGAGGGAAGGGTAAAATTCTATCGAGGAATCGTCGAATA
AATATGAGGAAGGTGAAGATTCTCGGGTTATCACGAACCCCTCTCATCGCCGAGAAAAAG
AGCACGGACAGTTGGACAGGCACGAAGTACCTTTCATTACCGCGTCTCCTCGATCGTCTTA
ATCCCGGTTGCTCGATCAATGCGCACCAAGTTGAAACTGAGGGAAGGGTGTGTTAAGCCGT
TGGAATTGGACGTCGAGGTAACGATAACCGATCGAACGAGATAGTATCGTGCCTCTTTCGC
GCCCCGCCCGTGTACCGTTTCGAGCCGTGAGAATAACCGTTTCGAGTCGATCGACTCGAAC
GAGGTGAATTTTTGTTGTTGGAAAGTGCGTCGAGGAAAGTGTGTGTGCAACAGCAGTAAGT
AGCAAGTGTGAAAAGACAAAAGCTACACGGTTCGTTCGCTTTCGTTTGGATTATCTCATCTT
TCGGTCGGTCTGTCTTGACCGATTTCCATCCCCGTTCCACGATGCCGTTCCTTCCATCGTAAA
AAAATATAACGGCTTATGGGGGACGACGGAGGGACGAAGAGTGAGAGGGAAGCGGGCCG
CGATATATCTGCACACCATTGGACGGTATAATCTCGTAAAAGCTGCACACAAGTGTCCATC
TACGTGAGCCCGTACGTAAAGCCGTCGACGAGGATCACGAGGTCTCTAACCAGGAAACGT
GGTACGCCGGCCACTTTCGACCACGCTTCGTTCCAACTGTATTCCACGTGACAGAGGAACG
TTTCCTCGAGGAAACGATCCCTCGAAGAATATACTCGGTCACGGACGGTGTTGAACCGTTG
GGTGATCTCGTAAACCCTATCCTGAACCCTCACCGTCGAGACTATATTTTTGGATGGATCTT
ATAATATATACGAAGTGACAGTAAGTTCTCTGTGATGCTTCGCTTCGTGAAAGCTTCTGGCT
GTTTCGAGCAATTTTATCGTATTTATACGTAAAAAAAAAAAAAAAGAAAAAGAAAAAGAA
GAAACGAATTAGAGGTATATAATATTTTATAATAACTAGATTTAAAAAAAAAAAGATAGA
TTCAATTTTGTTTTTAATTTTGTAAAGGAACGAGAGTTTGTAAGATTGTAGAAATTTTTACAC
GAGCGATCGTAAAGTGATTTTTTTAATATATATATATAATATTCGTCGTATATTGAATAGAA
AGAAGATTGTAGTTTTTGGTAATTAATTGGTAATGCGCGAGTGCCACGTTCACGTTATCTGA
ATCACGGAAGGATGGATCAAGTGCACGTGAAATCGTCGAGATCGACGTCGAGGAGCAACC
CGGCGAAAATGGCGTGCGAGAAGGCGAGCAAGAGGCTCAGGACGATTCTGGTTAAGGCGT
CGCGCCTCGGCGTCTCGACGACGGAAGTTGCCAAGTTGGCCAGCGCCAAGAAGCTCATTCC
GCAGAGAGATCACGGATGGAAGTTGGCGGTGTTCCTCCTGGTCATGTTCGGCGGCGGAGTG
ATCGTCGCCTTGGCCTGCACGGCGAGGAAAGGCTGCTCGAAGCTGGAGGAGATCATTCAG
GCATGTGGCCAAAAGCACGGACGTTGTCGTTATCATCGTCATTGGAGCATATGCCGAGCGA
AGAAGTCGAGCAACCGGAATACCTCTCTACCAAGCATCTTTTATTCCGGGTAAGCAATATA
TTTGCTGTACAGTATTTTCTATCAATCTGTTAAATGTACCAAAAAAGGACCGAGCGTTCATT
TTCGTTTATTCATCGCAAATTGATTCATCGATTTTATCCGAAACATCGTCAAAACTTTTATAT
TTGCCGAGTACTTTAAATATTCGGTATATAACCAATATTACTAAGATATTGGAAAAGAATG

GAATATTTCCGGAATAGAGAAAGAGAGGGTACGTATTTTCAATGTACGTGAAAGACTGGAT
GGACGTTTGCAACGTGTATCGAACTCAAAGTTTCAAGATGAGAACGCATAACGAAGTTACA
AAAGAGGCAAGGATAAAACTTGATTATTGAACTTGTTATTGGTTTCATCTGTTCGGAACACC
TTCGCTGTTTTGCCAGTTCTTTTCCCCCTTCCCTCTCCTTATTTCTTTTTTTTTTCTTTCTAGTTTT
TTTTTTTTTTTTTAGTACGAGCAAAACGAGAAGTTCACGACCCTGTAGCCTATATTACATCG
ATTCGATGAGAGACCATCGAACTTACTTTGAAACTTGAAATTGCATATTATTGGATTCTATG
GACGTCCACGATATTTCACGAGAAATACAATGACCACGTCCAAATTTCATCTTACACACAG
AATACAGCGTATAATTGCCACTATGATTATATTATCATTCATTTAATACACAT

356. >MSTRG.7582.6 Gene=MSTRG.7582
GGCTAGCAAGCCTCGCTTAAGTGTCGCTCGACAGTCGAAGCCGGTCTCTCGGCGTGCGAGT
ATCGAGAGTATCGCGAAAGGGTTCGTTCGAATTTCGTTCTCAACCGAGCGACGCGAGCTTG
TCACCTGTCAACCTTCGCGAAAGAAAGTTCGAAGTCGACCGAGACTTAACCTCCGCTCCGT
TTAAATCCCTTATCGTGTGTGTCGCGCTAACGAACCATCGAGTTCTCGTTGTGCGTTCGTTCG
TGTCGTGCCTGTTCGCCAACCACGTTTCCGCTCGCGTGTGTCCAGTGTGCAGAAGTGTATGG
ATGCCACGGCGGAAGCAACGCTGCCGAGATTGCGAGGACGTTCCTCCTGGCCGGTTAACTC
TTAGAGCCACCGAGGCGTGAGTGTCTACGCGAGTAACAGTTTTCCGACTCGGTTCCTAAAA
CTTTCACGGTTATTCGCCCGGGCCCGACAACTTCGTGAGAGATTGTCTTCTCGTATATAGAG
AACGAAAGCTGATGAATAGCGAACGGAAGTCGATCGTCTCGTCGGATCGGAAGCTGTGAA
CCTAGAACGTTCCCACGATTCTCACCAAGGTGAAACGATGCGGTGATACCCGAGGAGCCCC
GCTTCAACACCCGTACATATCAATTCATATCTCTGCCCGATAAACATCGCCAAACATCAAT
CGACCAATCAAACGAAGCATATCGAAACGCAATGAAGACCCAACGATGATCGAATCGCG
GCCAAGGCATATCAGGCGTCGATCAAGCGTGATCGGC
CAGACTGTTCAACCCACTCTATCCTAACCAGAGACCTGTCCATAAACCTTGCACCACCCCC
CGGTCGACGACACCAATTGAATCAACCTTGGTCCAACCGCCTCTCCCTCCCCCTCCCCCCTC
CTCCCCTCCACCACGTCCCATATCACCACGCCCAATCGGAAATCAACGGAGAGAAGCAAA
TAGAAGGAAGGATGTTGCTGAGGTAACGCGGCCTCTCGAGCCGGGGTTAAGAATCGATCG
TTAAATCGAAACCGTGCTCGAACAACGAAGGGGATACCAGGTGGCGGATTGCATAGTGGA
TTCGCGTAGGGGAGAGAGATCCAGAGTGAAGGATGGGTTTCCCCAGGAGGAGATCCCTGT
GGTTGAAGGTGGCCATCCTGGCCACCGCGGTTTGGGCGACCGTGTGCTTCCTCTTGTACACG
GAGGACCGTGCCAACGCGGCTGTTCAAGGGTTGGCGCCGTCAGGCGTCGCGGCCCCCCAA
ATGGCCAACGGTTTCGTGCCCGCAGCCGCGCCTCTTAGAAAGGAGACGCCGCAACCGAAG
CCAAAGGTGATTCAACCTGGACCCGAGCAAGGTAATTAATCGTTTGTTTATGCAAAGGGCG
GTCTTTTGGGAATTTTGCTTCAAAGGAAGTGGAAGGGGGAATGAGAAATTTTTATGGTAGG
AGTTTGGGAAGGAATTTCTGGAGTTTGTTCAGAAAAAATCGTGGAAATAAATGTGTAAGAT
GAGAATTATATAATACAATAGGTTAGGATTTGGGAATTGAAGGAGCAAGAAGGAGTTTCG
AAAGTTATATGAAGAAATAACCAGGAGCAGGGATATGGAGGTTATGTTTCTTGGCTGTATT
AAGCCAAGATGTGGATTTGGCATTTGTAGCGTTATGAATTTTATTCTATAGTTGAATTATAA
GGGAATTGAAATGAGGGAACATTTTACAAGATATTTTGTATATATTTTGTATTTTTTTGGATA
TAATATTACACAAATTTATGAAGTTTTGTAATGAAATTGTGCATCATGTACGAACATCGCCA
TAACCTCCAAAGCTATAAGCTTCAACCTAACATTTTTGCATATCTATCTCTCCTCCAAACTTC
ATCCTTCAAAGATATCTTATATTTTTTACAGACACCTTCCTCATCGTTAACTTATGGTTGGAA
TCATAAATCGAGGGGAAAAAGAGCAGCATCTTTCCAAGTATCTTTCTCTCTCTCTCTCTGCG

TCGTTTCAGGAACAAAGAACGTCCGATGTACAAAAGATTGCAACCGGAATTTCGAAAGAG
AGTCGAAGTGGAGGGCGGGGGTGGTTGATACACGAGGGTAGTTTCACCGCGGCAAAGTTC
GCAGCTCTAAAGCAATTTGTCGTGCTCTTTATTGCCGCATCGCGCGCACCGGGGCCCACCTC
GCTCGACCGGGATAAAATTATTTCCTAGACGAAGCCAAGATGCCGTGGAAACGTCTCTCCC
CCCTCCATCCCCCTCTGTCTCTCTCTCTCTTTCCCTCTGCCATCTTACCTATGCCTTACCGGTG
CCAATATGACGGGCGAAAAATAAAAGTCGAATTTGTTCCTGAAGGCTCTTAGAAAAGGAG
GCAGGTTGGATGGATACATTGTGAAAATTCAATTCGCGTCGTCTTCCGTTTTCCGCCGTCGTT
TCTGCAATCCATTCCGCCCTCGTTTGTATTTCGTTCGTTTGCCCTGTGTCTTTATTGCCCCCGC
GATAAAGAAATTGGATTTGGAAATCATTTTGTTAGCGGATCGATAATAAGTGGAATGAGGC
AATGTGGTTTTTTTGAAGGAAGGGAGAGAAGAAAAATAATTTTGAGCATATGTAGATCAGT
ATCAGTTTAGAGGATAACTATGTTTGAAGAATCTTAAAGAGAGAAGATATTTGTGAAATCT
TAAAGAAATTTTGTTTTTTATGAATTTTATGGTAGATAGATGAAAGTTCTTTTTTTTTTTTTTT
TTTTAAGTTTAAAGTTAGAAAGCGGAAGTTATCGAGGATTAAATTGTACGTGACATTTGTTA
GAGAGTTCATTAAATAAGAAGTAAGAAATTTTCTTCTACAATATTATGCGTGTATTATTAAC
AAGTTCTCTTAAGATAGAACTAAGAGGAACTTCATTATTGAACGTTATGAATCACTGGGAT
GTTCATGTAACTCTTTTATGAGTTTAAACTTGGGAGATATATCAGTCGAAGTTGGTGAAGAT
ATATAAGTTTAGCATTTCAAGAGCTTAAGATTTATGGTATTAAAGATTGATGCGCTTTTATC
AGTCAAGAGAAGTTATCCTTGACGTTCAATGCGATTTTATACATTTAAAGACTTTAGTTATT
AAACAACAACCAATAATAATAGAATCATTTCTTTTTAATGAAATAATTTTCAAATTTATTAA
TGCTAATTATTTAGTGTATTATATCTTTACAAGAATCATTGTTTTTTTTTTGAAGAAAAATAA
ATAAAGGATAAAGTTATTTTTGCTGACAATTTTTTTTGTATTTCTTTCTTTCTTTTTTTTTTTTTT
TTTAGGTGCTGGTGTGCTTGTTGCTCCCCGTGAACCAGATGCAAGCGCACCTGGCGAGATG
GGAAGGCCAGTGATTCTGCCAACGAATTTGACTGCCGAAACTAAAAAACTTGTTGACGATG
GATGGCTAAACAACGCGTTCAATCAGTACGTTAGCGATCTGATTTCTGTTCATAGAACACTG
CCCGACCCTCGTGATCCATGCCATGCAACCGAGTTTTCGGCGCGTCGCGCCGCCTTTTGCGG
AATTAACCCAACGTCGTCTAATTAGGATTATCTATGGCCATCCCGTGAATTTACACTGCGAA
ACATCCAATTAAGGCGGCACGATAGACAATGGGGATTGCACAGCGGCGGAATTACGTTGC
CAAGGACGGTCCCATGCAAATAGCCTTATGTATTCCCAACGATCTACGTACCCGCGAGAAC
GGGTACGAGACGAGACCAACCAACTTGCGCTTGCGGAAATTGGAAAAAGAACAGATGGA
CGACGGGGGAGAGGGAG

357. >MSTRG.7599.1 Gene=MSTRG.7599

GAAGGGGGAGAGTAGTAAATTCACGCGAGTGTTTGGGGGGGGAGGATTGATGAGAACGCC
TCTTCTCCTCTCTCTCTTTCTTTCTTTCTGGAACGAGACTACCGAGCATCGTTTCTTCAACGTT
CGACGAAATTGTGTTTCCAACGACACCAGGGAATATCGTCGTTCAAGAATCGATCGATCGA
AGAAAATTAATCAAAATCTGTGAGATTCGTGGGATGGACTTACGGATGTAATGATAACATT
TTTTAAATGGCGAATACGTGAATGAACCGGGATTAAATTGTTCCTAAAGTTTCCAAGAAGC
TCGATTTCGTGATACCAATCAAGAAATACTAATTGGAATTGACGCTTGAAAATTTCGGAAT
GAAATTATGATCTTTCAACTGTGGAGTTTCAGAGAAGATTGAACAATACTTAATGTTTATGG
AAAAATTAACTCAGCGGGGTCCAAATGGGGAACAATGGGGAGCAACGGTTCGAGTGTCGT
CTCTCTCTCGGGAAAAGTGGAAAGAGTCGGAAAGTAGGATGAACCATGCAACGGTCTTGA
TTCCTTTCGGCCGCCATTTCTAAGTGGCATCCAGAGTTCTCGCGTGAGTTCTTCTCTCCGTTC
ATCGCTCCCGCACCTTTCTTCCTTCCTTCCTTTCTTTCTTTCTTTCTTCTTCCGCCCGCCAAAGA

TGTCCCTCTCCCCTCACCTCTCGCCATCGCCAGCCCGGCTCAGCCTATTTTTTCCGCGGCTTC
TTTTAATGGCCTCTCTCGAGGTCTTATCGGTAACTCGTTCCCTCGAATCTTTTCCTCGATGAA
GAATCTCTCTCTCTCTCTCTCTGTCTGTCTGTCTGTCTGTTTTCCAGCGCTCTCTAGCAACGTG
GCGACGCTGTTGCATAAAATCGGCGAGGAAGGAGGGAAAGGAGAGAAGGAGGAAGACGA
AGAGGATGATGGGGCGCCACTGTATAAAGCATCCGAACGTTACGGGCGGGCCTGACTCTTT
GCTCTGCCCGTATCTTTTTTTCCACTCTGGGTGAGCAAAGTTTCAGGTGTGTCCGTGACAGTC
TCTGTCCTCGCGCGATCAACACCCTTTTTGGACGATCTGCATCCAACGGAGAGCGTGCTGCT
CGTGCTTCATCGATCGGGAAACATCGACGATTCGAAGTTGTGATTGTAAAATTCTATTGCGT
TTGATGGATGGATGTGTTGTGAAAATAAGAAAATAATCTTTTGTTATTGGAGTTGTGAAACT
GTGTTTTTTCGATCGATTTTTGTAGTTTTATAGAGAATAATTTAATAATAGACTCGAGGATTG
ATCGTTGGAATTGTTGGTCTTTGACGAATTTGTCAGCAAGTTTTATATAAGTTTGAGATTATT
CGATAAAGAGAGTGGAAGAAGTTTTAAGTTTTAAAAATTAAGAGAAATGGATAAATTTCG
AGTAAAATGATGAATTTTAAATATATATGTATTGGTGGTGGAATGTATATGAAACGATATTT
AGTTAAGAATTAAGAGGATGACACGGATGAGGTATATTTCCATGGCAATCATCGTATCCTC
GACAGTACTCTTACGCCTGAAATTTGCTTTTTACCTGGCCCTCGTGCTCTCTTACTTTGAGAA
AATAAAGGTGAGCAAAGTTTCAGGTGTGTGAGTGCGTTGGAATCAACAAATAAAATCCGG
ATAATGAAACGTTAAGGCGAAAGTTTTTAAAGTTTCTTCGAGACAAATTTTTATTAGAACGA
TATTTACTCTATTTTTATTATTTAAAAAATTTTATGAATATATTTATTGTATTTTTCCCAATTA
AAAAAGTATCGATGCGTTTAAAGTCTAATGGAGAAGAAATAAAAATTGTATTAATATAAA
AAACGAATAATATGTTATATATTATTATTGTATTACAGTATATTCATCGAA

358. >MSTRG.7738.4 Gene=MSTRG.7738
CGACCGTGTTGTGCGTGTATATCCATCGGAAGTTTTGTTTTATCCAAGTGGGAGTTTGTAGCT
GAATTTTTTAAACGAATAATTTATCTTCCTTAAAAAGTTTTCAACAAGAATCTCTAGAGTGT
ACGCGCGAATCGAGGGTAGCGTGCACAGATTGGGAGTAATCAAAAAATAAGAAGCGCGA
ATTTTATAGGTTAGGTTTTTCGTAGTCACTGAAAATAGACTTCTGAGCGTGATTCTTCGATCT
AATTAATCGATTAACATTATTTCTCGAAAACAAAAGTTTAAAAGTTCTAATTCAAAGTAAA
ACGAATACAGTTTTTACCAAAATCGCGCCGATATTCTGATATTCTGCCAAAGCGACTAATA
CTTCCGATCTGTGTTCAAAAACTTTTTAAGCGGATGTTAATTCGATCCAAACTCCCAAACGT
GGTCGTATAAAAGAAGCTAATACTACCAACCCCGTAATTTTTTCGCCTCGAGTCTCTTCGTA
CCAATTCCTTCTGGTGCCAAGAGATATACAAGAAAATGGCGTAAGTGCCAGCGACCTCGGT
CGTGATGAAGCCACTCACGTACAGCAAAAATGAGAATCCGTACCTGTAGGTGAAGGGTGG
CCCCTGAAATCGCAAGATTGCGACCAAGATATTCATTCACACGGGCCGTGTAACAGATGAG
AAGTAATTTTTAGGATACCGAAAGAAATATGAAACCGAGAATTTCATCAGCCTATGTAATA
TTCACATAGAGTTAGACGACACGCGAATTAAAATTCATCTACTGGAAAATGATCATGAACT
GACCTGAAACGACGATCTTGGTCTAAGCTTACTACCAACCTCGGCTTTGAATACAGATATGT
ACATCACCATTCCCACCAGCATTAACAAACCTGAAAGAGAAACTGATTTAATAACTTGAAA
CGTTATCTCTTGCGCCACCAATTCTCTTGTGACAATTTCACAAAGTATGGATCTCTCATCGTA
ACGCGATTATTTTGATGATGACTTTGATCTTTGATTTCTTCGCGCAAGTTCAAAGAGTGGATG
GACATGAAATACTTAATTCTATTCAGTTGAGAAGAAATGGCAGAGCGAGCAAAGTATCCTG
GTGCAATATAAGCTCGGTGTACTTTATTACTCACCGGAAACTATGAAGACCACGCCAGCGA
CAAAGACGCAAAGTCTGTGTCTCGGGTGAGTAACGTGAGCTGTGAAGTAACAAACTTCGGC
TACCACCAGCAGCGATGTTGCAGCGAAGAAGAACATTGCCGACTTGGTAACTGCATATGGT

ATAGCCATCGTCGAGTCGCTCGGATCAGGGCTGTACTCCTCGTTTGGAAAATAGTCGATCCT
CGAGCACTCGTATTCCATACGGGAGCC

359. >MSTRG.7747.3 Gene=MSTRG.7747
AAATATTATATAATGTTTATTTTTAAATTAAAGTATCTTTTTTTAGAAATTTATAAAGATTTA
GATATAGATATAAATGAAAGGTTTAGATATAGGATTTTCCAATATTTTTAGAATATTATATA
AATACGACATGATGATCAAAGTTTAATAAACACGGAAATGTGTTAATTTACTTTGATTGCG
GTACAAGAGCTGTTTGATATTGTGCAGTATAGTCTAAACCGCAATGTCGAAAAATAACTCT
GCTTTCTGAATATAGAAAGCAAAGAGGCACGACATATTCCATAATCTCTTTGAAATCCATT
GGGTGATGATCATTGAGACAAACCATGTCTGGCATAATTCTACATATAATATAGAAAATAA
TTCTATATAATATAATATAATATAATATTATAATACTATACTTACTTTTTTTGGTTTTTTTTTA
TTGTATTTTATACATATATATTTTTCTTTATTTATTTAGAGGTGAATAAGAAAGAAATTGTAG
AAGCAGTTACCATTTTAGAAACACCACCTATGATTGTAGTTGGTGTAGTTGGTTATATTGAA
ACTCCGCATGGTTTACGTGCTCTTACTACAGTATGGGCAGAACATTTATCTGAAGATTGTCG
AAGAAGATTTTATAAAAACTGGTAAGAAAAAAAAATTGCTATTTATATTTTTTTTTTATGT
ATTTATAGTATAGAAATATATTTTAATGAATCTTGAATTAGCTCTCCGCTCAATTTGATGAG
AGCATATATTTGAATCTTGCCTTAAAAAGAAATAATTCTTTTAAAAGGCTGATTCAAAATGT
GTATAGTCTCCGCTCATTACGATGAGACTAACACGTTGCAGATAAACAGGTATTTATAAATT
GAAAACAAAAAGCAGCCATTACGGTTCCTTTTCTACAGGTACAAAAGCAAAAAGAAGGCA
TTTACAAAAGCTTCCAAAAAATGGCAAGATGATCTTGGTCGTAAATCTATAGAAAACGATT
TTAAAAAAATCATTAAATATTGTAAAGTTGTACGAATTATCGCTCATACGCAGGTAATTCTA
TTACAAATCTAATTTATCTAATTTTTTTTTTAAAGTAAAATTTTTACTTATATAAAGTTTATCA
TAGATGAAATTATTAAGACAACGTCAAAAGAAAGCCCATATAATGGAAATTCAATTAAAT
GGTGGAACTATTGAGCAGAAAGTTCAATGGGCTCGTGAACATTTAGAAAAACCAGTACCTG
TCAGTAATGTTTTTGCTCCAGATGAAATGATAGATGTAATTGGTGTCACAAAAGGAAAAGG
ATATAAAGGTGTTACATCGCGTTGGCATACAAAAAAATTGCCACGCAAAACACATAAAGG
TCTAAGAAAAGTCGCTTGTATTGGAGCTTGGCATCCTAGCCGTGTGTCTTTTACTGTTGCAC
GTGCTGGTCAAAAGGGATATCATCATCGTACAGAAATGAATAAAAAAATTTATAGAATTGG
ACAAGGAATTCATACTAAAGATGGCAAGGTTGCTGTATGGGTCCGAAAAAACGTGTAATTA
CATTACGTAAGTCCTTATTAGTTCATACTAAACGATCAGCATTGGAGAAGATCAATCTTAAA
TTTATCGATACAAGTTCCAAATTTGGACATGGTCGTTTTCAAACTGCTGCTGATAAGACTTC
ATTTATGGGTCAACTTAAAAAAGATCGTATGCGTGAGGAACAACAAGCACAAGCTACTAC
CTCAGCACCATCGGCCGCAAAT

360. >MSTRG.7772.2 Gene=MSTRG.7772
GTTCTATATTGAGTATTTAATGATTTAATAATTATTAAATTATTTAATTATAGTGATATTTTTT
TAGTCACATATAAGGTATAAGAAATAATAATATATTGTTTTAAATATATATGCTATTATATT
TTATTATTTCCATATAATTTTGTAAATAAATAAATAATATACATTAAGAATTATTTAAACAA
ATCGTGTTTTAAATTTTAATTTCCTTTATATTAATAAATTTCGATTATTATAATTGTTAATTAA
ATATAATATTGTACATTAATAATTTAAATTATTAATGCGAAATTAATTTTCTGAAAGGTTATC
TTATAATATTATAACAATTGTCCAATATTATATCAATGATCAGATAAAACTTTTTTGCCTGAT
GAGAAGATAGATTTGTATAATTGCATTAACTTAATATTATTTATGTAAAAAAAAAATTATTT
ATTTAAAATATATTAAACGTTTCAAGAAATGCCACGATTACCATTACATCCTTCAACTGGAA

CTGGCATTTCTAATTTATGGCCTAAAACGATACGATTTGTTCGTTATGGATGTAGCAATAGA
CCTTTCTTTCATATTGTTGTTATGAATACTAAACATAGGCAATATAAACCTCCAATAGAACA
ATTAGGCACATATGATCCTTTGCCAAATAAGTATAATGAAAAAATAGTTGCTTTCAATTTTG
AACGTATACAATATTGGTTGGGACAAAATGTTACAGTATCAAAACCAGTAGCAATACTTTT
AGGACTTGCAGGTTTCTTTCCAATACATCCAAAAACATATATGATATCATGGAGGAAGCGA
AGAGCTATTGAAGAAGCTAAAGCAAA

361. >MSTRG.7825.1 Gene=MSTRG.7825
CGCCGTCTTCTCTGGAACGTTCACCAATCACAACAATGGCGAATGCAATTCGGTCAGTTCA
CTGAAACGCACGATTAATCCGTTGTAAAGCTAAACCGCGTCGTGGATTGATTCCATTTTATA
ATCGCATCTTCTTCGCGATAAAACACATCCGTCGTTATTGTTCATGAAAAAATTACTTCGAT
TTTTCTTAAGATATACCGCAGACAAACGCACAAAAGCAATATCGGCCGACATGTAAGATTT
TCGTTCGGAGCCACTTTAAGGGGCCAAGCGGGGTCGCCGATAGGCTGCGTGTGACGTGGTT
GCGTTTATTGACCATAACCCCTTCCTACTCCCGTCTGCAACTTCACGACGGACAGGTGTACG
ACCTGAGAGACCGCAAAACAATTGACCAAACCGACCGCGTCTTAAAATACACGGCATTGT
CAGAGAATCAGAAAGATGCTTGGAATGTTCGACCTCTTATCGTGTCCACTGATGGTGTTATT
ATTCTTCCTTTTCATCAATAATATGATTGTTTTCTGCAAAATATTCAACTCACGGTAATTTTTC
TTGACGCATTGAAAGACCGGTTTCTTCAAGAAATGAGAAAATTTTGACGAGCTTTCTTCGGT
TCTTTCAGACGAGAAGGTTATGTAAAAAGACCTTAATTATTCTATAACGTGTGAATTATAAT
CTCTAGAGGTATATAATAAAAAGCTATTGTTTCCAATGTATATTCCATCATATGAATATTAT
TTTCTTTTATTGCTAGTAAT

362. >MSTRG.7866.1 Gene=MSTRG.7866
AGCAATTAATAGACAAAATTGAGAAATATCTATATTAACTCTTAATATTTTTATATCACCTG
TAAGAAATAGAATCTAGACATTTGGTTTCTGTGGTTCTTCGGCGAAATGATGGCAGTTGATT
TCGTATCAATTAGAAAGTTTTAAAAATTAGTTTCTCTTCAGCGAGAAATAAAAATATTTTAA
GTGATTGGTAACGTGATATGTCAG
ATAATTACAAAATGTCTGTTGCTGAGTTTTTAAAGGGCCTGCCTTCTCACAATGAAAATAAC
TTCGCTAATTTTCATACAGATAGTGGAAATAGAACATGTGTAAAAAAACCTTCAGTTTATCT
TCCTACAAAAGATCATCCTTCAGAACAAATTATAGTTACAGAGAAAACAACCATACTACTA
AGGTATCTTCATCAACACTGGGACAAAAATCATGCTGATAGAAAACGTGATTTTTTGTCTGT
GAATGGTGATTCTGAAGATGATGCTGCTACAGTTCATAGCAAAAGGCCACGTCTTGATTTA
AACAATACTATATAAACTACAAACTCAGTTATAATTTCATTATTATTCACCTACCTACATTT
CCATTATTTGCTAAACTAATTTTCATTGTTTATTAAACTATATAATTCTTAAATATTATAAAC
ATATCTACGATTCAGAAAAATTATTTCAAAACGTTGCTTTTTAGATGTTAAATATACACAAA
TTGAATTAAATATTATGTGAATATTACAAATGTAGGAAATAACGTTTTCAAATAATATTTCT
TTGGATATGTGAAATCATGTAATAATAACAAGTATTACATTAATTTCATGTTCGTCGCTTACT
ATATTGTATATTTGATATTTGGTTCGAAAAATTTGTATTTAGCATAAATAATAATTGCTCTAT
ACTGCACAATATGTATAGGTGAACGTGTTCATTGTTATTGACAGCAAAACATGACTAATGT
AACATATGTGCGTTACGATTTAAGAAAAAATTATTAACATTTTATTAATTTAAGATATAATA
GAGCATTTATTGAAATAATTTTGTAAATTTGTTTTACAATGCACATGTTTTCAAATCTGTATT
ACATATTTATGTAGTAATAAGTTTAATGTACGTCTAATTTACAAAAAAATATATTCGATATA
TTATTCTTAAAA

363. >MSTRG.7931.1 Gene=MSTRG.7931
TTCGAAAAGGAAGCAGCCAGTCTGTTTCGGATGTTTGGAGTCGTAAGACGTAAACGGATAG
TATCTCTAAATGTTAACCGGTTCGCGAGAAGTGTGTCAAAGTCGATGTAACGTGTTCTGTGC
GTCCATAATCAACCGTAGCTCTAAGTGTCTTCGGACTTCGGATATGGTTTCTGTCTAATAAA
AAATGCCGGTGCGGTAGTGATCGTGGCCTCGAAGCGAGTCCTTTTACGGCGGGACGAGCA
TCGAAAGCGGAGGAGAGCAGCCTCCCTCTCCCCCTCCCTCTGTCTTTCTCTTTCTTCCCCCGA
CTTTGGCCCACGGCCGCCTTTATCCACCGGTCCAAGACTGGAGAAGATCGCGAGAAGGAA
GGTAGAAGAAGAGAGAAAGAGAGAAGAAAAAGGAGAGATCGAAAGCTAGACCAGTGTC
CCAGTGCGCTCGAAGTTGGCATGGAGGGATAAAACACGGTGCAGAGAATTGTGAGAAGAG
AAAGGGGGAGAGAGAGAGAGGGAGGAGGAGGAGGAGGAGGATTGGAAAAATTGGCGGT
GGATGGATAGCGATAAATTCGGCATGAGGTAGAGGAGAATTTCGAAGCTGGTTGGTCGGA
GAGAGGAGCGATCGATCGTGGAGGGATAAGGGGGGAAGCTAAATGCCAGTTAAAAGAAA
GGCGAGGAAGGAGGGTTAGAGGTGGAAAAGTGGTCGGGGATAGGAAGAGGCGGGTGGTG
AGGAGGGAGAGGAATGGGTGCCGGGATGGGCAGGAGCGGCACGAGCGGCGGCTTCCTCG
AGGCACTTCCCACGGGAACGCCGCTCCACGTGGCCATGCTGGGCCTCGACAGCGCGGGGA
AAACCACCGCGTTGTATCGGCTCAAGTTCGACCAATACCTGAACACTGTGCCCACTATCGG
CTTCAACTGCGAGAGAATCCGTGGCGGCATCGGGAAAGCGAAAGGTGTGAATTTTCTCGTA
TGGGACGTGGGCGGGCAAGAGAAATTACGGCCGTTATGGAAGTCTTACACCAGATGCACG
GACGGTATTATCTTCGTGGTCGACTCGTGCGATGCGGAACGGCTCGAGGAGGCCAAAATGG
AGCTGACCAGAACGGCGAGGAGCCCGGATAACGCCGGTGTGCCCATTCTGATCCTAGCCA
ACAAGCAGGATCTGCCAGGTTCCTGAACCGGTTCCTCCCGATGTCCACAGGTGCCAAAGAG
GTGGGCGAATTGGAAAAGCACCTGGGCGTGCTGGAATTGGCGGGGATGCCGGGGAGCGCG
TGCATCAGGGTGCAGCCGGCCTGCGCGATCACCGGCGAGGGGCTTCACGAGGGTTTGGAC
ACTCTTTATCAGCTGATACTGAAGCGGCGCAAGCTCGCGAAGCTGAACAGGAAACGGGCC
AGGTAGGCCAGGGCCTCGGAGGACTGCACGTGCGTCTTCTGATCTTGCAAGTCGCGGACAG
TCTCTCCTTCGGGCACAGCCACGCCTTCCACGGTGTCTTCTTCCTCCCCGTCACGATGCCGC
GGCGCCGACCCGCTCGAGTGAACAACGGAGCTGCGCGCGCATCCAACGTCTCCACGAACA
TTGCCCACTTCGCCGTGGACGAAGTCGTCTCGTCCGTTCCAACGTAGTTGGAATCTCTGTTG
CGTCGCGTCGAAGATCTTTCCAAGCTTTCATCGATTCGTAGAGGATCTCCTCTTTATTCCTCT
CGATCGGGAGATCTCGGTTCATCGAGTTTCGGATGACTCGAAACAAGGATGGAATTTGGAA
CGGGCGCTTTTTTAAACGCGCGAGGAGGAATGTCGAACGGGATATCCCTCTCCGCGAAGAG
GAGGATAAAATAATCTAGGAAATTTTTTTCTAGCCAGCCAGTGGATCGATCCACTCGCTGTT
GCGAGGCGGAGCGAAGGATGTCTGATTTACGACAACGAATCAAGTGCTTCTTCGCCCTCAG
AAATAACAGAGAAGCTCTATTTTGCCAAAATTTTCGAGAAAACGGAAACGTTCCTTAGGAC
GTTCCTCTAGGAAATACCAATTTTTAACGACCCCTTTCGGCCGATCGCCTCGGCCTTGTCAC
GGATAACGAGCTCCTCGGCTCTCCGTTCTTTTTCACACCGGCCGATGACGAAAGAAATAGG
AACGCGCGTTTTTTTTTCCTTTCTTTTCCTTTCTTTTCTTTTTCTTGCGCGAGCTCCAGGAGAAG
AGGATTTTCGCGATAACGAAGCTGCTGATAACAATGACGTTGAATCGTATTAATAATGCTG
ACGCGGGCCCGGGTCAAGGAGGGAGGCCAAGATAAAAGCCACGGGTATTAATAACCGTG
ACTCGAACCCCGCCAAATCATCGAGATTGATTAAAAATTATCTCCAACTCTCTTCCCTCCTC
CTCGTTTTCAAACGGATGATTTTTATCAATTTTCGGACAATTTTACGGATATCGTCGATTATT
AAGAGGAGAATTTTTTCCTTTTATTTTCTTTTTTTCAAAGGTAATCGCAAATTGGCCCTTCAA

TAAATTAATCGCGTTAGCAAGGCTAGGTAGTTGATACGCGTTTCGTAGAGCTCGAGGTGAA
CCACGAGAGTAAGAGAAAGAGAGAAAGAGAGAGAGAGAGAGGAAAAACGATCAAAC
GTCGATTGCTTCTTCCCGAACGGAATCTTTTTTCGGTAAAAACGATGTGATAACGATCCCCT
TGAAATATCGTACGTGTGTATATCGTGTTCGTTGTATCGTCTAAGGGGCTAATGCAGAAAAG
ACGAATCGATCGTTTTGAGCTCGATCGGAAATCTTGATACATATCTCGAAGAAACGGATGA
TCTATCCCAATGTATATATATCTTTTGTTATAATATTGTTCTTGGATACGTGATTCGTTGACTC
GATCTGATTTTTATTGCGTTAGTTTGTATATTGCGAGAAAAAATTTATTACACGTTTGTTACT
GTATATTTTTTTACGTCGTCCACGTTTCAAGGATGAATTTATTCTTAAAAACTAATTTAGAAT
TATTCAGAATTTCTGGAAACGGGTGGTGTGGAGCTCAAAACGGTGGGTGCTCGTGTTTCTCT
TGCCAATGTATAATACTTTTTATCCTTAACGGTCCCTTCCATCCGCGCGGACATCCGGTAGC
GTTCTTATCGACCATTGCCACCACCGGTAGTAACGCGAGACGATATTTTCTTAACGCTGTAT
AGGAAACGTATATATACGCGCCGGGAAAAATTATCCTCCCGACACTTGGACGCAAGCACG
CTCGTCGGTTTTCTTCGATCGTTCATTTTTTTTTTTCTCTTCTCGAGGAAGCAATCGATTTTAT
ATCGATTACACGAACGAATATACGGTTCCTATTCGTAGAAAATATCGTCGTCGACGTGGTG
ATTTATATATTTGGACATTTATATTCGGGAGAGAAGTCGGTGATTAGAAGTGCAGTTCGCCA
AACCAATCGAAGTTATTATTATTATTATTATTATTATTACTATTATTATTATTACTCGTTTTCG
TTATTTTCGAGCACAGCGCAAAACTTGTGATTTCGTTCGTGTTCTCGTTCGTGGCCTTCCACG
TCCCTTCCACGTTTCTTCCCCGAGGGCAAAACTTTCCACGAATCCTTCGTGCACGCTTTTCTC
TCCAACTTTTCCGTTGGAAAGGGAAACATCTCTCTCTCTCTTTCTTTCTTAAACTTTCTACCA
CTGTTCCTCTCCCCTTTCGCAAAAATCACTTCGATTCGAGGAAATCTCGTTGAGAAAGTCGA
GATACATTTACATTCGCGTCTCGAGAAGAAGTGCTTCGAGTGCCGTCTCGTGAATCAAGAG
ACAGCAAAGAAAGAGAGAGAGAGAGAAAGAGAGAAAGAAAAGAAACAGTTAAAATGGA
ATTTAAAGATTGGACGAGCACAGCAGACGATATTTTTATTCAAAATAGAGAGGATGTGATA
AAGTGATCGTGAGAGAGAGAGAGAGAGAGAGAGAGAATGATCATTTTCCAGGAAAAAGAAA
GAGGAAGGATAAGGGTAACTTGCTCTTTGGAAAAATCTGACCTGTTTCGTTTTAAATAGAG
GAGAAATGGGAATGGGGGCGTACGAAAATCGGACGAGGTATAATTTGACTGGTTATTTATT
GGGGTGCATGGATCGATATTAGACCGGGAGACACGAGAGGGCAACGCAAGACCGGAAAT
CGTGGTTTGCGGGCGACGATACATCAACGAAACAACGACCCGCTGTGACGGAAGTAAGCT
GGATCGAACGTGTGTCCCTCGTATGGAGAGAGAGAGAGAAAAAGAAAAAAAAATATGGA
AAATTCGAATCGTAGATCAAGACGCGTTGAAGTTCCTTCGGAAAAGAAATTCTCTTCCTAA
TTTTACTTTCGAGTTTATTTTGACTATGAAAGAATCTTTAAAAGATTTTATATTGATTCTTTTC
CGATTCTTCTTTCCCTCTCAAAATAAAAATTTTTCGAAAAGAAGAAGCCTTTTTTCTTCAAA
GGGAATTCGGAGGCTCTTCCTAATTTTGTTTTCGATTATTTTTCGATTCTTCTTTCTCTCTCAA
AATAAAAATTTTTCGAAAAGAAGGAGCCTTCAAAGGGAATTCGGAGGAAGGAGTCGGGAA
TCGATAAAGGGATTGCAATGAGTCACGTCCGGACACGGCGCCGCGATCGCAATTTATCGTT
GGAGGGGAGGCAAGCTCACCACGGTCACGCAATTTTTGAATATTTACTTTAAAATTGCGAC
GATAAAAGACGGGGACTGGAGGTGTACGGTGGGCACTATCCGTTCCAAGGATCGATATAT
CCTCTGATAGGGAGGGAAAGGGAAGGCAAGAGGGAAATGTGAGCGGAAATCGCGTTATC
GATGACACGGAAAAAGTAAGCAAACGGAGATAAAGGGGCATCAAAGTTGCACGAGAGAC
CAAAACCCGGTACTTCTCGAGGAAATGGGGGACGATGTCGCTTCGATGACGCGTATTACAT
CGGATATCGGCCAGCTGGATATATTTTCTAAGGGAAAATGGGCGAAACCCTCGTTCCATAT
ATCCTTTCCTCTCCCGATTTATTATTTCGCTCGGGGAGTTGAATCACAGTATGAATATTCTTTT
CTTCTTTTCTTTTTGCCATGCTCGATCCATAAAAAGATAAACATTTTCAAACCGACGTTCGA

GCTAAGAGGAGAATAATTAGACGATTCTCGTTCAACGATCGAGCGTTGCAAAATGACGAG
GAGAACGAAGGAGGATAAAATTCGTGGCCGGATCGGTGGAAAAGGAATGGCGGACGGAG
AGGAACGCATCAGGAACGGAACCAGTTTCTTCACACTTTGTTGCTCGAAGGAACGACTCCT
TCGCGGATACGCACACGCATACACGCGTGCATACTTCCTACGATTCGCGTCGCGTTCCGGA
ATGCGTTGAAAATGCATCGAAGGAACGATTTCGAGCGCGCGTTTTCCGCTCTCTTCCTCTCC
TCCAACTTCGAGAATCCCTCCTCCTCCTCCTCGATCCATCTCTCCGATTCTCTCTCCTCGAGA
TCTCTCTCTCGAGAATAAAATATTCGGGGGACAAGATACATATTCTCCTCTCCCCCTCCTCG
TCACAAGCAACCTCCTTAAACAGTCGCAACTTTCCTCTCCACGGCTCGCGCGTCCCACTTGC
GGCGCCGCCTTAACCCAGTTGCGGCCAACTTCCGGTGGCACCGATCGCCACCTTAATTGCC
ACCGCCTCTAATTACCGTGTCCTCCCGGTCCTCCGCTGACTTGGAAGGGCGTCGCGACGAG
AGCACGCGATCCCCCGTATATTGTGCGTGAATGTGTACGAGATTACGAGTGTGAGAGACGA
GACGAGAGAGAGTGAGTAATGCAAGATGAAATTTGTATTGTACACACGACTGTAACACGA
GTGTCGATGCTAGTCGAGCCGAGAATTATTAATTTCGGGGAACCAAGAGAGTGTGTGACGG
TAAACGAGTACGTGTAGCGTACGCCAAAAAAATATATATATATATATATATAAAGCGTTTA
TACTTGTAAGAACATTTGTAAATAGGCAATTGCAGGCTGTAAGTTTTTCGTAGGATTTCCTC
GGCGGCGTGGAAACCTGTTGGCTCGGTTCGGGCAAATTTCTTTTCTAAACGATTTAAGGATT
CGTGTTGTTCATTTATCGAGGTTGAACGTTCTTTCGATAGTTTATTCCGGTGTTGTTGTTCCGT
TGAGAGAGAGAGAGAGAGAGAATGATTAAATTTAAGATTAAGAGATTAAGTAGGAAATTT
CGTGAATTTTGATTTTCGTGATACGTACGAATTTTGATTGAACGGAAGGAAGGATCCGGATA
GTAAAAAATTTGAATAACTTAGATAGGTAATTGTTTAATTAAAGGAGAATTTGGTTCCGTTC
GAACGTTGTCGGATGGATTAGGATGATTAATCATCGCGCGACTGATGATTAAGAGCGACTG
ATCGACATATCAGTGTAATAATAATAATAATAATAATCATTGTACGATTTATATATAATTTG
AAGGAATTTAAATTTACCCGATCCGAGTGCCGAAGTGTTTTCCACACGTTAGAAAAAAGAA
AAAGAAAAAGAAAAAGAAAAAAAAAAGAAAGGAAATCGACCAATACGTCTGTCGAGTAT
GATTGTTGTTTTTATATCGACTGTCGTTCAAAAAAATTCTATCGTAACACTATACTTGCCCT
TGTTGTGTTTTATTACCATTCGATATTTTATCTCGTGCTTCTCGATCGAACGATAGATCGCAT
ATTTGTGTAACAATTAAACACGGAGGAGTAGCGACGAACGATTAAGAAAAGGAACGAGA
GGAGGAATTTGCGAGAGAGAATGATCGAGTAATTGCGAGTAACTGTGAAAGGAAGTAAGA
AAGAAAGACGGAGAGAACGTGGAAAGAGTGTACGTAAAGAAAGAAAAAAAAGAGAGAA
AAAAAACCGTGAAAACGAGAACAATTATTATTATTATTATATCGTCGTTCGTCCTCGCGCTT
CAATATTCGTATATATTTATATAGCATAGACTCGCCACTCTTCTCTTGTGTGCGTGCCGTAGA
TGCGCCAGGTGAATGTTGATTGTATTACAAGCCCGGGGACAACACCGGCTCCAGTCCCAAC
CAGACTGATGAACGAATAATCCTTTCCGATGATGACTGTGAGCGAGGATTTTTTCCAAATA
AATCAGCTAATTCCGTT

364. >MSTRG.7932.1 Gene=MSTRG.7932
GAAAGAAAGAAAAATAGAGAGAGAGGAACATTGGTTGGTGTAATCCAAAGCGGGCCACG
CGCGTTCGAACGCGAGGATGAGTGGCGCGCGAGACAAAAGGCCCTCTCCGCTCGAGAGGA
GAGTTCACGGACAGCTGAATGGTGAATAATATAGCGCAGGAGGACCAAGAGAGAGGACC
CAATGCCCTCTTTCTCGCAAGCCTTGACCACCTCAGGCGGTAGAGTGTCCAGCTTCAAATTC
AAGTCGTCGTGTATCTTCTCGATCCACTTCATCATCGAGAAGCTGTCCAGATTCGCTTCGTTT
GGCCCGACACAACTCTCGTACGCATCCTCGACGAACACCTGAAGACGGAGCACTTCCTCCG
AGCAAACGGAATCCCGGAACACGGTTTCCAAAAGCTCGTTCGCGTGCTTCTCCAAATCTAC

GGCTCTGTTCTCCAACACGTGTATGCTTTCCTAGAAAGTTTGGGTTTTTCGCGCGCATTTTGT
ATCGTGGTCATCATTTGAGCCATCGGCTCCGCCAGACTCTCCAAGTGGATCAACGCGTTCA
AATTTTGCATCTCGATCGATCTGAACACGTTGATCAGTTCGTACGGTTCCTTAAAATACAAA
TCTGCAGGACCTGCATCGCTGATATCTTGCTCGAAAAAATCTGCGAGAAAAACAAGTCGAA
TTATTCGTTTGAAACGGAGGGAGGGGAGATATTTGGTGCGTAATCACCTATAAGAGCGTCG
AGGGAGGCAACTTCGCCCAGCATTCTGTAACGGCCGAACAAATCCTCCGTATGGTCGTAAG
TGATGCTCTCCCCTGAATGACTGCGATGGATCCAATCGTGCTCCGTCCGCCAAGAAATCGG
AGCTATCTGATAGAGGAATCGTTGGCACATCTTCATCATTCTCCAACTCTCCTCCCAGTGGT
ATACTTCCAATCTGACCCGGCCATACTCGACGGAGAGCTTGTTCCTGTGATCCGTCATCTCT
CTAGTTTTCTTCGACTCGTTCTCAGCGAGTAATAGAAGTTCCATACTCTCCTCGTGATCCTTC
GAGAGAAATTCCTCGAAACCGCTTATGTATCGACGATATCGTCGCTGGAAAAAAAAAAAC
ACGGATGGATGGAAGAATAAGCGATTATATAAGATATTGGATCGTGGAAAATTTTTCTTTT
TTTTTTTTTTTGATTTAAAACAAACACCTTGA

365. >MSTRG.7999.4 Gene=MSTRG.7999
CTCAATAAAGGGCGCTAGTGTCCGTCAAGTGTCACAGCCGTTGCCCGTCTCCTCAGTATTGT
AACTTAAATCGGTGATTTAATTAGGAATAACGTATTTTTTTCTAGAATTTGTGAAACATTAA
GCAATAATGACCGACAAGAAAGCGGACCATTACAAAGACGATTTGATCCCAGTCAGTAGG
TGGAGCTTCGGAGACCGGCGTTCGCGCAGCCTAAGCCACCATACCCGGTGACACCTCATCC
ACCAACAGGTTTTGTCCCTGCGCCGGCACAGCCTGCTACTTACGGTAGTACACTTGCAGGTG
CCGCACCGTATGGAGTATTTCCTAATCAACCACAACTATATGCCGCGCAAGGCCCACCACC
GCCAACGTATGATCAAACTCTTACCCATCCAATGATGTATCAGCCAATGTATGGTCAAGGA
TATCCAGGATACTTAGCGGGTTATCCTGCATATGGTCCATTGCAATATTATCCACCATTAGC
TGCAGCTGCTGCATATTATCCCGCAGCGGCAATGCAACCTCCAGTTAGGCCTCCTACACTCG
TAATGCCTAATGGGTTCGATGCTACTAATCGATTCGATAGTATTTCACAACCTGTCTTGCCA
CCACCCCCAACAGTCCCAAATGCCGCCCAATTAGCTGCAATGGCAAGTCATAGCGTGGCTA
TATCGCAAAAGAAGAACTTTCTAGGAGGAGGAACAGAAGGCGGTTACACCTTTTGAATTA
ACGAGTGAATACGTGCACCAGTGGATGCGAGTTGTCTCATTCGCCTACAGGGATCGATATT
CAAAGTGTGTTCTAAGCCTTTTTTAAGCGCGACTTGGTTTTCTGCAAGACAGACCGAAGAAC
AAGGGGGCTACTCGTGTTATTTTTGTTACTTTCTACATGACTATATATATTTTTTTCGATATTT
TTTTCCTTTGGAGTCTTTAATGCTCCAACATTTAAAAAAATCATCTTGAGTAGCCCTAACGTT
TAGAAAAAGAAAAAGGAAAAAAAAAAAAAAATATGTACGTGATAGGAGCCCGAGGAATGC
GTGAGAGTCATTCATAGTTATGAGGCTAATTGTTGCGCTTTGTACTTCGAATTTAGCAAACA
ACTAGTAGTAGTAAAACTTTTATTATTCTTTTTTGATAAAAATTTTAGTGAAGGTAGCGAGTT
GGTAAAAAAAAACCTTGAAAAGGTTTTAACATCGTTAGAATCAACATAATTTTGACGTGTTTT
GCGATTAAAAGTATAAGTATGATGATATTTGAGGCTCGAGTGCCAAACGACAAAATTAAA
ATTATAAAAATATATCGAAGATTCTCACGTATTCAACGACTTCGAAGATACTTACCTATTCT
ATCTAAAAGTACGCACTCTCTTCTTTAAAATCAATAGTCTTTCTTCCTAGTTGTTTGATGTTTC
TTTTTTAAACACAGATTAGCTAGATCGGCTAACAAAGTTATTATTTAATTGAAAAAAGGCTT
GTCCACGGCGTTCGAATTTCAGTTTAAGCGGGTTAAACTGTGCTTTAATTGAAATAGTTACC
ATATAGTGAAATTTGTTGATAAATAATCTTTTTCTTCTGTCTCTTAAATAAAAAAAATACCTA
TAATATTCAAAATGATCAGTGTGAAATTTGAAAAGAATACGTGAGTATACATCCTTTCTAAT
TGCAATAATAATTTCAAATTTTGGTTCAAATTCCATAGTAATTATGTGGAAACTAGATTCAA

TCAAAGCTCATGAAGGAAACAAATAGTTGACTCAATTTCAAAGTACAAATTGAATATCCAT
GTAAGTAGTGGGAAAATTCGTAACGACGGGGTCAAGCTTTATGTCAGCCATATTAGGCGAG
TTGCGATGATGATAACGGTAAGCAACGCAGCGAATTCGTAATGCCTATAATTAATATTCAG
TGCAGAATTATTAACAACTATACTTTTTCTGAAAACGTAAGATCTATTTCAAGAAATGAAG
AGACATACGAAAAATTTCGAAGATCATTAAAAATTAGTTGCACAGCTACGAAAGCGACTG
TTTTGCTCCGCGATCATAGGCATATAGTACAGTGAACCTGCTGATAGGATACTTGGAATGTT
CCGTTCTGCAGGTGCACAATTAAACACGATATTAATTAAGCAATGAAGATCGAAGAAACTA
ATTTACAATTGGTGATAGCTTAGGATAGGACCAAGAATCGCAGTTCTATTTAGAAATTTTCG
AAATGGGTGTTCCGGCAAACTTGACCAGTACCCTTATATGTTTCGCATTGGGCTTTATCAGG
ATAGATGTTTAAAAATATCTATATTGTTAAAAAGTGATTTTTGCTTCTCCAATCAGAAGTTA
AAATTAATTGACGATAAATCAAATCATGATTTCTATTTTGCCCTGATGAATTATTATTTATAT
AAATCAAGTTTATTGCGTGAAAGTTGTAAATTTTACTTGAAAATGTTATCCAATATTAAATT
TTGCAGTTAAAACGCAGGAACTTCCTATAAAATTACTACTGTTCTTTGTATTTTGTGATATCT
GATTACCTCAAAATCTAAAACAGAATATGTAGGTATTTGTCTTGTTTTGTACCATCCTCGAA
TTTAATTCAGTTTCTACGAAAAAAAAAAAAAAAAAAAAAA

366. >MSTRG.8013.1 Gene=MSTRG.8013
CCTGTTGCGAAAGTTTTTGCTCTCTGCGTCCGATTTGTTGTCGGGGGGAGGGCAGGAGGGCG
CGCCGCCACGATGGTGCGAGTTCGACGCGGGAGAGGAGCCGGAGACGACATTCGGATTGG
CATTCCCGTCCAACATATCCGGCTCCGTGCTGTTCTGATACGACGCGTTCGAGCCACTCGCG
ACGAAGCCTTGCCTGGAGGACGGCAGCGTGGGCATACCTCCTCGCACCACCACCGTGGGA
CTGTCAGGTCGGCTGTGCGACCTGCTGCCTTTATGCACCACTATACTTGGTATCTCCAGGCG
GGCCATGTGTTAAATGCGTCATGGGGTTACGGTGCAGACTCTGCAACTGGTGGGTTCAAAC
AGGTGCAACTGGGTTAGTACGTGAATCTTTCGGGTGGCGTGCAGCTCGTGGGGCTGATACA
AGGAGCCAAATCACTGCACGTCCACCTTCCAACATCGTACACTCTGGTTCTAAATATACGC
ACGACACAACTTCACCATTTCACCAATTTTTCACTGTAC

367. >MSTRG.8043.3 Gene=MSTRG.8043
TAACAGATGATTGAACGCATTCTGCGGCGCGGTCTAGCATTTATCTGTCAAAACTGGTGAC
GGCCGGCCGATCGGCGATGAGCCGGCATACGCGTGCCTGTTGATTGTTTCGTGCTAAAGTTT
TAGGTTATCTACGCGAATATTTTAATAAAATACGTGTGTGTGTATATTTGTGCACTTAACATT
ACGTTTTCCGAGTGGCATAAACGCGTCGATCATTTTCCCGCAGGAAATGTCACAAATCAGA
CGAAACAATGCGATACCCAAAAGATGGAGAATCAATGCGATGAAAACGAGAGATGCTTTC
AAAATTCGAAAGGGGAAAAAGCATATTGCAAATGCAAGATAGGTTACGATCTGGAGAAAG
GCCGATGCGTCCAGGTTACGATTACCACGGTTGCCAGTGTAACTGATCAACCAAACGTCAC
GGCCAATTCAGCAGTTCTGTAGCCGCTGGCTTGTTAATACCAACTTTTCTCGTAGTAATGAG
CGTATTACTGTATTTTGTCGCGAGGCGATATAAATGGTTGCAACGAGTTCGACAGCTTCGTC
CAAACCATTATGGTAATGTCCTAGTCACAAGAGACGACGATGACGATGACGATCCACCTAT
AGCGTAAGCAGAC

368. >MSTRG.8043.2 Gene=MSTRG.8043
CGATGTCAATTGAGTTCGATAATTGCAAATGGTCGTTTAATAACAGATGATTGAACGCATTC
TGCGGCGCGGTCTAGCATTTATCTGTCAAAACTGGTGACGGCCGGCCGATCGGCGATGAGC

CGGCATACGCGTGCCTGTTGATTGTTTCGTGCTAAAGTTTTAGGTTATCTACGCGAATATTTT
AATAAAATACGTGTGTGTGTATATTTGTGCACTTAACATTACGTTTTCCGAGTGGCATAAAC
GCGTCGATCGTAAATATTCTTTAAATAATTGCGTCCATTATTATGTGATCGTGAATTGGGAA
CGAAGTACACGTAGAGAGAAAGACAAAGAGAGAGCGACAACACGAGAAGGAAAAAACA
AAGGAAAATGCAAAACATTCGAATCTATGTTTTCTGTTATCTCATCGTGGCAATACTTTTTCT
AACGCCATTCAATTACGCTTCAGATTTTCCCGCAGGAAATGTCACAAATCAGACGAAACAA
TGCGATACCCAAAAGATGGAGAATCAATGCGATGAAAACGAGAGATGCTTTCAAAATTCG
AAAGGGGAAAAAGCATATTGCAAATGCAAGATAGGTTACGATCTGGAGAAAGGCCGATGC
GTCCAGGTTACGATTACCACGGTTGCCAGTGTAACTGATCAACCAAACGTCACGGCCAATT
CAGCAGTTCTGTAGCCGCTGGCTTGTTAATACCAACTTTTCTCGTAGTAATGAGCGTATTACT
GTATTTTGTCGCGAGGCGATATAAATGGTTGCAACGAGTTCGACAGCTTCGTCCAAACCATT
ATGGTAATGTCCTAGTCACAAGAGACGACGATGACGATGACGATCCACCTATAGCGTAAG
CAGAC

369. >MSTRG.8086.1 Gene=MSTRG.8086
GCGCACGTGAGCAGCGCGGATTTTTTTTTTTCGATCGTAAATAAAACTTCCGTGCATGTACA
GCAGATATTTAATTACCGTCACTGCGTTGAATAAAATTAAAAGTATGGAATGAAAGATCTG
AGCAAGTATGGACAATATCGGATCTCTCTCTCTCTCTCTCTTTCTCTTTCTCTCTCCCTCTC
TCTCTCTCTCTCT

370. >MSTRG.8090.1 Gene=MSTRG.8090
ACCGACAATAATGTGCCATCTAGCGTAAAAAATAACACAAACATTTTTCATGGAAACACTA
CAGTTTGCTATTGAGATAAGCTTATATTTTTCCAATTATCATTCACCATATGCATGGCAAGTT
AAAATATTATAGAAAATTTTAAATAATTCACAAAAGAAAACGCGATGCCTACAACAAAAG
AGGAAGTTAACCAACTTATTGCATCTCATAGCATTGTGATATTTTCTAAAACGAGTTGTCCA
TTTTGCAAAATGGCTAAACAAGTATTTCATAATTTGCAAAAAGAATATACTGCTATCGAACT
GAATGAAAGGAATGATGGAGATGAAATTCAAAGTATATTAGGAGAAATGACTGGAGCTAG
AACAGTACCTAGAGTATTTGTAAATGGAGTATGCTTAGGAGGTGGAACAGATGTGAAGAA
ACTTTATGAAACAGGAGAATTGCAAAAAATGTTTTAACAGATTACTTCTGTTGCTTATATTC
TTACTACTC

371. >MSTRG.8086.2 Gene=MSTRG.8086
TGTGTTCGATGACCGTTTCTGTGACGATGACGCGCACGTGAGCAGCGCGGATTTTTTTTTTC
GATCGTAAATAAAACTTCCGTGCATGTACAGCAGATATTTAATTACCGTCACTGCGTTGAAT
AAAATTAAAAGTATGGAATGAAAGATCTGAGCAAGTATGGACAATATCGGATCTCTCTCTC
TCTCTCTCTCTCTCTCTCTCTCTCTCTCTCTCTCTCTCTCTCTCTCTCTCTGTGTGTGT

372. >MSTRG.8086.3 Gene=MSTRG.8086
ACGATGACGCGCACGTGAGCAGCGCGGATTTTTTTTTTCGATCGTAAATAAAACTTCCGTG
CATGTACAGCAGATATTTAATTACCGTCACTGCGTTGAATAAAATTAAAAGTATGGAATGA
AAGATCTGAGCAAGTATGGACAATATCGGATCTCTCTCTCTCTCTCTCTCTCTCTCTCTCTCT
CTCTCTCTCTCTCTCTCTCTCTCTCTCTGTGTGT

373. >MSTRG.8086.4 Gene=MSTRG.8086
GCGTTGTGTTCGATGACCGTTTCTGTGACGATGACGCGCACGTGAGCAGCGCGGATTTTTTT
TTTTCGATCGTAAATAAAACTTCCGTGCATGTACAGCAGATATTTAATTACCGTCACTGCGT
TGAATAAAATTAAAAGTATGGAATGAAAGATCTGAGCAAGTATGGACAATATCGGATCTCT
CTCTCTCTCTCTCTCTCTCTCTCTCTCTCTCTCTCTCTCAACATAATTAATCGTGCGTTTTGCA
ACGTGTCGCAATCACAAAATCGGAGCATCGTCCGAACGTTCATTTGCAAATGTCTTTTATTC
TTTTTTCCTTTTCTTTTTCCTATTTTCTCATATCATTTTATTATATATAATATAAGAAACAATAA
AAGTTTGTTGCGTTTAATTTACATTATGTGCGATATTTTTGTCGCCTCGGATAATTCCCAATTT
ATTATTCAGGCAAGAACGGAATTTCGTGTAAAATGCGTTGCAACTAGCGGAACGGTTGTTC
GATATAATGCGATGATAACGGTTGCCGTTTAAGATATTAAGGAACGTCGAAGATAATTAAA
CAAAATGTGCGTGCGTGTGTCACCGATATCGTGTAATATCCTTCCTAATCATAATGCTTTTTC
GATACGAAATAATAGATATATCGTATTTATGTAACTTTTCTTTCAAGAAACGAGAAAAGTG
CGTGCGGGTTCGACTATTCGAATCGTTTCCAGATTATCACACAAGTCTTAAAGATACGATTT
CAGAGACCCAATAGACTCGTAGGATGCGTCGATTCTGTATGGGAATCCAAATCTCGGTCGT
GCGAACATTATTATCATTAATCTGTAGTGACATAATATCGTTTTATTAACATATTTAGAATTA
ATACGTGAATAATTACCGAAGATTGATACGGCAATCGCAACAGCGCTCTGTTTAGCTTATA
AATACGTACGTACGTACATACGTACACACACACACACACACACACACACACACACACACA
CACACACACACACACACACACACACACACACGCATACGTACCCGCGCGTGCGTATATATA
CATTTTATATATATATGTATATATATGTATATGTATGTATATATATATATATATATATATATAT
ATGTATGTATGTATGTATCTATGACCACCGTTTTTCGTTTCGAGTAGCGTATACACGTAACG
ACGTGTACAATATTTCTCTCCCATCGTTTTCCCCTATTCCTTTTTCTTTGATGATACGACCTG
TTTTCAATGGTAATTAGGTTAATGATAATATCATACTAATAATGGTTTAAAAAAAGGAATGT
AATAACAATGTATTTGTGAAGATTCACAAAAAAGACGTTTCGTGTTCTTTAATCATTATTTG
CACTGTTTTTCGTCTCACTCAAGTTAATTGCCTCCTATTGAATCCCTTCTCCCATTCGACCATT
ACCCTGTATAATATCATCCAATCTATATTACCTTAACGATATTTTTTACCTTTTAAAGAAATT
GTTCGCCCCCGTAAAGGTTGGGTAAACGGATGTGCGCTCTATCTGGCTTCCTGCGTATCGCG
CGTTACAAACGTGTCGTGATCATTAAACCCACGATATTGACAATCCCTGGATATATTCGTAT
ATATACGTGTCGACTTCGTTTTCATGTCGCACTCTTTAAAAGAAAAAAAAAAGCGTATTATT
TTATATCCGCGCAATTACGTTTCTTGTATTATCTATTTTCACATTATAAATGCGGAAAAAAAT
TTATTTTTTATTTTGTTTTTTTTATTATATTTGTGCAAAAATCTAATACGCAATTAATATAAGG
ACAAATATTAATCTCAGTAATTACTCGATACTTATATTCATTACTCTATTATCGTATAAAGTA
ATGTTTCTCGTTTAAAAACATCATTATTATTAAAAAAAATATTTAAAGAATAATATAAATAA
ACTTATCCAGAAT

374. >MSTRG.8100.1 Gene=MSTRG.8100
CGTCTATTCTCTGCGTCTTCTCCGTGATATTGAATCTGCTCTATGCTCGTCCGATCCGTAGAG
GAATACGTTGATTAACTTTATTTTATACGTACGTGTCGTACACGCGTCTGTCGTCCGTATTAT
TTAACGCGAGTGAATAAAATAATTTTTATCGGTGATAAATGCGACGATCGATGTAGATTGC
GTCGCTTGTGATGTCTGTTTAAAAAGAACGCCATTGATTTCATCGTAACGTTATTTGTACTTG
CGTACTTATCGTGAACGTGTTTTATTTATAAAAATTGAAATTATTTTATTTTGAAATTATGGA
TTTATTACCAGAACATAATTCGACGTTTTGGAAAATTGGTAGATTAAAAACGTTTGAAGATT
GGCCGTTTCAATCTTCTGACAATTCGTGCAATCCCGAGCGGATGGCTGCTGCTGGTTTTTAT
GTAGTGGGTGGAATGAACATATGAAACACGAATCGCAATGTTCCTTCATTAGATTGGGAAA

ATCGGATGAGACTACTTGGACTGTGAACGAATTGTTCGATTTGTTTAAAAGATATATGGTTA
AGGAATGCATACACGAATTGGACAAAGCTGTGATTAAGGCGAAAGAGGAAAGTGCACGAT
TGGTACGTGAAATACCTCATATTTATAAGAAAGTAAAGAAAGGAAAGAGAGTAAAAAATT
AACGATATGCTCTAAAAAAATAAGAATTAAAATAGCGTTATTTATCCGATGATTAACGATG
TGATCTTTTCTTTTTTTTAATAAAATTTTATATTTTAAAATAAATATGAATAGGGTTTTCTTTC
GA

375. >MSTRG.8107.6 Gene=MSTRG.8107
AGGCGTTCGCCGGGCAGAGTGGAGGTGGTTACGTTGTCCGCAAGGATATGTGGTCGGTTAC
CGCGACGCCGCCGCGGTTCGCGCAGGCACAGGTACGAAGTGCGCGCGTTTTCGAGGCGGT
ATCACGAAAAGCGCAGGAGTGGCGTTTCTACGTATCGTTCCGATATCACTGCTACAGTTCAT
CGTTGATGTACAGTGGGTAGTAGGAAGTTCGTAAATCGGCAAAAAAAGAGGAAAGACGAG
AGGAAAAGAAGAAGAAAGAAAAGAGATAGAGCGCGGTTCTATCGCGCGGTGCAGTGTGA
AAAGTGTTCGCGAAGGAGGCTTTTGCTTCCTCTTGTGTGTGCGTGTACGTGCGTGTGCCACA
GCAAAAGCACGTAAGAACGTAACTAGTGACACACGGAAGAAGGAGAGAGAGAGAGAGA
AAGACGAATAAACACGGGTCGTCGGATAATCCTTGTGTACGGTGTGCGGGTTTCGTGAATG
TGCGTCAAATGACAGTGAGGTGATCAATGTGCTCGAAACATGGCTGAGGAGCTGGTGGTCA
CCCTGAACCGCGGCGACTCATCCGGATTTGGGTTTTCGCTCCTTGGTACCGCGGGTTTGCCG
CACGTCATCTACGACATCGTTGAAAATTCGCCGGCAGCAAAGAGCGGCAAGATTGGAGGT
GTTACATAATCAAGAGGCTGGTCTGTGGTCATTGGCCGCTAACCCATGGAAGATCCGCAAT
TCTTCAAAATAAATATGGAAGAAACCTCAGAGGATATGAGTATTTTATAAAATTCAAAGAA
AAATTAAAACGAAATTATTCGAATTATTGAATAATCCAATCTAAAAAAATATTGAACTATC
AAACTATTAGAATGTTTACTCTCGAATTTACAGAATTTTTAGAATCTCTTAAATTCACACATT
CATTCCATAACGAAC

376. >MSTRG.8116.7 Gene=MSTRG.8116
TTTTTTATATTAAACTTTACTAGAAATATTTTAATATTGACTAGTCTTAGATATATTTTAGAAT
ATGTAAATAAATTATTTATTAGATTTATAAAATTATTATTATAATAGATATAAACTTCTACA
AATACAATAAATATTCTTTAAATTGTAATATATATTCTTTAAATTATTATTATTATAACAGAT
ATAAAGTTCTATAAACACAATAAATATTCTTTAAATTATAATATATAAAATATATTCTTTCG
TATATACAATAATTCAGTTTATGATGATAGATCCAAACTAAATTTTTCTTTATTTATAAAGA
AGTTTTATTTATTAAGAAATCTTTATTTATAAAGTTTTACATATTTTTATATGTATATAGAGA
ATTAAGTTTTGAGATATAAAAAATCGGTCAAGATTTCAATATGCTCATATTGCCAAAAGCA
AATTGACTTCGGCAATAATCGGATACAATTAGTTTGTACAATTATTAAAAGTGATAGCTTGA
TAGTATCCGATTCCTTGAAATTGTATTGAAACTTTTAGAATTCTATCTTACTCAAATATTTCA
AAATTTATCTCGAATGTTCATTTTCTAATAACATTATCCATTAATTTGTTATTACATATATAG
AATTTTTTATTCAAACTTTTTCAAATCTTTTCTTTCGTTATTATTCGTTCACCAAAAACTTCTA
CGAATTAATTAATACAAGAGCAAATTTCAAAAGAAATCAGATCATACTTTCGGAAAATATT
TTATATATACAAAAATATTAAATTTTTTTTTTTATGTAATTAATTATTGAGGAAAATTTAGTTT
TTTGATCATCCTTTGTATATAAATCATAAATAATCACGAAAGTATAAAAATAAATTCATATC
AAGAACATAAGTTTATTGAATATAAATCCGTTAAAATAAAGAATATAAAACAGTTTAAACC
ATCTGACAATAACAAACCATCGAAAAAATTAAAGAAAAAAGGAATCGTCGAAAGCAAGA
GATCAGGAGGAAATACGTACATACACATGCGCTTAAGTACATGCATACATACATATCAAG

AGCAAATCGAGTTCTCCAGATGTTCTCGAGACGAGAACATTTTCGCCGTCTGTCTCGAGAA
AACAAGAAAGAGACGGAGCGAACGAATAATTTAAGCGGAGAAAGAGAGAGAAAGAGAG
AAAGAGAGAAAGAGAGAGAGAGAGAGAGAAAGACGCGTGCAAAAGTGAAACGGAAG
CAGAAAGAGAAAGAGGATACTGGAAGGAAGGAGAATATGCACCGTTTGCCAGGTTGCGAT
TGCAGGGAATGGTGGGGGCGGAAAGAGCCGAGTTAAAGAGGGGAGAGGTCTTCCAACTTG
GACATCCTGAAGTATTATCTATCTCGGAAGTTCGTCCCCGAGGCTGCGGCTGACTCCGTTGA
CGATGACGAAGACGACGAGAACGGTCACGCTAATCGCAGCTGGCTGGAGATTGGCTTTTA
GCACAGAGTTCGACCAACTTGTATCGCCAGGAAATCGCGGGCGCACCCCTCCGCTCAAATT
TACAGGGCGATTCCCTCGACCGCCCCCTTCGTCGCGAGGGATGGTATTTGCGGCCACGGGG
GAAGCATTGATGGAAGGGGTTACGATCAGGTCGACGTGGTCTCTAGCGGTGATTTAAATTG
CTCTAGGCGAGGGCCTCTGTGCACTTTAGTTTCTGGCTTCTTTTTAGAGATTGTTCTTTAGAA
GTTTGGTGGTTGATGAGGTCCTTGATGGTATACAGGTTGTAAGTGGTTTTCTTCATTGTTTTA
GCTTTATACATTTTTCTTTCAGGGATGACTCGAGATTGGATTATTAGATTGAATTTGAAATCA
TGTGAGATCTTAACATTTTTTTTAAATTCAAAAATATTTATTTTTTCTTCTTTCTTTTTTAAGA
AAAAAGATTTTCTTCTTATGTTTATTAATAATAATAATCTTAAAAAAATTTGATTGATCTTTG
AATTCGCGTTTTTATGATATTATAATAGTAATAATAGTAATAATTTGTTCTTTTGGATTATGA
AAAATTAAGTATTTATTTTAAATGTGATATTATATTAAAATAATATGAGACTAA

377. >MSTRG.8116.8 Gene=MSTRG.8116

AATAAAATATAATAAATATATATATACAAATAGATATATATATATATATAATTATAATGTTA
TAAATACTTAAAAGTCTTACTTAAATATTCTATTTATTAGAAAAATATAGTATTTTTTTATAT
TTCATCATAGTATCTTATTCCAATTTCCAGTCAATTGTATTTTTAGATTAATTTTTTTATATTA
AACTTTACTAGAAATATTTTAATATTGACTAGTCTTAGATATATTTTAGAATATGTAAATAA
ATTATTTATTAGATTTATAAAATTATTATTATAATAGATATAAACTTCTACAAATATTAAAAT
AAAGAATATAAAACAGTTTAAACCATCTGACAATAACAAACCATCGAAAAAATTAAAGAA
AAAAGGAATCGTCGAAAGCAAGAGATCAGGAGGAAATACGTACATACACATGCGCTTAA
GTACATGCATACATACATATCAAGAGCAAATCGAGTTCTCCAGATGTTCTCGAGACGAGAA
CATTTTCGCCGTCTGTCTCGAGAAAACAAGAAAGAGACGGAGCGAACGAATAATTTAAGC
GGAGAAAGAGAGAGAAAGAGAGAAAGAGAGAAAGAGAGAGAGAGAGAGAGAGAAAGA
CGCGTGCAAAAGTGAAACGGAAGCAGAAAGAGAAAGAGGATACTGGAAGGAAGGAGAA
TATGCACCGTTTGCCAGGTACGTTCTCGATGGACTTTTCTCGTAGTCACGAAAATAAATTGA
ATCACGCAGAAAATAGAAGTACAAACACATCCACGCCTACCGATGGCCGTAATTCCAAGC
TACTTTCCCAGGTTGCGATTGCAGGGAATGGTGGGGGCGGAAAGAGCCGAGTTAAAGAGG
GGAGAGGTCTTCCAACTTGGACATCCTGAAGTATTATCTATCTCGGAAGTTCGTCCCCGAGG
CTGCGGCTGACTCCGTTGACGATGACGAAGACGACGAGAACGGTCACGCTAATCGCAGCT
GGCTGGAGATTGGCTTTTAGCACAGAGTTCGACCAACTTGTATCGCCAGGAAATCGCGGGC
GCACCCCTCCGCTCAAATTTACAGGGCGATTCCCTCGACCGCCCCCTTCGTCGCGAGGGAT
GGTATTTGCGGCCACGGGGGAAGCATTGATGGAAGGGGTTACGATCAGGTCGACGTGGTCT
CTAGCGGTGATTTAAATTGCTCTAGGCGAGGGCCTCTGTGCACTTTAGTTTCTGGCTTCTTTT
TAGAGATTGTTCTTTAGAAGTTTGGTGGTTGATGAGGTCCTTGATGGTATACAGGTTGTAAG
TGGTTTTCTTCATTGTTTTAGCTTTATACATTTTTCTTTCAGGGATGACTCGAGATTGGATTAT
TAGATTGAATTTGAAATCATGTGAGATC

378. >MSTRG.8118.1 Gene=MSTRG.8118
TCCAATCTCGAGTCATCCCTGAAAGAAAAATGTATAAAGCTAAAACAATGAAGAAAACCA
CTTACAACCTGTATACCATCAAGGACCTCATCAACCACCAAACTTCTAAAGAACAATCTCT
AAAAAGAAGCCAGAAACTAAAGTGCACAGAGGCCCTCGCCTAGAGCAATTTAAATCACCG
CTAGAGACCACGTCGACCTGATCGTAACCCCTTCCATCAATGCTTCCCCCGTGGCCGCAAA
TACCATCCCTCGCGACGAAGGGGGCGGTCGAGGGAATCGCCCTGTAAATTTGAGCGGAGG
GGTGCGCCCGCGATTTCCTGGCGATACAAGTTGGTCGAACTCTGTGCTAAAAGCCAATCTC
CAGCCAGCTGCGATTAGCGTGACCGTTCTCGTCGTCTTCGTCATCGTCAACGGAGTCAGCCG
CAGCCTCGGGGACGAACTTCCGAGATAGATAATACTTCAGGATGTCCAAGTTGGAAGACCT
CTCCCCTCTTTAACTCGGCTCTTTCCGCCCCCACCATTCCCTGGCAAACGGTGCATATTCTCC
TTCCTTCCAGTATCCTCTTTCTCTTTCTGCTTCCGTTTCACTTTTGCACGCGTCTTTCTCTCTCT
CTCTCTCTCTCTTTCTCTCTTTCTCTCTTTCTCTCTCTTTCTCCGCTTAAATTATTCGTTCGCTCC
GTCTCTTTCTTGTTTTCTCGAGACAGACGGCGAAAATGTTCTCGTCTCGAGAACATCTGGAG
AACTCGATTTGCTCTTGATATGTATGTATGCATGTACTTAAGCGCATGTGTATGTACGTATTT
CCTCCTGATCTCTTGCTTTCGACGATTCCTTTTTTCTTTAATTTTTTCGATGGTTTGTTATTGTC
AGATGGTTTAAACTGTTTTATATTCTTTATTTTAACGGATTTATATTCAATAAACTTATGTTCT
TGATATGAATTTATTTTTATACTTTCGTGATTATTTATGATTTATATACAAAGG

379. >MSTRG.8118.2 Gene=MSTRG.8118
ATCTCGAGTCATCCCTGAAAGAAAAATGTATAAAGCTAAAACAATGAAGAAAACCACTTA
CAACCTGTATACCATCAAGGACCTCATCAACCACCAAACTTCTAAAGAACAATCTCTAAAA
AGAAGCCAGAAACTAAAGTGCACAGAGGCCCTCGCCTAGAGCAATTTAAATCACCGCTAG
AGACCACGTCGACCTGATCGTAACCCCTTCCATCAATGCTTCCCCCGTGGCCGCAAATACC
ATCCCTCGCGACGAAGGGGGCGGTCGAGGGAATCGCCCTGTAAATTTGAGCGGAGGGGTG
CGCCCGCGATTTCCTGGCGATACAAGTTGGTCGAACTCTGTGCTAAAAGCCAATCTCCAGC
CAGCTGCGATTAGCGTGACCGTTCTCGTCGTCTTCGTCATCGTCAACGGAGTCAGCCGCAGC
CTCGGGGACGAACTTCCGAGATAGATAATACTTCAGGATGTCCAAGTTGGAAGACCTCTCC
CCTCTTTAACTCGGCTCTTTCCGCCCCCACCATTCCCTGCAATCGCAACCTGGCAAACGGTG
CATATTCTCCTTCCTTCCAGTATCCTCTTTCTCTTTCTGCTTCCGTTTCACTTTTGCACGCGTCT
TTCTCTCTCTCTCTCTCTCTCTTTCTCTCTTTCTCTCTTTCTCTCTCTTTCTCCGCTTAAATTATT
CGTTCGCTCCGTCTCTTTCTTGTTTTCTCGAGACAGACGGCGAAAATGTTCTCGTCTCGAGAA
CATCTGGAGAACTCGATTTGCTCTTGATATGTATGTATGCATGTACTTAAGCGCATGTGTAT
GTACGTATTTCCTCCTGATCTCTTGCTTTCGACGATTCCTTTTTTCTTTAATTTTTTCGATGGTT
TGTTATTGTCAGATGGTTTAAACTGTTTTATATTCTTTATTTTAACGGATTTATATTCAATAAA
CTTATGTTCTTGATATGAATTTATTTTTATACTTTCGTGATTATTTATGATTTATATACAAAGG
ATGATCAAAAAACTAAATTTTCC

380. >MSTRG.8118.3 Gene=MSTRG.8118
TCTCGAGTCATCCCTGAAAGAAAAATGTATAAAGCTAAAACAATGAAGAAAACCACTTAC
AACCTGTATACCATCAAGGACCTCATCAACCACCAAACTTCTAAAGAACAATCTCTAAAAA
GAAGCCAGAAACTAAAGTGCACAGAGGCCCTCGCCTAGAGCAATTTAAATCACCGCTAGA
GACCACGTCGACCTGATCGTAACCCCTTCCATCAATGCTTCCCCCGTGGCCGCAAATACCAT
CCCTCGCGACGAAGGGGGCGGTCGAGGGAATCGCCCTGTAAATTTGAGCGGAGGGGTGCG

CCCGCGATTTCCTGGCGATACAAGTTGGTCGAACTCTGTGCTAAAAGCCAATCTCCAGCCA
GCTGCGATTAGCGTGACCGTTCTCGTCGTCTTCGTCATCGTCAACGGAGTCAGCCGCAGCCT
CGGGGACGAACTTCCGAGATAGATAATACTTCAGGATGTCCAAGTTGGAAGACCTCTCCCC
TCTTTAACTCGGCTCTTTCCGCCCCCACCATTCCCTGCAATCGCAACCTGGGAAAGTAGCTT
GGAATTACGGCCATCGGTAGGCGTGGATGTGTTTGTACTTCTATTTTCTGCGTGATTCAATTT
ATTTTCGTGACTACGAGAAAAGTCCATCGAGAACGTACCTGGCAAACGGTGCATATTCTCC
TTCCTTCCAGTATCCTCTTTCTCTTTCTGCTTCCGTTTCACTTTTGCACGCGTCTTTCTCTCTCT
CTCTCTCTCTCTTTCTCTCTTTCTCTCTTTCTCTCTCTTTCTCCGCTTAAATTATTCGTTCGCTCC
GTCTCTTTCTTGTTTTCTCGAGACAGACGGCGAAAATGTTCTCGTCTCGAGAACATCTGGAG
AACTCGATTTGCTCTTGATATGTATGTATGCATGTACTTAAGCGCATGTGTATGTACGTATTT
CCTCCTGATCTCTTGCTTTCGACGATTCCTTTTTCTTTAATTTTTCGATGGTTTGTTATTGTC
AGATGGTTTAAACTGTTTTATATTCTTATTTTAACGGATTTATATTCAATAAACTTATGTTCT
TGATATGAATTTATTTTTATACTTTCGTGATTATTTATGATTTATATACAAAGGATGATCAA
AAAACTAAA

381. >MSTRG.8116.9 Gene=MSTRG.8116
TTAAAACGTTGTCAGAAATGAGCGTTAATGTATATTGTCAGATACGCGGTATACATTCGCGT
GATCTTTAATGTTACTCCGTGTATTGCGTATCATTTCTCGCGCTGTATTTTCTACATATCGTGA
ACGTGTGTATGCACCACCGTGTTGTCGTTGTTTCTTATATGGGAATCACATTTATTACCGATC
ACGTATACACATATTTTCACTCGTATTACGTGCGTGTGTATAGACATGTTTGTGCAGTGTAAT
GTGCATGTGTACTAAAAGTCGCTTGCAATCCAGTCGAGAACGGAATCAAGGGAGCGCCTTT
CTGTGTGCTCATCGTGAACGGAAAACCATACGAGTATTATTGTGATCGCTCACGTATGATGC
GAGTTTCTAGAAGATTTGCGTGTATTTTGTGCGTGACAATATATGTTTTCTCTGACAAGTAAA
TTGTGGTTTTATTAGTATACTACGTCACATCGGTTTCTATGTGTGTCTATCTGTTACCGCAAA
CTGCGTATTGCATGTTTGATGGAATCATTGACCTCGCGAGCAGTTGACCGAGTTTTATGTTC
GTGGATTAAGAAAAACAATTATAGTTTTGTTTAAACTGAAAATTTCTTTTTTGGAAAACTTA
AAATTTTATGTTTTATTTTGGAATAGTTGTCTGTTTGAATTCCATTTGAATTCCATTTAAATCA
TTATAATAACATTCGTTGTATATATTTGAAATATCGCAACATCTAGATCCATTCTTTCAGCCA
TTTGGTATATGGCTCTCGGTAAGATCTACGCGTTCAAGGTCGCTGCACAAAACAAGTAGCC
GGCACGTAAAGTGGTCGCATGCACATGCACGCTGTACACGATACGCGATACTCGTTTCCTG
ACCAAAAATAATAGTTCGTCGAGCGCGCACAAGTCGAACCGGCCGCTGATCGAGGTCAGG
TTCTTTTGCATTCGTTTATTCGTTCATTCGCTTGTTCAGTTGCTCGCGTCTGCGATAAATTACC
AGCGACACGCGTAATCTCGACCCTTTCGACTCGCCTAGTGACTGTACTATAGTGTTCTTTCC
AATTGTACCGTTCGCGCTTACTTGCTCTCTTGCGCGCGCTGTTTTCTGTCTTTGTCGTGTATGT
ATTCGTGAAATACGCTTTCGGTTCGCTCGCACGCGACTCCGTGTATGGAAGTGTGGAGCAG
CGGTGCGTCGTATTTTCTTGTCTCGCATATATTTTATCGGTTGTTGATTTTTTTTATTTCACGT
GATTTGGTGTGATCGTAGTGAATGTTGTGAAACGAAGGTGAAGAAAGAAGAGAACCGACG
AAGATAGATAGAGAAAGAAAAGAAAGGGACAATAACGTTACAGGAATAAAGTGAAACA
ATATTGAGCGGGAAAAGCAAACATATAAAAAAGGGGAAAAAGCAAACTGGCGTGTGAGT
AGAAGGGCAAGTGGAACTTGGAGTAAGATAAAGACAGACGCGAACGAGGTAGATTGAAA
ATCGGTGAAACACGGCTACGAAAGAAAATTAATACTACGAAACAGTTGACGGGATATATC
TAGTAGAAAAAAAAAAGACAGTTCTTGATGCGTAACGCGGTTGGAGGAAAGGCCTGGCCA
TCGATTTGCCGTCCGTGAATCTAGTGCGCGTTTGAAATCGGGCTAGTCTCGCGCGCGCGTGT

GTGTTACGTGCACCTAAGCGAACGCAGCCAGCGAGGCGCGCGGACGGACGAGAAAAGGA
GGAAGAGGTGGCGGTGGTGGAAGAGGGGGCGGAGAAGGAGAGGCGGTTGAACAGGCGGG
CGGAGAGAAAAGTTCGAGAAGCGGCAACACCAGTGCCAGCCAACATCAACAACATCAAC
AACAACAACAATACAGATCGCTGCGGTGTATACAGGGCTGCTGCTCGTGGTGTGCGTTTTG
AACGTTTATCGCGTTGTGTGTCGCCGCGCGCGGCGGTGATCATCGGTGTGTGTGCCGGGAAT
TGCAGAGAAAGAGGTAGTGAACCGAAGCCGCCAGAATTACGCCAAACCGGGGAAATATG
AGCATTGCTGCCCTACTACAGGCCGCCGAGTATATTGAACGAAGGGAAAGAGAAGCTGAA
CATGGCTACGCTTCGACGATGCCGATGCCCGATGATATGCGGACGGTCACAAAAAGGCCA
AAGACGAAAAAATCCCAGGGCAGCAGGACAACTCATAACGAGCTGGAGAAAAATAGGTG
AGTGTACTTATTTCAATTAACCTACCATCATCGAGAAATTGATAAAGAATCTCTAAGGTACA
GTTACTTTAATCTGCCATTTCTCGATAATCATTTCGATATAACGATGAATATTAATCTCAGCA
CTCGGTCTAGATCGAATAAAGTTCGGAACGCTTGTTATCGAGTCGTTCTCGCGAATATTTCA
TTAATCAGAGTGATTTCGATTCCATTTATCACGGTGGAAAATTCGTTTCCAAACTTTTCGCTT
GAAAGACGCCATTTATGTTCGTCCTGGTTCAAAAGAGAATTTTAAGCAATCTTATCGCGTTA
GATTTATATATTTATATTGATTTATTCTCATATAAATTTACATCTAATTCTTGATGAATTAATT
TTTTAATTGATATAAATTTTATTGAAAAATCTTTATGTAAATCGCTTTTGATCTTTTTTTGAAA
AAAATTACTTTTTTATAAATTATTAATAATAAGAATTAATAACAATAATGCATTAAAATATG
TAATATTTACATTATTATAAAATGAATATTTATTTATTAATTATTTAATAGCC

382. >MSTRG.8164.4 Gene=MSTRG.8164
GAGAGCGGCCACGGGGGTTGGCGGAGGGAGAGCCCGGTGGACGAGCGGTTATTTCAAGCG
ACGACTCTTCTGGCACTTCGCCCCGAGCTTTCCTCGAGCGCATCCTCTCGCCTCGGACTCTC
GCGTTCTCGTGTCAGTGTCGCCGCGACGCCAGCCACAGTCAGTCAGTCAATCAGTCAATCG
GTGAACGGTGGGAGAGACGATTGGAAAATTTAAAGATCGTCGGCTCGGAAAAGAGGAAA
ATAAAATAAAATAAAATAAAATAAAATCGTTGGGGAGGAAAGTTAGGGTACTGTGGATAA
TTTCGAGGGGAAATTGAAGAAAAAGAAATTCCAAGAAGATTAAGAAAAATCCTGTCTGAT
CTAACCTAGAAAAAGATCCAACCTAGAAAAAGAGAATCTTTTCGAAAAAAAGAGCGAAA
ACGACCGATTTCCAAAGAGATCCTGCTTCTCCATTTAATCACGAGTGAAGAGTTTCAAAGTT
TCGAAGGATTCCCGCGGAAAGGTGGCCGGAAGTGCGTTCGCGACTCTTCTTTCGCGTGATC
AGTGACCGAGCAGTGGAAGTAAGGGTGGAACAGGTGTTTCCCCAACACGGTGGTGGTTGTC
CACAGATTGTAAAAAAGAAATATATAATATAAATATATATAAACGAAAAGTGTCGAAAAA
TTGTGCGAAATATCGTTCTTTTTTTTTTCCTTTCTTCGATCTTCGCGTTTCGAGGGAGGAAGGG
AGGAAGGGAAGGAGGGAGGGAGGGAGGGGAAGGGGGGGAGGGGGAGATCGATCCGGCA
CGAGAAGATGGAAAGGAGAGGAGGTGGATTTATAATACTCTCCGCTCTGCTCGCCTGAAAT
AACAGGAGGAATGCGCGCGTGGTGAATCAGCTTCCTCTTTGGAGAGTGCGAATTTCCTTCC
GACAGCGCGGCCACGCGATCCTGGATCGAGCGGCCAGGATCTCTGGATCGTGTTCGGGTGT
GTGCGCGCGCGCGCGCGTTCGTGCACCTTTGAGAAAAGCCAGAAACGCCAGAAGCTCGCG
ACACGGGGAGGAGGGAACGGGAGAAGAGAGGGCCCTCATCAACTCCGGTTTTTCTTGGAA
AGGAGTCGCGATTGCCTAGGGCCATGACGATATTTCGCTGGTTTCCTGGTGGGTCGTGCGAC
AATGTCGGGACGTCAACTCCCTGTAACGAACGCTTATTTATCCGGCTCGCGATCTGGCGGA
CCACCGCGTCGAAAACTGGTTTCTCGATGAATGAACGAAGAAGAT

383. >MSTRG.8168.2 Gene=MSTRG.8168

TAATGGAGTAATGTTCTCTTTCTTATAGTAGTGTATGGTTTCTAACGAAAGTATTATATGTTA
TAATTCTTCTATCTAGAATGCAACGAGTTATTACTACAATTTCTCGAAATATGGCTTTGTTGA
GCCAAGTAAGAATGGTTGGAGAACGTGGATCAGGAGCAGGTAAAGGAGGTGGTGGTGGTG
GCTCTATTCGTGAAGCTGGAGGATCTTTTGGAAAAATGGAAGCCGCTCATGAAGATCAGTA
TTTCTATAATCTGCAAAAACAACAGCTACAAAAACTTAAAGAAGATCTTCACGATGAAATC
TCTTTCCATGAAGAACAAATTAAACGTCATCAAGAAGCTATAAACCGCCATAAAAAAAGA
ATCACAGAAATGGATAAGAAATAAAATTATCCATGTATTTACTCTTTAAGCGTTTGTTATTC
TTTATAATGTAATGCTCTT

384. >MSTRG.8177.1 Gene=MSTRG.8177
ATTAACTGCTAATTTCATCGACAGTAATTAGCGCGCACGAGCGCGCGCCTGTCACGCGCTG
ATTTAATTGCACTCTCGCGGACACTCTCTTTACTTCCTCCCCCACCAGTCTTGTGGTGCATCT
TTTCCGTTTCTCCGCTTGGAACCGATCGCGCCACCGTTCCCGCAACTAGCCTTCTTGTTTTGG
TCGAATCCAATTAGCCTCTGAAATGAAACTGAAGACGAATGGATGCGGGTGAATGCGCGC
GCGCATGCTTGCAGGATATCCGCGGATGCAGGAGGCAGCCGGCTGGGAGGATGACGGCGA
GCGCGCAAGAGCGAGAAGTCGGTAACTTAACTGGCCTAAATGGGTTTAACTCGATGTGATT
TTGCTGGCGAACCACTAAATTGAACGAATACGAACTAAACTGAACTCAACTAAGCTAATCA
TATCACATTACTAGCGTTATTAGCCGAGAGACGATATAAGTGAAGGTCCGGTGGTACCAGG
CTGCAGTAACGGGTCGCCTCCTGCACTGACCCTCTGTACGACTTTCTTTTCGACCTCTTTTTC
TCGATCATAATGTTCGTGTCTTGTATTTATTCGATCTTTGAATTACGGAAATGTGTTTACGGT
CAAGTTTTATCGCAATTCGATTCCAAAACAACTTTTAGATTCGGAAATCGTGATTCAAAGGA
GAAAATGTAACATCGCAGCCCGATGACGTCGCATCCTTATTAACCCGACGTGGTCACATCC
GTCGTCATCCCGTTCTGTATTCACGTGCAGGGTGTTTCTCGTAATGATATCTTCATTTATGTA
TTGAAACTATCCTATAAAATTCTATCAAATTATGTATTTCATGTATTAAAATTTTCTAATATA
ATGGTACATATGATA

385. >MSTRG.8216.2 Gene=MSTRG.8216
CGTAGAAAGCCAGTTGGACGGCGGCGCCGCACCAGGTAGGCGTAGGGACGCGCCTTTTCG
ATCAAATAACGGCCGCTTGTGATTTCGTGCGCCCCTTTAGTGCCTCCCTTCCTCGTTGAAAC
GATTCAATTCGATTCAATTTTTCAATAGACGATTGTCGAGATCGAATGAGACAATTTGGAAG
AAAGAATTAAGAAGAGAAGGAAAGATAAAAGAGGACTTAGAGAATAAGAGAGAGAGGA
GGATCGGAATTCTCGATCTTCTCGATCGTGATATCACTTTTAATTGAGTGAGAGGAGGGAGA
ATAATCGTAATCGTGATCGAGTATAGTACACGGATAGAAGGAAAGTTTGGAAAGGGACGG
AAGTCGGTGGATCAAGTGCCTTTTTTCTTTCTGTTTCGCGGGGATACCGTTGGTCCCGCCCCT
CTGGGCGGCTCCTGGCACCCGGAAGTATCCTCGGCCCGAATACTGACGGACCTGTCCTCCC
AGTACGAGCTCCCATCGCCGCAACCAGGATGTTTTTGTCCTCGAGAGCTTTCCCGCCGATTC
TCTTCGCGGCGCTCTTCCTCTGCATCGGGCTGGCACACGGTATCAGATGTTACAAATGCGGA
CAGTACAACGAGGGAGTCGGCAGCATCACGCCGTGCATCAATTACACCGCTCACATGCATC
TGAAGGAATGTCCACCCTCGGCCGAATGGTGCATCAAATATGTCAGCGAAGGGTCGATAGT
TCGAGACTGCGTGCCAACGTGTGTGGAAAAGGAGGCCTGGAGCACGATGACGTATTGTTGC
CAGCAGGACGGTTGCAACTCGGCCCCATCGGTTGTATCCAGCAACAGCCTCGCGATCGTCG
CGATCACGATGGTGCTCCTCCTTGTGCTTCGTGGCTAATACGTCGTTTCCAGCGACCAGGTG
ACCGCGAATGGAAGCTACGGGATCCCTTCGACATCACCGCGCTAACCATCGATTATTGTCC

TGGCCTTTATCTTGCTGATTGGACGCTGACCAGTCGATAGTTGTCGAACAATCTTGATCTGT
GAAATGAATATCTGTGAAATTTCCATCCTTCAATCGATTTTCTACGCGCTCCACCATTCTCAT
AGATTCTTTGGTTTGAATAAATTCTTTTTTTTTTTGGTTTGATAAAGTAAGTTTGAAAAAGTAT
GTGATGCTCCATTTTTCTATATTTTCTTTTTATTTACCTTTCTATTATTCTTTTATTTGCATCGAC
GTTTATATTTTTCTTCTCCTTTTTCTGGGTTAATGAAGAAACCATTCTTAATACGTAATAAAA
TGTATAAATTAAACGGAAAGTTTAATTGGTGATTCAAGTTTCTTAATTTCACTTTAGTTCACC
AGCGAAACGCTCTCTCTGGAGAATTTTATTCGATTTAATTTAATTTTTGTGTAAAGAAATTCC
GTTCTATATACGTATACGAAAGTTCCTTAAGTTGTTTAATTCGTTTCCCCTCTCTCGTTAAAT
GTAAAACATTTAAGGAAATTAGGAACATCCGTGAGAGAGGTTAAGTTCTTGGTAAAATTCA
ATCTTTGTTCTTCGAGAGATAAAGATATTTCTTTTCTCTTTTGATTGAAATATTGTTTAAATTG
GATAATTTAAATTTTGTACAGATACATTCTACTAACTTCTTTTATTCTTTTTTTCTTTTTATCTA
AAGAAAACAATTTTTCTATGTTCATCAACCTTACACTTTGAACAATCTTATGATTCCATGAG
ATACTTTCTTATGGATTCTTTTTATGACATTTCATACATTCGAAGAAGATCGAATTCTTTTCA
CAGTTGAATTTCCATCGGTCAGCTTCCTTTTATTCGAAGCTGACACTCGCTTCTTGAGCGACT
CTTGAATTCGATTTATTCGAGAACTGGTTCGCCGATTCTTCCACCTCTTTTCTTCACTTCATAC
CACTGCAACCATGAGTGCTCGATCTAACAAGAGCTGGAAAAGGGGGATTCTCGATGACAC
GTTCTCTGCTTCGAACAGAGACGAAATGGAATTCTTCGAGGTTTGGAAAATGTTATTTCGAT
GATTCATTCGTATTACTTTCCTTATGTAATCTTTTATCATTAATTTCTTGAGTATCGATTTAAT
CCAAAGAAATTTCTCATATTACGATATTATATGATCCTGAAAATTATAGTATTTGGATTATT
ATTTTATAATAATAGTTAATAATTAATTGAATATTCATTAAATTTAAATTTGTTAAATATTGC
ATTAATTCGATTAGATTATTGTCGAATATTGATATGAAATAGATAATTGTTATAATTTTGTTA
TTTTCTTTTTATTATACATAATTTGAAAAGATTGAAATTTATTTATTCGACGATACCATTAGA
TTATCCGTCTATTCGCCATATACTTTATTTCCTTTTGCGTTTTGGTTTTGCTTCAAAAACCTCT
CAGCTTCATTCACGATGATCGATTTTCGATTCCTTCTTCCTCCTTCTATCGGTTTCCCATTTAG
TTCTTCGACTTTTTCCATCCCATTTTCCCAAGTTTATTCTCCTCTATTTTCCTTTCATGACAACA
CTTGCCACATATTCCACTGGATCTCTCTTCAAAATATTTCAAAACTTATTCCTGTAATATTCA
AACGCAAAAAAAATAAATAAATAAATAAATAAATAAATAAAGGAAAACGTGTATTATAA
TTCTAATATATTTTCGAAAAGAATCGAAGGAAAATATGAAATATAAAATGAGAGAGAAAA
AAAGGAGAAATAGACAACTATCGATTCATCGTCAATTCCAATCGAGCAAAAATTGGCTAC
CGCGATCGCGAGAAATAGAAAAAAAAAAAAAAAAAAATTGGACCACAAAAAACCCGTGAT
AGCGGATGAGATAAAAGCGGAAGTACGGCGAAAGCGCGCGAAAAGGATTTCTCGATGGTT
CGATCGTTCGTGGTCAATCCGACATATTTCTATAGTTCGTAAGATTCGTTCACGAAGGAGAA
AAAAGAAGGGCAAAAAAAAAAAAGAAAAAAAAGAATAAGTTAGTAATTTGGAAGAAATG
ATAAAAGAGAAAAAAAGAAAAAAAAAAATAGAAAAGAAAAAATGAAACCCGCGTGGATA
TATATATATGTATACCTATATATATATAACCTACATACAGACAACAGTTTAATGAGGAACA
GTGAGGATAATAATGATAATGTTATTATTATTATGATTACTATTATTACTATTATTATTATTG
ATTATTATTATTATGAATTATTATTATTACTATATTGCTACTAATTACTACTATTACCATTAAC
TATTACCATTACTAAAAGATTAACGACACTAAACTGGAAGTATTACACCGATTATACATAA
ACTTAATTATACATATATATATATATATATTATAAATATATACATAGTAGCGATAAAAGATC
GATAGGCGTCGAAATCGAATGGAGAGTTTGTCGATTATTATTACTACCGTTGTAACCGCTTG
AAGTAGCCTTAATAATTGTAATTAACGAACGCACGCGTGAGATATTAAAAAAAAAAAAAA
ACTGCGTAATATCTTATTGGTTGGTGTGAGGAGATTGCGTATTACCTCGTTTTAATCTTGTTT
TCTAAATATTTATTTTTTCCTTCCTCTTCTTTTCCAATGATTAAAAGTTTTGAAAGAAGTCGTC

AAGTAGAATTATAAACACTTCTACGAAGAATTTTATTAAATAACAATTTTTCTCTTGCACGG
TATCCTATTTCCATGCTGCTCGTATTGGTCAATTAAAAATCATTCGTCTTAGTCAATTACCAC
GAGTAGAGATTTACACGAGTTGAATTGCTATGAGAGATAAATTTAAAACGGACTTTTTAAT
CAAAATAAATAATTTTCCCAACTTTCTCTGATTCACGAATTTTTCTCTTTTTTTTTGTTTCCCC
TTCGCTAAATCAAATAATGGAAAACAAGAATTAAATATTTATTAAACTTAATTTTTATCGTT
TTCGATATAGACAATCGAGATTAAAACGAGAATATTCGTTCTCTTCTCAGGTTAACCAACAT
CTGAAATTCCTAAATTGTAGAATGATTCATCCTAGCCGGTAAGCGAGTCACAAATAGTAGA
GATGATAATCTACTTTACCTGTTTCACGAATGAAAGCAATGAAAATAATAAAAAAAAAAA
AAAAAAGAAAAAAATATCACGAAGGTAGCATACAGAAATTATTAGCGATTATATACCTGT
TCTTCTGAGATTAGGCGAAGAGATGATTTATCTAAAGTGAAAATTAGCTCGAGTCTAGCGT
AAATGATACTTAAACACTTAACCTGTTTCCCGTCACGGATGTCAACCGATCGATTAACATTG
TTATTGTTGGACGAATGTTTCGAGGTAGAAATTATTCCAAATTCGAATAATTTTTCAATTTTT
TTAAAAATAATGATAGAAATCGATTCGAATGTGGATTTTCGATGCAACGTTGTTAATTCGAG
ACGGAGAGGTTAGGTTTTGATTACGTACTGCCATTACGTATTTGAATGGAAATCGATAAATT
GACAAATTTAATATAAACATTCTGTAATACAGTAGAGAAAATATATGTGGAAAATTTATAA
TACAATGTAATTTTAAAATTTTTCTTGTATGATAAGAGCTTATATAATGAAAATATATATAA
TAGTATTCGTGAAATATATATTTGATGAAAACATCTACAATAGTATTTTCAATATTTAATGA
CACTAGTGTGAAAATTTGTCAGGTGATCGTATCAATATTGGAAATTTGTCACATCACAGGA
ACCGTATATTCGTACAAATATTTGAAATTTGTCACGATGTTACGATCAAAATGTCACAATGT
TCGCCATGATAAAATTCAGCCATATGACAGCAAATAAATTAATCTTTGCATTTTACTAGTCA
TCTTACAATTTCTTCTATATAACTGTTCTTATATCTAATATAATAAAAGGGGAGGGAAAAAT
CCCAAATTGAACAACGACACGAATCTATTTATATTAATACTTTCATTATTGAATCAAATAGA
AATAAGTTGCTTAAGATTCCAAATCTCCACGAATAATAATCTATGAGTTCTAATCTATAAAT
CTATTCTAATTTAATAAATCAAGATTCTATTAATTTAATCAAGTAGAAAAAATGTTTCATCA
AAAATCCATGAAGAATCGAATTCGATATTCCACACCACGAACATTATATAATCCCGTTTTA
AATTGAATAAAAATATTTGTTAAAAGCTACAATTGTTGCGGATCACTTCGTTCAAACTTCGC
TTACACTTTATCCATGTATTTTTATAACACAAAGTGACAAAGGTGGCGCTTGTGTCCACGTG
GATACGCAATCATTGTGGCTCTATCTACCTTCCAAATTTTCATAATCTCGATCTTCGATTGTT
CGTACATACATACATATACGATTTAAATTGCAAAAAAAAAAAAAAAAAAATAACAAAAATA
ATTTTAAAGCAAAAAAATTTTAAAGCAATTCTACCTCGAATCATTCCGTATTATATCCATAT
CAATAAAAAAATATATAAATAAAAAAAAAAAGAACGAAGGATTATTGAATATCGAAGTTTT
ATCAGGACGCACACAAGCAAAAAAAGAGCGAAAAAAAACCATGACTTTCGGATAATATA
TTAACAAGTTATATATTAACGATACTATAATTCTAGGCGACTTCCTTAGCGTGTAAGATTTT
ATTATATTCAACAAGGAACGCTCGATAATAAATCCGACATTGTTCCAAGCTCCTAAATAAA
TGAGTGAAGATTATTAACAAAAAAAAAAAAACAGAATAAACGAAAAAAAGAAAAAAAGA
AAAAGGATAAAAAGAAAAGTTTCTACATCGATTATCGGTTATCGAATAAAGATCGATAAG
TGGATACGTGATTTCCGTGCGAGAAAAGTGTTTAGAAAGTGTGAGTATAGATGGCGTTTCA
ACGTACAGCGTAACATTTTACATTTGCTTCGAGCACTATTTCAATAGATACATTATTAAAGA
AGTTCGTGAGAGAAAAGATAAAAAAAAAAACGTCTAAACGAG

386. >MSTRG.8216.1 Gene=MSTRG.8216
CGTAGAAAGCCAGTTGGACGGCGGCGCCGCACCAGGTAGGCGTAGGGACGCGCCTTTTCG
ATCAAATAACGGCCGCTTGTGATTTCGTGCGCCCCTTTAGTGCCTCCCTTCCTCGTTGAAAC

GATTCAATTCGATTCAATTTTTCAATAGACGATTGTCGAGATCGAATGAGACAATTTGGAAG
AAAGAATTAAGAAGAGAAGGAAAGATAAAAGAGGACTTAGAGAATAAGAGAGAGAGGA
GGATCGGAATTCTCGATCTTCTCGATCGTGATATCACTTTTAATTGAGTGAGAGGAGGGAGA
ATAATCGTAATCGTGATCGAGTATAGTACACGGATAGAAGGAAAGTTTGGAAAGGGACGG
AAGTCGGTGGATCAAGTGCCTTTTTTCTTTCTGTTTCGCGGGGATACCGTTGGTCCCGCCCCT
CTGGGCGGCTCCTGGCACCCGGAAGTATCCTCGGCCCGAATACTGACGGACCTGTCCTCCC
AGTACGAGCTCCCATCGCCGCAACCAGGATGTTTTTGTCCTCGAGAGCTTTCCCGCCGATTC
TCTTCGCGGCGCTCTTCCTCTGCATCGGGCTGGCACACGGTATCAGATGTTACAAATGCGGA
CAGTACAACGAGGGAGTCGGCAGCATCACGCCGTGCATCAATTACACCGCTCACATGCATC
TGAAGGAATGTCCACCCTCGGCCGAATGGTGCATCAAATATGTCAGCGAAGGGTCGATAGT
TCGAGACTGCGTGCCAACGTGTGTGGAAAAGGAGGCCTGGAGCACGATGACGTATTGTTGC
CAGCAGGACGGTTGCAACTCGGCCCCATCGGTTGTATCCAGCAACAGCCTCGCGATCGTCG
CGATCACGATGGTGCTCCTCCTTGTGCTTCGTGGCTAATACGTCGTTTCCAGCGACCAGGTG
ACCGCGAATGGAAGCTACGGGATCCCTTCGACATCACCGCGCTAACCATCGATTATTGTCC
TGGCCTTTATCTTGCTGATTGGACGCTGACCAGTCGATAGTTGTCGAACAATCTTGATCTGT
GAAATGAATATCTGTGAAATTTCCATCCTTCAATCGATTTTCTACGCGCTCCACCATTCTCAT
AGATTCTTTGGTTTGAATAAATTCTTTTTTTTTTTGGTTTGATAAAGTAAGTTTGAAAAAGTAT
GTGATGCTCCATTTTTCTATATTTTCTTTTTATTTACCTTTCTATTATTCTTTTATTTGCATCGAC
GTTTATATTTTTCTTCTCCTTTTTCTGGGTTAATGAAGAAACCATTCTTAATACGTAATAAAA
TGTATAAATTAAACGGAAAGTTTAATTGGTGATTCAAGTTTCTTAATTTCACTTTAGTTCACC
AGCGAAACGCTCTCTCTGGAGAATTTTATTCGATTTAATTTAATTTTTGTGTAAAGAAATTCC
GTTCTATATACGTATACGAAAGTTCCTTAAGTTGTTTAATTCGTTTCCCCTCTCTCGTTAAAT
GTAAAACATTTAAGGAAATTAGGAACATCCGTGAGAGAGGTTAAGTTCTTGGTAAAATTCA
ATCTTTGTTCTTCGAGAGATAAAGATATTTCTTTTCTCTTTTGATTGAAATATTGTTTAAATTG
GATAATTTAAATTTTGTACAGATACATTCTACTAACTTCTTTTATTCTTTTTTTCTTTTTATCTA
AAGAAAACAATTTTTCTATGTTCATCAACCTTACACTTTGAACAATCTTATGATTCCATGAG
ATACTTTCTTATGGATTCTTTTTATGACATTTCATACATTCGAAGAAGATCGAATTCTTTTCA
CAGTTGAATTTCCATCGGTCAGCTTCCTTTTATTCGAAGCTGACACTCGCTTCTTGAGCGACT
CTTGAATTCGATTTATTCGAGAACTGGTTCGCCGATTCTTCCACCTCTTTTCTTCACTTCATAC
CACTGCAACCATGAGTGCTCGATCTAACAAGAGCTGGAAAAGGGGGATTCTCGATGACAC
GTTCTCTGCTTCGAACAGAGACGAAATGGAATTCTTCGAGGTTTGGAAAATGTTATTTCGAT
GATTCATTCGTATTACTTTCCTTATGTAATCTTTTATCATTAATTTCTTGAGTATCGATTTAAT
CCAAAGAAATTTCTCATATTACGATATTATATGATCCTGAAAATTATAGTATTTGGATTATT
ATTTTATAATAATAGTTAATAATTAATTGAATATTCATTAAATTTAAATTTGTTAAATATTGC
ATTAATTCGATTAGATTATTGTCGAATATTGATATGAAATAGATAATTGTTATAATTTTGTTA
TTTTCTTTTTATTATACATAATTTGAAAAGATTGAAATTTATTTATTCGACGATACCATTAGA
TTATCCGTCTATTCGCCATATACTTTATTTCCTTTTGCGTTTTGGTTTTGCTTCAAAAACCTCT
CAGCTTCATTCACGATGATCGATTTTCGATTCCTTCTTCCTCCTTCTATCGGTTTCCCATTTAG
TTCTTCGACTTTTTCCATCCCATTTTCCCAAGTTTATTCTCCTCTATTTTCCTTTCATGACAACA
CTTGCCACATATTCCACTGGATCTCTCTTCAAAATATTTCAAAACTTATTCCTGTAATATTCA
AACGCAAAAAAAATAAATAAATAAATAAATAAATAAATAAAGGAAAACGTGTATTATAA
TTCTAATATATTTTCGAAAAGAATCGAAGGAAAATATGAAATATAAAATGAGAGAGAAAA
AAAGGAGAAATAGACAACTATCGATTCATCGTCAATTCCAATCGAGCAAAAATTGGCTAC

CGCGATCGCGAGAAATAGAAAAAAAAAAAAAAAAAAATTGGACCACAAAAAACCCGTGAT
AGCGGATGAGATAAAAGCGGAAGTACGGCGAAAGCGCGCGAAAAGGATTTCTCGATGGTT
CGATCGTTCGTGGTCAATCCGACATATTTCTATAGTTCGTAAGATTCGTTCACGAAGGAGAA
AAAAGAAGGGCAAAAAAAAAAAAGAAAAAAAAGAATAAGTTAGTAATTTGGAAGAAATG
ATAAAAGAGAAAAAAAGAAAAAAAAAATAGAAAAGAAAAAATGAAACCCGCGTGGATA
TATATATATGTATACCTATATATATATAACCTACATACAGACAACAGTTTAATGAGGAACA
GTGAGGATAATAATGATAATGTTATTATTATTATGATTACTATTATTACTATTATTATTATTG
ATTATTATTATTATGAATTATTATTATTACTATATTGCTACTAATTACTACTATTACCATTAAC
TATTACCATTACTAAAAGATTAACGACACTAAACTGGAAGTATTACACCGATTATACATAA
ACTTAATTATACATATATATATTATAAATATATACATAGTAGCGATAAAAGATCGATAGGC
GTCGAAATCGAATGGAGAGTTTGTCGATTATTATTACTACCGTTGTAACCGCTTGAAGTAGC
CTTAATAATTGTAATTAACGAACGCACGCGTGAGATATTAAAAAAAAAAAAAAAACTGCGT
AATATCTTATTGGTTGGTGTGAGGAGATTGCGTATTACCTCGTTTTAATCTTGTTTTCTAAAT
ATTTATTTTTTCCTTCCTCTTCTTTTCCAATGATTAAAAGTTTTGAAAGAAGTCGTCAAGTAG
AATTATAAACACTTCTACGAAGAATTTTATTAAATAACAATTTTTCTCTTGCACGGTATCCTA
TTTCCATGCTGCTCGTATTGGTCAATTAAAAATCATTCGTCTTAGTCAATTACCACGAGTAG
AGATTTACACGAGTTGAATTGCTATGAGAGATAAATTTAAAACGGACTTTTTAATCAAAAT
AAATAATTTTCCCAACTTTCTCTGATTCACGAATTTTTCTCTTTTTTTTTTGTTTCCCCTTCGCT
AAATCAAATAATGGAAAACAAGAATTAAATATTTATTAAACTTAATTTTTATCGTTTTCGAT
ATAGACAATCGAGATTAAAACGAGAATATTCGTTCTCTTCTCAGGTTAACCAACATCTGAA
ATTCCTAAATTGTAGAATGATTCATCCTAGCCGGTAAGCGAGTCACAAATAGTAGAGATGA
TAATCTACTTTACCTGTTTCACGAATGAAAGCAATGAAAATAATAAAAAAAAAAAAAAAA
AGAAAAAAATATCACGAAGGTAGCATACAGAAATTATTAGCGATTATATACCTGTTCTTCT
GAGATTAGGCGAAGAGATGATTTATCTAAAGTGAAAATTAGCTCGAGTCTAGCGTAAATGA
TACTTAAACACTTAACCTGTTTCCCGTCACGGATGTCAACCGATCGATTAACATTGTTATTGT
TGGACGAATGTTTCGAGGTAGAAATTATTCCAAATTCGAATAATTTTTCAATTTTTTTAAAA
ATAATGATAGAAATCGATTCGAATGTGGATTTTCGATGCAACGTTGTTAATTCGAGACGGA
GAGGTTAGGTTTTGATTACGTACTGCCATTACGTATTTGAATGGAAATCGATAAATTGACAA
ATTTAATATAAACATTCTGTAATACAGTAGAGAAAATATATGTGGAAAATTTATAATACAA
TGTAATTTTAAAATTTTTCTTGTATGATAAGAGCTTATATAATGAAAATATATATAATAGTAT
TCGTGAAATATATATTTGATGAAAACATCTACAATAGTATTTTCAATATTTAATGACACTAG
TGTGAAAATTTGTCAGGTGATCGTATCAATATTGGAAATTTGTCACATCACAGGAACCGTAT
ATTCGTACAAATATTTGAAATTTGTCACGATGTTACGATCAAAATGTCACAATGTTCGCCAT
GATAAAATTCAGCCATATGACAGCAAATAAATTAATCTTTGCATTTTACTAGTCATCTTACA
ATTTCTTCTATATAACTGTTCTTATATCTAATATAATAAAAGGGGAGGGAAAAATCCCAAAT
TGAACAACGACACGAATCTATTTATATTAATACTTTCATTATTGAATCAAATAGAAATAAGT
TGCTTAAGATTCCAAATCTCCACGAATAATAATCTATGAGTTCTAATCTATAAATCTATTCT
AATTTAATAAATCAAGATTCTATTAATTTAATCAAGTAGAAAAAATGTTTCATCAAAAATCC
ATGAAGAATCGAATTCGATATTCCACACCACGAACATTATATAATCCCGTTTTAAATTGAAT
AAAAATATTTGTTAAAAGCTACAATTGTTGCGGATCACTTCGTTCAAACTTCGCTTACACTT
TATCCATGTATTTTTATAACACAAAGTGACAAAGGTGGCGCTTGTGTCCACGTGGATACGC
AATCATTGTGGCTCTATCTACCTTCCAAATTTTCATAATCTCGATCTTCGATTGTTCGTACAT
ACATACATATACGATTTAAATTGCAAAAAAAAAAAAAAAAAAATAACAAAAATAATTTTAA

AGCAAAAAAATTTTAAAGCAATTCTACCTCGAATCATTCCGTATTATATCCATATCAATAAA
AAAATATATAAATAAAAAAAAAAGAACGAAGGATTATTGAATATCGAAGTTTTATCAGGA
CGCACACAAGCAAAAAAAGAGCGAAAAAAAACCATGACTTTCGGATAATATATTAACAA
GTTATATATTAACGATACTATAATTCTAGGCGACTTCCTTAGCGTGTAAGATTTTATTATATT
CAACAAGGAACGCTCGATAATAAATCCGACATTGTTCCAAGCTCCTAAATAAATGAGTGA
AGATTATTAACAAAAAAAAAAAAACAGAATAAACGAAAAAAAGAAAAAAAGAAAAAGGA
TAAAAAGAAAAGTTTCTACATCGATTATCGGTTATCGAATAAAGATCGATAAGTGGATACG
TGATTTCCGTGCGAGAAAAGTGTTTAGAAAGTGTGAGTATAGATGGCGTTTCAACGTACAG
CGTAACATTTTACATTTGCTTCGAGCACTATTTCAATAGATACATTATTAAAGAAGTTCGTG
AGAGAAAAGATAAAAAAAAAAACGTCTAAACGAGA

387. >MSTRG.8216.3 Gene=MSTRG.8216
CGTAGAAAGCCAGTTGGACGGCGGCGCCGCACCAGGTAGGCGTAGGGACGCGCCTTTTCG
ATCAAATAACGGCCGCTTGTGATTTCGTGCGCCCCTTTAGTGCCTCCCTTCCTCGTTGAAAC
GATTCAATTCGATTCAATTTTTCAATAGACGATTGTCGAGATCGAATGAGACAATTTGGAAG
AAAGAATTAAGAAGAGAAGGAAAGATAAAAGAGGACTTAGAGAATAAGAGAGAGAGGA
GGATCGGAATTCTCGATCTTCTCGATCGTGATATCACTTTTAATTGAGTGAGAGGAGGGAGA
ATAATCGTAATCGTGATCGAGTATAGTACACGGATAGAAGGAAAGTTTGGAAAGGGACGG
AAGTCGGTGGATCAAGTGCCTTTTTTCTTTCTGTTTCGCGGGGATACCGTTGGTCCCGCCCCT
CTGGGCGGCTCCTGGCACCCGGAAGTATCCTCGGCCCGAATACTGACGGACCTGTCCTCCC
AGTACGAGCTCCCATCGCCGCAACCAGGATGTTTTTGTCCTCGAGAGCTTTCCCGCCGATTC
TCTTCGCGGCGCTCTTCCTCTGCATCGGGCTGGCACACGGTATCAGATGTTACAAATGCGGA
CAGTACAACGAGGGAGTCGGCAGCATCACGCCGTGCATCAATTACACCGCTCACATGCATC
TGAAGGAATGTCCACCCTCGGCCGAATGGTGCATCAAATATGTCAGCGAAGGGTCGATAGT
TCGAGACTGCGTGCCAACGTGTGTGGAAAAGGAGGCCTGGAGCACGATGACGTATTGTTGC
CAGCAGGACGGTTGCAACTCGGCCCCATCGGTTGTATCCAGCAACAGCCTCGCGATCGTCG
CGATCACGATGGTGCTCCTCCTTGTGCTTCGTGGCTAATACGTCGTTTCCAGCGACCAGGTG
ACCGCGAATGGAAGCTACGGGATCCCTTCGACATCACCGCGCTAACCATCGATTATTGTCC
TGGCCTTTATCTTGCTGATTGGACGCTGACCAGTCGATAGTTGTCGAACAATCTTGATCTGT
GAAATGAATATCTGTGAAATTTCCATCCTTCAATCGATTTTCTACGCGCTCCACCATTCTCAT
AGATTCTTTGGTTTGAATAAATTCTTTTTTTTTTTGGTTTGATAAAGTAAGTTTGAAAAAGTAT
GTGATGCTCCATTTTTCTATATTTTCTTTTTATTTACCTTTCTATTATTCTTTTATTTGCATCGAC
GTTTATATTTTTCTTCTCCTTTTTCTGGGTTAATGAAGAAACCATTCTTAATACGTAATAAAA
TGTATAAATTAAACGGAAAGTTTAATTGGTGATTCAAGTTTCTTAATTTCACTTTAGTTCACC
AGCGAAACGCTCTCTCTGGAGAATTTTATTCGATTTAATTTAATTTTTGTGTAAAGAAATTCC
GTTCTATATACGTATACGAAAGTTCCTTAAGTTGTTTAATTCGTTTCCCCTCTCTCGTTAAAT
GTAAAACATTTAAGGAAATTAGGAACATCCGTGAGAGAGGTTAAGTTCTTGGTAAAATTCA
ATCTTTGTTCTTCGAGAGATAAAGATATTTCTTTTCTCTTTTGATTGAAATATTGTTTAAATTG
GATAATTTAAATTTTGTACAGATACATTCTACTAACTTCTTTTATTCTTTTTTTCTTTTTATCTA
AAGAAAACAATTTTTCTATGTTCATCAACCTTACACTTTGAACAATCTTATGATTCCATGAG
ATACTTTCTTATGGATTCTTTTTATGACATTTCATACATTCGAAGAAGATCGAATTCTTTTCA
CAGTTGAATTTCCATCGGTCAGCTTCCTTTTATTCGAAGCTGACACTCGCTTCTTGAGCGACT
CTTGAATTCGATTTATTCGAGAACTGGTTCGCCGATTCTTCCACCTCTTTTCTTCACTTCATAC

CACTGCAACCATGAGTGCTCGATCTAACAAGAGCTGGAAAAGGGGGATTCTCGATGACAC
GTTCTCTGCTTCGAACAGAGACGAAATGGAATTCTTCGAGGTTTGGAAAATGTTATTTCGAT
GATTCATTCGTATTACTTTCCTTATGTAATCTTTTATCATTAATTTCTTGAGTATCGATTTAAT
CCAAAGAAATTTCTCATATTACGATATTATATGATCCTGAAAATTATAGTATTTGGATTATT
ATTTTATAATAATAGTTAATAATTAATTGAATATTCATTAAATTTAAATTTGTTAAATATTGC
ATTAATTCGATTAGATTATTGTCGAATATTGATATGAAATAGATAATTGTTATAATTTTGTTA
TTTTCTTTTTATTATACATAATTTGAAAAGATTGAAATTTATTTATTCGACGATACCATTAGA
TTATCCGTCTATTCGCCATATACTTTATTTCCTTTTGCGTTTTGGTTTTGCTTCAAAAACCTCT
CAGCTTCATTCACGATGATCGATTTTCGATTCCTTCTTCCTCCTTCTATCGGTTTCCCATTTAG
TTCTTCGACTTTTTCCATCCCATTTTCCCAAGTTTATTCTCCTCTATTTTCCTTTCATGACAACA
CTTGCCACATATTCCACTGGATCTCTCTTCAAAATATTTCAAAACTTATTCCTGTAATATTCA
AACGCAAAAAAAATAAATAAATAAATAAATAAATAAATAAAGGAAAACGTGTATTATAA
TTCTAATATATTTTCGAAAAGAATCGAAGGAAAATATGAAATATAAAATGAGAGAGAAAA
AAAGGAGAAATAGACAACTATCGATTCATCGTCAATTCCAATCGAGCAAAAATTGGCTAC
CGCGATCGCGAGAAATAGAAAAAAAAAAAAAAAAAAATTGGACCACAAAAAACCCGTGAT
AGCGGATGAGATAAAAGCGGAAGTACGGCGAAAGCGCGCGAAAAGGATTTCTCGATGGTT
CGATCGTTCGTGGTCAATCCGACATATTTCTATAGTTCGTAAGATTCGTTCACGAAGGAGAA
AAAAGAAGGGCAAAAAAAAAAAGAAAAAAAAGAATAAGTTAGTAATTTGGAAGAAATG
ATAAAAGAGAAAAAAAGAAAAAAAAAATAGAAAAGAAAAAATGAAACCCGCGTGGATA
TATATATATGTATACCTATATATATATAACCTACATACAGACAACAGTTTAATGAGGAACA
GTGAGGATAATAATGATAATGTTATTATTATTATGATTACTATTATTACTATTATTATTATTG
ATTATTATTATTATGAATTATTATTATTACTATATTGCTACTAATTACTACTATTACCATTAAC
TATTACCATTACTAAAAGATTAACGACACTAAACTGGAAGTATTACACCGATTATACATAA
ACTTAATTATACATATATATATATATATATATATTATAAATATATACATAGTAGCGATAAAA
GATCGATAGGCGTCGAAATCGAATGGAGAGTTTGTCGATTATTATTACTACCGTTGTAACCG
CTTGAAGTAGCCTTAATAATTGTAATTAACGAACGCACGCGTGAGATATTAAAAAAAAA
AAAAACTGCGTAATATCTTATTGGTTGGTGTGAGGAGATTGCGTATTACCTCGTTTTAATCTT
GTTTTCTAAATATTTATTTTTTCCTTCCTCTTCTTTTCCAATGATTAAAAGTTTTGAAAGAAGT
CGTCAAGTAGAATTATAAACACTTCTACGAAGAATTTTATTAAATAACAATTTTTCTCTTGC
ACGGTATCCTATTTCCATGCTGCTCGTATTGGTCAATTAAAAATCATTCGTCTTAGTCAATTA
CCACGAGTAGAGATTTACACGAGTTGAATTGCTATGAGAGATAAATTTAAAACGGACTTTT
TAATCAAAATAAATAATTTTCCCAACTTTCTCTGATTCACGAATTTTTCTCTTTTTTTTTTGTTT
CCCCTTCGCTAAATCAAATAATGGAAAACAAGAATTAAATATTTATTAAACTTAATTTTTAT
CGTTTTCGATATAGACAATCGAGATTAAAACGAGAATATTCGTTCTCTTCTCAGGTTAACCA
ACATCTGAAATTCCTAAATTGTAGAATGATTCATCCTAGCCGGTAAGCGAGTCACAAATAG
TAGAGATGATAATCTACTTTACCTGTTTCACGAATGAAAGCAATGAAAATAATAAAAAAAA
AAAAAAAAAGAAAAAAATATCACGAAGGTAGCATACAGAAATTATTAGCGATTATATACC
TGTTCTTCTGAGATTAGGCGAAGAGATGATTTATCTAAAGTGAAAATTAGCTCGAGTCTAGC
GTAAATGATACTTAAACACTTAACCTGTTTCCCGTCACGGATGTCAACCGATCGATTAACAT
TGTTATTGTTGGACGAATGTTTCGAGGTAGAAATTATTCCAAATTCGAATAATTTTTCAATTT
TTTTAAAAATAATGATAGAAATCGATTCGAATGTGGATTTTCGATGCAACGTTGTTAATTCG
AGACGGAGAGGTTAGGTTTTGATTACGTACTGCCATTACGTATTTGAATGGAAATCGATAA
ATTGACAAATTTAATATAAACATTCTGTAATACAGTAGAGAAAATATATGTGGAAAATTTA

TAATACAATGTAATTTTAAAATTTTTCTTGTATGATAAGAGCTTATATAATGAAAATATATA
TAATAGTATTCGTGAAATATATATTTGATGAAAACATCTACAATAGTATTTTCAATATTTAA
TGACACTAGTGTGAAAATTTGTCAGGTGATCGTATCAATATTGGAAATTTGTCACATCACAG
GAACCGTATATTCGTACAAATATTTGAAATTTGTCACGATGTTACGATCAAAATGTCACAAT
GTTCGCCATGATAAAATTCAGCCATATGACAGCAAATAAATTAATCTTTGCATTTTACTAGT
CATCTTACAATTTCTTCTATATAACTGTTCTTATATCTAATATAATAAAAGGGGAGGGAAAA
ATCCCAAATTGAACAACGACACGAATCTATTTATATTAATACTTTCATTATTGAATCAAATA
GAAATAAGTTGCTTAAGATTCCAAATCTCCACGAATAATAATCTATGAGTTCTAATCTATAA
ATCTATTCTAATTTAATAAATCAAGATTCTATTAATTTAATCAAGTAGAAAAAATGTTTCAT
CAAAAATCCATGAAGAATCGAATTCGATATTCCACACCACGAACATTATATAATCCCGTTT
TAAATTGAATAAAAATATTTGTTAAAAGCTACAATTGTTGCGGATCACTTCGTTCAAACTTC
GCTTACACTTTATCCATGTATTTTTATAACACAAAGTGACAAAGGTGGCGCTTGTGTCCACG
TGGATACGCAATCATTGTGGCTCTATCTACCTTCCAAATTTTCATAATCTCGATCTTCGATTG
TTCGTACATACATACATATACGATTTAAATTGCAAAAAAAAAAAAAAAAAAATAACAAAAA
TAATTTTAAAGCAAAAAAATTTTAAAGCAATTCTACCTCGAATCATTCCGTATTATATCCAT
ATCAATAAAAAAATATATAAATAAAAAAAAAAGAACGAAGGATTATTGAATATCGAAGTT
TTATCAGGACGCACACAAGCAAAAAAAGAGCGAAAAAAAACCATGACTTTCGGATAATAT
ATTAACAAGTTATATATTAACGATACTATAATTCTAGGCGACTTCCTTAGCGTGTAAGATTT
TATTATATTCAACAAGGAACGCTCGATAATAAATCCGACATTGTTCCAAGCTCCTAAATAA
ATGAGTGAAGATTATTAACAAAAAAAAAAAACAGAATAAACGAAAAAAAGAAAAAAAG
AAAAAGGATAAAAAGAAAAGTTTCTACATCGATTATCGGTTATCGAATAAAGATCGATAA
GTGGATACGTGATTTCCGTGCGAGAAAAGTGTTTAGAAAGTGTGAGTATAGATGGCGTTTC
AACGTACAGCGTAACATTTTACATTTGCTTCGAGCACTATTTCAATAGATACATTATTAAAG
AAGTTCGTGAGAGAAAAGATAAAAAAAAAACGTCTAAACGAGAAAATTGTCTATTTTTTTT
TCTTCCTGCATTGATTTGTAATGATGAATGTGAAAGTTTATTTGTTTATGATATAGTTGAGTG
AATTTTACGCATATATATGATATGATTTGTATGAAGAACTTTTTTATTTGAATTTTATTTTTAA
TTTTTGTTATTGATCATAGAAATTTAAAAGTTAACTTGGTAGGATAATAAAAAGTGATTTTTT
GATTTTCTATTGAATTATTATTTTTTTATCTATAAGTTGAGAATATGTTTGAAATAATTCATGC
AAGAG

388. >MSTRG.8241.1 Gene=MSTRG.8241
CGCTCTGGCAAACGGATCGAGATTTTATGTTCTGCGTATATAACGTGCTTTTGACGAATACC
TTTCTATTTGTAAATTTCCAGTTTCCTTTCGTGTGAATGCCGCTCCGTGAAGCGTTTAATGTAT
TCCTCTTTTACATCGATCCCTGATGTACATTCGATAGCAGAGCACCGTGCGGTCGTAACGGC
GACACGAAGAAAGCGCAATGGCCGACCGCTGCACCTCTGCTGGTAGCTTCGATCCTTCCTG
GCCACGAGGACTTTGTACACACTTGATCGTTCAGGGATCGACACGCGCGCACGATACGCCT
TTTCCGAATCCCTGAGACCGTTAAGATCCGTGGATATTTTTCTTCCGTTTCACGAACGCAGCT
AGGGACGGTTCGTACTGTCCGATCGTATGAACCCGCTGCCACTCGCACCAGACCGCCACGC
ACCGTCCCGCCCGCTGGAT

389. >MSTRG.8247.1 Gene=MSTRG.8247
TTCTTAAGTTTGGATTGTGTCTTCACAAAGCAAGAGATAGATTCGAAGGAGCTCGTGCTTTC
GAGCACATAGTTTACGAGTTCGATCGCAGTATCTGGTTTCAGCGAGTGTTATGTGAGTTGGC

GTAGTGGCGAAAAAGAGCTTAAAGTGAAAGGAAAAATCGCATGAAGAGGCAGAAATTGA
CGGGCGCTGCTTGACGGATAATAATATCAGTTTTTAAAATAGAATGAACATCGTTTAGTGC
GCGTACATATACAGACACGTACGAGATATTGCTACAAAGTTAATTTCACGTTTTACGAACA
TATATGTACACGCGCGTGCGCGTTGCGAGGGTACGAATACATATAGTTTCGCTGTCGAACG
GTGATATATTGTGAAAAATCCGTGTGGAGCAGCGAAAACAAACAACTCGTGTCCGCCATCT
TGTGCCTCTCCAGCTACGTTGAAAGGAAAGTGAGAGAATTCGGACCGACGTGTGTGATTTT
ATTCGGTGAGAAAAAAGAGTGTAAGTAGAGGTGACCAACGGTCACCGAATGTGGTCAAAA
TTGCAAAAAACACGAATAGGGAGGACTGCGAGAGAACTCAAAGAAAGCGAGACGAGGTA
GCATTGCGCTCATAGGAGGTTCGTAGGATACCCCACCAAGTTTATTCCTCCGCGTTCCCCCT
ACCCTTTTGCCCTGGATCCCGAGATTATTGCGCAGTTAGAAAATTCATTGAATACAAACATC
GTCAAAAACTTCGTTGCATCTTTGGAAAGTGACCCGCGGACATTATACGACGGGTTGGTTG
ACTTTGTGAGTGATACGAAATTATTATAAAGCCATGACGAATTCGGTACCGAAATTGAAAA
ACAAGGTAGACCGGCAGGGTTCGCCAAATGTCGGCATTGATCGACATCATCACCGTTCGAT
AATTACACTCAAACCGCCGGCGTATGTCCACATGAGTGGGAGATCTCATGGCAGAAGTTCA
ACCGG
CAGATTAAGCTGCAGCCCACACGGTTCTTCAAAAGGTTCGAGAAATTCACCGTTGCGGTAT
GAATATAGTCCGCGCGGCAGTCCAACAAACAGTTTTTATGCGGGTGCAAAGTTTTCTGAAC
CGCCATCTCCTGCGAGCCTTCCGAAACCACCGAGCCATTGGACTACGAGACTGATGAGCAG
TTGTCAGCAATCCGACAGGATTTGCGATATTTCAAATCATCTGAAGATGATGTTAAACGTCC
AAGCCTGATATATAAAGACCAACAGACCGCTAAAAACGGTTACCAGCTCCTAAAAACAAA
GAGACAGGAAAAGATTCTACAAAGTATAATAACTGTACTTTTCTCAAGGCTGAAATATTTC
AAGTACCTGAATTATATGCATTGTTCTGCATTTCATTGTGATATTGTTGGATTCTTTTTTTCTG
CTGTATTGTAGGTGTGACTTCAGTATTTTTCCAACTAAGATGGCGGA

390. >MSTRG.8252.1 Gene=MSTRG.8252
CCAACGCCATCGCCAAGTTAAAGTGCGCAAAATCCAATATTGCGCGTTTTAGAGTCAAGCA
CTTTCCTCATTCCCGAAGTAATTAATCGAGAACCGAAGCAATGTCTAGTATATGTTATTTAG
TGTACCTGTTGAGAGAGTTGATGGTGCGCCGTGTAATGAGTAGTATGGACGGGTGGTGGTG
GCGCATGAGCATGATGAAGAGCCGCCACAGGACCTCCGGTCGGTGTGGGATATCCTCTGG
ATGTTGCGCCAACTATCGAACCAACCAAACTGTGACCTGGATGGGTTGGGTGAGCTGCTGA
ATAGTGACTATGATGCGTAACCTAT

391. >MSTRG.8277.7 Gene=MSTRG.8277
CAATTATTTTAGAATTACAATTCATTTTTTTACGTTATTTTTCAAGAGAAAGAAAAAACAAG
AAGAAGCATACATTAGACAGAAATATTCGTTTTCATTGCGTACGACAAACAAGATTGATAA
AAAAATTAGAGGATCTATTTCCTCCTTTTTTATCTTTTTTTTTTCTTTTTGCTTTTAGGCATCCT
CAATGTGCATAAATTGTGTTATCGGCGTTTAAATTTTCTTAAGTATAAATTTGTAGCAGTATC
AAAATAGTAAAAATGGTGCATATGAAGACCATGAGCACGGGTAGGACCAAAATGAAACG
GACTGAGCGGCGTAGGCCGCTGGGCTCTTGTCATACAACCTGTTCACCAGACTCGATCGAG
TCGAACAAAGGTCGTTCAGATTGTGGATCGTTGTTCGCCGATGATGCTGTACCTTCACCACC
GCTGGAACTCTCTCTTCTAGTTTTACTGGTGCCAGAAAAGCATAAACAACCGGACTGATTAC
GATTTCTTTTTAACGTTGGCTTATTCTCCCGATCTCGTTCCGCTAAACATTTTTGATAGTAATC
GTAGTCTTTTAAATACCACACCGGAGGCCATCGATGCTCATTTAAATATTCTTGATTCTCTG

GATGTAGAAGACGCACACTTCGTGGAGACATTTTGCACAATTTGTTATAATTCACGCATAA
GTAATTCATACACCAGTCGGCCAGTTGATCGGCATTATGTAACTTGCACGGTTCCAGTAATC
TCAGACAGTTCTCCACGGCCTCGTTTCCCTCGTTCCCAGACAATCTCTCGAGATCTTCGATTA
CTCTGCTCTCGACCAAGTTCACTAGTCGTGGTAAGCAGAGACGATTTGCCAATTCTAAGAG
GTTCAAACATCTGGCAGAGGAAATTGCTGGCACTTCATCCGTATAGAGATAACAAAGCAGT
TTATGAAACGTGTATTCGCGTACACCAGGAAACACTATTACTTTTGCGCTGCTTTCACGAAA
ATCACCGGAGAACATAGCTTTCATCACATCACATCGGGCAGTGAGAATCGCTTTATGGGCT
GGAACACTTCCATCATCTAATTCAAATGTTACATCGGCGAAAAGTCCTTGCTCTAGACAAA
TATCTTCTAAACGTTGCCTCACTACCTGTTTGTAGCGATTATTAAGATCATTATTAAATTGAT
CTCTAGTTTGTAGAGTTCCAAGCACCATCAACAATTGCGGAAGCTCCAAGAATTCCGCTGC
CTGTCTAATTTCTTGTAGATCATGATACCGTTTATCCAAGCTGCCCGTATAAATGAACTGCA
AACATTGCTGCATTGCCTGAGGCGTAATTAACTTAGACAGCGTAACCACTGTCGTTGATTGC
TGCATTCCGTTTGTATTCTCAGTCAAACATACACGAATGTTTTGGAAGGCAGGATGATTAAG
ATCTCTGGTAGTACCAGTCGGTTTCTTTTGGTTGTCCATAGTGGGCAGTACTTGAAAGCTCG
AACGTCGCTTCAATTGTTCCCAAACTTTTGCAGGTTTTGATTGATCGATACGAATCAGGCAC
TCGGTGTCATCGTTAAAATCACCGACCGTTGCTTCACCAAACGTACTTACCATGCTAGATTC
GCTGGAACTTCGAGGTGTTTGCTCTTGAACAAGTTCCATAGAGAAAAGTCGATGAAATGCA
GGCGATGCAGCGGCTAAAAGACACCTATGAGCGGAAAACGTGCAGTTTCCGGCTATTAAA
GTCACGTCAGTGTGAACAGGCTTTGTCCATAGTGTCTTCATATTCTCTTCATAAGTGCTTGCA
GCTAGACACACTTCCGGTGGAACAGGTTTCGGTGGACAAAATGGTGCCTGAAGCAGAGGT
CTCTGCACCCTCTTCAAGTTCGTCATCCAAAATCGTTGCTGACGCCTCGCAATTAAGGCAGC
ACGTATCGAATTTTCGAACACTTCATTAACACCGTAATACGTGAAGACACTGGTCTCGTAAT
AACAAACGCCAAGTTCACGTGCAACGGCTCGAGCTTGATCAGGCATCACCAGATCGCTCTT
TCTCGTGGCTCTCACGAACGGACTTCGATCACGAAAATAGCTCAAATAAGTTTCATCTCGAT
ACATGTAACGCAAATCGTTCTTGCATCCCACTAACAGCACGGGGGTCTGAGGGCAGAACC
GTCGTATCTCTGGGTACCACATCGCCTTGCAATTCCGTAGCGAAACGGGATTGGTTATGGAG
AAACATAGTAGCACGACGTCTGATCTGCCGTACGCGAAACGTCGATCTTTTTCGTGATCAC
CAAATGTGTCCCAAAGTCGCAGGGAGACATTCACATTGTCGACTACTTCCCAGGATCGTTC
CAAGACGTCCTTGTATATCCTGTACTGGTCGATGGCCCAAACGGTGGGCACGTGAGTTGTC
AAAAGTTGCGAAAGTGACACGTGTTTGTTGCAGGCCCTTGCGCATATCAGCCTGGTCTTTCC
AACTGCCGTGTCACCGACGACCACACACTTGACCAGTTCCTGATGCGGCTGCTCATTGTCCA
TCACCGACAATCACCCCTTCTCATCCAATACACCCACCCACGTGCCAGGTCGTTTAAACAC
GCGGCTACCTCCGTTGCTACCGTACCGCCGATCGAATGGCGCAATGTCCACATCCGATCTTC
ACAGAATCAAAGATGATGCCGCGCGTCGATCCTCGCGGTCCCTTTCTCAAGCTAACAATGC
AAATTAGAAGATGATTGATTTATTTGACAAACGATTCTGATACTGGCAGTGCTCTTGTTTTTA
TTTTTCTGTCTTCCAATATATATTCACTCAAGTATCACGCCAACAAATCGGCACAGAAAAAG
ATGTTTCATCAAATGTACACTATCCAATTAATACGAACATTCACGCCGGTTTCTTCGAGCAC
AATGAAACGCGAGAACATGCGACAGTTTCGACGCCCGTACCGTCTATGTCCGCAACGTATT
ACATATACCACTTTGCATAGATAAAACGAATCTTATAAATGCACGAAATCACGTCTATTTA
CACGCACTGATATTTAATGAGACAGTGTCTCTGTTAAAGAAAGGCGAGTCCATAAACTCGT
GTTCCACTAACATTCACGCGAGTTTATAATACGAAATGAAAAACGGGACGAACGAAGTGA
CAAAAGTGAGGTTACGTAGGTTCAAGCTCGATGATACAAGCCTTATTGAGACGCAGTCTGA
TTAGTCGATAGTTTTCACCGCAAGTTTCTTTCTTTTCCTTCTGTCTCTATCGTCTTTCGGATCGT

GACAATGGACTAGAAGCTGCAGCGGCACGCTGCACACCCAGGACGCACCACGCAAGTTCC
AGCTGACGTCCTGGCGAATCGAAGTGGGGAGCAGATGAAGCAGTCGAGCGTGGTAAACAG
CCCTCATGGATTTAGATCGGCCCTCGTCGTTCGACGGTCCATCACGTTTAGTTCGTCTTAATA
TTGATATTTGGTTGAGCAATCTGTGATACTTTTTTTTTCTTTTTAAATATAACACGTATTGAAA
TTTAACCAAACTTTTTTTAATAATTTAGAGATTATGAAGATTGGAATTTCGTGAGATTCTGAT
TGGAAAACAAAATGGGATTAAATCTGGATATCTTTTATAATCCTTTGGCTAATATGGCACCT
ATAAATACATATTGAAAAGATATTAAAGATGGAGATTTATGCTTTGCATACAGTAATTACT
ATTATATTAGAATTTATTTATTTTTTATTTCCAAACGAGTTTCAAAGAGTTTCTTAAAGATTA
AAGAAGATTAATTATCTCAAATTATTCAAAATAAATAAAAAAGATTATGCACTATTATTCA
ACGAAATTTTATTTTGATAAGAAAATGGAATAATAATTTCAAGCAAATAGGAAATGCATGA
TAATTGATGATAGTTAATATCCTAGAAATAGACTCTTAGAAAAATATCCCATCATTTCGACA
ATGCACATGTGCACGTTCAAAGGATCAACTCGATTGTTGACGACGAATTCGCTATCCCTCTA
AATCAGCTGGAGGAAGAGGGAATCAAAGCGCCAGGAAGGGAGAATCATCGAGACAGGAA
AGAAGGGAAATATAGGCATTGAGAAGAGTTCAAGCGAGAGAAAACAACGAGCAATCGAC
CAATCATGCGACATTGAATGAACTCGTCTCTGACTTCGACATGTCTTACTTCCTACCCTTGG
CACAACTACCCCATCATAATCACCCTTTTATCTCTTTCTTTCTTTATTTATCTGTTTTAGAGAG
ATTTTGAATCATGTCGACCATGATCATAATAATTAGCATTTAATTGTAATTATTACAAGATTT
TAAAGTATGATAATGATTTATCTGATGATACAACGTTGCTTGTGAAATGCTTAGTATATTTTT
TTTACTTGATATTCGCACGGATTTAATTTTTGATAAATTAAGATAACAAATATGCGGTTATTG
TCTTTTAAGAATTTAGATTTTTTTCAAATCTAAAATTCTATTTTACAAAATATAATTTATTCTA
TATTCACTTATTAAAATTAATTTTTCCATTTTTTGAATTTAAAAACAAGTATTAACGAGTCAA
TTCTTTCGAATTGTGAAAAAAGGAAGGTGATTTGACTTTGATCATCGAGATTATAGGACTTC
GATAAAGGGATATCAACCGGCAGATTACACAAAAAAAAAAAAAAGTCGAGATGTGGAAAT
AAAAAGCGGTTGGCATCTGTTTCACGCGCATGCTAGCCTCGCTCGTGACGTCAGAGGGTGA
AAAATCAATCCACCCTTGTACAGTGCGGCAAGATAGGATTGTATATCTGGCGGCCACAGTC
GCCATCATTAAATTAACCGAATTAATTGTACTTGTCTAATCGATCGCCCTTGGGCCAATTTTC
AGTTTTAGAATTCTAACGATCGATGATGTTTCGAAGATTTTTCTTGGATTTCTTGGAATGAAC
TTTTAAAAGGAAGATGCAGAAATGATTACGTATTGAAATATTTGACAGTATAAAGAATTTG
TTAAATATTGTAAAGAGACGATGATAGAGAATCTTAATATAGAAAGGATAACTGCTTAAAC
TAGAAAAGGAAAAAGAAAAAATGGATAACTTAATTGTTACTTGATGCAATGTGGCAAAAA
GATTTCCAAGTATGAAGGATTGAAATAGTATTTCTGCTTACATTTGTAAATTCATTTTATGAA
AGTTCTGCAATGAAATTGGCAAAGAGTCTGGGAACTATGGTTACATAATAG

392. >MSTRG.8277.8 Gene=MSTRG.8277

GAAAAAGATGTTTCATCAAATGTACACTATCCAATTAATACGAACATTCACGCCGGTTTCTT
CGAGCACAATGAAACGCGAGAACATGCGACAGTTTCGACGCCCGTACCGTCTATGTCCGC
AACGTATTACATATACCACTTTGCATAGATAAAACGAATCTTATAAATGCACGAAATCACG
TCTATTTACACGCACTGATATTTAATGAGACAGTGTCTCTGTTAAAGAAAGGCGAGTCCATA
AACTCGTGTTCCACTAACATTCACGCGAGTTTATAATACGAAATGAAAAACGGGACGAAC
GAAGTGACAAAAGTGAGGTTACGTAGGTTCAAGCTCGATGATACAAGCCTTATTGAGACGC
AGTCTGATTAGTCGATAGTTTTCACCGCAAGTTTCTTTCTTTTCCTTCTGTCTCTATCGTCTTTC
GGATCGTGACAATGGACTAGAAGCTGCAGCGGCACGCTGCACACCCAGGACGCACCACGC
AAGTTCCAGCTGACGTCCTGGCGAATCGAAGTGGGGAGCAGATGAAGCAGTCGAGCGTGG

TAAACAGCCCTCATGGATTTAGATCGGCCCTCGTCGTTCGACGGTCCATCACGTTTAGTTCG
TCTTAATATTGATATTTGGTTGAGCAATCTGTGATACTTTTTTTTTCTTTTTAAATATAACACG
TATTGAAATTTAACCAAACTTTTTTTAATAATTTAGAGATTATGAAGATTGGAATTTCGTGA
GATTCTGATTGGAAAACAAAATGGGATTAAATCTGGATATCTTTTATAATCCTTTGGCTAAT
ATGGCACCTATAAATACATATTGAAAAGATATTAAAGATGGAGATTTATGCTTTGCATACA
GTAATTACTATTATATTAGAATTTATTTATTTTTTATTTCCAAACGAGTTTCAAAGAGTTTCTT
AGAAATAGACTCTTAGAAAAATATCCCATCATTTCGACAATGCACATGTGCACGTTCAAAG
GATCAACTCGATTGTTGACGACGAATTCGCTATCCCTCTAAATCAGCTGGAGGAAGAGGGA
ATCAAAGCGCCAGGAAGGGAGAATCATCGAGACAGGAAAGAAGGGAAATATAGGCATTG
AGAAGAGTTCAAGCGAGAGAAAACAACGAGCAATCGACCAATCATGCGACATTGAATGA
ACTCGTCTCTGACTTCGACATGTCTTACTTCCTACCCTTGGCACAACTACCCCATCATAATCA
CCCTTTTATCTCTTTCTTTCTTTATTTATCTGTTTTAGAGAGATTTTGAATCATGTCGACCATG
ATCATAATAATTAGCATTTAATTGTAATTATTACAAGATTTTAAAGTATGATAATGATTTATC
TGATGATACAACGTTGCTTGTGAAATGCTTAGTATATTTTTTTTACTTGATATTCGCACGGAT
TTAATTTTTGATAAATTAAGATAACAAATATGCGGTTATTGTCTTTTAAGAATTTAGATTTTT
TTCAAATCTAAAATTCTATTTTACAAAATATAATTTATTCTATATTCACTTATTAAAATTAAT
TTTTCCATTTTTTGAATTTAAAAACAAGTATTAACGAGTCAATTCTTTCGAATTGTGAAAAA
AGGAAGGTGATTTGACTTTGATCATCGAGATTATAGGACTTCGATAAAGGGATATCAACCG
GCAGATTACACAAAAAAAAAAAAAAGTCGAGATGTGGAAATAAAAAGCGGTTGGCATCTGT
TTCACGCGCATGCTAGCCTCGCTCGTGACGTCAGAGGGTGAAAAATCAATCCACCCTTGTA
CAGTGCGGCAAGATAGGATTGTATATCTGGCGGCCACAGTCGCCATCATTAAATTAACCGA
ATTAATTGTACTTGTCTAATCGATCGCCCTTGGGCCAATTTTCAGTTTTAGAATTCTAACGAT
CGATGATGTTTCGAAGATTTTTCTTGGATTTCTTGGAATGAACTTTTAAAAGGAAGATGCAG
AAATGATTACGTATTGAAATATTTGACAGTATAAAGAATTTGTTAAATATTGTAAAGAGAC
GATGATAGAGAATCTTAATATAGAAAGGATAACTGCTTAAACTAGAAAAGGAAAAAGAA
AAAATGGATAACTTAATTGTTACTTGATGCAATGTGGCAAAAAGATTTCCAAGTATGAAGG
ATTGAAATAGTATTTCTGCTTACATTTGTAAATTCATTTTATGAAAGTTCTGCAATGAAATTG
GCAAAGAGTCTGGGAACTATGGTTACAT

393. >MSTRG.8307.1 Gene=MSTRG.8307
AAGTTCTACGAGCGTGTAAACCGAGGCAAAGTATTATTTGAAAAAGAGAAACGATGGTTC
CCTTAACGAAGGGTCGTCGGTTAAGAGTTAGAGAATAGGGAAAAGACGAAGGAACTTGA
AGCGAGGTGGCACAGTATGAAAGCAGTTTACTTCGCTCGAGACGAGAGCAACTCAAAGTT
CTTGAAACCGTTGAAACAATACTTTAACCACTTTGTGTACTTACGCTCTTCGAGCAGGTTGC
TCCTCGTAAATTAGTATAAACTGTTTTGTGTGAAAAGAAAAATAAAAAAAAAAAGAAAGAA
AGAAAAGCAAGGGTGGAAAACAAGGAAAGCTGGGTTTCGGTTGAACTTATCGTTAAAAAA
CGAACATAAAAATGGCACAGCTTTCCTCGTCACCCAAGGGGTACTGTGCCGGAACGCGTTC
CCGTTTTTTTACATGGCGGGGATCCCCAGGAGGCCTACACCATGCCACACGATATTTCCGTG
TGGAACAAATCAAAGAAACAGGGAAAGAGAAAACAAAAAAAAAAAATAAAGGCAAGATA
TATATTATATCGTGGTGTTTGAAAAATAATAAAGAGATCGAGAAGAGAAGTGTATAATCCT
TAAGCCATCAGTCTCGTGGATGTATATCAAATCTCGTAAGAAGTTCATCTTTCTGATTTCGA
AGTTCTTAGATAAATTAATTGTAGTCGTGCGGTCGATTTCCGGAGCGAGGGACGTTTGAACG
TTATCGCAATGACCCACGAATACCGATCGTGTTTGCAAACTCCATTTACGAGGCGAAAGTG

TTTCTCGAACCACACTAATGATCCGTCGTCGCGTATTTTTCCTGCTCGCGTTCGATTACGTGC
CCACGTGCAACTGCACTTTCTACGGCGTCCTAATCGGGGAATGGACCGATCGAACGAGTCG
TTTAATCGTTCCACCGATTTACTTTTGTCCATCGAATAGATTATCGGTATTCGAAGAGGCCA
CGCGATAACGATACGCTCTTCGAGGCGTCGGGATATATCATCTCCCTTTGATCGAAGCGAA
GAATGAATTTTCTAAAACGCATGAAAAATGAGAAAACATCGAATGAATTAAAAGAGAGAA
AGAGAGAAAAAATAAGAAAGAAAAATTTAGAAAAATCGATATTAATTTTAAACGACGCCT
CGATAGAGCTTTTTCGAAAAGGGGTGAAAAACACGGAAATCGTCCGTGTGACCACGGGAT
GGGTGTGAAACTTATCGCTAAACACCGGCATACGCACTATGCCGGTGCGTGGGTACCTTAT
ACGCTAATCCACTTAATCGACTACCTGCTTGTATACCTCTTCGATGGAACAGAGAGAAAGA
TCGGAAAAAAACGGAGAAGAAGAGCCACTAACACGTTCGAGGCCGAGGTGCGCGACACA
CCGATCACCGGAACTCAAAGAGGTGGCCACGTTACGGAAACAGGATCTTTTATACCCTGGA
AATTATTGATTTTCATCGGTCCGCCAGGCCGGTGGCACGTG

394. >MSTRG.8308.2 Gene=MSTRG.8308
CCTGTTTCCGTAACGTGGCCACCTCTTTGAGTTCCGGTGATCGGTGTGTCGCGCACCTCGGC
CTCGAACGTGTTAGTGGCTCTTCTTCTCCGTTTTTTTCCGATCTTTCTCTCTGTTCCATCGAAG
AGGTATACAAGCAGGTAGTCGATTAAGTGGATTAGCGTATAAGGTACCCACGCACCGGCA
TAGTGCGTATGCCGGTGTTTAGCGATAAGTTTCACACCCATCCCGTGGTCACACGGACGATT
TCCGTGTTTTTCACCCCTTTTCGAAAAACGTATCGTTATCGCGTGGCCTCTTCGAATACCGAT
AATCTATTCGATGGACAAAAGTAAATCGGTGGAACGATTAAACGACTCGTTCGATCGGTCC
ATTCCCCGATTAGGACGCCGTAGAAAGTGCAGTTGCACGTGGGCACGTAATCGAACGCGA
GCAGGAAAAATACGCGACGACGGATCATTAGTGTGGTTCGAGAAACACTTTCGCCTCGTAA
ATGGAGTTTGCAAACACGATCGGTATTCGTGGGTCATTGCGATAACGTTCAAACGTCCCTC
GCTCCGGAAATCGACCGCACGACTACAATTAATTTATCTAAGAACTTCGAAATCAGAAAGA
TGAACTTCTTACGAGATTTGATATACATCCACGAGACTGATGGCTTAAGGATTATACACTTC
TCTTCTCGATCTCTTTATTATTTTTCAAACACCACGATATAATATATATCTTGCCTTTATTTTTT
TTTTTGTTTTCTCTTTCCCTGTTTCTTTGATTTGTTCCACACGGAAATATCGTGTGGCATGGTG
TAGGCCTCCTGGGGATCCCCGCCATGTAAAAAAACGGGAACGCG

395. >MSTRG.8314.1 Gene=MSTRG.8314
GAATCGCAAGAGGACCTGTAGTCGTCTTCTCGAGGACATCGCGTCCACGTCTATGATCTGA
AGAAAATAAATTGTTCGTCCGATCAGGGATTAGGGAAAAGAAGGAAAAAGTGAAATTGGG
TTGGAAATTGATTCGAAGGAAGTTCGAATTAGAAAACACAACCGTGATAATAATTTTCCAG
TAATCAAAGAACACTTATGTGTCGATGAGGTGCAAGATTTTGGATTGTCAAGGAAATTCCC
CAACATTCTTACCCAGTGTTAGGCTCGCTAAAAATCTTCGAACTCGCAAAGGGAGTCGACG
TGGTAACGCTGGAAATCGTCTAGAATTGTTGGTTCTGGTTCATTTGCATCACGTGCCATAAA
ATAAAAAAAGAGATCAGTGAATCTGATAGTGTCAGAAATTAAAGTGAAAGTGCTGCCGTA
TCGGTTTTGATTGGCTTAAAATCGACAGAAAAGCTAGAAGTAGAAGGAAGATCGAACAAT
TTTCATTTAGAAAAGGGAAACGAAAAAGAGGTTCAAAAATGGTTCACACCGATATTCTCAT
CAAAGGGCCTTACGAAGATACGAGAAAGAACAAAAATGCATCGTCAGCACACCAGCAAC
AGCGACCTCAACAATCGGAGAACAAGAATGACAAGTATGGCTCTTGGGGTCCAGTGTATA
AGAACAAACAAAATTTCATCGACATGCATATTTGCTGATTACTGATAATGAGGTATAAATT
GAAAGATCGTATTGAGGATAATGAATAACTACGAATAAAAAAAAGTTTATATACATTTCT

CGCATCACTTTATCACTTTGATTTCGACACCAAAGATTTTTACTCTTTGTTAAAATTTGATAA
TTAGTAACAATAAAAATGAATAGAAGTAATTAGAAGCAATCAAAACGCTTGAGATTGATT
ATTCTTTAACCGATTGTTCAAAACAATTTCCATTGAAGAGGGAAAAAATGAATCTAATAAT
CTCTGAAA

396. >MSTRG.8328.1 Gene=MSTRG.8328
ATTATAATATATTCTATATTTTTATTATGAAATAATAACTGGTTTTAATATTCAAACAATTCG
CGGGAAAAGATTCAGATTTCATAATCAGAATTCTCTAGTATCTAATACTTAATACATTGAGA
AAATGTCGTCTCTTGAAGCAGGTCCAAGACCAGTACAAGTATCTCCTTTAATTAAATTTTCT
CGATGGACTTTCTTGTTAACTGGAATTCTATATGGTGCTTATCATCAAAAAAGACTTAGTAA
AAGAGAAAATGCTCTTCGTGAGCAAGAACTTAAAGAAAAACCTATTAGGGATGCAAAATT
AGCAGAAGAGAAAAAGAAGTTATTGGAAGCTGAAGCAAAAATGATAGAAGAATGGTCAG
GAATAAAAAAATAAAAAATAATACATTTTCAGTAGTATAAATGTGTATTTTTTTTAAAATTA
ATATTATATCTTTA

397. >MSTRG.8342.9 Gene=MSTRG.8342
GGTGGGCAGGCGATACGAGGAAAAGTTTATAGGTTTTACGTAAGCTTTTGAGTCGTTGGAG
GATATTTAGCCGCGGCGACTCGTCCCGTACGGTCAAATCGAATCGGCGAAACGTCTCGGTT
GGGTGGTGTGTGTTGTGATCGTGGGGAGAGAAAAACGGAGTGTGACGAATTCCAATCGAG
ATCCATAATAAGTATGAGCTTGCACGATAAGTGATTTTTCTTCGTATATATTGTTGATCGTAT
ATCGTAGGTGAGAAAGAAAATTTCTTCTTCTTCTTCTTTTTTTTTCGTTTATATTTTTATAT
ACGAGTGTCATTGCACGTGATTTCTTCTTTTCGTCGCTTTTATTCTATATATAGGTAAATGAA
TCGAGAAAAGAAATTTATTTTTTAAATATATTGCTATTCAACAAATGGGATAGGAGATATTA
TTCTTGGAAGTTTTTTTTTTCTTTTTTTTTAATCGTTTTGCAATATGATTTACCGTTATCGAAGT
TTTTAAGGACGGTCCGTGGACATTGCTTTTAACATTGCGAGGATGGCACGCAAACGCCATT
ATTATTTCTGAGATAGCCTTGCTCGCATCGGCAACCTCCGGTCAACGACCACGTGCATTCGA
TTCTCGGGCAAAATATACGATTCGGATGTGGAGCGTTGCAAGATGGCTCGCACAACGATCC
ACATATGTACGGTTTTTCATTTTCTCCGCATCTTATGTTTTCCTCGTTGTTTTGCGCGTCGACG
TAAACAGCGAGGAGTGTAGCAATAAGCATACACGTAAAGATGTAGCGGGACATTGGAATG
TATTCTTCTTTCAAAGCCGTTTTCGCTTCTACTGTCGGAAG

398. >MSTRG.8351.1 Gene=MSTRG.8351
GCCGAATAAATGGAATATAAAGGCCGGCCGTTTTTCACAAATTTCAAAAAAGTAGTTACTT
TTATTATTTTTCCGCTCGAGAAACTGTGATAGAATATCCTGCTTCAAAAATGTCCCGTGCCA
TACTCTTTGTCCTCCTTGTCGTTGCTTATATCTGTATAAGCAACGTGAACGCTTGTGGACAAA
ACGAACAATTTAAAGAATGCGGAGGTTGCGACCAAAGATGTGGAGTCGAAAGAATCGAAT
GTCTCGAGGTGTGTCAACCCGGCTGCGCTTGTATCAAAGGATTCATAAGGAATGCCGCAAA
CAAATGTGTCTTACCTAAACATTGTTAACATCCCTTGTTGCGAATCGATTGAAACAAATGTT
ATTCTTCAAAATATAAGAATATAAGATATATAAGATATTCTTCAAATATAGGAGTGTTCGA
AAAAAAATAAAATGTAATGATTAAACACGTGTAAATTTATTTTTATTTATTTCTTTTCTTTTTT
TTTTTTTTTTTTACATTCGTAATATTTTACACGATTTGTGAGGCAAGGATAATACGTAAATTT
GCATATATAATCCTTTCCAAAGATTTTTACGCATACTCGCGACTTATTTTCGAAAGATATTA
ACCCGTGTTTGCGTTTATCGTGTGTTAAACGATGTATCGAGTACACGATAATGTAACGCGTT

TCTTTCGATTCGTATGTACTTCGATGAATAACAAATGAATTAAGAAAACGAATAAAAGTTTT
GAAACGATAGTTTATTATTTATTGTTTATATATATATATATATACATGTTTGTTTTGAATTAA
AAGAAGATAACACTGCAAAGCAAACAAGAAAACAAAGAAACAAAAAAAGCGAAAGAAA
ACGAAAGAAAGCGAATTCCAATTAAACGGGGAATTTTAACAATTTTCTGGAAGGACACAT
GCCCCTTCGCCATTTCTCAAGAATCCTTCCTGACACTGACAACCGATACGACATTGCATGGT
ACAAATACGCGTTTTTGGCTGGGCACATGTGGGTGCGCACGCCGATCCACACGTATTGAAC
ACCTCGTTTGGTCCACACTCCTCGCCAAATGAACTGGAGAAAATCGCCATAGCGGCGAGCA
AAATGAAGATGATTCGAGACATATTTGATTCGAGACTTTTTCGAACGATCAACGACTGAAA
CTACTAAACGTATCGAACTTCTCAGCTTTATATAGTGACCTTACGCCACGCTTGCACCAAGA
CGAATTTTACTCCAACAA

399. >MSTRG.8401.1 Gene=MSTRG.8401
CACTAGCACATTTTTGCATAAGCTTAGCTTGTAATTTAAATTTTAGGATAGCTACTAACTCGT
ACTCTTTTTTTATATAATTACAAAAGCGAATAATCAAGCGTTATTCTTGGGCAACTATTGGT
ATAAAAAAATACGTAAATTTTATCTCTATTTCAATCGTTTTGTAATGACAGGAAGAATAGTA
AAAACACTCGTAAAGCCTTTGTCGATTGATTTCGATTAATCACTAAATTGTCATAATGATGT
TTCGCTGGCACCAGAACTCTGTGATTGAAGTGGATAATAATTTACTGCGCAGAATCATGCG
CAGTTCAATATTGAAAAGACAAGATGGCGATACGGAAAAGGCATCGTGGGATACGCAATA
AGAATGGCGTCGTTCAAAGTAATGAGAAAAATAAGAACGTTCGTAAATAAAATGCCTGGT
AAACGTTTCAAGGAATTTAGATTTTTACCGTTATGTTTTGTCATGGGTGGTGTGCTAGAATTC
CTTATGATTCATTGGCATGTTGGAGAGGTTAACTTTTATAAAACGTATAAAAAGAGAAGAA
TAGAAGAAGCAGTTGAACAGAGATTAAAAGAAATATATTAGTGAATAAATTAATTATAGC
AAATATTGTCTTCAAATTAATGGACGAAAGTGAAATTTGTATCTATGAAATTAGTCTTCAGA
ATAATGTTGATATAATTTATGAAGATACGAAGGATTTATGTAAAAACATATATGAAGTATA
ATAAATTATTTTACAAATGTTTAAGTACAATTTTGGCAGTTCTTCTTAACTTCTTTCATATCA
CACACCACATTTATGCTTGTTCATACCTGAGCAGCATGTCTTTCCACCATACCCAACATTTG
TTGTTCCAATTTTTTTAATCTGGATTGATATTTTTTCTTAGACTTCTCTAAAGTTTGTACCAAG
GCTCTAAAAATAAAATTAGAAGGTAATATTTTTCGATCAATAAAATTAATTTTTTTAATCCA
TGATTTAATTACCCATTGGCTCTTTTTAAATCGTTAATAAGCTCAGCTGATTGTTGATGCCTC
AATTCAGAATTTTTATTAACTTTATCTAAAGTAGAAACTAATTCTTGCAATCGTTCTTTCAGT
CTAGCCTCTCGACCTAATGCTTCTATTAATTCGGATTCCATGCCTTCACTCTCGTTATCGCTT
AAACCTCGATCTTTATTCGTAGCCATTGCCAGCATGGCCTCTGTATCTCTGAGTTGACCCTG
AAGATGTTCAATGGCGGCATGCTGAGCTGCAATTTGCGTATCACGTGCATTTAATTTCAATT
CTATAGTAGCTCGTTCCTTTTCCAACAGAAAAACTCGTGCTCGTAACTCTGCCTTATCCTCTC
TCATAGCCATTAATTCCTGCATAAGTACTGCTGTTTCTAAATCTAATTTTCGAGCTTCGGCGA
GAGAAATCGTGTTTTTCGAGTTTAGTGGCTCAGCGTGAACGCTTTCTAATTCCACCACAGTT
GATCTAACCATAGCTCGTTCCCCTTTCAGTCTGCTAATGTGTCTTCTAAGTCTTTGCTCATCTT
CAACGCACCAAGTCACTTCGGATCTAAGGAACACGAAATCGGATCGTGAAAAATAGTGTA
AATTTATCCATTATTGCTATTTTTTTTATATTTACTCGTCCGACAAAACTATACTATCTTCCCA
C

400. >MSTRG.8421.1 Gene=MSTRG.8421

GAACAGTTTATCGCTCAGTCGTCAGTCTGCAGTTGTGATGCGTGTGTCGTGCGCGTGTTTTTT
TTCCCTTTCCAAACAATTCTAACTCGTGAAATAAATGAATCGCGTGAGAAAAATATGAACA
TTTGTCATGAATTACTCGAGATCGTTTATCAATTTTAATAATCGTTCAGTGAATTAGATCACA
TTCTTCCACTTATTCTCTATGGCGTGGCGGATTTTAGAGAATGTTTGAAATTCTGATCAAATC
GAACGAATCACAGTTTCTTTATAGAAAGAATTTTTTATTATTGATATAATTATTCGACGAAC
ATGTGGGAAAGAATTGTGCAAGAGCGCAAGAAAAGCACGTGAAAAAGCATAAAACAACA
TGCATTATTTTACTCTATGTATATACATTCCATTCATATTCATTTCTGGTGACGGTTCGTATTT
CGATCCTTGGAGATCGTTTCTTGTGATTACCGTGCATTTCTCTCTTCATCAACTATTTATTAAT
CAGTTGAACTTTAAATATTTTTTCGGAATCTATTTGAAATGAGAGTGTGGTGTTACTATAAG
ATTTTCAAAATATTCCAACGTTATTCGAAGATCATAATAAACATCAGATGACTGGAGACTA
TAATATTAATAAGTGTGAAAGAATATCAATGGACGCTAGTGTCAAATAGAAGTTAAAGGA
ATATGCACTGCGTCAAAGGAATTCAATATGGCTATCAATAAATTCATAACAACGGAGGACC
TGCGATACATCGGAAGTTAATCCTGTCTTGTTCATAAGTACTAGTGCCGCAGGAGTGACTAG
GTTGGGTTAGAAATTACCATATCTCCTGCTGCTCAAGTGCTTATCAATGGGTCCGGACTCAC
ATCTATTTCTCAAAATTATGTATTGAGACTTTTTATCTAAAGTAAAATTTTGTCATAATACCT
AAAAATTCAAGATTGTAATTTGCGTATGTTAATGTAGTATTGCAATTTTTACTCCATATTTAT
AATAATTTTTTCTATACAAATATATTAGAAGTATAAATAAGTAAAAGAGAAAAAACACTAT
GCATAAATGTATTCAATACATGATAAAATATTGATATTGAAGAATAAAAAAAGTATTTATT
GGAGGTACTGGGATTTGAACCCAGGACTTTCAGCATGCGAAGCAGACACTCTACCACTGAG
TTACACCCCCAGTTGTATGTTGTGTGTTTTATATAAATTAAATGGTACAATTTTATT

401. >MSTRG.8423.2 Gene=MSTRG.8423
ATTTAGGAGATAGTGTGGTTATGTTTGGCAGATGCGAACTTCTGTCAACATAGCAAGAAAA
TGGGCTTGTTTGGCAAATCTCCAGAAAAAAATCCGAAAGAAATGGTTAGAATCGAAATGA
ATAATAATATCTATTCGATGTTTATACGAAATCGTATTTCAGGTTCAAGAATGGACACATAA
ATTAAGGAAAGAGGGTTATCAATTGGATAGACAGGTTAGAGCAATTCAACGAGAAGAAGA
TAAAATAAAACGTTCTTTGAAAGAAGCAGCGAAGAAAGGTGATAAAGATGTCTGTAAAAT
TCTAGCAAAAGAAATAATACGTGCACGAAAAACTTGTAACAAACTCTATACATCAAAG

402. >MSTRG.8436.1 Gene=MSTRG.8436
AAGACGAAAATTAATAGATAAGATAAAGCGAGGAAGAGAAAGTAAGATGTTTAAAGAGA
TGCAAAGAGAATTTGTTTACTTCCCGTTTTCGACTCGAGTCCACTTAATGGTAGGTAAGTAA
CTTACTTTCCCAGCCTATTCCCCCCACTCCTCCTCTCTCAACTTTTTTTACATTGCAGTTGCAG
ACCGCTGCCCGATCTTCGAAACCCCGCTACTTGATCTCAGTCTGGTGCGCACCGCCACCGA
GTGAGGGTGTTTAACGTTTTGTTTCGTTCGAAACGACAAATCGACCTAGAGAGATCAACAG
GCAAAATGTTGAAATCAGCCTCGCCGCAATATCCCAACAGAGGATCCGATGCCCGGGTCAT
CCGTTTGACCACGGATCAAGAAGCTGTCCTCCAGGAACAGTTCAATAGATGGCCGAGGGC
ACCTCATACAGCGGATATCGTCCTATTGGCGGCCGAGACAGGACTTTCTGAAGCCGATGTC
GAGGCCTGGTACTCTATCCGATTAGCACAGTGGAGGAAGGAACAGGGCCTGGGAGGAAAT
CTTGGTTTGCATTAACCCTTGACCCTTTTCTCGAATGTTATCGAATGTAACTGTGTACCTGAA
AATGTAATACGTGTACAGGATTGAACCTGATAACGATCAATTTCTTAATCTCGATCTCTCAT
TGCTTCTAGTTTAGCGATTATATACTCGTGTATTTTATTAATTAATGTATACGCGATTTATTTA
TATTGTCAATGTGTAGATGATTGTTAGAATGATTAAAAAGCGATGGTACCAAAAATTTTTCA

CTTTAAGAAGTAATCGCGGGTAGGAAAAGATGTAACAAGGAAATTAATTAACGTAGGTGT
TTTCCGATTCTGATTTTATCATTATTTATCTCGACACTAAAGTAGAAGGATATTAAAGCGCTC
GAGTAAAAATCTTTTTCCAAGTGTGTCCCGACATTTTTTTTTTTTTTT

403. >MSTRG.8447.1 Gene=MSTRG.8447
AAAGTAATCGTTCGATCGATCATTCTCTCCAGTCGTTTCGGTCATTCCATGATCATTCCGTAT
TTGTTTCTAAAAAAAAAAAAAATCTTTTTCTACCCAGCCTGCGTCTCAACGGGGCACAGACG
ATCCGCTGGAAGAACGATGGATGCTCGAACGATCTTTCGATTCTTTTCGCGAATGCGCGAT
ATATTTCTCGCATGCCCGATTCTCATCGGAGAAGGATGAACGAAACAACGCGTGAAAAAA
CGTGGTCGATGCCGTGCGCGCCATTTCTTTTTTTTTTTCTCGAAAATCTCGATAACTTTCCCTT
ATTTTCTCTTTCGTTGGACCGATAATGTAATTAGCGTTAGTGTTTACCATCGGCAAACAGGG
CATTCACATCGTATATCGAATCGAGAAGTTGAACACGAGTGACGAGGAGTTTAACGAGTGA
GTCGCGTCTAGAGGCGAAGATTAAACACATTCCGCGGAAGATCTTCGTTCCGCAGCTCTTCT
CTTTTATATTATAACAATAATAATAATAATAATAATAATAATAATAATTATAATTATAATAA
TATGTCCGATATGCGGACGTTCGCGCGTCTCTTCGCGCTGCTTTGCCACGTCGAAGGCGCGA
GACCATCGTTTAAATAACTTGTATAATCTAATTTGGGCATAAAATGGCGCGAACACCGGCA
CGCGGTGTTTGCCCGTGCGTCGAATAGAGATCAACCTTGTACATATAGACCGCGACATATA
GACATTATGCATCACTTTCTGTAAAACTGACACGTATACACGTACACATCAAATCACCACA
CAAATGCTGCGATTACGACACGATTACTAACCTTACCGGTACAACACTGTTTTTTCCTTCA
TCGAGTTTTCAACGATTACGGTGATATTTTCTTTAGGGAAAAAAAAAAAAAAAAAATTCATC
GAATCGCGAAACAAGATCTTTTCAGCCATATTCTTTTTCGTCGAAAAGTTTGCTCGATACTC
GTTCGATGCGATAGATCGAACGAATGTCGAATAACGTTTGACGAGAACGCCCTCTTTCTTCT
TTTAGATAGAAACTCGGGCAAAGAAAGATCGAAGAAGAAGAAAAAAAAAAAAAAGATAA
ATATAATTGCAACGGTCCCCTGGTCCCTAAGCACTCGTTGAACACTCGTCTAATCGCAACA
GAGCCAGTCTTAAATGTCTTATTAAAGCGGCGCGCAGAGGTCACGCGCGCGTTTACGCGGC
GTGTATATGCGCCGTTAATTAATCTCGTTGACGATTATTAATAATCGCGAGTCCGCTAGGAG
CAATCGAGCAAACGGCGTCGCGTTCGATCGTAACTCTGGGGAGGGAGGAGGGCCCTTTGA
GTTCGTCCACGGTAACCCTACGTCCACGTGCGAGCGAAACCACCGGCCCTTCATTCGCTAT
ACTAGGCAGCCATTCTCTATTTCACTTTTAAAGGTCGATCGCTCGCGCGATTTTTCTTTCGTT
TCGAACACTTTTCCTTTTCACCCGTTTCGCGATGCAGGAGAGCTCGTTGGAGGAATGTAAGC
GGGATCGATTCATTCAGACGATACACACAACATAACCTGTATCTTTCACGATACACACGAT
ACACGTATACATAAACGAATACATACACATGCTCGCGACAAATACACACGGTACACGGTT
TATTATTTGTAAAAGATTCGCGAAACAATACGTATATATTTACGAAACACATACTTGTACCA
CAATTGTAATACATCGATAGACCAATCTTTCGAGAAGTAAGACGCAGTTACGAATGCGCTT
GACCAATGTATCGCGTGCGAGATATATCGATATGCTTCGATCGAACGATGAGAACAGTCGA
ACGCGAATTGTATAGAAAGTTGACGTACGTAATCGTGGCGCGATTAGGATCGTCGTCGGGA
GAGATTAAAGTCGTGTTTTCTTTCTTTTTTTTTTTTTCTTTTTTAAATTGAATTTCAAAATCGTAT
AATTTTTTTTTTTTTTTTTATTGATCGATCGATTCACGGTATTATGTATACGGGGAAGAGAGGA
TATTCTGAAGTCGATGGTCCGCGATCGTCGATTCGTTCGAGGTATAAAAAAAAAAAAAAAA
AAAAAAAAAAAAACGATCGTATTCACCAGCGATGACTGCGTGCACCAATCGTCGGCAAATT
TATTCGATAATTGTTAAATTATAATCCGTGGATCCGTCAGCCAATTGAAATTTATCGGCGAT
CGCTCGCCATATTTCGATTTTTTATTAAACCGATAACAAAGAACCAACCGTCTGCCGCGAGT
GATCGCCGATAAAGAGAATTTTACGGTATTCGGGGGAGGCAAACATTGATGCATGAGTCCT

CGATGGTCTAACGGAAGCGGCTGTAAATGCGGTTTGAACGGTTGAGATCGAAGCTTGATCG
AGAGAGAGAGAGAGAAAAAAAAAAAGACATACTTTGGGCATTCTTTTGAATCGCGTTCGAA
TGCGAATTGCGAATGAATGAGAGTGGCGAGTGACGAACAGATCGCAAGTACGTGGTTCGC
AACTTTGAAACATGTGATACGTCAGTCGGCAGAATTGACTTCCGGTTATCGGGAACGATCG
GGAGAAGGGAGAAGGGATGATTGCGAATGGAAAAAGAGGAAACGTAAGCAGGAAACGG
AACAACGTAAGCATACATACTGGCAGTGGATTCGCTGGCTGACGCATTTTTTAAAGATTCTT
TGAAGTAGGAACGCTCTTTTGTTGAAAGGTTGATTGTTATCAAAATGGTTGTGAGGTTATAA
TGTTTGAAATGTGTTAATGATGCGACGCGAAAGTTCGATCGACCGATCGACACGACTTCCG
GAAACGAAATCGATTTGTTCGATTAATCGATTATCGATCGAAAGAAATTTTTTTTCTTTTTTT
CCCCCCTCCACGTTTCGAGAAGCCGTGTCGATCGTCTCGCCGAATAGCAGACGAGGAGACG
AGTAAGGAAAACGTGCGTTCGCGTTCCAAATTTTTTTTTTTCTTCTTTTTTCATTCGATTTCGC
GAACATTTCTTTTAATCGAAGTTACATTTTTTTCAACATTACGTATTATATATTTGCTATTTAT
GAACAGAATATACGAATATACGGTCGATCGGCCGTAGCATTTATTTCTCTTTACGTTTAAAT
TGAATTCGATTGGCGGGAGCGCACGTGTTCCCCCAATATTGTGAAATTCGTCTCCGAGGAA
GCGTGCAACTGGCATTACCCCGCATAAAGTCTGGTTCGAATTGTGTGTTCAATTTACGACAA
ATCGATGATGTTTTCCCGCGCTGTTTTCCCTTTCGATTCGAATAGGTTTAAAGTAAAAAAAA
GTGGAAGATCTCGTGCATTAAGAGGGAGTTAAGTAACGACAGGTAAGACATCCTCGAATT
GTTGTACGGAAGATGATCAACCAGGCCGATGTTGAAAAATGTGCGACTGATATAAACCAGT
ATTCGTGCGAGAAATAATAAATTGTAAAATAAAATTCGCGTAACCTAGCCTTAATGAGAAA
GATTAAAAATTAGGTGTAATAGAGGCGGTGCCGCGAATAATGGCGGCCTCGCGTCGAAATT
ATCTCGATTGGCTGTAAAATATTATCGTGGGAGATGGGAGCGACGGATTCGACGTTTGGAG
ACAAACGAAATTTCTCGAATTTTCTCTTCGAGAAGAAAAGATAAATCGATAACCGAAAGA
AAGAAACGAAAACGTCGCTCGAACGTCGTTCCCCTTCGCACGGTGATGTATCTAGTGAGAG
TAATGTTACTAGGTAACTTAAGCGTATTTTAAAAAAGAAAAAAAAGAAAAAAAGAGAAAA
AAGTATAACGAAATGCCAGTTGTAGTAAAAAAAGTTTTTCTTTTCGAGAAAAGGAAAGAG
AAAAAAGAGAAAAAAAAGACACAAAAAAAAAAGAAAGAAAGAAGAAGAAGAAAAAAA
AAAAAGAAACACACGCCGGAAAAAAAGTACGTGGGAAGGCATGTTCGATAAAAGGGAGA
ACATGTCTCGAGCGACGCGTCCATTCATTGCCGGGAAACGAAACTGTGCAACGAATTGGCA
ACACGAGTAGTAGAAGAAATAGAAAATTGATGGAACAGAAAGATATGAAATTTTCGTTTA
TTCGGTACGTTCGGCGAAAGGAAAGTTTCCCGCAAAACGGCAAAGAGATCGTATTTGGTCG
CGCGATGTCGTGGTACGATCGGTTTTTCGTTTTTCTTTCTTTTTGATTATTTCGGACCGCCCGA
AGGGAACGATCACAAGGGATGTTCCGCGAAACATGAAAATAGAGTGAACTGCTAAGGTGA
TAGAAGGGTAATAAAGGGTGTATACAATATAATAAACGGAAGCGAAAGAAAGAAAGGAT
AATGGCAGAGAAAAAAAAAGATGTTAATAAATATACATATATGTATGTATGGGCAACGGAA
CATTGTCACATGTGTATACGCGAATTTACAAATTTTTTCAAGAAACGACAGCTTTACCGATA
GATTAAACAATAGATTTTTCGCAACTTGGGCACACGTAAAGAAAACAAACACCTCAAACA
ACATGACACACGATTAACACATTACATACAATACGCATTACATGTAAAGCGATCCATGCGA
AACAAAAAAAAAAAAAAAAAAAA

404. >MSTRG.8488.2 Gene=MSTRG.8488
CTATTATGTTAGTGAGAATGCAGCTTAACATAATTTGTTTAATTATCAATAAAATTATTTTTA
AAGAATTTTGAATAAACAATTTCTATCTTATTCTAACAATATGGATGAAGAAATGCGTACTC
TTTGGTGTGGAAATTTAAGTGAAAAAGTGACAGAAGAAATTCTATATGAATTATTTTTACAG

AAATTTGAGAGTTATTCAAGACACAACATATATACACCATATTTTGCGAGATTATCATCCA
AGATATATTGTAATTGTACAGTTGAAAAATAAGTATAAGGAAAACAAATAATGGAAAAAG
GACAAAGATAAAATAAAAATAGTAGAATTAATATTATTTCTAGAATGTTGTAAATGTATAA
TGGTATAAACATACAAATAAAATAGACAAAGATGACAGG

405. >MSTRG.8504.1 Gene=MSTRG.8504
GTTTTTTAAAATATCCAAGATAATTTGACGGTGACAAAAATTTGGCACGAGTCGTTGTTCCA
CTTTCCTCGTCGCATCTTTCATCCCCGAGACGAGGATCGCGTTGCGAAAAATAATGGAGAG
TCGCGAAAATTCTACTTTCATCGTGTCAGCATCGGGCAAAAAGAGGCGCGTACGCGCGCGC
GCGAGAGTTTCACCCGTTTTACCTCGCTCTCTCTTTCCTGTTCCCCCTGTACTTGTGCTAATCC
TCGTCTTCGTCTTATTGTTTTTCAGCCGGTTTGCCGACGCAGCGAAACGATTCCGCAAAGGT
AGCCCTCCGCGCCTCCGGTTCGTCGTACCTCTCGCGTTTCCACGTATCCAGCCACGCGTTTG
CCTGTGTCGCGCGTGCCTGCCACTCACACAACGGGCGCCGCGCCCGTACGTGTAACTGCGT
ACGTGCGCTTATAAGTAGATACGTGTGGGACGAACGGGCCCCAGTTAGCATAACCTGATCT
CGAAGGCCTCTCTCGGGTATCCTCCCTTCACTCTCTTTGCCTTTTCTTTTTCCTTCCCTTTTCCT
CTCTCTCCTTTTCCTGCATCGATCCTGCAGCACCTCTTCCACCTTCTCTTTTCGTTTTCTTTCTC
TTTCTTTCTTCCCGTTTTATCCGTATATAACGCGCATGCGCGGTTGTGTGTGTATATATATATT
TCTCCAAGGGAGAGATACGGGGACGAGCAAAAAAACGAATGGACAATAATGGTATAAAC
GTTTACGACGTAGGAGAGGACGTTCAGAGGACGCCACGGATGCGTAATTTCGCGACCTCGT
GCGGGAGAAAGGAAAAACTAAGATCCGTATTCTAATTAAGGTTAAGGATAATCTTCGCGTC
GTAAGATCCAACTGGTTCCACATCTCTCTTCTAGCGGAGTGTTCGAGTTAAGATAGTAATTC
TAACATGATACAAATGATTGGAGGAAACAGTGTGATCGTGACGAATAACGATGGGACTAA
GATCGTCCAATCCGGTCGCACTTCCGACGGAAAACCTTATATCCGTGAAAGCACGGACAAA
ATTATAGGCGACACTCTCCGACATATTGAAAGAATTTACGATCCCATTACTAATACTTCAAA
GATGCACGGGTATACGTTGAATTTAAAGGATCCCTCGGCCAAACCTGTTCCGTTAAACGAT
ACTGTGTAAACGATGCCAAGGAAAAATGATGCTTAATCCTTATATTTAACAATAATCATTTA
TCTATCTATAATATTATATTTCCATGTATATGTAAAGGACGTAATAAATGAGTGAATAATCA
A

406. >MSTRG.8539.2 Gene=MSTRG.8539
ATTTATTCACTTACCTTATATTACTAATCTTGGTTATATTTTGAAATATTTTTTATAACAATTC
TTATAATTATAAAAACGTAATTAAACCATAGGGTATATAAATTATAATGAAGGGTATATGA
ATCTTTTACTTAAATAGATTAGAAGAAAGATGAAACCAAACGCAAAATTGCTTCGTTCGCT
CATACAAGAAATTCGACGAACGTCATCAGAACAAAAAGCAAAAAATAATATTATGATACA
GTATGTTTTGGAACAAGCAAAATCTCATCGAGAAACTTCTGAAGTCTTGTGCAAAGCACGA

407. >MSTRG.8595.1 Gene=MSTRG.8595
ATACCTTTGTTTATGGGGACGACGTCCGGATTTCTGTCGTCGGTATCGTACAAATCCTCGGA
ACTAGGAACGGCACTCTTCTTTAACGGTAGATCGCTCGGCCTCTGAGCCCTCCTCTCGTTTC
TTATCTTCATAGCTACCAGAGTAGCTATGGTAACGAAAAGTAACGCGGTCACTACACCGAT
CAGCCCTCCTAATAACGGAGTGATCTCAAACGGTACCGGTATACGTTTCGTCTCGGGATTGC
CAGCCAGACTCATCAGTAGGGCTACGACTGACAACCCCTGCCCTAGACGTTGACGGAAAA
TTGTCGAAATTCGTTAAAGAAATTTGTCGTAAATTCGCATTCGTTGGCAGAAGAGAGACGA

CGAGGAAGGAGAGAGAATTTCCTCTCGACGTCTCGTTGTCATCAATTCGTCGCGTCACGTCC
GGATCCCGGGGAACACGGGTTCTCTCAGAACGCTAGGTTTCTCTTGCTCTTAATCGCTAACC
GTTCACTCGAGATACGATGGTGCACGAAAAACACGTTTCGTCTCGGGATTGTCAGCCAGAC
TCATCAGTAGGGCTACGACTGACAAACCCTGCCCTAGACGTTG

408. >MSTRG.8616.1 Gene=MSTRG.8616
TGTGGCTAAGTCGGAACGCTGCTTCTTCCGCACGCGGAAAGACTCGGTTCGAGACTCGCTT
CTCGACTCGAGTTTTTTTCGCGAGCGACGGACAACTTCTCAACTTGTCACGAGGGAATGACC
CTCGGTGTCTTTCTCCCGCTACGGACTTGGGAAGTCACTCTCCGGTCTAGGGGGTTTTTCACC
CAGGAGACCACTCTCTCCGGTCCGGGGGGTGTCCGGATCGGGGAGGATAAAACTAAAAGG
ATAATATATTTGAAAAAATTAAAGAAAGAAATGAATTTAATCATTTATCATCAGATGGATT
ACTTTAAGTGGAGA

409. >MSTRG.8617.1 Gene=MSTRG.8617
GATGCAGTGAAAAATATTGTTTATTAGTGGAATTCACATCCGTGGAATAATTAAAACTGCA
AATTCAATATGCATTTGATCGCATTAATGGAATGCAAAAGATAATATAAAATTCGATGCAA
TATAGTAAATAGATTGCAAAATTGCCTCGACGACAATGATAATCACATAGAATAAATATTT
TTACTAAAAATGATTCGAAAATGGATCAATTCTTTGAGTTCGTATAAAAAATAGAAATTCA
TTAAATGAAAGGAAAAAATTCAACATTATCATATGTGGAGAACGAAGAGGCCATATGTGA
AAAAATATGCATTTTTTGTGACCGATGATGTGGAGCATATATAAAATGTAA

410. >MSTRG.8618.4 Gene=MSTRG.8618
ATTGGATCACAAATGAGGTTTATGTATAGATAAAATATTAAAAGAGGTGAAATAGAAAAT
ATATAAATTAAGTGATCAATATTCTTTGTGCTCTTTATATATTTTTAACAATTTTTTTGATTGT
ACTTACTAATAATTTAATTATCAATCTTGTTGATTTATAAACAGTTATAATTTTTATTACAGTT
GTACATTTTATTAAATAAAATTCAAGTGAACATATAATACAGTTATAATTGGATAGTAACA
AGAACATTTTCATATTATAAACAAACTTATTAAACACTTCATTGTAGTGTTTATTAAAATTG
GTTCAAAAATCTCTATTGCTTAAAATCTGTATATATTATTATTAAAATCACAGGTACAAAAT
TACGGAGCAGAAGTAAAAAATGGATTATTCTAACGGAAATGAATTTGAAATATCTTATTTT
AGAACGATATGGAGTGCTATTGCATCTATTGGGTGGTATTTAATTGCAATTGGAATCTCATT
TTGGCTTGCATCTCCTTATATTCGAGAGAAATATTTAAATTGGAAACTAAGAAGGGACGAA
TTGGAATATGCAGCAAAATATCGTAAAAATCCAGATCTGTTGCAAGCAAGATTATTAGGTT
TGGAAGCTTCAAGACAAAGGATGCAAGAGGAATATTATCAGAAATGTGCCATGTTAACAG
AAGAAAAAGGAAAAGAACAGAAAGAAAAGATAAAAGAAACATCAAAATTGACTTTGGA
CAGCAATGTCTTAGACTTTAGACTAGGAAAGAAAAAATAATGATTTCACAGAAAAGAAACA
TAAGTCATTAAGAGAAGAATATAATCCATTAATGGGTGATGGTTCTAGAGGATATCGACCA
CCTAAACGTACTTGTTGTGGAAAGGGAGGTTGCGGGT

411. >MSTRG.8676.1 Gene=MSTRG.8676
AGCTGAACGTTCATAGCTCCACCATTCCTTATTGTTAGAACAACTGTCAAACGGAATATTTC
GTTCGTGAATCTGTTTCCTATATATATTGATAAACACGATAAAAAAGCCAAAATTGGGTGAT
CTATTAACGTAAAAGCGTTTCGGACGCATCCGGGTAAGTTTTTTTTTTAAATTTCATTATAA
TTCAGAAAGTATTTACAAAATACATTCAAATGTCTAACTAAAACAAAATGGCGAAATAAC

GGGAATAGTACAATTAACTACAATTACAATGACTTTTAATTATCAATTAATTTTTCATAACC
TAATTATATTTTTTAAATAAACAATTTTTTTATGGTAATTTCTTTTTTATTATTAAAACAATTA
AAACAAAC

412. >MSTRG.8724.3 Gene=MSTRG.8724
CGATCAGTAGTCCTAATGTTGGACTGGCGAAATCGACGAAAACAATAATTTTTAGACGAGT
AATTACTAAATTTAGAAATCAGTACCGCACGAAGCAGGACAAAAAGCGTTGTTCTGCGTTC
GTTGTCGGATTTTATAGTGATATCTACGTTTCTTGAATCAGTAATAATTCAAGCTTTATGGTC
CAGATCATGCAAAATAGAAACGAGCGCCCATCGCCAATTGTGTGCATTGCGTATCATCAAT
TAAGTATAATGACTGGTATTAAAGATCCAACTAAACAACCCGATTTAGAGAAAATAAAAC
AAGAGAATGAACAAATGAATGTTATTGTTAAAAGAAGTTTTATGGATATAAAAAATATGTA
TATGCAACATTTTTTTAAATTTTCTAAAGATATGGCAGAATCTTTCAGGTCAATATTAGATG
ACATAAAAGTTCGTATTGAAGAATTTCATGAACAGAATCAATCTATATTTGATCTATTTTCA
CTTTCACAACTACAAAAGTGGTCTCAATCCCAACGTGAACAATAATTTGAAGTATGAAAAT
ATTGATGTTTTTTGATAGATATGTTTTTAAAATTTATAATTATAAAATAAATAACTTGACCAA
AATATATATTTACGTTCGCACATTCTTGATGAAATAAAATCATTATGTATATTTAAAATCGT
AAAATTAATAAAAAAAATATTATGAAAAAATTATTTATATTTAGAATATCTTAATTAATAA
ACAGTAGAAAATAATTATATAATATTTAATTATGTCATTAATTATATATTTTATACTTGAAAT
TTAAAATGTTGCAGTTGAATGTATATTATTTAAATGCTGATAATATGACGACGACGCATCTA
CATCGTACGCCATCTTAATTCGAGTATGGTTGCCGCCAATGTACTACGTTTCTCTAAATTTTA
TTACTTTGTGAATTCTTAAAAATTAATATTTTTTTTTGGAATTAATGATTACGTATTTATTTTTT
ACATAATCCATTTTTGTTCATTTATTTCTATATATTAATATGTCACAACAATACTGTTTTCTTT
GTGCAAGTGATGGTGGTGTTTTCTTGGATATTACGGAGGATAATAAACAAGTATATTATGAT
CAATTTGAGATCTGCTCACTTGTTAAGGTAGTTTTACATAAAAAAATGCGTGCTTAAAATCA
CTAATTTGCTAGTGAATATTCAAATCTGATTAAATTTTCTGGAGCAAATCTGAACGTGCG
413. >MSTRG.8750.1 Gene=MSTRG.8750
AGAATTGGTGTGGGTTGAATAACTATTGATAATCATGAAGAATTTTGCCTTAGCAATTCTTG
TTGTTACCTTTGTAGTCGCGGTATTTGGGAATACCAACCTAGATCCGCCTACTCGACCTACG
CGCCTTCGCCGTGAAGCTAAACCGGAAGCTGAACCCGGCAATAATCGGCCAGTATATATTC
CACAACCAAGACCACCTCATCCGCGTCTTCGCCGTGAAGCTGAACCGGAAGCTGAACCCG
GCAATAATCGACCAGTATATATTCCACAACCAAGACCACCTCATCCGCGAATCTAATTTAT
GAAGAATATGAAGCACGTGAAATATCAAATATTTAATAAAATATTTTTATTCGATTCATTAT
AAAATATTGTATGTAATGCTA

414. >MSTRG.8753.1 Gene=MSTRG.8753
TGCTGTGTTCTCGTTCGGATCTCGGTGTTCCGTATATTTCGATGACTGACTGAATCCGCAGTC
GATAGTAGCGATAGTTAGCTATCGATTGTCGAGCGTGATCCTCGTCGTCGACATCGACCCGT
ATCGAAATTGAAAGAACGATGCAAAAGTGAATGCGAGAAAAAGGTAACAGAATCAATGA
CAAGTCGTGGCAAAGTGAAAACAACTTTTTGCGAACATAATATCGACGTATAGTATAGAGT
AAGACGAAATTGGATGGGATAAACGGCGACAAGAGGGAACAATTACGATCTATCGAGAA
GAAAATCAGAACGTAGAGAGAAGAGGAGAGAACAAATCGAATGGAACAGTCGGTCGCGC
GCCAGCACCAAGAACGCAGTGACCGTCGTACCTGTCCGTCGGTGGCCGAAGACTTGTCCGA
TCGAGACTCGGGAGTGATGTGGCAACGTTGCTGGTGGCTCGCGAGTGGAGGAGTCAGTCGT

TGCTCGGCTGTTCTATCGTAGTCTGTGTCTGACAGTGCAGTGTCGCTGCCAATAGAACCGCG
AGGGCCCGTCAGGGCCCACGAACAACGCGAACACGAGCACCGCGATAAACACCGCGGTC
AAAATGACATATTTAAGAAAAATAAGAACGAATTACATCACGAGTATCGAAGGGACAATC
GTACAAACACGTCGTTCATTGCTACGATAGCGCATGTTCACGTTGTTTATTACCATCGTAAA
ATAGCTCAAGAGAAGAGAGAGTTTTCATGACTTGCGAGAGAGTAACAGTGTTTCGAAGCGT
ACAATCGCTGTGTGACGCGCGTATTCGTTGTATATACACATCCGTTTCGAGTCGCTACAAGC
GGGTTTTCTTCGAACCTTTGTAGAAGTTCCTAAAGTGCGTGCGTACGGTTACATACGCGGCT
GCTACTCGAATGTCGAGAAACGAATGAGTGCGTGAATCGTGACAACATCGTGTCGAAGTGT
ACAGAGAGTACTCAGAGAGAGAGTGAGAGAGAATCCGTCTCTCTTTCTCTCGCATCCCGTA
GAGGCCGTGGTCGGACTTTACGCCGTCGTACGCGCTTCTCCCCTCCCCCTTAATAGACTGCT
TCTTTCTCCCTCTCTCTCGCTGAAGGCCAATTTATCTATCCCGTCGGCCGTACGTCTGTCTTA
GTCTATACCATTCCTGTCTGTGCGCTGGCAGAGTTTTCACTGGCGCGACACGGCACGGCACG
CGCGCACGGTACGACACGTAAACGAAACCGGCTGTCCAGGCGAATCGGGAGTGTCAAAGA
TCCGAAAAATCACGGGCATGGGAACAGTGTGTGCCATGGACTGAAAAATAGTGCGGGACC
CGTTGTCTCGTGCTTACCGCCGTCCAAAGAAGATCTTGCATTTCATCCGGTGCGTGCTTGTGT
GTTCCTTTACACCAAAGGAGGAAGAGAGGTTAGGTTAGGCTAGGACTCGCGTCACCCCTGG
CCACAGTCCGTCCTTGTCTCTCTTACACTTGGTTCCCCTACCTCTCTTTCTCTGGCATACAAC
GAAGAGGAACGCAGACACAAGAGTCCAGACCGATTACATACACACGTACGCGCGCATAC
ACGCACACATACATACGCAGGACAATTGGGGAAAGAGGAGGAAAACACAAATGCCGATC
GCCCGTGGAATTCTGGTCGCTGGAACTGCGGCTCATTGCTGGTCTTCCGCCGCTGGCATGCT
TCGATTCCTCGCTAGACTTTCAGATGCGCCAACGGTTATATCGGGCAGAGATGCGAGTTTA
AGGATTTAGATGGTTCCTATTTACCATCCCGGCAACGTGTAATGTTGGAGACGGCAAGCAT
CGCCGGCGGTGCCACGATAGCAGTATTCCTGGTCGTTATCATTTGCATAGCGGCTTACATCC
ACTGTAAGCGAAAGCAAAAGGAATTGCGGTCGAGTAACTGCGTCGACACGGTGGATGGTC
CTGGCCGAGATCCGGAGTTGAGGCCGTTTAGCAACCGTAGCCGCTCCCTCATGATCTTCATG
ACCAAGAATCCCAACAGCTCGGCGGCTATAGAGCAAACGAGAATGCCCGGATGGAACTGC
CCTGAAGCGGAGTCAATGAGAATGGCGTCGATCAGCGAAGGGAAGCGTTCCAATCAATGA
TAAAAAAGGATGATCCCGGTAGGTGTGTGCCTGATAATTCCACGACACGGGCCCGTACGA
GGCGTCCACCTCGCGTGTGGAAGCGTTGTGGAGCATCAGCGAAACCGACGGAAGGGAAAA
GAAATCAGCTTTTCTTCACCTTCAAACCTCTGCTATCTGTCTCCGTCCATGGACCAATTCTCG
CGATTAGGCGAGCGTTAGAGAGGAAATGCAGGCAGAGAAAGAAAAGGAGGGGAAAGAA
TTTTTCTCTTTCTTATTTACACGAGCCTTTGTCACGTCGACGTTTCATTTTCCCCGCATGAATT
TATCCGTTTCTGCCGAGAGAAGGAAAAGAAGGAGGAAAGGGGAGGAAAAGGAGAGAGA
AAGAGAGAAGAAGAAGAAGAGAAAGAAAAGGAGGAGGAGGAGGAGGACCTCTTCT
CCTCCTCCTCCTCCTCCACGTGTTCGTTCACAATCGGACAAGATATATTAAAAAGCAAA
AAAACAAAAAAAAAAAGAAGATCTCGTCGTACTCGCTGTATAAATCGATACGTGGATCGG
GATCCTCGGGATCGATGATCAGGGGCAAGATAGATCTAGAAGAGATCGTTAGGATTAATC
GAGAAAAAGAAGTAACGTAGCTGGTAACTTTAAATAGCTGGACCTGAACAGCTGGAAGAA
CAAAGGACAAGAAAGTAGAAAAGAAGGGAAGCTTAAAAATAAAGCCTTCTAACTACTGA
ATAGGTGACTCTCTTAATAGAAAGGGGAAAAAAAGGAAGAGGTGTGAAGAGGGAGAGAC
AGAGAGAGAGTAAAGAAACATTTAGCGAGAACTAGAGTAGTAAGAAGGAGAAGAGGTAT
AAAGTAATTTGTTCCTATCGGAGTTACGAGATATATATATAATAGTTTAGAACAGACAATA
ATAACCGGAGAATAATTACGTAACGTTATGTATTAGCATCGGTTTAAGATAATAACGTATTC

AGTTAAGTTTTTCGAACGAGATAATCCATTTTGCTAAGTAGAAACAGTTGCCACTTTGTTTC
GTAGAAACATTCTTCTTTTTTCTTTTTCTTTCTTTCTTTTTCCTCGACCGAGGAGGAAACGCGC
GGAAATAAGCTTTCCTCCTTGGCACTGGGGGGAGGGGAGGAGAGAGGGAAGGGAGTTAGT
TTTCACTTTTGCTTCTTACCTTTTTTTTTTCCCTTTCATTTTCTTCTTTTTTCCCCTCCCACGCCC
TGTTTTCTGTTTCGTCCGATTTCGTTCGTTTCTTTTTCACATGGTAACTCCTCGTGTTTCGTTAC
CGACCGACGCCGAGATCGATCCGAGTTCCAAATTCAACGCATACACACACACACACACAC
ACAAGTCAAATCTCTTCCATCGAGACATGGAAAATAGAACGAGAATCATTTATATCGTATA
TCGATTTAACATAATGCAAATTACGTTGAGCGTATAATTCCTCCTTCGTCCAAGACGAGAAG
AAATTAAAAATCTTTGTATCGTTCGTTTTTCGTTCGATGAAACGATATATGTGTGTATATATA
TATGGACAATTGATCCGCGTGATCAAGACGAGAAAAAAAAACTAAACTTGAAAATGTTCTTC
TCATTGTAAAATGTTCGTTTAAATGTACGTACGTAATTATTTATTTGAAAATCTAATTTAAGT
ATATATATATATATATATATATATATATATGTATGTATACATACATGTATTATTTTCGTCCGC
ACAACTGTGTAACTTAATTTAAATTGGCGTGTGAAAAAGAGATGGAAAAGGGACTCGTGA
GTGGTGTCTCTGCGCGGTGGCGTGTTCAGACCTAAGCGAGAAAGGAGCTAAGCCTCGGTAT
AGCGTTCGATTAGAAAGAAAGGTCGATCGAATCCTAATAATTGCACGAGATGGCGTGGAT
GGTATGGTGGATCTTTGTCGAGAGCCACGTCGATGCGTATCCCTGTTTCTTGCCTCTCCTCTT
GTAAATAGTAAATTATCCTGAATAATATATATGTATATATATATGTATATACATACATGTAT
ATATATACGTATATATGTATAAAGGCCAGCCGACTCGTCCCCCGTGTCAAATTTATTTAAAA
CGCGAGCGATTGGCATCCATTGTAGCGGACAAACTACAATTATGACGACGCTAACCGGATT
TCCAATTCCTCCTCCATCATGACCTCCATTGACGAATAAATATTTCATCGGGAGACGAGTCT
GACAGGGATTCATCGTTGTGGCGTGTGCTCTCCTCTTCGAGAAGAATATTAATATTACGAAC
GAATAATTATTACGAAGAAACTTCACCATCGAAAGATATCTCGAGAGGTATCGAGGAATC
GAAAAATGCCATCCGTGACGCCATCTCGGGAGATTCTGGGGGAAGTAACTGGGATATATCG
ATTGGGATGGTCGAACCTGATATTCGATTTGTACATTTGTAGATAATGTGCCGTGGTGGAGT
GGACAAGCTTCCAAACGCTTCGAAGATAACGAAAAAGGTGAAATCGTTCGATGTACTTCTT
CTTCTTTTTTTTTTTTTTTTTTTTTCCCTCTCTTCTTCTTCTTCGCGTGTAAGAAAACGCGAGA
GTAAAACGTGTGCCGCGCTAGATTCATGACCCTCCCGGTTTCTTTCGTCTCCCGTGTTTCTCT
CTTTCGTATAACCTCCAAATCGAGGTTTTTCTATATCGAGGAAGGTAATCGAACGAACGGA
GGAAACGAGAAAAGAGCTAAAACGATAGAGAGCCGTATAATCGATAATTACGATATTTAC
GTAGAATTTAGGAAAAAATAAAAAATAAAAAAAATAAAAAAAGGGCAGAATCCGAGACG
GGAAACGCGTGAAGCTAGGTGGCCTAAAGTGTATTTTATTTTATTTTTATTATTATTATTATT
ATTATTGATTATTATTATTATTATTATTTTGTCACGGTTTACGAGGCCGTTTAATTCGTAATTC
GTACGGTGTAATTTATTATAAATAGTCCTAATTAACGTGAAGAAATCGTAGCGTGAAAAAA
GAAAAAAGGAAAAAAAACTCTTAATTTAATTATCCCCCCAATCTTACTTCTCTCGTAACTTTG
TACTTGGTTCCTAATTTATTCTTACATTATTATTACTATTACCATTATTATTACCACCACTACT
ACTACTATTACTATTATTACTGCTACTACTACTATTATTATTATTATTATTATTATTCTATGTC
CCCCTTTAATTATTTGTCACGGTTATTATTATCATTAACTTAACTAATTTACGATCCATTAAG
TTACGATCATTTAATTATTCTCCTTTTATTATTATTATTATTATTATCGTCGGATCACGGATTA
ATCAGTTTATTCCTTAATAATCGCTATTCGCGTTATTATTTGGTTAACACGGTTATCTTTTTAA
GGATTACGCACTTATTATTATTTATAGAAAAAAAGAAAAAAAAAGAAAAAAAAAAAGAA
CGAACGAACGAACGAACGAACAAAAGAGGAAGAAAAAAGAAAAGAGAGAACGATATAG
TATATATAAATAAATATATATATATATATATTATATTTAGAAAAAAAAAAAAAAAGAAAAA
AACGAAGAAAAGCACGCAATTTCCGACGCCAGTTTACCTGTGAGAACGCTCGCCGTTCCTC

TTATCCGTGCGTATCTCGACGAAGAAGATTCCTTTCGATTTGTTCTTTAATTTAGGAGTCATA
TTTTTCTTTTCCTTTCTCTCTTTTTAGAGATTCGCCTTTATCAAGATCAACAGAGACAAATATC
CCCGTGCAATTCAATTCCCGCGCGAAAAAAAATGTAATAATAATTCGAGAGAATGAAATTC
GAGTAATTGCTTTTGAACGATTGAGTTAACTGTTCCTCTTTAATCAATTGCGATTTTCGTTCG
TCGCGCATCGAGACGACAACACGAGCTATTACTCGAAACTGCCACCAATTGGCCGATTGAA
GGAGGGCAAATGGGAGGGAATCTTCTTAAAATGGTTACGTGATCGTCGAGGAAGGAAATA
GGTTCATAGGTAGATTACGAGTGTCTCAGCTTCTAGAAACTTGAAAAAAATTAATCACTCC
GTCACGTCCGAGTGATTGCTCCGCGACACCGATCGTCTTCGTATTATTGAATTTCTTTTAATT
TAAATGCAACCGATCGATCGAGGGTTTTTTTTTTTTTTTTTTTGATTTTTTGATTTTTTTTCTC
AATTTTCGATCTTGTCAATAGAGATTAAAAAAATGAAGACGAGGGAAAGAAAAAGAAAA
AGAAAAAAAAAAGAAAAGAAAAGGAATCGGTCGCGGTCGAGAGCGATGACTCGGGCCTG
GTTACTTTATTAGCGACCGTTTAAGCGTAGACAGTGGTGGCATTTTAGTACATCAGACAGCG
CCGTGGCCGTGTATCGCGAAGATCGAGACGAAGAGTGGGGGAAAAGGGTGGG

415. >MSTRG.8753.2 Gene=MSTRG.8753
TTAATCTGTGAAATCTTTCCTTTCTTCAATCTCGGCCGTGATTTCGGTTAATTAATTCCGTTCC
AAGGGCGAATCATCATCAGTTCACGGAGGAATACCGTTCGATCAAATCGACCATGCGCCA
ACGGTTATATCGGGCAGAGATGCGAGTTTAAGGATTTAGATGGTTCCTATTTACGTAAGTAT
CCAATTCTTCATTCTCTCATTGTATTCCCGCACGCTCGTCGTCCGTATCGTCTTATTCCATTCT
AGAAGCCTCGTTTCTATATAGCGTGTATAGGCGGTGTCTATACACCCATTAAATGTCGAGTG
TGTATAACGCGCTCGGTTACAATGTGAATTCGAAACGAACCTTCTCCTGTCTCTCTTTCTCTT
TCTCTCTTTCTTTCTTTCTTTCTCTCTCTTTCTTCCCCCTCCTTTCTTCCTTTTAACGTCCATTTTC
TTGAAACGCGTGCGCGAGCGGAGCGGTAAGCATAAGCGAAGGGGCAACGAGGTTGCTGCT
TGTGTGGTTAAGAAAGTGACAATAAGTTTTTCTTTTTTCGTTACAGCATCCCGGCAACGTGT
AATGTTGGAGACGGCAAGCATCGCCGGCGGTGCCACGATAGCAGTATTCCTGGTCGTTATC
ATTTGCATAGCGGCTTACATCCACTGTAAGCGAAAGCAAAAGGAATTGCGGTCGAGTAACT
GCGTCGACACGGTGGATGGTCCTGGCCGAGATCCGGAGTTGAGGCCGTTTAGCAACCGTAG
CCGCTCCCTCATGATCTTCATGACCAAGAATCCCAACAGCTCGGCGGCTATAGAGCAAACG
AGAATGCCCGGATGGAACTGCCCTGAAGCGGAGTCAATGAGAATGGCGTCGATCAGCGAA
GGGAAGCGTTCCAATCAATGATAAAAAAGGATGATCCCGGTAGGTGTGTGCCTGATAATTC
CACGACACGGGCCCGTACGAGGCGTCCACCTCGCGTGTGGAAGCGTTGTGGAGCATCAGC
GAAACCGACGGAAGGGAAAAGAAATCAGCTTTTCTTCACCTTCAAACCTCTGCTATCTGTC
TCCGTCCATGGACCAATTCTCGCGATTAGGCGAGCGTTAGAGAGGAAATGCAGGCAGAGA
AAGAAAAGGAGGGGAAAGAATTTTTCTCTTTCTTATTTACACGAGCCTTTGTCACGTCGACG
TTTCATTTTCCCCGCATGAATTTATCCGTTTCTGCCGAGAGAAGGAAAAGAAGGAGGAAAG
GGGAGGAAAAGGAGAGAGAAAGAGAGAAGAAGAAGAAGAGAAAGAAAAGGAGGAGGA
GGAGGAGGAGGACCTCTTCTCCTCCTCCTCCTCCTCCACGTGTTCGTTCACAATCGGAC
AAGATATATTAAAAAGCAAAAAAACAAAAAAAAAAAGAAGATCTCGTCGTACTCGCTGTA
TAAATCGATACGTGGATCGGGATCCTCGGGATCGATGATCAGGGGCAAGATAGATCTAGA
AGAGATCGTTAGGATTAATCGAGAAAAAGAAGTAACGTAGCTGGTAACTTTAAATAGCTG
GACCTGAACAGCTGGAAGAACAAAGGACAAGAAAGTAGAAAAGAAGGGAAGCTTAAAA
ATAAAGCCTTCTAACTACTGAATAGGTGACTCTCTTAATAGAAAGGGGAAAAAAAGGAAG
AGGTGTGAAGAGGGAGAGACAGAGAGAGAGTAAAGAAACATTTAGCGAGAACTAGAGTA

GTAAGAAGGAGAAGAGGTATAAAGTAATTTGTTCCTATCGGAGTTACGAGATATATATATA
ATAGTTTAGAACAGACAATAATAACCGGAGAATAATTACGTAACGTTATGTATTAGCATCG
GTTTAAGATAATAACGTATTCAGTTAAGTTTTTCGAACGAGATAATCCATTTTGCTAAGTAG
AAACAGTTGCCACTTTGTTTCGTAGAAACATTCTTCTTTTTTCTTTTTCTTTCTTTCTTTTTCCT
CGACCGAGGAGGAAACGCGCGGAAATAAGCTTTCCTCCTTGGCACTGGGGGGAGGGGAGG
AGAGAGGGAAGGGAGTTAGTTTTCACTTTTGCTTCTTACCTTTTTTTTTTCCCTTTCATTTTCTT
CTTTTTTCCCCTCCCACGCCCTGTTTTCTGTTTCGTCCGATTTCGTTCGTTTCTTTTTCACATGG
TAACTCCTCGTGTTTCGTTACCGACCGACGCCGAGATCGATCCGAGTTCCAAATTCAACGCA
TACACACACACACACACACACACAAGTCAAATCTCTTCCATCGAGACATGGAAAATAGAACG
AGAATCATTTATATCGTATATCGATTTAACATAATGCAAATTACGTTGAGCGTATAATTCCT
CCTTCGTCCAAGACGAGAAGAAATTAAAAATCTTTGTATCGTTCGTTTTTCGTTCGATGAAA
CGATATATGTGTGTATATATATATGGACAATTGATCCGCGTGATCAAGACGAGAAAAAAAA
CTAAACTTGAAAATGTTCTTCTCATTGTAAAATGTTCGTTTAAATGTACGTACGTAATTATTT
ATTTGAAAATCTAATTTAAGTATATATATATATATATATATATATATATATGTATGTATACAT
ACATGTATTATTTTCGTCCGCACAACTGTGTAACTTAATTTAAATTGGCGTGTGAAAAAGAG
ATGGAAAAGGGACTCGTGAGTGGTGTCTCTGCGCGGTGGCGTGTTCAGACCTAAGCGAGAA
AGGAGCTAAGCCTCGGTATAGCGTTCGATTAGAAAGAAAGGTCGATCGAATCCTAATAATT
GCACGAGATGGCGTGGATGGTATGGTGGATCTTTGTCGAGAGCCACGTCGATGCGTATCCC
TGTTTCTTGCCTCTCCTCTTGTAAATAGTAAATTATCCTGAATAATATATATGTATATATATA
TGTATATACATACATGTATATATATACGTATATATGTATAAAGGCCAGCCGACTCGTCCCCC
GTGTCAAATTTATTTAAAACGCGAGCGATTGGCATCCATTGTAGCGGACAAACTACAATTA
TGACGACGCTAACCGGATTTCCAATTCCTCCTCCATCATGACCTCCATTGACGAATAAATAT
TTCATCGGGAGACGAGTCTGACAGGGATTCATCGTTGTGGCGTGTGCTCTCCTCTTCGAGAA
GAATATTAATATTACGAACGAATAATTATTACGAAGAAACTTCACCATCGAAAGATATCTC
GAGAGGTATCGAGGAATCGAAAAATGCCATCCGTGACGCCATCTCGGGAGATTCTGGGGG
AAGTAACTGGGATATATCGATTGGGATGGTCGAACCTGATATTCGATTTGTACATTTGTAGA
TAATGTGCCGTGGTGGAGTGGACAAGCTTCCAAACGCTTCGAAGATAACGAAAAAGGTGA
AATCGTTCGATGTACTTCTTCTTCTTTTTTTTTTTTTTTTTTTTTCCCTCTCTTCTTCTTCTTCGC
GTGTAAGAAAACGCGAGAGTAAAACGTGTGCCGCGCTAGATTCATGACCCTCCCGGTTTCT
TTCGTCTCCCGTGTTTCTCTCTTTCGTATAACCTCCAAATCGAGGTTTTTCTATATCGAGGAA
GGTAATCGAACGAACGGAGGAAACGAGAAAAGAGCTAAAACGATAGAGAGCCGTATAAT
CGATAATTACGATATTTACGTAGAATTTAGGAAAAAATAAAAAATAAAAAAAATAAAAAA
AGGGCAGAATCCGAGACGGGAAACGCGTGAAGCTAGGTGGCCTAAAGTGTATTTTATTTTA
TTTTTATTATTATTATTATTATTATTGATTATTATTATTATTATTATTTTGTCACGGTTTACGAG
GCCGTTTAATTCGTAATTCGTACGGTGTAATTTATTATAAATAGTCCTAATTAACGTGAAGA
AATCGTAGCGTGAAAAAAGAAAAAAGGAAAAAAACTCTTAATTTAATTATCCCCCCAATC
TTACTTCTCTCGTAACTTTGTACTTGGTTCCTAATTTATTCTTACATTATTATTACTATTACCAT
TATTATTACCACCACTACTACTACTATTACTATTATTACTGCTACTACTACTATTATTATTATT
ATTATTATTATTCTATGTCCCCCTTTAATTATTTGTCACGGTTATTATTATCATTAACTTAACT
AATTTACGATCCATTAAGTTACGATCATTTAATTATTCTCCTTTTATTATTATTATTATTATTA
TCGTCGGATCACGGATTAATCAGTTTATTCCTTAATAATCGCTATTCGCGTTATTATTTGGTT
AACACGGTTATCTTTTTAAGGATTACGCACTTATTATTATTTATAGAAAAAAAGAAAAAAA
AAGAAAAAAAAAAAGAACGAACGAACGAACGAACGAACAAAAGAGGAAGAAAAAAGA

AAAGAGAGAACGATATAGTATATATAAATAAATATATATATATATATATTATATTTAGAAA
AAAAAAAAAAAGAAAAAAACGAAGAAAAGCACGCAATTTCCGACGCCAGTTTACCTGTG
AGAACGCTCGCCGTTCCTCTTATCCGTGCGTATCTCGACGAAGAAGATTCCTTTCGATTTGTT
CTTTAATTTAGGAGTCATATTTTTCTTTTCCTTTCTCTCTTTTTAGAGATTCGCCTTTATCAAGA
TCAACAGAGACAAATATCCCCGTGCAATTCAATTCCCGCGCGAAAAAAAATGTAATAATA
ATTCGAGAGAATGAAATTCGAGTAATTGCTTTTGAACGATTGAGTTAACTGTTCCTCTTTAA
TCAATTGCGATTTTCGTTCGTCGCGCATCGAGACGACAACACGAGCTATTACTCGAAACTG
CCACCAATTGGCCGATTGAAGGAGGGCAAATGGGAGGGAATCTTCTTAAAATGGTTACGTG
ATCGTCGAGGAAGGAAATAGGTTCATAGGTAGATTACGAGTGTCTCAGCTTCTAGAAACTT
GAAAAAAATTAATCACTCCGTCACGTCCGAGTGATTGCTCCGCGACACCGATCGTCTTCGT
ATTATTTTTTTTTCTTTATTATTATTATTATTATTATTAACATTATTAATATTATTTTT

416. >MSTRG.8767.1 Gene=MSTRG.8767
CACTTTATTTGCAATTCGAAATGTTCTAAACAGTTTTGGTGACTTTTATATAGTGAAATTTTA
ATATTGGAAATCCTGTTTAATATATTGTGCAATCAATTTGGCCATTTCGTCAGTTGCTATCCG
AACAAAATGTCGGCTCATAAAACGTTTATTATCAAACGAAAACTTGCTAAGAAATTAAAAC
AAAACAGACCGATTCCACAATGGGTCAGAATGCGAACTGGCAATACTATTAGATATAATG
CTAAACGGCGTCATTGGAGAAGAACCAAATTGAAACTGTGCTGGTAAATAATAATTTTTTG
GTGGTTTTCCTTCTTCTAAACGTACAAAGTAATGAGTATGTCTATAATGTACCATTCCAATTC
TTGATCTAGCATGACGTCGTATTCCTTTTATTACTTCACCTTTTGTTGCGAAAGATTCAGCTG
AAAAATTAATTAAAATAATATAATTTTATTAAATTAAAATAATATAATAAAATAATATTAT
ATTATAATATATTTGAGTATACTTTATAATTAATATACTGACCAACCCATAAATTACTTTTGA
ATTCAATATCATGTTCTTCTACAGCTATCTTTTGTGCTTCTAAGATCACTTCTTTGGCTATAGC
TGCACCTTTTTTCTTTAAAAAACTCAATTGTTTAACAGCTTCATCTACAGACATACCTCTTAC
AAAAGAAGCAATATACCACATTTTCAATGGACTATATTTTATGTTTTTTC

417. >MSTRG.8767.2 Gene=MSTRG.8767
AGAAAATTGAATTTCTTGATACTCACTTTATTTGCAATTCGAAATGTTCTAAATTTTGGTGAC
TTTTATATAGTGAAATTTTAATATTGGAAATCCTGTTTAATATATTGTGCAATCAATTTGGCC
ATTTCGTCAGTTGCTATCCGAACAAAATGTCGGCTCATAAAACGTTTATTATCAAACGAAA
ACTTGCTAAGAAATTAAAACAAAACAGACCGATTCCACAATGGGTCAGAATGCGAACTGG
CAATACTATTAGATATAATGCTAAACGGCGTCATTGGAGAAGAACCAAATTGAAACTGTAA
AATACATTTATGTATTTTCTCACATTTATTATAATATATTAATTATTCATTTTTAAATAAATTC
TAGTTGTAACATTATATTTACATCTTATATTTATAAAAATTTATTTTTTAATTAACACGAATTT
TATATCGATCAATAACCTCAGAAAGAAAACGAATTATTTATTTACATATATTTATATACATA
TTATAATGAATTAGATATTTTGCGGCGATGCATTTGTTCCATCCATTCTTTTAATAATTCTTCA
CCGCTTTTAGGTGCTGGTAAATAATAATTTTTTGGTGGTTTTCCTTCTTCTAAACGTACA

418. >MSTRG.8790.2 Gene=MSTRG.8790
CTTAAGGGATAGGCTTGAAATATAAAACTGATAAAATTTAGTGGTTTGTAGTAAAAGTATA
ATAATAATT
TAAGTATCGTCAAGTGTTCCATATTTTTGGAATTTATTATTAAAAATTGTATTTCATAAAAAA
TTTTGTTGCCTTTATCTGTTATGGATATGGTGACTACGAATTTACAACAACAACGACAAATC

ACGGAGCAGCTACGACGTGAAGCTGCTTTAAAACGAATTACGGTTAGCAAAGCTGTCGAA
GATATCATGAAATATATCACAGAACACGAACAGGAAGATTATCTTTTGCAGCAGGAATAG
ATTCTGCAATAAATATATCTTTGGGATCTGCAATGTCTAATATTCAACATGTTTATAAAATTC
AACAAGTTTATGGAATTCCTCTTTATGGATATCATGAGAAGAAACATTTATTTTTTAAAATTT
ATTTTTATAATCCAGCTATAATTAAACGAGCAGCTGATTTATTGCAAAATGGTA

419. >MSTRG.8817.1 Gene=MSTRG.8817
GTTGATAGGTATCACGATCAACTCGAAGACTCCCTTCATCCCCAGAGTCAAGATTTGGTGAT
TGGATATTTCTTGATATCCTTAAGGGACTAGAGAATTGGCGATTACGCGTGCTATCTATGGA
ACGTGGTGAATCAGTCACGGATCTATTTCGCCATATCTTTGTTTTATCACAATCGTTTCCTAT
ATCGTTGGCTGGTTGATTATTATTATTGGCTGATCCAGCGTTCACGTTGTTGTTAATCGTGTT
GGAATTGTTAGTTTGTTGTTGCAACGTTTGACTCAACAGTGAGGACCATTGCCTCATCACGT
CTTCCCCTTGGGCCTCCAATTCTGATAGAATGGTAGCGTTGTCCATATCCTGCAACGCCGTC
CCAGTGTCCGCTCGTTGCTCTTCGTAGGAGCTCGCGTCGCTGTGCTCGTAATCCAATATCAA
GTCATCGGAGGCGATAGACTCGCAAGGGGACAGAGTGTCCACGCTCCCTGCTCGGCTCATT
CGTTTCCCGTGATGAGGGCTGTTGCTCGCGTTCTTTCCATGCCCGGTTTTAGGGCTGGACTCG
ACCAGAGTGTGGCTGTTCTCGGTGACCGGTTGGCTAGCCACAGCTGACTGGGTCTCGCCCC
CTGCCCTCGAGTTGTCGTCGAAGACAAGGCCCGGTTGATCGGCTATCTCGTCGTCGAAGTA
CTCCCTGCCCTCCGACAAGCTCAACGAATTGGAAGCGTTGGTCGGGGTGCCGGGGCTGTCG
TCGTCGATGATCGCCGTCCCCGACCCTTCCATCCCGCCGTTCTCCTTCCTCGGCGTCCTCGTG
TCGGACTGGGTGATGCTGGCCGTCAGGCTGTCGAACAGGCTGTCGACGGGCGACGTCTCGT
CGTCTATGAGGGCGGTCGGGGTGGCGGCCGCCGCCGCGAACGCGGGATCCTCTATGGTGA
GGAGGACGGAGCGCAAGGCCGTTTTGGGCAGCGTCCCCCCGGGCTTAGCCAGGTTCGGGT
GTTGGCCGTCGGAGGGCGGCCGTTGACACTCGTCGCTCGAGCTGAGCGAGTACTCGTCGGC
CATCGCCTCCCCGTGCCCGTACTCAGGATCGTCCGAGCTCGGGGGGCTGAAGTCCGACTTG
ATGGAGGGGATCTTCTCGGAACGCGACCTGTCCTTGTAAACCTTCTCCTCGCCCAACCCCTC
GTCGTAGCCCTCCGAGGAGGTGGTGGACATGGACGTGGTCGACTCGGTGTACCTGTTGTCCT
TGTTCACGATCCTCTGGTACTGGGTCGCCCTCTCCCTCACCAGGCCGGTGCTCGCCGAGGTG
AACGTTTTCGGCAAAGGTATCACGGATATCTCCGTCACGGTGCTGTCGTCGTCCCTCACGTC
CCTCACGTCGAAGCTCTTCCCGCTCACAACCATCCTCAGGTTCCTCAGCCTGCCGCCGCCGT
GCCTCCTCAACAGGGAACAACCGTCCCCGTAATCCCTCGACTCGTTCTCCCCGCACCCTTGC
TGGCTCCCGACCCCGGAATCCTCCGTGCTCGACCCCTCCTTCGAGCTCGTCGACCCCTTCGA
CGCGGCCGACACCTTCCTGCTCTGGTTCGCGGCGGTCTTCGCGATCTTCGAGTTCGCGTCCG
TCATCCTCACGGCGTATTGGGGCAACGGCAGGTGGCTCGTGTGAAGCGTGTACCTGCCCAC
GGGCTCCGGTATACCGGACGGCACCGCTCTCCGATTCCCGTCCACGAGGCTCCCCCCGTTG
AACGAGACCGTGTTGTGCACAGGGGACGATCTCTGCAGCTTGAAACTCGGCTGGCAGCCCC
TGTGCGGGGGCTGCTTCGCGAAACTGCCTTCCTGCTGCCGGCTCTCCTGGTTGTAGTGGCTC
GCCGTGTGGCTGCTGCACACGTCGCCCACCTTGTTCGTGAACCTCGCTGCCTGGGGCTGCTG
CTGCCTGTACCCGAACCGATTCGTCCTCACCGCGATCCCGCTCGACTCCTTCTTGGGGGGGG
CGAGGCGGGGCGTGGAGCGGCCGGAACTCGAGCAATTCTGAAGCGTCAACGCCGGGTTTC
CGCCGCCGTTTTCCTCGTCGCGCCTCCGGCTCTGCTCGGGCCCGGCCGATTTCGATCGTTGGT
GGAGCGTGCCGCTCGCCTCGTAAGCGCCGCCGCTTATCGGCATCGGGATCCCGGAGGCCGG
CCTTCGCCGGAAACCGAACGACATGGCCGTGCCGCGCGTGTGCGTCCCGCCGCGCTTCGAC

GCCCCCTTGTGCTCGTTCGCAACACCAATTCCTTTCTCGTTCTCCAGGGAATCATCCTCGCAC
CTGGTGCCAACAGACGACCGGTAATCCATGTCTTCGGGCCAGTGTCACCCGGCTGTTAAGA
AGACTGTATTCCCGATCGATCCCGGCGTCCAGCTTGCGAGAAAGTTACGTTAGTTTCTTCTA
AATCCGCAACGTCGTCATCCTGCACCGCTGTGCGTGAATATTTTTAACCGAGGGAGGCACG
GAGAGCCGAAATTTACACGGCTATGTTTTTTCAGCTCGAACAAACCTTCCCCGTATAATCTG
TCACCTCTTTCGGATGCTGTACGCGTTCAGCTCGTACGCGTTGTCGCCAAAAGACACGCGCA
CCAATACCTCCTCCGATTCTCCCTCTTCCGTCTCCCTAACCTCACTCCGTCCTCTCACTCTGTC
ACTCTCTTTCTTCCCTCACCGCTTCCTTCTCTCTCTTTCTGAAGTTCACCCTCGCGCTCAATCC
TCGGCCAGCATACCCCCTTTTCTTCTTTCCGCCTGAGTTCGTGTGTGCTCGTCACTGTTCGCG
TTTCCTCGCCACTACACGGACAGGCCAAATCGTGGAATCGATTGACGGTACTGCGCCAGCG
CGGCGTCGCGACGCCACCGCACACTATCGTAACCACTCGCGCATAGGCTGCGAACGACTG
ATGTCACGAGTGCGCCCGCGCCGCCGTTTGCCTTTCGATGTATTTCTATATCTAAGTATTACG
CTTTTGTCGGCCTCCCTCTGTTTCCTATCTGTACAGTGACATGCACAAAAAGCGGACCATCA
AACTTAACCTTCTCATCCGGCAAGGGCTCAATTCTTACCGTTTAACGATCACGATGTAGTAT
GCACTCGTGTACCGTCCAGTTGATTCCGAGTAATCGCATTACCGACTGGTTTCACGTTCGAA
TATTTTAGCAGAGTTTCTATGGATATACAATACCGGTGAATGTTTGTTACACAATTTTGAGA
AATCGTGAGAATAAAATATTTTATATATATATATATATATACTATTTTTTTTTAATTGATTCG
TTTGATGCCAATCTAAAAGACTTTCTAAGTTTCATCCTTTTTTTTTTTCTTTTTCTTTAAATTAT
ATGTATATACCTTTTCATAGGTACATTTACCCG

420. >MSTRG.8874.1 Gene=MSTRG.8874
GACGTGATATACAGATTATACAGAATTCAAAGAATTTCTTTTTCAAGATTATCAATTCATTA
ATGCATTTGCTAATTTAATGTCAATTTTTTGTATTTCAATTGCAGTGTATCTTGATCGTCCATT
TCTTGTCACTATTAAACTATTAAACTATTAAACTATTAAACTATGTGAAAGATACAGAAGGT
GAAAGAAACGGTAGCATTGCATATCTAGATGCACTAACATCTGAGCGAAAGGGCTGCGCA
ATTTTATTTTTCATCCACGAATAAGATAACGTGTGTAACTCTAGGATGCACTCCAATGCGCA
TGCGATTTACGAATGGTGGGGAAATGATATTTTCCCTTTCTCGGAAAAAGATATTCCATATT
TTTGGTCAAAGAGGAATATATACTCCTGTTTTAAAAAAAGAAAAAATGGAAAACTTACTC
AAGAAGAACGAGAGAAAGATTTAAAACCATTATTATTTAACAATTGGTCTGTGCAACAAG
ATAGAGATGCTATTTACAAAGAATTTTTATTTAAAAATTTCAATCAGGCATTTGGTTTTATG
ACCAAAGTTGCATTGCAAGCAGAAAAAATGAATCATCATCCTGAATGGTTTAATGTTTACA
ATAAAGTAAACATTACTTTATCTTCTCACGATGTTAATGGTTTATCACAAAGAGATATTAAG
CTTGCATCTTTTATAGATAACATTGCTAAAATGGAAGAAAACTAAGATTAAGAATATATGA
TTGGAATCATCATTAGGAGGTGGTTCTTTGCCAATAGCCCAAGCTTCTTTAATAGCTTTTGA
AACACCAGGACCATTATTTATAAGTAAAAATAATCCACATAATGTAATTGCTGAGAGGAA
AATCAATGAAAGATGCAAAAGTTTTGGTAATAGTGGTCCAACAACTCTAGGACGTGCATAA
TCTGTAATAATAGCTTCCAGACCCCTATTTGAAATTTCAAGAAATAAATGAATTAACACATT
GTATATTGGAACACATTATTGAATTAATGAATTATAACATTGCATAAATATCATGATGATAA
AGTATGATTAATAACAAATTTTAAAAAATAATTCATGAATAATTACCAATGCATATGTATTA
CAATTAATAAGGACATTAAACCATCACAGATAACATTTTCCATCGTTAAAGCAACAGGTAT
TATAACTGGAAGTGCTGCTGAAGCAATTTTTTCTAAAATCCACATACGTACATGATCACTTG
CAGTTGTACTTGTTTTGCGAGTTATTTCTGTATTTAGAATGATG

421. >MSTRG.8874.3 Gene=MSTRG.8874
CGCGCATGCGATTTACGAATGGTGGGAAATGATATTTTCCCTTTCTCGGAAAAAGATATTC
CATATTTTTGGTCAAAGAGGAATATATACTCCTGTTTTAAAAAAAGAAAAAATGGGAAAAC
TTACTCAAGAAGAACGAGAGAAAGATTTAAAACCATTATTATTTAACAATTGGTCTGTGCA
ACAAGATAGAGATGCTATTTACAAAGAATTTTTATTTAAAAATTTCAATCAGGCATTTGGTT
TTATGACCAAAGTTGCATTGCAAGCAGAAAAAATGAATCATCATCCTGAATGGTTTAATGT
TTACAATAAAGTAAACATTACTTTATCTTCTCACGATGTTAATGGTTTATCACAAAGAGATA
TTAAGCTTGCATCTTTTATAGATAACATTGCTAAAATGGAAGAAAACTAAGATTAAGAATA
TATGATTGGAATCATCATTAGGAGGTGGTTCTTTGCCAATAGCCCAAGCTTCTTTAATAGCT
TTTGAAACACCAGGACCATTATTTATAAGTAAAAATAATCCACATAATGTAATTGCTGAGA
GGAAAATCAATGAAAGATGCAAAAGTTTTGGTAATAGTGGTCCAACAACTCTAGGACGTG
CATAATCTGTAATAATAGCTTCCAGACCCCAATGCATATGTATTACAATTAATAAGGACATT
AAACCATCACAGATAACATTTTCCATCGTTAAAGCAACAGGTATTATAACTGGAAGTGCTG
CTGAAGCAATTTTTTCTAAAATCCACATACGTACATGATCACTTGCAGTTGTACTTGTTTTGC
GAGTTATTTCTGTATTTAGAATGATGGTATTTGTTGGAAACTTCAATAATTGTCTTTCATTTTT
ATTCAGACTAG

422. >MSTRG.8891.1 Gene=MSTRG.8891
GAGTACGTGAAACCGTTCAGGGGTAAACCTGAGAAACCCAAAAGATCGAATGGGGAGATT
CATCGGCAACGGTGCTGGCTCCCGTTGGTGCGCGATGCTCCGGATGAATCCTCGTGGTTCAT
CGGCGAGGGCACACCACCTTCGGCTCGAATGTTCCGGCATCGTAGTCGTGCACTTCTCCCCT
AGTAGAACGTCGCGACCCGTTGTGTGTCGGTCTACGGCCCGAGAGGGCGCCTGTCGCGTCG
CTTCGCGCGCACGCGGCAGACCCTCGGTCGCCTGGCCGGCTGCACGACGGTACTCGCACGG
TATCGGGCCGCAACCAATCCATTTTTTCGAATGTCTGAACGGGGCGTGGTCTTGTACCTCGC
ACCCCGGATCCGAGCTCGGTCCCGTGCCTTGGCCTCCCGCGGATCTTCCTTGCTGCGAGGCT
TCCGTGGCGGCTCTGCCGTCGTGGTCGCTCTCTTCGGCCGCCATTCAACGCTCAGCTCAGAA
CTGGCACGGACTAGGGG

423. >MSTRG.8895.3 Gene=MSTRG.8895
CGGCGGGAGTAACTATGACTCTTGGTAATCAAATGCCTCGCTATTTAGTAGCGGTAGCGCT
CCGCCCGCGCAGGAACCATTGACGCCGCCGTAGTGTGGGTGATTTTATATCCAACCAATCA
CGTCAACTACGATCATTTGTAATCACCGACGGTACTTGGTAGGGGTACCACATGGGCATTCT
TGCTCATTCCACAACGCCGCCTCCATCATGGCAACAATTTAAAATATATATAAATTCTTAAG
GTTTGACCGTATTCATATATATATATATATATTTAATATTACAACCATAAATCTTATATCGAG
CCTTCTATTTGGTCTCAAAAGCAATACGTTGTCAGATCTTGTAGAACATCAGGAGTGAGCGG
TTTGCTGTGGTATCCGTGCTTTGTGCCGCGGCGACAAACCAATACGCTGCTGCCTGTCGCAA
AGCAATACGCTGCTGATTCTCGGATGCGGGTGTCGACGGTCACGCAAAGCGATACGCTGGT
GGGGTTTCAAAACAATACGGCGCTGGTGCTAAAAAGCATTATGCCGCTAACGGCTGGATTG
TCGATCGCCGCTGCGGGGGCTAGTGGCGCACCCAGAGAGGTGCGACGCGCAAGCATTGGT
TCTGTGCGAAGCGGAGTTCTTGAGAGTAATGGTTGCTGGGGGCACAAAGCGCAACATATAG
CCTCTTATGCCTCAAGTCGTAGTTCGTACCTCCACGTGGTCCCGCTGGAATGCCTATCGACT
CCTCCCCGGAGGATCATAGAGTTCGAAACCGGCTACGGCGAGGCAAGGGCGGTGAGGTGC
ACACCGATGGGAAGCAGCGACCCCACTTACCCATAGCTAAGAGAGCAGGCGATCCGCCAA

CTGTCAGCACGAAATAAACTAATCATGTGTATACGAGGGAGGTTTTACAACGGGTACCTTG
TGCCCGAACCGCCTGTAGGTATCACCTACATGCGTTAAAATGAATCTGATAACTGGCGGGT
CGTTGACCCTCTCTGATGGGT

424. >MSTRG.8901.1 Gene=MSTRG.8901
CGTGGGATTATGCCTGAACGCCTCTAAGGCCGTATCCTTTCTAGTCAAAGATGGCAACGAT
ATCTCTAGGAGTCTCGTGTGGGTCGAAAGGCTCAAAACAATGTGACACTACTAGGTGGCCG
GCCCTCGTGACCGGTCATCGCACGGGCCCCAGTTTGCCGTACGGGCGTCTTTGGATTCGTCG
TCGGGATCTCGCCGATCGACGGCCACGGCGCTCTAACGGTCGATCATGGGTACTCCAACTT
CGACGTCGAGACTCGGAATCGTCTGTAGACGACTTAGGTACCTGGCGGGGTGTTGTACTCG
GTAGAGCAGTTACCACGCTGCGATCTGTTGAGACTCAGCCCTATGCTTGGGGATTCGTCTTG
TCGGTTAGACGAGACCCCACGGAATATAGTCAAGAAAATTATATGAAGAGGAGAGAGAGA
GAGAGAGAGAGAAAGAAAGCAGAGAAAAGAGACGCAAGAGAAAAAAATATACATCCAA
CCAAAAAACGAACAACAATGTAATGCACGCGAAGTATTATAGTAATAATACCCGTGTAAC
ATACGTTGTAAAACTACATGATAATATAATATATATATTACACACATATATATAATATATAT
TATATACATAGATATATAATATATTATTAAAAAAAAATTTTTTTTTTTTTTTAAATTTCAGA
AAGCAATACGCCGCTGGAACTTATAAAAAAAAAAAAAAAAAAAATTTTAGCTGGT

425. >MSTRG.8917.1 Gene=MSTRG.8917
TTTTGAGCAAAATAATCCTAAGCGATGAAGCACATTTTCGATGGCCTTGTTAATTATCGTGT
TTGCGGTTTGAACAACTCACGTGTGATTAGCAAAAAATAAATGCACCCACAACGTGTCACT
GTTTGGTGCGGATTTTGGGAAGGAGGCATCATCGGACCATACTTTTTTGAGAATGAGCAACT
AATTAAGCAGCAATCAGCTGTTAATGGTGCTCGATATCGCGACATGATAACACAATTTTTTC
TGTCGAAATTGGATAATATTTATGTGACCAATATGTGGCTTCAACAAGGCGAAGCCACATG
TCATACAGCCAATGAAACAATTTAATTACTGCATAAGACATTTCCTGATCGTATACTCTCTC
GTTTCGGTGATCAGAATTGGCCCCTAGATCATGTGATTTAACACCATTTGATTTCTTCTTATG
GGATTATTTGAAGTCAAAGGTCTATGTCAACAATCCCATAACCACATATGCATTACAAGAG
GAAATTAAATGTTGCATCAACGAAATTCAGCCATAATTATGCAGAAAGGTCATGAAAAATT
TCTACAAAAGGATTTAACGCCGTTAGACTTTTTTTTAGAATTTTTTGAAGTCTAAAGTTTATA
CCAATAAGCCCATGATCATCTATATCTTGAAGGGAAAATTTCAACGTAATATCAATGAAAT
TCAGTCACATTTATATAAAATGATCATGGAAAATTTCAATAAAAGAGTGCATATGTATCAA
AACCGTGGAAACCATTTATCCGATATGCTATTCCATATATAATCGTATATTGTATACTTCAT
GAATCAATAAAAAATTTACAATTTTTAATTGTAAACCTGCATTTTCAATGAAAATTATTTCTT
GCATGAATTTTGG

426. >MSTRG.8919.1 Gene=MSTRG.8919
ATTTCTTACCGATCTTTCTCCACACTGCAGAGCCGCGGGTCGGTGTCTATGACCGAATGACT
CTTGTGCGGCAAAGCGACCTATAGGGGGACGCTGGTGACCTGTGTGCATGGCTTGCCATGT
GTACTTGTACGACTGAGCATCAACTTTTAGCCTCGAGCGATCGGGCATTTCCAACACCTAA
GGATATTAATTATAAATCCTTTAGGGCGTACGGGCGTGTCGAATAGTTCTTTTCGACTTGCA
ATACGGCACGTTTCGTATGATGAGCTTTGAGTTTGTAAAAGAGAAATGAGAGAGCACGCGG
CAGACCCTCGGTCGCCTGGCCGGCTGCACGACGGTACTCGCACGGTATCGGGCCGCAACCA
ATCCATTTTTTCGAATGTGTGTGCGTCAGGCCCGCCGCAAGCTCGGTCAGTTCTTACCCGGA

GGCACGGACCTGGTGCCGTCCCCGGGCCTGGCCAGCTGTTAGCAGGCGGTGTCCTTGGACT
GGCCAAGACTCGAATTACCGGTCGGCGACGCTATTGCTTTGGGTACTCTCAGGACCCGTCTT
GAAACACGGACCAAGGAGTCTAACATGTGCGCGAGTCATTGGGATGCAAAAACCTAAAGG
CGAAATGAAAGTGAAGATCGGACTTTGTGCCGATCGAGGGAGGA

427. >MSTRG.8921.1 Gene=MSTRG.8921
CAGCGGGTAATCTCGCCTACTCTGAGGTCGTCTTTCTACGTATAAGCGAAAAATATATTTTC
GCGACATGTAGCGTGTACCAATGAGAAGAATAAGAAAGAAAGTTAACGAGAGAAGGTTAT
TTTTTCTCTCCTTTCCTTTTCCATCCCTCTTTTTAAAAATTAAATTCGAATAGAGGGAAGAAA
AAAAAGAGAAAAAAAAAAAAAATCGAAATGGAAGAAGAATTTTTCTCACTCGAATATTACC
CACCATCGTATTACCCCCTTGACTTCGCTAGATATATCTCTCTTTTCTTTCTTTTTTTCAAAAA
AGCAACAAGAGTATTATATACATTGCGAAGAAGAAGAATATTCCTCCTTTTAGCAATCGTA
GTGTTTTAAATATGCACCGCACCATAGTGCCAATTATCGTATGTCGTATTCATGTTAGAGCC
AAGACTCACGCAAGATATCCGAATGTCGGTTTCGTTTTAACGCCCGTCCTCTATTTTAGCTTC
CCGTTGACAATTTCCAGCGGTTTCTACTACTTTTTTTAAAAAACCTCGGGTAAAGTGAGGTA
AAGTGAGAAACTCACGCAAGGAAAAATATCACGAGAGCCTTTTCCGGGGACTGGACGACC
GACATGGATACGCGGTCCGGCGATAGACAACGAAAAAGGCATGGGATGACCAACACTAG
ATTCAGGAATTTCTCCTCGGCGCGGAGATGCCATAATTTGTCGTCTCGAAAAGAAGAAAAA
AAAAAAAAAAAAGAAAAAGAATGAAAGAGCCTCGCCAAAGAGAATCGTACTCGTCCTCA
TCTTACGTATCCCTTATATCCTCGTGGAGTCTTCGATCTCTGATACGAAAACTGGTATCGCTT
AAGGACTCGCGTGCACGTTGATGTGACGGTCCAGCGACAGTTCCGACAGAGTATGACAAA
CACGAAGGAACGGAGTGGGTATTCCGATTCCTGGGGCTCTTTTCTCTCTCTCTCTCTCTCT
CTCTCTTTCTCACAATCTCAACGCACGGCAAACATCGCACGCCATCAAGGGATGGTGGGAT
CTTTCTTCCCTCCTCTCTCGATTAAACTCCCATATATTTTTATCCTTTTTCTCACGGTATCGCA
GAAATATTAGTTTCAGACGACGTTAGGGGCGCGGTATGTAATTCGTGATCCAAAAATGGAC
GAATCGAAACAGTGGCGGGTACGAAAAACAAGCACGAAGCGGTGAACACGGGCGGTCAT
CAGTTATATGAAACGGACGACTCACGACACCGAGAACACTCACGCAAGTCCGTGACATGT
CTTGAATTCGTGAATACGATTTCCTCGTACCACACGCGAGCACATGTATTTCGATTCTGTTAT
CTCGGTCACGACCGCGACGAACGCCAACGAACGTCCAACAATCGCTCGTACGTCGCGCAT
CTTCTTCTCCTTATAATTTGTCCAACTCCGATTTTTGACACCCGACTCGGAAACGTTCGATTT
TGTGTCTGGTAAAATTCAAAAATATATAAATTCAAAAAAAAAAAAAAATGATAAATCAAT
CGGATACAACGGACGGAAGGGATTTTTCTCTCCCTCCTCACGTAAGCCATCGTTCCGTGATT
TCATCATTCGTTCATATGTTCGATATACAAAGTTTTGGTATTTGATAATGGATTCAAAAAATC
CATTTTATAAATATATATATAAAATATATATATTTATCATCCCCTTCTTTCAATTCTCTGTTTG
TTTTGTTACGCATTTTCTCTTTTTGTATGTTTCATTCGTTAATGATCCTTCCGCAGG

428. >MSTRG.8927.1 Gene=MSTRG.8927
TCGAACGCACATTGCGGTCCACGGATACAATTCCTGGACCACGCCTGGCTGAGGGTCGTTC
ACGTAACCCAAATACTGCTTGCGTTGCGTATTGGTTATTCTTCGTACATTTGATCCTTTTCTC
CTTCTTTTTGTAATCCCCGCCGTCGTTCTTAAAATCGAACGTTTCCGAGTCGGGTGTCAAAA
ATCGGAGTTGGACAAATTATAAGGAGAAGAAGATGCGCGACGTACGAGCGATTGTTGGAC
GTTCGTTGGCGTTCGTCGCGGTCGTGACCGAGATAACAGAATCGAAATACATGTGCTCGCG
TGTGGTACGAGGAAATCGTATTCACGAATTCAAGACATGTCACGGACTTGCGTGAGTGTTC

TCGGTGTCGTGAGTCGTCCGTTTCATATAACTGATGACCGCCCGTGTTCACCGCTTCGTGCTT
GTTTTTCGTACCCGCCACTGTTTCGATTCGTCCATTTTTGGATCACGAATTACATACCGCGCC
CCTAACGTCGTCTGAAACTAATATTTCTGCGATACCGTGAGAAAAAGGATAAAAATATATG
GGAGTTTAATCGAGAGAGGAGGGAAGAAAGATCCCACCATCCCTTGATGGCGTGCGATGT
TTGCCGTGCGTTGAGATTGTGAGAAAGAGAGAGAGAGAGAGAGAGAGAGAGAGAGAGAAAA
GAGCCCCAGGAATCGGAATACCCACTCCGTTCCTTCGTGTTTGTCATACTCTGTCGGAACTG
TCGCTGGACCGTCACATCAACGTGCACGCGAGTCCTTAAGCGATACCAGTTTTCGTATCAG
AGATCGAAGACTCCACGAGGATATAAGGGATACGTAAGATGAGGACGAGTACGATTCTCT
TTGGCGAGGCTCTTTCATTCTTTTTCTTTTTTTTTTTTTTTTTCTTCTTTTCGAGACGACAAATT
ATGGCATCTCCGCGCCGAGGAGAAATTCCTGAATCTAGTGTTGGTCATCCCATGCCTTTTTC
GTTGTCTATCGCCGGACCGCGTATCCATGTCGGTCGTCCAGTGTCCTAAGATCATACACACA
AACATATAAAAATTGAAATATGAGACCAGAAACACACCCATTGGGCGAAAGGGAATCCG
GTTCCTATTCCGGAACCCGGCAGCGGAACCGCATACCATTCGGGCCCTCGTAAGAGTGTTC
GTCGGGGTAACCCAAAATGACCTGGAGACGCCGTCGGGAGATCCGGGAAGAGTTTTCTTTT
CTGTATAAGCGTTCGAGTTCCCTGGAAACCTCTAGCAGGGAGATAGGGTTTGGAACGCGAA
GAGCACCGCAGTTGCGGCGGTGTCCGGATCTTCCCCTCGGACCTTGAAAATCCAGGAGAGG
GCCACGTGGAGGTGTCGCGCCGGTTCGTACCCATATCCGCAGCAGGTCTCCAAGGT

429. >MSTRG.8930.2 Gene=MSTRG.8930
GTCGTCTTTCTACGTATAAAGCGAAAAATATATTTTCGCGACATTGTAGCGTGTACCAATGA
GAAGAATAAGAAAGAAAGTTAACGAGAGAAGGTTATTTTTTCTCTCCTTTCCTTTTCCATCC
CTCTTTTTAAAAATTAAATTCGAATAGAGGGAAGAAAAAAAAGAGAAAAAAAAAAAAAA
TCGAAATGGAAGAAGAATTTTTCTCACTCGAATATTACCCACCATCGTATTACCCCCTTGAC
TTCGCTAGATATATCTCTCTTTTCTTTCTTTTTTTCCGGGGACTGGACGACCGACATGGATAC
GCGGTCCGGCGATAGACAACGAAAAAGGCATGGGATGACCAACACTAGATTCAGGAATTT
CTCCTCGGCGCGGAGATGCCATAATTTGTCGTCTCGAAAAGAAGAAAAAAAAAAAAAAAA
AAGAAAAAGAATGAAAGAGCCTCGCCAAAGAGAATCGTACTCGTCCTCATCTTACGTATC
CCTTATATCCTCGTGGAGTCTTCGATCTCTGATACGAAAACTGGTATCGCTTAAGGACTCGC
GTGCACGTTGATGTGACGGTCCAGCGACAGTTCCGACAGAGTATGACAAACACGAAGGAA
CGGAGTGGGTATTCCGATTCCTGGGGCTCTTTTCTCTCTCTCTCTCTCTCTCTCTCTCTCTTT
CTCACAATCTCAACGCACGGCAAACATCGCACGCCATCAAGGGATGGTGGGATCTTTCTTC
CCTCCTCTCTCGATTAAACTCCCATATATTTTTATCCTTTTTCTCACGGTATCGCAGAAATATT
AGTTTCAGACGACGTTAGGGGCGCGGTATGTAATTCGTGATCCAAAAATGGACGAATCGAA
ACAGTGGCGGGTACGAAAAACAAGCACGAAGCGGTGAACACGGGCGGTCATCAGTTATAT
GAAACGGACGACTCACGACACCGAGAACACTCACGCAAGTCCGTGACATGTCTTGAATTC
GTGAATACGATTTCCTCGTACCACACGCGAGCACATGTATTTCGATTCTGTTATCTCGGTCA
CGACCGCGACGAACGCCAACGAACGTCCAACAATCGCTCGTACGTCGCGCATCTTCTTCTC
CTTATAATTTGTCCAACTCCGATTTTTGACACCCGACTCGGAAACGTTCGATTTTAAGAACG
ACGGCGGGGATTACAAAAAGAAGGAGAAAAGGATCAAATGTACGAAGAATAACCAATAC
GCAACGCAAGCAGTATTTGGGTTACGTGAACGACCCTCAGCCAGGCGTGGTCCAGGAATTG
TATCCGTGGACCGCAATGTGCGTTCGAAATGTCGATGTTCATGTGTCCTGCAGTTCACACGT
TGACGCGCAATTAGCTGCGTTCTTC

430. >MSTRG.8930.1 Gene=MSTRG.8930
GTCGTCTTTCTACGTATAAAGCGAAAAATATATTTTCGCGACATTGTAGCGTGTACCAATGA
GAAGAATAAGAAAGAAAGTTAACGAGAGAAGGTTATTTTTTCTCTCCTTTCCTTTTCCATCC
CTCTTTTTAAAAATTAAATTCGAATAGAGGGAAGAAAAAAAAAGAGAAAAAAAAAAAAAA
TCGAAATGGAAGAAGAATTTTTCTCACTCGAATATTACCCACCATCGTATTACCCCCTTGAC
TTCGCTAGATTCCTCCTTTTAGCAATCGTAGTGTTTTAAATATGCACCGCACCATAGTGCCA
ATTTATCGTATGTCGTATTCATGTTAGAGCCAAGACTCACGCAAGATATCCGAATGTCGGTT
TCGTTTTAACGCCCGTCCTCTATTTTAGCTTCCCGTTGACAATTTCCAGCGGTTTCTACTACTT
TTTTTAAAAAACCTCGGGTAAAGTGAGGTAAAGTGAGAAACTCACGCAAGGAAAAATATC
ACGAGAGCCTTTTCCGGGGACTGGACGACCGACATGGATACGCGGTCCGGCGATAGACAA
CGAAAAAGGCATGGGATGACCAACACTAGATTCAGGAATTTCTCCTCGGCGCGGAGATGC
CATAATTTGTCGTCTCGAAAAGAAGAAAAAAAAAAAAAAAAAAAGAAAAAGAATGAAAGA
GCCTCGCCAAAGAGAATCGTACTCGTCCTCATCTTACGTATCCCTTATATCCTCGTGGAGTC
TTCGATCTCTGATACGAAAACTGGTATCGCTTAAGGACTCGCGTGCACGTTGATGTGACGGT
CCAGCGACAGTTCCGACAGAGTATGACAAACACGAAGGAACGGAGTGGGTATTCCGATTC
CTGGGGCTCTTTTCTCTCTCTCTCTCTCTCTCTCTCTCTCTCTTTCTCACAATCTCAACGCACG
GCAAACATCGCACGCCATCAAGGGATGGTGGGATCTTTCTTCCCTCCTCTCTCGATTAAACT
CCCATATATTTTTATCCTTTTTCTCACGGTATCGCAGAAATATTAGTTTCAGACGACGTTAGG
GGCGCGGTATGTAATTCGTGATCCAAAAATGGACGAATCGAAACAGTGGCGGGTACGAAA
AACAAGCACGAAGCGGTGAACACGGGCGGTCATCAGTTATATGAAACGGACGACTCACGA
CACCGAGAACACTCACGCAAGTCCGTGACATGTCTTGAATTCGTGAATACGATTTCCTCGT
ACCACACGCGAGCACATGTATTTCGATTCTGTTATCTCGGTCACGACCGCGACGAACGCCA
ACGAACGTCCAACAATCGCTCGTACGTCGCGCATCTTCTTCTCCTTATAATTTGTCCAACTC
CGATTTTTGACACCCGACTCGGAAACGTTCGATTTTAAGAACGACGGCGGGGATTACAAAA
AGAAGGAGAAAAGGATCAAATGTACGAAGAATAACCAATACGCAACGCAAGCAGTATTT
GGGTTACGTGAACGACCCTCAGCCAGGCGTGGTCCAGGAATTGTATCCGTGGACCGCAATG
TGCGTTCGAAATGTCGATGTTCATGTGTCCTGCAGTTCACACGTTGACGCGCAATTAGCTGC
GTTCTTCATCGACCCACGAGCCAAGTGATCCACCGTTCAGGGTAATCGTATTTTATGTAAAA
TTTTTTTTTTTTTCTTTACTTTTATAAAGGAAACGACGAGGATTACCCCGATACTGAATCCTTT
TTCTCTCTCTCTTCTCTTCAAACCGTCGCTCAACCTGGAGAGAACGACTCGTTCGAAGCATG
GTCGCAGAGCCCATACGCTTAAAAAAAAATTCGATGTTGAAAACTAATAATTCGTTCAGGTA
CGTATATCTCGATGAAAAGATTCGAAAAGATCGATGGGACACGAAAAATTCGATCGGTCTC
GAGCGGTCTTTCCTCCCAATTTCCGAAGCGAATATTCGCTACGGTACGGGTGTCCGACTCAC
GACACGACGACCTCTCTTCTCTCTCTGTGTAACACAGATTCGATATTCCCCTCTGAACGAAT
AACGTTTCAACTTTTTTAAGTAAGAAGCGATGAAACGGGCTCACGTCGACGCGATGCATCA
TTTTCTCGCCGTTCTTGGTTTCGTCGGTTTAAAGGTACAACGACGACGACGACGACACCATG
GGTAGGAAGCACGATTCGAGAATGATCATAGAAGCGTATAGGAGAAAAGAGGTCGAACT
GGTATCCATCGAAGAGAACGTCGCGAAACAAAGTTCGACGGCGGCTCGTCCATCCTTCGAT
TTCGTTTTACTATCGACGCGCTACTTGTCCCCTTATCGCTTTTTCTCTTTCTCATATCTCGACA
GCTTCGTTCGATCCCAAAGTCTTGGTGCGTTGCGTCTCCTCGTCCATCTCTCGTTTTTATATA
AGAGGAGAGCTCAGGCAACGAAAATAAAGAAGAAGAGAAATGCACAGAAATATGAACTT
GGTACTTTTACTGCACGTATCGATGTACATACACATACGTAACGCACGTATATACGTTCATA
AAGACGGCGGGTCTATTACAAAAGACCTCACGCAAGCGCCTTGTGTTGTGTCAAGCCATTT

ATCATTTTGTTTTTGTGTTTAGTTTCATTTTATATATATATATATATATTTTATATCAGTAGGGAC
GTAATGTTATCGAAGCGGCGGGTCGAATTTAAAAATTCGACCTCACGCAAGCGCTTCCTTTT
GATTATTATTTCGTATATATTATTATGCGTATACATACGTAATACGTACACGTACGTGCAAA
ACGTACCGTCAAATCTTTATTAAAAATATACATCATCGAGACGGCGGATCGTCCGTACATG
TGTGTATATATATATATATAAAATATATACGACGACGCGACGACGATCTCACGCAAGCGTC
CCTTTGTATTTTTGCGTACCATAACATTCATCTCATCGAGACGGCGGGTCTATTTATTTATAA
TAAACGACCTTCGCGCAAGCGCCTCTTCGTATTTATCGTTTATATATCTCTCTCTCTCTCGTG
GAAAGAGAAAGAATTTCTCTCTTCGTATCAAATTTTCGTGTCTGGTAAAATTCAAAAATATA
TAAATTCAAAAAAAAAAAAAAATGATAAATCAATCGGATACAACGGACGGAAGGGATTTT
TCTCTCCCTCCTCACGTAAGCCATCGTTCCGTGATTTCATCATTCGTTCATATGTTAGATATA
CAAAGTTTTGGTATTTGATAATGGATTCAAAAAATCCATTTTATAAATATATATATAAAATA
TATATATTTATCATCCCCTTCTTTCAATTCTCTGTTTGTTTTGTTACGCATTTTCTCTTTTTG

431. >MSTRG.8930.3 Gene=MSTRG.8930
TTTCTACGTATAAAGCGAAAAATATATTTTCGCGACATTGTAGCGTGTACCAATGAGAAGA
ATAAGAAAGAAAGTTAACGAGAGAAGGTTATTTTTTCTCTCCTTTCCTTTTCCATCCCTCTTT
TTAAAAATTAAATTCGAATAGAGGGAAGAAAAAAAAGAGAAAAAAAAAAAAAATCGAA
ATGGAAGAAGAATTTTTCTCACTCGAATATTACCCACCATCGTATTACCCCCTTGACTTCGC
TAGATATATCTCTCTTTTCTTTCTTTTTTTCAAAAAAGCAACAAGAGTATTATATACATTGCG
AAGAAGAAGAATATTCCTCCTTTTCCGGGGACTGGACGACCGACATGGATACGCGGTCCGG
CGATAGACAACGAAAAAGGCATGGATGACCAACACTAGATTCAGGAATTTCTCCTCGGC
GCGGAGATGCCATAATTTGTCGTCTCGAAAAGAAGAAAAAAAAAAAAAAAAAAGAAAAA
GAATGAAAGAGCCTCGCCAAAGAGAATCGTACTCGTCCTCATCTTACGTATCCCTTATATCC
TCGTGGAGTCTTCGATCTCTGATACGAAAACTGGTATCGCTTAAGGACTCGCGTGCACGTTG
ATGTGACGGTCCAGCGACAGTTCCGACAGAGTATGACAAACACGAAGGAACGGAGTGGGT
ATTCCGATTCCTGGGGCTCTTTTCTCTCTCTCTCTCTCTCTCTCTCTCTCTTTCTCACAATCT
CAACGCACGGCAAACATCGCACGCCATCAAGGGATGGTGGGATCTTTCTTCCCTCCTCTCTC
GATTAAACTCCCATATATTTTTATCCTTTTTCTCACGGTATCGCAGAAATATTAGTTTCAGAC
GACGTTAGGGGCGCGGTATGTAATTCGTGATCCAAAAATGGACGAATCGAAACAGTGGCG
GGTACGAAAAACAAGCACGAAGCGGTGAACACGGGCGGTCATCAGTTATATGAAACGGA
CGACTCACGACACCGAGAACACTCACGCAAGTCCGTGACATGTCTTGAATTCGTGAATACG
ATTTCCTCGTACCACACGCGAGCACATGTATTTCGATTCTGTTATCTCGGTCACGACCGCGA
CGAACGCCAACGAACGTCCAACAATCGCTCGTACGTCGCGCATCTTCTTCTCCTTATAATTT
GTCCAACTCCGATTTTTGACACCCGACTCGGAAACGTTCGATTTTAAGAACGACGGCGGGG
ATTACAAAAAGAAGGAGAAAAGGATCAAATGTACGAAGAATAACCAATACGCAACGCAA
GCAGTATTTGGGTTACGTGAACGACCCTCAGCCAGGCGTGGTCCAGGAATTGTATCCGTG

432. >MSTRG.8931.1 Gene=MSTRG.8931
ACCTGCGGAAGGATCATTAACGAATGAAACATACAAAAAGAGAAAATGCGTAACAAAAC
AAACAGAGAATTGAAAGAAGGGGATGATAAATATATATATTTTATATATATATTTATAAAA
TGGATTTTTTGAATCCATTATCAAATACCAAAACTTTGTATATCTAACATATGAACGAATGA
TGAAATCACGGAACGATGGCTTACGTGAGGAGGGAGAGAAAAATCCCTTCCGTCCGTTGTA
TCCGATTGATTTATCATTTTTTTTTTTTTTTGAATTTATATATTTTTGAATTTTACCAGACACGA

AAATTTGATACGAAGAGAGAAATTCTTTCTCTTTCCACGAGAGAGAGAGAGATATATAAAC
GATAAATACGAAGAGGCGCTTGCGCGAAGGTCGTTTATTATAAATAAATAGACCCGCCGTC
TCGATGAGATGAATGTTATGGTACGCAAAAATACAAAGGGACGCTTGCGTGAGATCGTCGT
CGCGTCGTCGTATATATTTTATATATATATATATACACACATGTACGGACGATCCGCCGTCT
CGATGATGTATATTTTTAATAAAGATTTGACGGTACGTTTTGCACGTACGTGTACGTATTAC
GTATGTATACGCATAATAATATATACGAAATAATAATCAAAAGGAAGCGCTTGCGTGAGGT
CTTTTGTAATAGACCCGCCGTCTTTATGAACGTATATACGTGCGTTACGTATGTGTATGTACA
TCGATACGTGCAGTAAAAGTACCAAGTTCATATTTCTGTGCATTTCTCTTCTTCTTTATTTTCG
TTGCCTGAGCTCTCCTCTTATATAAAAACGAGAGATGGACGAGGAGACGCAACGCACCAA
GACTTTGGGATCGAACGAAGCTGTCGAGATATGAGAAAGAGAAAAAGCGATAAGGGGAC
AAGTAGCGCGTCGATAGTAAAACGAAATCGAAGGATGGACGAGCCGCCGTCGAACTTTGT
TTCGCGACGTTCTCTTCGATGGATACCAGTTCGACCTCTTTTCTCCTATACGCTTCTATGATC
ATTCTCGAATCGTGCTTCCTACCCATGGTGTCGTCGTCGTCGTCGTTGTACCTTTAAACCGAC
GAAACCAAGAACGGCGAGAAAATGATGCATCGCGTCGACGTGAGCCCGTTTCATCGCTTCT
TACTTAAAAAAGTTGAAACGTTATTCGTTCAGAGGGGAATATCGAATCTGTGTTACACAGA
GAGAGAAGAGAGGTCGTCGTGTCGTGAGTCGGACACCCGTACCGTAGCGAATATTCGCTTC
GGAAATTGGGAGGAAAGACCGCTCGAGACCGATCGAATTTTTCGTGTCCCATCGATCTTTT
CGAATCTTTTCATCGAGATATACGTACCTGAACGAATTATTAGTTTTCAACATCGAATTTTTT
TTAAGCGTATGGGCTCTGCGACCATGCTTCGAACGAGTCGTTCTCTCCAGGTTGAGCGACGG
TTTGAAGAGAAGAGAGAGAGAAAAAGGATTCAGTATCGGGGTAA

Table S7. Information of target genes regulated by DElncRNAs from the intersection of NDB-vs-EDB and NDB-vs-LDB via the cis-regulatory mechanism.

lncRNA_ID	Gene_ID	up/down_Stream	distance	Symbol	Description
XR_003304701.1	ncbi_408855	DOWNSTREAM	4373	Esrra	PREDICTED: estrogen-related receptor isoform X1 [Apis mellifera]
XR_003304701.1	ncbi_100578476	UPSTREAM	1368	--	PREDICTED: uncharacterized protein LOC100578476 [Apis mellifera]
XR_001703122.2	ncbi_102656766	DOWNSTREAM	5850	ato	PREDICTED: ETS-like protein pointed [Apis mellifera]
XR_001703122.2	ncbi_100577107	UPSTREAM	5682	--	PREDICTED: uncharacterized protein LOC100577107 [Apis mellifera]
XR_001703122.2	ncbi_725215	UPSTREAM	5704	ACPP	PREDICTED: venom acid phosphatase Acph-1-like [Apis mellifera]
XR_003305156.1	ncbi_724225	UPSTREAM	2975	SPR	PREDICTED: sex peptide receptor-like isoform X1 [Apis mellifera]
XR_003305156.1	ncbi_411290	UPSTREAM	8091	SOCS3	Suppressor of cytokine signalling 2 [Melipona quadrifasciata]
XR_003305156.1	ncbi_412161	DOWNSTREAM	1089	br	PREDICTED: nucleus accumbens-associated protein 2-like isoform X1 [Bombus terrestris]
XR_003305473.1	ncbi_725877	DOWNSTREAM	2901	bca-1	PREDICTED: beta carbonic anhydrase 1 isoform X1 [Apis mellifera]
XR_003305473.1	ncbi_724408	DOWNSTREAM	6133	Rraga	PREDICTED: ras-related GTP-binding protein A [Apis mellifera]
MSTRG.6803.3	ncbi_725781	UPSTREAM	4161	Vps50	PREDICTED: syndetin [Apis mellifera]
MSTRG.6803.3	ncbi_409241	DOWNSTREAM	259	EAG_01737	PREDICTED: prohormone-4 [Apis mellifera]
MSTRG.6803.3	ncbi_411861	DOWNSTREAM	390	Nkrf	PREDICTED: NF-kappa-B-repressing factor [Apis mellifera]
MSTRG.6803.3	ncbi_411862	UPSTREAM	3218	MRPS2	PREDICTED: 28S ribosomal protein S2, mitochondrial [Apis mellifera]
MSTRG.6803.3	ncbi_411863	DOWNSTREAM	4737	DHRS7	PREDICTED: dehydrogenase/reductase SDR family member 7 [Apis mellifera]
MSTRG.6803.3	ncbi_409636	DOWNSTREAM	7656	--	PREDICTED: phospholipid scramblase 1-like isoform X1 [Eufriesea mexicana]
MSTRG.6803.3	ncbi_409634	UPSTREAM	9434	--	PREDICTED: LOW QUALITY PROTEIN: protein PFC0760c [Apis florea]

Note: EDB referred to the waggling dancers after foraging on a feeder with 1.5 M sucrose solution in the first phase. NDB referred to the non-dancing bees after foraging on another feeder with 0.5 M sucrose solution replacing the feeder with 1.5 M sucrose solution in the second phase. LDB referred to the waggling dancers after foraging on the feeder with 1.5 M sucrose solution replacing the feeder with 0.5 M sucrose solution in the third phase.

Table S8. Information of target genes regulated by DElncRNAs from the intersection of QB-vs-EDB and QB-vs-LDB via the cis-regulatory mechanism.

lncRNA_ID	Gene_ID	up/down_Stream	distance	Symbol	Description
XR_001706449.2	ncbi_107966053	DOWNSTREAM	359	--	PREDICTED: uncharacterized protein LOC107966053 isoform X1 [Apis mellifera]
XR_001706449.2	ncbi_412412	UPSTREAM	3317	Mfap1a	PREDICTED: microfibrillar-associated protein 1-like isoform X1 [Apis dorsata]
XR_001706449.2	ncbi_726766	DOWNSTREAM	5242	dicer1	PREDICTED: LOW QUALITY PROTEIN: endoribonuclease Dicer [Apis mellifera]
XR_001706449.2	ncbi_725976	DOWNSTREAM	61	FKBP46	PREDICTED: 46 kDa FK506-binding nuclear protein [Apis mellifera]
MSTRG.1195.1	ncbi_412003	UPSTREAM	4307	RasGAP1	PREDICTED: LOW QUALITY PROTEIN: ras GTPase-activating protein 3 [Apis mellifera]
MSTRG.1195.1	ncbi_727456	DOWNSTREAM	4590	YMR099C	PREDICTED: glucose-6-phosphate 1-epimerase [Apis mellifera]
NR_003565.1	ncbi_408663	UPSTREAM	8140	SCM-1	PREDICTED: soma ferritin [Apis mellifera]
NR_003565.1	ncbi_410815	UPSTREAM	5705	wupA	PREDICTED: troponin I isoform X1 [Apis mellifera]
XR_003304726.1	ncbi_408827	DOWNSTREAM	4023	ca2	PREDICTED: carbonic anhydrase 1 [Apis mellifera]
XR_003305156.1	ncbi_724225	UPSTREAM	2975	SPR	PREDICTED: sex peptide receptor-like isoform X1 [Apis mellifera]
XR_003305156.1	ncbi_411290	UPSTREAM	8091	SOCS3	Suppressor of cytokine signalling 2 [Melipona quadrifasciata]
XR_003305156.1	ncbi_412161	DOWNSTREAM	1089	br	PREDICTED: nucleus accumbens-associated protein 2-like isoform X1 [Bombus terrestris]
MSTRG.6803.3	ncbi_725781	UPSTREAM	4161	Vps50	PREDICTED: syndetin [Apis mellifera]
MSTRG.6803.3	ncbi_409241	DOWNSTREAM	259	EAG_01737	PREDICTED: prohormone-4 [Apis mellifera]
MSTRG.6803.3	ncbi_411861	DOWNSTREAM	390	Nkrf	PREDICTED: NF-kappa-B-repressing factor [Apis mellifera]
MSTRG.6803.3	ncbi_411862	UPSTREAM	3218	MRPS2	PREDICTED: 28S ribosomal protein S2, mitochondrial [Apis mellifera]
MSTRG.6803.3	ncbi_411863	DOWNSTREAM	4737	DHRS7	PREDICTED: dehydrogenase/reductase SDR family member 7 [Apis mellifera]
MSTRG.6803.3	ncbi_409636	DOWNSTREAM	7656	--	PREDICTED: phospholipid scramblase 1-like isoform X1 [Eufriesea mexicana]
MSTRG.6803.3	ncbi_409634	UPSTREAM	9434	--	PREDICTED: LOW QUALITY PROTEIN: protein PFC0760c [Apis florea]

Note: EDB referred to the waggling dancers after foraging on a feeder with 1.5 M sucrose solution in the first phase. LDB referred to the waggling dancers after foraging on the feeder with 1.5 M sucrose solution replacing the feeder with 0.5 M sucrose solution in the third phase. QB referred to quiescent bees on the comb after removing the feeder for 1 hour in the fourth phase.

Table S9. Top 20 of GO terms enrichment analysis of target genes regulated by DElncRNAs from the intersection between QB-vs-EDB and QB-vs-LDB via the cis-regulatory mechanism.

GO term (level 2)	number of genes	P-value	Gene ID
iron ion binding	1	0.00366888	ncbi_408663
cellular transition metal ion homeostasis	1	0.003985824	ncbi_408663
transition metal ion homeostasis	1	0.005312075	ncbi_408663
cellular chemical homeostasis	1	0.006637147	ncbi_408663
cellular cation homeostasis	1	0.006637147	ncbi_408663
cellular metal ion homeostasis	1	0.006637147	ncbi_408663
cellular ion homeostasis	1	0.006637147	ncbi_408663
metal ion homeostasis	1	0.009283761	ncbi_408663
transition metal ion transport	1	0.009283761	ncbi_408663
inorganic ion homeostasis	1	0.013244862	ncbi_408663
cation homeostasis	1	0.013244862	ncbi_408663
ion homeostasis	1	0.013244862	ncbi_408663
chemical homeostasis	1	0.013244862	ncbi_408663
cellular homeostasis	1	0.023756147	ncbi_408663
homeostatic process	1	0.032892061	ncbi_408663
metal ion transport	1	0.041970798	ncbi_408663
regulation of GTPase activity	1	0.045844228	ncbi_412003
regulation of biological quality	1	0.053559736	ncbi_408663
regulation of hydrolase activity	1	0.059957464	ncbi_412003
regulation of catalytic activity	1	0.068865751	ncbi_412003

Note: EDB referred to the waggling dancers after foraging on a feeder with 1.5 M sucrose solution in the first phase. LDB referred to the waggling dancers after foraging on the feeder with 1.5 M sucrose solution replacing the feeder with 0.5 M sucrose solution in the third phase. QB referred to quiescent bees on the comb after removing the feeder for 1 hour in the fourth phase.

Table S10. Top 20 of KEGG pathway enrichment analysis of target genes regulated by DElncRNAs from the intersection between QB-vs-EDB and QB-vs-LDB via the cis-regulatory mechanism.

Pathway	number of genes	P-value	Gene ID
Nitrogen metabolism	1	0.02306483	ncbi_408827
Ferroptosis	1	0.03065387	ncbi_408663
Mineral absorption	1	0.03631336	ncbi_408663
Necroptosis	1	0.08782041	ncbi_408663
Glycolysis / Gluconeogenesis	1	0.09140989	ncbi_727456
MicroRNAs in cancer	1	0.1109424	ncbi_726766
Ras signalling pathway	1	0.126662	ncbi_412003
MAPK signalling pathway - fly	1	0.14044	ncbi_412003

Ribosome	1	0.2130234	ncbi_411862
Metabolic pathways	1	0.8345884	ncbi_727456

Note: EDB referred to the waggling dancers after foraging on a feeder with 1.5 M sucrose solution in the first phase. LDB referred to the waggling dancers after foraging on the feeder with 1.5 M sucrose solution replacing the feeder with 0.5 M sucrose solution in the third phase. QB referred to quiescent bees on the comb after removing the feeder for 1 hour in the fourth phase.

Table S11. Top 20 of GO terms enrichment analysis of target genes regulated by DElncRNAs in QB-vs-NDB via the cis-regulatory mechanism.

GO term (level 2)	number of genes	P-value	Gene ID
sulfate transport	2	0.000529056	ncbi_413934; ncbi_724952
sulfur compound transport	2	0.00073805	ncbi_413934; ncbi_724952
inorganic anion transport	2	0.001905713	ncbi_413934; ncbi_724952
inorganic anion transmembrane transporter activity	2	0.002198829	ncbi_413934; ncbi_724952
glutamine family amino acid biosynthetic process	1	0.006205674	ncbi_408859
ER-nucleus signalling pathway	1	0.006205674	ncbi_411459
proline biosynthetic process	1	0.006205674	ncbi_408859
lipid binding	2	0.006448893	ncbi_408855; ncbi_411459
alcohol binding	1	0.007342818	ncbi_411459
sterol binding	1	0.007342818	ncbi_411459
steroid binding	1	0.007342818	ncbi_411459
anion transport	2	0.00769749	ncbi_413934; ncbi_724952
anion transmembrane transporter activity	2	0.008894474	ncbi_413934; ncbi_724952
sterol metabolic process	1	0.012375572	ncbi_411459
cellular transition metal ion homeostasis	1	0.018509885	ncbi_408663
steroid metabolic process	1	0.018509885	ncbi_411459
macromolecule catabolic process	2	0.018934091	ncbi_409985; ncbi_550794
organic substance catabolic process	2	0.022134527	ncbi_409985; ncbi_550794
transition metal ion homeostasis	1	0.024608803	ncbi_408663
proline metabolic process	1	0.024608803	ncbi_408859

Note: NDB referred to the non-dancing bees after foraging on another feeder with 0.5 M sucrose solution replacing the feeder with 1.5 M sucrose solution in the second phase. QB referred to quiescent bees on the comb after removing the feeder for 1 hour in the fourth phase.

Table S12. Top 20 of KEGG pathway enrichment analysis of target genes regulated by DElncRNAs in QB-vs-NDB via the cis-regulatory mechanism.

Pathway	number of genes	P-value	Gene ID
Nitrogen metabolism	1	0.04564107	ncbi_725877
Ferroptosis	1	0.06042512	ncbi_408663

Mineral absorption	1	0.07137506	ncbi_408663
beta-Alanine metabolism	1	0.0822079	ncbi_552823
Propanoate metabolism	1	0.08936531	ncbi_552823
Endocrine and other factor-regulated calcium reabsorption	1	0.0929248	ncbi_411147
Arginine and proline metabolism	1	0.0929248	ncbi_408859
Fanconi anemia pathway	1	0.1140152	ncbi_411121
Bacterial invasion of epithelial cells	1	0.1346553	ncbi_724158
Synaptic vesicle cycle	1	0.1380521	ncbi_411147
Shigellosis	1	0.1380521	ncbi_724158
Proteasome	1	0.1481694	ncbi_550794
Necroptosis	1	0.1680782	ncbi_408663
Progesterone-mediated oocyte maturation	1	0.1746191	ncbi_724158
Biosynthesis of amino acids	1	0.1811128	ncbi_408859
RNA degradation	1	0.2066221	ncbi_552826
Peroxisome	1	0.2159988	ncbi_552823
AMPK signalling pathway	1	0.2191017	ncbi_552823
Oocyte meiosis	1	0.2314011	ncbi_724158
Cell cycle	1	0.2701582	ncbi_724158

Note: NDB referred to the non-dancing bees after foraging on another feeder with 0.5 M sucrose solution replacing the feeder with 1.5 M sucrose solution in the second phase. QB referred to quiescent bees on the comb after removing the feeder for 1 hour in the fourth phase.
